# Design of New Potent and Selective Thiophene-Based K_V_1.3 Inhibitors and Their Potential for Anticancer Activity

**DOI:** 10.3390/cancers14112595

**Published:** 2022-05-24

**Authors:** Špela Gubič, Louise Antonia Hendrickx, Xiaoyi Shi, Žan Toplak, Štefan Možina, Kenny M. Van Theemsche, Ernesto Lopes Pinheiro-Junior, Steve Peigneur, Alain J. Labro, Luis A. Pardo, Jan Tytgat, Tihomir Tomašič, Lucija Peterlin Mašič

**Affiliations:** 1 Faculty of Pharmacy, University of Ljubljana, Aškerčeva 7, 1000 Ljubljana, Slovenia; spela.gubic@ffa.uni-lj.si (Š.G.); zan.toplak@ffa.uni-lj.si (Ž.T.); stefan.mozina@ffa.uni-lj.si (Š.M.); tihomir.tomasic@ffa.uni-lj.si (T.T.); 2 Campus Gasthuisberg, University of Leuven, Toxicology and Pharmacology, Onderwijs en Navorsing 2, Herestraat 49, 3000 Leuven, Belgium; louise.hendrickx@kuleuven.be (L.A.H.); ernesto.lopes@kuleuven.be (E.L.P.-J.); steve.peigneur@kuleuven.be (S.P.); jan.tytgat@kuleuven.be (J.T.); 3 AG Oncophysiology, Max-Planck Institute for Multidisciplinary Sciences, Hermann-Rein-Str. 3, 37075 Göttingen, Germany; shi@mpinat.mpg.de (X.S.); pardo@mpinat.mpg.de (L.A.P.); 4 Laboratory for Molecular, Cellular and Network Excitability, Department of Biomedical Sciences, University of Antwerp, 2610 Wilrijk, Belgium; kenny.vantheemsche@uantwerpen.be (K.M.V.T.); alain.labro@ugent.be (A.J.L.); 5 Department of Basic and Applied Medical Sciences, Ghent University, Corneel Heymanslaan 10 (Entrance 36), 9000 Ghent, Belgium

**Keywords:** K_V_1.3, potassium ion channels, antiproliferative activity, apoptosis, anticancer drugs

## Abstract

**Simple Summary:**

In this article, we describe the discovery of a new class of potent and selective thiophene-based inhibitors of the voltage-gated potassium channel K_V_1.3 and their potential to induce apoptosis and inhibit proliferation. The K_V_1.3 channel has only recently emerged as a molecular target in cancer therapy. The most potent K_V_1.3 inhibitor **44** had an IC_50_ K_V_1.3 value of 470 nM (oocytes) and 950 nM (Ltk^−^ cells) and appropriate selectivity for other K_V_ channels. New K_V_1.3 inhibitors significantly inhibited proliferation of Panc-1 cells and K_V_1.3 inhibitor **4** induced significant apoptosis in tumor spheroids of Colo-357 cells.

**Abstract:**

The voltage-gated potassium channel K_V_1.3 has been recognized as a tumor marker and represents a promising new target for the discovery of new anticancer drugs. We designed a novel structural class of K_V_1.3 inhibitors through structural optimization of benzamide-based hit compounds and structure-activity relationship studies. The potency and selectivity of the new K_V_1.3 inhibitors were investigated using whole-cell patch- and voltage-clamp experiments. 2D and 3D cell models were used to determine antiproliferative activity. Structural optimization resulted in the most potent and selective K_V_1.3 inhibitor **44** in the series with an IC_50_ value of 470 nM in oocytes and 950 nM in Ltk^−^ cells. K_V_1.3 inhibitor **4** induced significant apoptosis in Colo-357 spheroids, while **14**, **37**, **43**, and **44** significantly inhibited Panc-1 proliferation.

## 1. Introduction

The voltage-gated potassium channel K_V_1.3 is a transmembrane protein belonging to the K_V_1 (Shaker) subfamily, which is the largest subfamily of voltage-gated potassium channels with eight members (K_V_1.1–K_V_1.8). K_V_1.3 is formed by four monomers, each containing six transmembrane segments (S1–S6) [1]. The S5–S6 domain of each monomer assembles to form the K^+^ conducting pore that is surrounded by four voltage sensor domains (VSD), each consisting of segments S1 to S4. The VSDs detect depolarization of the membrane and initiate conformational changes that lead to the opening of the pore. The selectivity filter in the K^+^ conducting pore consists of oxygen cages that mimic the hydration shell of potassium in solution and allow the rapid and selective passage of potassium ions [2,3,4].

The K_V_1.3 channel has only recently emerged as a molecular target in cancer therapy. Cancer cells display mutations enabling extensive proliferation and resistance to apoptosis. K_V_1.3 channel activity has been found to be involved in cell proliferation, migration, and invasion, which are among the most important processes in cancer progression. Moreover, overexpression of K_V_1.3 enhances tumorigenic processes and promotes tumor progression [5,6,7,8]. The use of small-molecule K_V_1.3 inhibitors could selectively suppress the proliferation of cancer cells, thus providing a new potential therapeutic approach [9]. Although K_V_1.3 has been recognized as a tumor marker in cancer tissues with predominantly higher K_V_1.3 expression, a clear pattern of altered K_V_1.3 expression in cancer cells compared with healthy cells has not yet been found. The type and stage of disease also influence K_V_1.3 expression. Up-regulated K_V_1.3 expression was detected in breast, colon, and prostate tumors, in smooth muscle and skeletal muscle cancers, and in mature neoplastic B cells in chronic lymphocytic leukemia [10].

**Figure 1:**
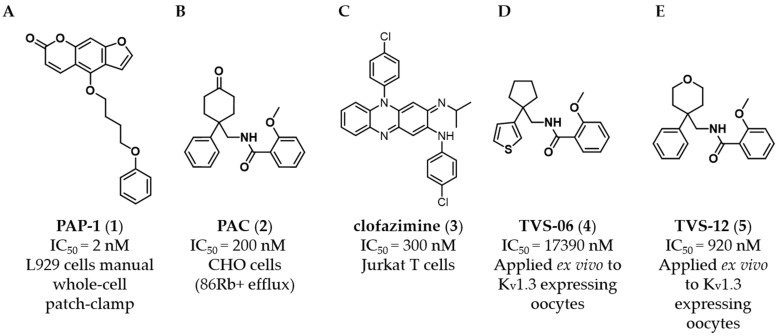
Structures of known representative K_V_1.3 inhibitors. (**A**) PAP-1 (**1**). (**B**) PAC (**2**). (**C**) Clofazimine (**3**) (**D**) TVS-06 (**4**). (**E**) TVS-12 (**5**).

To date, several small-molecule K_V_1.3 inhibitors have been discovered (Figure 1) [11,12,13]. Originally, K_V_1.3 inhibitors were extensively studied to selectively target the proliferation of effector memory T (T_EM_) cells in the immune system and to develop a new therapy for T-cell—mediated autoimmune and chronic inflammatory diseases [14]. However, none of these compounds have been optimised to have suitable properties for clinical trials. Since K_V_1 family channels have high subtype homology, it is very difficult to find a potent isoform-selective K_V_1.3 inhibitor [15]. For most of the described compounds, only low or moderate selectivity towards other Kv1.x channels was achieved, preventing subsequent optimization. Cryogenic electron microscopy (cryoEM) was recently used to determine the structures of human K_V_1.3 alone and bound to dalazatide, which may aid the structure-based design of new inhibitors in the future. To date, the exact binding site for most known K_V_1.3 inhibitors has not been experimentally demonstrated, and these new cryoEM structures may help to solve this problem [16,17].

The psoralen derivative PAP-1 (**1**, Figure 1A) is the most potent (IC_50_ = 2 nM, L929 cells, manual whole-cell patch clamp) and selective (i.e., 23-fold over K_V_1.5) small-molecule K_V_1.3 inhibitor known [11]. Benzamide PAC (**2**, Figure 1B), which inhibits K_V_1.3 with an IC_50_ of 200 nM (CHO cells, ^86^Rb^+^ efflux), is not selective toward K_V_1.x family channels [12]. The antimycobacterial drug clofazimine (**3**, Figure 1C,) is also a K_V_1.3 inhibitor (IC_50_ = 300 nM, Jurkat T cells), inhibiting K_V_1.3 with tenfold higher potency than K_V_1.1, K_V_1.2, K_V_1.5 and K_V_3.1 [13]. PAP-1 (**1**) at a concentration of 20 µM, and clofazimine (**3**) at a concentration of 1 or 10 µM in combination with the multi-drug resistance (MDR) pump inhibitors (MDRi) cyclosporine H (4 µM) and probenecid (100 µM), which induced mild apoptosis in cancer cells (human SAOS2, mouse B16F10 melanoma, and human B-CLL) by specifically inhibiting K_V_1.3. Similarly, clofazimine (**3**) induced significant apoptosis in several pancreatic ductal adenocarcinoma (PDAC) cell lines with EC_50_ values in the micromolar range and significantly reduced primary tumor weight in an orthotopic PDAC xenotransplantation model in SCID beige mice [18,19,20].

In previous work, we identified two new K_V_1.3 inhibitors by virtual screening based on a 3D similarity search using the K_V_1.3 inhibitor PAC (**2**, Figure 1B) as a query [21]. Hit compound **4** (Figure 1D), with an IC_50_ of 17.4 µM, was a much weaker K_V_1.3 inhibitor than hit compound **5** (Figure 1E), with an IC_50_ of 920 nM (applied ex vivo in *Xenopus* oocytes expressing K_V_1.3). However, Kv1.3 inhibitor **4** exhibited a good selectivity profile and did not affect other K_V_1.x family channels or more distantly related channels. In this paper, we describe the optimization of new thiophene-based Kv1.3 inhibitors, their selectivity profile against K_V_1.x and some other selected ion channels, and the potential of this new class of Kv1.3 inhibitors for antiproliferative activity in 2D and 3D cell models.

## 2. Materials and Methods

### 2.1. Oocyte Collection, Oocyte Injection, Drug Solutions, Electrophysiological Recordings, and Statistical Analysis in Xenopus Laevis Oocytes

Stage V–VI oocytes [21] were isolated by partial ovariectomy from *Xenopus laevis* frogs (African clawed frogs). Mature female frogs were purchased from CRB Xénopes (Rennes, France) and were housed in the Aquatic Facility (KU Leuven) in compliance with the regulations of the European Union (EU) concerning the welfare of laboratory animals as declared in Directive 2010/63/EU. The use of *Xenopus laevis* was approved by the Animal Ethics Committee of the KU Leuven (Project nr. P186/2019). After anaesthetizing the frogs by a 15-min submersion in 0.1% tricaine methanesulfonate (amino benzoic acid ethyl ester; Merck, Kenilworth, NJ, USA), pH 7.0, the oocytes were collected. The isolated oocytes were then washed with a 1.5 mg/mL collagenase solution for 2 h to remove the follicle layer.

Ion channels (Kv1.x, Kv2.1, Kv4.2, Kv10.1) were expressed in *Xenopus laevis* oocytes by linearization of the plasmids and subsequent in vitro transcription using a commercial T7 or SP6 mMESSAGE mMACHINE transcription kit (Ambion, Carlsbad, California, USA). Defolliculated *Xenopus* oocytes were then injected with 20–50 nL of the cRNA at a concentration of 1 ng/nL using a micro-injector (Drummond Scientific1, Broomall, PA, USA). The oocytes were incubated in a solution containing (in mM): NaCl, 96; KCl, 2; CaCl_2_, 1.8; MgCl_2_, 2 and HEPES, 5 (pH 7.5), supplemented with 50 mg/L gentamycin sulfate and 90 mg/L theophylline. After ex vivo translation, the ion channels were correctly inserted in the cell membrane of the oocytes.

Two-electrode voltage-clamp recordings were performed at room temperature (18–22 °C) using a Geneclamp 500 amplifier (Molecular Devices, San Jose, CA, USA) and pClamp data acquisition (Axon Instruments, Union City, CA, USA) and using an integrated digital TEVC amplifier controlled by HiClamp, an automated Voltage-Clamp Screening System (Multi Channel Systems MCS GmbH, Reutlingen, Germany). Whole-cell currents from oocytes were recorded 1–4 days after injection. The bath solution composition was ND96 (in mM): NaCl, 96; KCl, 2; CaCl_2_, 1.8; MgCl_2_, 2 and HEPES, 5 (pH 7.5). Voltage and current electrodes were filled with a 3 M solution of KCl in H_2_O. Resistances of both electrodes were kept between 0.5 and 1.5 MΩ. The elicited Kv1.x, Kv2.1, Kv4.2 and Kv10.1 currents were filtered at 0.5 kHz and sampled at 2 kHz using a four-pole low-pass Bessel filter. Leak subtraction was performed using a P/4 protocol [21].

For the electrophysiological analysis of the compounds, a number of protocols were applied from a holding potential of −90 mV. Currents for Kv1.x, Kv2.1, Kv4.2 and Kv10.1 were evoked by 1 s depolarizing pulses either to 0 mV or to a range of voltage steps between −80 mV and +40 mV. For the analysis of the data, only the inhibition at the 0 mV step was used. The concentration dependency of all compounds was assessed by measuring the current inhibition in the presence of increasing compound concentrations. To this end, a stock solution of the compounds was prepared in 100% DMSO for the sake of solubility. From this stock solution, adequate dilutions with a maximum of 0.5% DMSO were made for testing. The data of the concentration-response curves were fitted with the Hill equation: y = 100/{1 + (IC_50_/[compound])^h^}, where y is the amplitude of the compound-induced effect, IC_50_ is the compound concentration at half-maximal efficacy, [compound] is the compound concentration, and h is the Hill coefficient.

All electrophysiological data (Figure S1) are presented as means ± S.E.M of n ≥ three independent experiments unless otherwise indicated. All data were analyzed using pClamp Clampfit 10.4 (Molecular Devices, Downingtown, PA, USA), OriginPro 9 (Originlab, Northampton, MA, USA), GraphPad Prism 8 software (GraphPad Software, Inc., San Diego, CA, USA) and DataMining (Multi Channel Systems MCS GmbH, Reutlingen, Germany). The Dunnett test and one-way ANOVA were performed to calculate the significance of the induced inhibition compared to the control.

### 2.2. Cell Culture, Transfection, Drug Solutions, Electrophysiological Recordings, and Statistical Analysis in Ltk^−^ Cells

Ltk^−^ cells were cultured in Dulbecco’s Modified Eagle Medium (DMEM) supplemented with 10% horse serum and 1% penicillin/streptomycin (Invitrogen, Waltham, CA, USA). Human hK_V_1.3 channels were transiently expressed in these Ltk^−^ cells by transfecting subconfluent 60 × 15 mm cell culture dishes (Corning, NY, USA) with 1-1.5 µg plasmid DNA containing the hKv1.3 sequence (KCNA3) using lipofectamine 2000 (Thermo Fisher Scientific, Waltham, MA, USA), according to the manufacturer’s protocol. The coding sequence of hKv1.3 was cloned in a pEGFP plasmid without removing the stop codon (i.e., eGFP was not transcribed). Therefore, during transfection 0.5 µg of pEGFP plasmid (expressing eGFP) was added as transfection marker Cells were collected 24h post-transfection using a 0.05% trypsin-EDTA solution (Thermo Fisher Scientific, Waltham, MA, USA) and transferred to the recording chamber mounted on the stage of an inverted Nikon Eclipse TE2000 fluorescence microscope (Nikon, Minato, Japan). The compounds were dissolved in 100% DMSO and stock solutions were stored at -20°C. Before use, the stock concentrations were diluted with extracellular recording solution to appropriate concentrations, making sure that the final DMSO concentration never exceeded 0.1%. A 0.1% DMSO solution was prepared as a vehicle control.

All recordings were conducted at room temperature (20–23 °C) in the whole-cell configuration using an axopatch 200b amplifier (Molecular Devices, San Jose, CA, USA). Applied voltage pulse protocols and current recordings were controlled with pClamp 10 software and digitized using an axon digidata 1440 (Molecular Devices, San Jose, CA, USA). The cells in the recording chamber were continuously superfused with an extracellular solution (containing in mM): NaCl 145, KCl 4, MgCl_2_ 1, CaCl_2_ 1.8, HEPES 10, and glucose 10 (adjusted to pH 7.35 with NaOH). Patch pipettes were pulled from borosilicate glass (World Precision Instruments, Sarasota, FL, USA), using a P-2000 puller (Sutter Instruments, Novato, CA, USA), with resistances ranging from 1.5–2 MΩ. These pipettes were backfilled with an intracellular solution (containing in mM): KCl 110, K_2_ATP 5, MgCl_2_ 2, HEPES 10, and K_4_BAPTA 5 (adjusted to pH 7.2 with KOH). Junction potentials were zeroed with the filled pipette in the extracellular solution of the recording chamber. A series resistance compensation of 80% was employed. Recordings were passed through a 5 kHz low-pass filter while being sampled at 10 kHz. A single step from holding potential (−80 mV) to +40 mV was utilized to monitor K_V_1.3 current inhibition. The pulse duration was 200 ms with an interpulse interval of 15 s. As PAP-1 blocks K_V_1.3 in a use-dependent manner, by preferentially binding to the channels C-type inactivated state, the protocol was slightly modified: the pulse duration and interval between pulses were increased to 2 s and 30 s, respectively [11]. A longer pulse duration maximizes the number of channels in their inactivated state. Different concentrations of the compounds were independently added to the recording chamber in the vicinity of the cell investigated using a pressurized fast-perfusion system (custom built with electro-fluidic valves from the Lee Company, Westbrook, CT, USA).

The data was analyzed with pClamp 10 software (Molecular Devices, San Jose, CA, USA), and the dose-response curves shown were made using Sigmaplot 11.0 (Systat software, Palo Alto, CA USA). Data was excluded when the estimated voltage error exceeded 5mV after series resistance compensation. Dose-response curves were obtained by plotting y, the normalized current, as a function of compound concentration. Results are expressed as mean ± SEM, with n being the number of cells analyzed. EC_50_ values were determined by fitting the Hill equation to the dose-response curve.

### 2.3. Cell Culture for Apoptosis and Proliferation Assays

Cell lines Panc-1 (ACC 783) and Colo-357 (CVCL_0221) were obtained through the validated collection of the pHioniC consortium (MSC-ITN 813834). Both cell lines were grown in RPMI1640, supplemented with 1 mM sodium pyruvate and 1% Glutamax (all Gibco Thermo-Fisher, Waltham, MA USA) 10%FBS (BioChrom, Berlin, Germany), at 5% CO_2_ and 37 °C in a humidified atmosphere.

### 2.4. Apoptosis and Proliferation Assays

For cell proliferation assays, cells were seeded at a density of 10,000 cells/well in 96-well flat bottom culture plates (Corning, Kaiseslautern, Germany) and proliferation was measured through culture confluency using an IncuCyte device (Sartorius, Göttingen, Germany). Every hour, two phase contrast images per well were acquired, and a confluency mask was generated by training the analysis algorithm using representative images. Every image was then analyzed using the obtained parameters to determine culture confluency. Treatments were then added when cells reached a confluency of ~30% (Panc-1) or ~55% (Colo-357), and proliferation was determined for the following 60 h. Proliferation is reported as confluency increase with respect to the start of the treatment.

Spheroids were cultured in round bottom ultra-low attachment 96-well plates (Corning). The optimal seeding densities were empirically determined (8000 cells/well for Colo-357 or 10,000 cells/well for Panc-1 spheroids. The cells were suspended in 2% Matrigel (Corning), centrifuged at 1000× *g* for 10 min and the spheroids were allowed to form in the incubator. Once the spheroids were formed, the treatments indicated together with 8.9 µM cycloheximide (as apoptosis sensitizer) were added and apoptosis was determined by live imaging in the Incucyte system in real time for approximately 60 h using Caspase-3/7 green reagent (Sartorius) as a reporter of apoptosis, which crosses the cell membrane and is cleaved by the activated Caspase-3/7, resulting in the fluorescent staining of nuclear DNA. Apoptosis on Panc-1 tumor spheroids is presented as integrated green fluorescence in the whole spheroid.

### 2.5. Ligand-Based Pharmacophore Modeling

Nine K_V_1.3 inhibitors with inhibition greater than 75% at 10 µM were used as a training set for the generation of ten pharmacophore models in LigandScout 4.4 Expert (Inte:Ligand GmbH, Vienna, Austria) [22]. For each inhibitor, a maximum of 200 conformations were computed using iCon algorithm in LigandScout [23] with default “BEST” settings. Ten ligand-based pharmacophore models were created using the default settings. For each of the models, the creation of exclusion volumes coat around the alignment of the ligands was enabled. For the interaction analyses and image preparation, the highest ranked ligand-based pharmacophore model was used.

### 2.6. Turbidimetric Solubility Assay

For the phosphate-buffered saline, we dissolved 2.38 g of disodium hydrogen phosphate dodecahydrate, 0.19 g of potassium dihydrogen phosphate, and 8.0 g of sodium chloride in water and diluted it to 1000 mL with the same solvent. Then we adjusted the pH to 7.4. The stock DMSO solutions of the compounds were prepared at concentrations of 50 mM, 20 mM, 10 mM, and 1 mM. The final solutions (500 µM, 200 µM, 100 µM, and 10 µM) were prepared by adding 2 µL of the stock DMSO solution to 198 µL of phosphate-buffered saline. The absorbance (area scan) was measured at 620 nm for sample volume of 200 µL and the appearance of the solutions was checked by visual inspection.

## 3. Results

### 3.1. Design and Chemistry

**Figure 2:**
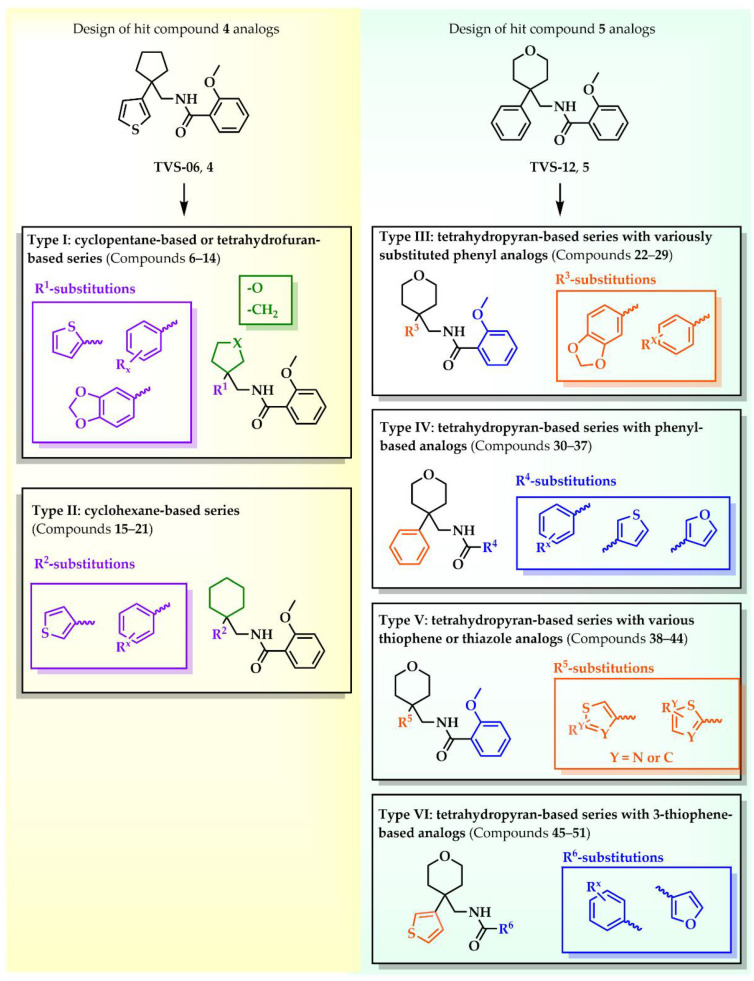
Design of new K_V_1.3 inhibitors of structural Types I–VI.

Six different types of cyclopentane-, cyclohexane- and tetrahydropyran-based K_V_1.3 inhibitors were designed and synthesized with the aim of increasing potency on K_V_1.3, and selectivity against other members of K_V_1 family of the hit compounds **4** (TVS-06) and **5** (TVS-12), and to investigate structure-activity relationships (SAR) important for K_V_1.3 inhibition. Hit compound **4** (TVS-06) (Figure 2), which contained a scaffold of 3-thiophene, cyclopentane, and 2-methoxybenzamide, was first modified in the aromatic (R^1^) or cyclopentane portion of the molecule to yield a new series of cyclopentane or tetrahydrofuran analogs that were screened for K_V_1.3 inhibition (Figure 2: Type I compounds). The cyclopentane was replaced by cyclohexane to give new cyclohexane analogs with aromatic R^2^ substituents (Figure 2: Type compounds II). In both Type I and II compounds, the 2-methoxybenzamide moiety was retained because it is important for binding to the K_V_1.3 channel.

Hit compound **5** with benzene, 2-methoxybenzamide, and tetrahydropyran structural moieties was modified at both aromatic moieties (Figure 2) to give four tetrahydropyran series (Figure 2: Types III–VI). The first strategy was to replace the benzene ring of hit compound **5** with several small substituents to obtain a new series of 2-methoxybenzamide-based compounds (Figure 2: Type III). Based on biological data, an unsubstituted phenyl ring was selected to be included in novel phenyl-based analogs in which modifications were introduced to the 2-methoxybenzamide moiety (Figure 2: Type IV). The unsubstituted phenyl from hit compound **5** was then replaced with various thiophene or thiazole rings (Figure 2: Type V compounds). Finally, new analogs were prepared with the most promising 3-thiophene moiety and with various modifications to the 2-methoxybenzamide moiety (Figure 2: Compounds of Type VI).

The synthesis of all new analogs is shown in Figures S2–S7. Synthetic procedures, analytical data, chemicals, reagents, and equipment used for organic synthesis and analysis are listed in the Supplementary Materials under the Chemistry section.

### 3.2. K_V_1.3 Potencies of Structural Type I and Type II Compounds

In the Type I series, compound **14** with 2-thiophene and the new tetrahydrofuran ring was the most potent with an IC_50_ value of 0.57 ± 0.36 µM (Table 1), whereas the compounds in the cyclohexane series (Type II compounds **15**–**21**) were inactive (Table 1).

### 3.3. K_V_1.3 Potencies of Type III–VI Compounds

New tetrahydropyran K_V_1.3 inhibitors were designed and synthesized to provide a focused library that was screened for K_V_1.3 inhibition (Table 2). The new compounds of Type III **22–29** (Table 2) were modified at the phenyl ring (R^3^, Figure 2) of hit compound **5**. The highest percentage of K_V_1.3 inhibition of the Type III compounds was achieved with the 3-substituted phenyl analogs (**28**–**29**), which were less effective compared with hit compound **5**.

The phenyl-based Type IV compounds (**30**–**37**) contained several modifications of the 2-methoxybenzamide moiety of the molecule. The activity of hit compound **5** was maintained with the new 2-methoxy-4-methylbenzamide (**36**) and 5-fluoro-2-methoxybenzamide (**37**) analogs (Table 2), which exhibited approximately two times higher IC_50_ values than **5**.

In the Type V series, the benzene ring of hit compound **5** was replaced by several thiophene or thiazole moieties (Figure 2, **38**–**44**) to obtain a suitable moiety that would give better selectivity and potency compared with the benzene ring of hit compound **5**. Two potent Type V inhibitors were obtained by using 2-thiophene (**43**) or 3-thiophene (**44**) moieties. The 2-thiophene analog **43** had a nanomolar IC_50_ value of 0.59 ± 0.15 µM and the most potent Type V compound was the 3-thiophene analog **44** with an IC_50_ value of 0.47 ± 0.02 µM.

Because the Type V compound **44** had a lower IC_50_ value for K_V_1.3 compared to hit compound **5**, the 3-thiophene moiety was determined to be the most promising bioisosteric replacement for the benzene ring and was incorporated into a new Type VI 3-thiophene-based analog **45**–**50** (Table 2) with modifications of the 2-methoxybenzamide part of the molecule (Figure 2). The most potent Type VI compound was the 2-methoxy-4-methylbenzamide analog **51**, which had an IC_50_ value of 2.92 ± 0.12 µM (Table 2).

### 3.4. Selectivity and IC_50_ Determinations of the Most Potent K_V_1.3 Inhibitors

**Figure 3:**
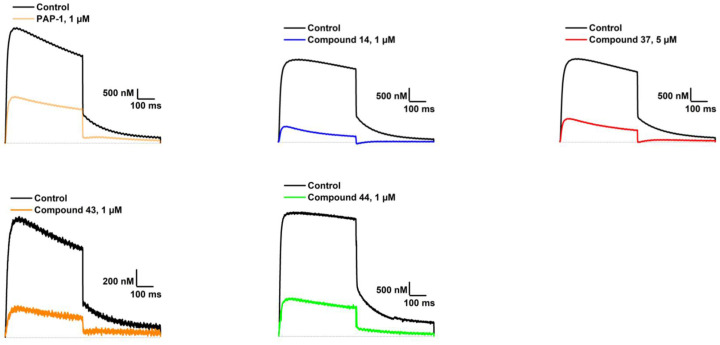
Raw traces of K_V_1.3 currents in control conditions in ND96 (black line) and after addition of PAP-1 (light orange line), **14** (blue line), **37** (red line), **43** (orange line), and **44** (green line). The dotted line indicates the zero current level. Measured by manual voltage clamp on *Xenopus laevis* oocytes.

The most potent compounds from Table 1 and Table 2 (**14**, **37**, **43** and **44**) and the reference compound PAP-1 (**1**) were tested for K_V_1.3 inhibition with an additional independent method of manual patch-clamp procedures on Ltk^−^ cells (Table 3). The aim was to demonstrate the inhibition of K_V_1.3 in a mammalian cell line and to have a direct comparison with the positive control PAP-1 (**1**), which was previously tested in L929 cells and human T-cells (IC_50_ of 2 nM**)** [11]. Interestingly, the reference compound PAP-1 (**1**) had an IC_50_ value of 780 nM (manual voltage clamp on oocytes) and 0.4 nM (manual patch clamp on Ltk^−^ cells), PAP-1 had a much lower potency on oocytes compared with the literature data (IC_50_ of 2 nM, L929 cells, manual whole-cell patch-clamp) and the IC_50_ value determined based on Ltk^−^ cells. The best compound of Types I- VI had comparable potency on oocytes (Figure 3, manual voltage-clamp) and Ltk^−^ cells (manual patch-clamp) of 470 nM and 950 nM, respectively.

**Figure 4:**
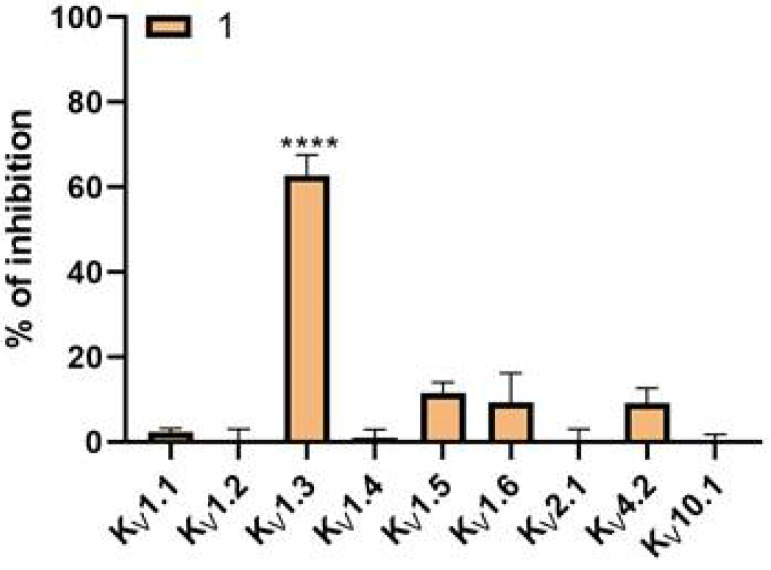
Selectivity determination of PAP-1 (**1**) screened at a concentration of 10 µM (HiClamp *Xenopus laevis* heterologous expression system) on a panel of ion channels. (**** *p* < 0.0001, Mean ± S.E.M, N = 3–12).

**Figure 5:**
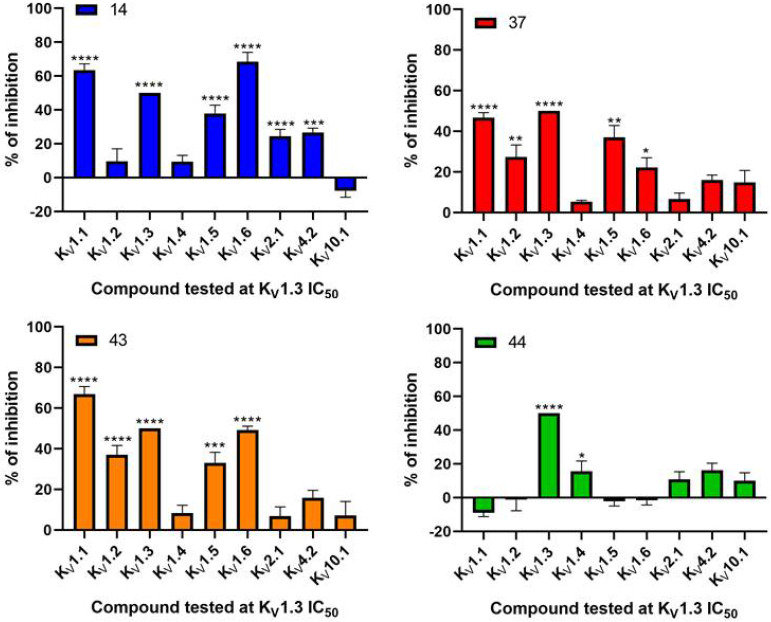
Selectivity determination of **14**, **37**, **43**, and **44** screened at a concentration of their IC_50_ values for K_V_1.3 on a panel of ion channels. **** *p* < 0.0001, *** *p* < 0.001, ** *p* < 0.01 * *p* < 0.05, Mean ± S.E.M, N = 3–12).

The aim of new potent K_V_1.3 inhibitors is sufficient selectivity against other voltage-gated potassium channels. Therefore, Kv1.3 inhibitors **14**, **37**, **43**, and **44** were screened against a panel of voltage-dependent channels using the *Xenopus laevis* heterologous expression system together with the reference compound PAP-1 (**1**). These channels were selected based on their importance in cardiac physiology and their structural similarity to K_V_1.3. PAP-1 (**1**) was tested at a concentration of 10 µM (Figure 4) and showed no significant effects on the channels K_V_1.1, K_V_1.2, K_V_1.4, K_V_1.5, K_V_1.6, K_V_2.1, K_V_4.2, and K_V_10.1 in *Xenopus laevis* oocytes. Compounds **14**, **37**, **43**, and **44** (Figure 5) were screened at the concentration of their IC_50_ values for K_V_1.3. IC_50_ values for other K_V_ channels were determined for the compounds that showed significant activity on other potassium channels compared with K_V_1.3 (Table 4).

At a concentration of its IC_50_ value (IC_50_ = 1.99 ± 0.61 µM, Table 4) for K_V_1.3, compound **44** with the 3-thiophene ring showed no significant effects on channels K_V_1.1, K_V_1.2, K_V_1.5, K_V_1.6, K_V_2.1 and K_V_10.1. However, it induced approximately a 16% inhibition of K_V_1.4 at this concentration, which is why the IC_50_ value was determined for K_V_1.4 (8.48 ± 2.21 µM, Table 4). Compound **44** showed the most appropriate selectivity profile compared to the other new K_V_1.3 inhibitors **14**, **37**, and **43**.

At a concentration of their IC_50_ values for K_V_1.3, **14** and **43**, based on 2-thiophene scaffolds, showed significant effects on K_V_1.1, Kv1.2, K_V_1.5, and K_V_1.6 (Figure 5), so IC_50_ values were determined for these channels. Compound **14** (IC_50_ = 1.03 ± 0.03 µM for K_V_1.3, Table 4) very potently inhibited K_V_1.1 (IC_50_ = 0.71 ± 0.26 µM, Table 4) and K_V_1.6 currents (IC_50_ = 0.43 ± 0.02 µM, Table 4). Similarly, **43** (IC_50_ = 1.20 ± 0.02 µM for K_V_1.3, Table 4) proved to be an even more potent inhibitor of K_V_1.1 (IC_50_ = 0.56 ± 0.12 µM, Table 4) and K_V_1.6 (IC_50_ = 0.83 ± 0.01 µM, Table 4) channels.

Compound **37**, containing a phenyl ring, at the concentration of the IC_50_ for K_V_1.3 (IC_50_ = 1.97 ± 0.14 µM, Table 4) significantly inhibited the currents of K_V_1.1, K_V_1.2, K_V_1.5, and K_V_1.6 (Table 4).

### 3.5. Effects on the Growth of Cell Lines in 2D Cell Culture

**Figure 6:**
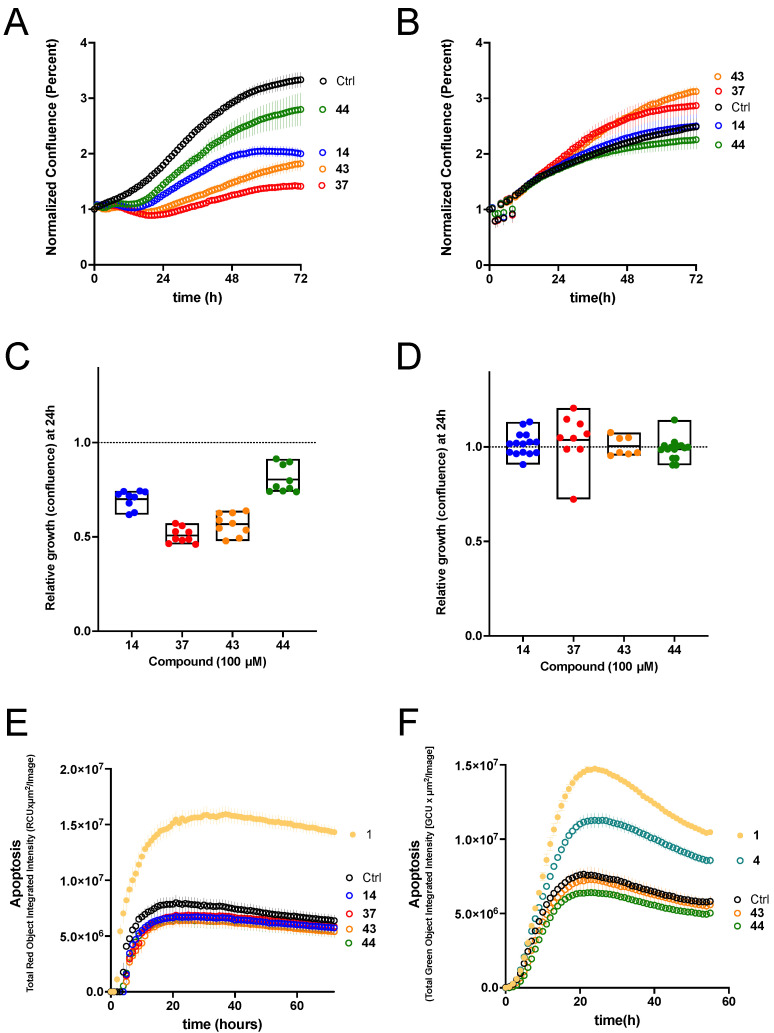
Effects on pancreatic cancer cell lines in 2D and 3D cell cultures. (**A**) Growth curves (culture confluence measured through phase contrast imaging) of Panc-1 cells in the presence of the indicated compounds (100 µM) or the vehicle (DMSO, black symbols). Mean ± S.E.M, N = 9. (**B**) The equivalent experiment on Colo-357 cells revealed that the growth inhibition was cell-type specific. Mean ± S.E.M, N = 15. The inhibition 24 h after the start of treatment is quantified in (**C**,**D**) for the data presented in (**A**,**B**) respectively. (**E**) Apoptosis measured as caspase 3/7 activity on Panc-1 tumor spheroids in the presence of the indicated compounds. (**F**) Apoptosis in Colo-357 tumor spheroids (*p* < 0.001, Mean ± S.E.M, N = 4).

K_V_1.3 is associated with the control of cell proliferation in various cancer cell types. Therefore, we investigated the effects of compounds **14**, **37**, **43** and **44** on the proliferation of two pancreatic cancer cell lines, Panc-1 and Colo-357, which have been reported to overexpress K_V_1.3 [19]. The compounds were tested at a concentration of 100 µM, which is the maximal concentration we can achieve while maintaining a low concentration of vehicle (1% DMSO, which was also added to the control). Proliferation was determined as confluence of the culture using live cell imaging over a 72 h period. For Panc-1 cells (Figure 6A,C), compound **44** caused moderate growth inhibition, whereas **14**, **43**, and especially **37** caused strong inhibition. However, the growth of Colo-357 cells (Figure 6B,D) was not significantly affected by any of the compounds.

### 3.6. Effects on the Growth of Cell Lines in 3D Cell Culture

To test the ability of the compounds to inhibit tumour progression in a more predictive system, we performed experiments on tumour spheroids of the pancreatic cancer cell lines used for 2D culture. In Panc-1 spheroids (Figure 6E), the compounds that effectively reduced proliferation in 2D culture (**14**, **37**, **43**, and **44**) did not induce detectable levels of apoptosis, whereas the reference compound PAP-1 did. In Colo-357 cell spheroids, significant induction of apoptosis was observed in the presence of the hit compound **4** (Figure 6F). None of the other compounds tested differed significantly from the control. The degree of apoptosis induction was dose-dependent, although we could not use concentrations higher than 100 µM and therefore cannot determine the IC_50_ for induction of apoptosis. The level of apoptosis achieved by 100 µM of hit compound **4** was similar to that achieved by 50 µM PAP-1 (**1**).

## 4. Discussion

**Figure 7:**
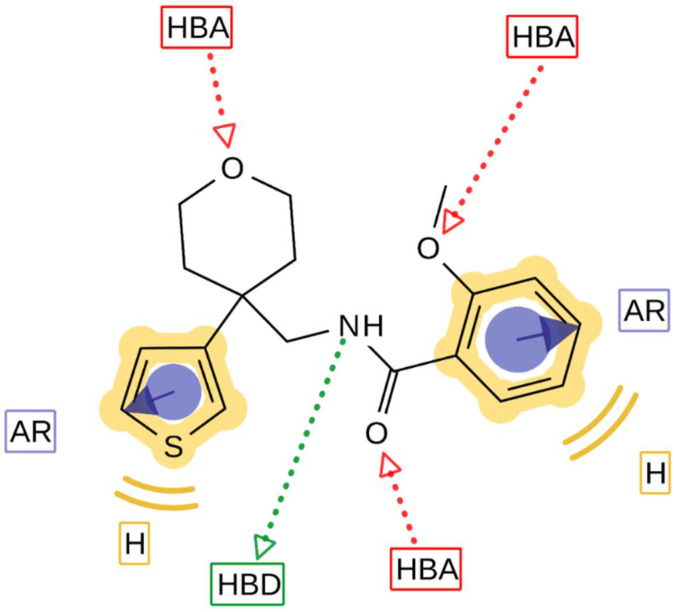
Pharmacophore model of new K_V_1.3 inhibitors. The model was prepared in the Ligandscout 4.4 Expert (Inte:Ligand GmbH) [22,29] using molecules with inhibition of K_V_1.3 higher than 75% at 10 µM concentration. Pharmacophore features represent: hydrophobic interactions, yellow spheres; aromatic interactions, blue discs; hydrogen bond donor, green arrow; hydrogen bond acceptors, red arrows. The pharmacophore model shows the most important features of K_V_1.3 inhibitors with the most potent compound **44** aligned to the pharmacophore model.

With the structure-activity relationship studies of novel structural classes K_V_1.3 inhibitors, we identified structural parts that are important for K_V_1.3 inhibition. With the structural optimization of hit compounds **4** and **5** we developed new K_V_1.3 inhibitors (e.g., **14**, **43**, and **44)** with improved potency compared to hit compounds. To explain the structural requirements for K_V_1.3 inhibition, we created a pharmacophore model of the new K_V_1.3 inhibitors in Ligandscout 4.4 Expert (Inte:Ligand GmbH) using inhibitors that achieved K_V_1.3 inhibition greater than 75% at a concentration of 10 µM. The ligand-based pharmacophore model (Figure 7) is presented with the structure of the most potent K_V_1.3 inhibitor **44**. Structural elements in the most potent analogs that were required for high-affinity interaction with K_V_1.3 were the amide bond (hydrogen bond acceptor, HBA, hydrogen bond donor, HBD), two aromatic features, oxygen in the 2-methoxy group as HBA, and oxygen in the rings of tetrahydrofuran (Type I) or tetrahydropyran (Type III-VI) rings as HBA. 2-Methoxybenzamide was present in the first aromatic feature (Types I, III and FThe second aromatic feature of most potent inhibitors included 3-substituted (Type III, **28** and **29**) or unsubstituted benzene (Type IV, **36**, **37**), unsubstituted 2-thiophene (Type I, **14** and Type V, **43**) or unsubstituted 3-thiophene (Type V, **44** and Type VI, **51**).

Several known small molecule K_V_1.3 inhibitors lack selectivity for K_V_1.3 over the closely related K_V_1.x family channels, which have high subtype homology. The lack of selectivity for K_V_1.5 raises many concerns regarding potential acute cardiac toxicity. Obtaining a selective small molecule inhibitor remains a major challenge, and often the lack of selectivity prevents subsequent optimization. Based on literature data, PAP-1 (Figure 1, **1**) is selective toward K_V_1 channels, whereas it has the lowest selectivity toward the K_V_1.5 channel (i.e., 23-fold over K_V_1.5). We also included into our testing the reference compound PAP-1 to have a direct comparison of potency and selectivity with newly designed compounds. We determined IC_50_ values for PAP-1 using three independent methods: manual voltage clamp on oocytes (IC_50_ = 0.78 ± 0.01 µM), HiClamp system on oocytes (IC_50_ = 2.67 ± 0.30 µM), and manual patch clamp on Ltk^−^ cells (IC_50_ = 0.0004 ± 0.00002 µM). Surprisingly, the IC_50_ values determined for PAP-1 on oocytes were approximately 390- to 1335-fold higher than the literature IC_50_ value of 2 nM (L929 cells, manual whole-cell patch-clamp) [24]. Regarding selectivity, the PAP-1 tested at a concentration of 10 µM showed no significant effects on channels K_V_1.1, K_V_1.2, K_V_1.4, K_V_1.5, K_V_1.6, K_V_2.1, K_V_4.2, and K_V_10.1 using the HiClamp system on oocytes.

Comparing the IC_50_ values in Table 3, we can see some differences between the different test systems can be seen. The IC_50_ values determined based on oocytes using the manual and HiClamp methods are in the same order of magnitude (middle-nanomolar to low-micromolar range), but the IC_50_ values determined based on mammalian cells are in the low-nanomolar range. These differences can be attributed to several factors:

First, both mammalian and amphibian cells were used, which differ in size, composition, ion channel expression, and permeability to compounds [25].

Second, when comparing the two different methods used on oocytes (manual voltage clamp and HiClamp), it seems that IC_50_ values are generally slightly higher for the HiClamp method than for the two manual techniques. This may be due to differences in perfusion between the three systems. In the manual setup, oocytes are manually impaled in the recording chamber with two electrodes, and the test solution is either externally applied to the bath filled with ND96 (in the case of the manual voltage-clamp experiments) or applied by continuous extracellular perfusion using a pressurized fast-perfusion system (in the case of the manual patch-clamp experiments), while K_V_1.3 currents are measured. The HiClamp, on the other hand, is a semi-automatic system, in which the oocyte is picked up from a 96-well plate, deposited in a basket and automatically impaled by two electrodes in the washing chamber. Next, the basket will submerge the oocyte in the test solution in another 96-well plate while K_V_1.3 currents are measured. In this plate, magnets are continuously stirring the test solutions to assure homogenous perfusion. The higher IC_50_s observed at the HiClamp could be due to adhesion of the compound to the walls of the 96-well plate or because of the continuous stirring by the magnet, which is not present in the two other experimental setups.

Third, the voltage protocols used are slightly different. In the voltage-clamp experiments, depolarizing pulses of 1 s duration were used at intervals of 1 s, whereas in the patch-clamp experiments, depolarizing pulses of 200 ms duration were applied at intervals of 15 s. The voltage protocol used in the patch-clamp experiments is slightly different. The voltage protocol can affect the IC_50_ value depending on the state dependence and kinetics of the compound [26,27]. If the compound prefers to bind to a certain state of the channel and the voltage-protocol favors this state, then the observed IC_50_ will be lower compared to when a voltage-protocol that does not favor this state is used. Similarly, lower affinities will be observed if compounds with slow binding kinetics are measured in a voltage-protocol with a short occupancy period of the open or inactivated state, and vice-versa [28].

Because the solubility of the new drug candidates in aqueous media is relatively low in many cases, we performed a turbidimetric solubility test (Table S1) in phosphate-buffered saline (with a final 1% DMSO concentration) for compounds **37**, **43**, and **44**. Compounds **43** and **44** were soluble at a concentration of 500 µM and compound **37** was soluble at a concentration of 200 µM. For the determination of IC_50_, the concentrations indicated on the x-axis in logarithmic scale were 0.01 µM, 0.1 µM, 1 µM, 10 µM, and 100 µM. At these concentrations, compounds **37**, **43** and **44** were soluble, therefore the IC_50_ values determined are not affected by the solubility of these compounds.

Our aim was to design novel potent and selective K_V_1.3 inhibitors based on previously determined hit compounds **4** and **5**. Based on selectivity data for hit compounds **4** and **5**, we hypothesized that the 3-thiophene scaffold and/or the 2-methoxybenzamide moiety together might be responsible for the high selectivity of hit compound **4**. To test this hypothesis, we designed six types of novel analogs (Types I-VI) that incorporated 3-thiophene (Types V and VI), 2-thiophene (Types I and V), or benzene (Types III or IV) as scaffolds in the amine portion of the molecules. Compounds **43** (Type I) and **44** (Type V) had very similar affinity for K_V_1.3 (IC_50_ = 0.59 ± 0.15 µM and 0.47 ± 0.02 µM, respectively), but interestingly, the 2- or 3-thiophene position leads to a completely different selectivity profile. Among the new K_V_1.3 inhibitors, **44** (Type V) with 3-thiophene scaffold and 2-methoxybenzamide moiety showed the best selectivity profile showing no significant inhibition of K_V_1.x channels, except it inhibited K_V_1.4 with IC_50_ value of 8.48 ± 2.21 µM. In contrast, inhibitors **14**, and **43** with 2-thiophene scaffold and 2-methoxy benzamide moiety lacked selectivity toward K_V_1.1, K_V_1.2, K_V_1.5, and K_V_1.6. It appears that both the 3-thiophene scaffold and the 2-methoxybenzamide moiety are responsible for the high selectivity of hit compound **4** and the novel benzamide analog **44**, but the new structural elements of tetrahydropyran (**37**, **43** and **44**) and tetrahydrofuran (**14**) allow higher potency for K_V_1.3.

K_V_1.3 activity is required for cell proliferation, migration, and invasion, which are very important events in cancer progression [9]. To date, there are several lines of evidence that cell-permeable inhibitors of K_V_1.3, PAP-1 (**1**) and clofazimine (**3**), specifically target K_V_1.3 to mediate apoptosis. However, PAP-1-based mitochondria-targeted derivatives (PAPTP and PCARBTP) bound to the lipophilic triphenylphosphonium cation (TPP) were found to be very effective in several cancer models, including pancreatic ductal adenocarcinoma, melanoma, and glioblastoma [30,31]. PAPTP and PCARBTP were able to decrease the cell survival with EC_50_ of approximately 3 and 6 µM in Panc-1 cell line. The decrease in MTS levels when 10 µM PAPTP or PCARBTP was used was due to the apoptosis of Panc-1, while clofazimine only induced cell death of less than 30% even at a concentration of 20 µM. On the other hand, cell death of Colo-357 was achieved only after treatment with high concentrations of PAPTP, PCARBTP, and clofazimine (EC_50_s of 3.7, 2, and 1.5 µM, respectively) [31].

We tested the ability of our new potent K_V_1.3 inhibitors to inhibit the proliferation of two K_V_1.3-expressing PDAC cell lines, Colo-357 (metastatic) and Panc-1 (from the pancreatic duct). Growth of Panc-1 cells was inhibited by K_V_1.3 inhibitors **14**, **37**, **43**, and **44**, with **37** in particular causing strong inhibition. Colo-357 growth was unaffected in the presence of these compounds. The fact that Colo-357 cells were unaffected, together with the modest effects observed in Panc-1, indicate that plasma membrane K_V_1.3 is dispensable for the proliferation of these cell types. The discrepancy between cell lines can be attributed to the selectivity profiles of the compounds, since K_V_1.1 and K_V_1.5 have also been implicated in cell proliferation (see for example REF) [32] and are targets for compounds **14**, **37** and **43**, while the most selective of the new compounds (**44**) had the smallest effect. In Colo-357 tumor spheroids, starting hit compound **4** induced a significant amount of apoptosis, and the degree of apoptosis achieved by 100 µM of **4** was similar to that achieved by 50 µM PAP-1 (**1**). All compounds were ineffective as apoptosis inducers in Panc-1 tumor spheroids, whereas PAP-1 was able to induce significant apoptosis. Based on our efficacy data in PDAC cell lines and Colo-357 tumor spheroids, we hypothesize that inhibition of mitochondrial K_V_1.3 ion channels is required for significant anticancer activity, as previously demonstrated with PAP-1-based mitochondrial K_V_1.3 inhibitors (PAPTP and PCARBTP). Whether the ability of compound **4**, which is specific against K_V_1.3 [21], is due to its ability to reach mitochondrial K_V_1.3 channels remains to be investigated. Our results, however, highlight the potential to develop new mitochondrial Kv1.3 inhibitors by adding a TPP moiety to our potent and selective thiophene-based K_V_1.3 inhibitors.

## 5. Conclusions

To discover a novel structural class of K_V_1.3 inhibitors overexpressed in many different tumour types, we used a structural optimization approach and successfully prepared novel potent and selective thiophene-based K_V_1.3 inhibitors. We identified the potent and appropriately selective nanomolar K_V_1.3 inhibitor **44**, which contains 3-thiophene and tetrahydropyran scaffolds. We demonstrated the inhibition of the Panc-1 cancer cell line proliferation by the new K_V_1.3 inhibitors. Hit compound **4** induced significant apoptosis in Colo-357 tumour spheroids, and the extent of apoptosis achieved by 100 µM of **4** was comparable to that achieved by 50 µM PAP-1 (**1**). Based on the efficacy data in PDAC cell lines and Colo-357 tumour spheroids, we can assume that newly developed K_V_1.3 inhibitors do not reach the mitochondrial K_V_1.3 channels required for the induction of apoptosis. There is an opportunity to further develop the new structural class of potent and selective K_V_1.3 inhibitors into mitochondrial K_V_1.3 inhibitors by adding mitochondrial targeting moieties.

## Figures and Tables

**Table 1:** K_V_1.3 inhibitory activities of new analogs **6**–**21** from Type I and II series, manually voltage-clamped to determine the percentage of inhibition at 10 μM and the IC_50_s values determined with manual voltage-clamp on *Xenopus laevis* oocytes.

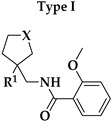				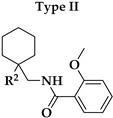		
Compound ID	R^1^	X	% of K_V_1.3 Inhibition or IC_50_ [µM]	Compound ID	R^2^	% of K_V_1.3 Inhibition or IC_50_ [µM]
**6**	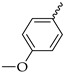	CH_2_	<10%	**15**	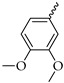	<10%
**7**	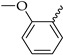	CH_2_	<10%	**16**	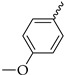	<10%
**8**	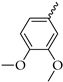	CH_2_	<10%	**17**	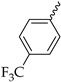	<10%
**9**	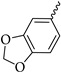	CH_2_	<10%	**18**	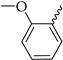	<10%
**10**	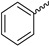	CH_2_	<10%	**19**	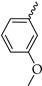	12 ± 2%
**11**	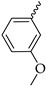	CH_2_	18 ± 3%	**20**	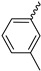	27 ± 2%
**12**	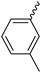	CH_2_	16 ± 4%	**21**	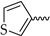	30 ± 8%
**13**	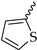	CH_2_	57 ± 9%			
**14**	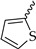	O	92 ± 4% 0.57 ± 0.36 µM			

**Table 2:** Structures and K_V_1.3 inhibitory potencies of the designed and synthesized Type IV–VI analogs **22**–**51**, manually voltage-clamped to determine the percentage of inhibition at 10 μM and the IC_50_s values determined with manual voltage-clamp or HiClamp * on oocytes.

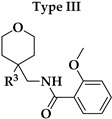			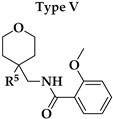		
Compound ID	R^3^	% of K_V_1.3 Inhibition or IC_50_ [µM]	Compound ID	R^5^	% of K_V_1.3 Inhibition or IC_50_ [µM]
**22**	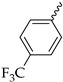	<10%	**38**	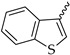	<10%
**23**	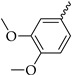	<10%	**39**	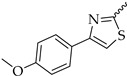	<10%
**24**	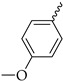	59 ± 5%	**40**	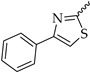	<10%
**25**	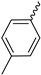	52 ± 8%	**41**	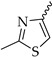	41 ± 10%
**26**	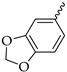	60 ± 9%	**42**	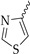	54 ± 10%
**27**	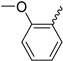	78 ± 4%	**43**	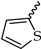	92 ± 1% 0.59 ± 0.15 µM
**28**	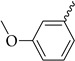	88 ± 4% 5.75 ± 1.11 µM	**44**	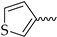	96 ± 1% 0.47 ± 0.02 µM
**29**	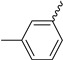	87 ± 4% 4.30 ± 0.33 µM			
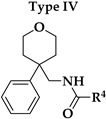			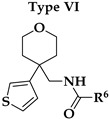		
**Compound ID**	**R^4^**	**% of K_V_1.3 Inhibition or IC_50_ [µM]**	**Compound ID**	**R^6^**	**% of K_V_1.3 Inhibition or IC_50_ [µM]**
**30**	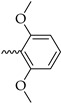	<10%	**45**	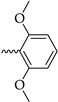	<10%
**31**	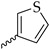	11 ± 4%	**46**	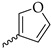	21 ± 6%
**32**	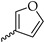	27 ± 6%	**47**	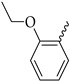	35 ± 8%
**33**	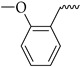	38 ± 5%	**48**	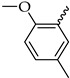	42 ± 6%
**34**	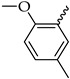	47 ± 1%	**49**	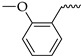	43 ± 1%
**35**	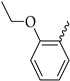	74 ± 9%	**50**	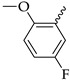	47 ± 9%
**36**	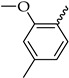	89 ± 4% 1.65 ± 0.38 µM *	**51**	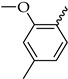	82 ± 1% 2.92 ± 0.12 µM *
**37**	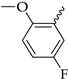	94 ± 1% 1.97 ± 0.14 µM *			

**Table 3:** Comparison of K_V_1.3 IC_50_ values for compounds **14**, **37**, **43**, **44**, and PAP-1 (**1**) obtained with HiClamp and manual voltage-clamp on *Xenopus laevis* oocytes (Table 1 and Table 2) and with manual patch-clamp on the Ltk^−^ cell-line.

Compound ID	IC_50_ (Manual Voltage-Clamp Oocytes) [µM]	IC_50_ (HiClamp Oocytes) [µM]	IC_50_ (Manual Patch-Clamp Ltk^−^) [µM]
**PAP-1 (1)**	0.78 ± 0.01	2.67 ± 0.30	0.0004 ± 0.00002
**14**	0.57 ± 0.36	1.03 ± 0.03	1.33 ± 0.20
**37**	3.96 ± 0.47	1.97 ± 0.14	1.35 ± 0.04
**43**	0.59 ± 0.15	1.20 ± 0.02	1.02 ± 0.07
**44**	0.47 ± 0.02	1.99 ± 0.61	0.95 ± 0.24

**Table 4:** IC_50_ values [µM] for analogs **PAP-1**, **14**, **37**, **43**, and **44** on relevant voltage-gated ion channels. IC_50_ values were determined with HiClamp *Xenopus laevis* heterologous expression system for analogs which showed significant inhibition on the tested ion channels in Figure 5. The NA means that the analogue did not show significant inhibition on the tested ion channel and hence no IC_50_ was determined.

	PAP-1 (1)	14	37	43	44
K_V_1.1	NA	0.71 ± 0.26	1.76 ± 0.40	0.56 ± 0.12	NA
K_V_1.2	NA	4.00 ± 0.20	2.34 ± 0.26	2.76 ± 0.56	NA
K_V_1.3	2.67 ± 0.30	1.03 ± 0.03	1.97 ± 0.14	1.20 ± 0.02	1.99 ± 0.61
K_V_1.4	NA	NA	NA	NA	8.48 ± 2.21
K_V_1.5	NA	2.07 ± 0.13	8.64 ± 0.03	4.33 ± 0.01	NA
K_V_1.6	NA	0.43 ± 0.02	4.33 ± 1.21	0.83 ± 0.01	NA
K_V_2.1	NA	6.45 ± 0.42	NA	NA	NA
K_V_4.2	NA	2.41 ± 0.88	NA	NA	NA
K_V_10.1	NA	NA	NA	NA	NA

## Data Availability

The data presented in this study are available on request from the corresponding author.

## Supplementary Materials

The following are available online at https://www.mdpi.com/2072-6694/14/11/2595/s1, Figure S1: Addition to Table 4; Figure S2: Synthesis of cyclopentane and tetrahydrofuran derivatives; Figure S3: Synthesis of cyclohexane derivatives; Figure S4: Synthesis of benzene-based tetrahydropyran derivatives; Figure S5: Synthesis of benzene-based tetrahydropyran derivatives; Figure S6: Synthesis of heterocyclic tetrahydropyran derivatives; Figure S7: Synthesis of 3-thiophene-based tetrahydropyran derivatives; Table S1: Chemistry: analytical procedures description and NMR spectrums.
