# Correction: Gubič et al. Design of New Potent and Selective Thiophene-Based K_V_1.3 Inhibitors and Their Potential for Anticancer Activity. *Cancers* 2022, *14*, 2595

**DOI:** 10.3390/cancers15112925

**Published:** 2023-05-26

**Authors:** Špela Gubič, Louise Antonia Hendrickx, Xiaoyi Shi, Žan Toplak, Štefan Možina, Kenny M. Van Theemsche, Ernesto Lopes Pinheiro-Junior, Steve Peigneur, Alain J. Labro, Luis A. Pardo, Jan Tytgat, Tihomir Tomašič, Lucija Peterlin Mašič

**Affiliations:** 1 Faculty of Pharmacy, University of Ljubljana, Aškerčeva 7, 1000 Ljubljana, Slovenia; 2 Campus Gasthuisberg, University of Leuven, Toxicology and Pharmacology, Onderwijs en Navorsing 2, Herestraat 49, 3000 Leuven, Belgium; 3 AG Oncophysiology, Max-Planck Institute for Multidisciplinary Sciences, Hermann-Rein-Str. 3, 37075 Göttingen, Germany; 4 Laboratory for Molecular, Cellular and Network Excitability, Department of Biomedical Sciences, University of Antwerp, 2610 Wilrijk, Belgium; 5 Department of Basic and Applied Medical Sciences, Ghent University, Corneel Heymanslaan 10 (Entrance 36), 9000 Ghent, Belgium

## 1. Error in Table

In the original publication [1], there was a mistake in **Table 3** as published. The **IC_50_ value determined based on Ltk cells for PAP-1 is 1000 times lower**. The corrected **Table 3** appears below.

## 2. Text Correction

There was an error in the original publication. The **IC_50_ value determined based on Ltk^-^ cells for PAP-1 is 1000 times lower**.

A correction has been made to the following sections: **3. Results**, *3.4. Selectivity and IC_50_ Determinations of the Most Potent K_V_1.3 Inhibitors*, first paragraph:

The most potent compounds from Tables 1 and 2 (**14**, **37**, **43** and **44**) and the reference compound PAP-1 (**1**) were tested for K_V_1.3 inhibition with an additional independent method of manual patch-clamp procedures on Ltk^−^ cells (Table 3). The aim was to demonstrate the inhibition of K_V_1.3 in a mammalian cell line and to have a direct comparison with the positive control PAP-1 (**1**), which was previously tested in L929 cells and human T-cells (IC_50_ of 2 nM**)** [11]. Interestingly, the reference compound PAP-1 (**1**) had an IC_50_ value of 780 nM (manual voltage clamp on oocytes) and 0.4 nM (manual patch clamp on Ltk^−^ cells), **PAP-1 had a much lower potency on oocytes compared with the literature data** (IC_50_ of 2 nM, L929 cells, manual whole-cell patch-clamp) **and the IC_50_ value determined based on Ltk^−^ cells**. The best compound of Types I- VI had comparable potency on oocytes (Figure 3, manual voltage-clamp) and Ltk^−^ cells (manual patch-clamp) of 470 nM and 950 nM, respectively.

A correction has been made to the **4. Discussion**, second paragraph:

Several known small molecule K_V_1.3 inhibitors lack selectivity for K_V_1.3 over the closely related K_V_1.x family channels, which have high subtype homology. The lack of selectivity for K_V_1.5 raises many concerns regarding potential acute cardiac toxicity. Obtaining a selective small molecule inhibitor remains a major challenge, and often the lack of selectivity prevents subsequent optimization. Based on literature data, PAP-1 (Figure 1, **1**) is selective toward K_V_1 channels, whereas it has the lowest selectivity toward the K_V_1.5 channel (i.e., 23-fold over K_V_1.5). We also included into our testing the reference compound PAP-1 to have a direct comparison of potency and selectivity with newly designed compounds. We determined IC_50_ values for PAP-1 using three independent methods: manual voltage clamp on oocytes (IC_50_ = 0.78 ± 0.01 µM), HiClamp system on oocytes (IC_50_ = 2.67 ± 0.30 µM), and manual patch clamp on Ltk^−^ cells (**IC_50_ = 0.0004 ± 0.00002 µM**). Surprisingly, the IC_50_ values determined for PAP-1 **on oocytes** were approximately **390-** to 1335-fold higher than the literature IC_50_ value of 2 nM (L929 cells, manual whole-cell patch-clamp) [24]. Regarding selectivity, the PAP-1 tested at a concentration of 10 µM showed no significant effects on channels K_V_1.1, K_V_1.2, K_V_1.4, K_V_1.5, K_V_1.6, K_V_2.1, K_V_4.2, and K_V_10.1 using the HiClamp system on oocytes.

Comparing the IC_50_ values in Table 3, we can see some differences between the different test systems **can be seen**. **The IC_50_ values determined based on oocytes using the manual and HiClamp methods** are in the same order of magnitude (middle-nanomolar to low-micromolar range), **but the IC_50_ values determined based on mammalian cells are in the low-nanomolar range. These differences** can be attributed to several factors:

First, both mammalian and amphibian cells were used, which differ in size, composition, ion channel expression, and permeability to compounds [25].

Second, when comparing the **two** different methods **used on oocytes** (manual voltage clamp **and** HiClamp), it seems that IC_50_ values are generally slightly higher for the HiClamp method than for the two manual techniques. This may be due to differences in perfusion between the three systems. In the manual setup, oocytes are manually impaled in the recording chamber with two electrodes, and the test solution is either externally applied to the bath filled with ND96 (in the case of the manual voltage-clamp experiments) or applied by continuous extracellular perfusion using a pressurized fast-perfusion system (in the case of the manual patch-clamp experiments), while K_V_1.3 currents are measured. The HiClamp, on the other hand, is a semi-automatic system, in which the oocyte is picked up from a 96-well plate, deposited in a basket and automatically impaled by two electrodes in the washing chamber. Next, the basket will submerge the oocyte in the test solution in another 96-well plate while K_V_1.3 currents are measured. In this plate, magnets are continuously stirring the test solutions to assure homogenous perfusion. The higher IC_50_s observed at the HiClamp could be due to adhesion of the compound to the walls of the 96-well plate or because of the continuous stirring by the magnet, which is not present in the two other experimental setups.

The authors state that the scientific conclusions are unaffected. This correction was approved by the Academic Editor. The original publication has also been updated.

## Figures and Tables

**Table 3:** Comparison of K_V_1.3 IC_50_ values for compounds **14**, **37**, **43**, **44**, and PAP-1 (**1**) obtained with HiClamp and manual voltage-clamp on *Xenopus laevis* oocytes (Tables 1 and 2) and with manual patch-clamp on the Ltk^−^ cell-line.

Compound ID	IC_50_ (Manual Voltage-Clamp Oocytes) [µM]	IC_50_ (HiClamp Oocytes) [µM]	IC_50_ (Manual Patch-Clamp Ltk^−^) [µM]
**PAP-1 (1)**	0.78 ± 0.01	2.67 ± 0.30	**0.0004 ± 0.00002**
**14**	0.57 ± 0.36	1.03 ± 0.03	1.33 ± 0.20
**37**	3.96 ± 0.47	1.97 ± 0.14	1.35 ± 0.04
**43**	0.59 ± 0.15	1.20 ± 0.02	1.02 ± 0.07
**44**	0.47 ± 0.02	1.99 ± 0.61	0.95 ± 0.24
