# Supplementary material for: Design of New Potent and Selective Thiophene-Based KV1.3 Inhibitors and Their Potential for Anticancer Activity
Source: Cancers (Basel). 2022 May 24;14(11):2595. doi: 10.3390/cancers14112595 (PMC9179341; doi:10.3390/cancers14112595)

cancers

MDPI

Supplementary Materials: Design of New Potent and Selective Thiophene-Based Kv1.3 Inhibitors and Their Potential for Anti-cancer Activity

Špela Gubič, Louise Antonia Hendrickx, Xiaoyi Shi, Žan Toplak, Štefan Možina, Kenny M. Van Theemsche, Ernesto Lopes Pinheiro-Junior, Steve Peigneur, Alain J. Labro, Luis A. Pardo, Jan Tytgat, Tihomir Tomašič and Lucija Peterlin Mašič

Supplementary concentration-response curves

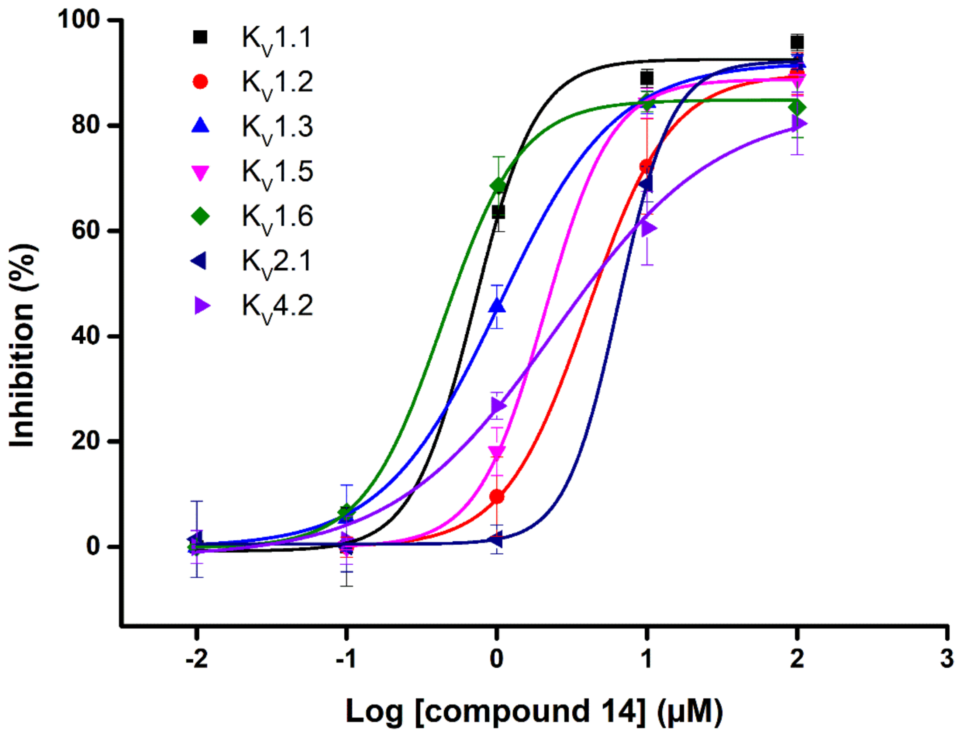

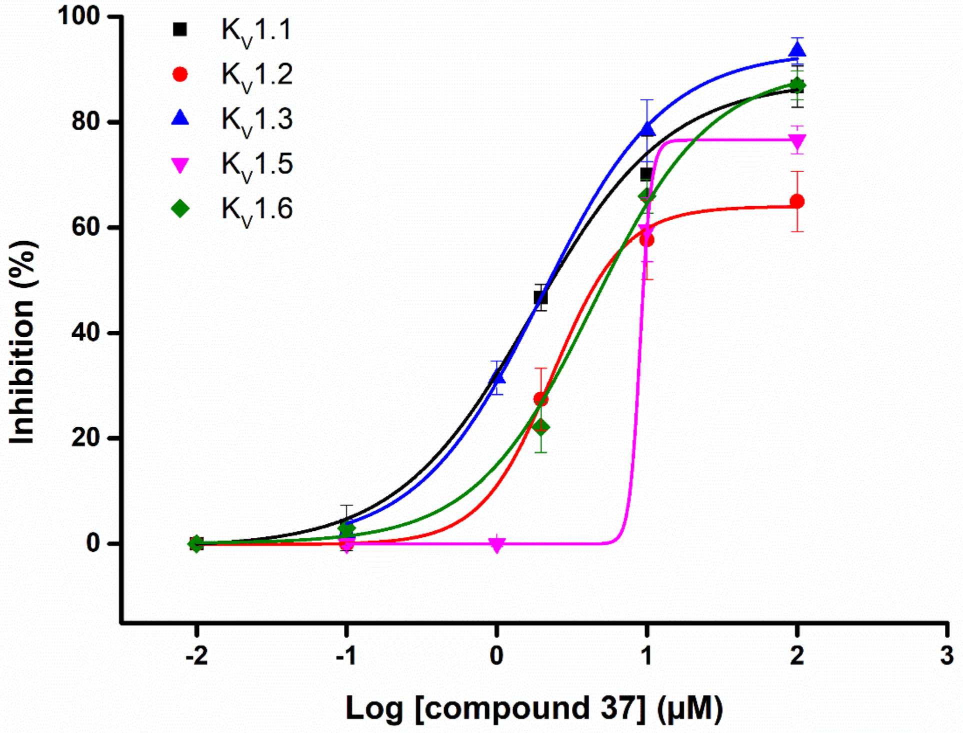

Inhibition (%)
Log [compound 37] (μM)
K_V1.1
K_V1.2
K_V1.3
K_V1.5
K_V1.6

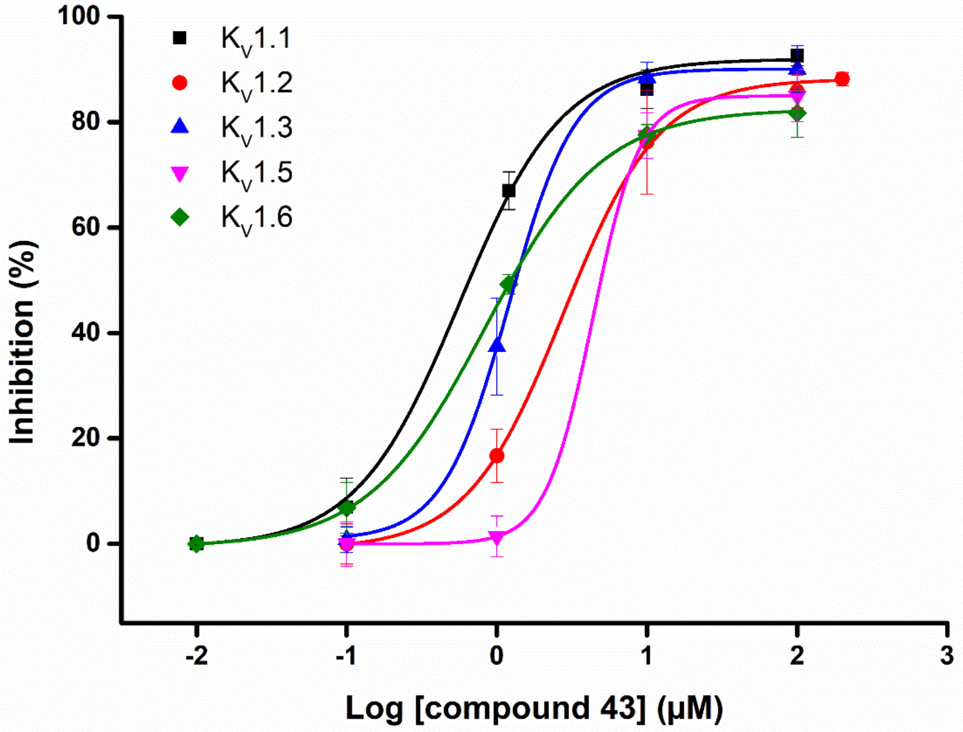

Inhibition (%)
Log [compound 43] (μM)
K_V1.1
K_V1.2
K_V1.3
K_V1.5
K_V1.6

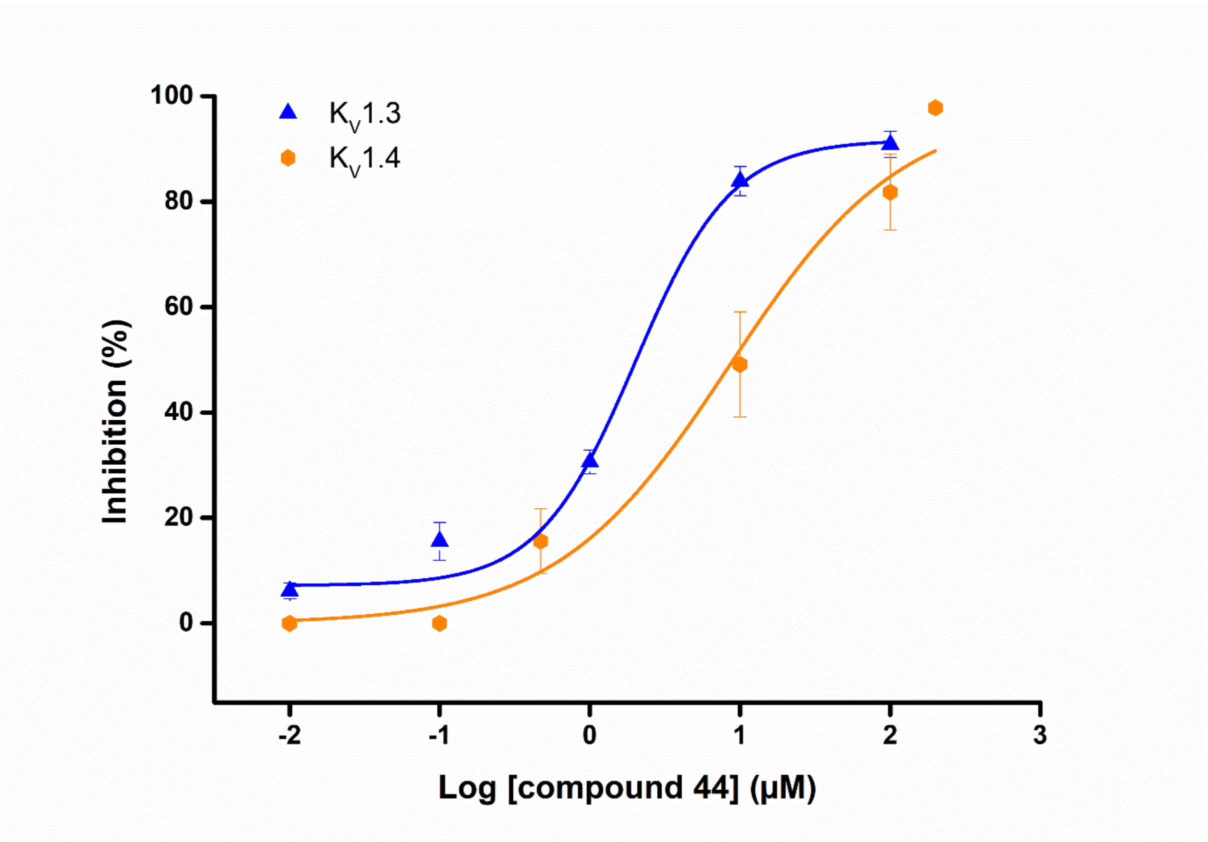

Figure S1. Addition to Table 4: concentration-response curves for compounds **14**, **37**, **43**, and **44** on the relevant voltage-gated ion channels. The suboptimal fit for compound 37 on Kv1.5 is most likely due to the scarcity of data points.

Supplementary tables

Table S1. Turbidimetric Solubility Assay. Compounds **4**, **37**, **43**, and **44** tested at 1 µM, 10 µM, 100 µM, and 500 µM with their absorbance (area scan at 620 nm).

Concentration [µM] in PBS with 1 % final DMSO concentration	Compound ID and their absorbance at 620 nm			
	**4**	**44**	**37**	**43**
500	0,189	0,014	0,077	0,011
200	0,127	0,003	0,005	0,003
100	0,035	0,003	0,003	0,002
10	0	0	0	0,01

Chemistry

Reagents and solvents were obtained from Acros Organics (Geel, Belgium), Sigma-Aldrich (St. Louis, MO, USA), Fluorochem (Derbyshire, United Kingdom), TCI EUROPE N.V. (Zwijndrecht, Belgium) and Apollo Scientific (Stockport, UK). Merck 60 F254 plates (0.25 mm) were used for the TLC analysis visualized with UV light and spray reagents. Flash column chromatography was performed on silica gel 60 (particle size 240–400 mesh). Melting points were determined on a hot-stage microscope from Reichert. ^1H and ^{13}C NMR spectra were recorded at 400 and 100 MHz on a Bruker AVANCE III 400 spectrometer (Bruker Corporation, MA, USA) with the DMSO-d_6 or $CDCl_3$ solutions. For HPLC analyses an Thermo Scientific Dionex Ultimate 3000 (Thermo Fisher Scientific, Waltham, MA, USA) and Acquity UPLC C18 column (1,7 µm, 2,1 mm × 50 mm). Mobile phase consisted of acetonitrile (as solvent A) and 0.1% TFA acid and ultrapure water (as solvent B). The gradient for solvent A was 10–90% in 8 min and 90% to 11 min with flow rate 0.4 mL/min and

injection volume: 2 µL. Mass spectra were obtained using ADVION expression CMSL mass spectrometer (Advion Inc., Ithaca, USA).

Synthetic procedures and analytical data

Cycloalkane rings were prepared using sodium hydride to deprotonate aryl acetonitriles under inert atmosphere while ice cooling in anhydrous DMF followed by double alkylation with addition of dihalogenated alkanes. Synthesis of cyclopentane (Figure S2) and cyclohexane (Figure S3) substituted aryl acetonitriles was carried out by double alkylation of 1,4-dibromobutane or 1,5-dibropentane. Tetrahydropyran rings (Figure S4-S7) were prepared by double alkylation of 1-chloro-2-(2-chloroethoxy)ethane to corresponding aryl acetonitriles and tetrahyrofuran ring by double alkylation of 1-chloro-2-(chloromethoxy)ethane while ice cooling at -35 °C (Figure S2). Thiazoles (Figure S6) were synthesized by condensation of α-haloketones and cyanoethanethioamide. The Hantzsch thiazole synthesis was carried out in anhydrous DMF using basic conditions while ice cooling.

$LiAlH_4$ was used for reduction of aryl acetonitriles to primary amines under inert atmosphere in anhydrous THF overnight. Hydride nucleophile attacked the electrophilic carbon in the nitrile and after 16 hours water was added to protonate the formed dianion.

The acids were transformed to acyl chlorides using oxalyl chloride and small amount of DMF as catalyst overnight in dichloromethane. The primary amines were converted to the corresponding benzamides (Figure S4 and Figure S7) by treatment with acyl chlorides and triethylamine in dichloromethane overnight.

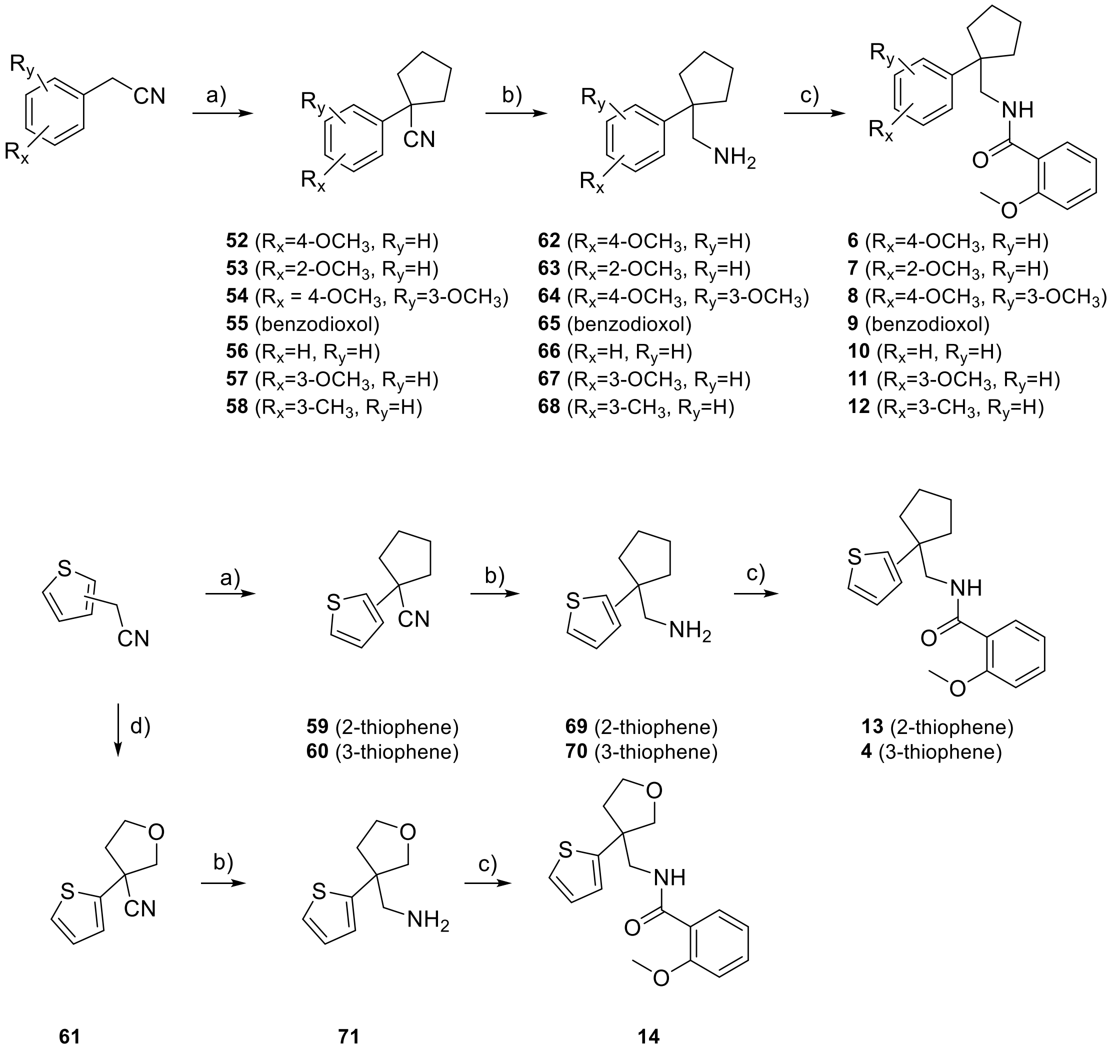

Figure S2. Synthesis of cyclopentane and tetrahydrofuran derivatives. Reagents, solvents and conditions: **a)** 1,4-dibromobutane, NaH, DMF, 0 °C and r.t., 2 to 3 days; **b)** $LiAlH_4$, THF, 0°C and r.t., 24 h; **c)** 2-methoxybenzoyl chloride, Et_3N, DCM, r.t., 24 h; **d)** 1-chloro-2-(chloromethoxy)ethane, NaH, DMF, -35 °C and r.t., 24 h.

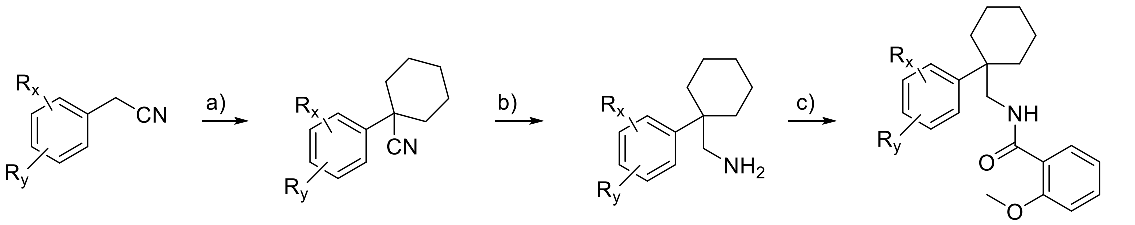

72 (R_x=4-OCH$_3$, R_y=3-OCH$_3$)
73 (R_x=4-OCH$_3$, R_y=H)
74 (R_x=4-CF$_3$, R_y=H)
75 (R_x=2-OCH$_3$, R_y=H)
76 (R_x=3-OCH$_3$, R_y=H)
77 (R_x=3-CH$_3$, R_y=H)

79 (R_x=4-OCH$_3$, R_y=3-OCH$_3$)
80 (R_x=4-OCH$_3$, R_y=H)
81 (R_x=4-CF$_3$, R_y=H)
82 (R_x=2-OCH$_3$, R_y=H)
83 (R_x=3-OCH$_3$, R_y=H)
84 (R_x=3-CH$_3$, R_y=H)

15 (R_x=4-OCH$_3$, R_y=3-OCH$_3$)
16 (R_x=4-OCH$_3$,R_y=H)
17 (R_x=4-CF$_3$, R_y=H)
18 (R_x=2-OCH$_3$, R_y=H)
19 (R_x=3-OCH$_3$,R_y=H)
20 (R_x=3-CH$_3$, R_y=H)

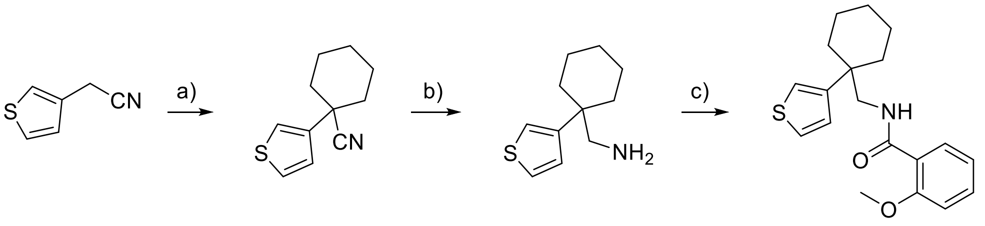

78 **85** **21**

Figure S3. Synthesis of cyclohexane derivatives. Reagents, solvents and conditions: **a)** 1,5-dibromopentane, NaH, DMF, 0 °C and r.t., 2 to 3 days; **b)** LiAlH$_4$, THF, 0°C and r.t., 24 h; **c)** 2-methoxybenzoyl chloride, Et$_3$N, DCM, r.t., 24 h.

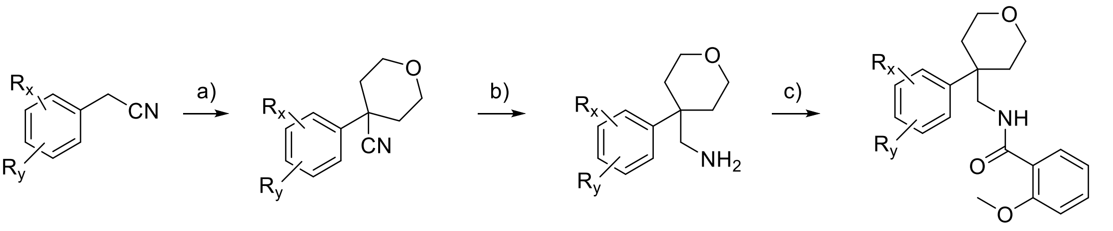

87 (R_x=4-CF$_3$, R_y=H)
88 (R_x=4-OCH$_3$, R_y=3-OCH$_3$)
89 (R_x=4-OCH$_3$, R_y=H)
90 (R_x=4-CH$_3$, R_y=H)
91 (benzodioxol)
92 (R_x=2-OCH$_3$, R_y=H
93 (R_x=3-OCH$_3$, R_y=H)
94 (R_x=3-CH$_3$, R_y=H)

96 (R_x=4-CF$_3$, R_y=H)
97 (R_x=4-OCH$_3$, R_y=3-OCH$_3$)
98 (R_x=4-OCH$_3$, R_y=H)
99 (R_x=4-CH$_3$, R_y=H)
100 (benzodioxol)
101 (R_x=2-OCH$_3$, R_y=H)
102 (R_x=3-OCH$_3$, R_y=H)
103 (R_x=3-CH$_3$, R_y=H)

22 (R_x=4-CF$_3$, R_y=H)
23 (R_x=4-OCH$_3$, R_y=3-OCH$_3$)
24 (R_x=4-OCH$_3$, R_y=H)
25 (R_x=4-CH$_3$, R_y=H)
26 (benzodioxol)
27 (R_x=2-OCH$_3$, R_y=H)
28 (R_x=3-OCH$_3$, R_y=H)
29 (R_x=3-CH$_3$, R_y=H)

Figure S4. Synthesis of benzene-based tetrahydropyran derivatives. Reagents, solvents and conditions: **a)** 1-chloro-2-(chloroethoxy)ethane, NaH, DMF, 0 °C and r.t., 2 to 3 days; **b)** LiAlH$_4$, THF, 0°C and r.t., 24 h; **c)** 2-methoxybenzoyl chloride, Et$_3$N, DCM, r.t., 24 h.

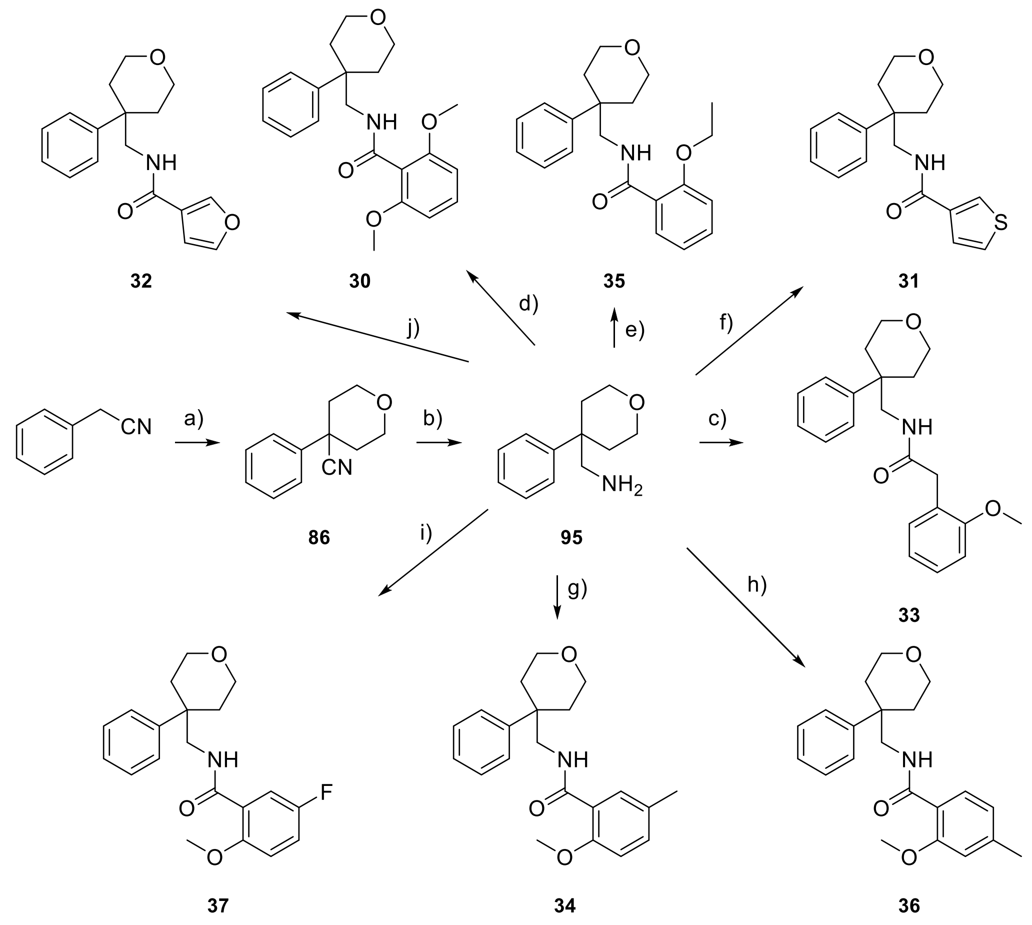

Figure S5. Synthesis of benzene-based tetrahydropyran derivatives. Reagents, solvents and conditions: **a)** 1-chloro-2-(chloroethoxy)ethane, NaH, DMF, 0 °C and r.t., 2 to 3 days; **b)** $LiAlH_4$, THF, 0°C and r.t., 24 h; **c)** 2-(2-methoxyphenyl)acetyl chloride, Et_3N, DCM, r.t., 24 h; **d)** 2,6-dimethoxybenzoyl chloride, Et_3N, DCM, r.t., 24 h; **e)** 2-ethoxybenzoyl chloride, Et_3N, DCM, r.t., 24 h; **f)** thiophene-3-carbonyl chloride, Et_3N, DCM, r.t., 24 h; **g)** 2-methoxy-5-methylbenzoyl chloride, Et_3N, DCM, r.t., 24 h; **h)** 2-methoxy-4-methylbenzoyl chloride, Et_3N, DCM, r.t., 24 h; **i)** 5-fluoro-2-methoxybenzoyl chloride, Et_3N, DCM, r.t., 24 h; **j)** furan-3-carbonyl chloride, Et_3N, DCM, r.t., 24 h.

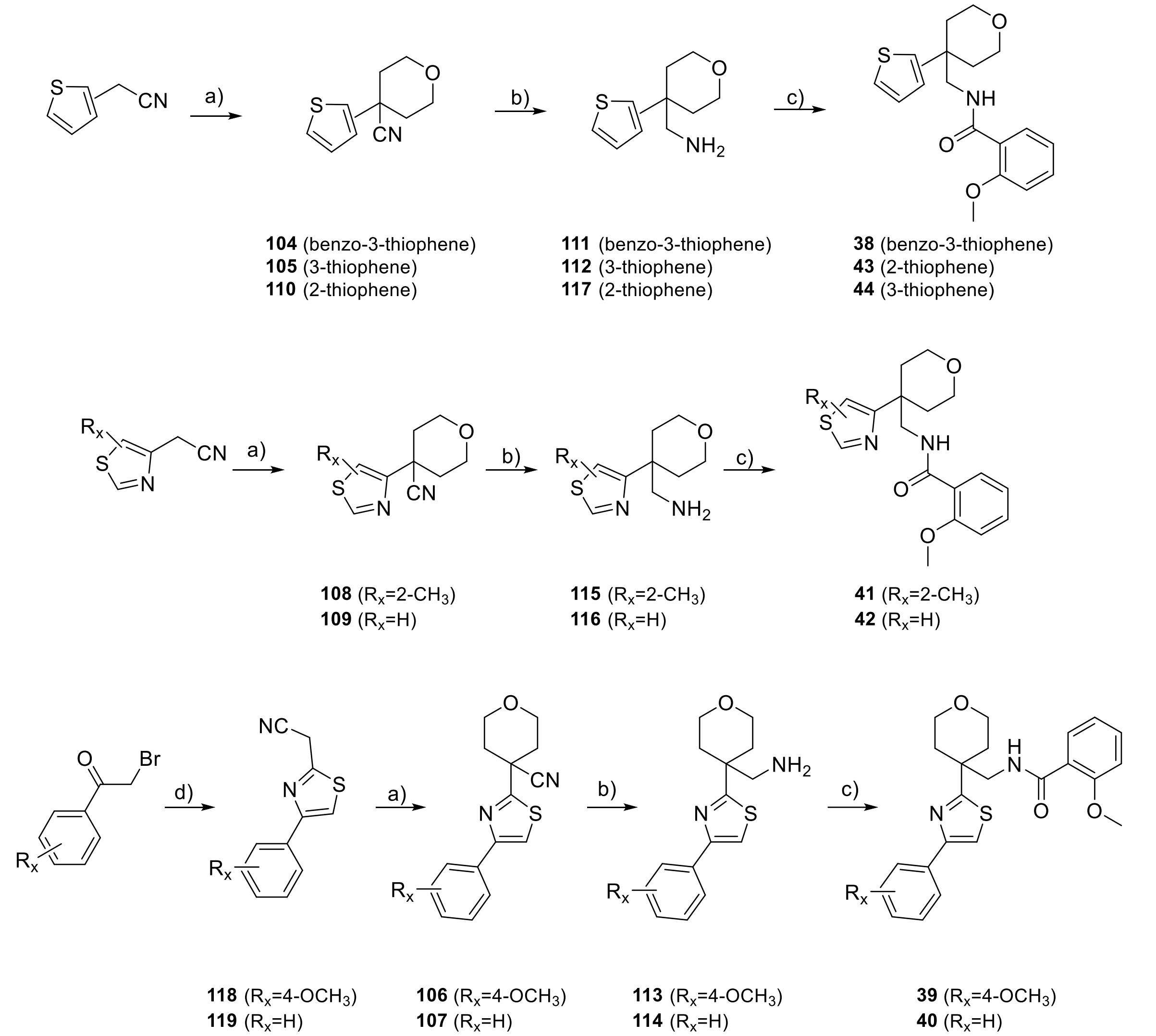

Figure S6. Synthesis of heterocyclic tetrahydropyran derivatives. Reagents, solvents and conditions: **a)** 1-chloro-2-(chloroethoxy)ethane, NaH, DMF, 0 °C and r.t., 2 to 3 days; **b)** $LiAlH_4$, THF, 0°C and r.t., 24 h; **c)** 2-methoxybenzoyl chloride, Et_3N, DCM, r.t., 24 h; **d)** 2-cyanoethanethioamide, TEA, DMF, 0°C and r.t. overnight.

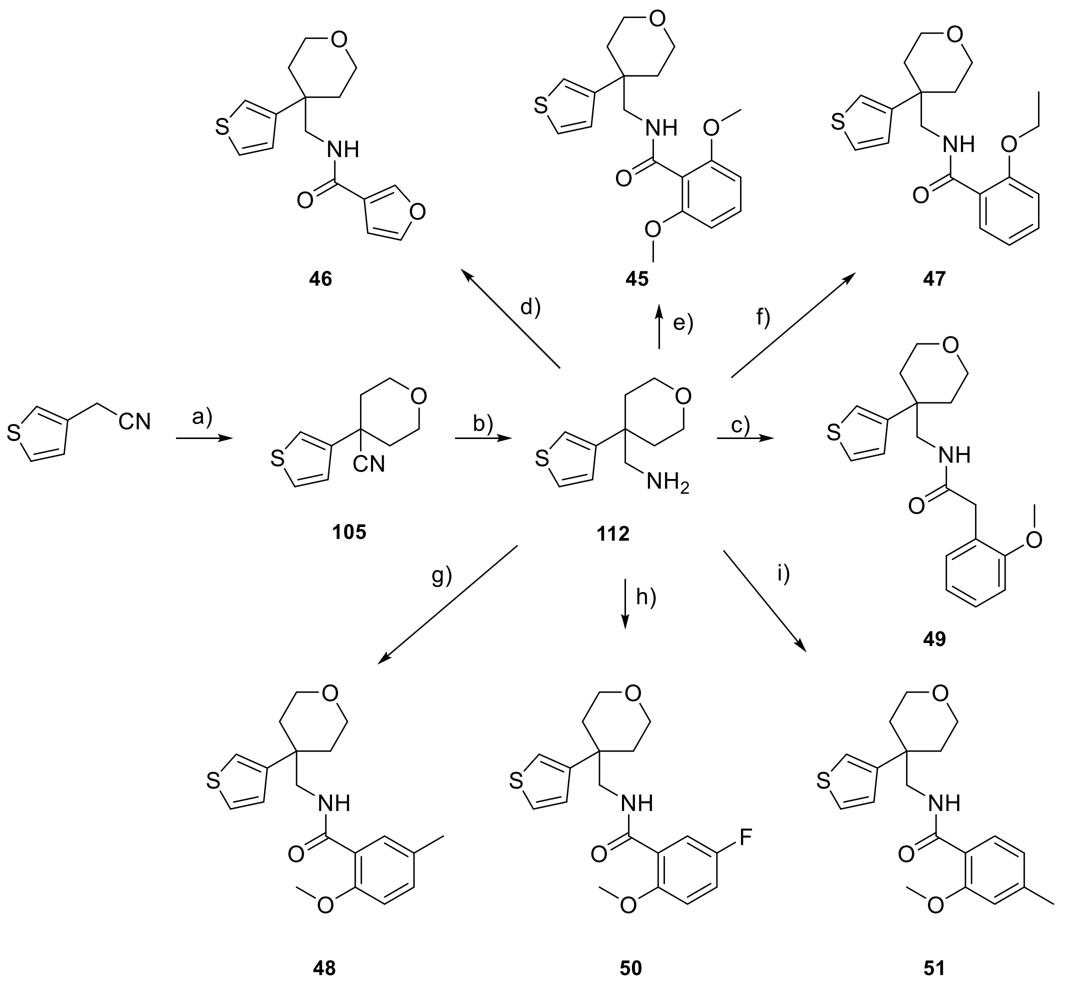

Figure S7. Synthesis of 3-thiophen-based tetrahydropyran derivatives. Reagents, solvents and conditions: **a)** 1-chloro-2-(chloroethoxy)ethane, NaH, DMF, 0 °C and r.t., 2 to 3 days; **b)** $LiAlH_4$, THF, 0°C and r.t., 24 h; **c)** 2-(2-methoxyphenyl)acetyl chloride, Et_3N, DCM, r.t., 24 h; **d)** furan-3-carbonyl chloride, Et_3N, DCM, r.t., 24 h; **e)** 2,6-dimethoxybenzoyl chloride, Et_3N, DCM, r.t., 24 h; **f)** 2-ethoxybenzoyl chloride, Et_3N, DCM, r.t., 24 h; **g)** 2-methoxy-4-methylbenzoyl chloride, Et_3N, DCM, r.t., 24 h; **h)** 5-fluoro-2-methoxybenzoyl chloride, Et_3N, DCM, r.t., 24 h; **i)** 2-methoxy-4-methylbenzoyl chloride, Et_3N, DCM, r.t., 24 h.

General procedure A: Synthesis of benzamide analogues 4, 6, 7, 8, 9, 10, 11, 12, 13, and 14 (with 6 given as an example).

2-Methoxybenzoic acid (190 mg, 1.25 mmol, 1.0 equiv) was dissolved in dichloromethane (20 mL) and the batch was stirred while ice cooling for 10 minutes. Oxalyl chloride (0.330 mL, 3.75 mmol, 3.0 equiv) was added dropwise on ice bath followed by the addition of 5 drops of DMF. The batch was stirred at room temperature overnight and next day the solvent was evaporated. (1-(4-Methoxyphenyl)cyclopentyl)methanamine (257 mg, 1.25 mmol, 1.0 equiv) and Et_3N (0.52 mL, 3.75 mmol, 3.0 equiv) were dissolved in dichloromethane (75 mL). Benzoyl chloride intermediate (1.25 mmol), dissolved in dichloromethane (20 mL), was then added dropwise. Batch was stirred at room temperature overnight. Next day organic phase was diluted with dichloromethane (50 mL) and washed with saturated aqueous $NaHCO_3$ solution (2 x 25 mL), 1M aqueous HCl solution (1 x 25 mL), water (2 x 25 mL), saturated brine solution (25 mL), dried over Na_2SO_4, and organic phase was removed under reduced pressure. Product was purified with column chromatography using ethyl acetate:*n*-hexane = 1:4 as mobile phase and additionally using Biotage Isolera One System reversed-phase chromatography (Biotage SNAP Cartridge KP-C18-HS 12 g column), MF: gradient water in H_2O/acetonitril. 171 mg of product was isolated.

2-Methoxy-*N*-((1-(thiophen-3-yl)cyclopentyl)methyl)benzamide (4)

Synthesized from (1-(thiophen-3-yl)cyclopentyl)methanamine (308 mg, 1.70 mmol, 1.0 equiv), Et_3N (0.71 mL, 5.10 mmol, 3.0 equiv) and 2-methoxybenzoyl chloride intermediate (1.70 mmol) according to general procedure A. Column chromatography, EtOAc/n-hex = 1/5 (v/v). Yield: 58.1 %; white solid (311 mg); ^1H NMR (400 MHz, $CDCl_3$) δ 8.20 (dd, J_1 =

7.8 Hz, J_2 = 1.9 Hz, 1H), 7.75 (brs, 1H), 7.38 (ddd, J_1 = 8.3 Hz, J_2 = 7.3 Hz, J_3 = 1.9 Hz, 1H), 7.33 (dd, J_1 = 5.0 Hz, J_2 = 2.9 Hz, 1H), 7.11 – 7.00 (m, 3H), 6.87 (d, J = 7.9 Hz, 1H), 3.69 (s, 3H), 3.66 (d, J = 5.6 Hz, 2H), 1.95 – 1.88 (m, 4H), 1.88 – 1.77 (m, 2H), 1.77 – 1.66 (m, 2H); ^{13}C NMR (101 MHz, $CDCl_3$) δ 165.25, 157.52, 148.82, 132.62, 132.36, 127.06, 125.82, 121.42, 121.19, 119.87, 111.21, 55.57, 49.63, 48.59, 36.66, 24.00 ppm; HRMS (ESI$^+$) for $C_{18}H_{22}NO2S$ ([M+H]$^+$) calculated 316.1366, found 316.1360; HPLC retention time: 5.877 min (99.55 % at 254 nm).

2-Methoxy-*N*-((1-(4-methoxyphenyl)cyclopentyl)methyl)benzamide (6).
Synthesized from (1-(4-methoxyphenyl)cyclopentyl)methanamine (257 mg, 1.25 mmol, 1.0 equiv), Et_3N (0.52 mL, 3.76 mmol, 3.0 equiv) and 2-methoxybenzoyl chloride intermediate (1.25 mmol) according to general procedure A. Column chromatography, EtOAc/*n*-hex = 1/5 (v/v). and Biotage Isolera One System reversed-phase chromatography (Biotage SNAP Cartridge KP-C18-HS 12 g column), MF: gradient water in H_2O/acetonitril. Yield: 40.3 %; white solid (171 mg); ^{1}H NMR (400 MHz, $CDCl_3$) δ 8.20 (dd, J_1 = 7.8 Hz, J_2 = 1.8 Hz, 1H), 7.66 (brs, 1H), 7.39 (ddd, J_1 = 8.3 Hz, J_2 = 7.3 Hz, J_3 = 1.9 Hz, 1H), 7.31 – 7.25 (m, 2H), 7.04 (td, J_1 = 7.8 Hz, J_2 = 1.0 Hz, 1H), 6.93 – 6.89 (m, 2H), 6.87 (d, J = 7.8 Hz, 1H), 3.82 (s, 3H), 3.65 (s, 3H), 3.63 (d, J = 5.7 Hz, 2H), 2.05 – 1.94 (m, 2H), 1.93 – 1.82 (m, 4H), 1.78 – 1.69 (m, 2H); ^{13}C NMR (101 MHz, $CDCl_3$) δ 165.25, 157.87, 157.46, 139.44, 132.57, 132.34, 128.11, 121.41, 121.14, 113.66, 111.13, 55.49, 55.34, 51.01, 48.91, 35.95, 23.62 ppm; HRMS (ESI$^+$) for $C_{21}H_{26}NO_3$ ([M+H]$^+$) calculated 340.1907, found 340.1904; HPLC retention time: 5.927 min (98.33 % at 254 nm).

2-Methoxy-*N*-((1-(2-methoxyphenyl)cyclopentyl)methyl)benzamide (7)
Synthesized from (1-(2-methoxyphenyl)cyclopentyl)methanamine (2.67 g, 13.0 mmol, 1.0 equiv), Et_3N (5.44 mL, 39.0 mmol, 3.0 equiv) and 2-methoxybenzoyl chloride intermediate (13.0 mmol) according to general procedure A. Column chromatography, EtOAc/*n*-hex = 1/5 (v/v). Yield: 61.1 %; white solid (2.7 g); ^{1}H NMR (400 MHz, $CDCl_3$) δ 8.18 (dd, J_1 = 7.8 Hz, J_2 = 1.9 Hz, 1H), 7.50 (brs, 1H), 7.36 (ddd, J_1 = 8.3 Hz, J_2 = 7.3 Hz, J_3 = 1.9 Hz, 1H), 7.30 – 7.23 (m, 2H), 7.02 (td, J_1 = 7.8 Hz, J_2 = 1.0 Hz, 1H), 6.97 – 6.89 (m, 2H), 6.83 (dd, J_1 = 8.3 Hz, J_2 = 0.6 Hz, 1H), 3.83 (s, 3H), 3.80 (d, J = 5.6 Hz, 2H), 3.56 (s, 3H), 2.19 – 2.09 (m, 2H), 1.92 – 1.78 (m, 4H), 1.75 – 1.63 (m, 2H); ^{13}C NMR (101 MHz, $CDCl_3$) δ 165.21, 158.29, 157.49, 134.58, 132.41, 132.31, 128.72, 127.77, 121.62, 121.07, 120.11, 111.41, 111.09, 55.39, 55.15, 50.76, 44.79, 35.39, 23.47 ppm; HRMS (ESI$^+$) for $C_{21}H_{26}NO_3$ ([M+H]$^+$) calculated 340.1907, found 340.1901; HPLC retention time: 6.203 min (98.70 % at 254 nm).

***N*-((1-(3,4-Dimethoxyphenyl)cyclopentyl)methyl)-2-methoxybenzamide (8)**
Synthesized from (1-(3,4-dimethoxyphenyl)cyclopentyl)methanamine **(**236 mg, 1.0 mmol, 1.0 equiv), Et_3N (0.42 mL, 3.0 mmol, 3.0 equiv) and 2-methoxybenzoyl chloride intermediate (1.0 mmol **)** according to general procedure A. Column chromatography, EtOAc/*n*-hex = 1/4 (v/v) and Biotage Isolera One System reversed-phase chromatography (Biotage SNAP Cartridge KP-C18-HS 12 g column), MF: gradient water in H_2O/acetonitril. Yield: 39.1 %; white solid (145 mg); ^{1}H NMR (400 MHz, $CDCl_3$) δ 8.21 (dd, J_1 = 7.8 Hz, J_2 = 1.8 Hz, 1H), 7.67 (brs, 1H), 7.40 (ddd, J_1 = 8.3 Hz, J_2 = 7.3 Hz, J_3 = 1.9 Hz, 1H), 7.05 (td, J_1 = 7.8 Hz, J_2 = 1.0 Hz, 1H), 6.93 – 6.84 (m, 4H), 3.89 (s, 3H), 3.87 (s, 3H), 3.65 (d, J = 4.3 Hz, 2H), 3.64 (s, 3H), 2.05 – 1.95 (m, 2H), 1.94 – 1.83 (m, 4H), 1.81 – 1.70 (m, 2H); ^{13}C NMR (101 MHz, $CDCl_3$) δ 165.26, 157.47, 148.72, 147.40, 140.16, 132.64, 132.40, 121.42, 121.23, 119.16, 111.17, 111.00, 110.83, 56.05, 55.97, 55.45, 51.47, 48.93, 35.98, 23.68 ppm; HRMS (ESI$^+$) for $C_{22}H_{28}NO_4$ ([M+H]$^+$) calculated 370.2013, found 370.2003; HPLC retention time: 5.393 min (98.32 % at 254 nm).

***N*-((1-(Benzo[*d*][1,3]dioxol-5-yl)cyclopentyl)methyl)-2-methoxybenzamide (9)**
Synthesized from (1-(benzo[*d*][1,3]dioxol-5-yl)cyclopentyl)methanamine (152 mg, 0.7 mmol, 1.0 equiv), Et_3N (0.29 mL, 2.1 mmol, 3.0 equiv) and 2-methoxybenzoyl chloride intermediate (0.7 mmol) according to general procedure A. Column chromatography, EtOAc/*n*-hex = 1/5 (v/v). Yield: 65.1 %; white solid (160 mg); ^{1}H NMR (400 MHz, $CDCl_3$) δ 8.19 (dd, J_1 = 7.8 Hz, J_2 = 1.8 Hz, 1H), 7.68 (brs, 1H), 7.42 – 7.36 (m, 1H), 7.03 (td, J_1 = 7.9, J_2 = 1.0 Hz, 1H), 6.90 – 6.86 (m, 2H), 6.81 (d, J = 1.1 Hz, 2H), 5.94 (s, 2H), 3.72 (s, 3H), 3.60 (d, J = 5.7 Hz, 2H), 2.01 – 1.94 (m, 2H), 1.91 – 1.79 (m, 4H), 1.78 – 1.69 (m, 2H); ^{13}C NMR (101 MHz, $CDCl_3$) δ 165.29, 157.49, 147.67, 145.77, 141.67, 132.63, 132.42, 121.42, 121.20, 119.99, 111.15, 107.98, 107.91, 100.97, 55.51, 51.60, 48.87, 35.98, 23.52 ppm; HRMS (ESI$^+$) for $C_{21}H_{24}NO_4$ ([M+H]$^+$) calculated 354.1700, found 354.1693; HPLC retention time: 5.783 min (98.29 % at 254 nm).

2-Methoxy-*N*-((1-phenylcyclopentyl)methyl)benzamide (10).

Synthesized from (1-phenylcyclopentyl)methanamine (257 mg, 1.47 mmol, 1.0 equiv), Et_3N (0.61 mL, 4.4 mmol, 3.0 equiv) and 2-methoxybenzoyl chloride intermediate (1.47 mmol) according to general procedure A. Column chromatography, EtOAc/*n*-hex = 1/5 (v/v). Yield: 58.8 %; white solid (267 mg); ^1H NMR (400 MHz, $CDCl_3$) δ 8.20 (dd, J_1 = 7.8 Hz, J_2 = 1.9 Hz, 1H), 7.66 (brs, 1H), 7.40 – 7.34 (m, 5H), 7.28 – 7.22 (m, 1H), 7.05 – 7.00 (m, 1H), 6.85 (dd, J_1 = 8.3, J_2 = 0.6 Hz, 1H), 3.67 (d, J = 5.7 Hz, 2H), 3.60 (s, 3H), 2.08 – 1.98 (m, 2H), 1.95 – 1.83 (m, 4H), 1.80 – 1.69 (m, 2H); ^{13}C NMR (101 MHz, $CDCl_3$) δ 165.32, 157.53, 147.56, 132.63, 132.43, 128.39, 127.21, 126.16, 121.43, 121.21, 111.17, 55.46, 51.80, 48.88, 35.78, 23.66 ppm; HRMS (ESI$^+$) for $C_{20}H_{24}NO_2$ ([M+H]$^+$) calculated 310.1802, found 310.1798; HPLC retention time: 6.020 min (95.37 % at 254 nm).

2-Methoxy-*N*-((1-(3-methoxyphenyl)cyclopentyl)methyl)benzamide (11)
Synthesized from (1-(3-methoxyphenyl)cyclopentyl)methanamine (414 mg, 2.0 mmol, 1.0 equiv), Et_3N (0.84 mL, 6.0 mmol, 3.0 equiv) and 2-methoxybenzoyl chloride (2.0 mmol) intermediate according to general procedure A. Column chromatography, EtOAc/*n*-hex = 1/5 (v/v). Yield: 51.1 %; white solid (350 mg); ^1H NMR (400 MHz, $CDCl_3$) δ 8.20 (dd, J_1 = 7.8 Hz, J_2 = 1.8 Hz, 1H), 7.69 (brs, 1H), 7.38 (ddd, J_1 = 8.3 Hz, J_2 = 7.3 Hz, J_3 = 1.9 Hz, 1H), 7.29 (t, J = 7.9 Hz, 1H), 7.06 – 7.00 (m, 1H), 6.96 (ddd, J_1 = 7.7 Hz, J_2 = 1.6 Hz, J_3 = 0.9 Hz, 1H), 6.93 – 6.89 (m, 1H), 6.86 (d, J = 7.8 Hz, 1H), 6.80 (ddd, J_1 = 8.2 Hz, J_2 = 2.5 Hz, J_3 = 0.7 Hz, 1H), 3.81 (s, 3H), 3.65 (d, J = 5.7 Hz, 2H), 3.65 (s, 3H), 2.05 – 1.95 (m, 2H), 1.94 – 1.81 (m, 4H), 1.81 – 1.68 (m, 2H); ^{13}C NMR (101 MHz, $CDCl_3$) δ 165.31, 159.59, 157.56, 149.36, 132.62, 132.41, 129.35, 121.40, 121.17, 119.66, 113.71, 111.15, 110.81, 55.38, 55.23, 51.82, 48.85, 35.73, 23.62 ppm; HRMS (ESI$^+$) for $C_{21}H_{26}NO_3$ ([M+H]$^+$) calculated 340.1907, found 340.1894; HPLC retention time: 5.917 min (98.67 % at 254 nm).

2-Methoxy-*N*-((1-(*m*-tolyl)cyclopentyl)methyl)benzamide (12)
Synthesized from (1-(*m*-tolyl)cyclopentyl)methanamine (270 mg, 1.43 mmol, 1.0 equiv), Et_3N (0.60 mL, 4.28 mmol, 3.0 equiv) and 2-methoxybenzoyl chloride intermediate (1.43 mmol) according to general procedure A. Column chromatography, EtOAc/*n*-hex = 1/5 (v/v). Yield: 59.2 %; white solid (273 mg); ^1H NMR (400 MHz, $CDCl_3$) δ 8.20 (dd, J_1 = 7.8 Hz, J_2 = 1.9 Hz, 1H), 7.66 (brs, 1H), 7.37 (ddd, J_1 = 8.3 Hz, J_2 = 7.3 Hz, J_3 = 1.9 Hz, 1H), 7.28 – 7.22 (m, 1H), 7.16 (d, J = 6.6 Hz, 2H), 7.09 – 7.05 (m, 1H), 7.02 (td, J_1 = 7.9 Hz, J_2 = 1.0 Hz, 1H), 6.88 – 6.83 (m, 1H), 3.65 (d, J = 5.7 Hz, 2H), 3.60 (s, 3H), 2.37 (s, 3H), 2.06 – 1.96 (m, 2H), 1.94 – 1.83 (m, 4H), 1.77 – 1.70 (m, 2H); ^{13}C NMR (101 MHz, $CDCl_3$) δ 165.27, 157.50, 147.46, 137.80, 132.58, 132.38, 128.20, 127.94, 126.88, 124.21, 121.45, 121.17, 111.14, 55.35, 51.59, 48.89, 35.76, 23.64, 21.75 ppm; HRMS (ESI$^+$) for $C_{21}H_{26}NO_2$ ([M+H]$^+$) calculated 324.1958, found 324.1945; HPLC retention time: 6.380 min (99.29 % at 254 nm).

2-Methoxy-*N*-((1-(thiophen-2-yl)cyclopentyl)methyl)benzamide (13)
Synthesized from (1-(thiophen-2-yl)cyclopentyl)methanamine (217 mg, 1.20 mmol, 1.0 equiv), Et_3N (0.5 mL, 3.59 mmol, 3.0 equiv)and 2-methoxybenzoyl chloride intermediate (1.20 mmol) according to general procedure A. Column chromatography, EtOAc/*n*-hex = 1/5 (v/v). Yield: 59.5 %; white solid (225 mg); ^1H NMR (400 MHz, $CDCl_3$) δ 8.20 (dd, J_1 = 7.8 Hz, J_2 = 1.8 Hz, 1H), 7.90 (brs, 1H), 7.40 (ddd, J_1 = 8.3 Hz, J_2 = 7.3 Hz, J_3 = 1.9 Hz, 1H), 7.22 (dd, J_1 = 5.1 Hz, J_2 = 1.2 Hz, 1H), 7.05 (td, J_1 = 7.8 Hz, J_2 = 1.0 Hz, 1H), 7.00 (dd, J_1 = 5.1 Hz, J_2 = 3.5 Hz, 1H), 6.93 (dd, J_1 = 3.5 Hz, J_2 = 1.2 Hz, 1H), 6.89 (d, J = 8.3 Hz, 1H), 3.72 (d, J = 5.7 Hz, 2H), 3.70 (s, 3H), 2.04 – 1.93 (m, 4H), 1.90 – 1.71 (m, 4H); ^{13}C NMR (101 MHz, $CDCl_3$) δ 165.32, 157.57, 152.67, 132.67, 132.37, 126.71, 123.58 (2 × Ar-$\underline{C}$), 121.43, 121.21, 111.25, 55.53, 50.36, 49.68, 38.22, 24.11 ppm; HRMS (ESI$^+$) for $C_{18}H_{22}NO_2S$ ([M+H]$^+$) calculated 316.1366, found 316.1363; HPLC retention time: 5.893 min (98.44 % at 254 nm).

2-Methoxy-*N*-((3-(thiophen-2-yl)tetrahydrofuran-3-yl)methyl)benzamide (14)
Synthesized from (3-(thiophen-2-yl)tetrahydrofuran-3-yl)methanamine (222 mg, 1.21 mmol, 1.0 equiv), Et_3N (0.51 mL, 3.63 mmol, 3.0 equiv) and 2-methoxybenzoyl chloride intermediate (1.21 mmol) according to general procedure A. Column chromatography, EtOAc/*n*-hex = 1/5 (v/v). Yield: 61.1 %; white solid (235 mg); ^1H NMR (400 MHz, $CDCl_3$) δ 8.20 (dd, J_1 = 7.8 Hz, J_2 = 1.8 Hz, 1H), 8.05 (brs, 1H), 7.41 (ddd, J_1 = 8.4 Hz, J_2 = 7.4 Hz, J_3 = 1.9 Hz, 1H), 7.27 (dd, J_1 = 5.1 Hz, J_2 = 1.1 Hz, 1H), 7.08 – 7.02 (m, 2H), 6.97 (dd, J_1 = 3.5 Hz, J_2 = 1.1 Hz, 1H), 6.90 (d, J = 8.3 Hz, 1H), 4.16 – 4.08 (m, 1H), 4.05 – 3.92 (m, 4H), 3.82 – 3.73 (m, 1H), 3.70 (s, 3H), 2.34 – 2.26 (m, 2H); ^{13}C NMR (101 MHz, $CDCl_3$) δ 165.49, 157.53, 147.90, 132.87, 132.34, 127.02, 124.19, 124.15, 121.27, 121.15, 111.29, 77.27, 67.71, 55.55, 50.44, 47.78, 37.34 ppm; HRMS (ESI$^+$) for $C_{17}H_{20}NO_3S$ ([M+H]$^+$) calculated 318.1158, found 318.1156; HPLC retention time: 4.047 min (97.3 % at 254 nm).

General procedure B: Synthesis of cyclopentane analogues 52, 53, 54, 55, 56, 57, 58, 59, and 60.

2-(4-Methoxyphenyl)acetonitrile (320 mg, 2.2 mmol) was slowly added to a stirred solution of NaH (60 % dispersion in mineral oil) (176 mg, 4.4 mmol) in anhydrous DMF (30 mL) while ice cooling under argon atmosphere. The batch was stirred for 15 minutes and then solution 1,4-dibromobutane (0.32 mL, 2.64 mmol) in anhydrous DMF (20 mL) was added dropwise while ice cooling. The batch was stirred at room temperature overnight. Next day water (50 mL) was added to the reaction mixture and washed with diethylether (3 x 100 mL). Combined organic phases were washed with saturated brine solution (2 x 100 mL), dried over Na_2SO_4, filtered and then solvent was removed under reduced pressure. Product was purified with column chromatography using ethyl acetate:*n*-hexane = 1:6 as mobile phase. 319 mg of uncoloured oil was isolated.

1-(4-Methoxyphenyl)cyclopentane-1-carbonitrile (52)

Synthesized from 2-(4-methoxyphenyl)acetonitrile (319 mg, 2.17 mmol, 1.0 equiv), 1,4-dibromobutane (0.31 mL, 2.60 mmol, 1.2 equiv), and NaH (174 mg, 4.34 mmol, 2.0 equiv) according to general procedure B. Column chromatography, EtOAc/*n*-hex = 1/6 (v/v). Yield: 73.2 %; oil (319 mg); ^{1}H NMR (400 MHz, DMSO) δ 7.43 – 7.37 (m, 2H), 6.99 – 6.94 (m, 2H), 3.76 (s, 3H), 2.41 – 2.30 (m, 2H), 2.08 – 1.95 (m, 2H), 1.91 – 1.81 (m, 4H).

1-(2-Methoxyphenyl)cyclopentane-1-carbonitrile (53)

Synthesized from 2-(2-methoxyphenyl)acetonitrile (4.57 g, 31.04 mmol, 1.0 equiv), 1,4-dibromobutane (4.45 mL, 37.25 mmol, 1.2 equiv), and NaH (2.48 g, 62.08 mmol, 2.0 equiv) according to general procedure B. Column chromatography, EtOAc/*n*-hex = 1/6 (v/v). Yield: 52.5 %; oil (3.28 g); ^{1}H NMR (400 MHz, DMSO) δ 7.39 – 7.29 (m, 2H), 7.11 (dd, J_1 = 8.2 Hz, J_2 = 1.1 Hz, 1H), 6.97 (td, J_1 = 7.6 Hz, J_2 = 1.2 Hz, 1H), 3.87 (s, 3H), 2.48 – 2.40 (m, 2H), 2.10 – 1.99 (m, 2H), 1.90 – 1.73 (m, 4H).

1-(3,4-Dimethoxyphenyl)cyclopentane-1-carbonitrile (54)

Synthesized from 2-(3,4-dimethoxyphenyl)acetonitrile (324 mg, 1.83 mmol, 1.0 equiv), 1,4-dibromobutane (0.26 mL, 2.19 mmol, 1.2 equiv), and NaH (146 mg, 3.66 mol, 2.0 equiv) according to general procedure B. Column chromatography, EtOAc/*n*-hex = 1/6 (v/v). Yield: 68.8 %; oil (291 mg); ^{1}H NMR (400 MHz, DMSO) δ 7.04 – 6.99 (m, 2H), 6.98 – 6.94 (m, 1H), 3.79 (s, 3H), 3.76 (s, 3H), 2.42 – 2.34 (m, 2H), 2.11 – 2.01 (m, 2H), 1.91 – 1.84 (m, 4H).

1-(Benzo[*d*][1,3]dioxol-5-yl)cyclopentane-1-carbonitrile (55)

Synthesized from 2-(benzo[*d*][1,3]dioxol-5-yl)acetonitrile (185 mg, 1.15 mmol, 1.0 equiv), 1,4-dibromobutane (0.16 mL, 1.38 mmol, 1.2 equiv), and NaH (92 mg, 2.3 mmol, 2.0 equiv) according to general procedure B. Column chromatography, EtOAc/*n*-hex = 1/6 (v/v): 79.8 %; oil (197 mg); ^{1}H NMR (400 MHz, DMSO) δ 7.08 (d, *J* = 1.8 Hz, 1H), 6.97 (dd, J_1 = 8.1 Hz, J_2 = 1.9 Hz, 1H), 6.94 – 6.90 (m, 1H), 6.04 (s, 2H), 2.40 – 2.32 (m, 2H), 2.06 – 1.96 (m, 2H), 1.90 – 1.81 (m, 4H).

1-Phenylcyclopentane-1-carbonitrile (56)

Synthesized from 2-phenylacetonitrile (241 mg, 2.1 mmol, 1.0 equiv), 1,4-dibromobutane (0.30 mL, 2.52 mmol, 1.2 equiv), and NaH (168 mg, 4.2 mmol, 2.0 equiv) according to general procedure B. Column chromatography, EtOAc/*n*-hex = 1/6 (v/v). Yield: 83.1 %; oil (293 mg); ^{1}H NMR (400 MHz, DMSO) δ 7.53 – 7.47 (m, 2H), 7.45 – 7.38 (m, 2H), 7.38 – 7.31 (m, 1H), 2.44 – 2.34 (m, 2H), 2.11 – 2.00 (m, 2H), 1.93 – 1.83 (m, 4H).

1-(3-Methoxyphenyl)cyclopentane-1-carbonitrile (57 PSG-216)

Synthesized from 2-(3-methoxyphenyl)acetonitrile (493 mg, 3.35 mmol, 1.0 equiv), 1,4-dibromobutane (0.48 mL, 4.02 mmol, 1.2 equiv), and NaH (257 mg, 6.70 mmol, 2.0 equiv) according to general procedure B. Column chromatography, EtOAc/*n*-hex = 1/6 (v/v). Yield: 73.6 %; oil (496 mg); ^{1}H NMR (400 MHz, DMSO) δ 7.34 (t, *J* = 8.0 Hz, 1H), 7.07 (ddd, J_1 = 7.8 Hz, J_2 = 1.8 Hz, J_3 = 0.8 Hz, 1H), 7.03 – 7.00 (m, 1H), 6.93 (ddd, J_1 = 8.3 Hz, J_2 = 2.5 Hz, J_3 = 0.8 Hz, 1H), 3.78 (s, 3H), 2.43 – 2.34 (m, 2H), 2.12 – 2.02 (m, 2H), 1.92 – 1.83 (m, 4H).

1-(*m*-Tolyl)cyclopentane-1-carbonitrile (58)

Synthesized from 2-(*m*-tolyl)acetonitrile (277 mg, 2.11 mmol, 1.0 equiv), 1,4-dibromobutane (0.3 mL, 2.53 mmol, 1.2 equiv), and NaH (169 mg, 4.22 mmol, 2.0 equiv) according to general procedure B. Column chromatography, EtOAc/*n*-hex = 1/6 (v/v). Yield: 72.8 %; oil (285 mg); ^{1}H NMR (400 MHz, DMSO) δ 7.33 – 7.27 (m, 3H), 7.18 – 7.13 (m, 1H), 2.42 – 2.35 (m, 2H), 2.33 (s, 3H), 2.10 – 2.00 (m, 2H), 1.91 – 1.85 (m, 4H).

1-(Thiophen-2-yl)cyclopentane-1-carbonitrile (59)

Synthesized from 2-(thiophen-2-yl)acetonitrile (257 mg, 2.1 mmol, 1.0 equiv), 1,4-dibromobutane (0.30 mL, 2.52 mmol, 1.2 equiv), and NaH (168 mg, 4.2 mmol, 2.0 equiv) according to general procedure B. Column chromatography, EtOAc/*n*-hex = 1/6 (v/v). Yield: 71.8 %; oil (266 mg); ^{1}H NMR (400 MHz, DMSO) δ 7.54 (dd, J_1 = 5.1 Hz, J_2 = 1.2 Hz, 1H), 7.19 (dd, J_1 = 3.6 Hz, J_2 = 1.2 Hz, 1H), 7.04 (dd, J_1 = 5.1 Hz, J_2 = 3.6 Hz, 1H), 2.47 – 2.39 (m, 2H), 2.18 – 2.09 (m, 2H), 1.91 – 1.80 (m, 4H).

1-(Thiophen-3-yl)cyclopentane-1-carbonitrile (60)

Synthesized from 2-(thiophen-3-yl)acetonitrile (370 mg, 3.0 mmol, 1.0 equiv), 1,4-dibromobutane (0.43 mL, 3.6 mmol, 1.2 equiv), and NaH (240 mg, 6.0 mmol, 2.0 equiv) according to general procedure B. Column chromatography, EtOAc/*n*-hex = 1/6 (v/v). Yield: 71.8 %; oil (382 mg); ^{1}H NMR (400 MHz, DMSO) δ 7.61 (dd, J_1 = 5.0 Hz, J_2 = 2.9 Hz, 1H), 7.55 (dd, J_1 = 2.9 Hz, J_2 = 1.5 Hz, 1H), 7.22 (dd, J_1 = 5.0 Hz, J_2 = 1.4 Hz, 1H), 2.38 – 2.29 (m, 2H), 2.13 – 2.02 (m, 2H), 1.89 – 1.78 (m, 4H).

Synthesis of compound 61 (3-(thiophen-2-yl)tetrahydrofuran-3-carbonitrile)

2-(Thiophen-2-yl)acetonitrile (2.22 mL, 20 mmol, 1.0 equiv) was slowly added to a stirred solution of NaH (60 % dispersion in mineral oil) (2.4 g, 60 mmol, 2.0 equiv) in anhydrous DMF (20 mL) under argon atmosphere while ice cooling at -35 °C. Solution of 1-chloro-2-(chloromethoxy)ethane (2.02 mL, 20 mmol, 1.0 equiv) dissolved in anhydrous DMF (20 mL) was added slowly while ice cooling at -35°C. The batch was stirred at room temperature overnight. For workup, water (50 mL) was added to the reaction mixture and the reaction mixture was washed with diethylether (3 x 100 mL). Combined organic phases were washed with saturated brine solution (2 x 100 mL), dried over Na_2SO_4, filtered and then solvent was removed under reduced pressure. Product was purified with column chromatography using ethyl acetate:*n*-hexane = 1:6 as mobile phase. 225 mg of yellow oil was isolated. Yield: 6.3 %; yellow oil (225 mg); ^{1}H NMR (400 MHz, $CDCl_3$) δ 7.30 (dd, J_1 = 5.2 Hz, J_1 = 1.2 Hz, 1H), 7.19 (dd, J_1 = 3.6 Hz, J_2 = 1.2 Hz, 1H), 7.00 (dd, J_1 = 5.1 Hz, J_2 = 3.6 Hz, 1H), 4.35 (d, J = 8.9 Hz, 1H), 4.20 – 4.14 (m, 2H), 4.01 (d, J = 8.9 Hz, 1H), 2.86 – 2.78 (m, 1H), 2.51 (dt, J_1 = 12.9 Hz, J_2 = 7.8 Hz, 1H).

General procedure C: Synthesis of amine analogues 62, 63, 64, 65, 66, 67, 68, 69, 70, and 71 (62 is given as an example). 1-(4-Methoxyphenyl)cyclopentane-1-carbonitrile (319 mg, 1.59 mmol, 1.0 equiv) was dissolved in anhydrous THF (20 mL) under argon atmosphere while ice cooling. $LiAlH_4$ (181 mg, 4.77 mmol, 3.0 equiv) was added in portions on ice bath and the batch was stirred at room temperature overnight. For workup, diethylether (100 mL) was added to reaction mixture with ice cooling and then aqueous brine solution (5-10 mL) was slowly added while the batch was stirred on ice bath. Residual water was removed by addition of Na_2SO_4. Precipitate was filtered off and additionally washed with diethylether (3 x 100 mL). Organic solvent was removed under reduced pressure and the product was used without further purification unless stated otherwise.

(1-(4-Methoxyphenyl)cyclopentyl)methanamine (62)

Synthesized from 1-(4-methoxyphenyl)cyclopentane-1-carbonitrile (319 mg, 1,58 mmol, 1.0 equiv) and $LiAlH_4$ (180 mg, 4.75 mmol, 3.0 equiv) according to general procedure C. Yield: 78.6 %; oil (257 mg), ^{1}H NMR (400 MHz, DMSO) δ 7.19 – 7.12 (m, 2H), 6.88 – 6.81 (m, 2H), 3.72 (s, 3H), 2.53 (s, 2H), 1.94 – 1.86 (m, 2H), 1.74 – 1.53 (m, 6H), 0.92 (brs, 2H).

(1-(2-Methoxyphenyl)cyclopentyl)methanamine (63)

Synthesized from 1-(2-methoxyphenyl)cyclopentane-1-carbonitrile (3.28 g, 16.30 mmol, 1.0 equiv) and $LiAlH_4$ (1.86 g, 48.89 mmol, 3.0 equiv) according to general procedure C. Yield: 79.8 %; oil (2.67 g), ^{1}H NMR (400 MHz, $CDCl_3$) δ 7.25 – 7.16 (m, 2H), 6.94 – 6.83 (m, 2H), 3.80 (s, 3H), 2.84 (s, 2H), 2.08 – 1.98 (m, 2H), 1.87 – 1.82 (m, 2H), 1.69 – 1.62 (m, 4H), 1.26 (brs, 2H).

(1-(3,4-Dimethoxyphenyl)cyclopentyl)methanamine (64)

Synthesized from 1-(3,4-dimethoxyphenyl)cyclopentane-1-carbonitrile (291 mg, 1.26 mmol, 1.0 equiv) and $LiAlH_4$ (143 mg, 3.77 mmol, 3.0 equiv) according to general procedure C. Yield: 82.8 %; oil (236 mg); ^{1}H NMR (400 MHz, DMSO) δ 6.86 (d, J = 8.3 Hz, 1H), 6.80 (d, J = 2.1 Hz, 1H), 6.75 (dd, J_1 = 8.3 Hz, J_2 = 2.1 Hz, 1H), 3.74 (s, 3H), 3.72 (s, 3H), 2.55 (s, 2H), 1.94 – 1.89 (m, 2H), 1.77 – 1.68 (m, 2H), 1.67 – 1.54 (m, 4H), 0.93 (brs, 2H).

(1-(Benzo[*d*][1,3]dioxol-5-yl)cyclopentyl)methanamine (65)

Synthesized from 1-(benzo[*d*][1,3]dioxol-5-yl)cyclopentane-1-carbonitrile (197 mg, 0.92 mmol, 1.0 equiv) and $LiAlH_4$ (104 mg, 2.75 mmol, 3.0 equiv) according to general procedure C. Yield: 75.9 %; oil (152 mg); ^{1}H NMR (400 MHz, DMSO) δ 6.84 – 6.79 (m, 2H), 6.69 (dd, J_1 = 8.1 Hz, J_2 = 1.8 Hz, 1H), 5.96 (s, 2H), 2.52 (s, 2H), 1.94 – 1.84 (m, 2H), 1.71 – 1.54 (m, 6H), 0.95 (brs, 2H).

(1-Phenylcyclopentyl)methanamine (66)

Synthesized from 1-phenylcyclopentane-1-carbonitrile (293 mg, 1.71 mmol, 1.0 equiv) and $LiAlH_4$ (195 mg, 5.13 mmol, 3.0 equiv) according to general procedure C. Yield: 85.9 %; oil (257 mg); ^{1}H NMR (400 MHz, $CDCl_3$) δ 7.34 – 7.25 (m, 4H), 7.22 – 7.16 (m, 1H), 2.73 (s, 2H), 1.95 – 1.85 (m, 4H), 1.75 – 1.66 (m, 4H), 1.26 (brs, 2H).

(1-(3-Methoxyphenyl)cyclopentyl)methanamine (67)

Synthesized from 1-(3-methoxyphenyl)cyclopentane-1-carbonitrile (496 mg, 2.46 mmol, 1.0 equiv) and $LiAlH_4$ (281 mg, 7.40 mmol, 3.0 equiv) according to general procedure C. Yield: 81.8 %; oil (414 mg); ^{1}H NMR (400 MHz, DMSO) δ 7.21 (t, J = 7.9 Hz, 1H), 6.83 (ddd, J_1 = 7.7 Hz, J_2 = 1.6 Hz, J_3 = 0.9 Hz, 1H), 6.79 – 6.72 (m, 2H), 3.74 (s, 3H), 2.56 (s, 2H), 1.96 – 1.89 (m, 2H), 1.76 – 1.55 (m, 6H), 0.97 (brs, 2H).

(1-(*m*-Tolyl)cyclopentyl)methanamine (68)

Synthesized from 1-(*m*-tolyl)cyclopentane-1-carbonitrile (285 mg, 1.54 mmol, 1.0 equiv) and $LiAlH_4$ (175 mg, 4.61 mmol, 3.0 equiv) according to general procedure C. Yield: 92.8 %; oil (270 mg); ^{1}H NMR (400 MHz, DMSO) δ 7.17 (t, J = 7.6 Hz, 1H), 7.09 – 7.01 (m, 2H), 6.98 (d, J = 7.4 Hz, 1H), 2.56 (s, 2H), 2.29 (s, 3H), 1.99 – 1.87 (m, 2H), 1.77 – 1.54 (m, 6H), 0.93 (brs, 2H).

(1-(Thiophen-2-yl)cyclopentyl)methanamine (69)

Synthesized from 1-(thiophen-2-yl)cyclopentane-1-carbonitrile (266 mg, 1.5 mmol, 1.0 equiv) and $LiAlH_4$ (171 mg, 4.5 mmol, 3.0 equiv) according to general procedure C. Yield: 79.8 %; oil (217 mg); ^{1}H NMR (400 MHz, $CDCl_3$) δ 7.16 (dd, J_1 = 5.1 Hz, J_2 = 1.2 Hz, 1H), 6.94 (dd, J_1 = 5.1 Hz, J_2 = 3.5 Hz, 1H), 6.84 (dd, J_1 = 3.5 Hz, J_2 = 1.2 Hz, 1H), 2.80 (s, 2H), 2.03 – 1.94 (m, 2H), 1.86 – 1.78 (m, 2H), 1.75 – 1.67 (m, 4H), 1.12 (brs, 2H).

(1-(Thiophen-3-yl)cyclopentyl)methanamine (70)

Synthesized from 1-(thiophen-3-yl)cyclopentane-1-carbonitrile (382 mg, 2.15 mmol, 1.0 equiv) and $LiAlH_4$ (245 mg, 6.46 mmol, 3.0 equiv) according to general procedure C. Yield: 78.8 %; oil (308 mg); ^{1}H NMR (400 MHz, DMSO) δ 7.44 (dd, J_1 = 5.0 Hz, J_2 = 2.9 Hz, 1H), 7.15 (dd, J_1 = 2.9 Hz, J_2 = 1.4 Hz, 1H), 7.04 (dd, J_1 = 5.0 Hz, J_2 = 1.4 Hz, 1H), 2.59 (s, 2H), 1.85 – 1.71 (m, 4H), 1.68 – 1.52 (m, 4H), 1.03 (brs, 2H).

(3-(Thiophen-2-yl)tetrahydrofuran-3-yl)methanamine (71)

Synthesized from 3-(thiophen-2-yl)tetrahydrofuran-3-carbonitrile (225 mg, 1.26 mmol, 1.0 equiv) and $LiAlH_4$ (143 mg, 3.77 mmol, 3.0 equiv) according to general procedure C. Yield: 94.5 %; oil (222 mg); ^{1}H NMR (400 MHz, $CDCl_3$) δ 7.21 (dd, J_1 = 5.1 Hz, J_2 = 1.2 Hz, 1H), 6.98 (dd, J_1 = 5.1 Hz, J_2 = 3.5 Hz, 1H), 6.88 (dd, J_1 = 3.5 Hz, J_2 = 1.2 Hz, 1H), 4.05 – 3.87 (m, 4H), 2.96 (s, 2H), 2.30 – 2.16 (m, 2H), 1.38 (brs, 2H).

Cyclohexane ring containing compounds 15, 16, 17, 18, 19, 20, and 21 were synthesized according to General procedure A (17 is given as an example).

2-Methoxybenzoic acid (1.08 g, 7.10 mmol, 1.0 equiv) was dissolved in dichloromethane (50 mL) with ice cooling. Oxalyl chloride (1.86 mL, 21.3 mmol, 3.0 equiv) was added dropwise followed by 5 drops of DMF. The batch was stirred at

room temperature overnight and next day the solvent was evaporated. (1-(4-(Trifluoromethyl)phenyl)cyclohexyl)methanamine (1.83 g, 7.10 mmol, 1.0 equiv) and Et_3N (2.95 mL, 21.3 mmol, 3.0 equiv) were dissolved in dichloromethane (50 mL) with ice cooling and then 2-methoxybenzoyl chloride intermediate (7.10 mmol), dissolved in dichloromethane (20 mL), was added dropwise. Reaction mixture was stirred at room temperature overnight. Next day organic phase was diluted with dichloromethane (100 mL) and washed with saturated aqueous $NaHCO_3$ solution (2 x 50 mL), 1M aqueous HCl solution, water (2 x 50 mL), saturated brine solution (50 mL), dried over Na_2SO_4, and remained organic phase was removed under reduced pressure. Product was purified with column chromatography using ethyl acetate:*n*-hexane = 1:4 as mobile phase and additionally using Biotage Isolera One System reversed-phase chromatography (Biotage SNAP Cartridge KP-C18-HS 12 g column), MF: gradient water in H_2O/acetonitril.

***N*-((1-(3,4-Dimethoxyphenyl)cyclohexyl)methyl)-2-methoxybenzamide (15)**
Synthesized from (1-(3,4-dimethoxyphenyl)cyclohexyl)methanamine (106 mg, 0.43 mmol, 1.0 equiv), Et_3N (0.18 mL, 1.28 mmol, 3.0 equiv) and 2-methoxybenzoyl chloride intermediate (0.43 mmol) according to general procedure A. Column chromatography, EtOAc/*n*-hex = 1/4 (v/v). Yield: 67.4 %; %; white solid (110 mg); ^{1}H NMR (400 MHz, $CDCl_3$) δ 8.20 (dd, J_1 = 7.8 Hz, J_2 = 1.8 Hz, 1H), 7.55 (brs, 1H), 7.38 (ddd, J_1 = 8.4 Hz, J_2 = 7.4 Hz, J_3 = 1.9 Hz, 1H), 7.03 (t, *J* = 7.5 Hz, 1H), 6.99 – 6.89 (m, 3H), 6.86 (d, *J* = 8.2 Hz, 1H), 3.90 (s, 3H), 3.87 (s, 3H), 3.65 (d, *J* = 5.8 Hz, 2H), 3.62 (s, 3H), 2.06 – 2.00 (m, 2H), 1.78 – 1.70 (m, 2H), 1.68 – 1.60 (m, 2H), 1.49 – 1.40 (m, 4H); ^{13}C NMR (101 MHz, $CDCl_3$) δ 165.17, 157.45, 148.92, 147.28, 138.03, 132.60, 132.44, 121.44, 121.22, 119.32, 111.20, 111.13, 110.60, 56.00 (2 x C-O), 55.42, 50.50, 41.93, 34.30, 26.41, 22.20 ppm; HRMS (ESI$^+$) for $C_{23}H_{30}NO_4$ ([M+H]$^+$) calculated 384.2169, found 384.2165; HPLC retention time: 5.653 min (98.35 % at 254 nm).

2-Methoxy-*N*-((1-(4-methoxyphenyl)cyclohexyl)methyl)benzamide (16)
Synthesized from (1-(4-methoxyphenyl)cyclohexyl)methanamine (178 mg, 0.81 mmol, 1.0 equiv), Et_3N (0.34 mL, 2.43 mmol, 3.0 equiv) and 2-methoxybenzoyl chloride intermediate (0.81 mmol) according to general procedure A. Column chromatography, EtOAc/*n*-hex = 1/4 (v/v). Yield: 71.3 %; %; white solid (205 mg); ^{1}H NMR (400 MHz, $CDCl_3$) δ 8.19 (dd, J_1 = 7.8 Hz, J_2 = 1.9 Hz, 1H), 7.54 (brs, 1H), 7.40 – 7.31 (m, 3H), 7.02 (td, J_1 = 7.9 Hz, J_2 = 1.0 Hz, 1H), 6.96 – 6.91 (m, 2H), 6.85 (d, *J* = 7.8 Hz, 1H), 3.82 (s, 3H), 3.64 (d, *J* = 5.5 Hz, 2H), 3.63 (s, 3H), 2.07 – 1.98 (m, 2H), 1.76 – 1.67 (m, 2H), 1.67 – 1.57 (m, 2H), 1.48 – 1.36 (m, 4H); ^{13}C NMR (101 MHz, $CDCl_3$) δ 165.18, 157.75, 157.45, 137.27, 132.55, 132.41, 128.10, 121.47, 121.16, 113.89, 111.11, 55.49, 55.33, 50.61, 41.56, 34.25, 26.47, 22.16 ppm; HRMS (ESI$^+$) for $C_{22}H_{28}NO3$ ([M+H]$^+$) calculated 354.2064, found 354.2058; HPLC retention time: 6.187 min (96.58 % at 254 nm).

2-Methoxy-*N*-((1-(4-(trifluoromethyl)phenyl)cyclohexyl)methyl)benzamide (17)
Synthesized from (1-(4-(trifluoromethyl)phenyl)cyclohexyl)methanamine (1.83 g, 7.1 mmol, 1.0 equiv), Et_3N (2.97 mL, 2.13 mmol, 3.0 equiv) and 2-methoxybenzoyl chloride intermediate (7.1 mmol) according to general procedure A. Column chromatography, EtOAc/*n*-hex = 1/4 (v/v). Yield: 56.0 %; white solid (1.56 g); ^{1}H NMR (400 MHz, $CDCl_3$) δ 8.20 (dd, J_1 = 7.8 Hz, J_2 = 1.8 Hz, 1H), 7.66 (d, *J* = 8.3 Hz, 2H), 7.56 (d, *J* = 8.3 Hz, 2H), 7.48 (brs, 1H), 7.40 (ddd, J_1 = 8.4 Hz, J_2 = 7.4 Hz, J_3 = 1.9 Hz, 1H), 7.08 – 7.00 (m, 1H), 6.86 (d, *J* = 8.2 Hz, 1H), 3.69 (d, *J* = 6.1 Hz, 2H), 3.57 (s, 3H), 2.14 – 2.05 (m, 2H), 1.83 – 1.74 (m, 2H), 1.72 – 1.62 (m, 2H), 1.51 – 1.43 (m, 2H), 1.43 – 1.35 (m, 2H), ^{13}C NMR (101 MHz, $CDCl_3$) δ 165.25, 157.42, 149.94, 132.80, 132.53, 128.40 (q, *J* = 32.4 Hz), 127.68, 125.44 (q, *J* = 3.7 Hz), 124.38 (q, *J* = 271.8 Hz), 121.33, 121.19, 111.17, 55.38, 50.27, 43.01, 34.01, 26.32, 22.14 ppm; HRMS (ESI$^+$) for $C_{22}H_{25}F_3NO_2$ ([M+H]$^+$) calculated 392.1832, found 392.1824; HPLC retention time: 6.903 min (95.84 % at 254 nm).

2-Methoxy-*N*-((1-(2-methoxyphenyl)cyclohexyl)methyl)benzamide (18)
Synthesized from (1-(2-methoxyphenyl)cyclohexyl)methanamine (525 mg, 2.39 mmol, 1.0 equiv), Et_3N (1.0 mL, 7.18 mmol, 3.0 equiv) and 2-methoxybenzoyl chloride intermediate according to general procedure A. Column chromatography, EtOAc/*n*-hex = 1/4 (v/v). Yield: 72.4 %; white solid (613 mg); ^{1}H NMR (400 MHz, $CDCl_3$) δ 8.18 (dd, J_1 = 7.8 Hz, J_2 = 1.9 Hz, 1H), 7.42 (brs, 1H), 7.36 – 7.31 (m, 2H), 7.26 (ddd, J_1 = 9.0 Hz, J_2 = 7.6 Hz, J_3 = 1.7 Hz, 1H), 6.99 (td, J_1 = 7.8 Hz, J_2 = 1.1 Hz, 2H), 6.93 (dd, J_1 = 8.2 Hz, J_2 = 1.1 Hz, 1H), 6.79 (dd, J_1 = 8.3 Hz, J_2 = 0.6 Hz, 1H), 3.99 (d, *J* = 5.6 Hz, 2H), 3.82 (s, 3H), 3.48 (s, 3H), 2.15 – 2.07 (m, 2H), 1.94 – 1.86 (m, 2H), 1.70 – 1.62 (m, 2H), 1.52 – 1.38 (m, 4H); ^{13}C NMR (101 MHz, $CDCl_3$) δ 165.10, 158.91, 157.47, 132.90, 132.35, 132.31, 129.73, 127.77, 121.64, 121.05, 120.44, 111.93, 111.08, 55.31, 55.19, 45.27, 42.62, 33.72, 26.62, 22.47 ppm; HRMS (ESI$^+$) for $C_{22}H_{28}NO3$ ([M+H]$^+$) calculated 354.2064, found 354.2054; HPLC retention time: 6.493 min (99.64 % at 254 nm).

2-Methoxy-*N*-((1-(3-methoxyphenyl)cyclohexyl)methyl)benzamide (19).

Synthesized from (1-(3-methoxyphenyl)cyclohexyl)methanamine (314 mg, 1.43 mmol, 1.0 equiv), Et_3N (0.60 mL, 4.29 mmol, 3.0 equiv) and 2-methoxybenzoyl chloride intermediate (1.43 mmol) according to general procedure A. Column chromatography, EtOAc/*n*-hex = 1/4 (v/v). Yield: 76.8 %; white solid (389 mg); ^{1}H NMR (400 MHz, $CDCl_3$) δ 8.20 (dd, J_1 = 7.8 Hz, J_2 = 1.9 Hz, 1H), 7.56 (brs, 1H), 7.38 (ddd, J_1 = 8.3 Hz, J_2 = 7.3 Hz, J_3 = 1.9 Hz, 1H), 7.33 (t, J = 8.0 Hz, 1H), 7.06 – 7.00 (m, 2H), 6.99 – 6.97 (m, 1H), 6.88 – 6.83 (m, 1H), 6.81 (ddd, J_1 = 8.2 Hz, J_2 = 2.5 Hz, J_2 = 0.6 Hz, 1H), 3.82 (s, 3H), 3.66 (d, J = 5.8 Hz, 2H), 3.63 (s, 3H), 2.12 – 2.00 (m, 2H), 1.78 – 1.69 (m, 2H), 1.68 – 1.58 (m, 2H), 1.50 – 1.36 (m, 4H); ^{13}C NMR (101 MHz, $CDCl_3$) δ 165.26, 159.92, 157.56, 147.35, 132.61, 132.50, 129.58, 121.47, 121.20, 119.50, 113.85, 111.14, 110.63, 55.39, 55.26, 50.56, 42.31, 34.20, 26.46, 22.26 ppm; HRMS (ESI$^+$) for $C_{22}H_{28}NO_3$ ([M+H]$^+$) calculated 354.2064, found 354.2049; HPLC retention time: 6.217 min (96.11 % at 254 nm).

2-Methoxy-*N*-((1-(*m*-tolyl)cyclohexyl)methyl)benzamide (20)

Synthesized from (1-(*m*-tolyl)cyclohexyl)methanamine (258 mg, 1.27 mmol, 1.0 equiv), Et_3N (0.53 mL, 3.81 mmol, 3.0 equiv) and 2-methoxybenzoyl chloride intermediate (1.27 mmol) according to general procedure A. Column chromatography, EtOAc/*n*-hex = 1/4 (v/v). Yield: 70.6 %; white solid (302 mg); ^{1}H NMR (400 MHz, $CDCl_3$) δ 8.20 (dd, J_1 = 7.8 Hz, J_2 = 1.9 Hz, 1H), 7.54 (brs, 1H), 7.37 (ddd, J_1 = 8.4 Hz, J_2 = 7.3 Hz, J_3 = 1.9 Hz, 1H), 7.31 – 7.26 (m, 1H), 7.22 (d, J = 6.2 Hz, 2H), 7.07 (d, J = 7.2 Hz, 1H), 7.04 – 6.99 (m, 1H), 6.84 (d, J = 7.9 Hz, 1H), 3.67 (d, J = 5.8 Hz, 2H), 3.58 (s, 3H), 2.38 (s, 3H), 2.10 – 2.00 (m, 2H), 1.78 – 1.68 (m, 2H), 1.68 – 1.57 (m, 2H), 1.52 – 1.36 (m, 4H); ^{13}C NMR (101 MHz, $CDCl_3$) δ 165.18, 157.48, 145.45, 137.96, 132.54, 132.42, 128.43, 127.75, 126.80, 124.12, 121.47, 121.16, 111.11, 55.33, 50.36, 42.04, 34.16, 26.46, 22.23, 21.89 ppm; HRMS (ESI$^+$) for $C_{22}H_{28}NO_2$ ([M+H]$^+$) calculated 338.2115, found 338.2109; HPLC retention time: 6.680 min (97.60 % at 254 nm).

2-Methoxy-*N*-((1-(thiophen-3-yl)cyclohexyl)methyl)benzamide (21)

Synthesized from (1-(thiophen-3-yl)cyclohexyl)methanamine (276 mg, 1.41 mmol, 1.0 equiv), Et_3N (0.59 mL, 4.23 mmol, 3.0 equiv) and 2-methoxybenzoyl chloride intermediate (1.41 mmol) according to general procedure A. Column chromatography, EtOAc/*n*-hex = 1/4 (v/v). Yield: 69.7 %; white solid (321 mg); ^{1}H NMR (400 MHz, $CDCl_3$) δ 8.21 (dd, J_1 = 7.8 Hz, J_2 = 1.9 Hz, 1H), 7.62 (brs, 1H), 7.42 – 7.35 (m, 2H), 7.12 – 7.07 (m, 2H), 7.03 (td, J_1 = 7.8 Hz, J_2 = 1.0 Hz, 1H), 6.88 (d, J = 8.3 Hz, 1H), 3.71 (s, 3H), 3.65 (d, J = 5.9 Hz, 2H), 2.02 – 1.93 (m, 2H), 1.76 – 1.69 (m, 2H), 1.66 – 1.60 (m, 2H), 1.50 – 1.40 (m, 4H); ^{13}C NMR (101 MHz, $CDCl_3$) δ 165.19, 157.52, 147.77, 132.62, 132.47, 126.57, 125.66, 121.43, 121.20, 120.82, 111.17, 55.59, 49.86, 41.31, 34.83, 26.34, 22.16 ppm; HRMS (ESI$^+$) for $C_{19}H_{24}NO_2S$ ([M+H]$^+$) calculated 330.1522, found 330.1515; HPLC retention time: 6.160 min (98.72 % at 254 nm).

General procedure D. Synthesis of cyclohexane analogues 72, 73, 74, 75, 76, 77 and 78 (with 74 as an example).

2-(4-(Trifluoromethyl)phenyl)acetonitrile (5.3 g, 28.7 mmol, 1.0 equiv) was slowly added to a stirred solution of NaH (60 % dispersion in mineral oil) (2.3 g, 57.4 mmol, 2.0 equiv) in anhydrous DMF (50 mL) while ice cooling under argon atmosphere. The batch was stirred for 15 minutes and then solution 1,5-dibropentane (4.7 mL, 34.4 mmol, 1.2 equiv) in anhydrous DMF (30 mL) was added dropwise while ice cooling. The batch was stirred at room temperature overnight. Next day water (50 mL) was added to the reaction mixture and the reaction mixture was washed with diethylether (3 x 100 mL). Combined organic phases were washed with saturated brine solution (2 x 100 mL), dried over Na_2SO_4, filtered and then solvent was removed under reduced pressure. Product was purified with column chromatography using ethyl acetate:*n*-hexane = 1:6 as mobile phase. 2.9 g (11.3 mmol) of yellow oil was isolated.

1-(3,4-Dimethoxyphenyl)cyclohexane-1-carbonitrile (72)

Synthesized from 2-(3,4-dimethoxyphenyl)acetonitrile (246 mg, 1.39 mmol, 1.0 equiv), NaH (111 mg, 2.78 mmol, 2.0 equiv) and 1,5-dibropentane (0.23 mL, 1.67 mmol, 1.2 equiv) according to general procedure D. Column chromatography, EtOAc/*n*-hex = 1/6 (v/v). Yield: 48.8 %; oil (166 mg); ^{1}H NMR (400 MHz, DMSO) δ 7.07 – 7.02 (m, 2H), 6.98 (d, J = 8.8 Hz, 1H), 3.78 (s, 3H), 3.75 (s, 3H), 2.06 (d, J = 12.1 Hz, 2H), 1.88 – 1.76 (m, 4H), 1.74 (d, J = 13.3 Hz, 1H), 1.68 – 1.56 (m, 2H), 1.36 – 1.24 (m, 1H).

1-(4-Methoxyphenyl)cyclohexane-1-carbonitrile (73)

Synthesized from 2-(4-methoxyphenyl)acetonitrile (262 mg, 1.78 mmol, 1.0 equiv), NaH (142 mg, 3.56 mmol, 2.0 equiv) and 1,5-dibropentane (0.29 mL, 2.14 mmol, 1.2 equiv) according to general procedure D. Column chromatography,

EtOAc/*n*-hex = 1/6 (v/v). Yield: 49.3 %; oil (189 mg); ^{1}H NMR (400 MHz, DMSO) δ 7.46 – 7.40 (m, 2H), 7.01 – 6.95 (m, 2H), 3.76 (s, 3H), 2.04 (d, *J* = 13.9 Hz, 2H), 1.86 – 1.69 (m, 5H), 1.67 – 1.55 (m, 2H), 1.34 – 1.22 (m, 1H).

1-(4-(Trifluoromethyl)phenyl)cyclohexane-1-carbonitrile (74)

Synthesized from 2-(4-(trifluoromethyl)phenyl)acetonitrile (5.39 g, 29.14 mmol, 1.0 equiv), NaH (2.33 g, 58.28 mmol, 2.0 equiv) and 1,5-dibropentane (4.76 mL, 34.97 mmol, 1.2 equiv) according to general procedure D. Column chromatography, EtOAc/*n*-hex = 1/6 (v/v). Yield: 39.3 %; oil (2.9 g); ^{1}H NMR (400 MHz, DMSO) δ 7.84 – 7.76 (m, 4H), 2.08 (d, *J* = 12.5 Hz, 2H), 1.97 – 1.81 (m, 4H), 1.80 – 1.72 (m, 1H), 1.71 – 1.56 (m, 2H), 1.41 – 1.24 (m, 1H).

1-(2-Methoxyphenyl)cyclohexane-1-carbonitrile (75)

Synthesized from 2-(2-methoxyphenyl)acetonitrile (882 mg, 6.0 mmol, 1.0 equiv), NaH (480 mg, 12.0 mmol, 2.0 equiv) and 1,5-dibropentane (0.98 mL, 7.2 mmol, 1.2 equiv) according to general procedure D. Column chromatography, EtOAc/*n*-hex = 1/6 (v/v). Yield: 48.3 %; oil (623 mg); ^{1}H NMR (400 MHz, DMSO) δ 7.35 (dtd, J_1 = 9.6 Hz, J_2 = 7.7 Hz, J_3 = 1.6 Hz, 2H), 7.12 (dd, J_1 = 8.2 Hz, J_2 = 1.0 Hz, 1H), 7.00 (td, J_1 = 7.7 Hz, J_2 = 1.2 Hz, 1H), 3.86 (s, 3H), 2.27 (d, *J* = 10.9 Hz, 2H), 1.84 – 1.60 (m, 7H), 1.32 – 1.16 (m, 1H).

1-(3-Methoxyphenyl)cyclohexane-1-carbonitrile (76)

Synthesized from 2-(3-methoxyphenyl)acetonitrile (571 mg, 3.88 mmol, 1.0 equiv), NaH (310 mg, 7.76 mmol, 2.0 equiv) and 1,5-dibropentane (0.63 mL, 4.66 mmol, 1.2 equiv) according to general procedure D. Column chromatography, EtOAc/*n*-hex = 1/6 (v/v). Yield: 58.8 %; oil (491 mg); ^{1}H NMR (400 MHz, DMSO) δ 7.35 (t, *J* = 8.0 Hz, 1H), 7.10 (ddd, J_1 = 7.8 Hz, J_2 = 1.8 Hz, J_3 = 0.8 Hz, 1H), 7.07 – 7.01 (m, 1H), 6.92 (ddd, J_1 = 8.2 Hz, J_2 = 2.5 Hz, J_3 = 0.8 Hz, 1H), 3.78 (s, 3H), 2.04 (d, *J* = 12.5 Hz, 2H), 1.88 – 1.77 (m, 4H), 1.77 – 1.69 (m, 1H), 1.69 – 1.55 (m, 2H), 1.37 – 1.23 (m, 1H).

1-(*m*-Tolyl)cyclohexane-1-carbonitrile (77)

Synthesized from 2-(*m*-tolyl)acetonitrile (432 mg, 3.29 mmol, 1.0 equiv), NaH (263 mg, 6.58 mmol, 2.0 equiv) and 1,5-dibropentane (0.54 mL, 3.95 mmol, 1.2 equiv) according to general procedure D. Column chromatography, EtOAc/*n*-hex = 1/6 (v/v). Yield: 59.9 %; oil (393 mg); ^{1}H NMR (400 MHz, DMSO) δ 7.34 (d, *J* = 0.8 Hz, 1H), 7.32 – 7.28 (m, 2H), 7.18 – 7.13 (m, 1H), 2.34 (s, 3H), 2.03 (d, *J* = 13.0 Hz, 2H), 1.87 – 1.78 (m, 4H), 1.78 – 1.69 (m, 1H), 1.69 – 1.55 (m, 2H), 1.36 – 1.23 (m, 1H).

1-(Thiophen-3-yl)cyclohexane-1-carbonitrile (78)

Synthesized from 2-(thiophen-3-yl)acetonitrile (355 mg, 2.89 mmol, 1.0 equiv), NaH (231 mg, 5.78 mmol, 2.0 equiv) and 1,5-dibropentane (0.47 mL, 3.47 mmol, 1.2 equiv) according to general procedure D. Column chromatography, EtOAc/*n*-hex = 1/6 (v/v). Yield: 76.8 %; oil (424 mg); ^{1}H NMR (400 MHz, DMSO) δ 7.61 (dd, J_1 = 5.1 Hz, J_2 = 3.0 Hz, 1H), 7.53 (dd, J_1 = 2.9 Hz, J_2 = 1.4 Hz, 1H), 7.27 (dd, J_1 = 5.1 Hz, J_2 = 1.4 Hz, 1H), 2.16 (d, *J* = 11.5 Hz, 2H), 1.84 – 1.70 (m, 5H), 1.66 – 1.52 (m, 2H), 1.33 – 1.22 (m, 1H).

Compounds 79, 80, 81, 82, 83, 84, and 85 were synthesized according to general procedure C (81 is given as an example).

1-(4-(Trifluoromethyl)phenyl)cyclohexane-1-carbonitrile (2.9 g, 11.3 mmol, 1.0 equiv) was dissolved in anhydrous THF (30 mL) under argon atmosphere with ice cooling. $LiAlH_4$ (1.30 g, 34.35 mmol, 3.0 equiv) was added in portions on ice bath and the batch was stirred at room temperature overnight. For workup, diethylether (100 mL) was added to reaction mixture with ice cooling and then aqueous brine solution (5-10 mL) was slowly added while the batch was stirred on ice bath. Residual water was removed by addition of Na_2SO_4. Precipitate was filtered off and additionally washed with diethylether (3 x 100 mL). Organic solvent was removed under reduced pressure and the product was used without further purification unless stated otherwise. 1.83 g (7.1 mmol) of yellow oil was obtained. Yield: 62.8 %

(1-(3,4-Dimethoxyphenyl)cyclohexyl)methanamine (79)

Synthesized from 1-(3,4-dimethoxyphenyl)cyclohexane-1-carbonitrile (166 mg, 0.68 mmol, 1.0 equiv) and $LiAlH_4$ (77 mg, 2.0 mmol, 3.0 equiv) according to general procedure C. Yield: 62.8 %; oil (106 mg); ^{1}H NMR (400 MHz, DMSO) δ 6.90 (d, *J* = 8.4 Hz, 1H), 6.87 – 6.79 (m, 2H), 3.74 (s, 3H), 3.73 (s, 3H), 3.38 (s, 2H), 2.00 (d, *J* = 9.4 Hz, 2H), 1.55 – 1.38 (m, 4H), 1.35 – 1.11 (m, 4H), 0.84 (brs, 2H).

(1-(4-Methoxyphenyl)cyclohexyl)methanamine (80)

Synthesized from 1-(4-methoxyphenyl)cyclohexane-1-carbonitrile (189 mg, 0.88 mmol, 1.0 equiv) and $LiAlH_4$ (100 mg, 2.63 mmol, 3.0 equiv) according to general procedure C. Yield: 92.8 %; oil (178 mg); ^{1}H NMR (400 MHz, DMSO) δ 7.26 – 7.19 (m, 2H), 6.91 – 6.85 (m, 2H), 3.73 (s, 3H), 2.47 (s, 2H), 2.01 (d, *J* = 13.1 Hz, 2H), 1.52 – 1.40 (m, 5H), 1.32 – 1.20 (m, 3H), 0.85 (brs, 2H).

(1-(4-(Trifluoromethyl)phenyl)cyclohexyl)methanamine (81)

Synthesized from 1-(4-(trifluoromethyl)phenyl)cyclohexane-1-carbonitrile (2.9 g, 11.45 mmol, 1.0 equiv) and $LiAlH_4$ (1.30 g, 34.35 mmol, 3.0 equiv) according to general procedure C. Yield: 62.8 %; oil (1.83 g); ^{1}H NMR (400 MHz, $CDCl_3$) δ 7.60 (d, *J* = 8.2 Hz, 2H), 7.46 (d, *J* = 8.2 Hz, 2H), 2.72 (s, 2H), 2.20 – 2.10 (m, 2H), 1.88 – 1.82 (m, 4H), 1.60 – 1.52 (m, 4H).

(1-(2-Methoxyphenyl)cyclohexyl)methanamine (82)

Synthesized from 1-(2-methoxyphenyl)cyclohexane-1-carbonitrile (623 mg, 2.89 mmol, 1.0 equiv) and $LiAlH_4$ (329 mg, 8.68 mmol, 3.0 equiv) according to general procedure C. Yield: 82.8 %; oil (525 mg); ^{1}H NMR (400 MHz, $CDCl_3$) δ 7.27 – 7.16 (m, 2H), 6.97 – 6.84 (m, 2H), 3.79 (s, 3H), 2.95 (s, 2H), 2.28 (dd, J_1 = 12.7 Hz, J_2 = 6.8 Hz, 2H), 1.65 – 1.51 (m, 4H), 1.50 – 1.35 (m, 4H), 1.11 (brs, 2H).

(1-(3-Methoxyphenyl)cyclohexyl)methanamine (83)

Synthesized from 1-(3-methoxyphenyl)cyclohexane-1-carbonitrile (491 mg, 2.28 mmol, 1.0 equiv) and $LiAlH_4$ (260 mg, 6.84 mmol, 3.0 equiv) according to general procedure C. Yield: 62.8 %; oil (314 mg); ^{1}H NMR (400 MHz, DMSO) δ 7.25 (t, *J* = 8.0 Hz, 1H), 6.92 – 6.87 (m, 1H), 6.85 – 6.82 (m, 1H), 6.76 (ddd, J_1 = 8.1 Hz, J_2 = 2.5 Hz, J_3 = 0.6 Hz, 1H), 3.74 (s, 3H), 2.51 (s, 2H), 2.02 (d, *J* = 10.5 Hz, 2H), 1.56 – 1.43 (m, 5H), 1.33 – 1.20 (m, 3H), 0.92 (brs, 2H).

(1-(*m*-Tolyl)cyclohexyl)methanamine (84)

Synthesized from 1-(*m*-tolyl)cyclohexane-1-carbonitrile (393 mg, 1.97 mmol, 1.0 equiv) and $LiAlH_4$ (225 mg, 5.92 mmol, 3.0 equiv) according to general procedure C. Yield: 64.3 %; oil (258 mg); ^{1}H NMR (400 MHz, DMSO) δ 7.21 (t, *J* = 7.6 Hz, 1H), 7.15 – 7.09 (m, 2H), 6.98 (d, *J* = 7.4 Hz, 1H), 2.51 (s, 2H), 2.30 (s, 3H), 2.04 (d, *J* = 12.3 Hz, 2H), 1.54 – 1.41 (m, 5H), 1.35 – 1.17 (m, 3H), 0.87 (brs, 2H).

(1-(Thiophen-3-yl)cyclohexyl)methanamine (85)

Synthesized from 1-(thiophen-3-yl)cyclohexane-1-carbonitrile (424 mg, 2.22 mmol, 1.0 equiv) and $LiAlH_4$ (252 mg, 6.65 mmol, 3.0 equiv) according to general procedure C. Yield: 63.8 %; oil (276 mg); ^{1}H NMR (400 MHz, DMSO) δ 7.46 (dd, J_1 = 5.0 Hz, J_2 = 2.9 Hz, 1H), 7.15 (dd, J_1 = 2.9 Hz, J_2 = 1.4 Hz, 1H), 7.06 (dd, J_1 = 5.0 Hz, J_2 = 1.4 Hz, 1H), 2.51 (s, 2H), 1.94 (d, *J* = 8.0 Hz, 2H), 1.55 – 1.41 (m, 5H), 1.35 – 1.21 (m, 3H), 0.90 (brs, 2H).

Compounds 22, 23, 24, 25, 26, 27, 28 and 29 were synthesized according to general procedure A (29 is given as an example).

2-Methoxybenzoic acid (806 mg, 5.3 mmol, 1.0 equiv) was dissolved in dichloromethane (50 mL) with ice cooling. Oxalyl chloride (1.4 mL, 15.9 mmol, 1.0 equiv) was added dropwise followed by 5 drops of DMF. The batch was stirred at room temperature overnight and next day the solvent was evaporated. (4-(*m*-Tolyl)tetrahydro-2*H*-pyran-4-yl)methanamine (1.09 g, 5.3 mmol, 1.0 equiv) and Et_3N (2.2 mL, 15.9 mmol, 3.0 equiv) were dissolved in dichloromethane (50 mL) with ice cooling and then 2-methoxybenzoyl chloride intermediate (5.3 mmol), dissolved in dichloromethane (20 mL). Reaction mixture was stirred at room temperature overnight. Organic phase was then diluted with dichloromethane (30 mL) and washed with saturated aqueous $NaHCO_3$ solution (2x50 mL), 1M aqueous HCl solution, water (2x50mL), saturated brine solution (50 mL), dried over Na_2SO_4, and remained organic phase was removed under reduced pressure. Product was purified with column chromatography using ethyl acetate:*n*-hexane = 1:1 as mobile phase and additionally using Biotage Isolera One System reversed-phase chromatography (Biotage SNAP Cartridge KP-C18-HS 12 g column), MF: gradient water in H_2O/acetonitril.

2-Methoxy-*N*-((4-(4-(trifluoromethyl)phenyl)tetrahydro-2*H*-pyran-4-yl)methyl)benzamide (22)

Synthesized from (4-(4-(trifluoromethyl)phenyl)tetrahydro-2*H*-pyran-4-yl)methanamine (275 mg, 1.06 mmol, 1.0 equiv), Et_3N (0.44 mL, 3.18 mmol, 3.0 equiv) and 2-methoxybenzoyl chloride (1.06 mmol) according to general procedure A. Column chromatography, EtOAc/*n*-hex = 1/1 (v/v) and Biotage Isolera One System reversed-phase chromatography (Biotage SNAP Cartridge KP-C18-HS 12 g column), MF: gradient water in H_2O/acetonitril. Yield: 23.5 %; white solid (98 mg); ^1H NMR (400 MHz, DMSO-d_6) δ 7.77 (d, *J* = 8.3 Hz, 2H), 7.72 (dd, J_1 = 7.8 Hz, J_2 = 1.8 Hz, 1H), 7.73 – 7.61 (m, 3H), 7.47 – 7.40 (m, 1H), 7.07 (d, *J* = 8.0 Hz, 1H), 7.03 – 6.96 (m, 1H), 3.83 – 3.71 (m, 2H), 3.65 (s, 3H), 3.62 (d, *J* = 6.2 Hz, 2H), 3.51 – 3.40 (m, 2H), 2.11 – 2.00 (m, 2H), 1.97 – 1.83 (m, 2H); ^{13}C NMR (101 MHz, $CDCl_3$) δ 165.38, 157.38, 149.29, 133.00, 132.46, 128.94 (q, *J* = 32.6 Hz), 127.33, 125.65 (q, *J* = 3.7 Hz), 124.20 (q, *J* = 271.8 Hz), 121.37, 120.85, 111.18, 64.07, 55.37, 48.47, 40.94, 33.73 ppm; HRMS (ESI$^+$) for $C_{21}H_{23}F_3NO_3$ ($[M+H]^+$) calculated 394.1625, found 394.1624; HPLC retention time: 4.047 min (97.2 % at 254 nm).

_N_-((4-(3,4-Dimethoxyphenyl)tetrahydro-2_H_-pyran-4-yl)methyl)-2-methoxybenzamide (23)

Synthesized from (4-(3,4-dimethoxyphenyl)tetrahydro-2*H*-pyran-4-yl)methanamine (313 mg, 1.25 mmol, 1.0 equiv), Et_3N (0.52 mL, 3.75 mmol, 3.0 equiv) and 2-methoxybenzoyl chloride (1.25 mmol) according to general procedure A. Column chromatography, EtOAc/*n*-hex = 1/1 (v/v). Yield: 28.1 %; white solid (135 mg); ^1H NMR (400 MHz, $CDCl_3$) δ 8.19 (dd, J_1 = 7.8 Hz, J_2 = 1.8 Hz, 1H), 7.59 (t, *J* = 5.3 Hz, 1H), 7.40 (ddd, J_1 = 8.4 Hz, J_2 = 7.4 Hz, J_3 = 1.9 Hz, 1H), 7.09 – 7.01 (m, 1H), 6.93 (s, 2H), 6.90 – 6.84 (m, 2H), 3.91 (s, 3H), 3.90 – 3.84 (m, 2H), 3.87 (s, 3H), 3.77 (d, *J* = 6.0 Hz, 2H), 3.71 – 3.64 (m, 2H), 3.62 (s, 3H), 2.13 – 2.05 (m, 2H), 1.99 – 1.90 (m, 2H); NMR (101 MHz, $CDCl_3$) δ 165.32, 157.44, 149.12, 147.72, 137.19, 132.83, 132.45, 121.32, 121.17, 119.10, 111.33, 111.17, 110.24, 64.21, 56.06 (2 × C-O), 55.45, 49.10, 40.02, 34.12 ppm; HRMS (ESI$^+$) for $C_{22}H_{28}NO_5$ ($[M+H]^+$) calculated 386.1962, found 386.1954; HPLC retention time: 4.007 min (97.49 % at 254 nm).

2-Methoxy-_N_-((4-(4-methoxyphenyl)tetrahydro-2_H_-pyran-4-yl)methyl)benzamide (24)

Synthesized from (4-(4-methoxyphenyl)tetrahydro-2*H*-pyran-4-yl)methanamine (444 mg, 2.0 mmol, 1.0 equiv), Et_3N (0.84 mL, 6.0 mmol, 3.0 equiv) and 2-methoxybenzoyl chloride (2.0 mmol) according to general procedure A. Column chromatography, EtOAc/*n*-hex = 1/1 (v/v). Yield: 29.3 %; white solid (209 mg); ^1H NMR (400 MHz, $CDCl_3$) δ 8.18 (dd, J_1 = 7.8 Hz, J_2 = 1.9 Hz, 1H), 7.57 (brs, 1H), 7.39 (ddd, J_1 = 8.3 Hz, J_2 = 7.3 Hz, J_3 = 1.9 Hz, 1H), 7.32 – 7.27 (m, 2H), 7.04 (td, J_1 = 7.9 Hz, J_2 = 1.0 Hz, 1H), 6.99 – 6.94 (m, 2H), 6.87 (dd, J_1 = 8.3 Hz, J_2 = 0.6 Hz, 1H), 3.90 – 3.84 (m, 2H), 3.84 (s, 3H), 3.75 (d, *J* = 6.0 Hz, 2H), 3.68 – 3.60 (m, 2H), 3,63 (s, 3H), 2.13 – 2.05 (m, 2H), 1.98 – 1.90 (m, 2H); ^{13}C NMR (101 MHz, $CDCl_3$) δ 165.36, 158.17, 157.47, 136.44, 132.79, 132.46, 127.91, 121.30, 121.24, 114.15, 111.16, 64.22, 55.53, 55.43, 49.32, 39.71, 34.12 ppm; HRMS (ESI$^+$) for $C_{21}H_{26}NO_4$ ($[M+H]^+$) calculated 356.1856, found 356.1849; HPLC retention time: 4.507 min (98.39 % at 254 nm).

2-Methoxy-_N_-((4-(_p_-tolyl)tetrahydro-2_H_-pyran-4-yl)methyl)benzamide (25)

Synthesized from (4-(*p*-tolyl)tetrahydro-2H-pyran-4-yl)methanamine (444 mg, 2.16 mmol, 1.0 equiv), Et_3N (0.90 mL, 6.49 mmol, 3.0 equiv) and 2-methoxybenzoyl chloride (2.16 mmol) according to general procedure A. Column chromatography, EtOAc/*n*-hex = 1/1 (v/v). Yield: 24.4 %; white solid (179 mg); ^1H NMR (400 MHz, $CDCl_3$) δ 8.18 (d, *J* = 7.8 Hz, 1H), 7.56 (brs, 1H), 7.39 (t, *J* = 7.8 Hz, 1H), 7.25 (q, *J* = 8.2 Hz, 4H), 7.03 (t, *J* = 7.5 Hz, 1H), 6.86 (d, *J* = 8.3 Hz, 1H), 3.91 – 3.82 (m, 2H), 3.76 (d, *J* = 5.9 Hz, 2H), 3.68 – 3.61 (m, 2H), 3.60 (s, 3H), 2.38 (s, 3H), 2.16 – 2.06 (m, 2H), 1.99 – 1.89 (m, 2H); ^{13}C NMR (101 MHz, $CDCl_3$) δ 165.36, 157.46, 141.47, 136.11, 132.77, 132.45, 129.53, 126.73, 121.27, 121.24, 111.14, 64.25, 55.42, 49.27, 39.96, 34.01, 20.99 ppm; HRMS (ESI$^+$) for $C_{21}H_{26}NO_3$ ($[M+H]^+$) calculated 340.1907, found 340.1901; HPLC retention time: 5.040 min (100 % at 254 nm).

_N_-((4-(Benzo[_d_][1,3]dioxol-5-yl)tetrahydro-2_H_-pyran-4-yl)methyl)-2-methoxybenzamide (26)

Synthesized from (4-(benzo[*d*][1,3]dioxol-5-yl)tetrahydro-2*H*-pyran-4-yl)methanamine (301 mg, 1.28 mmol, 1.0 equiv), Et_3N (0.53 mL, 3.84 mmol, 3.0 equiv) and 2-methoxybenzoyl chloride (1.28 mmol) according to general procedure A. Column chromatography, EtOAc/*n*-hex = 1/1 (v/v). Yield: 44.4 %; white solid (210 mg); ^1H NMR (400 MHz, $CDCl_3$) δ 8.18 (dd, J_1 = 7.8 Hz, J_2 = 1.8 Hz, 1H), 7.60 (t, *J* = 5.2 Hz, 1H), 7.40 (ddd, J_1 = 8.4 Hz, 7.3, J_2 = 1.9 Hz, 1H), 7.05 (td, J_1 = 7.9 Hz, J_2 = 1.0 Hz, 1H), 6.92 – 6.80 (m, 4H), 5.98 (s, 2H), 3.90 – 3.81 (m, 2H), 3.73 (d, *J* = 6.0 Hz, 2H), 3.71 (s, 3H), 3.69 – 3.61 (m, 2H), 2.10 – 2.00 (m, 2H), 1.96 – 1.89 (m, 2H); ^{13}C NMR (101 MHz, $CDCl_3$) δ 165.36, 157.46, 148.25, 146.11, 138.69, 132.82, 132.46, 121.30, 121.20, 119.93, 111.17, 108.34, 107.36, 101.20, 64.16, 55.53, 49.19, 40.15, 34.21 ppm; HRMS (ESI$^+$) for $C_{21}H_{24}NO_5$ ($[M+H]^+$) calculated 370.1649, found 370.1642; HPLC retention time: 4.410 min (98.72 % at 254 nm).

2-Methoxy-*N*-((4-(2-methoxyphenyl)tetrahydro-2*H*-pyran-4-yl)methyl)benzamide (27).

Synthesized from (4-(2-methoxyphenyl)tetrahydro-2H-pyran-4-yl)methanamine (880 mg, 3.98 mmol, 1.0 equiv), Et_3N (1.66 mL, 11.93 mmol, 3.0 equiv) and 2-methoxybenzoyl chloride (3.98 mmol) according to general procedure A. Column chromatography, EtOAc/*n*-hex = 1/1 (v/v). Yield: 40.3 %; white solid (570 mg); ^{1}H NMR (400 MHz, $CDCl_3$) δ 8.17 (dd, J_1 = 7.8 Hz, J_2 = 1.9 Hz, 1H), 7.43 (brs, 1H), 7.36 (ddd, J_1 = 8.3 Hz, J_2 = 7.3 Hz, J_3 = 1.9 Hz, 1H), 7.31 (ddd, J_1 = 8.1 Hz, J_2 = 7.4 Hz, J_3 = 1.7 Hz, 1H), 7.25 (dd, J_1 = 7.9 Hz, J_2 = 1.7 Hz, 1H), 7.01 (tdd, J_1 = 7.6 Hz, J_2 = 3.3 Hz, J_3 = 1.1 Hz, 2H), 6.97 (dd, J_1 = 8.2 Hz, J_2 = 1.1 Hz, 1H), 6.81 (dd, J_1 = 8.3 Hz, J_2 = 0.7 Hz, 1H), 4.10 (d, J = 5.8 Hz, 2H), 3.93 – 3.86 (m, 2H), 3.84 (s, 3H), 3.74 – 3.67 (m, 2H), 3.49 (s, 3H), 2.30 – 2.21 (m, 2H), 2.13 – 2.04 (m, 2H); ^{13}C NMR (101 MHz, $CDCl_3$) δ 165.28, 158.70, 157.47, 132.60, 132.36, 131.71, 129.37, 128.25, 121.40, 121.19, 120.60, 112.00, 111.12, 64.40, 55.37, 55.19, 44.12, 40.67, 33.63 ppm; HRMS (ESI$^+$) for $C_{21}H_{26}NO_4$ ([M+H]$^+$) calculated 356.1856, found 356.1849; HPLC retention time: 4.777 min (99.29 % at 254 nm).

2-Methoxy-*N*-((4-(3-methoxyphenyl)tetrahydro-2*H*-pyran-4-yl)methyl)benzamide (28)

Synthesized from (4-(3-methoxyphenyl)tetrahydro-2H-pyran-4-yl)methanamine (918 mg, 4.14 mmol, 1.0 equiv), Et_3N (1.73 mL, 12.42 mmol, 3.0 equiv) and 2-methoxybenzoyl chloride (4.15 mmol) according to general procedure A. Column chromatography, EtOAc/*n*-hex = 1/1 (v/v). Yield: 41.5 %; white solid (612 mg); ^{1}H NMR (400 MHz, DMSO) δ 7.84 (dd, J_1 = 7.7 Hz, J_2 = 1.8 Hz, 1H), 7.65 (t, J = 5.8 Hz, 1H), 7.45 (ddd, J_1 = 8.4 Hz, J_2 = 7.3 Hz, J_3 = 1.9 Hz, 1H), 7.36 (t, J = 8.0 Hz, 1H), 7.11 – 7.06 (m, 1H), 7.05 – 6.98 (m, 2H), 6.96 – 6.93 (m, 1H), 6.89 (dd, J_1 = 8.0 Hz, J_2 = 2.2 Hz, 1H), 3.77 (s, 3H), 3.76 – 3.70 (m, 2H), 3.67 (s, 3H), 3.60 (d, J = 5.9 Hz, 2H), 3.51 – 3.42 (m, 2H), 2.06 – 1.98 (m, 2H), 1.89 – 1.80 (m, 2H); ^{13}C NMR (101 MHz, $CDCl_3$) δ 165.38, 160.02, 157.52, 146.51, 132.81, 132.47, 129.85, 121.26, 121.17, 119.12, 113.68, 111.16, 111.02, 64.25, 55.39, 55.30, 49.07, 40.35, 34.00 ppm; HRMS (ESI$^+$) for $C_{21}H_{26}NO_4$ ([M+H]$^+$) calculated 356.1856, found 356.1849; HPLC retention time: 4.570 min (100 % at 254 nm).

2-Methoxy-*N*-((4-(*m*-tolyl)tetrahydro-2*H*-pyran-4-yl)methyl)benzamide (29)

Synthesized from (4-(*m*-tolyl)tetrahydro-2*H*-pyran-4-yl)methanamine (1.09 g, 5.31 mmol, 1.0 equiv), Et_3N (2.22 mL, 15.93 mmol, 3.0 equiv) and 2-methoxybenzoyl chloride (5.31 mmol) according to general procedure A. Column chromatography, EtOAc/*n*-hex = 1/1 (v/v) and Biotage Isolera One System reversed-phase chromatography (Biotage SNAP Cartridge KP-C18-HS 12 g column), MF: gradient water in H_2O/acetonitril. Yield: 41.4 %; white solid (745 mg); ^{1}H NMR (400 MHz, $CDCl_3$) δ 8.19 (dd, J_1 = 7.8 Hz, J_2 = 1.8 Hz, 1H), 7.57 (brs, 1H), 7.38 (ddd, J_1 = 8.4 Hz, J_2 = 7.4 Hz, J_3 = 1.9 Hz, 1H), 7.34 – 7.28 (m, 1H), 7.20 – 7.15 (m, 2H), 7.12 (d, J = 7.5 Hz, 1H), 7.06 – 6.99 (m, 1H), 6.85 (d, J = 8.1 Hz, 1H), 3.92 – 3.83 (m, 2H), 3.79 (d, J = 6.0 Hz, 2H), 3.70 – 3.61 (m, 2H), 3.58 (s, 3H), 2.39 (s, 3H), 2.16 – 2.07 (m, 2H), 2.00 – 1.89 (m, 2H); ^{13}C NMR (101 MHz, $CDCl_3$) δ 165.27, 157.43, 144.57, 138.27, 132.73, 132.36, 128.62, 127.43, 127.31, 123.83, 121.19, 121.14, 111.12, 64.21, 55.31, 48.97, 40.10, 33.94, 21.84 ppm; HRMS (ESI$^+$) for $C_{21}H_{26}NO_3$ ([M+H]$^+$) calculated 340.1907, found 340.1903; HPLC retention time: 4.990 min (98.16 % at 254 nm).

Compounds 30, 31, 32, 33, 34, 35, 36 and 37 were synthesized according to general procedure A using various acid derivatives (listed below) as precursors

2,6-Dimethoxy-*N*-((4-phenyltetrahydro-2*H*-pyran-4-yl)methyl)benzamide (30).

2,6-Dimethoxybenzoic acid (273 mg, 1.5 mmol, 1.0 equiv) was dissolved in dichloromethane (30 mL) with ice cooling. Oxalyl chloride (0.4 mL, 4.5 mmol, 3.0 equiv) was added dropwise followed by 5 drops of DMF. The batch was stirred at room temperature overnight and next day the solvent was evaporated. (4-Phenyltetrahydro-2H-pyran-4-yl)methanamine (287 mg, 1.5 mmol, 1.0 equiv) and Et_3N (0.63 mL, 4.5 mmol, 30 equiv) were dissolved in dichloromethane (30 mL) with ice cooling and then 2,6-dimethoxybenzoyl chloride intermediate (1.5 mmol), dissolved in dichloromethane (20 mL), was added ropwise. Reaction mixture was stirred at room temperature overnight. Next day organic phase was diluted with dichloromethane (30 mL) and washed with saturated aqueous $NaHCO_3$ solution (2 x 50 mL), 1M aqueous HCl solution, water (2 x 50mL), saturated brine solution (50 mL), dried over Na_2SO_4, and organic phase was removed under reduced pressure. Product was purified with column chromatography using ethyl acetate:*n*-hexane = 1:2 as mobile phase and additionally using Biotage Isolera One System reversed-phase chromatography (Biotage SNAP Cartridge KP-C18-HS 12 g column), MF: gradient water in H_2O/acetonitril. Yield: 30.1 %; white solid (160 mg); ^{1}H NMR (400 MHz, $CDCl_3$) δ 7.38 – 7.32 (m, 4H), 7.26 – 7.18 (m, 2H), 6.51 (d, J = 8.4 Hz, 2H), 5.34 (t, J = 6.0 Hz, 1H), 3.96 – 3.89 (m, 2H), 3.79 (s, 6H), 3.73 (d, J = 6.5 Hz, 2H), 3.71 – 3.63 (m, 2H), 2.18 – 2.03 (m, 4H); ^{13}C NMR (101 MHz, $CDCl_3$) δ 165.90, 157.30,

144.60, 130.63, 128.80, 126.71, 126.63, 116.09, 104.00, 64.30, 55.91, 48.48, 40.65, 33.62 ppm; HRMS (ESI^+) for $C_{21}H_{26}NO_4$ ($[M+H]^+$) calculated 356.1856, found 356.1852; HPLC retention time: 4.037 min (99.50 % at 254 nm).

N-((4-Phenyltetrahydro-2H-pyran-4-yl)methyl)thiophene-3-carboxamide (31)

Thiophene-3-carboxylic acid (192 mg, 1.5 mmol, 1.0 equiv) was dissolved in dichloromethane (30 mL) with ice cooling. Oxalyl chloride (0.4 mL, 4.5 mmol, 3.0 equiv) was added dropwise followed by 5 drops of DMF. The batch was stirred at room temperature overnight and next day the solvent was evaporated. (4-Phenyltetrahydro-2H-pyran-4-yl)methanamine (287 mg, 1.5 mmol, 1.0 equiv) and Et_3N (0.63 mL, 4.5 mmol, 3.0 equiv) were dissolved in dichloromethane (30 mL) with ice cooling and then thiophene-3-carbonyl chloride (1.5 mmol), dissolved in dichloromethane (20 mL), was added dropwise. Reaction mixture was stirred at room temperature overnight. Next day organic phase was diluted with dichloromethane (30 mL) and washed with saturated aqueous $NaHCO_3$ solution (2 x 50 mL), 1M aqueous HCl solution, water (2 x 50mL), saturated brine solution (50 mL), dried over Na_2SO_4, and remained organic phase was removed under reduced pressure. Product was purified with column chromatography using ethyl acetate:*n*-hexane = 1:2 as mobile phase. Yield: 31.1 %; white solid (141 mg); ^1H NMR (400 MHz, $CDCl_3$) δ 7.66 (dd, J_1 = 3.0 Hz, J_2 = 1.3 Hz, 1H), 7.47 – 7.41 (m, 2H), 7.38 – 7.30 (m, 3H), 7.29 – 7.26 (m, 1H), 7.14 (dd, J_1 = 5.1 Hz, J_2 = 1.3 Hz, 1H), 5.49 (brs, 1H), 3.91 – 3.83 (m, 2H), 3.66 (d, J = 6.3 Hz, 2H), 3.66 – 3.60 (m, 2H), 2.18 – 2.09 (m, 2H), 2.01 – 1.92 (m, 2H); ^{13}C NMR (101 MHz, $CDCl_3$) δ 163.02, 143.87, 137.43, 129.20, 128.15, 127.09, 126.73, 126.63, 125.81, 64.19, 48.81, 40.81, 33.76 ppm; HRMS (ESI^+) for $C_{17}H_{20}NO_2S$ ($[M+H]^+$) calculated 302.1209, found 302.1205; HPLC retention time: 3.827 min (99.59 % at 254 nm).

N-((4-Phenyltetrahydro-2H-pyran-4-yl)methyl)furan-3-carboxamide (32).
Furan-3-carboxylic acid (170 mg, 1.5 mmol, 1.0 equic) was dissolved in dichloromethane (30 mL) with ice cooling. Oxalyl chloride (0.4 mL, 4.5 mmol, 3.0 equiv) was added dropwise followed by 5 drops of DMF. The batch was stirred at room temperature overnight and next day the solvent was evaporated. (4-Phenyltetrahydro-2*H*-pyran-4-yl)methanamine (287 mg, 1.5 mmol, 1.0 equiv) and Et_3N (0.63 mL, 4.5 mmol, 3.0 equiv) were dissolved in dichloromethane (30 mL) with ice cooling and then furan-3-carbonyl chloride (1.5 mmol), dissolved in dichloromethane (20 mL), was added dropwise. Reaction mixture was stirred at room temperature overnight. Next day organic phase was diluted with dichloromethane (30 mL) and washed with saturated aqueous $NaHCO_3$ solution (2 x 50 mL), 1M aqueous HCl solution (1 x 50 mL), water (2 x 50mL), saturated brine solution (50 mL), dried over Na_2SO_4, and organic phase was removed under reduced pressure. Product was purified with column chromatography using ethyl acetate:*n*-hexane = 1:2 as mobile phase. Yield: 29.3 %; white solid (125 mg); ^1H NMR (400 MHz, $CDCl_3$) δ 7.77 (s, 1H), 7.48 – 7.40 (m, 2H), 7.39 – 7.28 (m, 4H), 6.36 (d, J = 1.0 Hz, 1H), 5.28 (brs, 1H), 3.91 – 3.81 (m, 2H), 3.70 – 3.58 (m, 2H), 3.63 (d, J = 6.4 Hz, 2H), 2.20 – 2.08 (m, 2H), 2.01 – 1.89 (m, 2H); ^{13}C NMR (101 MHz, $CDCl_3$) δ 162.58, 144.67, 143.93, 143.80, 129.19, 127.10, 126.63, 122.51, 108.07, 64.18, 48.54, 40.79, 33.73 ppm; HRMS (ESI^+) for $C_{17}H_{20}NO_3$ ($[M+H]^+$) calculated 286.1438, found 286.1434; HPLC retention time: 3.467 min (98.29 % at 254 nm).

2-(2-Methoxyphenyl)-N-((4-phenyltetrahydro-2H-pyran-4-yl)methyl)acetamide (33)

2-(2-Methoxyphenyl)acetic acid (250 mg, 1.5 mmol, 1.0 equiv) was dissolved in dichloromethane (30 mL) with ice cooling. Oxalyl chloride (0.4 mL, 4.5 mmol, 3.0 equiv) was added dropwise followed by 5 drops of DMF. The batch was stirred at room temperature overnight and next day the solvent was evaporated. (4-Phenyltetrahydro-2*H*-pyran-4-yl)methanamine (287 mg, 1.5 mmol, 1.0 equiv) and Et_3N (0.63 mL, 4.5 mmol, 3.0 equiv) were dissolved in dichloromethane (30 mL) with ice cooling and then 2-(2-methoxyphenyl)acetyl chloride (1.5 mmol), dissolved in dichloromethane (20 mL), was added dropwise. Reaction mixture was stirred at room temperature overnight. Next day organic phase was diluted with dichloromethane (30 mL) and washed with saturated aqueous $NaHCO_3$ solution (2 x 50 mL), 1M aqueous HCl solution (1 x 50 mL), water (2 x 50mL), saturated brine solution (50 mL), dried over Na_2SO_4, and organic phase was removed under reduced pressure. Product was purified with column chromatography using ethyl acetate:*n*-hexane = 1:2 as mobile phase. Yield: 29.8 %; white solid (152 mg); ^1H NMR (400 MHz, $CDCl_3$) δ 7.30 – 7.17 (m, 4H), 7.12 (dd, J_1 = 7.4 Hz, J_2 = 1.7 Hz, 1H), 7.04 – 6.98 (m, 2H), 6.90 (td, J_1 = 7.4 Hz, J_2 = 1.0 Hz, 1H), 6.83 (d, J = 8.2 Hz, 1H), 5.35 (brs, 1H), 3.82 – 3.74 (m, 2H), 3.64 (s, 3H), 3.58 – 3.50 (m, 2H), 3.47 (s, 2H), 3.42 (d, J = 6.3 Hz, 2H), 1.97 – 1.89 (m, 2H), 1.81 – 1.72 (m, 2H); ^{13}C NMR (101 MHz, $CDCl_3$) δ 171.45, 156.94, 143.87, 131.23, 128.89 (3 x Ar -$\underline{C}$), 126.44, 126.29 (2 x Ar-$\underline{C}$), 123.71, 121.20, 110.74, 64.11, 55.25, 48.40, 40.24, 38.87, 33.51 ppm; HRMS (ESI^+) for $C_{21}H_{26}NO_3$ ($[M+H]^+$) calculated 340.1907, found 340.1903; HPLC retention time: 4.440 min (99.11 % at 254 nm).

2-Methoxy-5-methyl-N-((4-phenyltetrahydro-2H-pyran-4-yl)methyl)benzamide (34)

2-Methoxy-5-methylbenzoic acid (250 mg, 1.25 mmol, 1.0 equiv) was dissolved in dichloromethane (30 mL) with ice cooling. Oxalyl chloride (0.4 mL, 4.5 mmol, 3.0 equiv) was added dropwise followed by 5 drops of DMF. The batch was stirred at room temperature overnight and next day the solvent was evaporated. (4-Phenyltetrahydro-2*H*-pyran-4-yl)methanamine (287 mg, 1.5 mmol, 1.0 equiv) and Et_3N (0.63 mL, 4.5 mmol, 3.0 equiv) were dissolved in dichloromethane (30 mL) with ice cooling and then 2-methoxy-5-methylbenzoyl chloride (1.5 mmol), dissolved in dichloromethane (20 mL), was added dropwise. Reaction mixture was stirred at room temperature overnight. Next day organic phase was diluted with dichloromethane (30 mL) and washed with saturated aqueous $NaHCO_3$ solution (2 x 50 mL), 1M aqueous HCl solution (1 x 50 mL), water (2 x 50mL), saturated brine solution (50 mL), dried over Na_2SO_4, and organic phase was removed under reduced pressure. Product was purified with column chromatography using ethyl acetate:*n*-hexane = 1:2 as mobile phase. Yield: 30.2 %; white solid (154 mg); ^{1}H NMR (400 MHz, $CDCl_3$) δ 7.99 (d, *J* = 2.1 Hz, 1H), 7.61 (t, *J* = 5.1 Hz, 1H), 7.46 – 7.35 (m, 4H), 7.33 – 7.27 (m, 1H), 7.18 (ddd, J_1 = 8.4 Hz, J_1 = 2.4 Hz, J_1 = 0.6 Hz, 1H), 6.75 (d, *J* = 8.4 Hz, 1H), 3.93 – 3.84 (m, 2H), 3.79 (d, *J* = 6.1 Hz, 2H), 3.70 – 3.62 (m, 2H), 3.54 (s, 3H), 2.29 (s, 3H), 2.16 – 2.07 (m, 2H), 2.01 – 1.92 (m, 2H); ^{13}C NMR (101 MHz, $CDCl_3$) δ 165.54, 155.48, 144.74, 133.21, 132.71, 130.61, 128.84, 126.81, 126.59, 120.63, 111.15, 64.24, 55.52, 48.93, 40.35, 33.92, 20.39 ppm; HRMS (ESI$^+$) for $C_{21}H_{26}NO_3$ ([M+H]$^+$) calculated 340.1907, found 340.1901; HPLC retention time: 5.027 min (99.70 % at 254 nm).

2-Ethoxy-*N*-((4-phenyltetrahydro-2*H*-pyran-4-yl)methyl)benzamide (35)

2-Ethoxybenzoic acid (0.23 mL, 1.5 mmol, 1.0 equiv) was dissolved in dichloromethane (30 mL) with ice cooling. Oxalyl chloride (0.4 mL, 4.5 mmol, 3.0 equiv) was added dropwise followed by 5 drops of DMF. The batch was stirred at room temperature overnight and next day the solvent was evaporated. (4-Phenyltetrahydro-2*H*-pyran-4-yl)methanamine (287 mg, 1.5 mmol, 1.0 equiv) and Et_3N (0.63 mL, 4.5 mmol, 3.0 equiv) were dissolved in dichloromethane (30 mL) with ice cooling and then 2-ethoxybenzoyl chloride (1.5 mmol), dissolved in dichloromethane (20 mL), was added dropwise. Reaction mixture was stirred at room temperature overnight. Next day organic phase was diluted with dichloromethane (30 mL) and washed with saturated aqueous $NaHCO_3$ solution (2 x 50 mL), 1M aqueous HCl solution, water (2 x 50mL), saturated brine solution (50 mL), dried over Na_2SO_4, and remained organic phase was removed under reduced pressure. Product was purified with column chromatography using ethyl acetate:*n*-hexane = 1:2 as mobile phase. Yield: 29.5 %; white solid (150 mg); ^{1}H NMR (400 MHz, $CDCl_3$) δ 8.21 (dd, J_1 = 7.8 Hz, J_2 = 1.8 Hz, 1H), 7.78 (brs, 1H), 7.43 – 7.33 (m, 5H), 7.30 – 7.23 (m, 1H), 7.06 – 6.98 (m, 1H), 6.85 (d, *J* = 8.3 Hz, 1H), 3.97 – 3.92 (m, 2H), 3.92 – 3.85 (m, 2H), 3.81 (d, *J* = 6.3 Hz, 2H), 3.69 – 3.60 (m, 2H), 2.16 – 2.06 (m, 2H), 2.02 – 1.92 (m, 2H), 1.01 (t, *J* = 7.0 Hz, 3H); ^{13}C NMR (101 MHz, $CDCl_3$) δ 165.57, 156.79, 144.64, 132.73, 132.51, 128.83, 126.73, 126.59, 121.21, 121.07, 112.15, 64.32, 64.20, 48.84, 40.57, 33.92, 14.24 ppm; HRMS (ESI$^+$) for $C_{21}H_{26}NO_3$ ([M+H]$^+$) calculated 340.1907, found 340.1903; HPLC retention time: 5.003 min (98.52 % at 254 nm).

2-Methoxy-4-methyl-*N*-((4-phenyltetrahydro-2*H*-pyran-4-yl)methyl)benzamide (36)

2-Methoxy-4-methylbenzoic acid (250 mg, 1.5 mmol, 1.0 equiv) was dissolved in dichloromethane (30 mL) with ice cooling. Oxalyl chloride (0.4 mL, 4.5 mmol, 3.0 equiv) was added dropwise followed by 5 drops of DMF. The batch was stirred at room temperature overnight and next day the solvent was evaporated. (4-Phenyltetrahydro-2*H*-pyran-4-yl)methanamine (287 mg, 1.5 mmol, 1.0 equiv) and Et_3N (0.63 mL, 4.5 mmol, 3.0 equiv) were dissolved in dichloromethane (30 mL) with ice cooling and then 2-methoxy-4-methylbenzoyl chloride (1.5 mmol), dissolved in dichloromethane (20 mL), was added dropwise. Reaction mixture was stirred at room temperature overnight. Next day organic phase was diluted with dichloromethane (30 mL) and washed with saturated aqueous $NaHCO_3$ solution (2 x 50 mL), 1M aqueous HCl solution (1 x 50 mL), water (2 x 50mL), saturated brine solution (50 mL), dried over Na_2SO_4, and organic phase was removed under reduced pressure. Product was purified with column chromatography using ethyl acetate:*n*-hexane = 1:2 as mobile phase. Yield: 29.8 %; white solid (152 mg); ^{1}H NMR (400 MHz, $CDCl_3$) δ 8.07 (d, *J* = 8.0 Hz, 1H), 7.54 (t, *J* = 5.5 Hz, 1H), 7.46 – 7.35 (m, 4H), 7.33 – 7.28 (m, 1H), 6.84 (dd, J_1 = 8.0 Hz, J_2 = 0.7 Hz, 1H), 6.65 (s, 1H), 3.93 – 3.84 (m, 2H), 3.79 (d, *J* = 6.1 Hz, 2H), 3.69 – 3.61 (m, 2H), 3.56 (s, 3H), 2.34 (s, 3H), 2.16 – 2.07 (m, 2H), 2.00 – 1.92 (m, 2H); ^{13}C NMR (101 MHz, $CDCl_3$) δ 165.43, 157.40, 144.75, 143.62, 132.39, 128.82, 126.81, 126.57, 122.06, 118.42, 111.87, 64.24, 55.35, 48.90, 40.34, 33.93, 21.78 ppm; HRMS (ESI$^+$) for $C_{21}H_{26}NO_3$ ([M+H]$^+$) calculated 340.1907, found 340.1902; HPLC retention time: 4.977 min (100 % at 254 nm).

5-Fluoro-2-methoxy-*N*-((4-phenyltetrahydro-2*H*-pyran-4-yl)methyl)benzamide (37)

5-Fluoro-2-methoxybenzoic acid (260 mg, 1.5 mmol, 1.0 equiv) was dissolved in dichloromethane (30 mL) with ice cooling. Oxalyl chloride (0.4 mL, 4.5 mmol, 3.0 equiv) was added dropwise followed by 5 drops of DMF. The batch was

stirred at room temperature overnight and next day the solvent was evaporated. (4-Phenyltetrahydro-2*H*-pyran-4-yl)methanamine (287 mg, 1.5 mmol, 1.0 equiv) and Et_3N (0.63 mL, 4.5 mmol, 3.0 equiv) were dissolved in dichloromethane (30 mL) with ice cooling and then 5-fluoro-2-methoxybenzoyl chloride (1.5 mmol), dissolved in dichloromethane (20 mL), was added dropwise. Reaction mixture was stirred at room temperature overnight. Next day organic phase was diluted with dichloromethane (30 mL) and washed with saturated aqueous $NaHCO_3$ solution (2 x 50 mL), 1M aqueous HCl solution (1 x 50 mL), water (2 x 50 mL), saturated brine solution (50 mL), dried over Na_2SO_4, and organic phase was removed under reduced pressure. Product was purified with column chromatography using ethyl acetate:*n*-hexane = 1:2 as mobile phase. Yield: 30.2 %; white solid (156 mg); ^{1}H NMR (400 MHz, $CDCl_3$) δ 7.89 (dd, J_1 = 9.5 Hz, J_2 = 3.3 Hz, 1H), 7.60 (brs, 1H), 7.47 – 7.41 (m, 2H), 7.40 – 7.35 (m, 2H), 7.35 – 7.28 (m, 1H), 7.08 (ddd, J_1 = 9.0 Hz, J_2 = 7.2 Hz, J_3 = 3.3 Hz, 1H), 6.80 (dd, J_1 = 9.1 Hz, J_2 = 4.1 Hz, 1H), 3.92 – 3.84 (m, 2H), 3.79 (d, *J* = 6.0 Hz, 2H), 3.70 – 3.61 (m, 2H), 3.56 (s, 3H), 2.18 – 2.08 (m, 2H), 2.01 – 1.91 (m, 2H); ^{13}C NMR (101 MHz, $CDCl_3$) δ 164.17(d, *J* = 1.8 Hz), 157.17 (d, *J* = 239.8 Hz), 153.61 (d, *J* = 2.1 Hz), 144.52, 128.92, 126.83, 126.71, 122.64 (d, *J* = 6.7 Hz), 119.02 (d, *J* = 31.1 Hz), 118.78 (d, *J* = 32.7 Hz), 112.49 (d, *J* = 7.6 Hz), 64.22, 56.07, 49.21, 40.32, 33.97 ppm; HRMS (ESI$^+$) for $C_{20}H_{23}FNO_3$ ($[M+H]^+$) calculated 344.1657, found 344.1653; HPLC retention time: 4.813 min (97.05 % at 254 nm).

General procedure E: Synthesis of tetrahydro-2*H*-pyran analogues 86, 87, 88, 89, 90, 91, 92, 93 and 94 (94 is given as an example).

2-(*m*-Tolyl)acetonitrile (1 g, 7.6 mmol, 1.0 equiv) was slowly added to a stirred solution of NaH (60 % dispersion in mineral oil) (610 mg, 15.2 mmol, 2.0 equiv) in anhydrous DMF (30 mL) while ice cooling under argon atmosphere. The batch was stirred for 15 minutes on ice bath and then solution of 1-chloro-2-(2-chloroethoxy)ethane (0.89 mL, 7.6 mmol, 1.0 equiv) in anhydrous DMF (20 mL) was added dropwise while ice cooling. The batch was stirred at room temperature overnight. Next day water (50 mL) was added to the reaction mixture and the reaction mixture was washed with diethylether (3 x 100 mL). Combined organic phases were washed with saturated brine solution (2 x 100 mL), dried over Na_2SO_4, filtered and then solvent was removed under reduced pressure. Product was purified with column chromatography using ethyl acetate:*n*-hexane = 1:5 as mobile phase.

4-Phenyltetrahydro-2*H*-pyran-4-carbonitrile (86)

Synthesized from 2-phenylacetonitrile (3.82 g, 32.62 mmol, 1.0 equiv), NaH (2.61 g, 65.24 mmol, 2.0 equiv), and 1-chloro-2-(2-chloroethoxy)ethane (3.82 mL, 32.62 mmol, 1.0 equiv) according to general procedure E. Column chromatography, EtOAc/*n*-hex = 1/5 (v/v). Yield: 83.5 %; oil (5.1 g); ^{1}H NMR (400 MHz, $CDCl_3$) δ 7.53 – 7.46 (m, 2H), 7.46 – 7.39 (m, 2H), 7.38 – 7.31 (m, 1H), 4.08 (ddd, J_1 = 6.9 Hz J_2 = 5.3 Hz, J_3 = 4.6 Hz, 2H), 3.90 (td, J_1 = 12.2 Hz, J_2 = 2.2 Hz, 2H), 2.21 – 2.09 (m, 2H), 2.08 – 1.99 (m, 2H).

4-(4-(Trifluoromethyl)phenyl)tetrahydro-2*H*-pyran-4-carbonitrile (87)

Synthesized from 2-(4-(trifluoromethyl)phenyl)acetonitrile (952 mg, 5.14 mmol, 1.0 equiv), NaH (411 mg, 10.28 mmol, 2.0 equiv), and 1-chloro-2-(2-chloroethoxy)ethane (0.60 mL, 5.14 mmol, 1.0 equiv) according to general procedure E. Column chromatography, EtOAc/*n*-hex = 1/5 (v/v). Yield: 28.5 %; oil (374 mg); ^{1}H NMR (400 MHz, DMSO) δ 7.82 (q, *J* = 8.7 Hz, 4H), 4.07 – 3.99 (m, 2H), 3.67 (ddd, J_1 = 12.3 Hz, J_2 = 9.7 Hz, J_3 = 4.3 Hz, 1H), 2.17 – 2.07 (m, 4H).

4-(3,4-Dimethoxyphenyl)tetrahydro-2*H*-pyran-4-carbonitrile (88)

Synthesized from 2-(3,4-dimethoxyphenyl)acetonitrile (384 mg, 2.17 mmol, 1.0 equiv), NaH (174 mg, 4.34 mmol, 2.0 equiv), and 1-chloro-2-(2-chloroethoxy)ethane (0.25 mL, 2.17 mmol, 1.0 equiv) according to general procedure E. Column chromatography, EtOAc/*n*-hex = 1/5 (v/v). Yield: 64.4 %; oil (345 mg); ^{1}H NMR (400 MHz, DMSO) δ 7.12 (dt, J_1 = 8.3 Hz, J_2 = 2.2 Hz, 2H), 7.06 (d, *J* = 8.3 Hz, 1H), 4.11 – 4.01 (m, 2H), 3.86 (s, 3H), 3.82 (s, 3H), 3.71 (td, J_1 = 11.8 Hz, J_2 = 2.9 Hz, 2H), 2.21 – 2.05 (m, 4H).

4-(4-Methoxyphenyl)tetrahydro-2H-pyran-4-carbonitrile (89)

Synthesized from 2-(4-methoxyphenyl)acetonitrile (612 mg, 4.16 mmol, 1.0 equiv), NaH (333 mg, 8.32 mmol, 2.0 equiv), and 1-chloro-2-(2-chloroethoxy)ethane (0.49 mL, 4.16 mmol, 1.0 equiv) according to general procedure E. Column chromatography, EtOAc/*n*-hex = 1/5 (v/v). Yield: 57.4 %; oil (519 mg); ^{1}H NMR (400 MHz, DMSO) δ 7.49 – 7.42 (m, 2H), 7.03 – 6.96 (m, 2H), 4.03 – 3.95 (m, 2H), 3.77 (s, 3H), 3.64 (td, J_1 = 12.0 Hz, J_2 = 2.3 Hz, 2H), 2.11 – 1.95 (m, 4H).

4-(*p*-Tolyl)tetrahydro-2H-pyran-4-carbonitrile (90)

Synthesized from 2-(*p*-tolyl)acetonitrile (485 mg, 3.70 mmol, 1.0 equiv), NaH (296 mg, 7.4 mmol, 2.0 equiv), and 1-chloro-2-(2-chloroethoxy)ethane (0.43 mL, 3.70 mmol, 1.0 equiv) according to general procedure E. Column chromatography, EtOAc/*n*-hex = 1/5 (v/v). Yield: 77.7 %; oil (578 mg); ^{1}H NMR (400 MHz, DMSO) δ 7.45 – 7.40 (m, 2H), 7.26 (d, *J* = 7.9 Hz, 2H), 4.03 – 3.96 (m, 2H), 3.69 – 3.61 (m, 2H), 2.31 (s, 3H), 2.10 – 1.97 (m, 4H).

4-(Benzo[*d*][1,3]dioxol-5-yl)tetrahydro-2*H*-pyran-4-carbonitrile (91)

Synthesized from 2-(benzo[*d*][1,3]dioxol-5-yl)acetonitrile (255 mg, 1.58 mmol, 1.0 equiv), NaH (126 mg, 3.16 mmol, 2.0 equiv), and 1-chloro-2-(2-chloroethoxy)ethane (0.19 mL, 1.58 mmol, 1.0 equiv) according to general procedure E. Column chromatography, EtOAc/*n*-hex = 1/5 (v/v). Yield: 87.4 %; oil (320 mg); ^{1}H NMR (400 MHz, DMSO) δ 7.15 (d, *J* = 1.9 Hz, 1H), 7.01 (dd, J_1 = 8.2 Hz, J_2 = 2.0 Hz, 1H), 6.96 (d, *J* = 8.2 Hz, 1H), 6.05 (s, 2H), 3.99 (dd, J_1 = 12.4 Hz, J_2 = 2.2 Hz, 2H), 3.63 (td, J_1 = 12.0 Hz, J_2 = 2.2 Hz, 2H), 2.11 – 1.95 (m, 4H).

4-(2-Methoxyphenyl)tetrahydro-2*H*-pyran-4-carbonitrile (92)

Synthesized from 2-(2-methoxyphenyl)acetonitrile (2.21 g, 14.99 mmol, 1.0 equiv), NaH (1.20 g, 29.98 mmol, 2.0 equiv), and 1-chloro-2-(2-chloroethoxy)ethane (1.75 mL, 14.99 mmol, 1.0 equic) according to general procedure E. Column chromatography, EtOAc/*n*-hex = 1/5 (v/v). Yield: 28.9 %; oil (941 mg); ^{1}H NMR (400 MHz, DMSO) δ 7.39 (ddd, J_1 = 8.3 Hz, J_2 = 7.4 Hz, J_3 = 1.6 Hz, 1H), 7.32 (dd, J_1 = 7.8 Hz, J_2 = 1.6 Hz, 1H), 7.14 (dd, J_1 = 8.3 Hz, J_2 = 1.0 Hz, 1H), 7.02 (td, J_1 = 7.6 Hz, J_2 = 1.2 Hz, 1H), 3.98 (dd, J_1 = 11.7 Hz, J_2 = 3.0 Hz, 2H), 3.87 (s, 3H), 3.70 (td, J_1 = 12.2 Hz, J_2 = 1.7 Hz, 2H), 2.26 (dd, J_1 = 13.3 Hz, J_2 = 1.7 Hz, 2H), 2.05 – 1.94 (m, 2H).

4-(3-Methoxyphenyl)tetrahydro-2*H*-pyran-4-carbonitrile (93)

Synthesized from 2-(3-methoxyphenyl)acetonitrile (1.62 g, 10.99 mmol, 1.0 equiv), NaH (879 mg, 21.98 mmol, 2.0 equiv), and 1-chloro-2-(2-chloroethoxy)ethane (1.29 mL, 10.99 mmol, 1.0 equiv) according to general procedure E. Column chromatography, EtOAc/*n*-hex = 1/5 (v/v). Yield: 38.9 %; oil (929 mg); ^{1}H NMR (400 MHz, DMSO) δ 7.37 (t, *J* = 8.0 Hz, 1H), 7.12 (ddd, J_1 = 7.8 Hz, J_2 = 1.8 Hz, J_3 = 0.8 Hz, 1H), 7.06 (t, *J* = 2.1 Hz, 1H), 6.95 (ddd, J_1 = 8.3 Hz, J_2 = 2.5 Hz, J_3 = 0.8 Hz, 1H), 4.06 – 3.96 (m, 2H), 3.79 (s, 3H), 3.70 – 3.61 (m, 2H), 2.12 – 2.00 (m, 4H).

4-(*m*-Tolyl)tetrahydro-2*H*-pyran-4-carbonitrile (94)

Synthesized from 2-(*m*-tolyl)acetonitrile (0.99 g, 7.58 mmol, 1.0 equiv), NaH (606 mg, 15.16 mmol, 2.0 equiv), and 1-chloro-2-(2-chloroethoxy)ethane (0.89 mL, 7.58 mmol, 1.0 equiv) according to general procedure E. Column chromatography, EtOAc/*n*-hex = 1/5 (v/v). Yield: 71.4 %; oil (1.09 g); ^{1}H NMR (400 MHz, DMSO) δ 7.36 (d, *J* = 1.0 Hz, 1H), 7.33 (ddd, J_1 = 6.1 Hz, J_2 = 3.2 Hz, J_3 = 1.3 Hz, 2H), 7.22 – 7.16 (m, 1H), 4.05 – 3.96 (m, 2H), 3.71 – 3.60 (m, 2H), 2.35 (s, 3H), 2.10 – 2.00 (m, 4H).

Compounds 95, 96, 97, 98, 99, 100, 101, 102 and 103 were synthesized according to General procedure C (103 is given as an example).

4-(*m*-Tolyl)tetrahydro-2*H*-pyran-4-carbonitrile (1.09 g, 5.4 mmol, 1.0 equiv) was dissolved in anhydrous THF (30 mL) under argon atmosphere while ice cooling. $LiAlH_4$ (615 mg, 16.2 mmol, 3.0 equiv) was added in portions on ice bath and batch was stirred at room temperature overnight. For workup, diethylether (100 mL) was added to reaction mixture with ice cooling and then aqueous brine solution (5-10 mL) was slowly added while the batch was stirred on ice bath. Residual water was removed by the addition of Na_2SO_4. Precipitate was filtered off and additionally washed with diethylether (3 x 100 mL). Organic solvent was removed under reduced pressure and the product was used without further purification unless stated otherwise.

(4-Phenyltetrahydro-2*H*-pyran-4-yl)methanamine (95)

Synthesized from 4-phenyltetrahydro-2*H*-pyran-4-carbonitrile (5.10 g, 27.24 mmol, 1.0 equiv) and $LiAlH_4$ (3.10 g, 81.72 mmol, 3.0 equiv) according to general procedure C. Yield: 83.5 %; oil (4.35 g); ^{1}H NMR (400 MHz, $CDCl_3$) δ 7.40 – 7.34 (m, 2H), 7.32 – 7.27 (dt, 2H), 7.26 – 7.21 (m, 1H), 3.84 – 3.74 (m, 2H), 3.54 (ddd, J_1 = 11.8 Hz, J_2 = 9.5 Hz, J_3 = 2.7 Hz, 2H), 2.79 (s, 2H), 2.20 – 2.12 (m, 2H), 1.83 (ddd, J_1 = 13.7, J_2 = 9.5 Hz, J_3 = 3.8 Hz, 2H), 0.77 (brs, 2H).

(4-(4-(Trifluoromethyl)phenyl)tetrahydro-2*H*-pyran-4-yl)methanamine (96)

Synthesized from 4-(4-(trifluoromethyl)phenyl)tetrahydro-2*H*-pyran-4-carbonitrile (374 mg, 1.47 mmol, 1.0 equiv) and $LiAlH_4$ (167 mg, 4.40 mmol, 3.0 equiv) according to general procedure C. Yield: 72.4 %; oil (275 mg); ^{1}H NMR (400 MHz, $CDCl_3$) δ 7.64 (d, *J* = 8.2 Hz, 2H), 7.42 (d, *J* = 8.2 Hz, 2H), 3.83 – 3.76 (m, 2H), 3.55 – 3.48 (m, 2H), 2.82 (s, 2H), 2.20 – 2.12 (m, 2H), 1.88 – 1.82 (m, 2H).

(4-(3,4-Dimethoxyphenyl)tetrahydro-2*H*-pyran-4-yl)methanamine (97)

Synthesized from 4-(3,4-dimethoxyphenyl)tetrahydro-2*H*-pyran-4-carbonitrile (345 mg, 1.40 mmol, 1.0 equic) and $LiAlH_4$ (159 mg, 4.19 mmol, 3.0 equiv) according to general procedure C. Yield: 89.4 %; oil (313 mg); ^{1}H NMR (400 MHz, DMSO) δ 6.91 (d, *J* = 8.4 Hz, 1H), 6.85 (d, *J* = 2.2 Hz, 1H), 6.81 (dd, J_1 = 8.4 Hz, J_2 = 2.2 Hz, 1H), 3.75 (s, 3H), 3.74 (s, 3H), 3.69 – 3.63 (m, 2H), 3.39 (ddd, J_1 = 11.6 Hz, J_2 = 9.1 Hz, J_3 = 2.7 Hz, 2H), 2.60 (s, 2H), 1.98 (dd, J_1 = 12.0 Hz, J_2 = 2.3 Hz, 2H), 1.79 – 1.72 (m, 2H), 0.89 (brs, 2H).

(4-(4-Methoxyphenyl)tetrahydro-2H-pyran-4-yl)methanamine (98)

Synthesized from 4-(4-methoxyphenyl)tetrahydro-2H-pyran-4-carbonitrile (519 mg, 2.39 mmol, 1.0 equiv) and $LiAlH_4$ (272 mg, 7.17 mmol, 3.0 equiv) according to general procedure C. Yield: 84.0 %; oil (444 mg); ^{1}H NMR (400 MHz, $CDCl_3$) δ 7.21 (d, *J* = 8.5 Hz, 2H), 6.92 (d, *J* = 8.5 Hz, 2H), 3.82 (s, 3H), 3.81 – 3.76 (m, 2H), 3.54 (t, *J* = 9.6 Hz, 2H), 2.76 (s, 2H), 2.12 (d, *J* = 13.8 Hz, 2H), 1.83 – 1.75 (m, 2H).

(4-(*p*-Tolyl)tetrahydro-2H-pyran-4-yl)methanamine (99)

Synthesized from 4-(*p*-tolyl)tetrahydro-2H-pyran-4-carbonitrile (578 mg, 2.87 mmol, 1.0 equiv) and $LiAlH_4$ (327 mg, 8.62 mmol, 3.0 equiv) according to general procedure C. Yield: 75.2 %; oil (444 mg); ^{1}H NMR (400 MHz, DMSO) δ 7.20 (d, *J* = 8.1 Hz, 2H), 7.15 (d, *J* = 8.1 Hz, 2H), 3.69 – 3.61 (m, 2H), 3.35 (dd, J_1 = 14.9 Hz, J_2 = 5.5 Hz, 2H), 2.59 (s, 2H), 2.28 (s, 3H), 1.99 (d, *J* = 14.1 Hz, 2H), 1.80 – 1.70 (m, 2H), 0.88 (brs, 2H).

(4-(Benzo[*d*][1,3]dioxol-5-yl)tetrahydro-2*H*-pyran-4-yl)methanamine (100)

Synthesized from 4-(benzo[*d*][1,3]dioxol-5-yl)tetrahydro-2*H*-pyran-4-carbonitrile (320 mg, 1.38 mmol, 1.0 equiv) and $LiAlH_4$ (158 mg, 4.15 mmol, 3.0 equiv) according to general procedure C. Yield: 92.5 %; oil (301 mg); ^{1}H NMR (400 MHz, DMSO) δ 6.91 (d, *J* = 1.8 Hz, 1H), 6.87 (d, *J* = 8.2 Hz, 1H), 6.75 (dd, J_1 = 8.2 Hz, J_2 = 1.9 Hz, 1H), 5.98 (s, 2H), 3.68 – 3.60 (m, 2H), 3.37 (ddd, J_1 = 11.6 Hz, J_2 = 9.0 Hz, J_3 = 2.8 Hz, 2H), 2.58 (s, 2H), 1.98 – 1.90 (m, 2H), 1.78 – 1.69 (m, 2H), 0.91 (brs, 2H).

(4-(2-Methoxyphenyl)tetrahydro-2H-pyran-4-yl)methanamine (101)

Synthesized from 4-(2-methoxyphenyl)tetrahydro-2*H*-pyran-4-carbonitrile (941 mg, 4.33 mol, 1.0 equiv) and $LiAlH_4$ (493 mg, 13.00 mmol, 3.0 equiv) according to general procedure C. Yield: 91.9 %; oil (880 mg); ^{1}H NMR (400 MHz, DMSO) δ 7.22 (ddd, J_1 = 8.2 Hz, J_2 = 7.3 Hz, J_3 = 1.7 Hz, 1H), 7.16 (dd, J_1 = 7.8 Hz, J_2 = 1.7 Hz, 1H), 7.00 (dd, J_1 = 8.2 Hz, J_2 = 1.1 Hz, 1H), 6.93 (td, J_1 = 7.7 Hz, J_2 = 1.3 Hz, 1H), 3.75 (s, 3H), 3.70 – 3.62 (m, 2H), 3.46 – 3.36 (m, 2H), 2.87 (s, 2H), 2.24 – 2.13 (m, 2H), 1.87 – 1.78 (m, 2H), 0.79 (brs, 2H).

(4-(3-Methoxyphenyl)tetrahydro-2*H*-pyran-4-yl)methanamine (102)

Synthesized from 4-(3-methoxyphenyl)tetrahydro-2*H*-pyran-4-carbonitrile (929 mg, 4.28 mmol, 1.0 equiv) and $LiAlH_4$ (487 mg, 12.83 mmol, 3.0 equiv) according to general procedure C. Yield: 97.0 %; oil (918 mg); ^{1}H NMR (400 MHz, $CDCl_3$) δ 7.31 (t, *J* = 8.0 Hz, 1H), 6.89 (d, *J* = 7.8 Hz, 1H), 6.85 (s, 1H), 6.79 (d, *J* = 8.2 Hz, 1H), 3.82 (s, 3H), 3.82 – 3.76 (m, 2H), 3.56 (t, *J* = 9.6 Hz, 2H), 2.79 (s, 2H), 2.14 (d, *J* = 14.0 Hz, 2H), 1.84 – 1.77 (m, 2H), 1.56 (brs, 2H).

(4-(*m*-Tolyl)tetrahydro-2*H*-pyran-4-yl)methanamine (103)

Synthesized from 4-(*m*-tolyl)tetrahydro-2*H*-pyran-4-carbonitrile (1.09 g, 5.4 mmol, 1.0 equiv) and $LiAlH_4$ (615 mg, 16.2 mmol, 3.0 equiv) according to general procedure C. Yield: 98.0 %; oil (1.09 g); ^{1}H NMR (400 MHz, $CDCl_3$) δ 7.29 – 7.22 (m, 1H), 7.11 – 7.03 (m, 3H), 3.83 – 3.71 (m, 2H), 3.55 (ddd, J_1 = 11.8 Hz, J_2 = 9.4 Hz, J_3 = 2.7 Hz, 2H), 2.78 (s, 2H), 2.37 (s, 3H), 2.15 (ddd, J_1 = 7.6 Hz, J_2 = 5.3 Hz, J_3 = 2.8 Hz, 2H), 1.87 – 1.76 (m, 2H), 0.92 (brs, 2H).

Compounds 38, 39, 40, 41, 42, 43 and 44 were synthesized according to general procedure A (50 is given as an example).

2-Methoxybenzoic acid (1.9 g, 12.4 mmol, 1.0 equiv) was dissolved in dichloromethane (50 mL) with ice cooling. Oxalyl chloride (3.25 mL, 37.2 mmol, 3.0 equiv) was added dropwise, followed by 5 drops of DMF. The batch was stirred at room temperature overnight and next day the solvent was evaporated. (4-(thiophen-2-yl)tetrahydro-2H-pyran-4-yl)methanamine (2.5 g, 12.4 mmol, 1.0 equiv) and Et_3N (5.2 mL, 37.2 mmol, 3.0 equiv) were dissolved in dichloromethane (50 mL) with ice cooling and then 2-methoxybenzoyl chloride intermediate (12.4 mmol), dissolved in dichloromethane (20 mL), was added dropwise. Reaction mixture was stirred at room temperature overnight. Next day organic phase was diluted with dichloromethane (30 mL) and washed with saturated aqueous $NaHCO_3$ solution (2 x 50 mL), 1M aqueous HCl solution, water (2 x 50mL), saturated brine solution (50 mL), dried over Na_2SO_4, and organic phase was removed under reduced pressure. Product was purified with column chromatography using ethyl acetate:*n*-hexane = 1:1 as mobile phase and additionally using Biotage Isolera One System reversed-phase chromatography (Biotage SNAP Cartridge KP-C18-HS 12 g column), MF: gradient water in H_2O/acetonitril.

***N*-((4-(Benzo[*b*]thiophen-3-yl)tetrahydro-2*H*-pyran-4-yl)methyl)-2-methoxybenzamide (38).**
Synthesized from (4-(benzo[*b*]thiophen-3-yl)tetrahydro-2*H*-pyran-4-yl)methanamine (526 mg, 2.12 mmol, 1.0 equiv), Et_3N (0.89 mL, 6.38 mmol, 3.0 equiv) and 2-methoxybenzoyl chloride (2.12 mmol) according to general procedure A. Column chromatography, EtOAc/*n*-hex = 1/2 (v/v) and Biotage Isolera One System reversed-phase chromatography (Biotage SNAP Cartridge KP-C18-HS 12 g column), MF: gradient water in H_2O/acetonitril. Yield: 23.3 %; white solid (189 mg); ^1H NMR (400 MHz, $CDCl_3$) δ 8.17 (dd, J_1 = 7.8 Hz, J_2 = 1.8 Hz, 1H), 8.12 – 8.07 (m, 1H), 7.96 – 7.90 (m, 1H), 7.55 (t, J = 5.5 Hz, 1H), 7.41 – 7.32 (m, 3H), 7.25 (s, 1H), 7.04 – 6.98 (m, 1H), 6.79 (d, J = 7.8 Hz, 1H), 4.17 (d, J = 5.9 Hz, 2H), 3.97 – 3.88 (m, 2H), 3.79 – 3.70 (m, 2H), 3.46 (s, 3H), 2.38 – 2.27 (m, 2H), 2.20 – 2.09 (m, 2H); ^{13}C NMR (101 MHz, $CDCl_3$) δ 165.38, 157.44, 141.66, 139.30, 136.96, 132.80, 132.32, 124.32, 124.25, 124.09, 123.95, 123.59, 121.20, 120.98, 111.14, 64.05, 55.42, 45.43, 40.91, 34.19 ppm; HRMS (ESI$^+$) for $C_{22}H_{24}NO_3S$ ([M+H]$^+$) calculated 382.1471, found 382.1465; HPLC retention time: 5.227 min (98.70 % at 254 nm).

2-Methoxy-*N*-((4-(4-(4-methoxyphenyl)thiazol-2-yl)tetrahydro-2*H*-pyran-4-yl)methyl)benzamide (39)

Synthesized from (4-(4-(4-methoxyphenyl)thiazol-2-yl)tetrahydro-2*H*-pyran-4-yl)methanamine (288 mg, 0.95 mmol, 1.0 equiv), Et_3N (0.40 mL, 2.84 mmol, 3.0 equiv) and 2-methoxybenzoyl chloride (0.95 mmol) according to general procedure A. Column chromatography, EtOAc/*n*-hex = 1/1 (v/v) and Biotage Isolera One System reversed-phase chromatography (Biotage SNAP Cartridge KP-C18-HS 12 g column), MF: gradient water in H_2O/acetonitril. Yield: 20.5 %; white solid (85 mg); ^1H NMR (400 MHz, $CDCl_3$) δ 8.21 (brs, 1H), 8.17 (dd, J_1 = 7.8 Hz, J_2 = 1.8 Hz, 1H), 7.89 – 7.82 (m, 2H), 7.42 – 7.37 (m, 1H), 7.36 (s, 1H), 7.07 – 7.01 (m, 1H), 6.98 – 6.92 (m, 2H), 6.84 (d, J = 8.3 Hz, 1H), 3.99 (d, J = 6.0 Hz, 2H), 3.97 – 3.90 (m, 2H), 3.86 (s, 3H), 3.78 – 3.67 (m, 2H), 3.53 (s, 3H), 2.33 – 2.23 (m, 2H), 2.04 – 1.97 (m, 2H); ^{13}C NMR (101 MHz, $CDCl_3$) δ 176.37, 165.54, 159.72, 157.59, 154.83, 132.80, 132.32, 127.73, 127.50, 121.35, 121.18, 114.17, 111.25, 110.82, 64.26, 55.66, 55.41, 48.07, 43.71, 35.14; HRMS (ESI$^+$) for $C_{24}H_{27}N_2O_4S$ ([M+H]$^+$) calculated 439.1686, found 439.1678; HPLC retention time: 4.047 min (97.3 % at 254 nm).

2-Methoxy-*N*-((4-(4-phenylthiazol-2-yl)tetrahydro-2*H*-pyran-4-yl)methyl)benzamide (40)
Synthesized from (4-(4-phenylthiazol-2-yl)tetrahydro-2H-pyran-4-yl)methanamine (1.15 g, 4.19 mmol, 1.0 equiv), Et_3N (1.75 mL, 12.57 mmol, 3.0 equiv) and 2-methoxybenzoyl chloride (4.19 mmol) according to general procedure A. Column chromatography, EtOAc/*n*-hex = 1/1 (v/v) and Biotage Isolera One System reversed-phase chromatography (Biotage SNAP Cartridge KP-C18-HS 12 g column), MF: gradient water in H_2O/acetonitril. Yield: 21.4 %; white solid (366 mg); ^1H NMR (400 MHz, $CDCl_3$) δ 8.21 (brs, 1H), 8.17 (dd, J_1 = 7.8 Hz, J_2 = 1.8 Hz, 1H), 7.93 (dd, J_1 = 8.3 Hz, J_2 = 1.2 Hz, 2H), 7.50 (s, 1H), 7.46 – 7.31 (m, 4H), 7.08 – 7.00 (m, 1H), 6.83 (d, J = 8.3 Hz, 1H), 4.00 (d, J = 6.0 Hz, 2H), 3.98 – 3.91 (m, 2H), 3.78 – 3.69 (m, 2H), 3.52 (s, 3H), 2.33 – 2.24 (m, 2H), 2.06 – 1.98 (m, 2H); ^{13}C NMR (101 MHz, $CDCl_3$) δ 176.66, 165.60, 157.62, 155.10, 134.55, 132.84, 132.40, 128.89, 128.34, 126.48, 121.40, 121.25, 112.65, 111.28, 64.31, 55.68, 48.10, 43.83, 35.20 ppm; HRMS (ESI$^+$) for $C_{23}H_{25}N_2O_3S$ ([M+H]$^+$) calculated 409.1580, found 409.1572; HPLC retention time: 4.047 min (97.3 % at 254 nm).

2-Methoxy-*N*-((4-(2-methylthiazol-4-yl)tetrahydro-2*H*-pyran-4-yl)methyl)benzamide (41)
Synthesized from (4-(2-methylthiazol-4-yl)tetrahydro-2*H*-pyran-4-yl)methanamine (257 mg, 1.21 mmol, 1.0 equiv), Et_3N (0.51 mL, 3.63 mmol, 3.0 equiv) and 2-methoxybenzoyl chloride (1.21 mmol) according to general procedure A. Column chromatography, EtOAc/*n*-hex = 1/2 (v/v). Yield: 23.4 %; white solid (98 mg); ^1H NMR (400 MHz, $CDCl_3$) δ 8.18 (dd, J_1 =

7.8 Hz, J_2 = 1.8 Hz, 1H), 7.86 (brs, 1H), 7.45 – 7.38 (m, 1H), 7.09 – 7.03 (m, 1H), 6.92 (dd, J_1 = 8.3 Hz, J_2 = 0.9 Hz, 1H), 6.86 (s, 1H), 3.91 – 3.84 (m, 2H), 3.81 (d, J = 5.9 Hz, 2H), 3.81 (s, 3H), 3.63 – 3.55 (m, 2H), 2.74 (s, 3H), 2.20 – 2.13 (m, 2H), 1.97 – 1.88 (m, 2H); ^{13}C NMR (101 MHz, $CDCl_3$) δ 165.72, 165.45, 160.54, 157.52, 132.76, 132.50, 121.63, 121.40, 113.77, 111.30, 64.35, 55.81, 48.14, 40.57, 33.83, 19.51 ppm; HRMS (ESI$^+$) for $C_{18}H_{23}N_2O_3S$ ([M+H]$^+$) calculated 347.1424, found 347.1416; HPLC retention time: 4.047 min (97.3 % at 254 nm).

2-Methoxy-*N*-((4-(thiazol-4-yl)tetrahydro-2*H*-pyran-4-yl)methyl)benzamide (42).

Synthesized from (4-(thiazol-4-yl)tetrahydro-2*H*-pyran-4-yl)methanamine (148 mg, 0.75 mmol, 1.0 equiv), Et_3N (0.31 mL, 2.24 mmol, 3.0 equiv) and 2-methoxybenzoyl chloride ***(0.75 mmol)*** according to general procedure A. Column chromatography, EtOAc/*n*-hex = 1/2 (v/v). Yield: 24.1 %; white solid (60 mg); ^{1}H NMR (400 MHz, $CDCl_3$) δ 8.88 (d, J = 1.9 Hz, 1H), 8.18 (dd, J_1 = 7.8 Hz, J_2 = 1.8 Hz, 1H), 7.93 (brs, 1H), 7.41 (ddd, J_1 = 8.4 Hz, J_2 = 7.4 Hz, J_3 = 1.9 Hz, 1H), 7.12 (d, J = 1.9 Hz, 1H), 7.07 – 7.01 (m, 1H), 6.91 (d, J = 8.2 Hz, 1H), 3.94 – 3.86 (m, 2H), 3.84 (d, J = 5.9 Hz, 2H), 3.80 (s, 3H), 3.62 – 3.52 (m, 2H), 2.26 – 2.17 (m, 2H), 2.02 – 1.93 (m, 2H); ^{13}C NMR (101 MHz, $CDCl_3$) δ 165.35, 161.76, 157.45, 152.68, 132.74, 132.35, 121.35, 121.25, 114.24, 111.23, 64.21, 55.69, 48.26, 40.55, 33.96 ppm; HRMS (ESI$^+$) for $C_{17}H_{21}N_2O_3S$ ([M+H]$^+$) calculated 333.1267, found 333.1259; HPLC retention time: 3.183 min (98.17 % at 254 nm).

2-Methoxy-*N*-((4-(thiophen-2-yl)tetrahydro-2*H*-pyran-4-yl)methyl)benzamide (43)

Synthesized from(4-(thiophen-2-yl)tetrahydro-2H-pyran-4-yl)methanamine (2.45 g, 12.42 mmol, 1.0 equiv), Et_3N (5.19 mL, 37.26 mmol, 3.0 equiv) and 2-methoxybenzoyl chloride (12.42 mmol) according to general procedure A. Column chromatography, EtOAc/*n*-hex = 1/2 (v/v). Yield: 29.4 %; white solid (1.21 g); ^{1}H NMR (400 MHz, $CDCl_3$) δ 8.20 (dd, J_1 = 7.8 Hz, J_2 = 1.9 Hz, 1H), 7.80 (t, J = 5.2 Hz, 1H), 7.41 (ddd, J_1 = 8.3 Hz, J_2 = 7.3 Hz, J_3 = 1.9 Hz, 1H), 7.31 (dd, J_1 = 5.1 Hz, J_2 = 1.1 Hz, 1H), 7.09 – 7.02 (m, 2H), 6.93 (dd, J_1 = 3.5 Hz, J_2 = 1.1 Hz, 1H), 6.90 (dd, J_1 = 8.3 Hz, J_2 = 0.6 Hz, 1H), 3.90 – 3.83 (m, 2H), 3.74 (d, J = 6.1 Hz, 2H), 3.72 (s, 3H), 3.68 – 3.60 (m, 2H), 2.12 – 2.04 (m, 2H), 2.01 – 1.92 (m, 2H); ^{13}C NMR (101 MHz, $CDCl_3$) δ 165.34, 157.49, 150.06, 132.82, 132.40, 126.99, 124.29, 121.23, 121.12, 111.20, 64.06, 55.54, 50.92, 40.52, 35.59 ppm; HRMS (ESI$^+$) for $C_{18}H_{22}NO_3S$ ([M+H]$^+$) calculated 332.1315, found 332.1309; HPLC retention time: 4.503 min (99.68 % at 254 nm).

2-Methoxy-*N*-((4-(thiophen-3-yl)tetrahydro-2*H*-pyran-4-yl)methyl)benzamide (44)

Synthesized from (4-(thiophen-3-yl)tetrahydro-2H-pyran-4-yl)methanamine (1.97 g, 9.99 mmol, 1.0 equiv), Et_3N (4.18 mL, 29.96 mmol, 3.0 equiv) and 2-methoxybenzoyl chloride (9.99 mmol) according to general procedure A. Column chromatography, EtOAc/*n*-hex = 1/2 (v/v). Yield: 28.1 %; white solid (930 mg); ^{1}H NMR (400 MHz, $CDCl_3$) δ 8.20 (dd, J_1 = 7.8 Hz, J_2 = 1.9 Hz, 1H), 7.66 (brs, 1H), 7.43 – 7.37 (m, 2H), 7.11 – 7.07 (m, 2H), 7.07 – 7.01 (m, 1H), 6.89 (d, J = 7.9 Hz, 1H), 3.90 – 3.81 (m, 2H), 3.73 (d, J = 6.1 Hz, 2H), 3.72 (s, 3H), 3.65 – 3.57 (m, 2H), 2.09 – 2.02 (m, 2H), 1.96 – 1.88 (m, 2H); ^{13}C NMR (101 MHz, $CDCl_3$) δ 165.32, 157.47, 146.35, 132.81, 132.43, 126.34, 126.18, 121.26, 121.22, 121.13, 111.19, 64.13, 55.61, 49.18, 39.46, 34.66 ppm; HRMS (ESI$^+$) for $C_{18}H_{22}NO_3S$ ([M+H]$^+$) calculated 332.1315, found 332.1308; HPLC retention time: 4.457 min (98.42 % at 254 nm).

Compounds 45, 46, 47, 48, 49, 50, and 51 were synthesized according to general procedure A using different acid derivatives as reagents.

2,6-Dimethoxy-*N*-((4-(thiophen-3-yl)tetrahydro-2*H*-pyran-4-yl)methyl)benzamide (45)

2,6-Dimethoxybenzoic acid (200 mg, 1.1 mmol, 1.0 equiv) was dissolved in dichloromethane (30 mL) with ice cooling. Oxalyl chloride (0.3 mL, 3.3 mmol, 3.0 equiv) was added dropwise, followed by 5 drops of DMF. The batch was stirred at room temperature overnight and next day the solvent was evaporated. (4-(Thiophen-3-yl)tetrahydro-2*H*-pyran-4-yl)methanamine (217 mg, 1.1 mmol, 1.0 equiv) and Et_3N (0.5 mL, 3.3 mmol, 3.0 equiv) were dissolved in dichloromethane (30 mL) with ice cooling and then 2,6-dimethoxybenzoyl chloride (1.1 mmol), dissolved in dichloromethane (20 mL), was added dropwise. Reaction was stirred at room temperature overnight. Next day organic phase was diluted with dichloromethane (30 mL) and washed with saturated aqueous $NaHCO_3$ solution (2 x 50 mL), 1M aqueous HCl solution, water (2 x 50 mL), saturated brine solution (50 mL), dried over Na_2SO_4, and remained organic phase was removed under reduced pressure. Product was purified with column chromatography using ethyl acetate:*n*-hexane = 1:1 as mobile phase and additionally using Biotage Isolera One System reversed-phase chromatography (Biotage SNAP Cartridge KP-C18-HS 12 g column), MF: gradient water in H_2O/acetonitril. Yield: 27.1 %; white solid (110 mg); ^{1}H NMR (400 MHz, $CDCl_3$) δ 7.33 (dd, J_1 = 4.9 Hz, J_2 = 3.1 Hz, 1H), 7.24 (t, J = 8.4 Hz, 1H), 7.07 – 7.01 (m, 2H), 6.53 (d, J = 8.4 Hz, 2H), 5.48 (t, J = 6.0 Hz, 1H), 3.93 – 3.85 (m, 2H), 3.79 (s, 6H), 3.65 (d, J = 6.5 Hz, 2H), 3.64 – 3.57 (m, 2H), 2.12 – 2.05 (m,

2H), 2.05 – 1.97 (m, 2H); ^{13}C NMR (101 MHz, $CDCl_3$) δ 165.90, 157.28 (2 x Ar-C), 146.20, 130.65, 126.39, 126.08, 121.11, 116.11, 104.06 (2 x Ar-C), 64.19 (2 x C-O), 55.93 (2 x C-O), 48.67, 39.76, 34.39 ppm; HRMS (ESI$^+$) for $C_{19}H_{24}NO_4S$ ([M+H]$^+$) calculated 362.1421, found 392.1419; HPLC retention time: 3.817 min (98.62 % at 254 nm).

N-((4-(Thiophen-3-yl)tetrahydro-2H-pyran-4-yl)methyl)furan-3-carboxamide (46)

Furan-3-carboxylic acid (170 mg, 1.5 mmol 1.0 equiv) was dissolved in dichloromethane (30 mL) with ice cooling. Oxalyl chloride (0.4 mL, 4.5 mmol, 3.0 equiv) was added dropwise, followed by 5 drops of DMF. The batch was stirred at room temperature overnight and next day the solvent was evaporated. (4-(thiophen-3-yl)tetrahydro-2*H*-pyran-4-yl)methanamine (296 mg, 1.5 mmol, 1.0 equiv) and Et_3N (0.63 mL, 4.5 mmol, 3.0 equiv) were dissolved in dichloromethane (30 mL) with ice cooling and then furan-3-carbonyl chloride (1.5 mmol), dissolved in dichloromethane (20 mL), was added dropwise. Reaction was stirred at room temperature overnight. Next day organic phase was diluted with dichloromethane (30 mL) and washed with saturated aqueous $NaHCO_3$ solution (2 x 50 mL), 1M aqueous HCl solution, water (2 x 50 mL), saturated brine solution (50 mL), dried over Na_2SO_4, and organic phase was removed under reduced pressure. Product was purified with column chromatography using ethyl acetate:*n*-hexane = 1:1 as mobile phase and additionally using Biotage Isolera One System reversed-phase chromatography (Biotage SNAP Cartridge KP-C18-HS 12 g column), MF: gradient water in H_2O/acetonitril. Yield: 27.1 %; white solid (118 mg); ^{1}H NMR (400 MHz, $CDCl_3$) δ 7.81 (dd, J_1 = 1.4 Hz, J_2 = 0.9 Hz, 1H), 7.43 (dd, J_1 = 5.0 Hz, J_2 = 3.0 Hz, 1H), 7.40 (t, J = 1.7 Hz, 1H), 7.09 – 7.02 (m, 2H), 6.42 (dd, J_1 = 1.9 Hz, J_2 = 0.9 Hz, 1H), 5.44 (brs, 1H), 3.89 – 3.80 (m, 2H), 3.60 (d, J = 3.0 Hz, 2H), 3.60 – 3.53 (m, 2H), 2.09 – 2.00 (m, 2H), 1.96 – 1.86 (m, 2H); ^{13}C NMR (101 MHz, $CDCl_3$) δ 162.57, 145.52, 144.70, 143.95, 127.17, 125.97, 122.51, 121.33, 108.08, 64.11, 48.79, 39.93, 34.57 ppm; HRMS (ESI$^+$) for $C_{15}H_{18}NO_3S$ ([M+H]$^+$) calculated 292.10019, found 292.09997; HPLC retention time: 3.253 min (97.97 % at 254 nm).

2-Ethoxy-N-((4-(thiophen-3-yl)tetrahydro-2H-pyran-4-yl)methyl)benzamide (47)

2-Ethoxybenzoic acid (0.23 ml, 1.5 mmol, 1.0 equiv) was dissolved in dichloromethane (30 mL) with ice cooling. Oxalyl chloride (0.4 mL, 4.5 mmol, 3.0 equiv) was added dropwise, followed by 5 drops of DMF The batch was stirred at room temperature overnight and next day the solvent was evaporated. (4-(thiophen-3-yl)tetrahydro-2*H*-pyran-4-yl)methanamine (296 mg, 1.5 mmol, 1.0 equiv) and Et_3N (0.63 mL, 4.5 mmol, 3.0 equiv) were dissolved in dichloromethane (30 mL) with ice cooling and then 2-ethoxybenzoyl chloride (1.5 mmol), dissolved in dichloromethane (20 mL), was added dropwise. Reaction mixture was stirred at room temperature overnight. Next day organic phase was diluted with dichloromethane (30 mL) and washed with saturated aqueous $NaHCO_3$ solution (2 x 50 mL), 1M aqueous HCl solution, water (2 x 50 mL), saturated brine solution (50 mL), dried over Na_2SO_4, and organic phase was removed under reduced pressure. Product was purified with column chromatography using ethyl acetate:*n*-hexane = 1:1 as mobile phase and additionally using Biotage Isolera One System reversed-phase chromatography (Biotage SNAP Cartridge KP-C18-HS 12 g column), MF: gradient water in H_2O/acetonitril. Yield: 27.5 %; white solid (143 mg); ^{1}H NMR (400 MHz, $CDCl_3$) δ 8.21 (dd, J_1 = 7.8 Hz, J_1 = 1.9 Hz, 1H), 7.86 (brs, 1H), 7.43 – 7.34 (m, 2H), 7.11 – 7.01 (m, 3H), 6.89 (d, J = 8.3 Hz, 1H), 4.04 (q, J = 7.0 Hz, 2H), 3.91 – 3.83 (m, 2H), 3.75 (d, J = 6.3 Hz, 2H), 3.65 – 3.56 (m, 2H), 2.09 – 2.00 (m, 2H), 1.98 – 1.88 (m, 2H), 1.18 (t, J = 7.0 Hz, 3H); ^{13}C NMR (101 MHz, $CDCl_3$) δ 165.61, 156.85, 146.40, 132.80, 132.59, 126.38, 126.18, 121.32, 121.21, 121.18, 112.24, 64.50, 64.18, 49.01, 39.73, 34.69, 14.48 ppm; HRMS (ESI$^+$) for $C_{19}H_{24}NO_3S$ ([M+H]$^+$) calculated 346.1471, found 346.1471; HPLC retention time: 4.817 min (100 % at 254 nm).

2-Methoxy-5-methyl-N-((4-(thiophen-3-yl)tetrahydro-2H-pyran-4-yl)methyl)benzamide (48)

2-Methoxy-5-methylbenzoic acid (190 mg, 1.25 mmol, 1.0 equiv) was dissolved in dichloromethane (30 mL) with ice cooling. Oxalyl chloride (0.4 mL, 4.5 mmol, 3.0 equiv) was added dropwise, followed by 5 drops of DMF. The batch was stirred at room temperature overnight and next day the solvent was evaporated. (4-(thiophen-3-yl)tetrahydro-2*H*-pyran-4-yl)methanamine (296 mg, 1.5 mmol, 1.0 equiv) and Et_3N (0.63 mL, 4.5 mmol, 3.0 equiv) were dissolved in dichloromethane (30 mL) with ice cooling and then 2-methoxy-5-methylbenzoyl chloride (1.5 mmol), dissolved in dichloromethane (20 mL), was added dropwise. Reaction mixture was stirred at room temperature overnight. Next day organic phase was diluted with dichloromethane (30 mL) and washed with saturated aqueous $NaHCO_3$ solution (2 x 50 mL), 1M aqueous HCl solution, water (2 x 50 mL), saturated brine solution (50 mL), dried over Na_2SO_4, and organic phase was then removed under reduced pressure. Product was purified with column chromatography using ethyl acetate:*n*-hexane = 1:1 as mobile phase and additionally using Biotage Isolera One System reversed-phase chromatography (Biotage SNAP Cartridge KP-C18-HS 12 g column), MF: gradient water in H_2O/acetonitril. Yield: 28.7 %; white solid (149 mg); ^{1}H NMR (400 MHz, $CDCl_3$) δ 8.01 (d, J = 2.2 Hz, 1H), 7.70 (brs, 1H), 7.41 (dd, J_1 = 5.0 Hz, J_2 = 3.0 Hz, 1H), 7.20 (dd,

J_1 = 8.4 Hz, J_2 = 2.4 Hz, 1H), 7.12 – 7.05 (m, 2H), 6.79 (d, J = 8.4 Hz, 1H), 3.90 – 3.83 (m, 2H), 3.73 (d, J = 6.1 Hz, 2H), 3.69 (s, 3H), 3.65 – 3.57 (m, 2H), 2.30 (s, 3H), 2.10 – 2.02 (m, 2H), 1.97 – 1.88 (m, 2H); ^{13}C NMR (101 MHz, $CDCl_3$) δ 165.55, 155.52, 146.44, 133.25, 132.75, 130.66, 126.33, 126.22, 121.24, 120.67, 111.21, 64.18, 55.71, 49.14, 39.53, 34.69, 20.40 ppm; HRMS (ESI$^+$) for $C_{19}H_{24}NO_3S$ ([M+H]$^+$) calculated 346.1471, found 346.1470; HPLC retention time: 4.847 min (98.82 % at 254 nm).

2-(2-Methoxyphenyl)-*N*-((4-(thiophen-3-yl)tetrahydro-2*H*-pyran-4-yl)methyl)acetamide (49)

2-(2-Methoxyphenyl)acetic acid (200 mg, 1.2 mmol, 1.0 equiv) was dissolved in dichloromethane (30 mL) with ice cooling. Oxalyl chloride (0.32 mL, 3.6 mmol, 3.0 equiv) was added dropwise, followed by 5 drops of DMF. Reaction was stirred at room temperature overnight. The batch was stirred at room temperature overnight and next day the solvent was evaporated. (4-(thiophen-3-yl)tetrahydro-2*H*-pyran-4-yl)methanamine (237 mg, 1.2 mmol, 1.0 equiv) and Et_3N (0.5 mL, 3.6 mmol, 3.0 equiv) were dissolved in dichloromethane (30 mL) with ice cooling and then 2-(2-methoxyphenyl)acetyl chloride (1.2 mmol), dissolved in dichloromethane (20 mL), was added dropwise. Reaction mixture was stirred at room temperature overnight. Next day organic phase was diluted with dichloromethane (30 mL) and washed with saturated aqueous $NaHCO_3$ solution (2 x 50 mL), 1M aqueous HCl solution, water (2 x 50 mL), saturated brine solution (50 mL), dried over Na_2SO_4, and remained organic phase was removed under reduced pressure. Product was purified with column chromatography using ethyl acetate:*n*-hexane = 1:1 as mobile phase and additionally using Biotage Isolera One System reversed-phase chromatography (Biotage SNAP Cartridge KP-C18-HS 12 g column), MF: gradient water in H_2O/acetonitril. Yield: 26.1 %; white solid (108 mg); ^{1}H NMR (400 MHz, $CDCl_3$) δ 7.32 – 7.27 (m, 1H), 7.25 (dd, J_1 = 5.0 Hz, J_2 = 2.9 Hz, 1H), 7.15 (dd, J_1 = 7.4 Hz, J_2 = 1.6 Hz, 1H), 6.93 (td, J_1 = 7.4 Hz, J_2 = 1.0 Hz, 1H), 6.88 (d, J = 8.2 Hz, 1H), 6.77 (dd, J_1 = 5.0 Hz, J_2 = 1.4 Hz, 1H), 6.58 (dd, J_1 = 2.9 Hz, J_2 = 1.4 Hz, 1H), 5.40 (brs, 1H), 3.80 – 3.73 (m, 2H), 3.73 (s, 3H), 3.51 (s, 2H), 3.50 – 3.44 (m, 2H), 3.35 (d, J = 6.2 Hz, 2H), 1.88 – 1.80 (m, 2H), 1.77 – 1.68 (m, 2H); ^{13}C NMR (101 MHz, $CDCl_3$) δ 171.41, 157.01, 145.38, 131.28, 128.98, 126.48, 125.66, 123.81, 121.28, 120.87, 110.86, 64.05, 55.39, 48.55, 39.45, 38.81, 34.32 ppm; HRMS (ESI$^+$) for $C_{19}H_{24}NO_3S$ ([M+H]$^+$) calculated 346.1471, found 346.1469; HPLC retention time: 4.280 min (99.71 % at 254 nm).

5-Fluoro-2-methoxy-*N*-((4-(thiophen-3-yl)tetrahydro-2*H*-pyran-4-yl)methyl)benzamide (50)

5-Fluoro-2-methoxybenzoic acid (260 mg, 1.5 mmol, 1.0 equiv) was dissolved in dichloromethane (30 mL) with ice cooling. Oxalyl chloride (0.4 mL, 4.5 mmol, 3.0 equiv) was added dropwise, followed by 5 drops of DMF. The batch was stirred at room temperature overnight and next day the solvent was evaporated. (4-(thiophen-3-yl)tetrahydro-2*H*-pyran-4-yl)methanamine (296 mg, 1.5 mmol, 1.0 equiv) and Et_3N (0.63 mL, 4.5 mmol, 3.0 equiv) were dissolved in dichloromethane (30 mL) with ice cooling and then 5-fluoro-2-methoxybenzoyl chloride (1.5 mmol), dissolved in dichloromethane (20 mL), was added dropwise. Reaction mixture was stirred at room temperature overnight. Next day organic phase was diluted with dichloromethane (30 mL) and washed with saturated aqueous $NaHCO_3$ solution (2 x 50 mL), 1M aqueous HCl solution, water (2 x 50mL), saturated brine solution (50 mL), dried over Na_2SO_4, and remained organic phase was removed under reduced pressure. Product was purified with column chromatography using ethyl acetate:*n*-hexane = 1:1 as mobile phase and additionally using Biotage Isolera One System reversed-phase chromatography (Biotage SNAP Cartridge KP-C18-HS 12 g column), MF: gradient water in H_2O/acetonitril. Yield: 30.3 %; white solid (159 mg); ^{1}H NMR (400 MHz, $CDCl_3$) δ 7.91 (dd, J_1 = 9.5 Hz, J_2 = 3.3 Hz, 1H), 7.69 (brs, 1H), 7.44 – 7.39 (m, 1H), 7.13 – 7.06 (m, 3H), 6.84 (dd, J_1 = 9.1 Hz, J_2 = 4.1 Hz, 1H), 3.89 – 3.82 (m, 2H), 3.72 (d, J = 6.1 Hz, 2H), 3.71 (s, 3H), 3.65 – 3.57 (m, 2H), 2.10 – 2.03 (m, 2H), 1.96 – 1.87 (m, 2H); ^{13}C NMR (101 MHz, $CDCl_3$) δ 164.17 (d, J = 1.8 Hz), 157.19 (d, J = 239.9 Hz), 153.65 (d, J = 2.1 Hz), 146.24, 126.50, 126.20, 122.67 (d, J = 6.7 Hz), 121.33, 119.05 (d, J = 31.9 Hz), 118.81 (d, J = 33.6 Hz), 112.56 (d, J = 7.6 Hz), 64.15, 56.26, 49.40, 39.49, 34.71 ppm; HRMS (ESI$^+$) for $C_{18}H_{21}FNO_3S$ ([M+H]$^+$) calculated 350.1221, found 350.1220; HPLC retention time: 4.623 min (98.55 % at 254 nm).

2-Methoxy-4-methyl-*N*-((4-(thiophen-3-yl)tetrahydro-2*H*-pyran-4-yl)methyl)benzamide (51)

2-Methoxy-4-methylbenzoic acid (250 mg, 1.5 mmol, 1.0 equiv) was dissolved in dichloromethane (30 mL) with ice cooling. Oxalyl chloride (0.4 mL, 4.5 mmol, 3.0 equiv) was added dropwise, followed by 5 drops of DMF. The batch was stirred at room temperature overnight and next day the solvent was evaporated. (4-(thiophen-3-yl)tetrahydro-2*H*-pyran-4-yl)methanamine (296 mg, 1.5 mmol, 1.0 equiv) and Et_3N (0.63 mL, 4.5 mmol, 3.0 equiv) were dissolved in dichloromethane (30 mL) with ice cooling and then 2-methoxy-4-methylbenzoyl chloride (1.5 mmol), dissolved in dichloromethane (20 mL), was added dropwise. Reaction mixture was stirred at room temperature overnight. Next day organic phase was diluted with dichloromethane (30 mL) and washed with saturated aqueous $NaHCO_3$ solution (2 x 50 mL),

1M aqueous HCl solution (1 x 50 mL), water (2 x 50 mL), saturated brine solution (50 mL), dried over Na_2SO_4, and remained organic phase was removed under reduced pressure. Product was purified with column chromatography using ethyl acetate:*n*-hexane = 1:1 as mobile phase and additionally using Biotage Isolera One System reversed-phase chromatography (Biotage SNAP Cartridge KP-C18-HS 12 g column), MF: gradient water in H_2O/acetonitril. Yield: 29.1 %; white solid (151 mg); ^1H NMR (400 MHz, $CDCl_3$) δ 8.08 (d, *J* = 8.0 Hz, 1H), 7.63 (t, *J* = 5.2 Hz, 1H), 7.41 (dd, J_1 = 5.0 Hz, J_2 = 3.0 Hz, 1H), 7.11 – 7.05 (m, 2H), 6.86 (dd, J_1 = 8.0 Hz, J_2 = 0.7 Hz, 1H), 6.69 (s, 1H), 3.90 – 3.81 (m, 2H), 3.72 (d, *J* = 6.1 Hz, 2H), 3.70 (s, 3H), 3.65 – 3.57 (m, 2H), 2.36 (s, 3H), 2.09 – 2.02 (m, 2H), 1.96 – 1.88 (m, 2H); ^{13}C NMR (101 MHz, $CDCl_3$) δ 165.45, 157.44, 146.47, 143.67, 132.45, 126.32, 126.25, 122.12, 121.22, 118.46, 111.93, 64.19, 55.55, 49.12, 39.53, 34.70, 21.80 ppm; HRMS (ESI$^+$) for $C_{19}H_{24}NO_3S$ ([M+H]$^+$) calculated 346.1471, found 346.1470; HPLC retention time: 4.797 min (99.40 % at 254 nm).

Tetrahydro-2*H*-pyran analogues 104, 105, 106, 107, 108, 109, and 110 were synthesized according to general procedure E (110 is given as an example).

2-(Thiophen-2-yl)acetonitrile (2 mL, 19 mmol, 1.0 equiv) was slowly added to a stirred solution of NaH (60 % dispersion in mineral oil) (1.52 g, 38 mmol, 2.0 equiv) in anhydrous DMF (50 mL) while ice cooling under argon atmosphere. The batch was stirred for 15 minutes and then solution of 1-chloro-2-(2-chloroethoxy)ethane (2.2 mL, 19 mmol, 1.0 equiv) in anhydrous DMF (30 mL) was added dropwise while ice cooling. The batch was stirred at room temperature overnight. Next day water (50 mL) was added to the reaction mixture and the reaction mixture was washed with diethylether (3 x 100 mL). Combined organic phases were washed with saturated brine solution (2 x 100 mL), dried over Na_2SO_4, filtered and then solvent was removed under reduced pressure. Product was purified with column chromatography using ethyl acetate:*n*-hexane = 1:4 as mobile phase.

4-(Benzo[*b*]thiophen-3-yl)tetrahydro-2*H*-pyran-4-carbonitrile (104)

Synthesized from 2-(benzo[*b*]thiophen-3-yl)acetonitrile (1.41g, 8.12 mmol, 1.0 equiv), NaH (650 mg, 16.24 mmol, 2.0 equiv) and 1-chloro-2-(2-chloroethoxy)ethane (0.95 mL, 8.12 mmol, 1.0 equiv) according to general procedure E. Column chromatography, EtOAc/*n*-hex = 1/4 (v/v). Yield: 27.5 %; oil (543 mg); ^1H NMR (400 MHz, $CDCl_3$) δ 8.13 (dd, J_1 = 7.4 Hz, J_2 = 1.1 Hz, 1H), 7.90 (ddd, J_1 = 5.0 Hz, J_2 = 3.1 Hz, J_3 = 2.2 Hz, 1H), 7.48 – 7.39 (m, 2H), 7.37 (s, 1H), 4.11 (ddd, J_1 = 11.5 Hz, J_2 = 3.5 Hz, J_3 = 1.0 Hz, 2H), 4.01 (td, J_1 = 12.2 Hz, J_2 = 1.9 Hz, 2H), 2.40 (dd, J_1 = 13.6 Hz, J_2 = 1.9 Hz, 2H), 2.24 (ddd, J_1 = 13.7 Hz, J_2 = 12.0 Hz, J_3 = 4.6 Hz, 2H).

4-(Thiophen-3-yl)tetrahydro-2*H*-pyran-4-carbonitrile (105)

Synthesized from 2-(thiophen-3-yl)acetonitrile, NaH (650 mg, 16.24 mmol, 2.0 equiv) and 1-chloro-2-(2-chloroethoxy)ethane (0.95 mL, 8.12 mmol, 1.0 equiv) according to general procedure E. Column chromatography, EtOAc/*n*-hex = 1/4 (v/v). Yield: 42.4 %; yellow oil (3.5 g); ^1H NMR (400 MHz, $CDCl_3$) δ 7.39 (dd, J_1 = 5.1 Hz, J_2 = 3.0 Hz, 1H), 7.31 (dd, J_1 = 3.0 Hz, J_2 = 1.5 Hz, 1H), 7.14 (dd, J_1 = 5.1 Hz, J_2= 1.5 Hz, 1H), 4.05 (dt, J_1 = 12.1 Hz, J_2 = 3.2 Hz, 2H), 3.86 (ddd, J_1 = 12.4 Hz, J_2 = 9.2 Hz, J_3 = 5.0 Hz, 2H), 2.12 – 2.07 (m, 4H).

4-(4-(4-Methoxyphenyl)thiazol-2-yl)tetrahydro-2*H*-pyran-4-carbonitrile (106)

Synthesized from 2-(4-(4-methoxyphenyl)thiazol-2-yl)acetonitrile (1.11 g, 4.80 mmol, 1.0 equiv), NaH (384 mg, 9.60 mmol, 2.0 equiv) and 1-chloro-2-(2-chloroethoxy)ethane (0.56 mL, 4.80 mmol, 1.0 equiv) according to general procedure E. Column chromatography, EtOAc/*n*-hex = 1/4 (v/v). Yield: 20.8 %; yellow solid (300 mg); ^1H NMR (400 MHz, $CDCl_3$) δ 7.87 – 7.83 (m, 2H), 7.35 (s, 1H), 6.99 – 6.91 (m, 2H), 4.11 – 4.05 (m, 2H), 3.94 – 3.86 (m, 2H), 3.85 (s, 3H), 2.45 – 2.27 (m, 4H).

4-(4-Phenylthiazol-2-yl)tetrahydro-2H-pyran-4-carbonitrile (107)

Synthesized from 2-(4-phenylthiazol-2-yl)acetonitrile (2.1 g, 10.52 mmol, 1.0 equiv), NaH (842mg, 21.04 mmol, 2.0 equiv) and 1-chloro-2-(2-chloroethoxy)ethane (1.23 mL, 10.52 mmol, 1.0 equiv) according to general procedure E. Column chromatography, EtOAc/*n*-hex = 1/4 (v/v). Yield: 59.4 %; yellow solid (1.69 g); ^1H NMR (400 MHz, $CDCl_3$) δ 7.90 (dt, J_1 = 8.3, Hz J_2 = 1.8 Hz, 2H), 7.49 (s, 1H), 7.46 – 7.39 (m, 2H), 7.38 – 7.32 (m, 1H), 4.12 – 4.04 (m, 2H), 3.94 – 3.85 (m, 2H), 2.45 – 2.28 (m, 4H).

4-(2-Methylthiazol-4-yl)tetrahydro-2*H*-pyran-4-carbonitrile (108)

Synthesized from 2-(2-methylthiazol-4-yl)acetonitrile (400 mg, 2.90 mmol, 1.0 equiv), NaH (232mg, 5.79 mmol, 2.0 equiv) and 1-chloro-2-(2-chloroethoxy)ethane (0.34 mL, 2.90 mmol, 1.0 equiv) according to general procedure E. Column

chromatography, EtOAc/*n*-hex = 1/4 (v/v). Yield: 43.6 %; oil (263 mg); ^{1}H NMR (400 MHz, $CDCl_3$) δ 7.13 (s, 1H), 4.08 – 4.01 (m, 2H), 3.85 (td, J_1 = 12.2 Hz, J_2 = 2.1 Hz, 2H), 2.71 (s, 3H), 2.28 (ddd, J_1 = 13.7 Hz, J_2 = 12.2 Hz, J_3 = 4.5 Hz, 2H), 2.10 (dd, J_1 = 13.7 Hz, J_2 = 2.0 Hz, 2H).

4-(Thiazol-4-yl)tetrahydro-2*H*-pyran-4-carbonitrile (109)

Synthesized from 2-(thiazol-4-yl)acetonitrile (318 mg, 2.56 mmol, 1.0 equiv), NaH (205mg, 5.12 mmol, 2.0 equiv) and 1-chloro-2-(2-chloroethoxy)ethane (0.30 mL, 2.56 mmol, 1.0 equiv) according to general procedure E. Column chromatography, EtOAc/*n*-hex = 1/4 (v/v). Yield: 40.0 %; oil (199 mg); ^{1}H NMR (400 MHz, $CDCl_3$) δ 8.84 (d, J = 2.0 Hz, 1H), 7.40 (d, J = 2.0 Hz, 1H), 4.12 – 4.03 (m, 2H), 3.88 (td, J_1 = 12.3 Hz, J_2 = 2.1 Hz, 2H), 2.33 (ddd, J_1 = 13.8 Hz, J_2 = 12.2 Hz, J_3 = 4.5 Hz, 2H), 2.13 (dd, J_1 = 13.7 Hz, J_2 = 2.0 Hz, 2H).

4-(Thiophen-2-yl)tetrahydro-2H-pyran-4-carbonitrile (110)

Synthesized from 2-(thiophen-2-yl)acetonitrile (2.34 g, 19.04 mmol, 1.0 equiv), NaH (1.52 g, 38.08 mmol, 2.0 equiv) and 1-chloro-2-(2-chloroethoxy)ethane (2.23 mL, 19.04 mmol, 1.0 equiv) according to general procedure E. Column chromatography, EtOAc/*n*-hex = 1/4 (v/v). Yield: 76.1 %; oil (2.8 g); ^{1}H NMR (400 MHz, DMSO) δ 7.59 (dd, J_1 = 5.1 Hz, J_2 = 1.2 Hz, 1H), 7.25 (dd, J_1 = 3.6 Hz, J_2 = 1.2 Hz, 1H), 7.08 (dd, J_1 = 5.1 Hz, J_2 = 3.6 Hz, 1H), 3.97 (ddd, J_1 = 6.4 Hz, J_2 = 3.8 Hz, J_3 = 2.1 Hz, 2H), 3.63 (td, J_1 = 12.2 Hz, J_2 = 2.1 Hz, 2H), 2.29 – 2.20 (m, 2H), 2.05 (ddd, J_1 = 13.7 Hz, J_2 = 11.7 Hz, J_3 = 4.3 Hz, 2H).

Compounds 111, 112, 113, 114, 115, 116, and 117 were synthesized according to general procedure C (117 is given as an example).

4-(Thiophen-2-yl)tetrahydro-2*H*-pyran-4-carbonitrile (2.8 g, 14.5 mmol, 1.0 equiv) was dissolved in anhydrous THF (50 mL) under argon atmosphere while ice cooling. $LiAlH_4$ (1.65 g, 43.5 mmol, 3.0 equiv) was added in portions on ice bath and the batch was stirred at room temperature overnight. For workup, diethylether (200 mL) was added to reaction mixture with ice cooling and then aqueous brine solution (5-10 mL) was slowly added while the batch was stirred on ice bath. Residual water was removed by addition of Na_2SO_4. Precipitate was filtered off and additionally washed with diethylether. Organic solvent was removed under reduced pressure and the product was used without further purification unless stated otherwise.

(4-(Benzo[*b*]thiophen-3-yl)tetrahydro-2*H*-pyran-4-yl)methanamine (111)

Synthesized from 4-(benzo[*b*]thiophen-3-yl)tetrahydro-2*H*-pyran-4-carbonitrile (543 mg, 2.23 mmol, 1.0 equiv) and $LiAlH_4$ (254 mg, 6.69 mmol, 3.0 equiv) according to general procedure C. Yield: 95.2 %; oil (526 mg); ^{1}H NMR (400 MHz, $CDCl_3$) δ 8.01 – 7.96 (m, 1H), 7.92 – 7.86 (m, 1H), 7.36 – 7.30 (m, 2H), 7.19 (s, 1H), 3.84 (ddd, J_1 = 11.6 Hz, J_2 = 5.3 Hz, J_3 = 4.0 Hz, 2H), 3.70 – 3.63 (m, 2H), 3.19 (s, 2H), 2.43 – 2.34 (m, 2H), 2.04 – 1.94 (m, 2H).

(4-(Thiophen-3-yl)tetrahydro-2H-pyran-4-yl)methanamine (112)

Synthesized from 4-(thiophen-3-yl)tetrahydro-2*H*-pyran-4-carbonitrile (3.5 g, 18.11 mmol, 1.0 equiv) and $LiAlH_4$ (2.06 g, 54.33 mmol, 3.0 equiv) according to general procedure C. Yield: 55.4 %; yellow oil (1.97 g); ^{1}H NMR (400 MHz, DMSO) δ 7.50 (dd, J_1 = 5.0 Hz, J_2 = 2.9 Hz, 1H), 7.22 (dd, J_1 = 2.9 Hz, J_2 = 1.4 Hz, 1H), 7.07 (dd, J_1 = 5.0 Hz, J_2 = 1.4 Hz, 1H), 3.67 (dt, J_1 = 11.5 Hz, J_2 = 4.2 Hz, 2H), 3.36 – 3.30 (m, 2H), 2.56 (s, 2H), 1.98 – 1.91 (m, 2H), 1.71 (ddd, J_1 = 17.8 Hz, J_2 = 9.4 Hz, J_3 = 4.4 Hz, 2H), 0.95 (brs, 2H).

(4-(4-(4-Methoxyphenyl)thiazol-2-yl)tetrahydro-2*H*-pyran-4-yl)methanamine (113)

Synthesized from 4-(4-(4-methoxyphenyl)thiazol-2-yl)tetrahydro-2*H*-pyran-4-carbonitrile (300 mg, 1.0 mmol, 1.0 equiv) and $LiAlH_4$ (114 mg, 3.0 mmol, 3.0 equiv) according to general procedure C. Yield: 95.0 %; oil (288 mg); ^{1}H NMR (400 MHz, $CDCl_3$) δ 7.86 – 7.79 (m, 2H), 7.32 (s, 1H), 6.97 – 6.90 (m, 2H), 4.09 (ddd, J_1 = 11.4 Hz, J_2 = 4.0 Hz, J_3 = 2.1 Hz, 2H), 3.84 (s, 3H), 3.65 – 3.58 (m, 2H), 2.91 (s, 2H), 2.35 – 2.27 (m, 2H), 2.14 – 2.06 (m, 2H).

(4-(4-Phenylthiazol-2-yl)tetrahydro-2H-pyran-4-yl)methanamine (114)

Synthesized from 4-(4-phenylthiazol-2-yl)tetrahydro-2H-pyran-4-carbonitrile (1.69 g, 6.25 mmol, 1.0 equiv) and $LiAlH_4$ (712 mg, 18.75 mmol, 3.0 equiv) according to general procedure C. Yield: 67.2 %; yellow solid (1.15 g); ^{1}H NMR (400 MHz, $CDCl_3$) δ 7.94 – 7.85 (m, 2H), 7.46 – 7.37 (m, 2H), 7.36 (s, 1H), 7.35 – 7.29 (m, 1H), 4.14 – 4.04 (m, 2H), 3.65 – 3.53 (m, 2H), 2.92 (s, 2H), 2.32 (d, J = 13.7 Hz, 2H), 2.15 – 2.04 (m, 2H).

(4-(2-Methylthiazol-4-yl)tetrahydro-2*H*-pyran-4-yl)methanamine (115)

Synthesized from 4-(2-methylthiazol-4-yl)tetrahydro-2*H*-pyran-4-carbonitrile (263 mg, 1.26 mmol, 1.0 equiv) and $LiAlH_4$ (144 mg, 3.79 mmol, 3.0 equiv) according to general procedure C. Yield: 95.6 %; oil (257 mg); ^{1}H NMR (400 MHz, $CDCl_3$) δ 6.80 (s, 1H), 3.82 (dt, J_1 = 11.7 Hz, J_2 = 4.1 Hz, 2H), 3.53 – 3.46 (m, 2H), 2.83 (s, 2H), 2.70 (s, 3H), 2.22 – 2.16 (m, 2H), 1.81 – 1.73 (m, 2H).

(4-(Thiazol-4-yl)tetrahydro-2*H*-pyran-4-yl)methanamine (116)

Synthesized from 4-(thiazol-4-yl)tetrahydro-2*H*-pyran-4-carbonitrile (199 mg, 1.02 mmol, 1.0 equiv) and $LiAlH_4$ (117 mg, 3.07 mmol, 3.0 equiv) according to general procedure C. Yield: 73.1 %; oil (148 mg); ^{1}H NMR (400 MHz, $CDCl_3$) δ 8.83 (d, J = 1.9 Hz, 1H), 7.06 (d, J = 1.9 Hz, 1H), 3.84 (dt, J_1 = 11.7 Hz, J_2 = 4.2 Hz, 2H), 3.47 (ddd, J_1 = 14.2 Hz, J_2 = 10.2 Hz, J_3 = 2.5 Hz, 2H), 2.87 (s, 2H), 2.28 – 2.21 (m, 2H), 1.84 (ddd, J_1 = 14.2 Hz, J_2 = 8.8 Hz, J_3 = 4.2 Hz, 2H).

(4-(Thiophen-2-yl)tetrahydro-2H-pyran-4-yl)methanamine (117)

Synthesized from 4-(thiophen-2-yl)tetrahydro-2H-pyran-4-carbonitrile (2.8 g, 14.49 mmol, 1.0 equiv) and $LiAlH_4$ (1.65 g, 43.46 mmol, 3.0 equiv) according to general procedure C. Yield: 85.2 %; oil (2.4 g); ^{1}H NMR (400 MHz, $CDCl_3$) δ 7.25 (dd, J_1 = 5.1 Hz, J_2 = 1.1 Hz, 1H), 7.01 (dd, J_1 = 5.1 Hz, J_2 = 3.5 Hz, 1H), 6.85 (dt, J_1 = 4.7 Hz, J_2 = 2.4 Hz, 1H), 3.83 (dt, J_1 = 11.7 Hz, J_2 = 4.0 Hz, 2H), 3.62 – 3.52 (m, 2H), 2.75 (s, 2H), 2.06 (ddd, J_1 = 5.8 Hz, J_2 = 3.6 Hz, J_2 = 1.8 Hz, 2H), 1.88 – 1.76 (m, 2H), 1.05 (brs, 2H).

General procedure F: The Hantzsch thiazole synthesis of compounds 118 and 119 (118 is given as an example).

2-Bromo-1-(4-methoxyphenyl)ethan-1-one (3 g, 13.1 mmol, 1.0 equiv) was dissolved in anhydrous DMF (50 mL) while ice cooling. The batch was stirred for 10 minutes and then Et_3N (5.4 mL, 39.3 mmol, 3.0 equiv) was added dropwise. Solution of 2-cyanoethanethioamide (1.3 g, 13.1 mmol) in anhydrous DMF was added dropwise while ice cooling. The batch was stirred at room temperature overnight. For workup, 1M aqueous HCl solution (1 x 50 mL) was added and washed with diethylether (3 x 100 mL). Combined organic phases were washed aqueous brine solution (2 x 100 mL), dried over Na_2SO_4, filtered and solvent was removed under reduced pressure. Product was purified with column chromatography using EtOAc:*n*-hexane = 1:4 as mobile phase. 1.1 g of yellow solid was isolated.

2-(4-(4-Methoxyphenyl)thiazol-2-yl)acetonitrile (118)

Synthesized from 2-bromo-1-(4-methoxyphenyl)ethan-1-one (3 g, 13.1 mmol, 1.0 equiv), Et_3N (5.4 mL, 39.3 mmol, 3.0 equiv), and 2-cyanoethanethioamide (1.3 g, 13.1 mmol) according to general procedure F. Column chromatography, EtOAc/*n*-hex = 1/4 (v/v). Yield: 36.6 %; yellow solid (1.11 g); ^{1}H NMR (400 MHz, $CDCl_3$) δ 7.84 – 7.77 (m, 2H), 7.34 (s, 1H), 6.98 – 6.91 (m, 2H), 4.16 (s, 2H), 3.85 (s, 3H).

2-(4-Phenylthiazol-2-yl)acetonitrile (119)

Synthesized from 2-bromo-1-phenylethan-1-one (3 g, 15 mmol, 1.0 equiv), Et_3N (6.3 mL, 45 mmol, 3.0 equiv), and 2-cyanoethanethioamide (1.5 g, 15 mmol, 1.0 equiv) according to general procedure F. Column chromatography, EtOAc/*n*-hex = 1/4 (v/v). Yield: 70.0 %; yellow solid (2.10 g); ^{1}H NMR (400 MHz, $CDCl_3$) δ 7.90 – 7.84 (m, 2H), 7.46 (s, 1H), 7.45 – 7.40 (m, 2H), 7.38 – 7.32 (m, 1H), 4.17 (s, 2H).

^{1}H and [13]C Spectra of compounds

2-Methoxy-*N*-((1-(4-methoxyphenyl)cyclopentyl)methyl)benzamide (6)

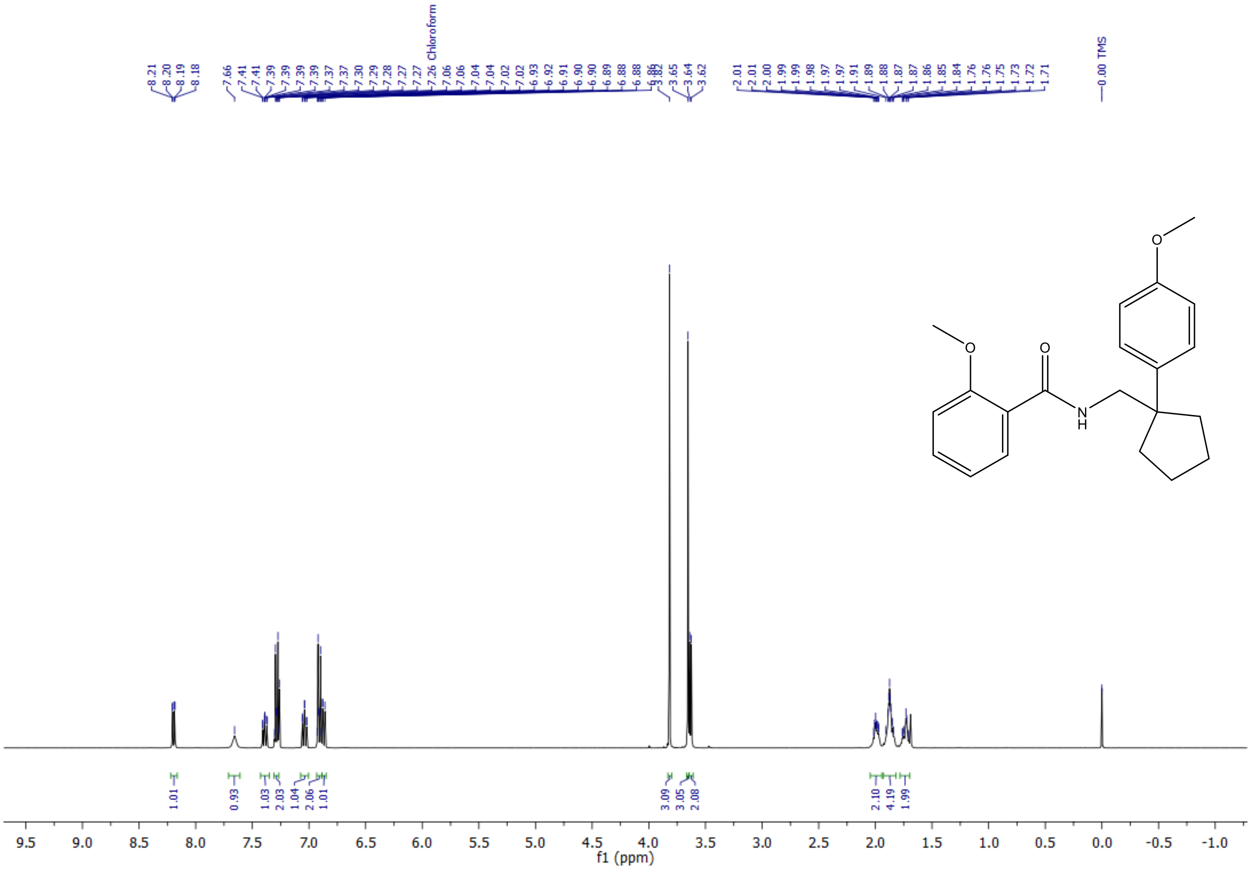

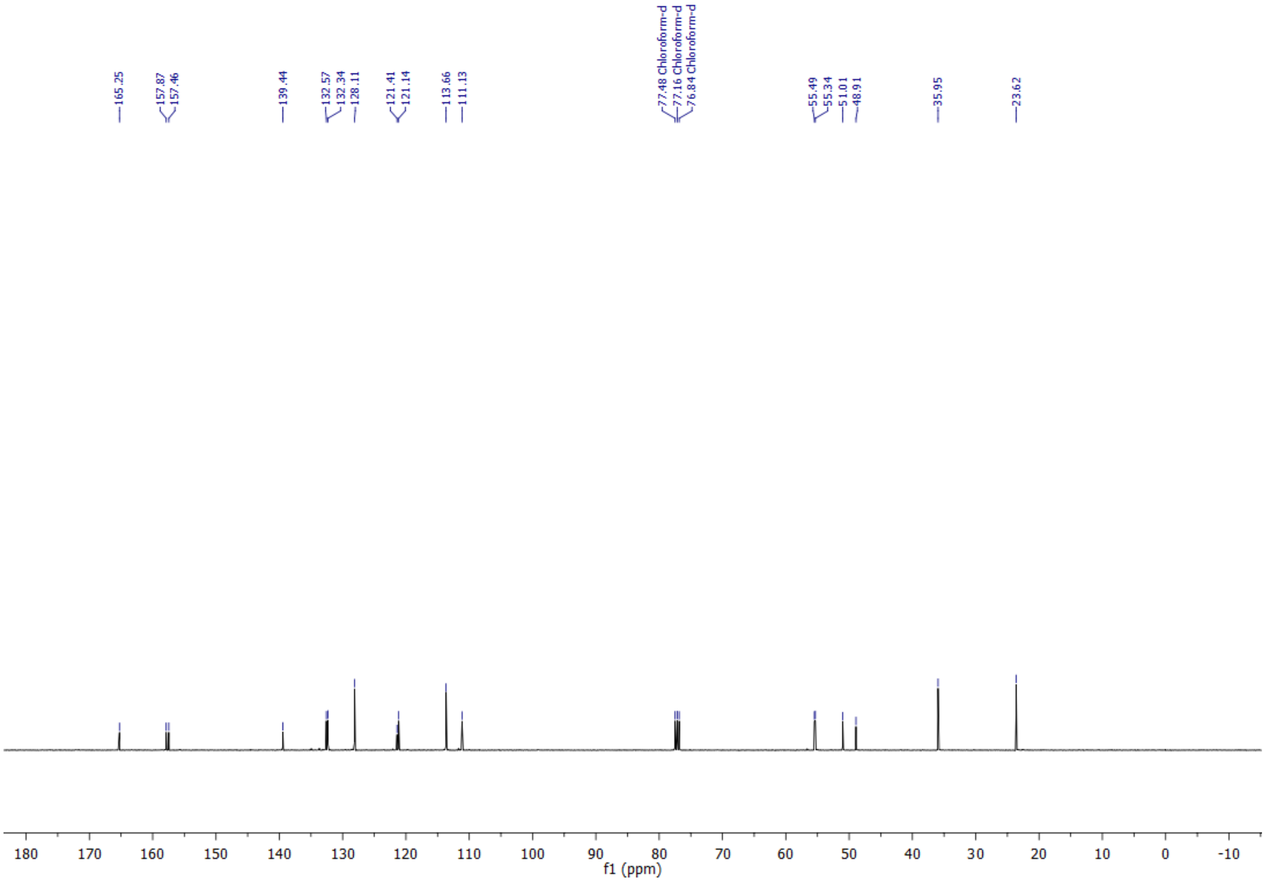

2-Methoxy-*N*-((1-(2-methoxyphenyl)cyclopentyl)methyl)benzamide (7)

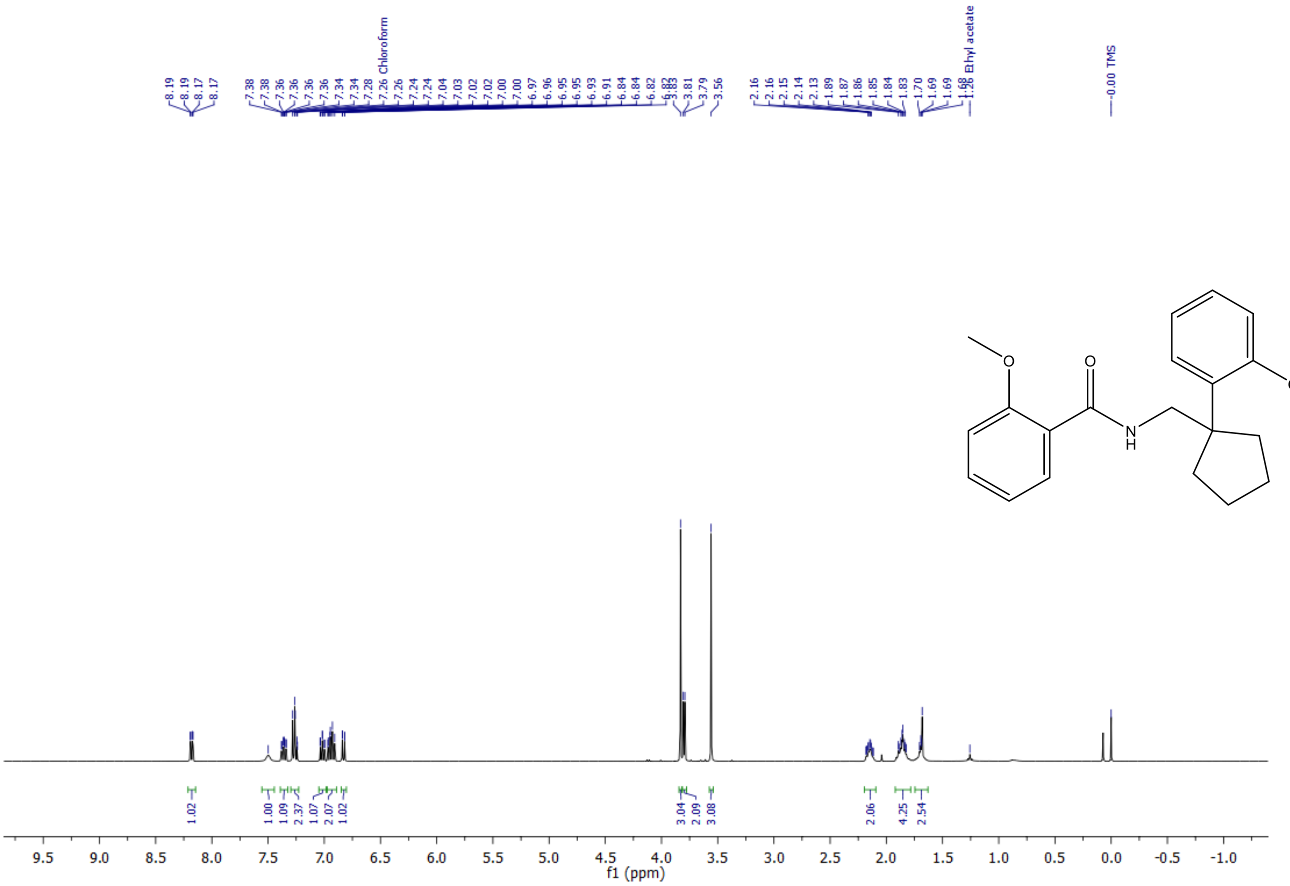
Chloroform
Ethyl acetate
0.00 TMS
O
O
N
H
O
f1 (ppm)

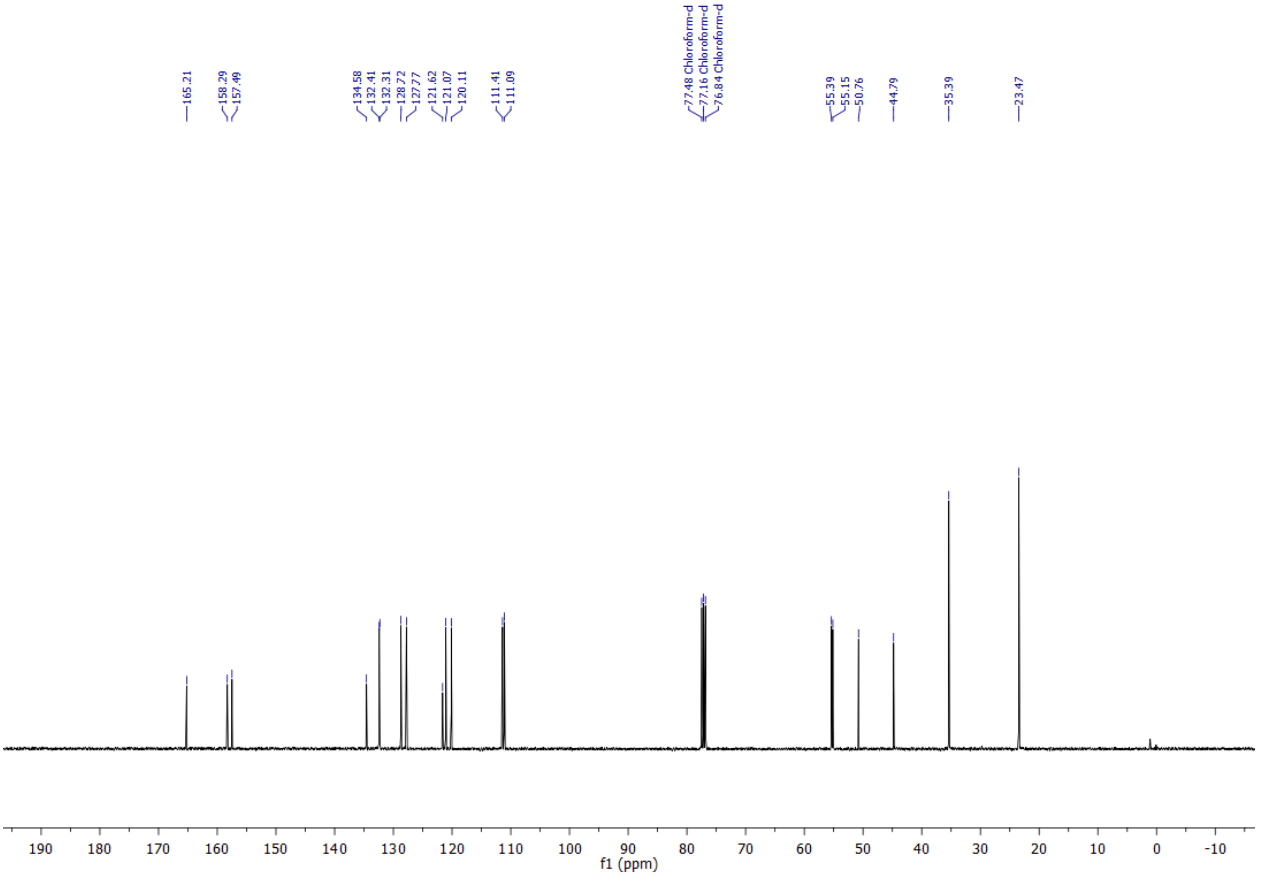
165.21
158.29
157.49
134.58
132.41
132.31
128.72
127.77
121.62
121.07
120.11
111.41
111.09
77.48 Chloroform-d
77.16 Chloroform-d
76.84 Chloroform-d
55.39
55.15
50.76
44.79
35.39
23.47
f1 (ppm)

***N*-((1-(3,4-Dimethoxyphenyl)cyclopentyl)methyl)-2-methoxybenzamide (8)**

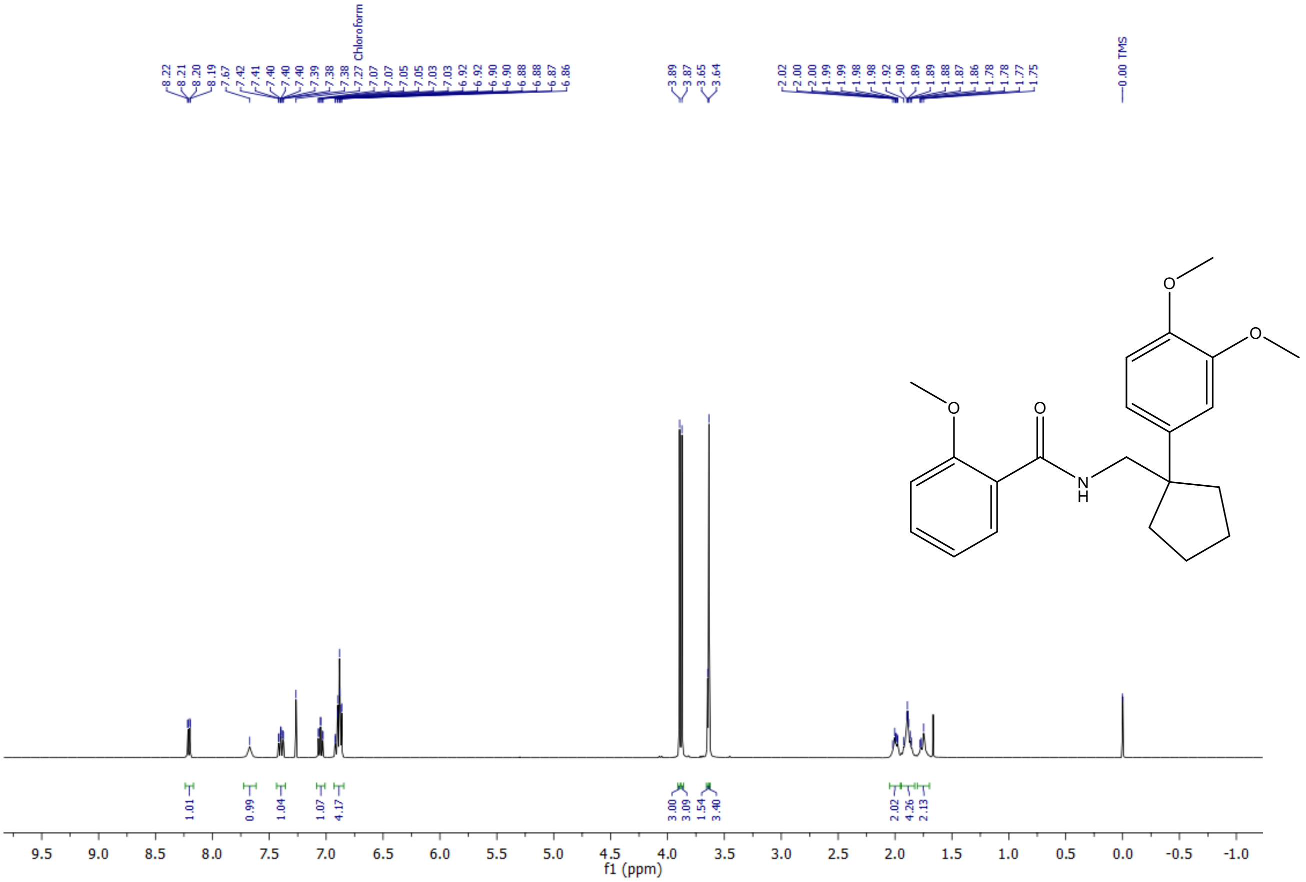

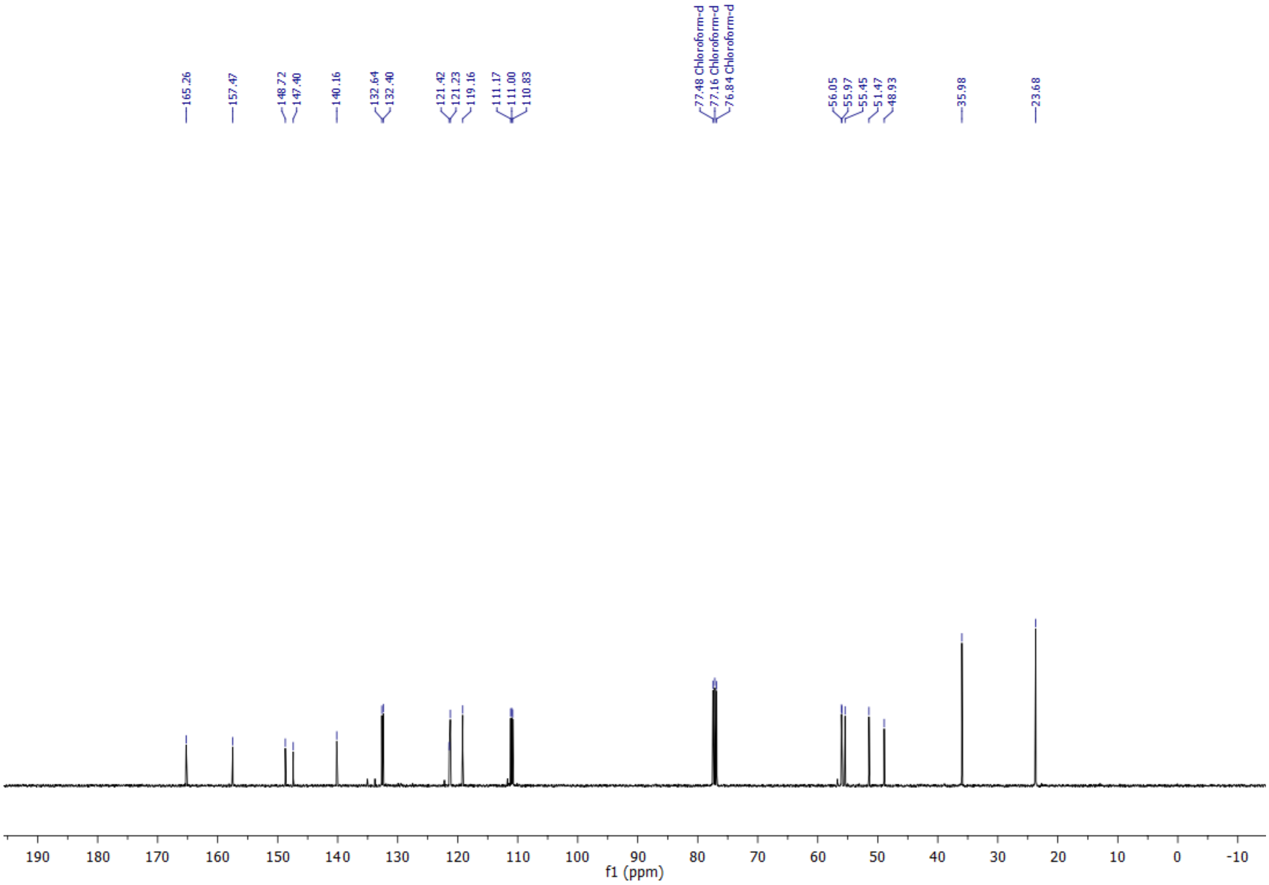

***N*-((1-(Benzo[*d*][1,3]dioxol-5-yl)cyclopentyl)methyl)-2-methoxybenzamide (9)**

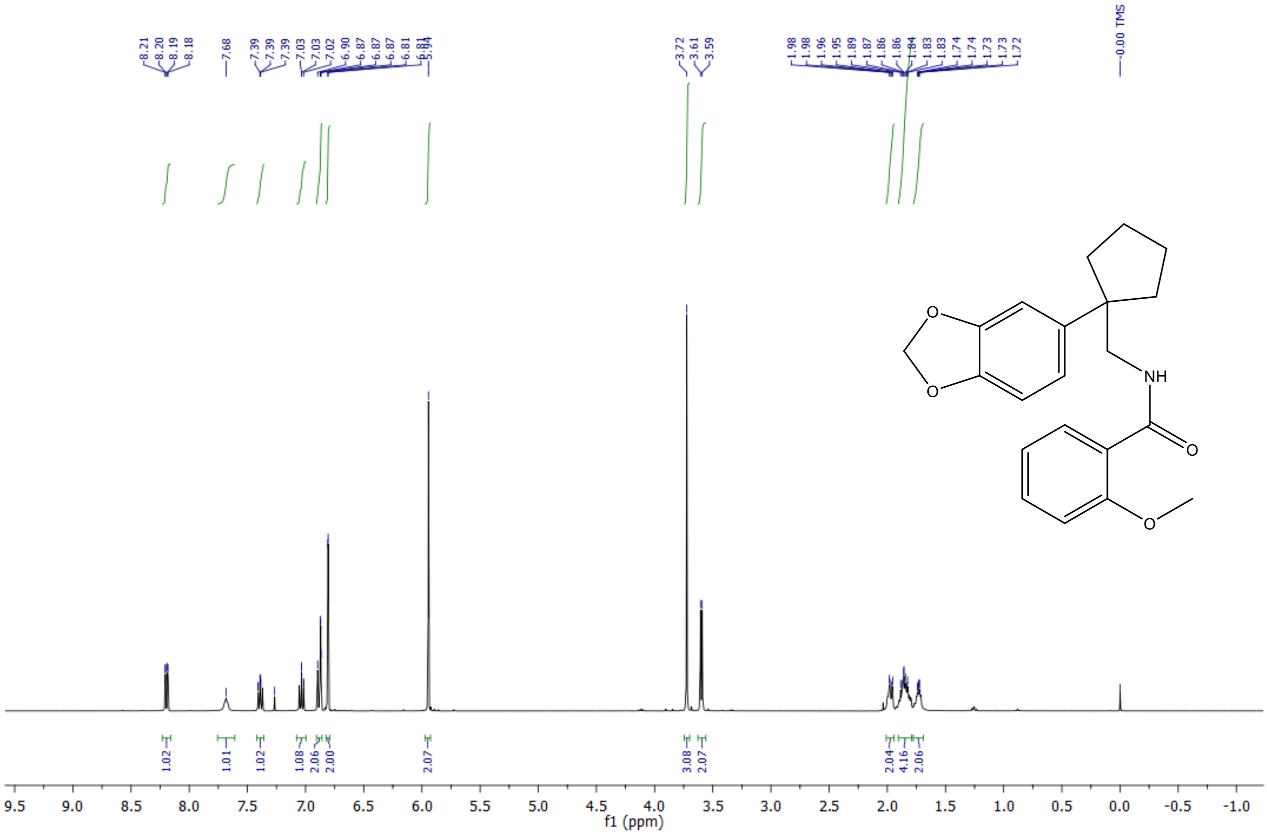

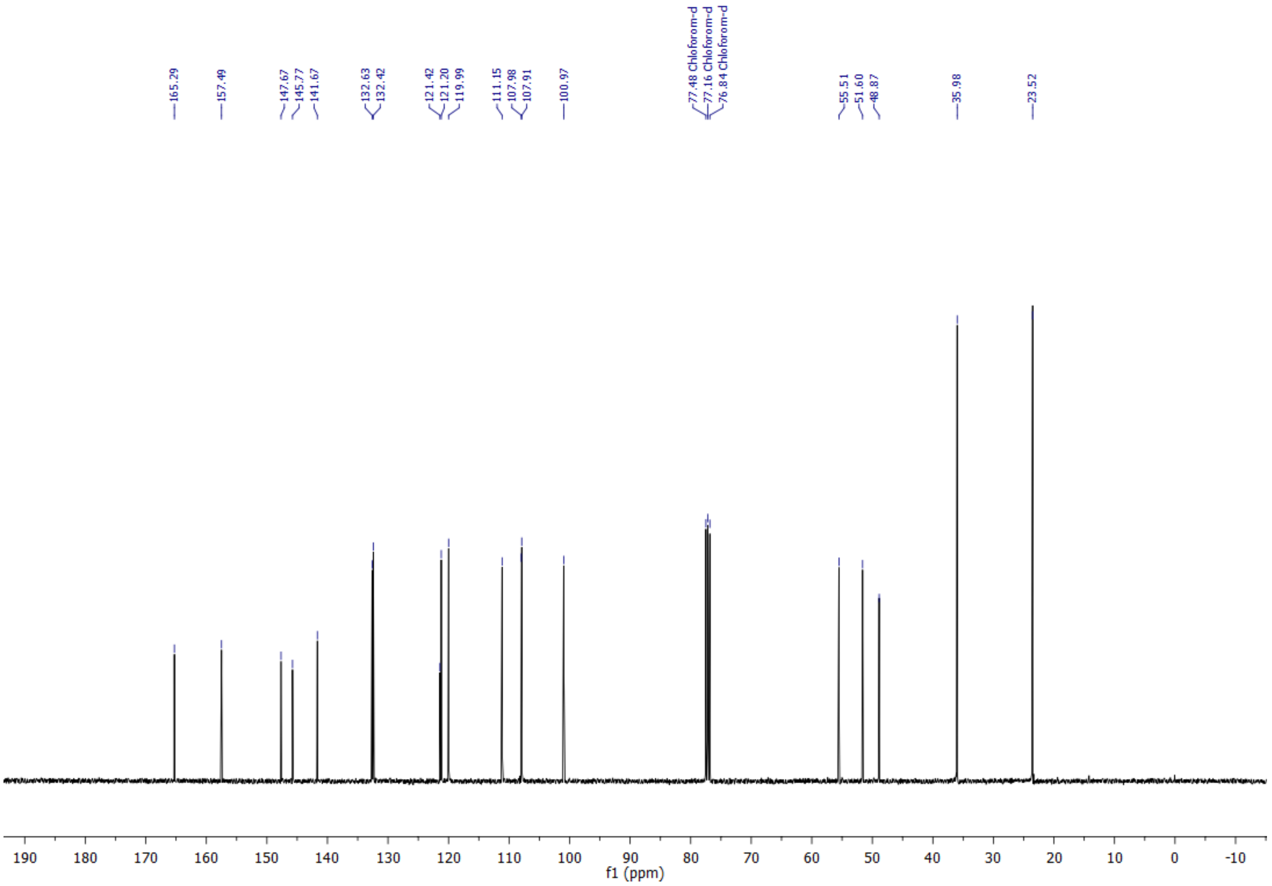

2-Methoxy-*N*-((1-phenylcyclopentyl)methyl)benzamide (10)

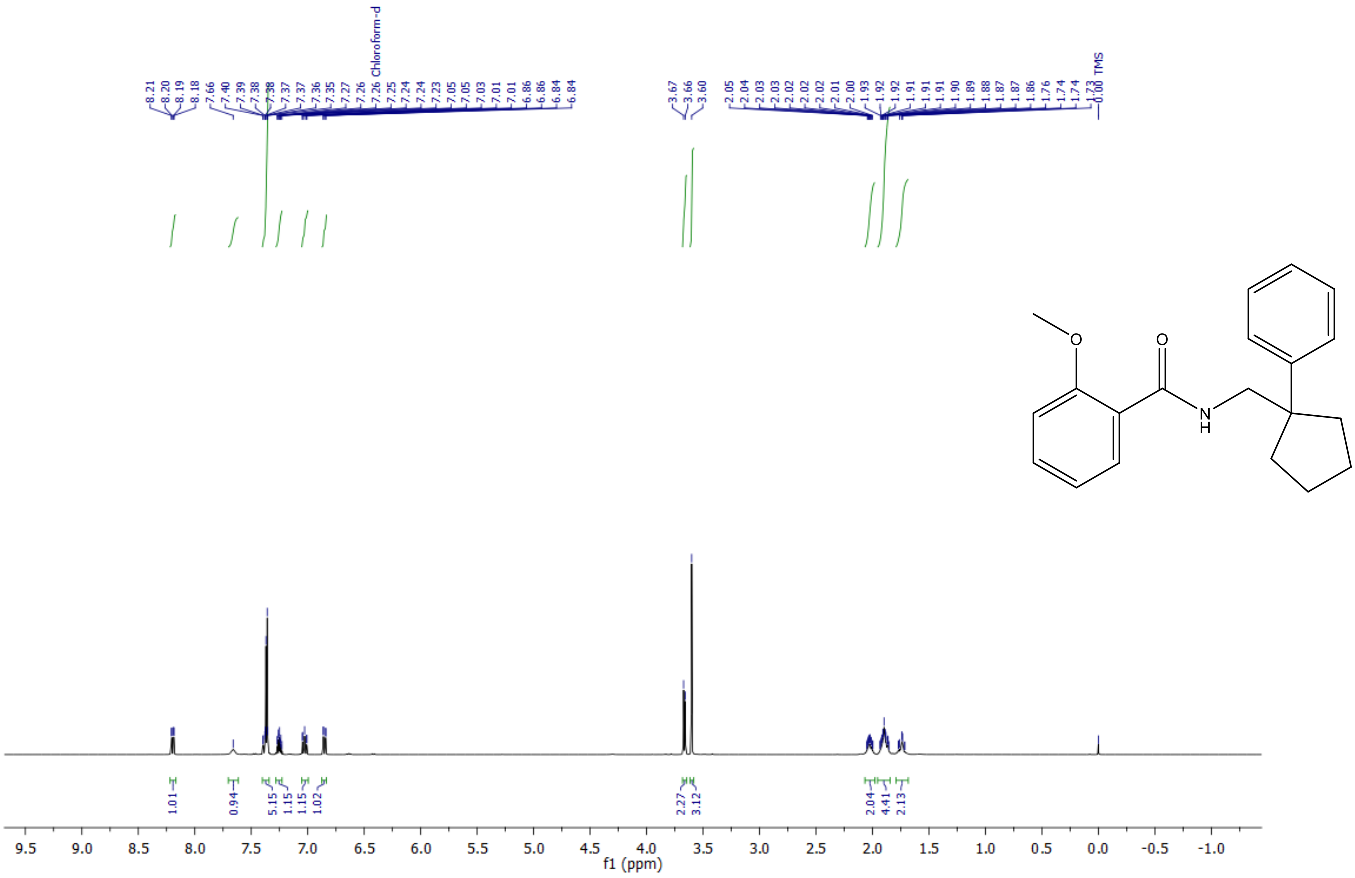

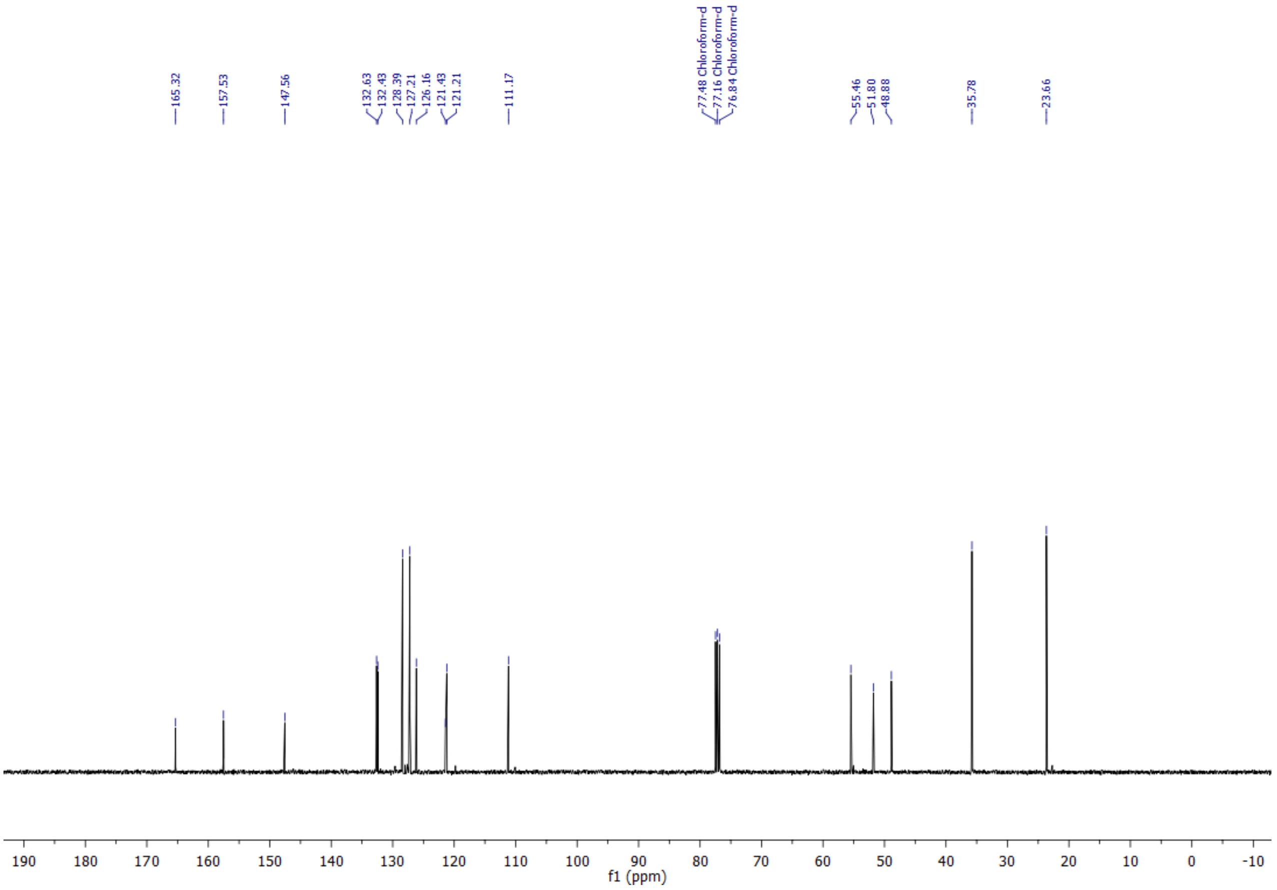

2-Methoxy-*N*-((1-(3-methoxyphenyl)cyclopentyl)methyl)benzamide (11)

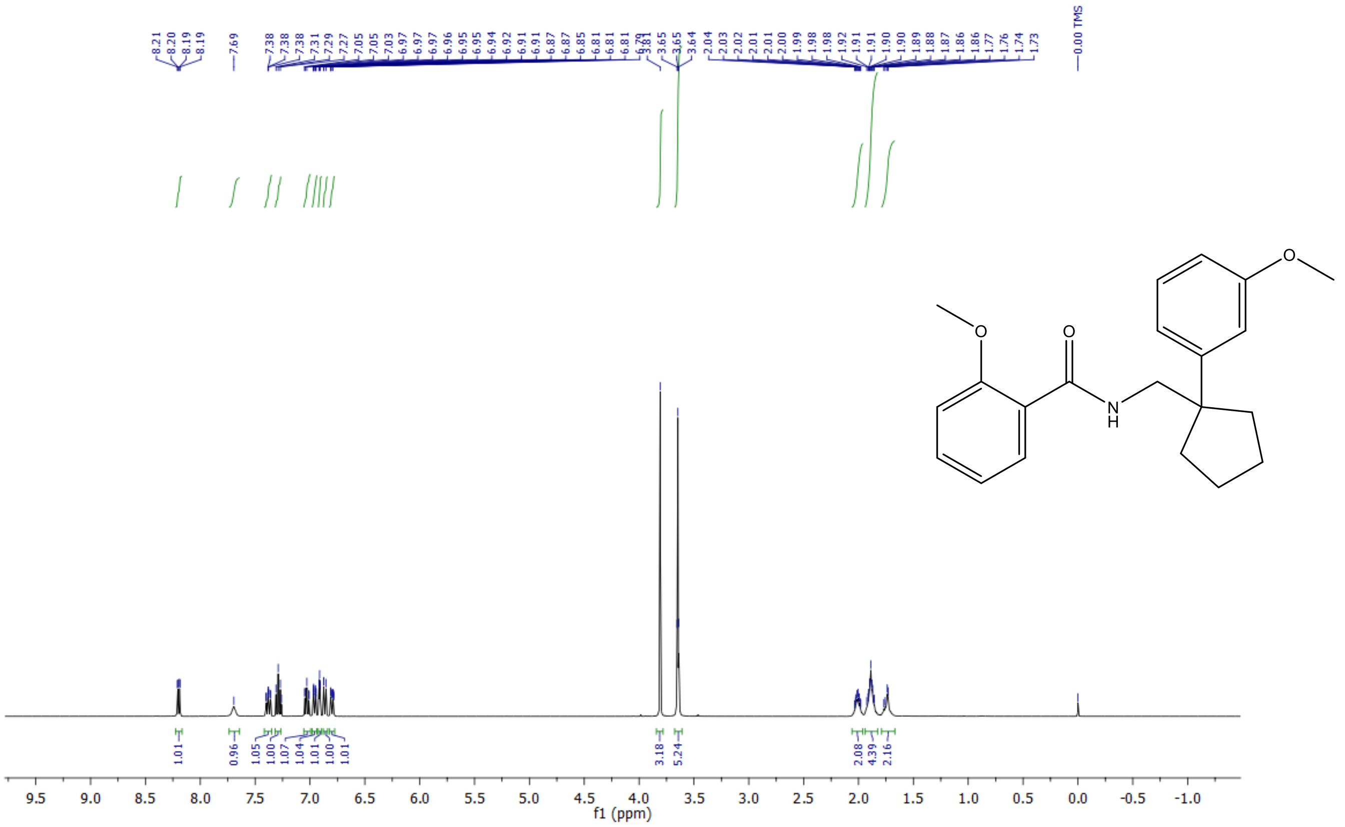

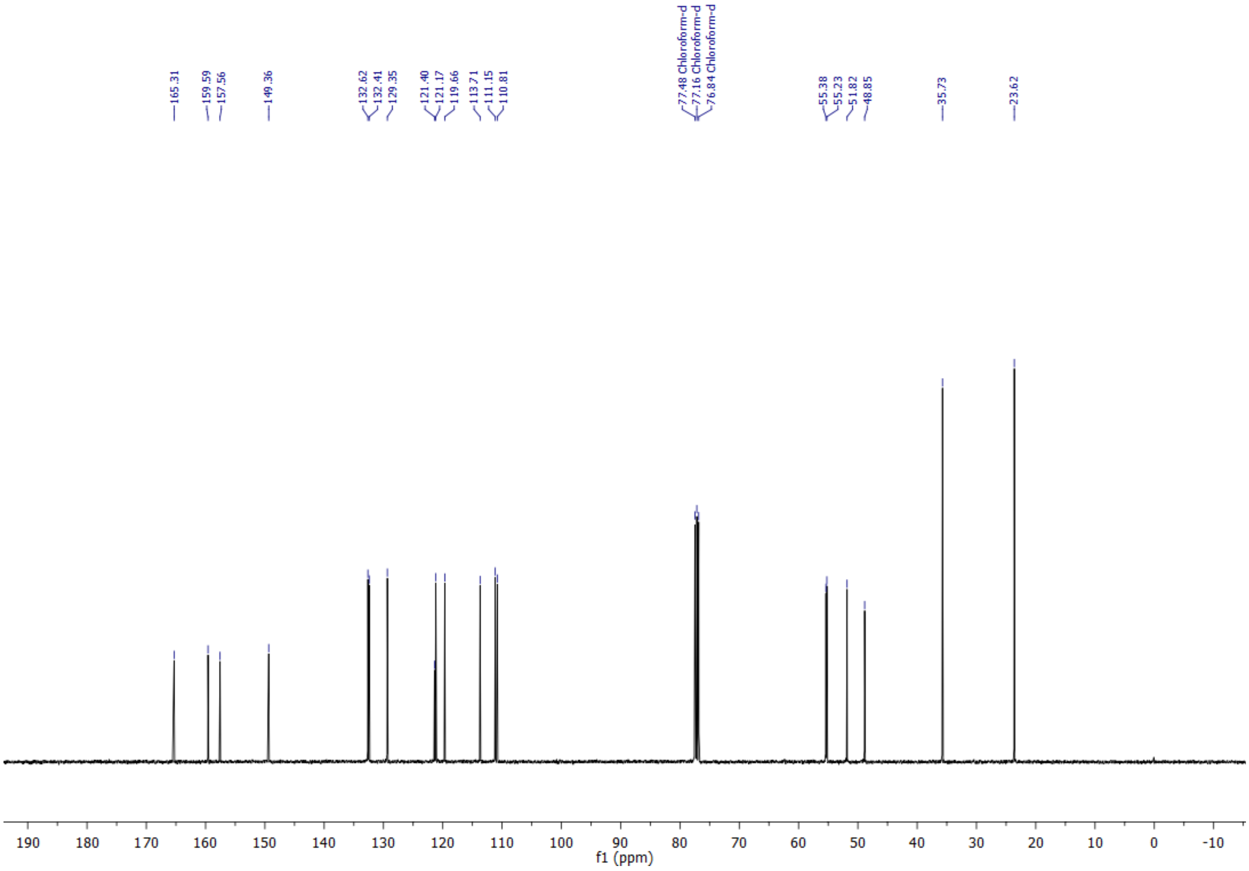

2-Methoxy-*N*-((1-(*m*-tolyl)cyclopentyl)methyl)benzamide (12)

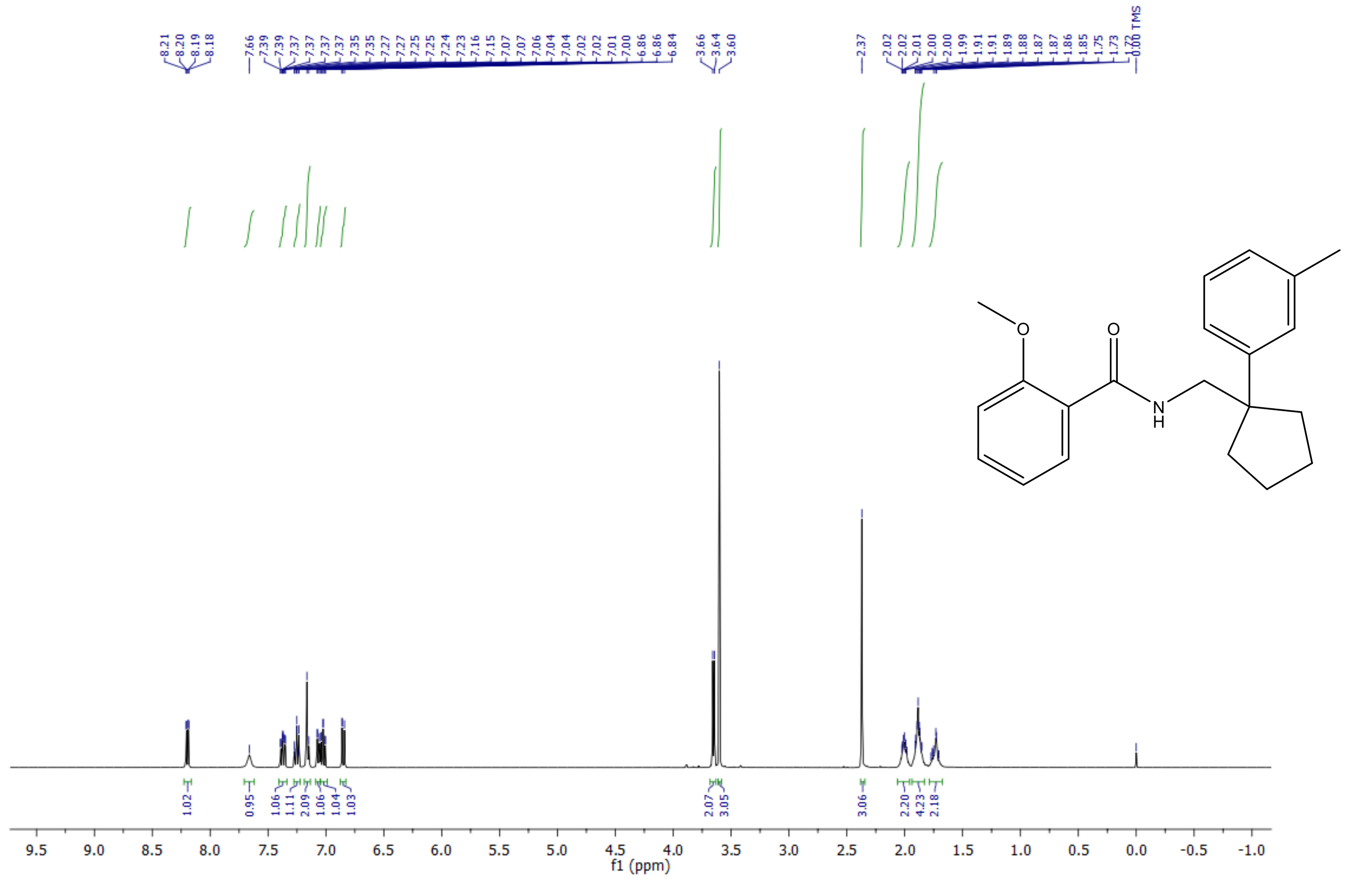

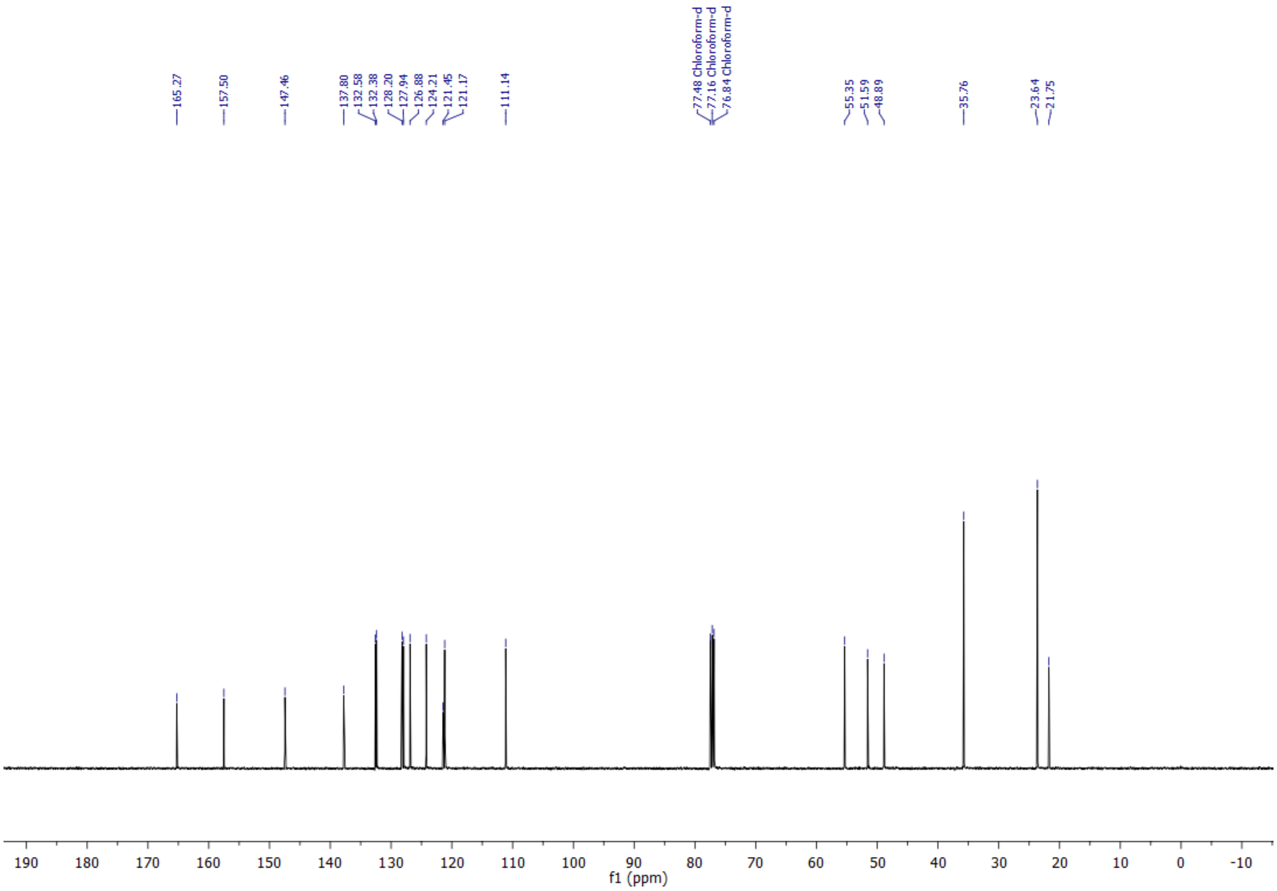

2-Methoxy-*N*-((1-(thiophen-2-yl)cyclopentyl)methyl)benzamide (13)

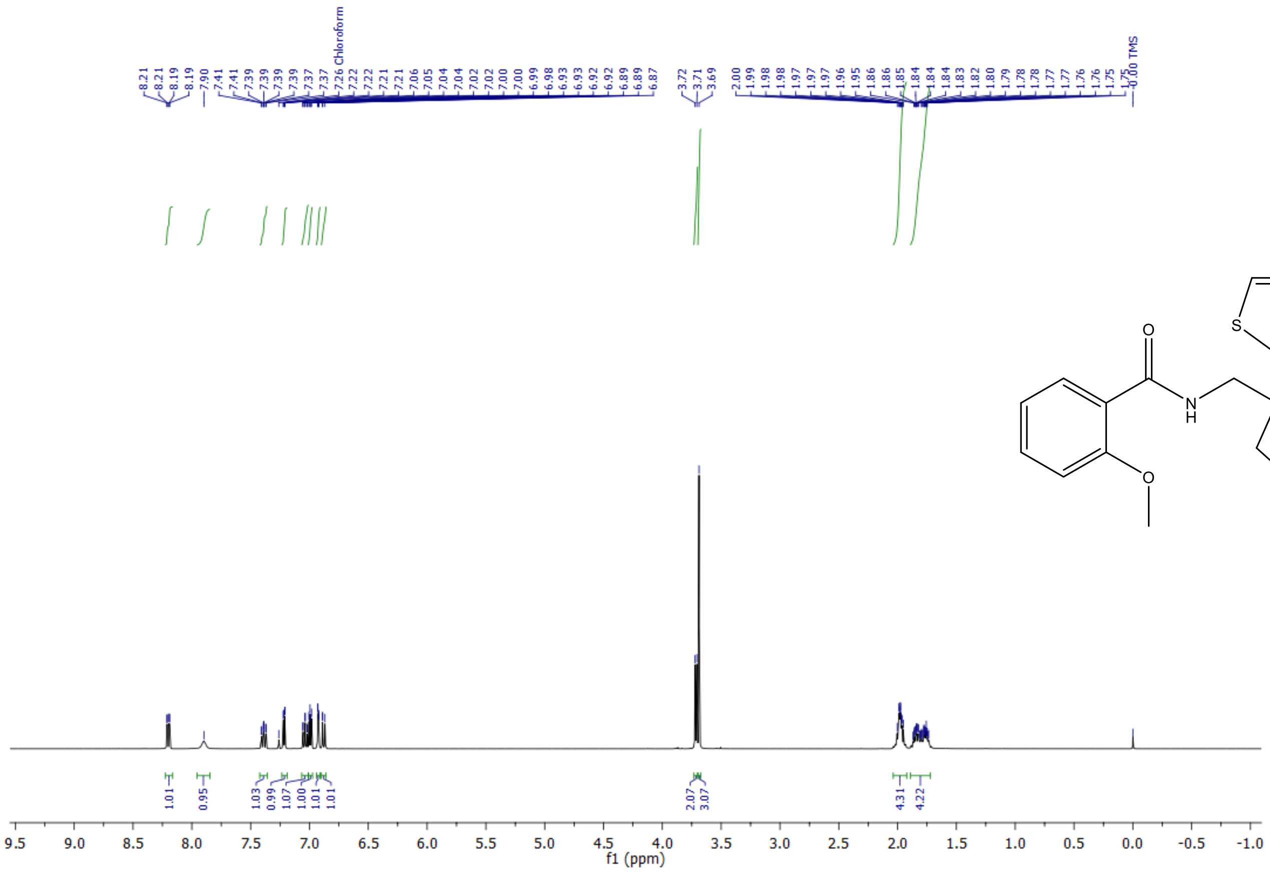
8.21
8.21
8.19
8.19
7.90
7.41
7.41
7.39
7.39
7.39
7.39
7.37
7.37
7.26 Chloroform
7.22
7.22
7.21
7.21
7.06
7.05
7.04
7.04
7.02
7.02
7.00
7.00
6.99
6.98
6.93
6.93
6.92
6.92
6.89
6.89
6.87
3.72
3.71
3.69
2.00
1.99
1.98
1.98
1.97
1.97
1.97
1.96
1.95
1.86
1.86
1.85
1.84
1.84
1.84
1.83
1.82
1.80
1.79
1.78
1.78
1.77
1.77
1.76
1.76
1.75
1.75
0.00 TMS
O
N
H
S
O
1.01
0.95
1.03
0.99
1.07
1.00
1.01
1.01
2.07
3.07
4.31
4.22
f1 (ppm)

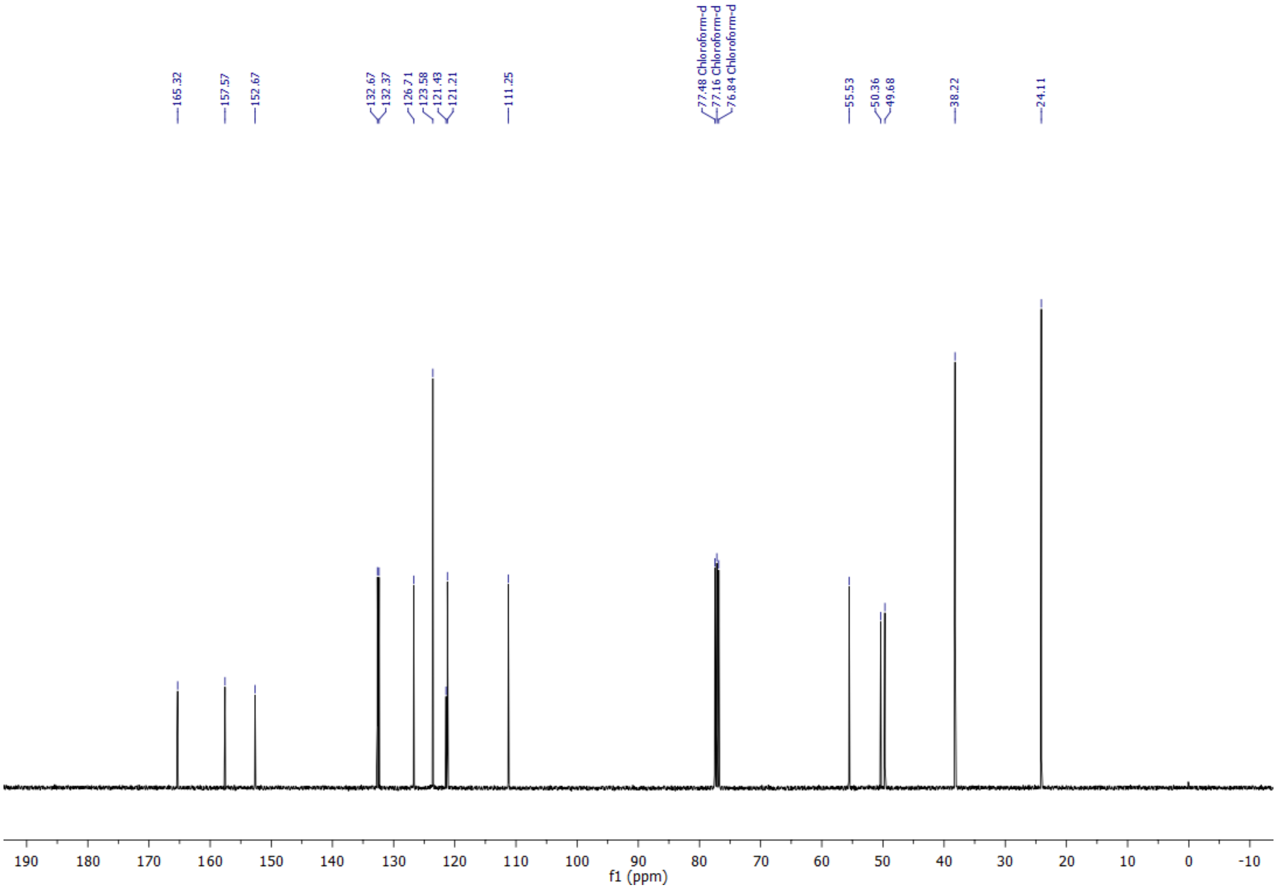
165.32
157.57
152.67
132.67
132.37
126.71
123.58
121.43
121.21
111.25
77.48 Chloroform-d
77.16 Chloroform-d
76.84 Chloroform-d
55.53
50.36
49.68
38.22
24.11
f1 (ppm)

2-Methoxy-*N*-((1-(thiophen-3-yl)cyclopentyl)methyl)benzamide (4)

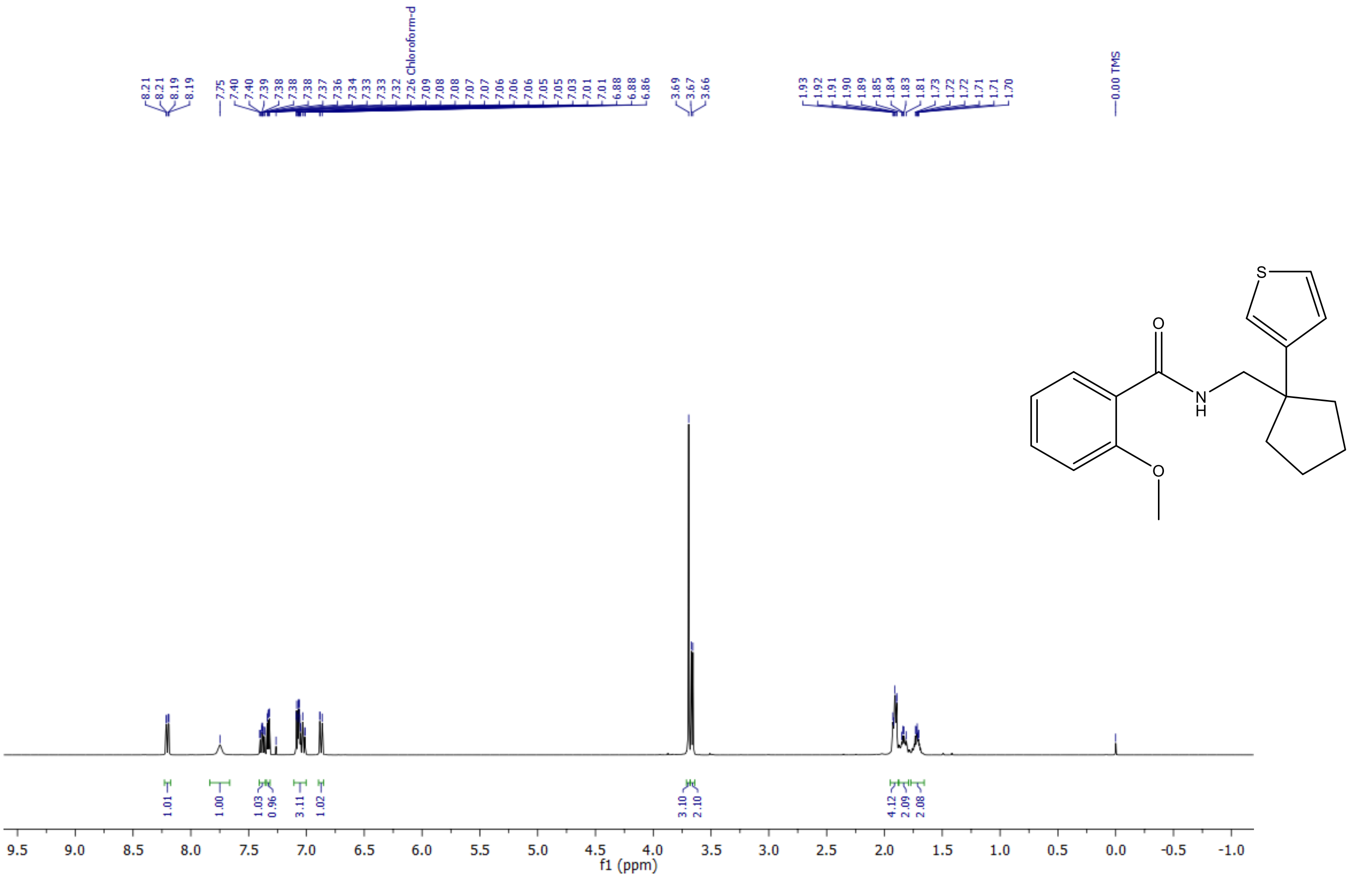

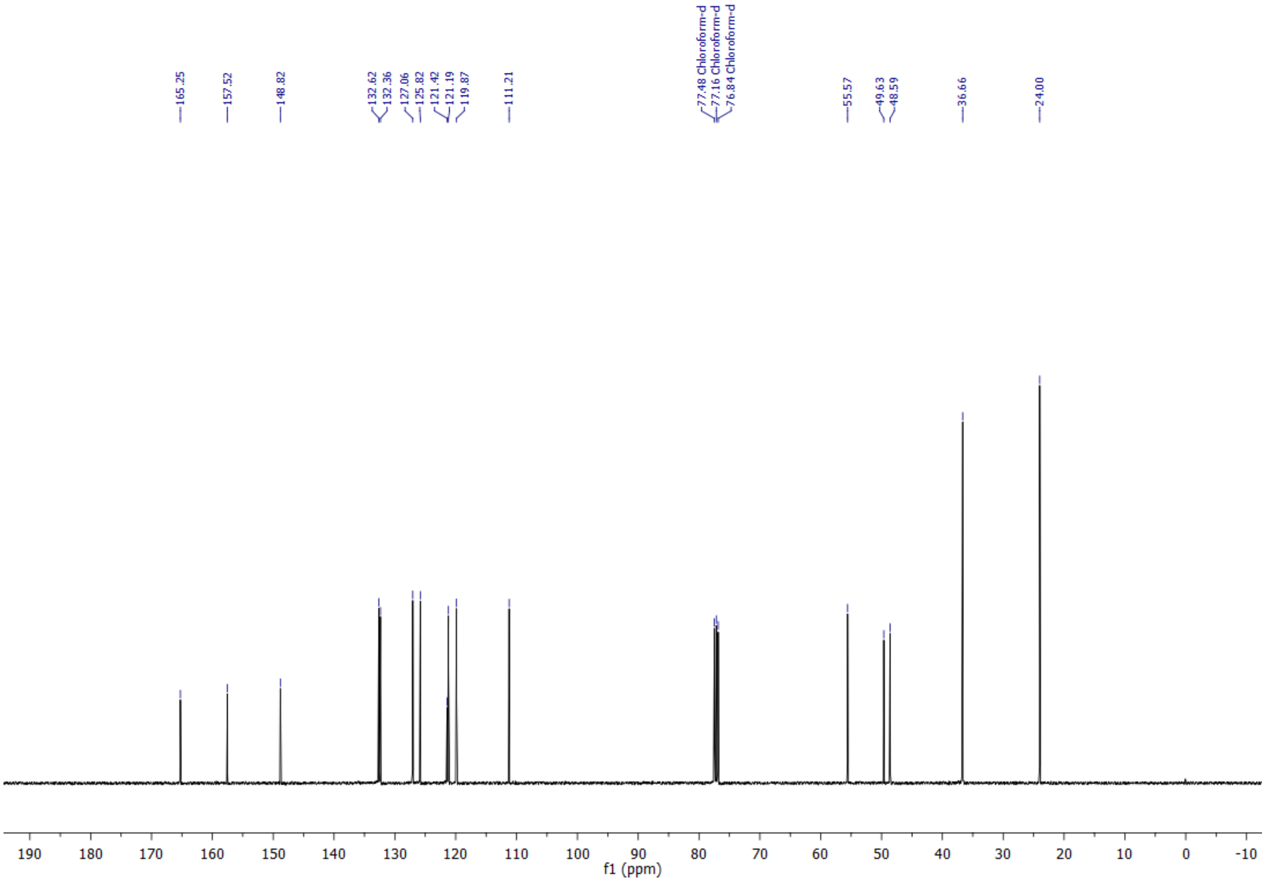

2-Methoxy-*N*-((3-(thiophen-2-yl)tetrahydrofuran-3-yl)methyl)benzamide (14)

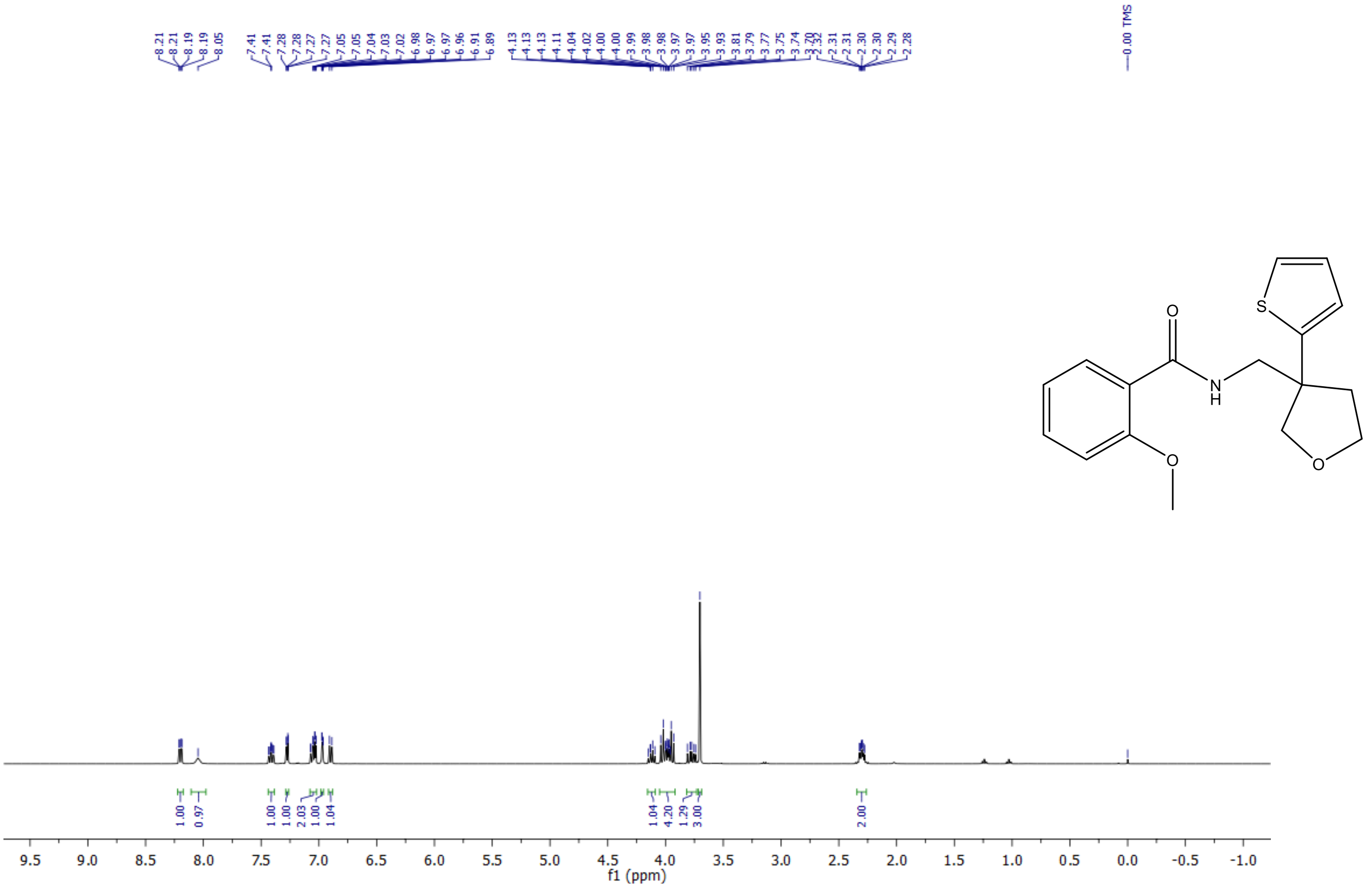

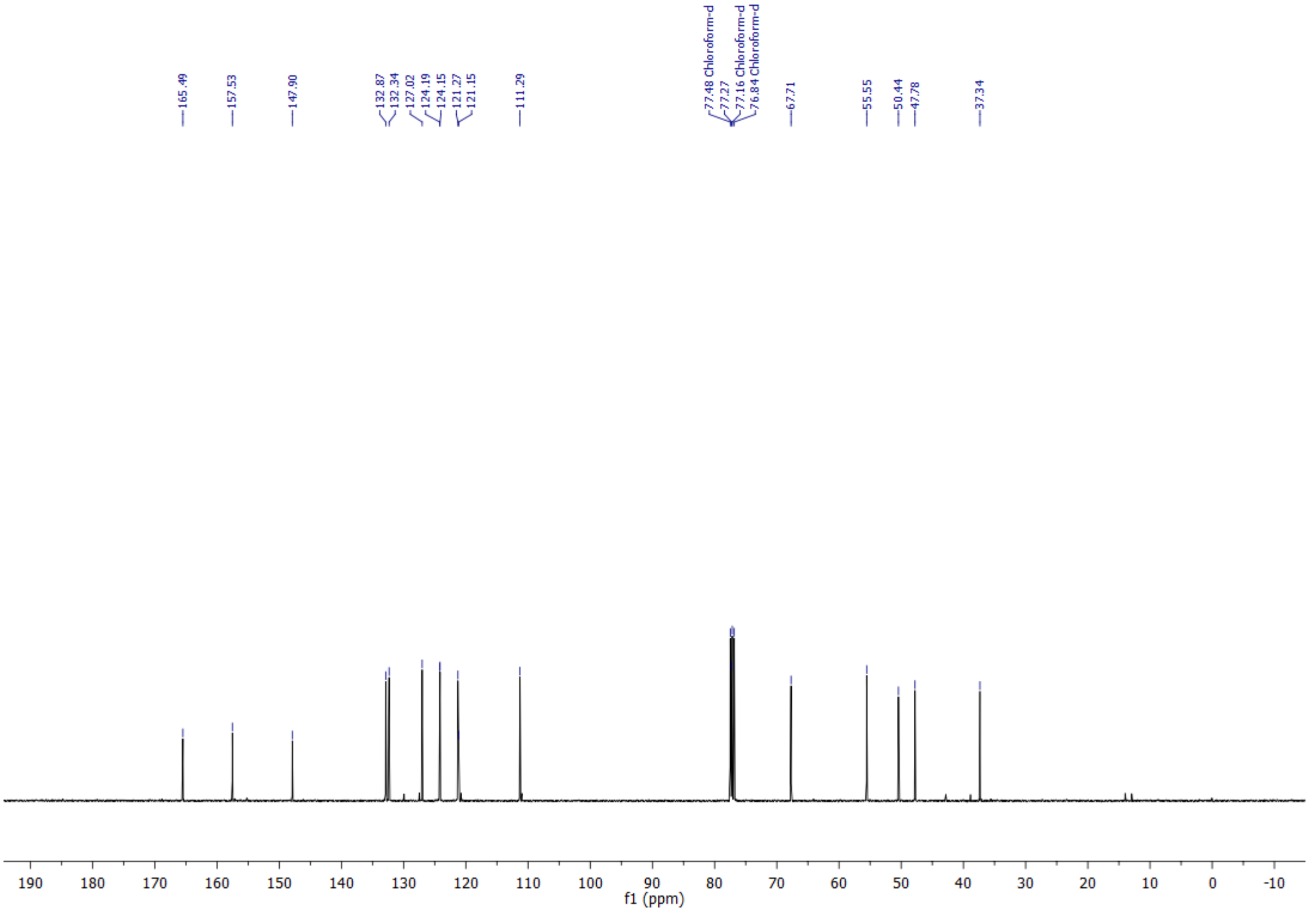

1-(4-Methoxyphenyl)cyclopentane-1-carbonitrile (52)

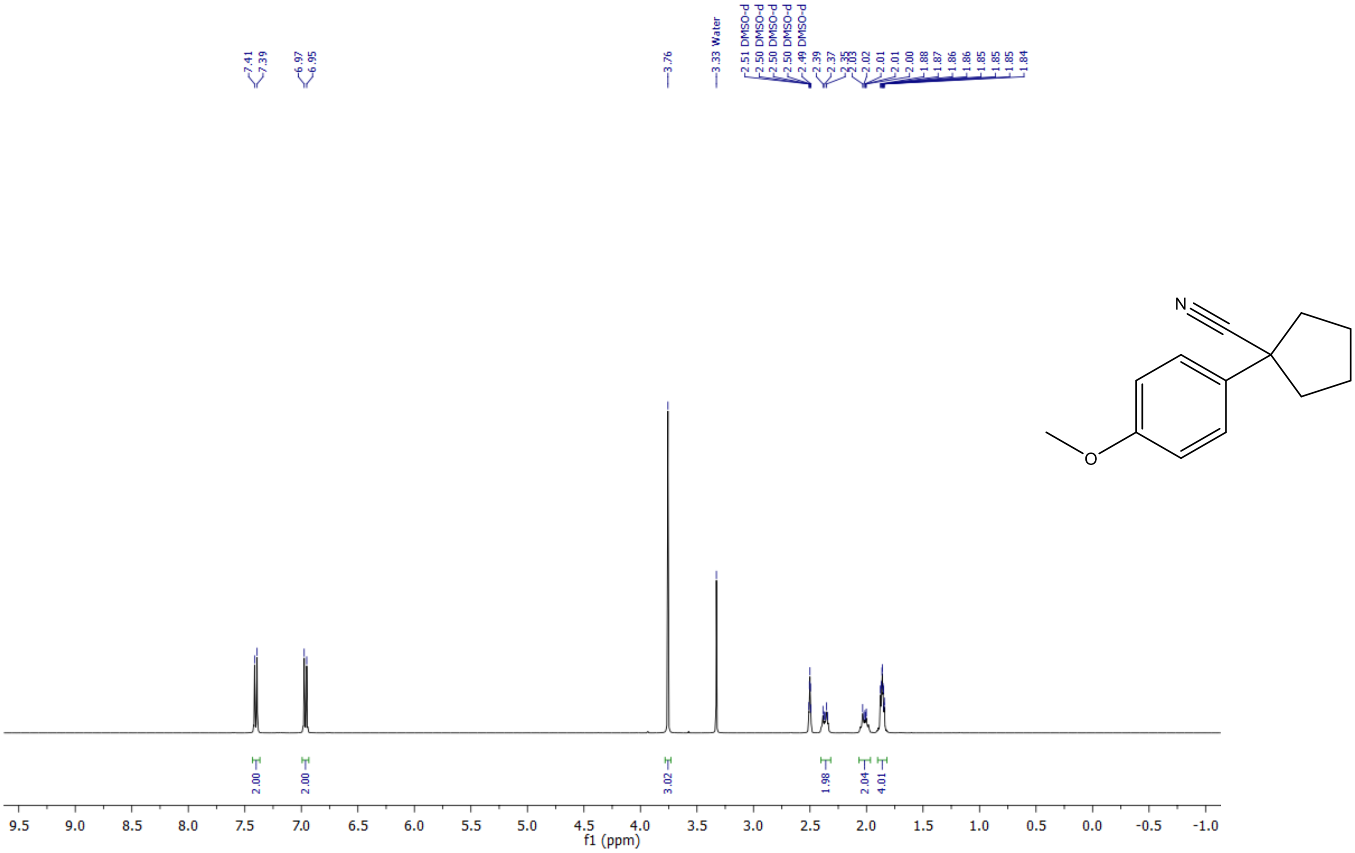

1-(2-Methoxyphenyl)cyclopentane-1-carbonitrile (53)

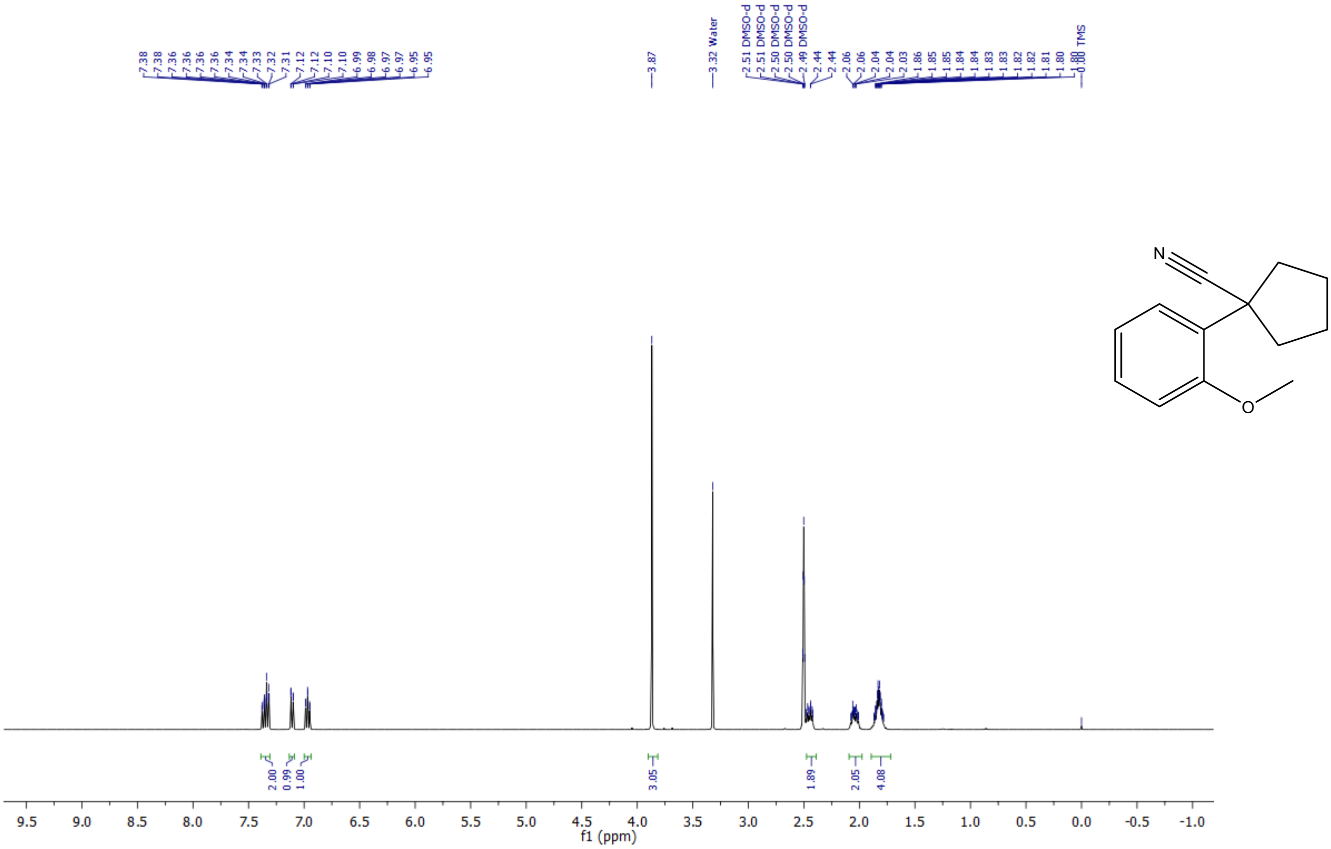

1-(3,4-Dimethoxyphenyl)cyclopentane-1-carbonitrile (54)

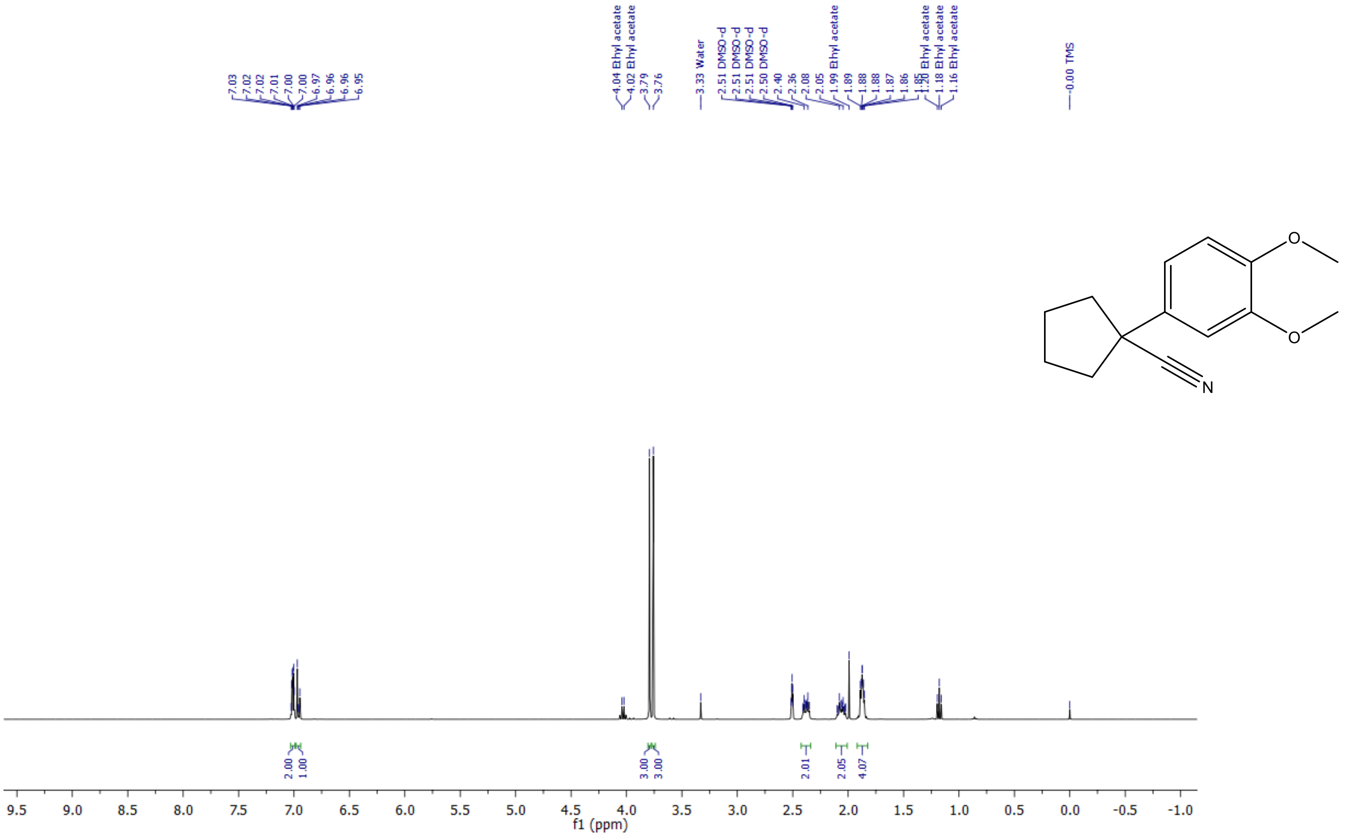

1-(Benzo[*d*][1,3]dioxol-5-yl)cyclopentane-1-carbonitrile (55)

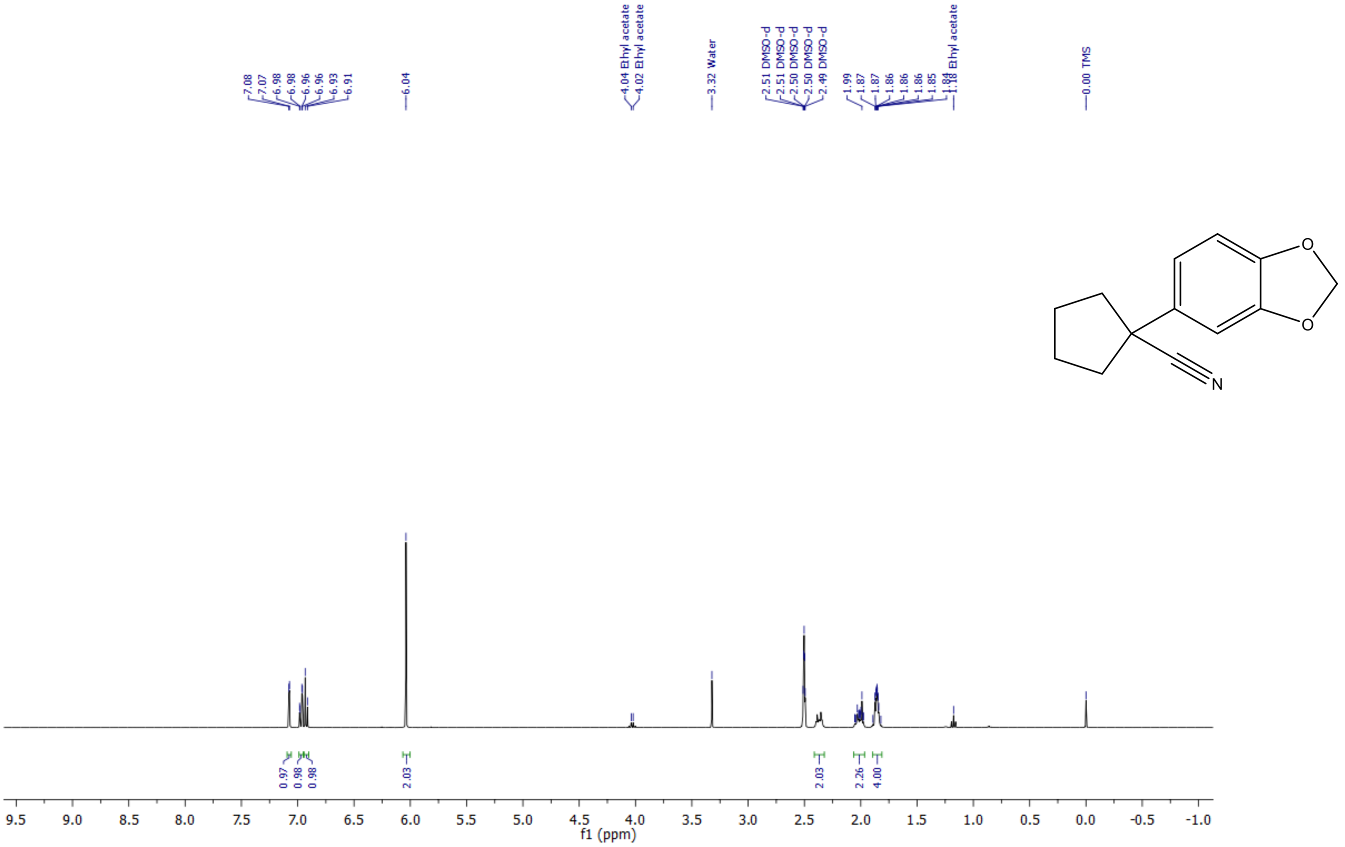

1-Phenylcyclopentane-1-carbonitrile (56)

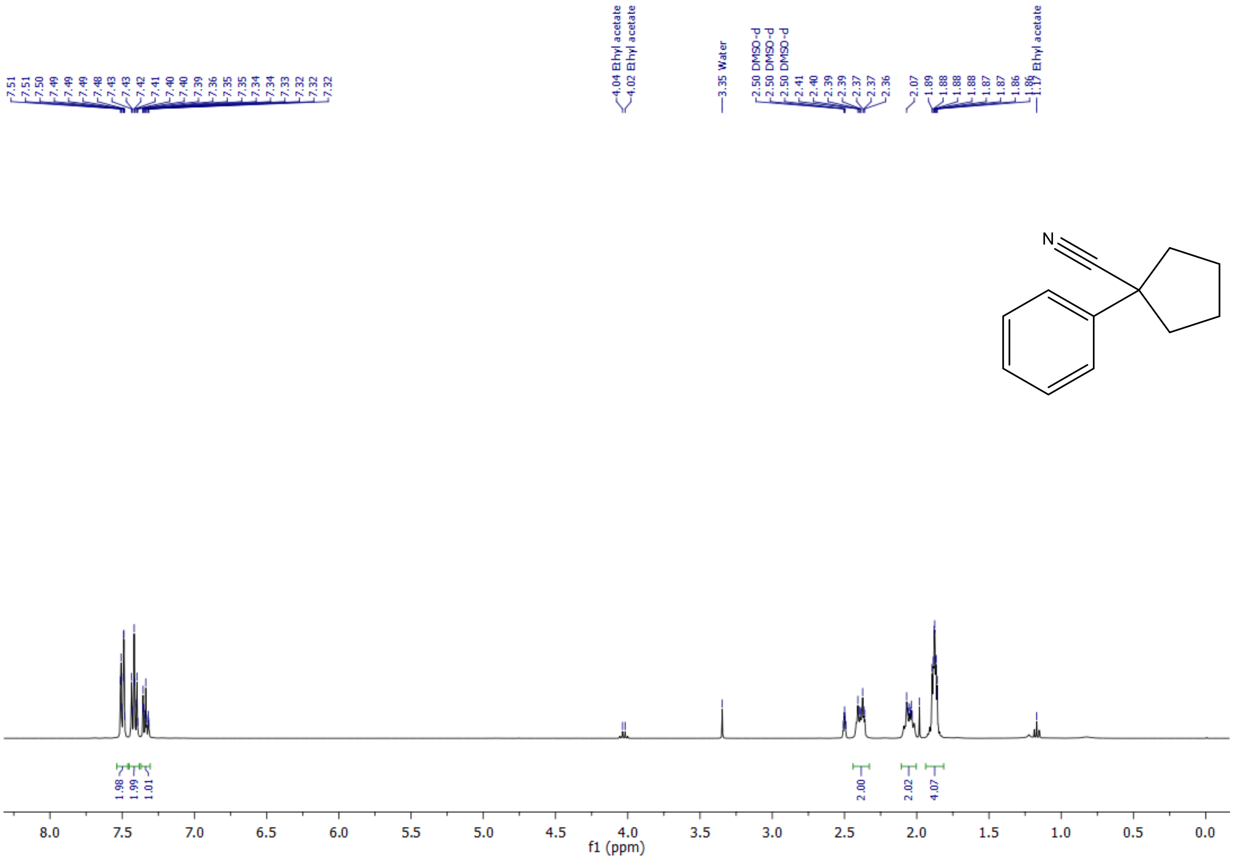

1-(3-Methoxyphenyl)cyclopentane-1-carbonitrile (57)

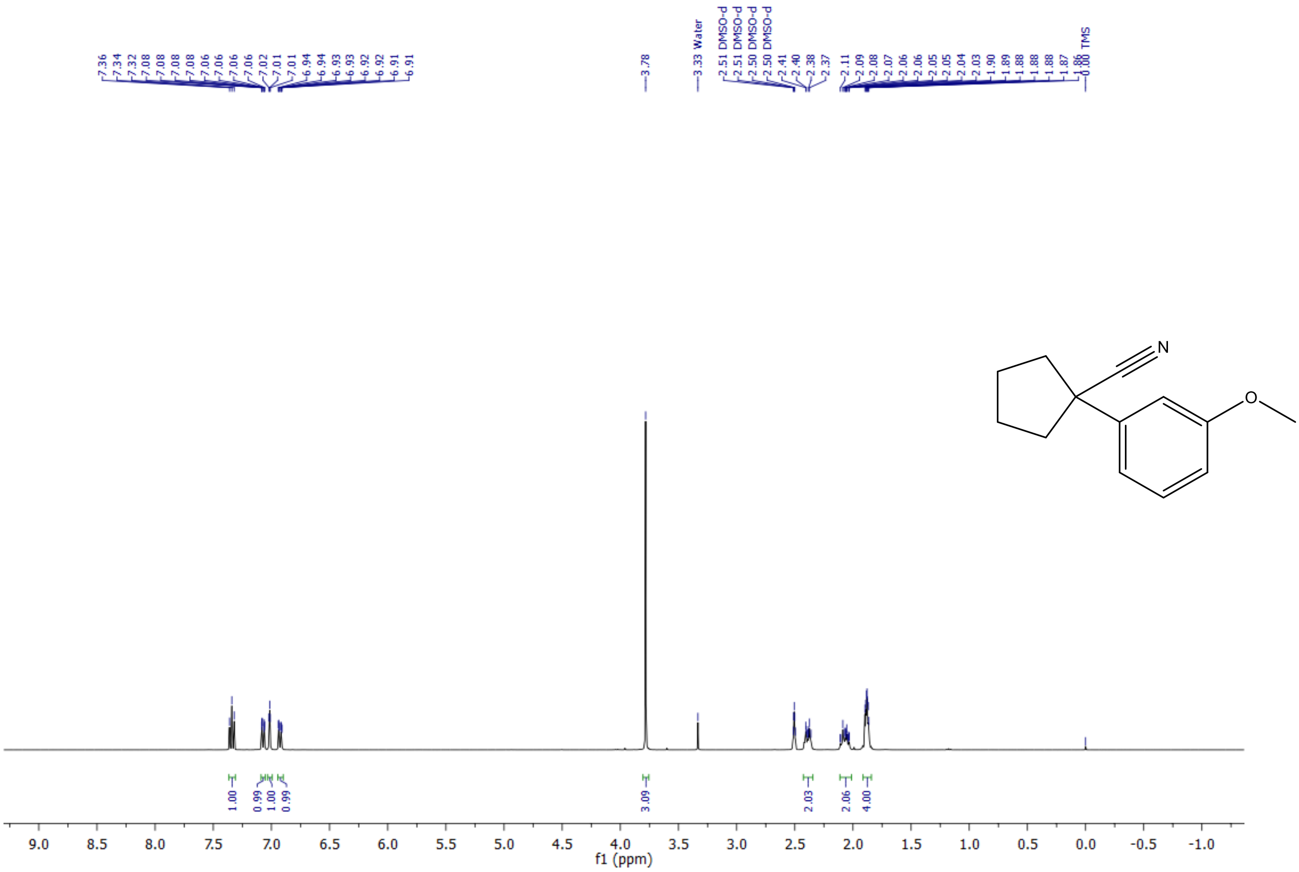

1-(*m*-Tolyl)cyclopentane-1-carbonitrile (58)

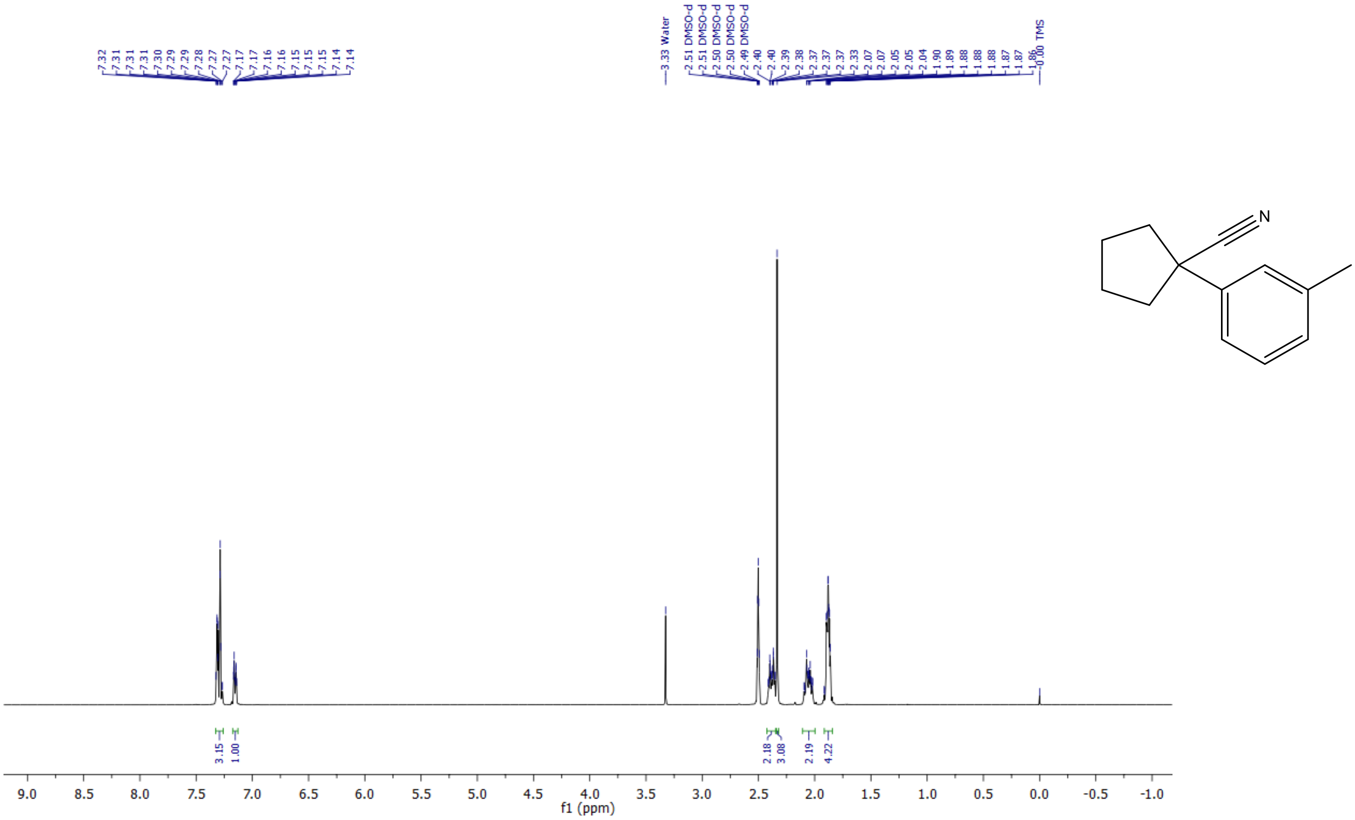

1-(Thiophen-2-yl)cyclopentane-1-carbonitrile (59)

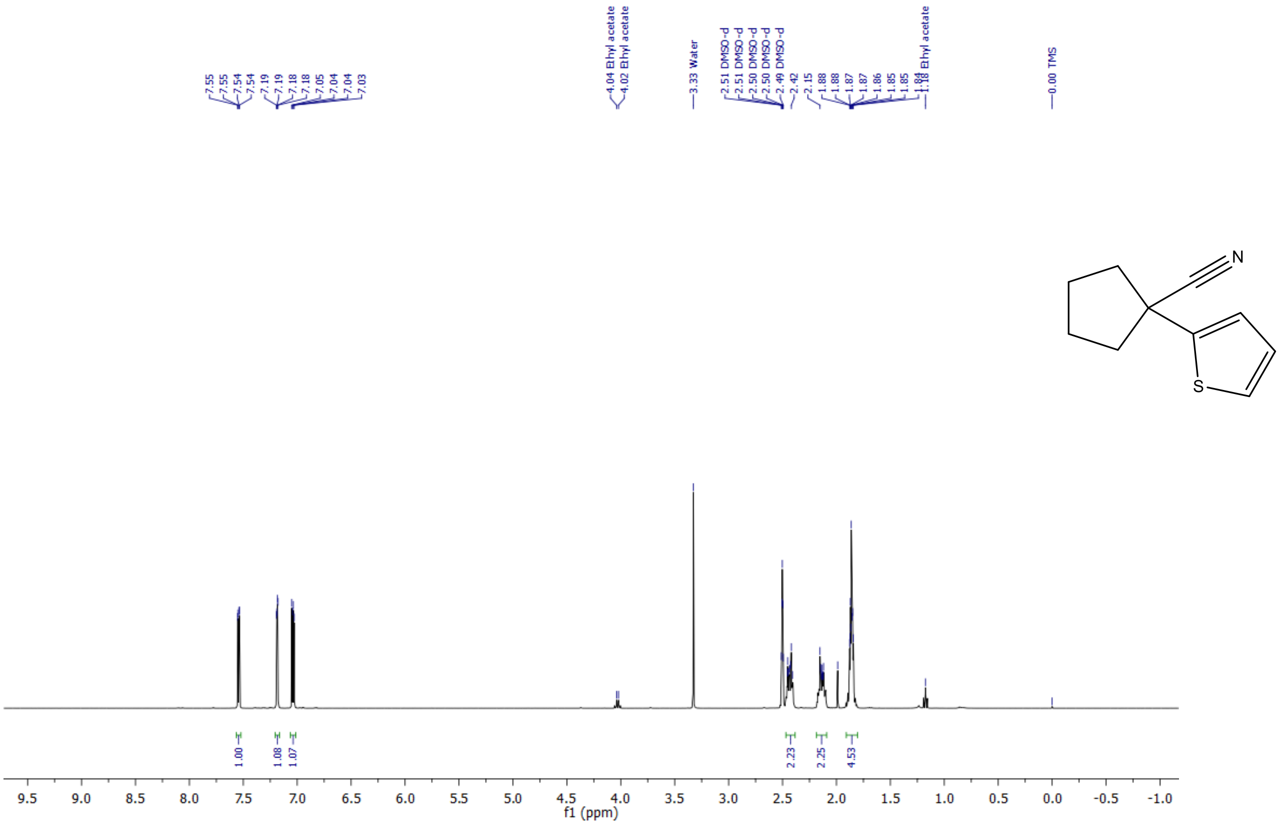

1-(Thiophen-3-yl)cyclopentane-1-carbonitrile (60)

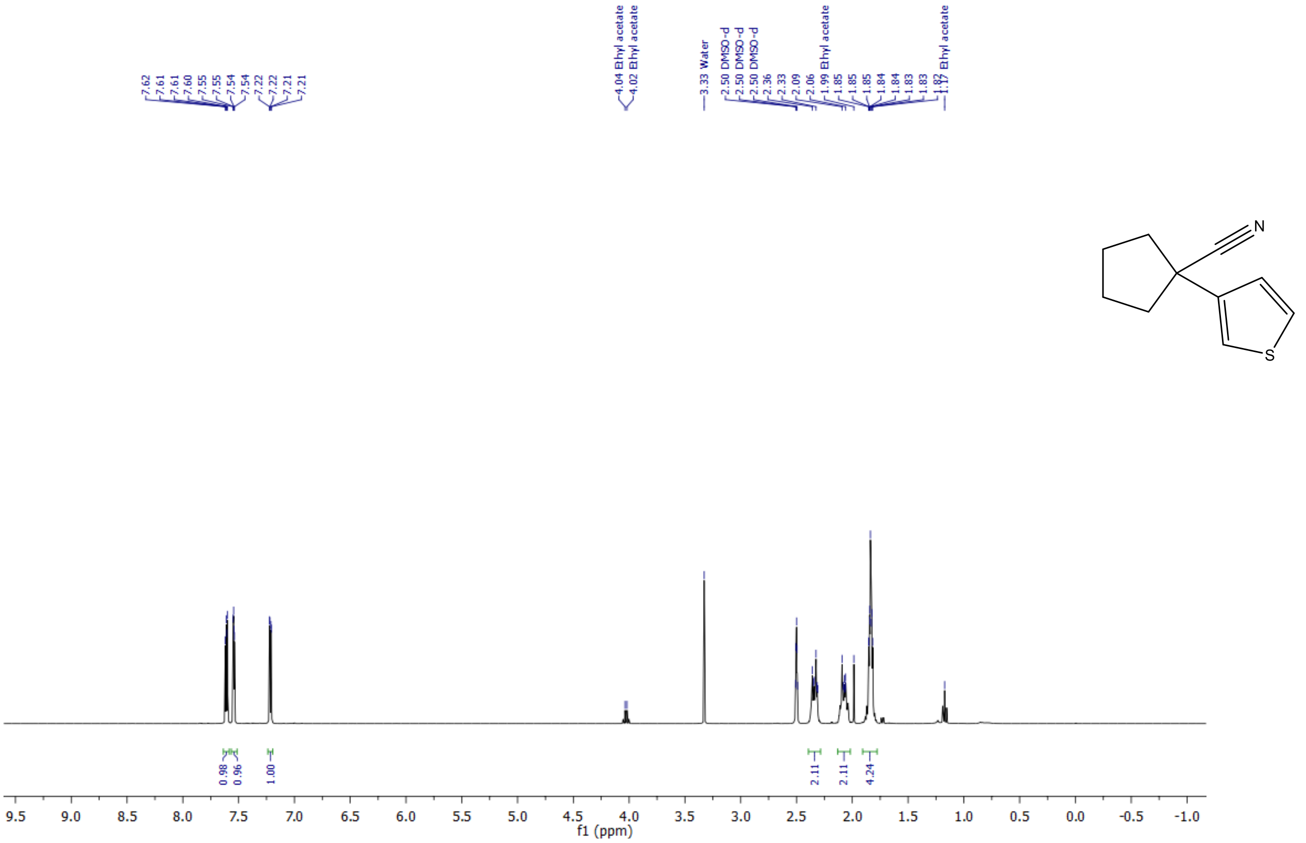

3-(Thiophen-2-yl)tetrahydrofuran-3-carbonitrile (61)

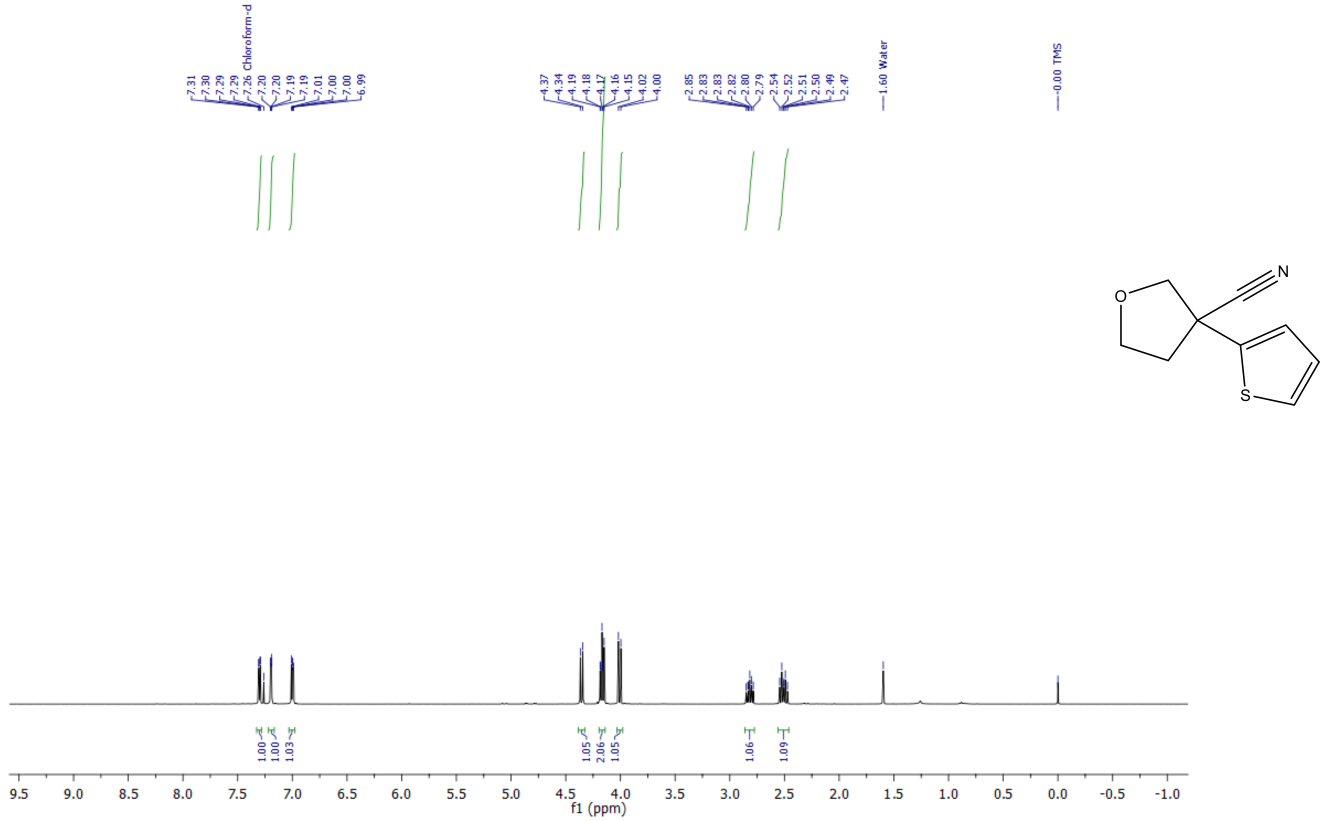

1-(4-Methoxyphenyl)cyclopentyl)methanamine (62)

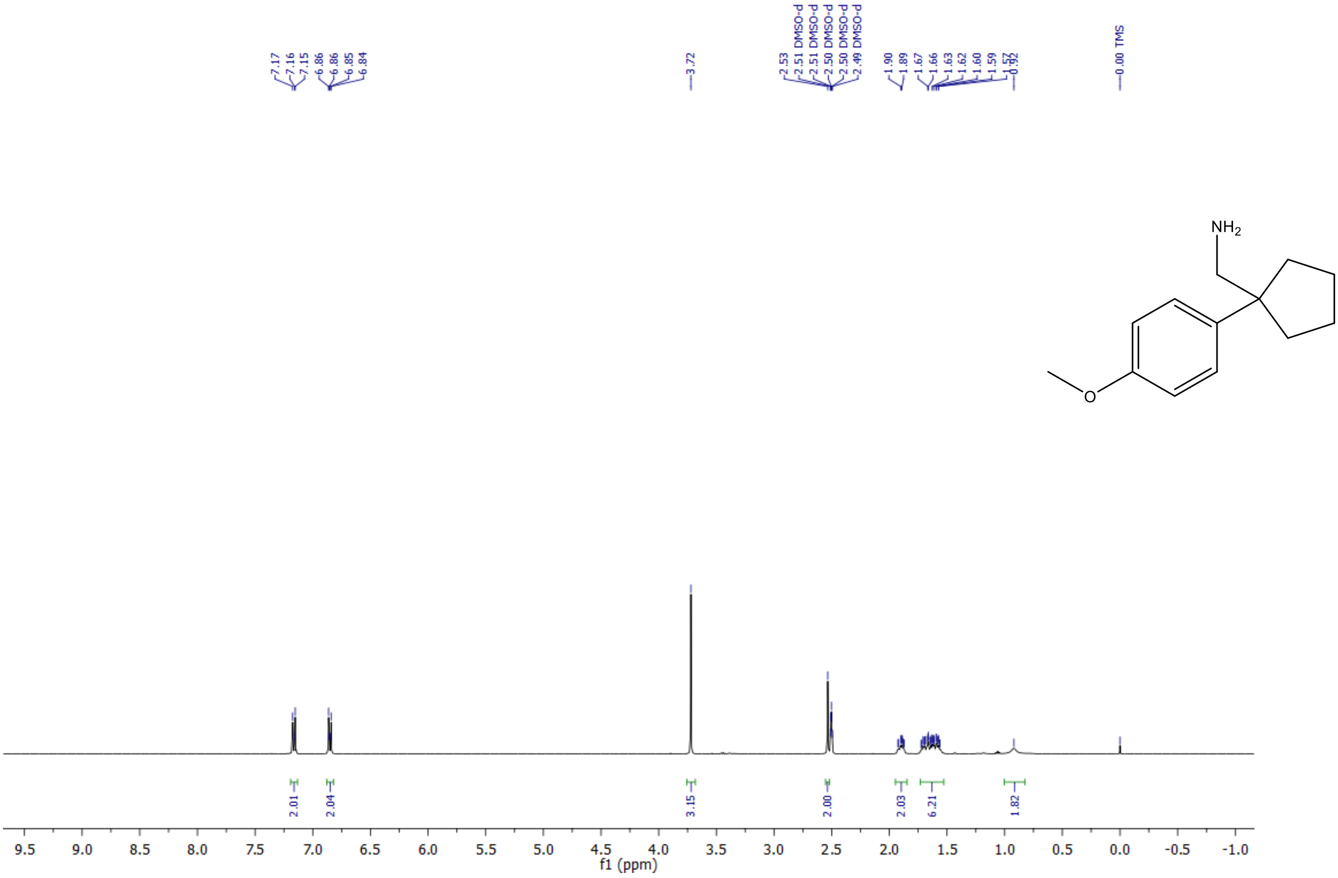

1-(2-Methoxyphenyl)cyclopentyl)methanamine (63)

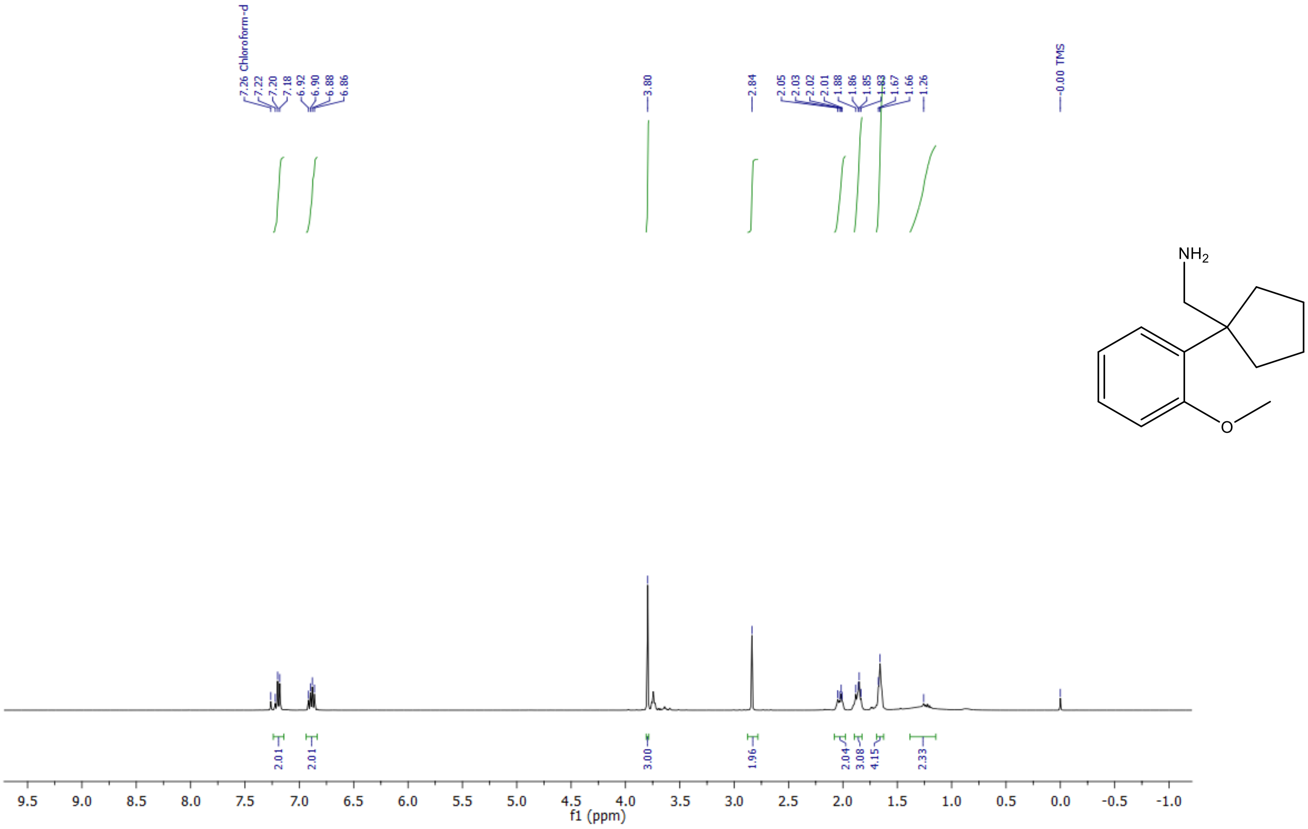

1-(3,4-Dimethoxyphenyl)cyclopentyl)methanamine (64)

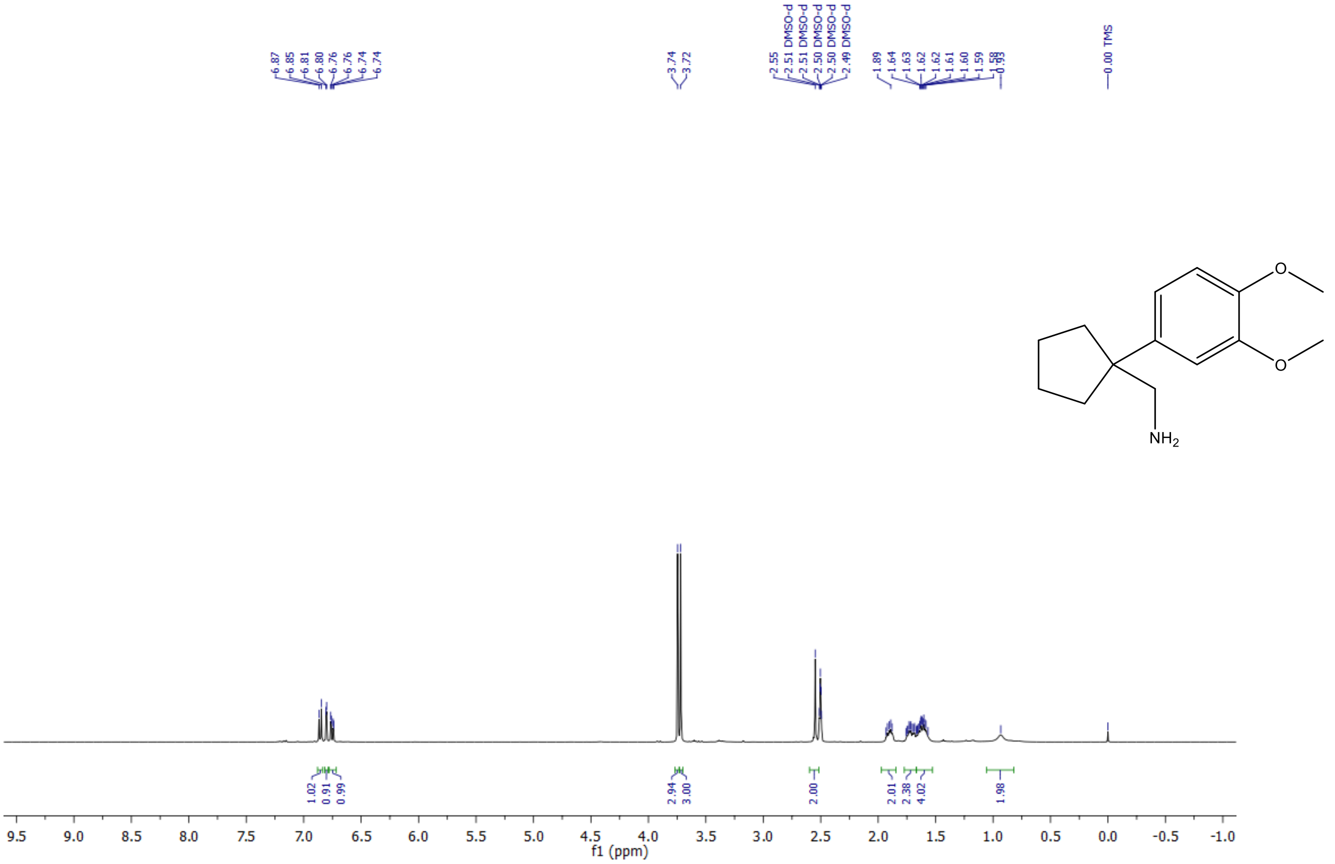

1-(Benzo[*d*][1,3]dioxol-5-yl)cyclopentyl)methanamine (65)

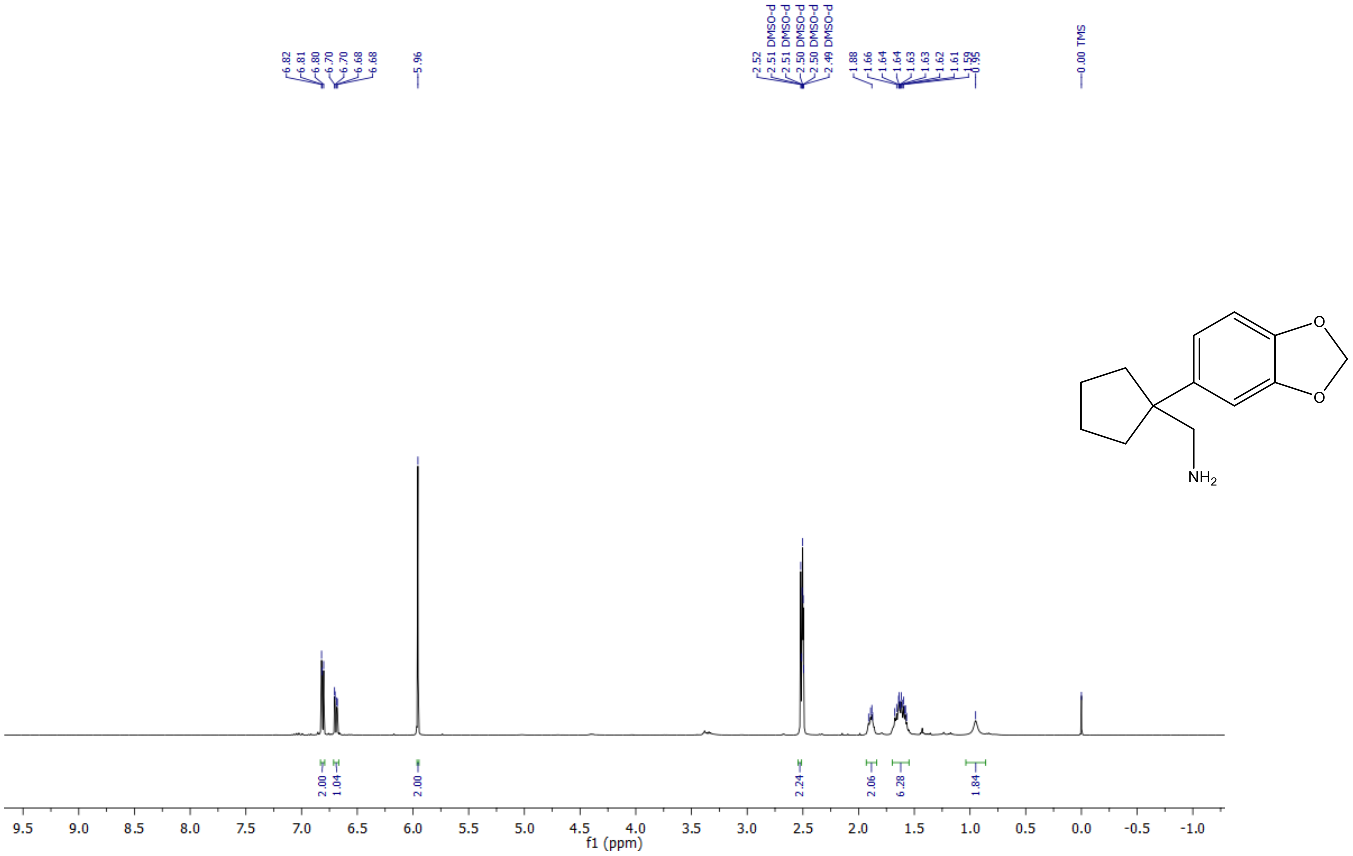

1-Phenylcyclopentyl)methanamine (66)

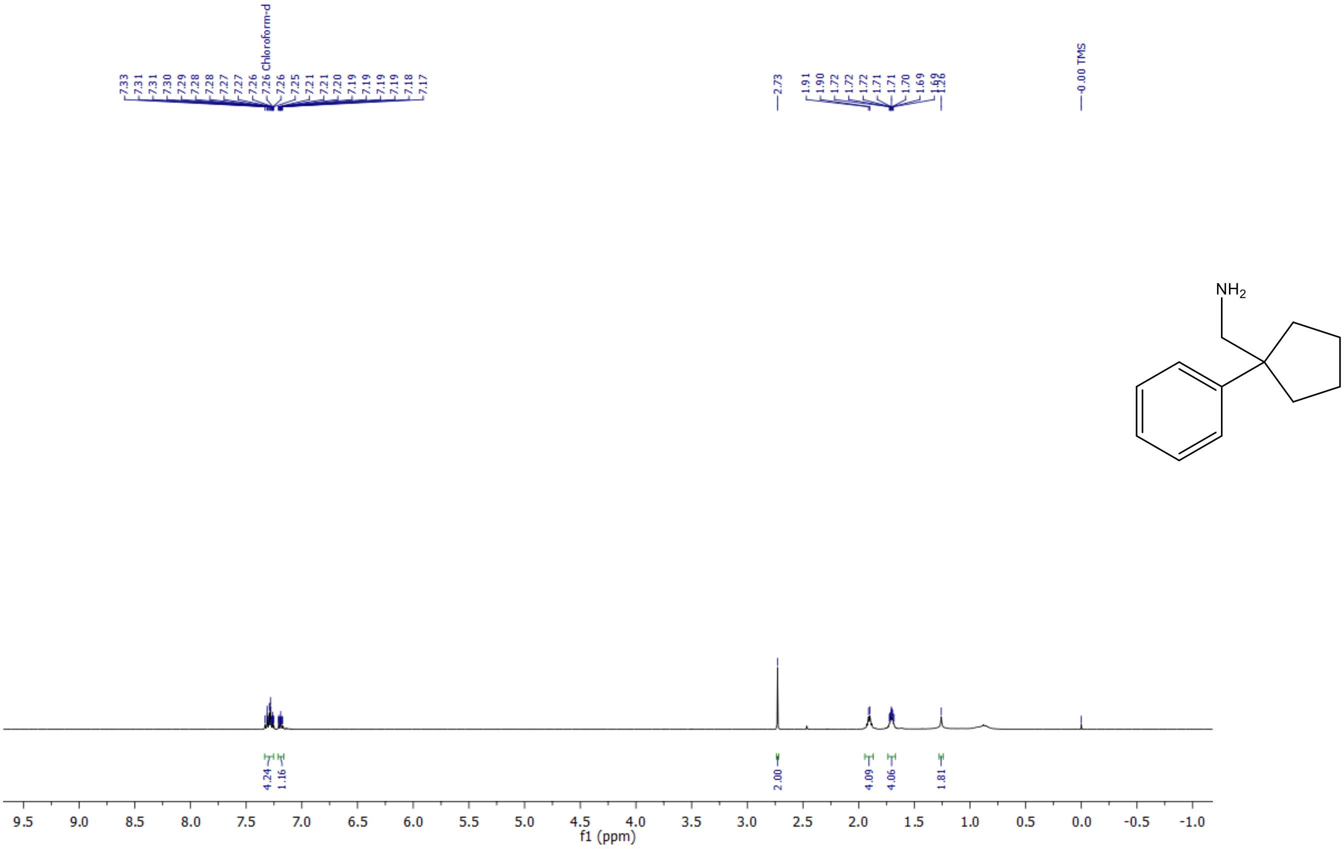

1-(3-Methoxyphenyl)cyclopentyl)methanamine (67)

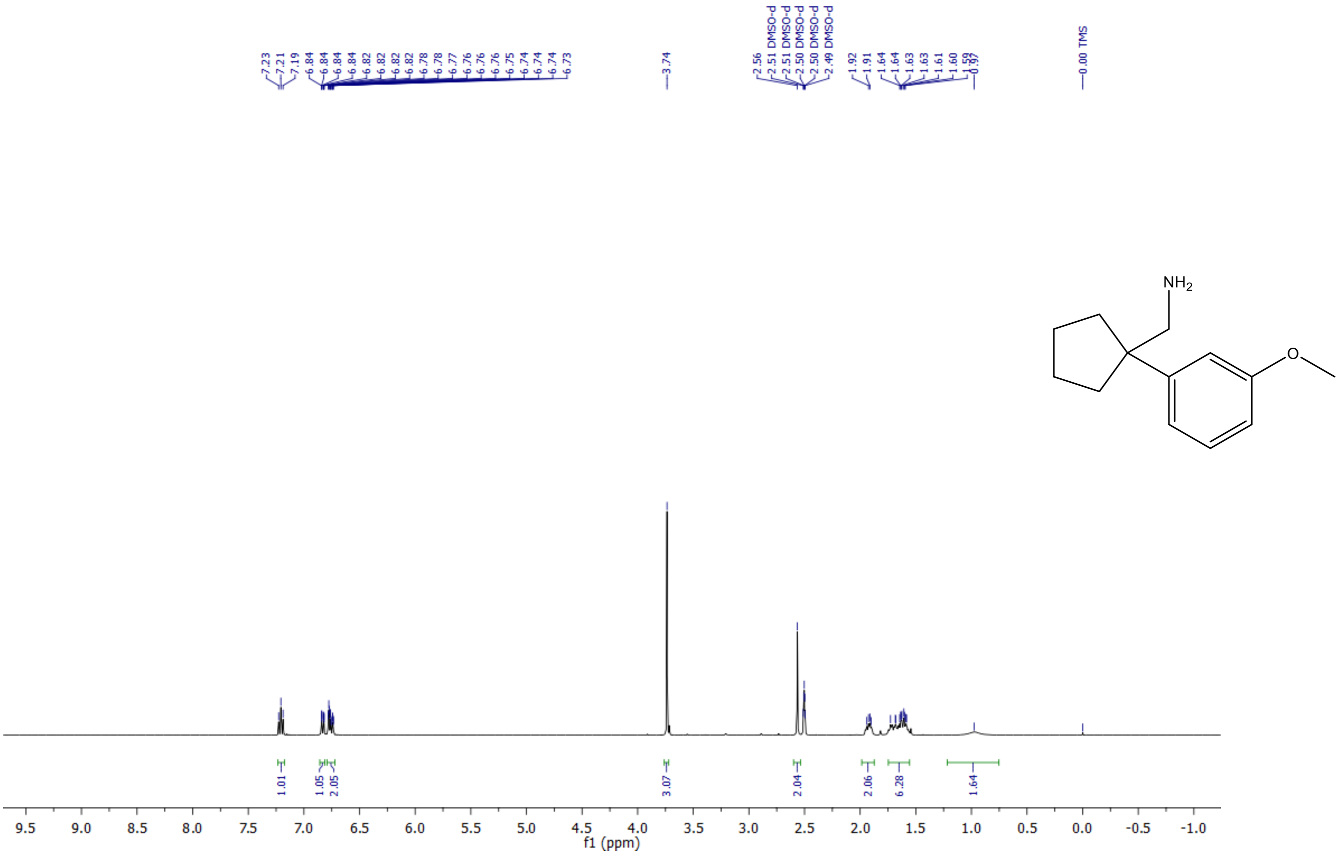

(1-(*m*-Tolyl)cyclopentyl)methanamine (68)

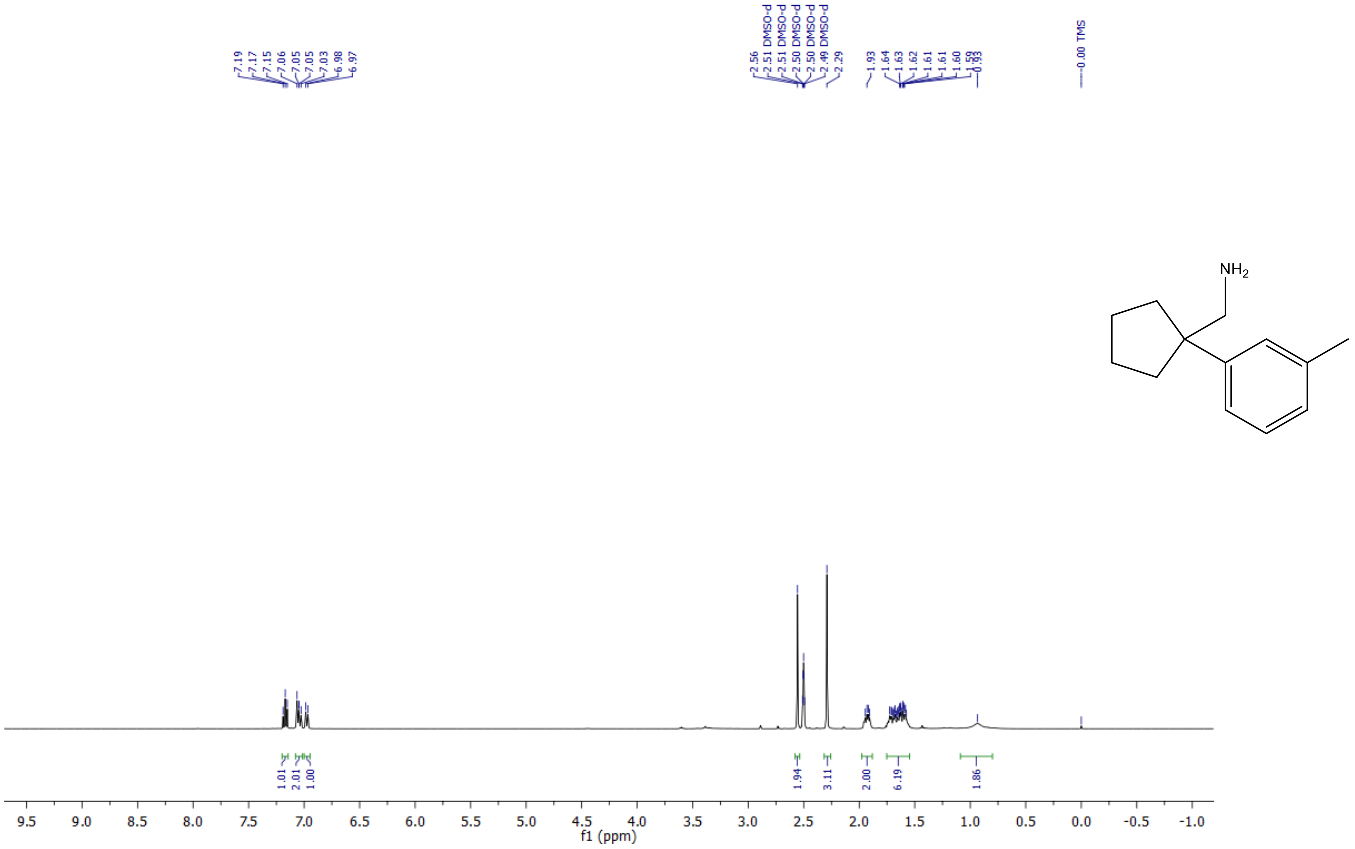

1-(Thiophen-2-yl)cyclopentyl)methanamine (69)

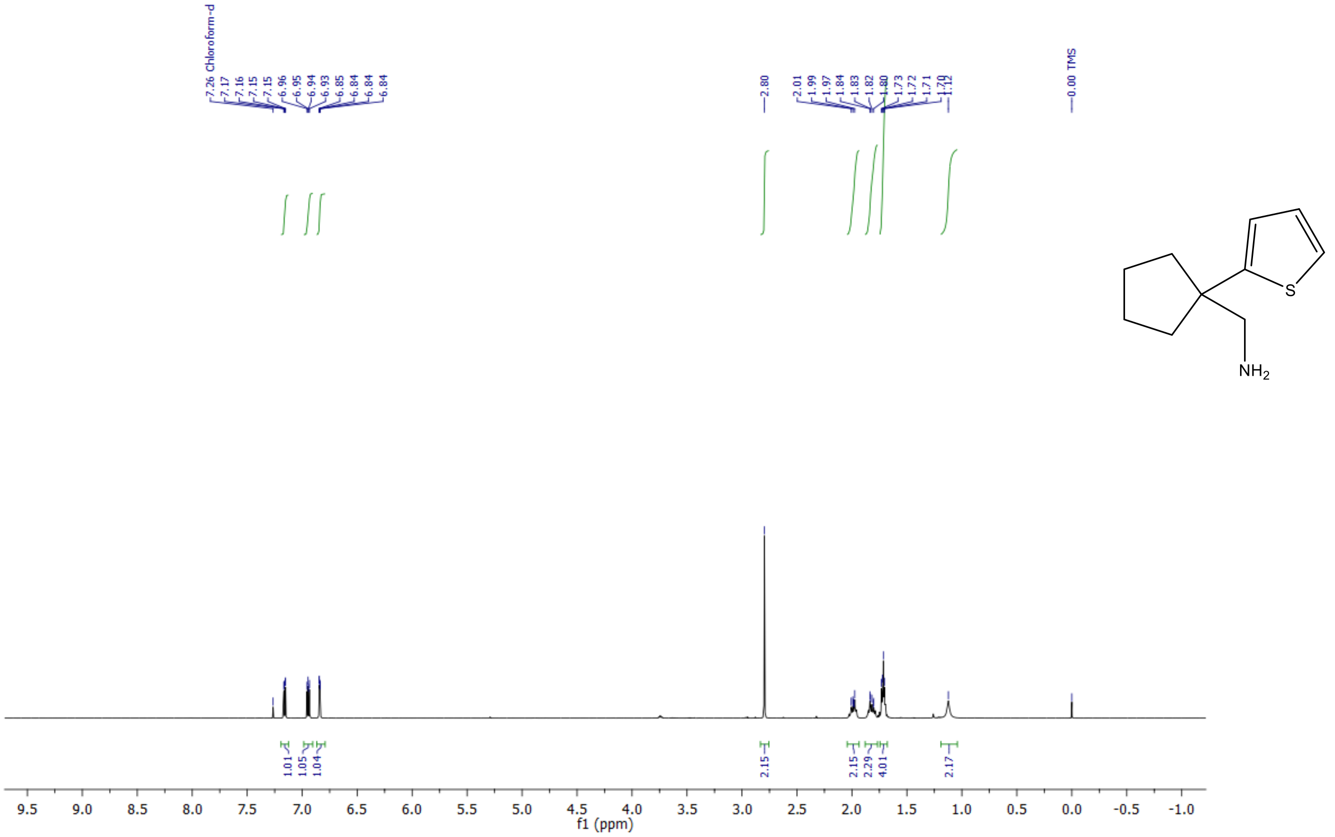

1-(Thiophen-3-yl)cyclopentyl)methanamine (70)

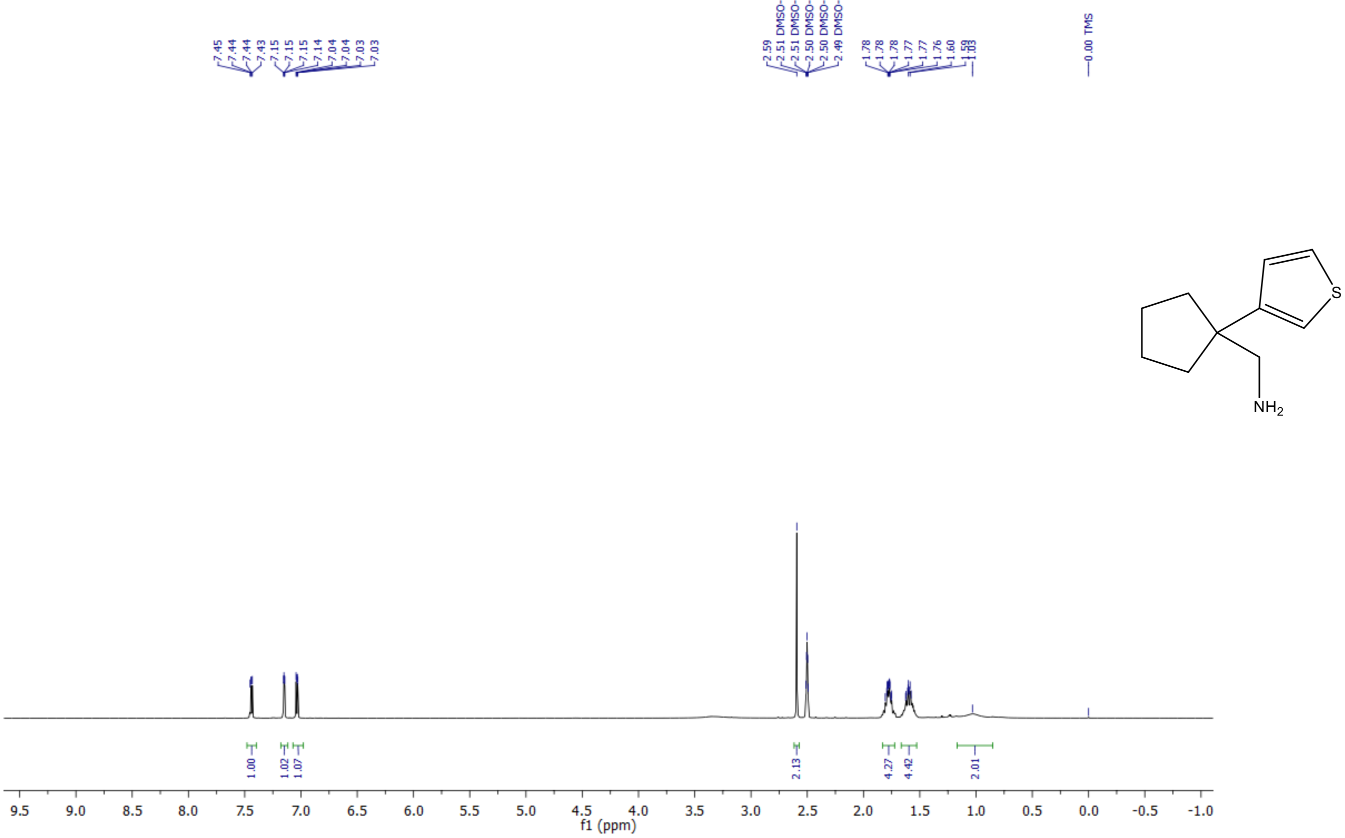

3-(Thiophen-2-yl)tetrahydrofuran-3-yl)methanamine (71)

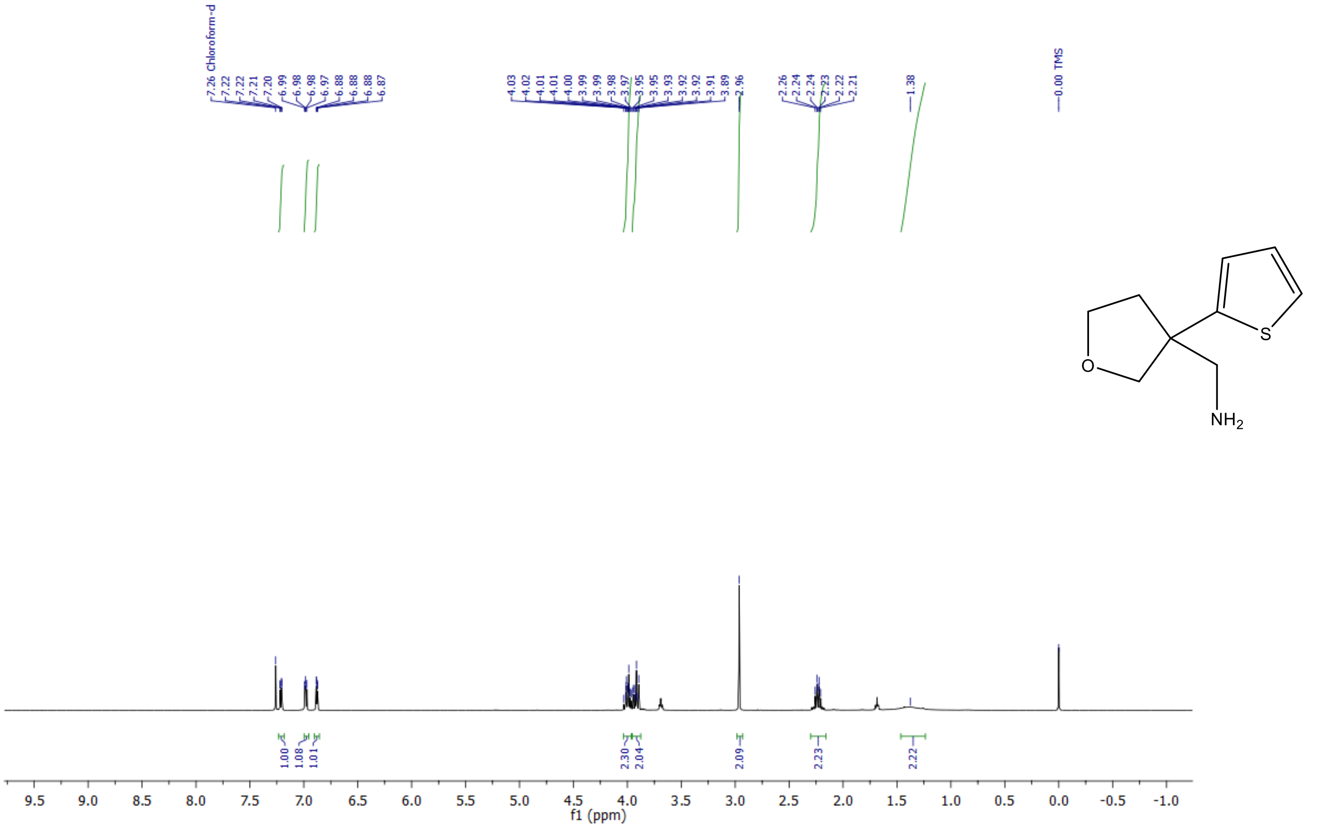

***N*-((1-(3,4-Dimethoxyphenyl)cyclohexyl)methyl)-2-methoxybenzamide (15)**

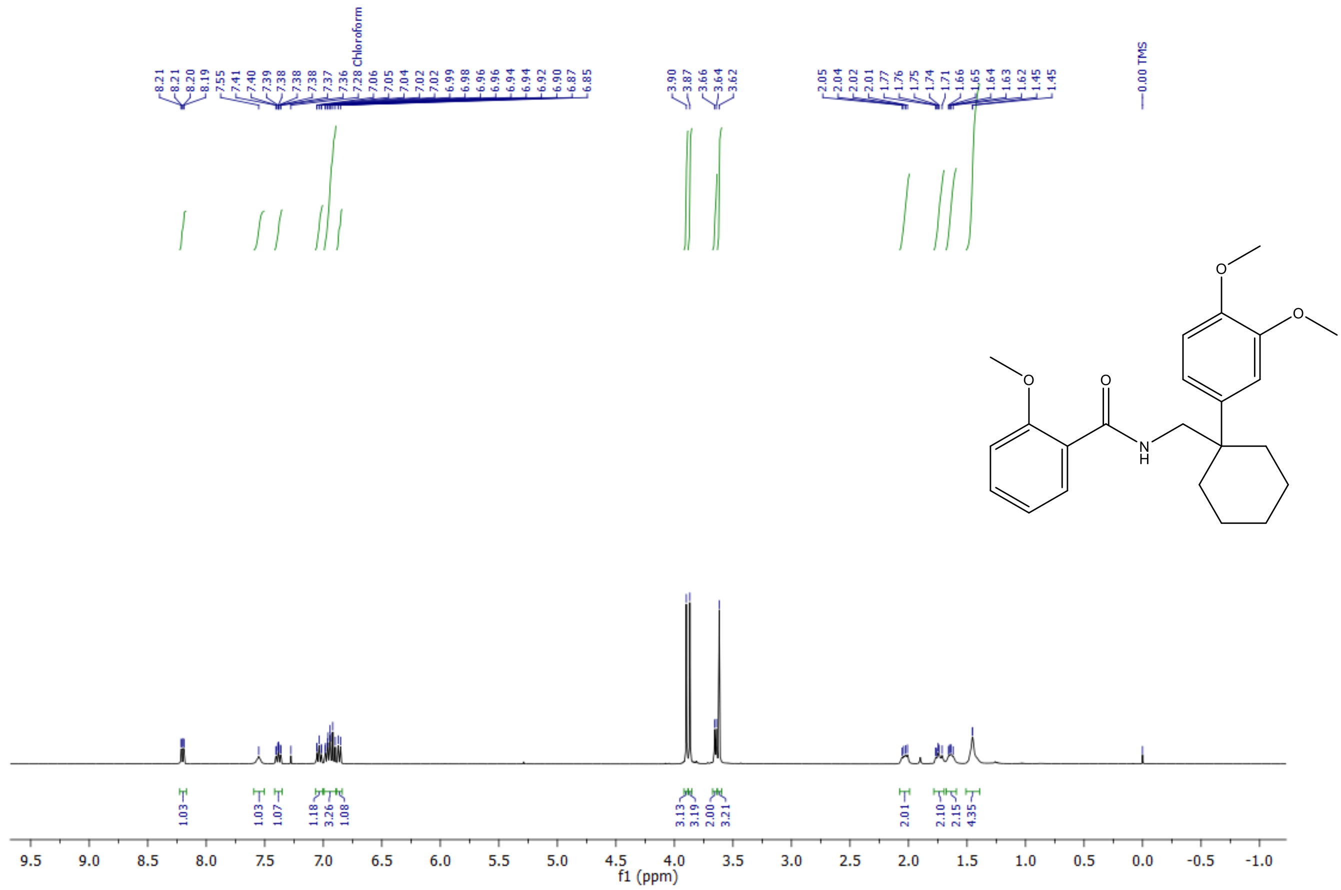

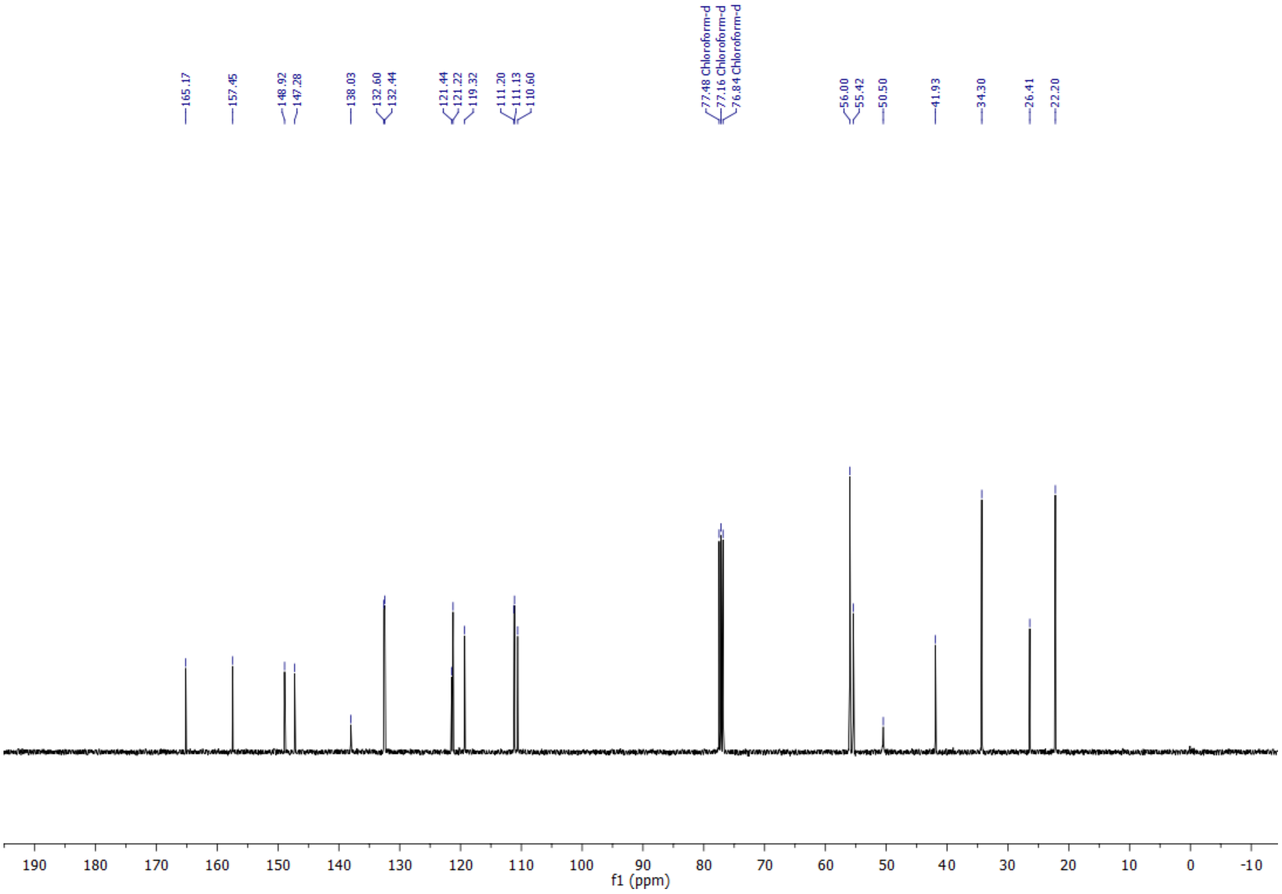

2-Methoxy-*N*-((1-(4-methoxyphenyl)cyclohexyl)methyl)benzamide (16)

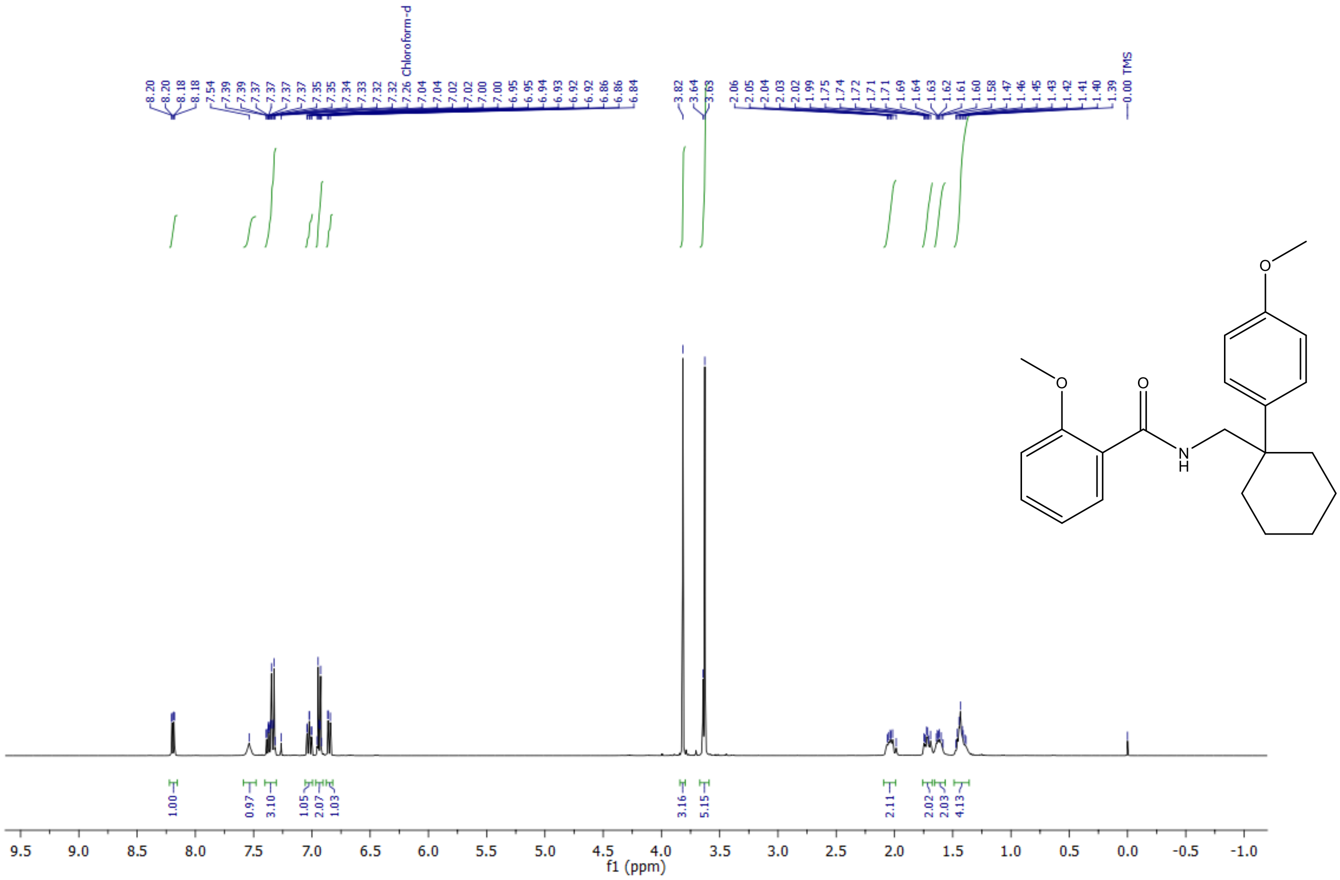

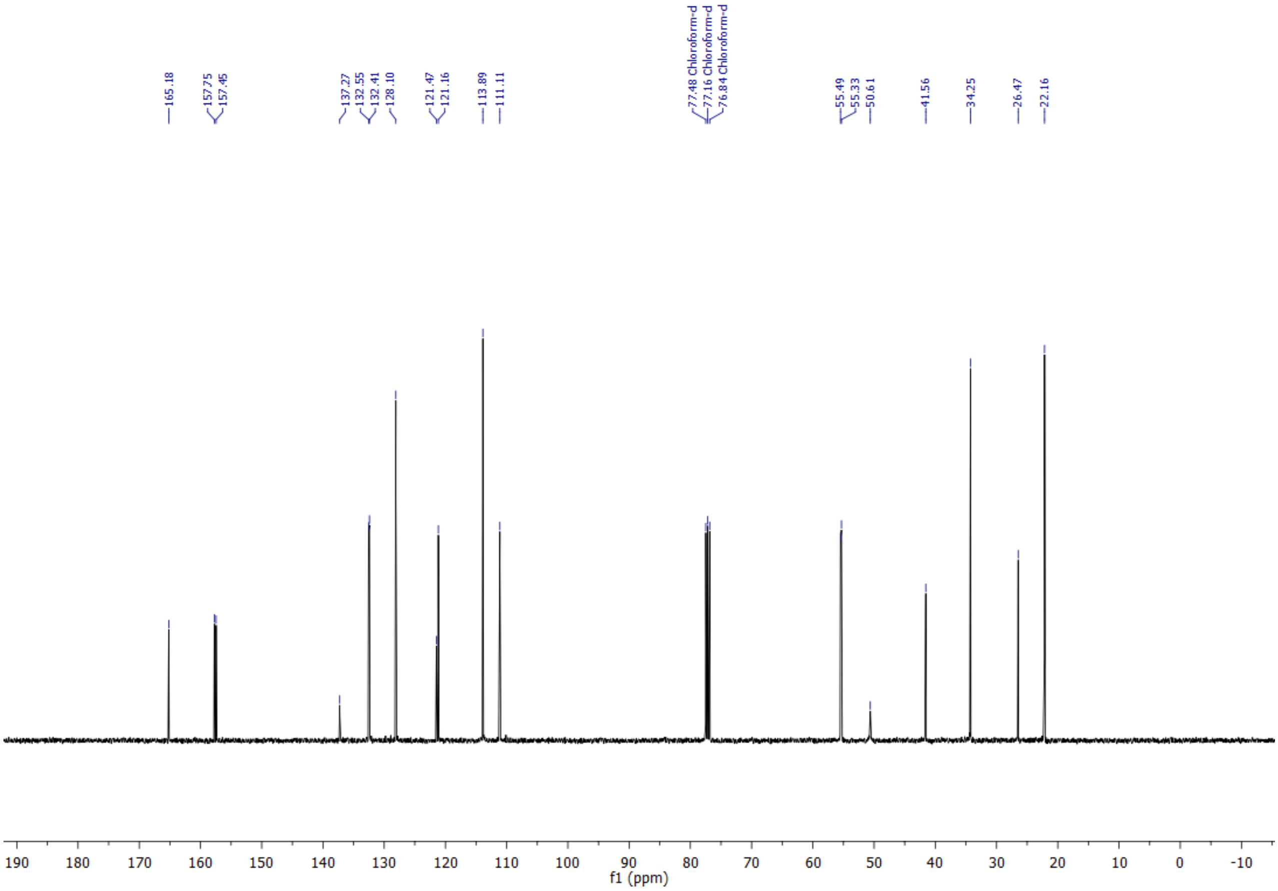

2-Methoxy-*N*-((1-(4-(trifluoromethyl)phenyl)cyclohexyl)methyl)benzamide (17)

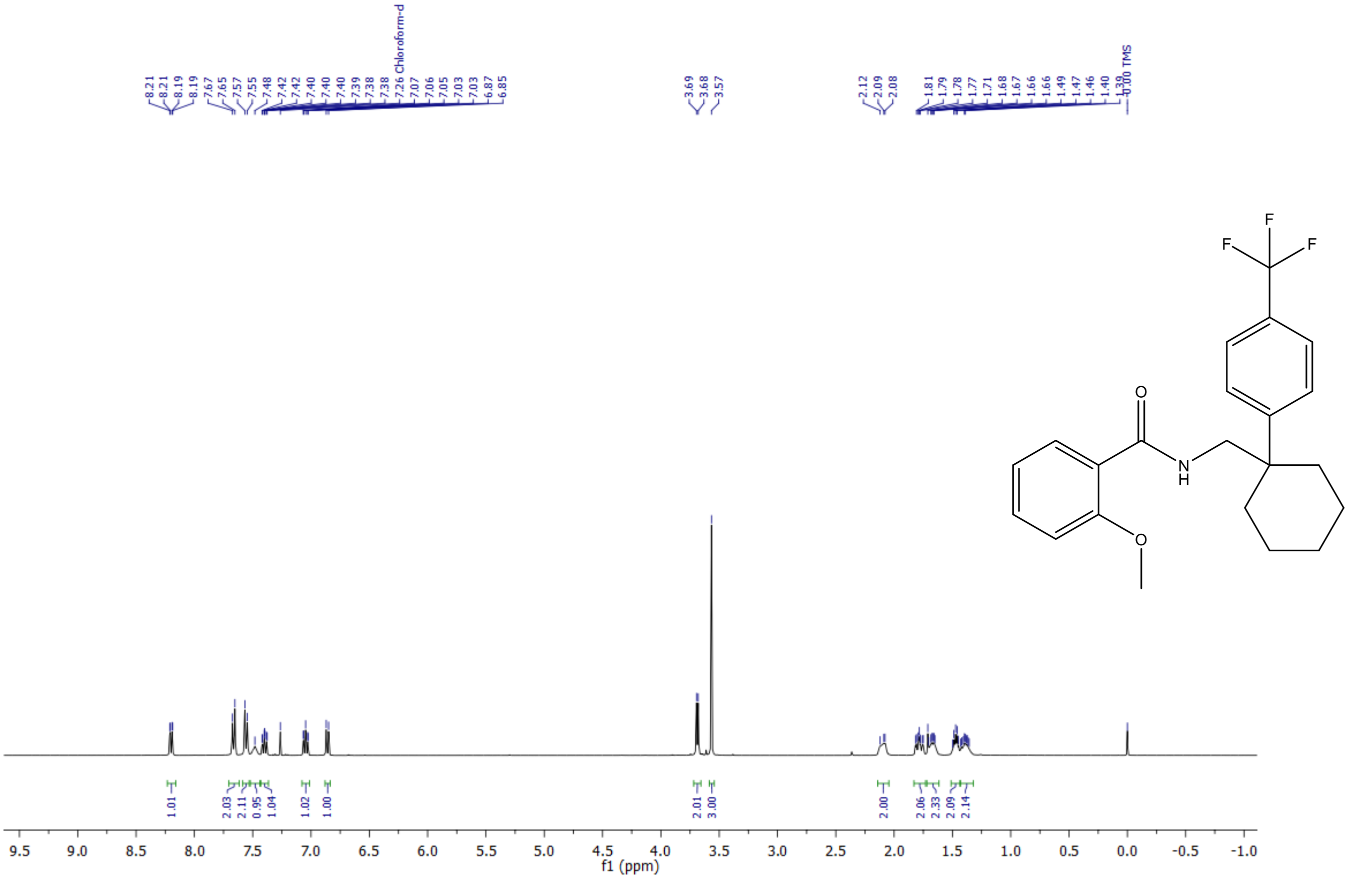

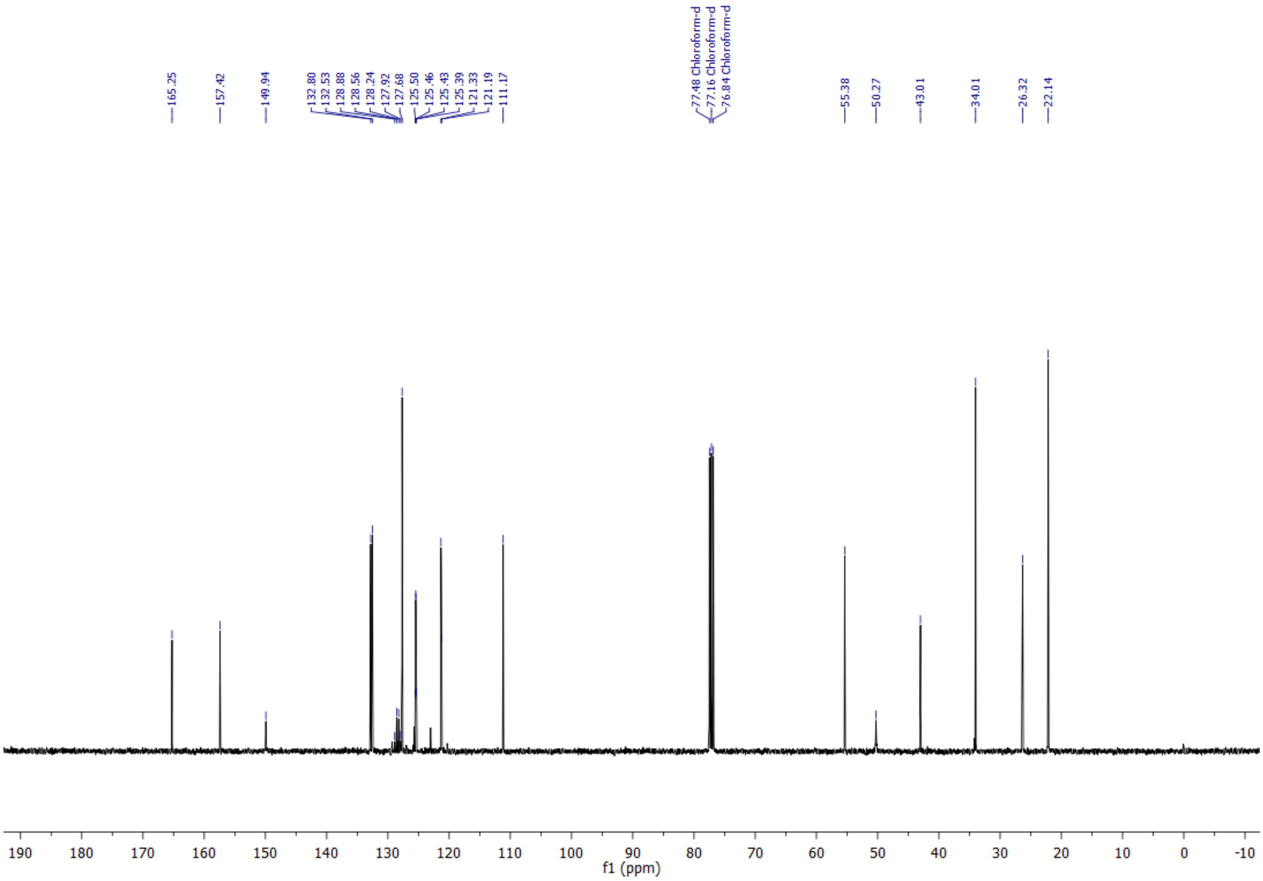

2-Methoxy-*N*-((1-(2-methoxyphenyl)cyclohexyl)methyl)benzamide (18)

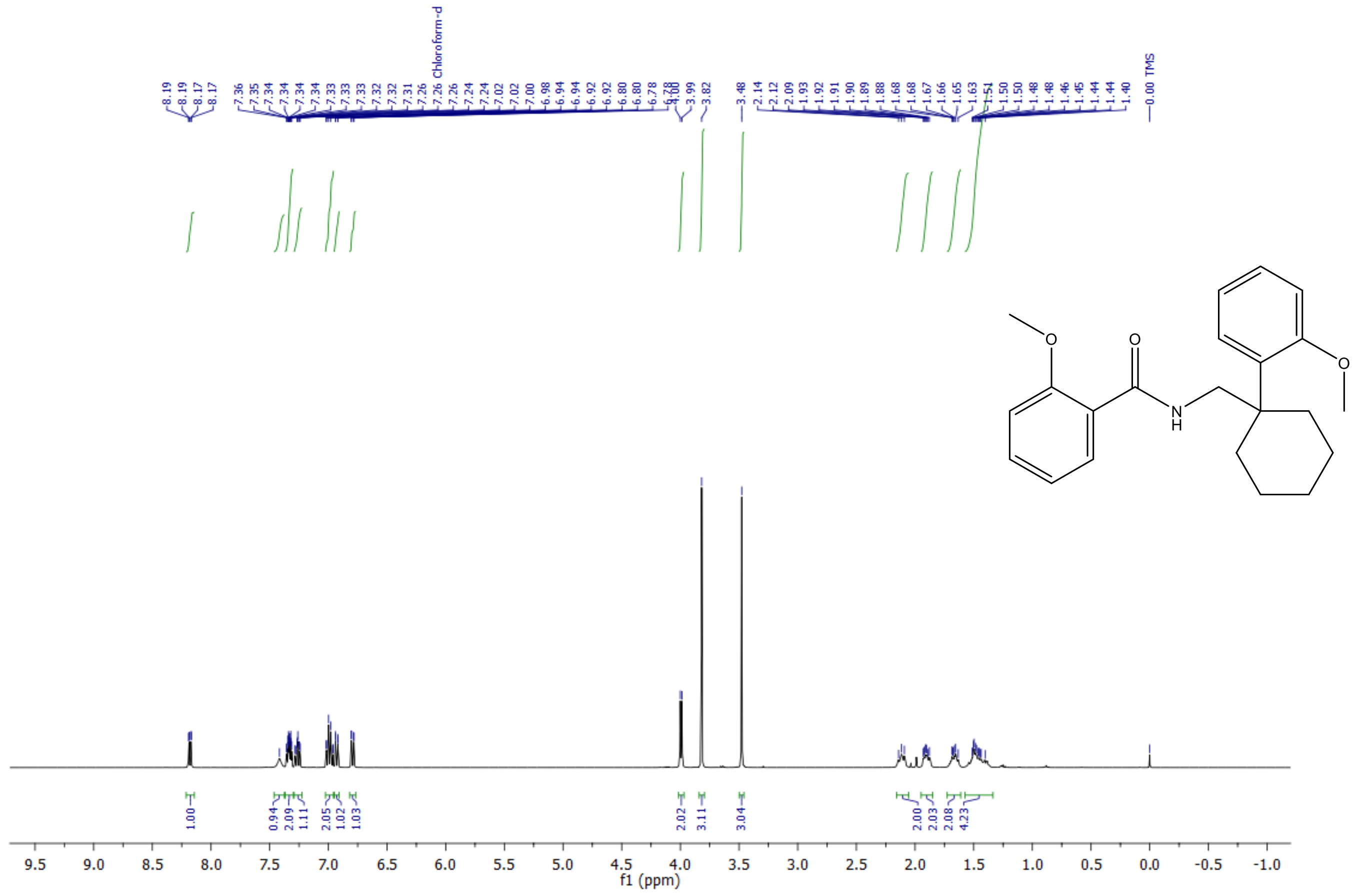

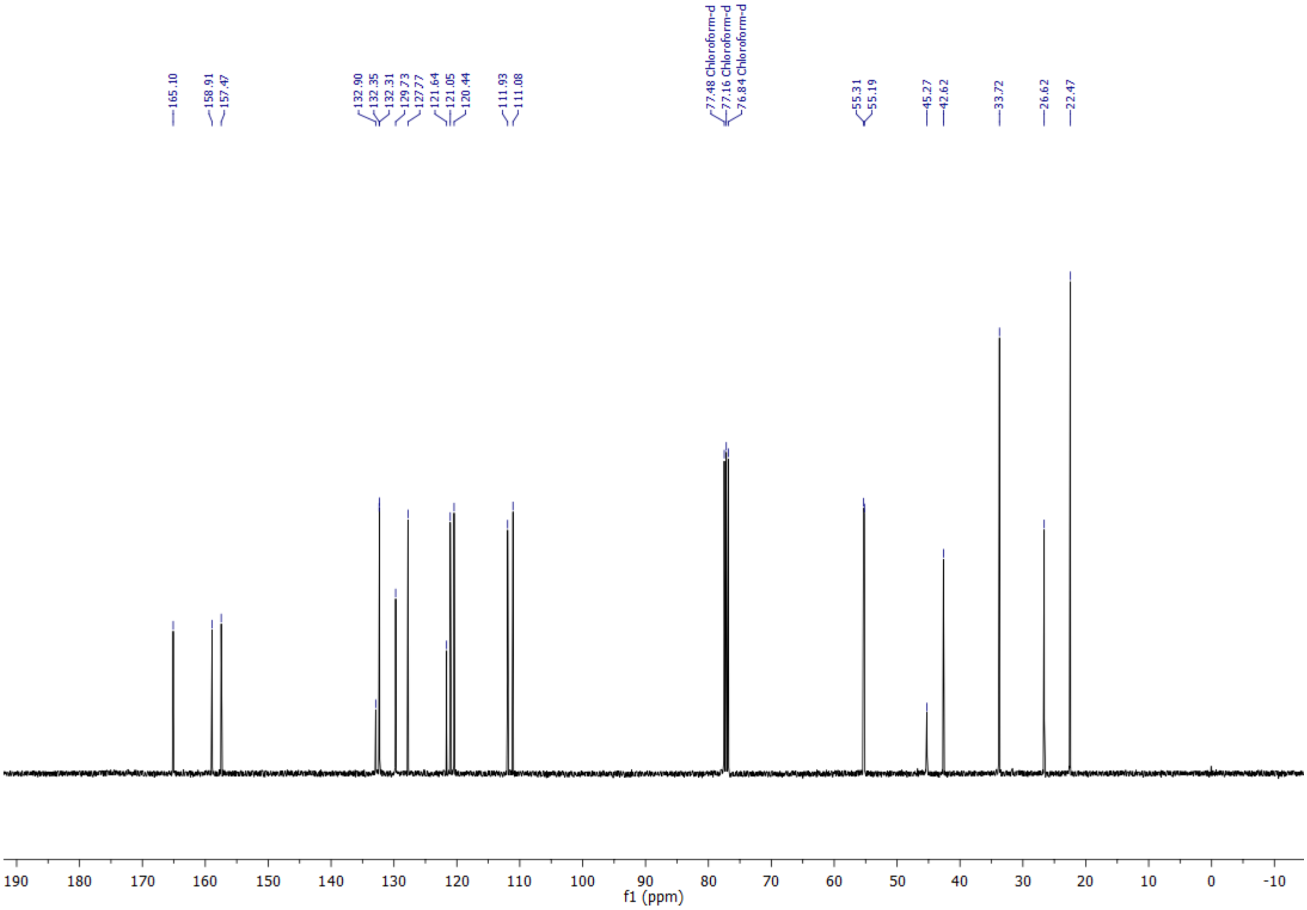

2-Methoxy-*N*-((1-(3-methoxyphenyl)cyclohexyl)methyl)benzamide (19)

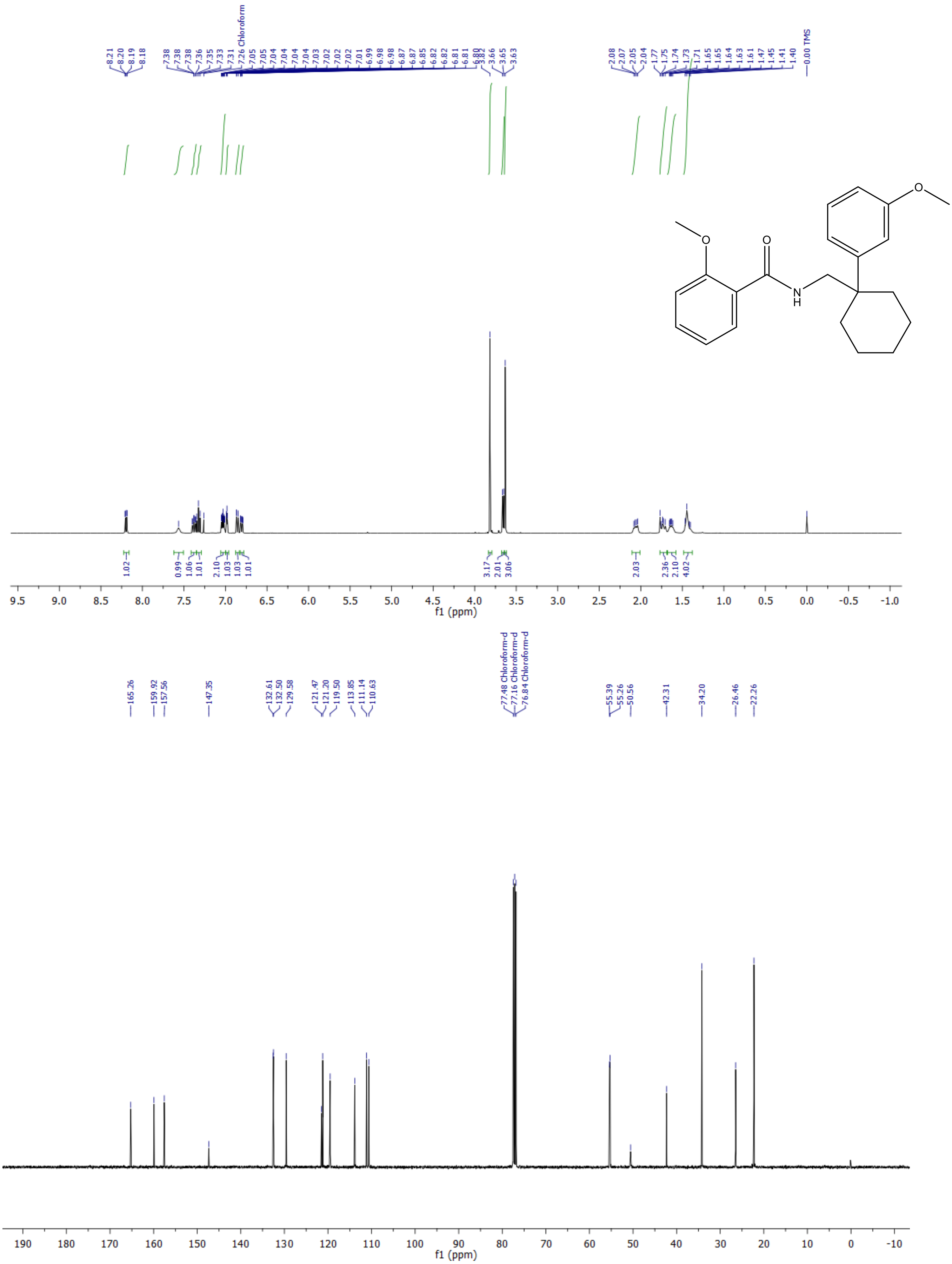

2-Methoxy-*N*-((1-(*m*-tolyl)cyclohexyl)methyl)benzamide (20)

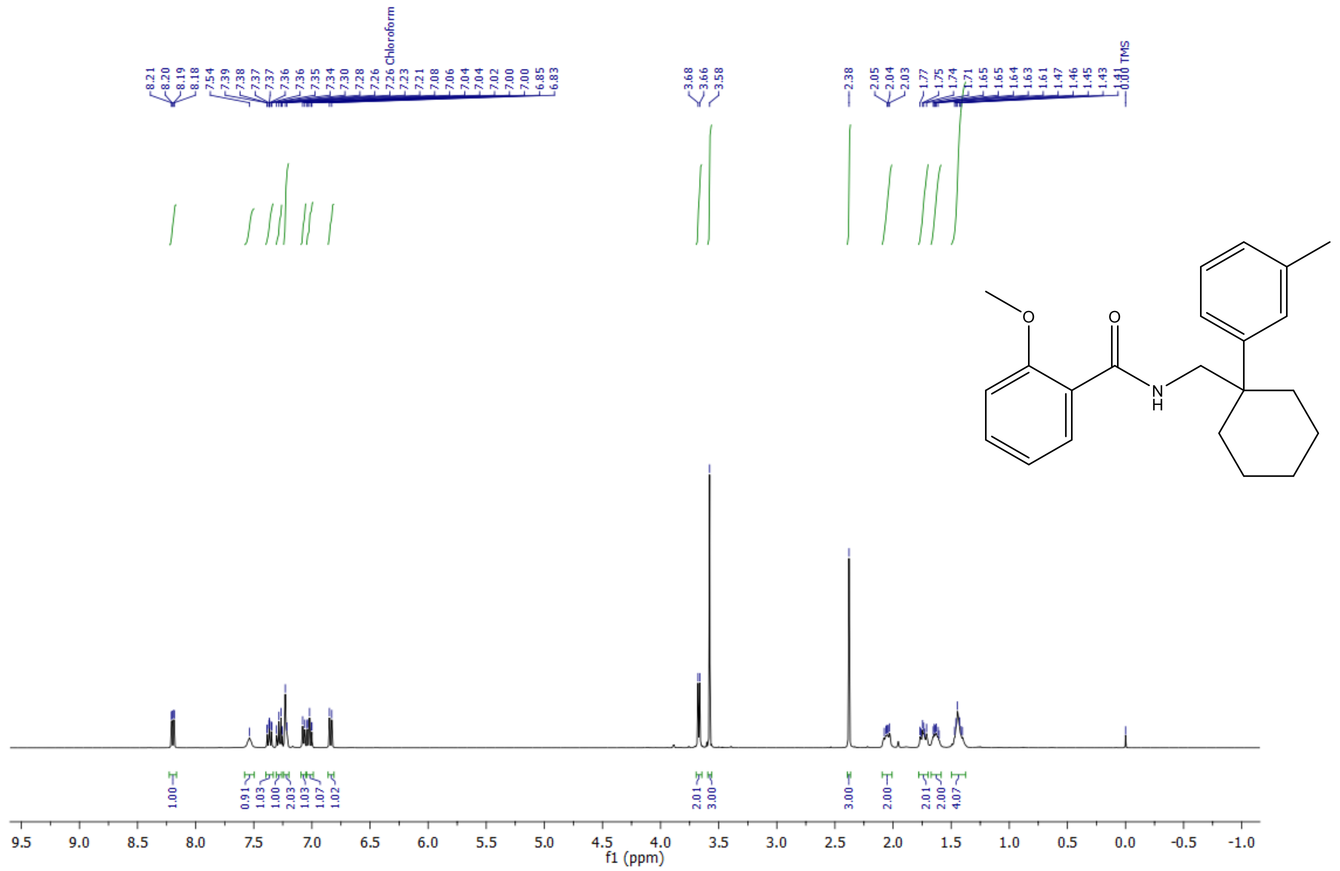

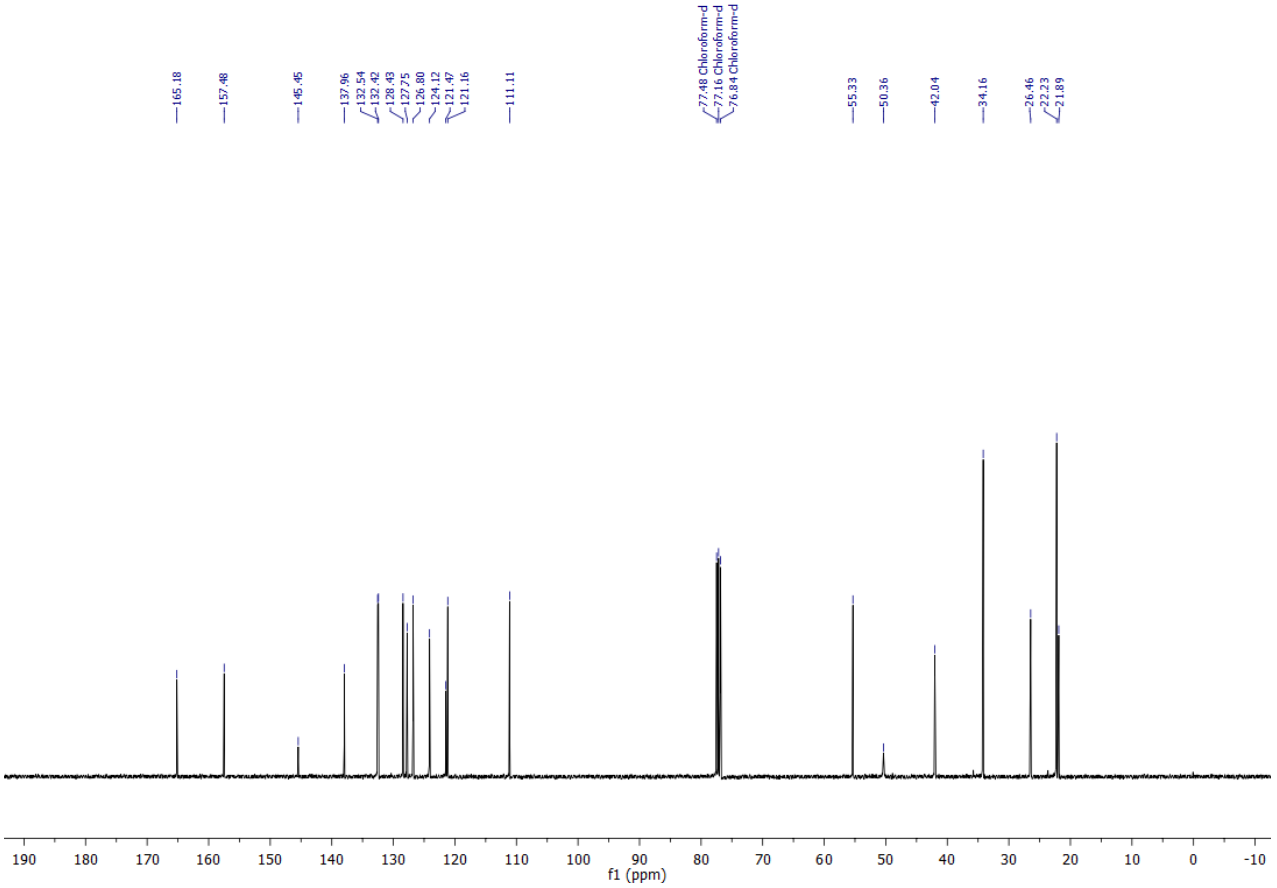

2-Methoxy-*N*-((1-(thiophen-3-yl)cyclohexyl)methyl)benzamide (21)

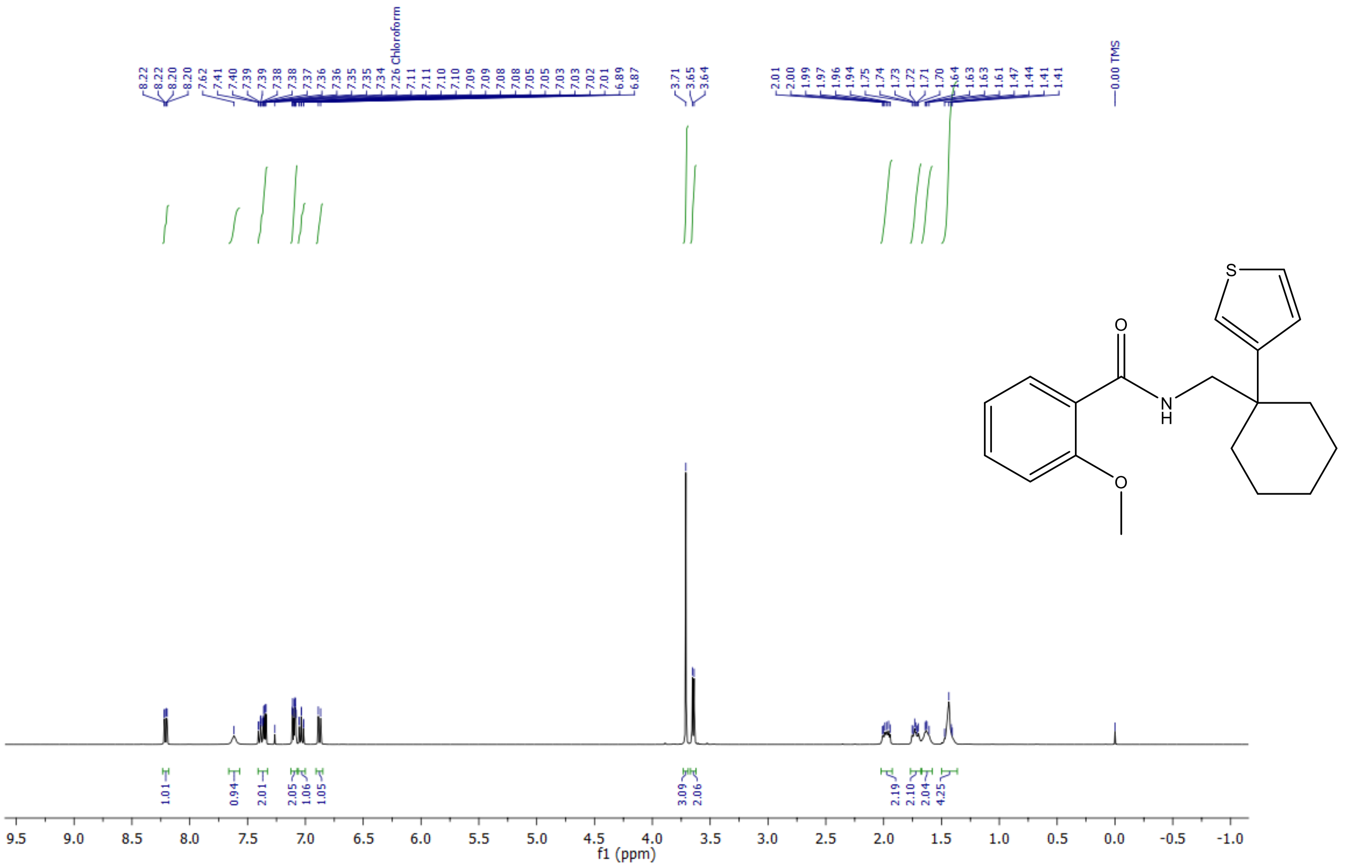

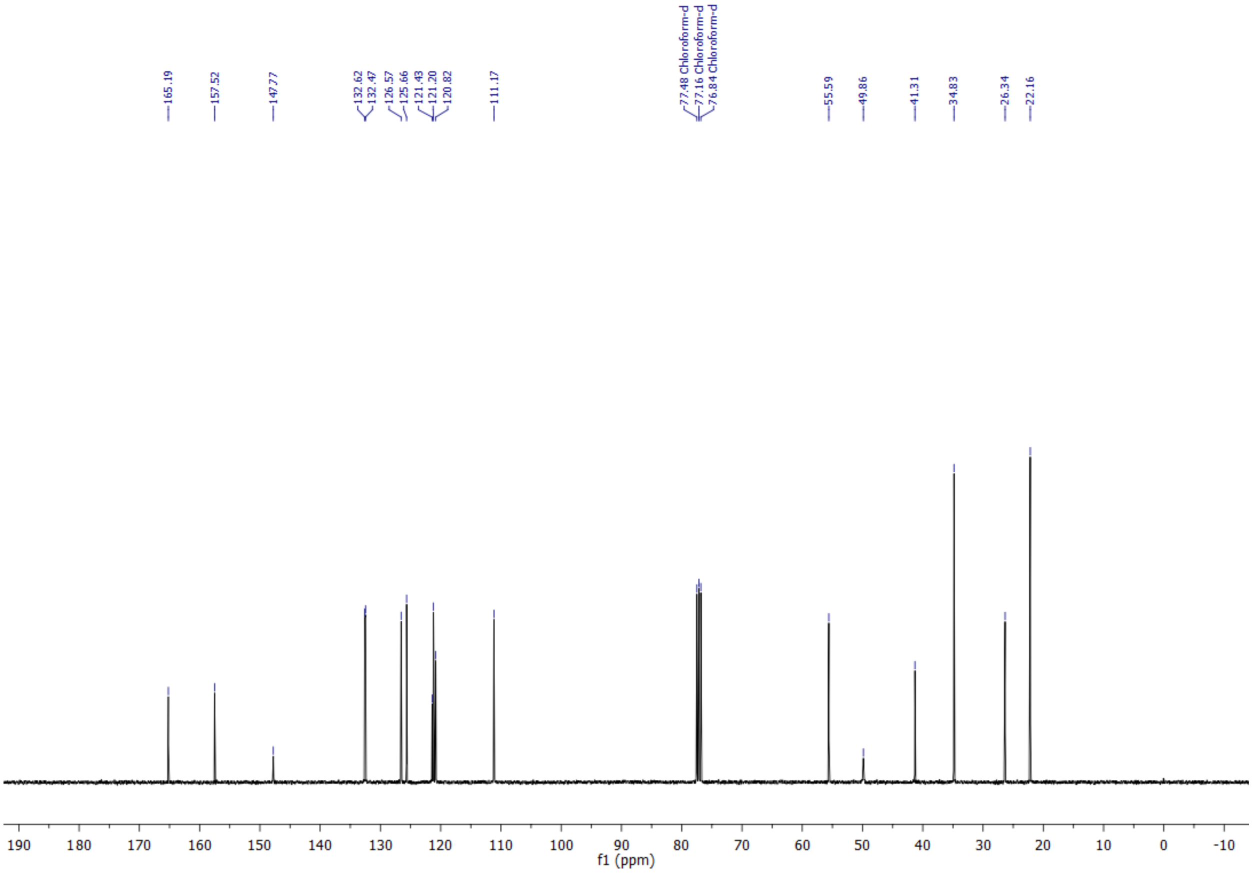

1-(3,4-Dimethoxyphenyl)cyclohexane-1-carbonitrile (72)

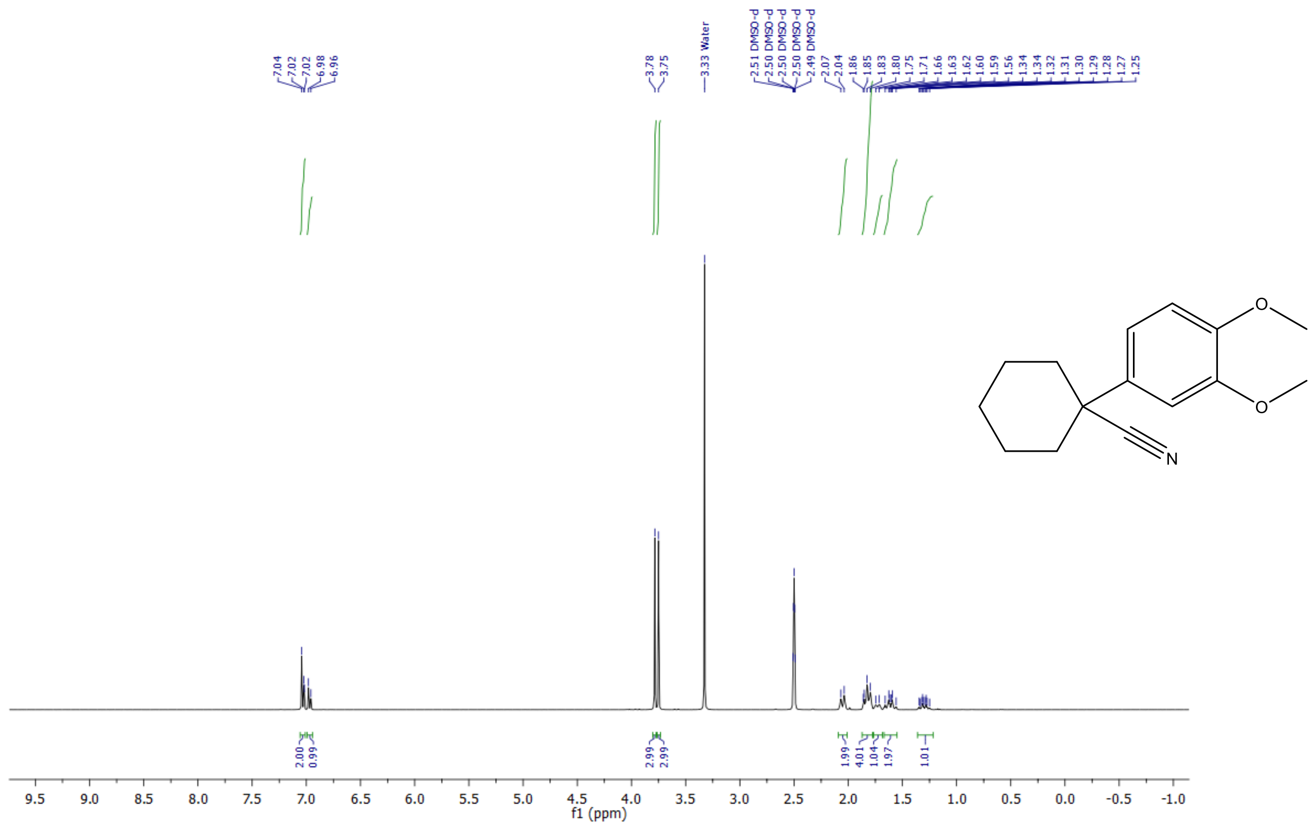

1-(4-Methoxyphenyl)cyclohexane-1-carbonitrile (73)

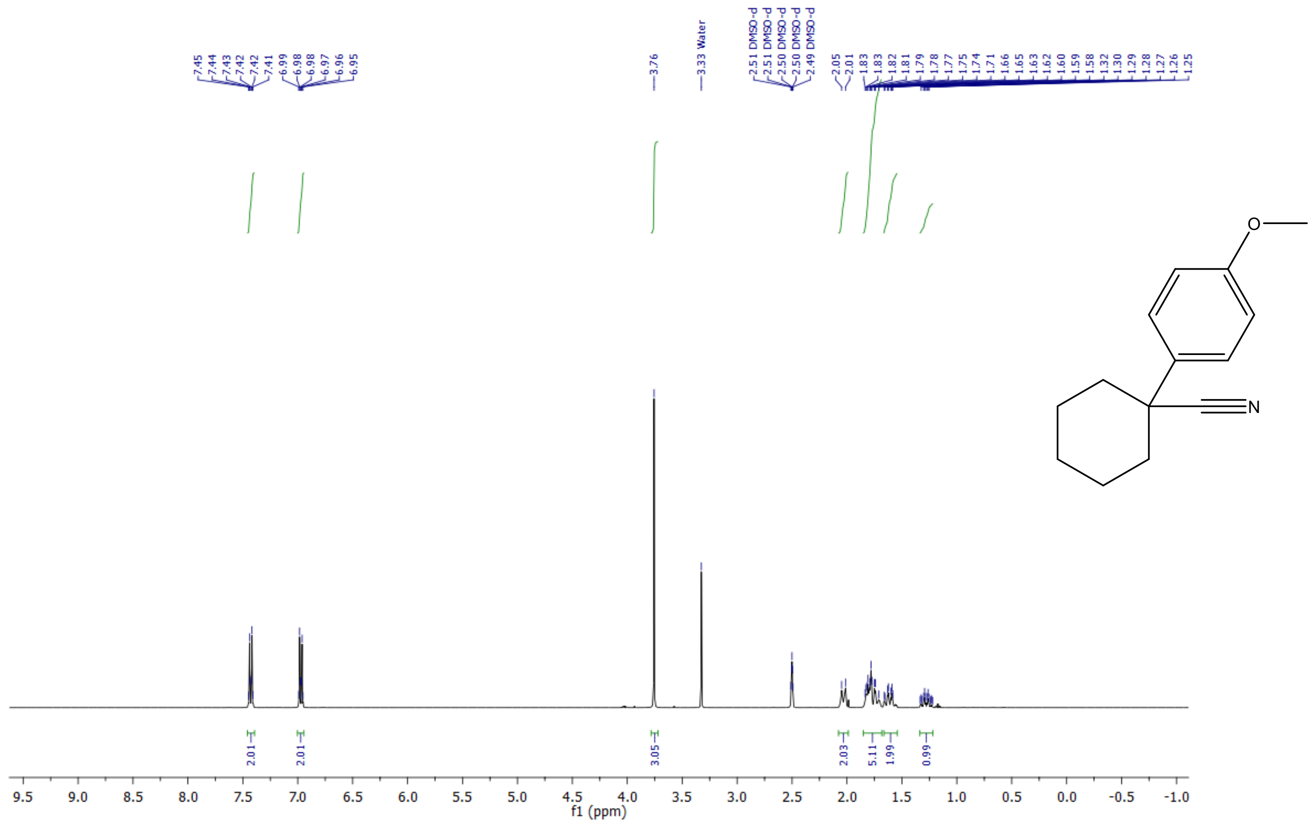

1-(4-(Trifluoromethyl)phenyl)cyclohexane-1-carbonitrile (74)

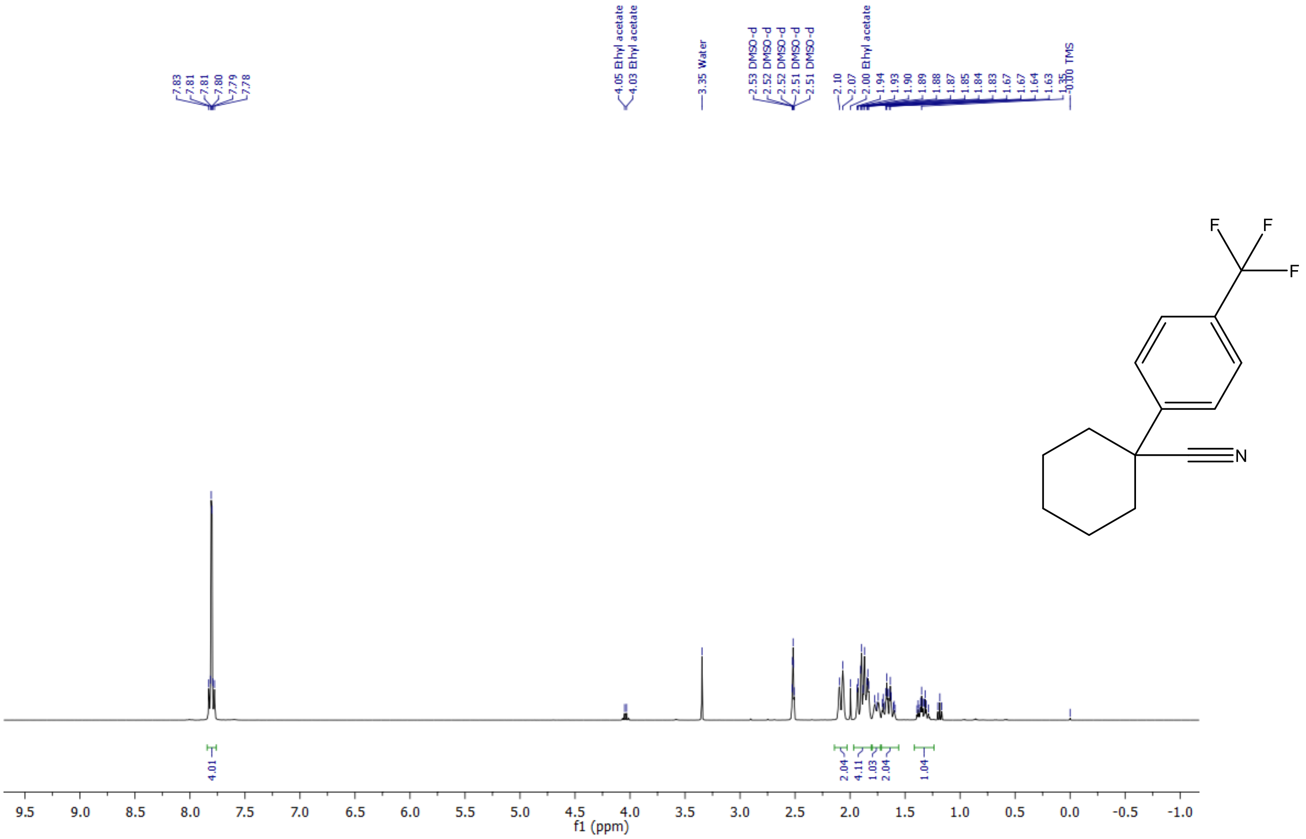

1-(2-Methoxyphenyl)cyclohexane-1-carbonitrile (75)

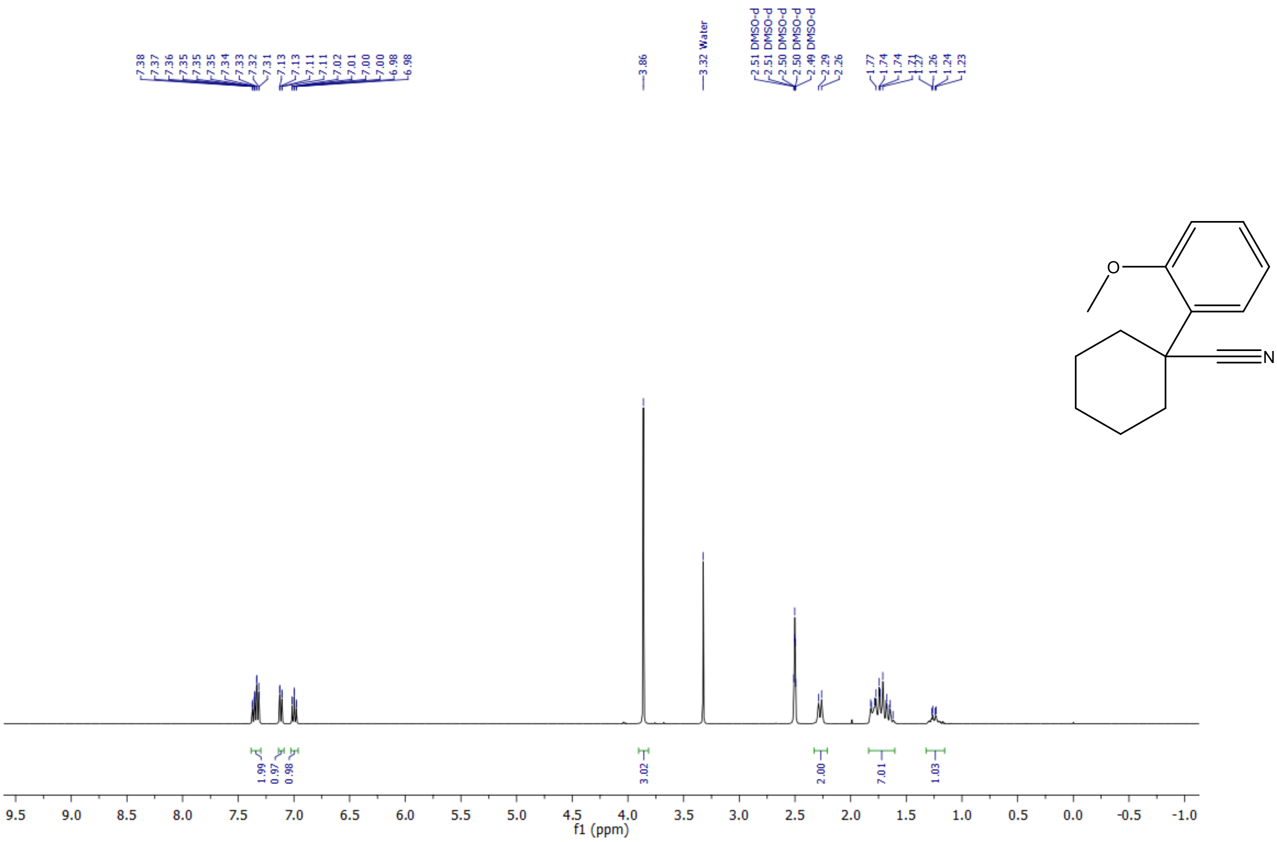

1-(3-Methoxyphenyl)cyclohexane-1-carbonitrile (76)

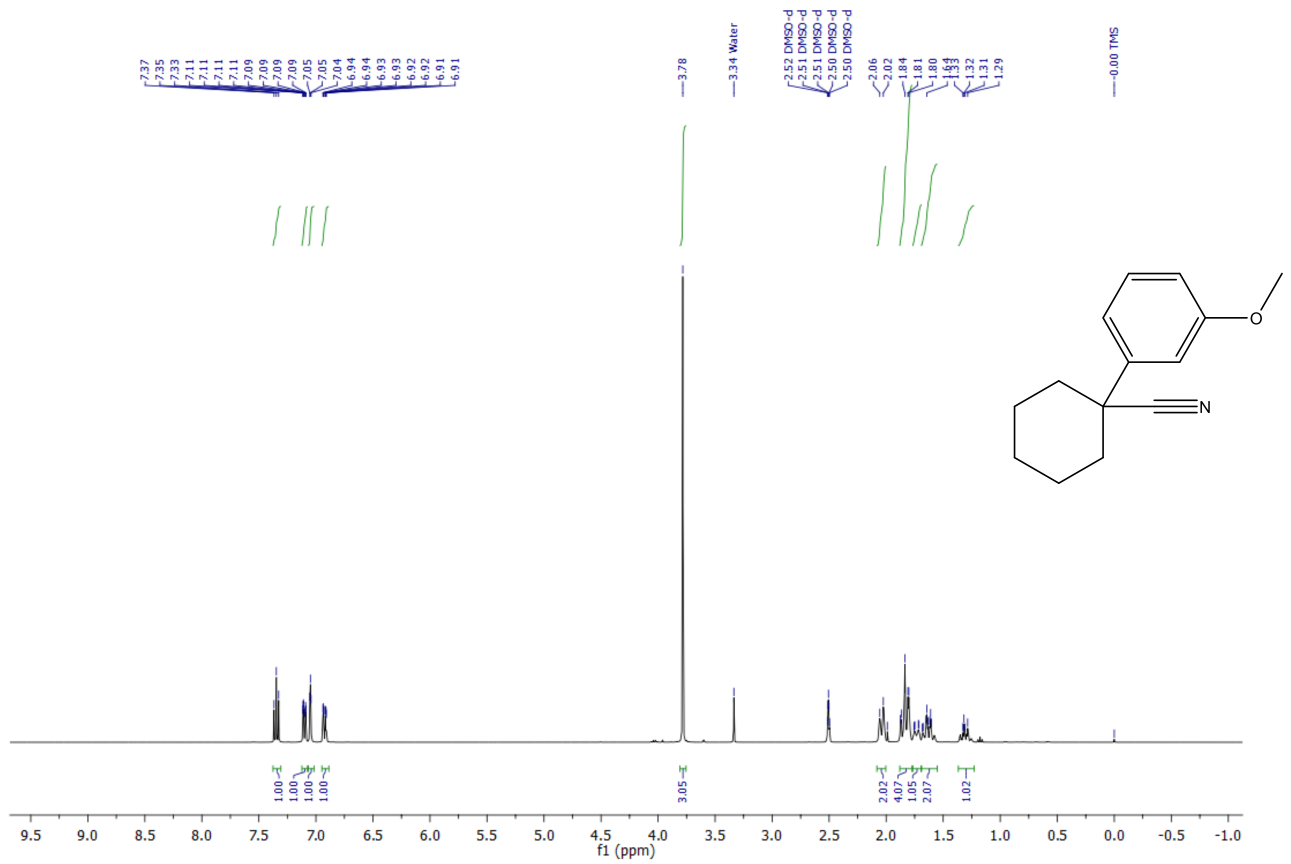

1-(*m*-Tolyl)cyclohexane-1-carbonitrile (77)

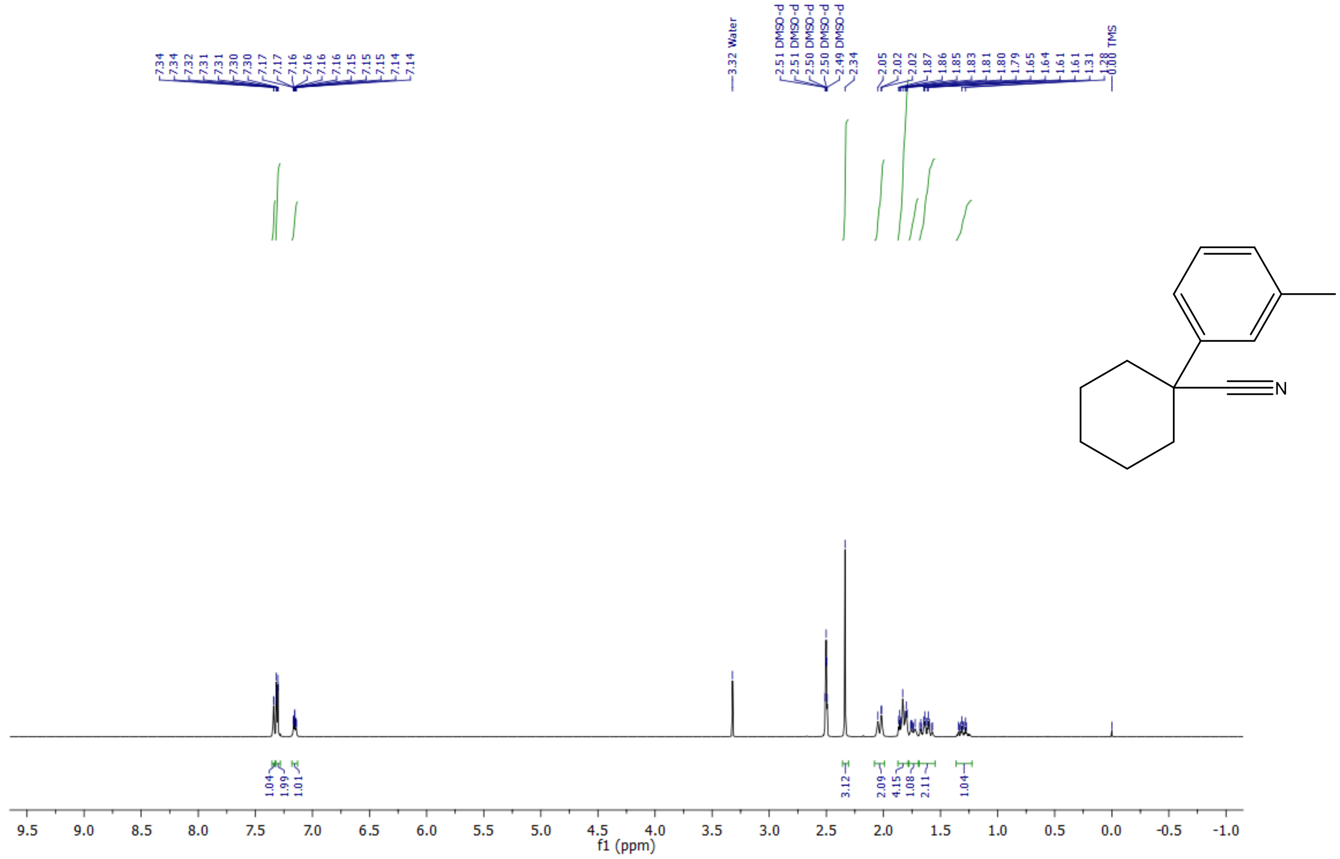

1-(Thiophen-3-yl)cyclohexane-1-carbonitrile (78)

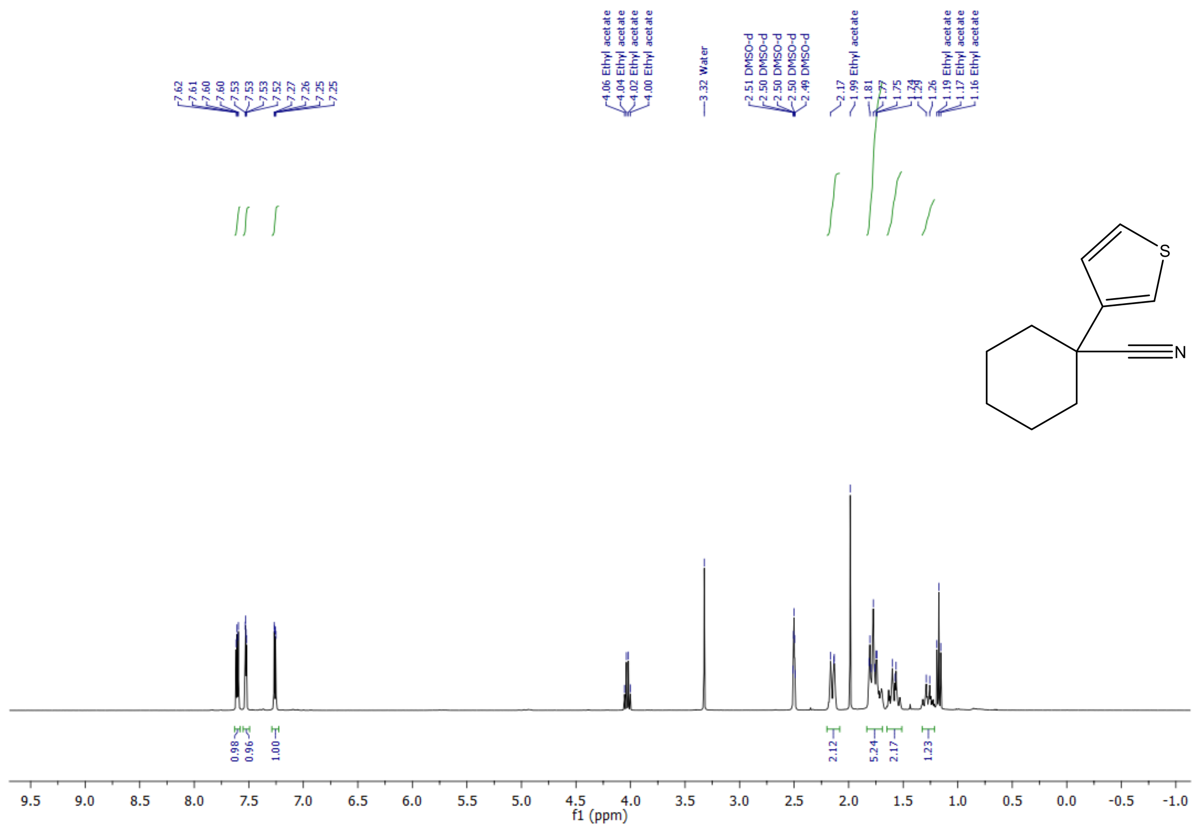

(1-(3,4-Dimethoxyphenyl)cyclohexyl)methanamine (79)

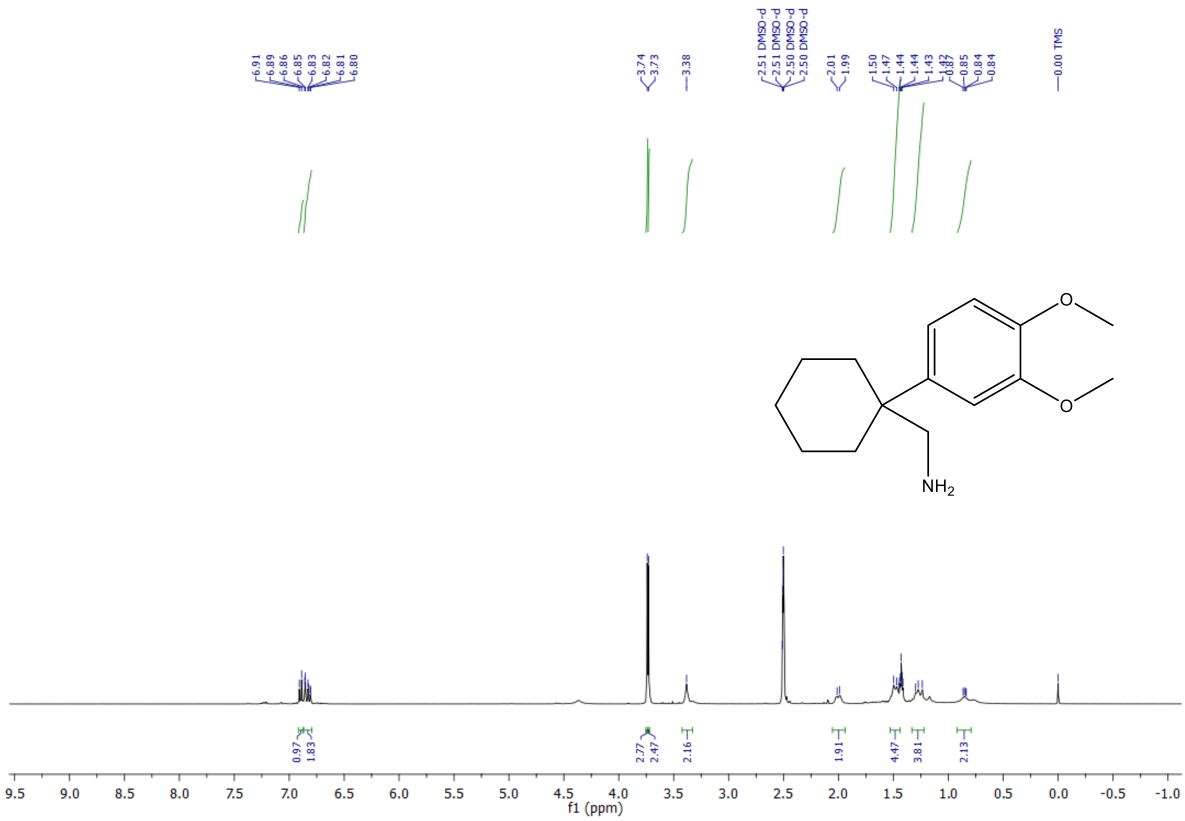

(1-(4-Methoxyphenyl)cyclohexyl)methanamine (80)

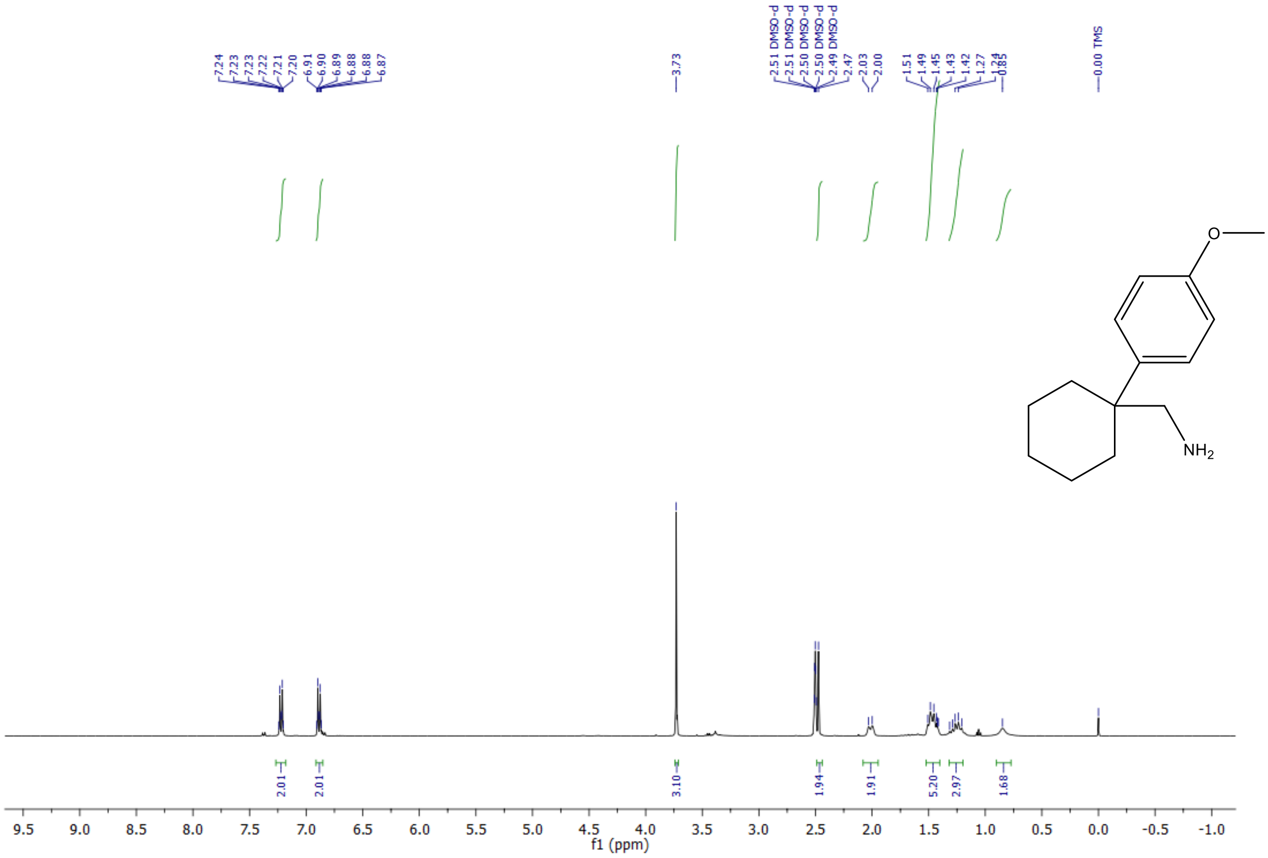

(1-(4-(Trifluoromethyl)phenyl)cyclohexyl)methanamine (81)

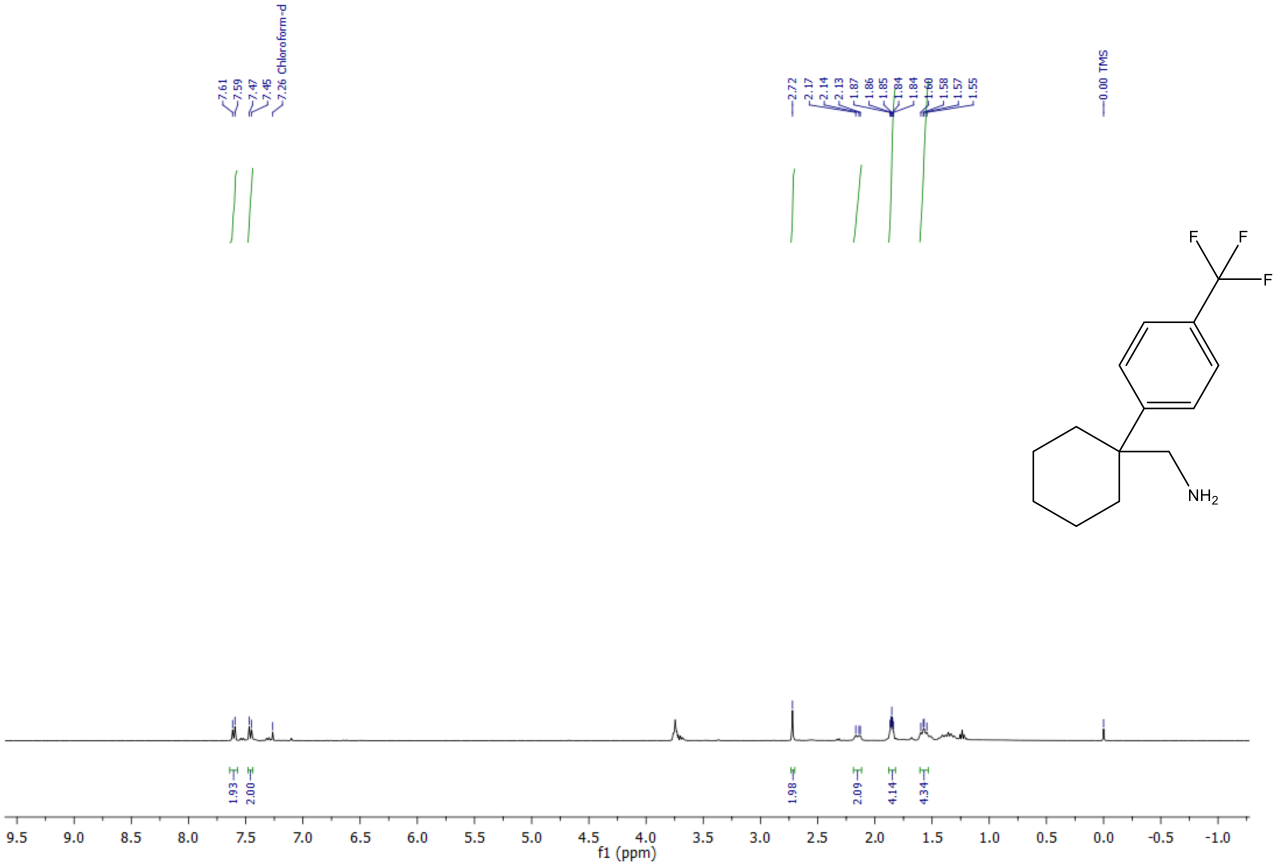

(1-(2-Methoxyphenyl)cyclohexyl)methanamine (82)

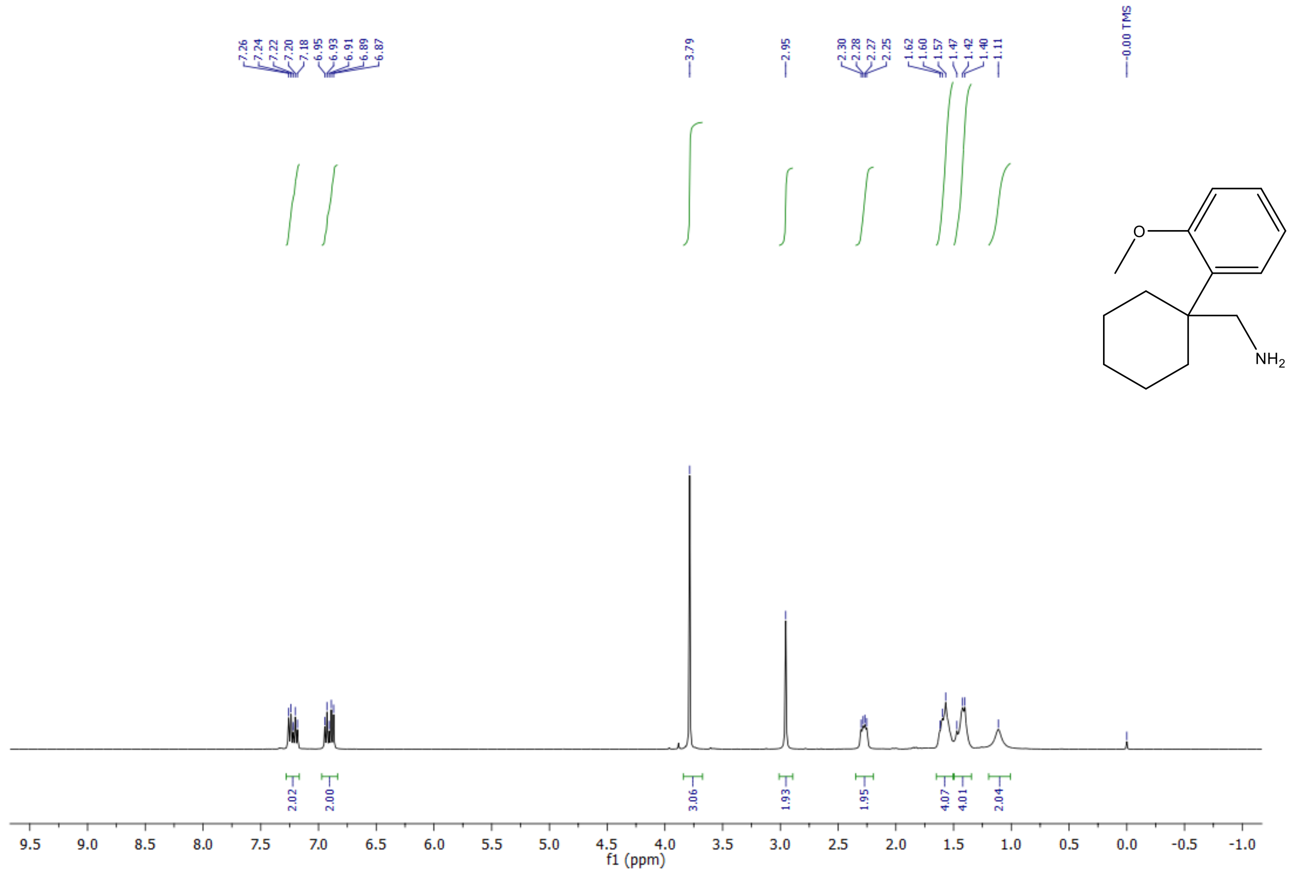

(1-(3-Methoxyphenyl)cyclohexyl)methanamine (83)

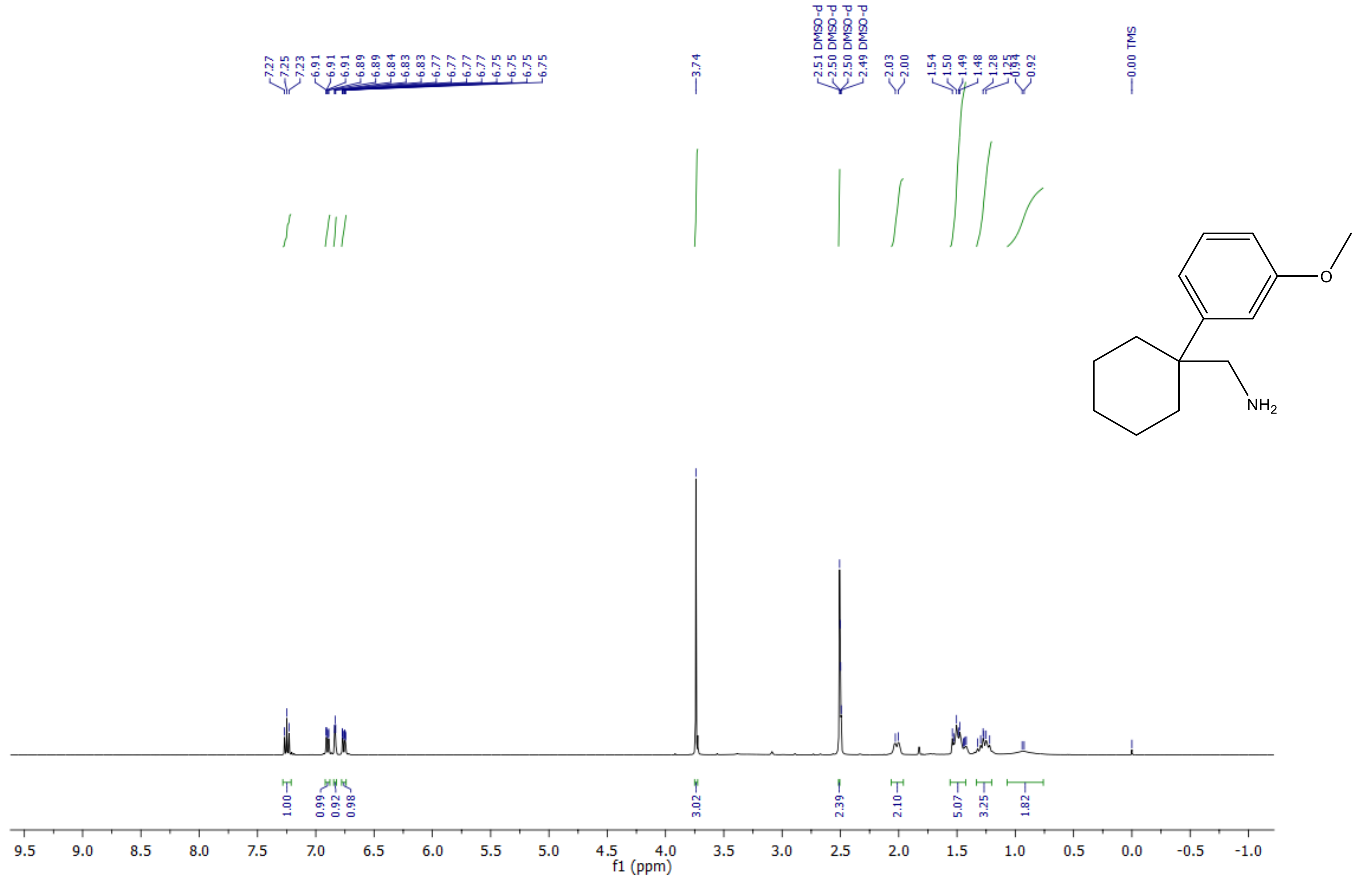

(1-(*m*-Tolyl)cyclohexyl)methanamine (84)

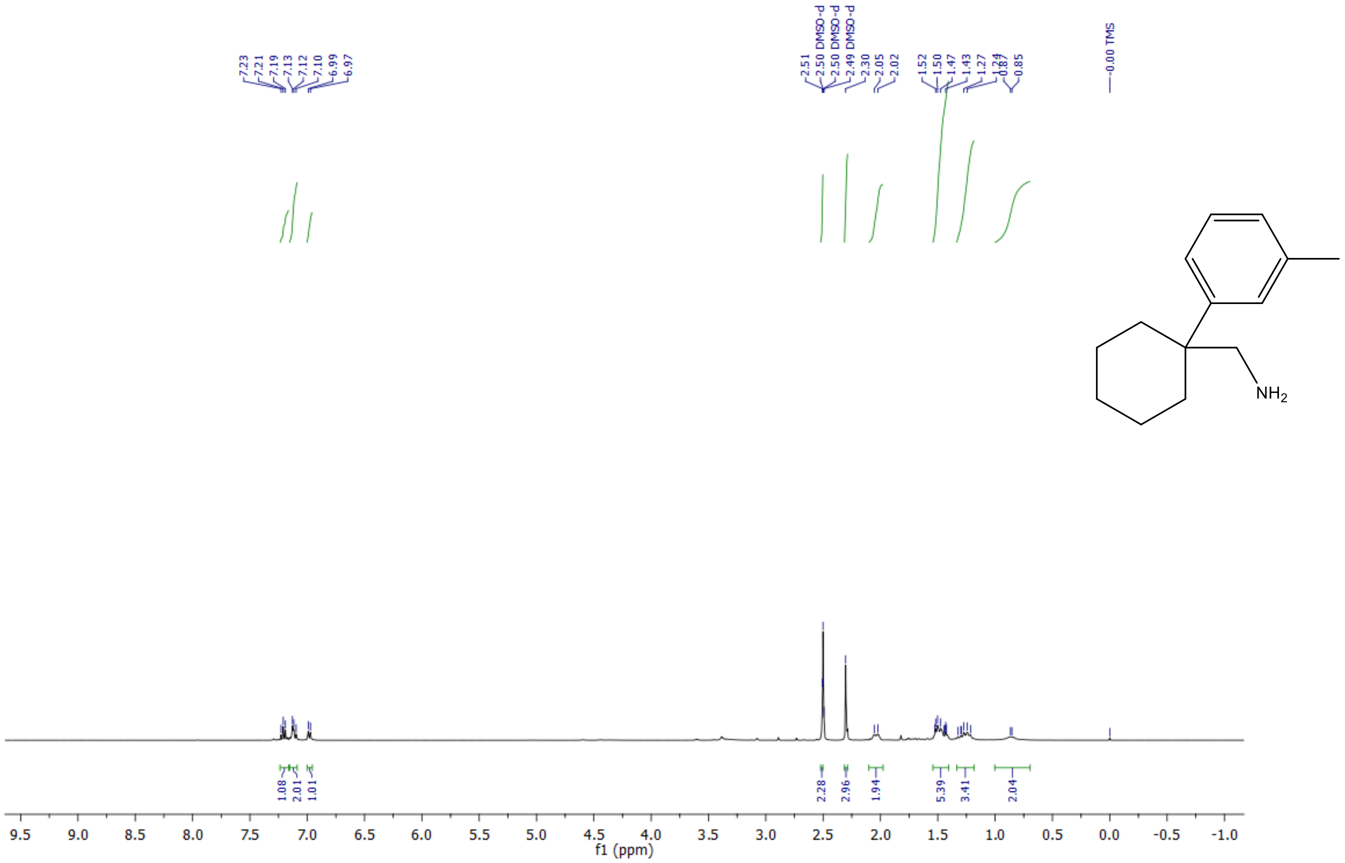

(1-(Thiophen-3-yl)cyclohexyl)methanamine (85)

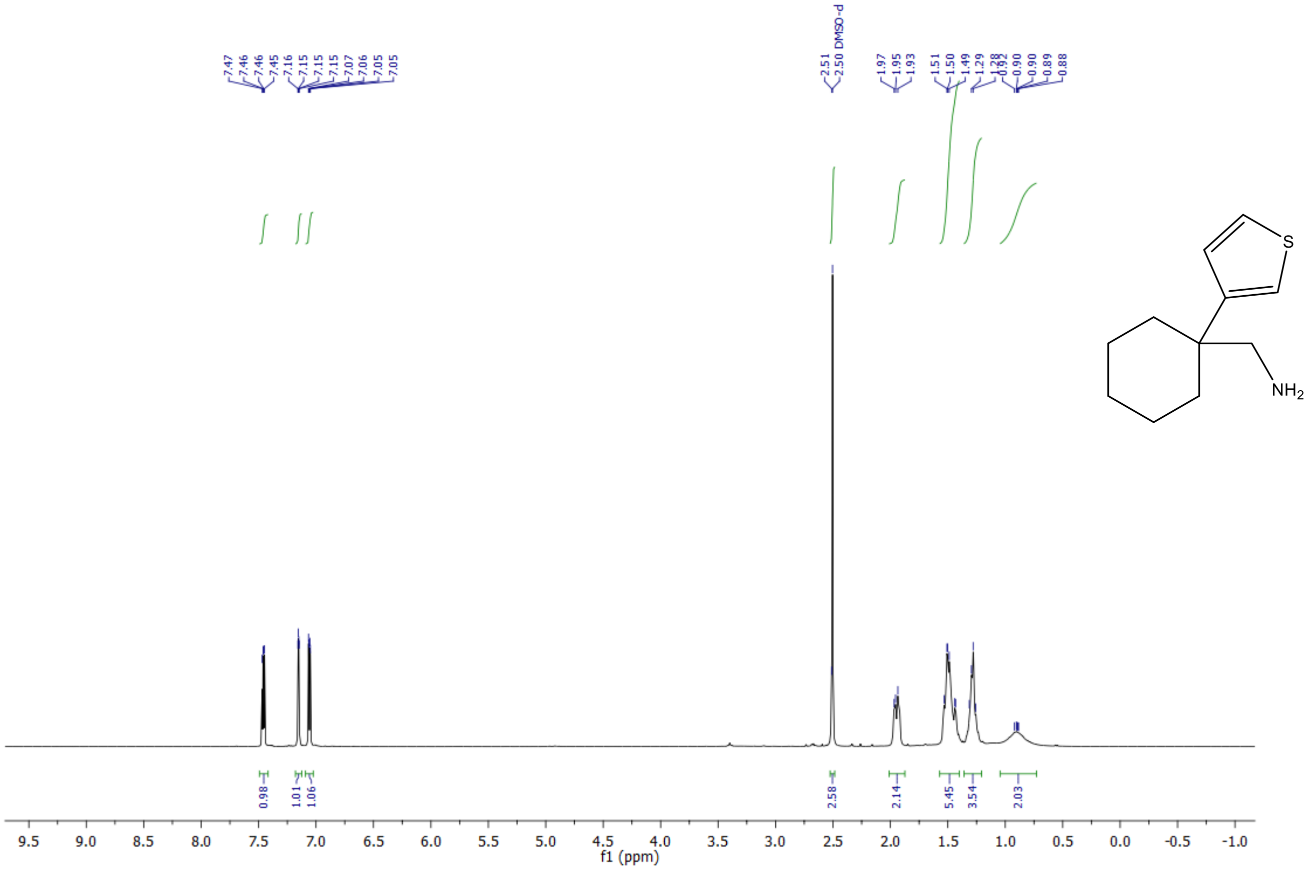

2-Methoxy-*N*-((4-(4-(trifluoromethyl)phenyl)tetrahydro-2*H*-pyran-4-yl)methyl)benzamide (22)

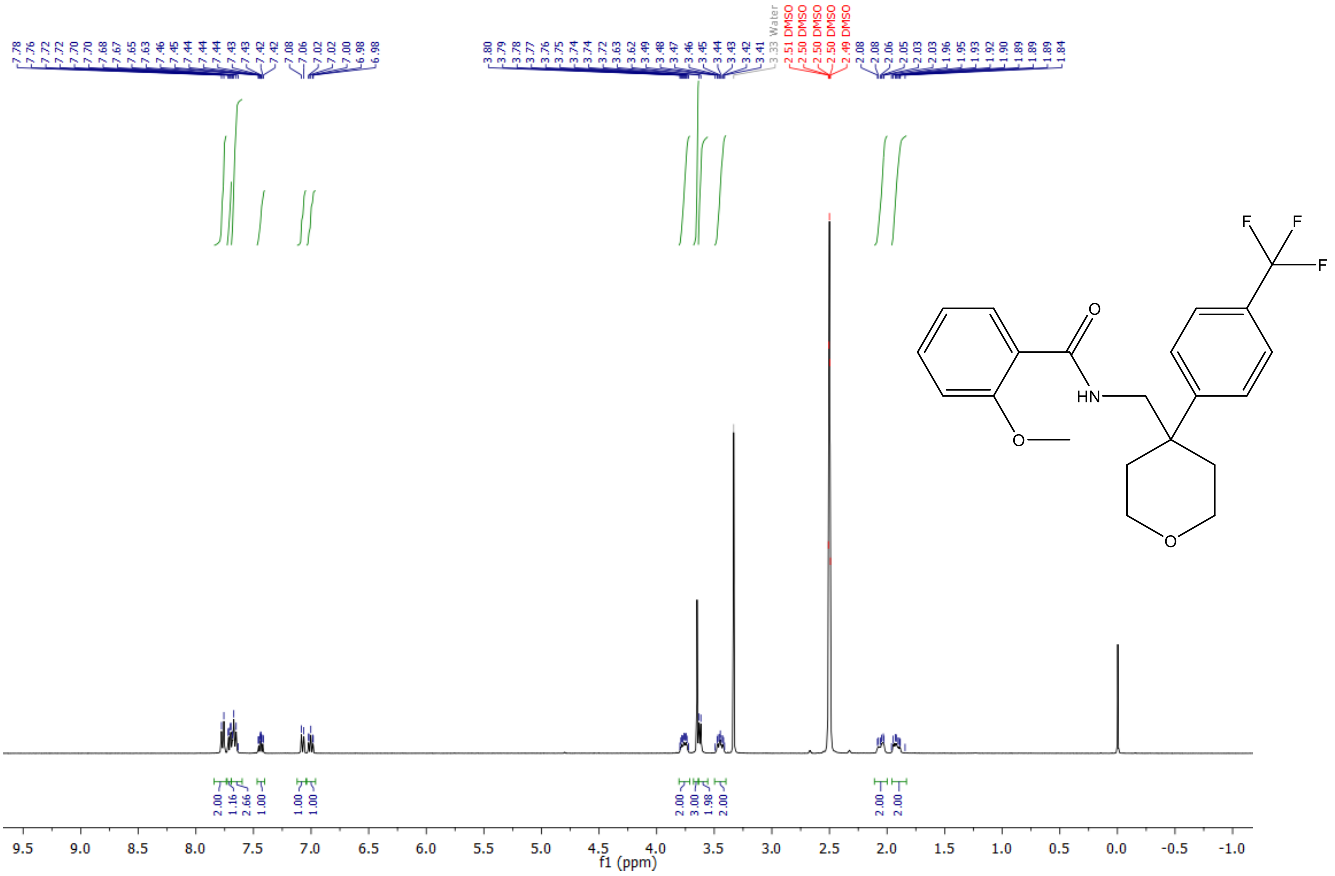
7.78 7.76 7.72 7.72 7.70 7.70 7.68 7.67 7.65 7.63 7.46 7.45 7.44 7.44 7.44 7.43 7.43 7.42 7.08 7.06 7.02 7.02 7.00 6.98 6.98
3.80 3.79 3.78 3.77 3.76 3.75 3.74 3.74 3.72 3.63 3.62 3.49 3.48 3.47 3.46 3.45 3.44 3.43 3.42 3.41
3.33 Water
2.51 DMSO 2.50 DMSO 2.50 DMSO 2.50 DMSO 2.49 DMSO
2.08 2.06 2.05 2.03 2.03 1.96 1.95 1.93 1.92 1.90 1.89 1.89 1.84
2.00 1.16 2.66 1.00 1.00 1.00
2.00 3.00 1.98 2.00
2.00 2.00
F
F
F
O
HN
O
O
9.5 9.0 8.5 8.0 7.5 7.0 6.5 6.0 5.5 5.0 4.5 4.0 3.5 3.0 2.5 2.0 1.5 1.0 0.5 0.0 -0.5 -1.0
f1 (ppm)

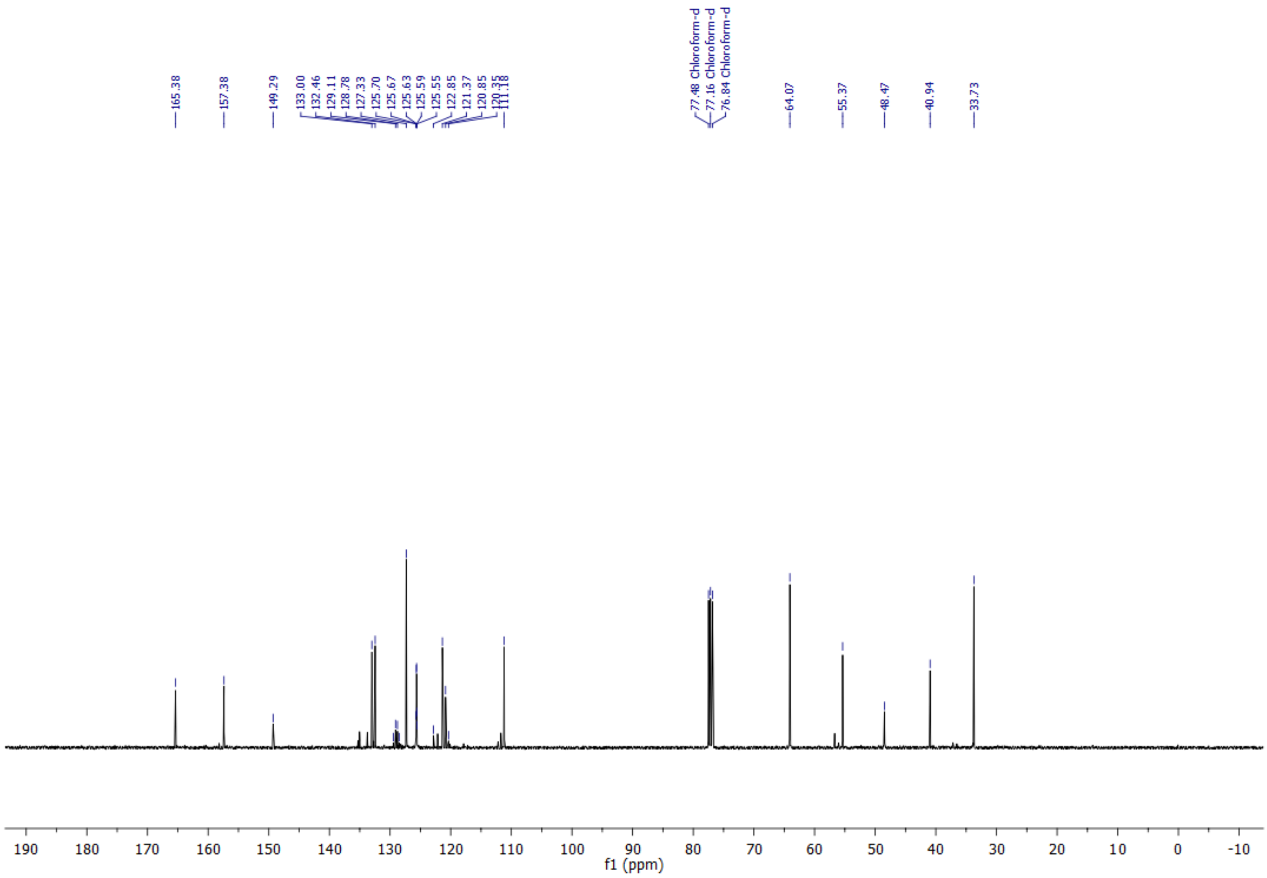
165.38
157.38
149.29
133.00 132.46 129.11 128.78 127.33 125.70 125.67 125.63 125.59 125.55 122.85 121.37 120.85 120.35 111.18
77.48 Chloroform-d 77.16 Chloroform-d 76.84 Chloroform-d
64.07
55.37
48.47
40.94
33.73
190 180 170 160 150 140 130 120 110 100 90 80 70 60 50 40 30 20 10 0 -10
f1 (ppm)

N-((4-(3,4-Dimethoxyphenyl)tetrahydro-2H-pyran-4-yl)methyl)-2-methoxybenzamide (23)

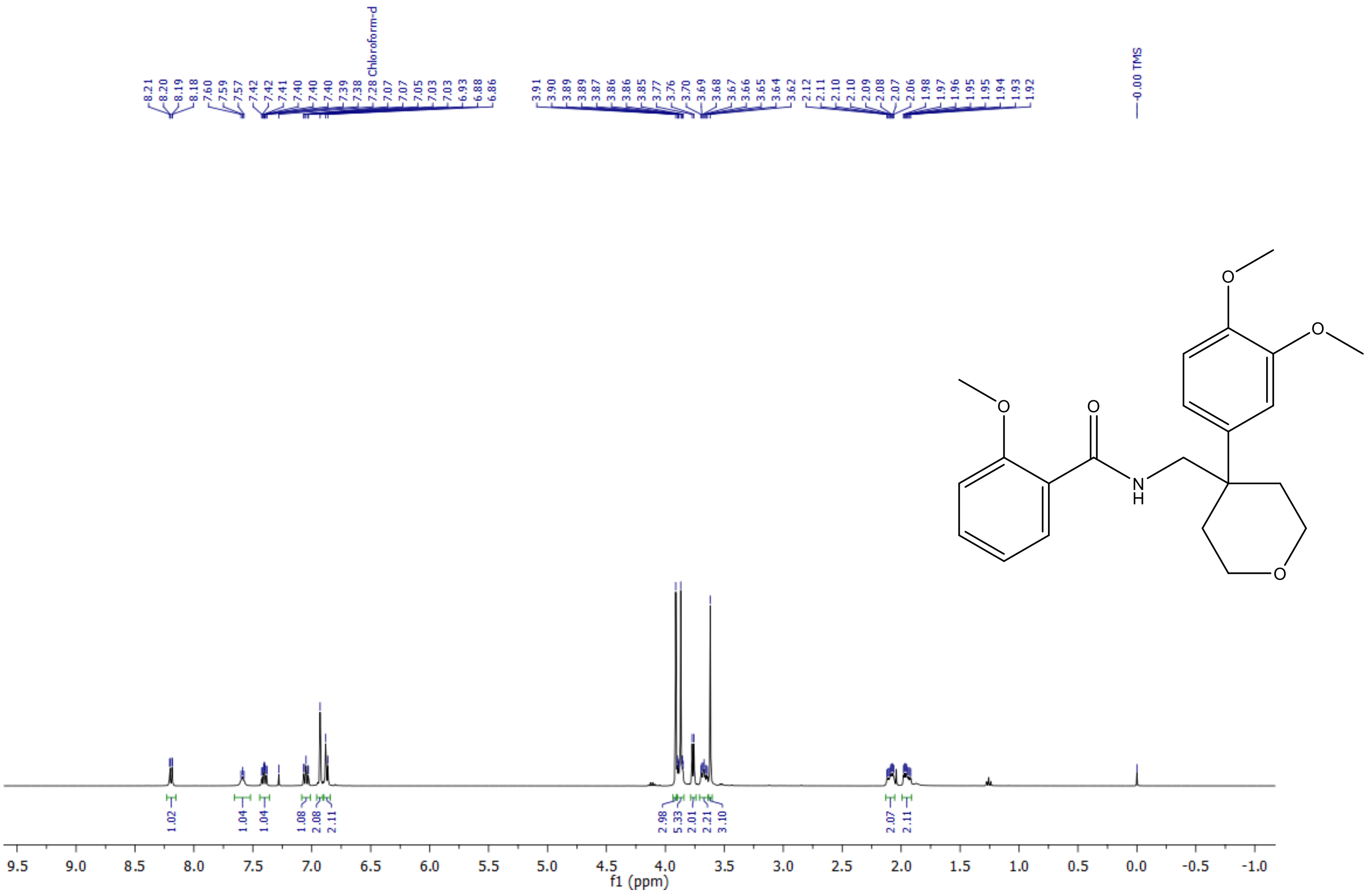

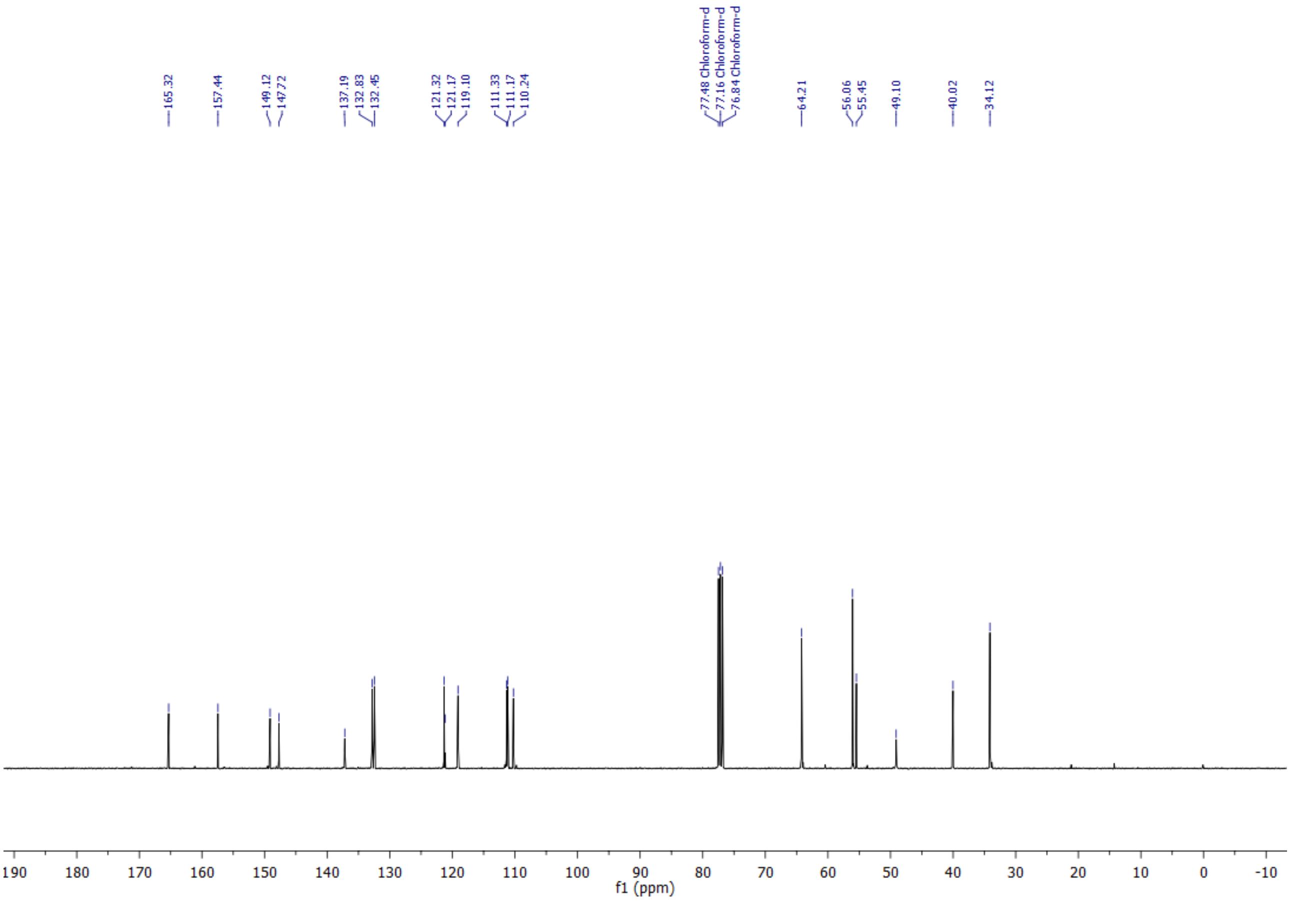

2-Methoxy-N-((4-(4-methoxyphenyl)tetrahydro-2H-pyran-4-yl)methyl)benzamide (24)

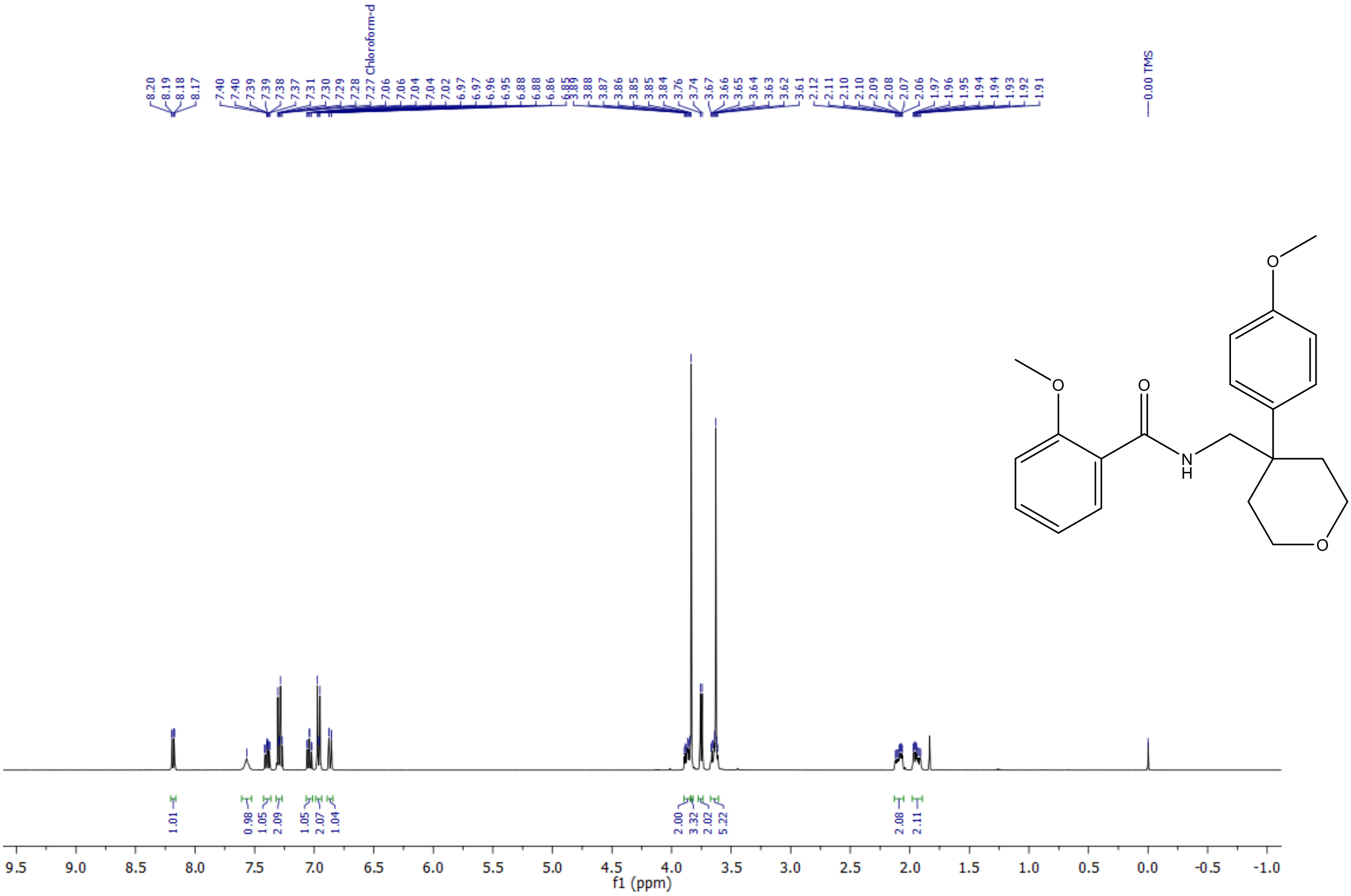

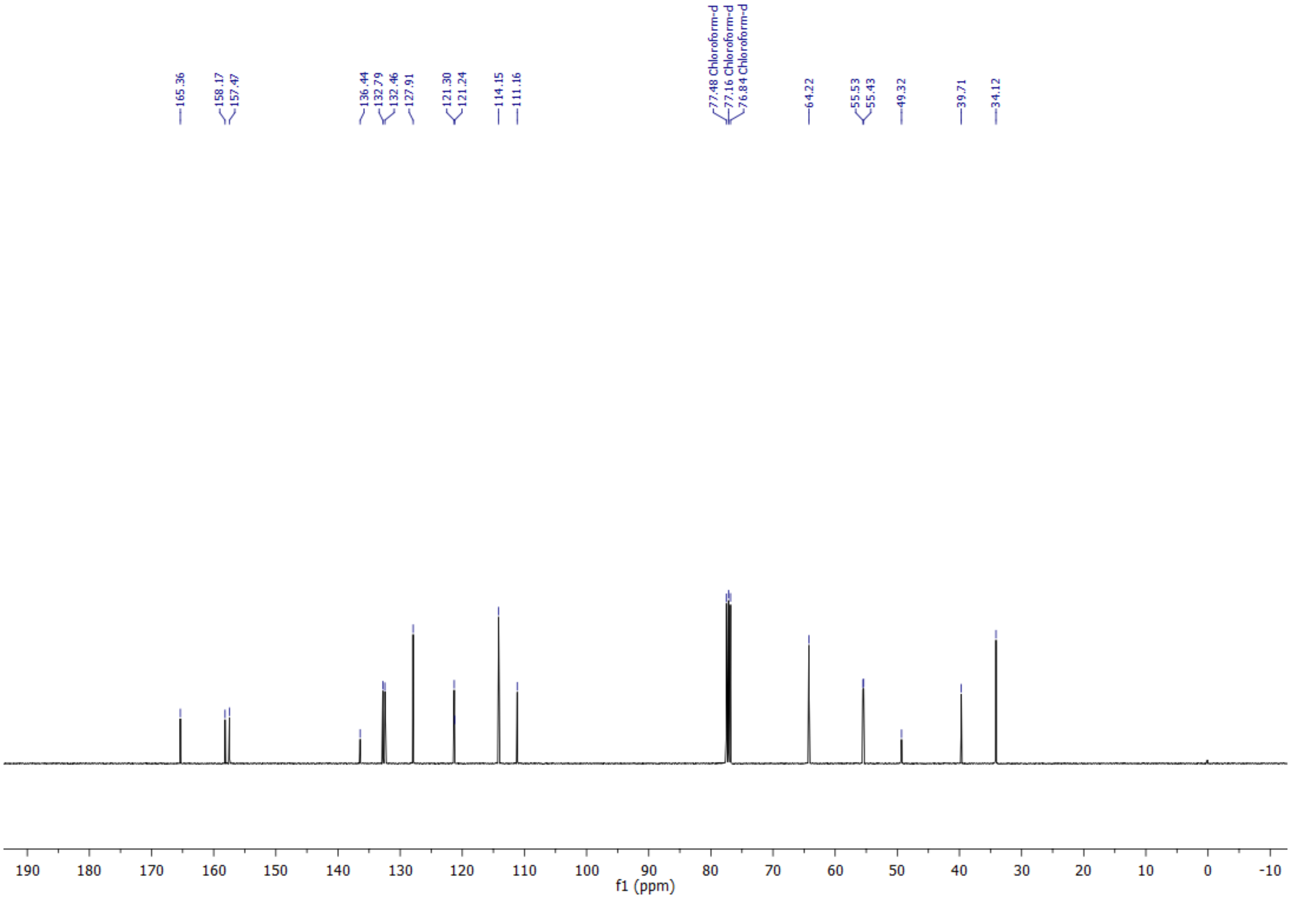

2-Methoxy-*N*-((4-(p-tolyl)tetrahydro-2*H*-pyran-4-yl)methyl)benzamide (25)

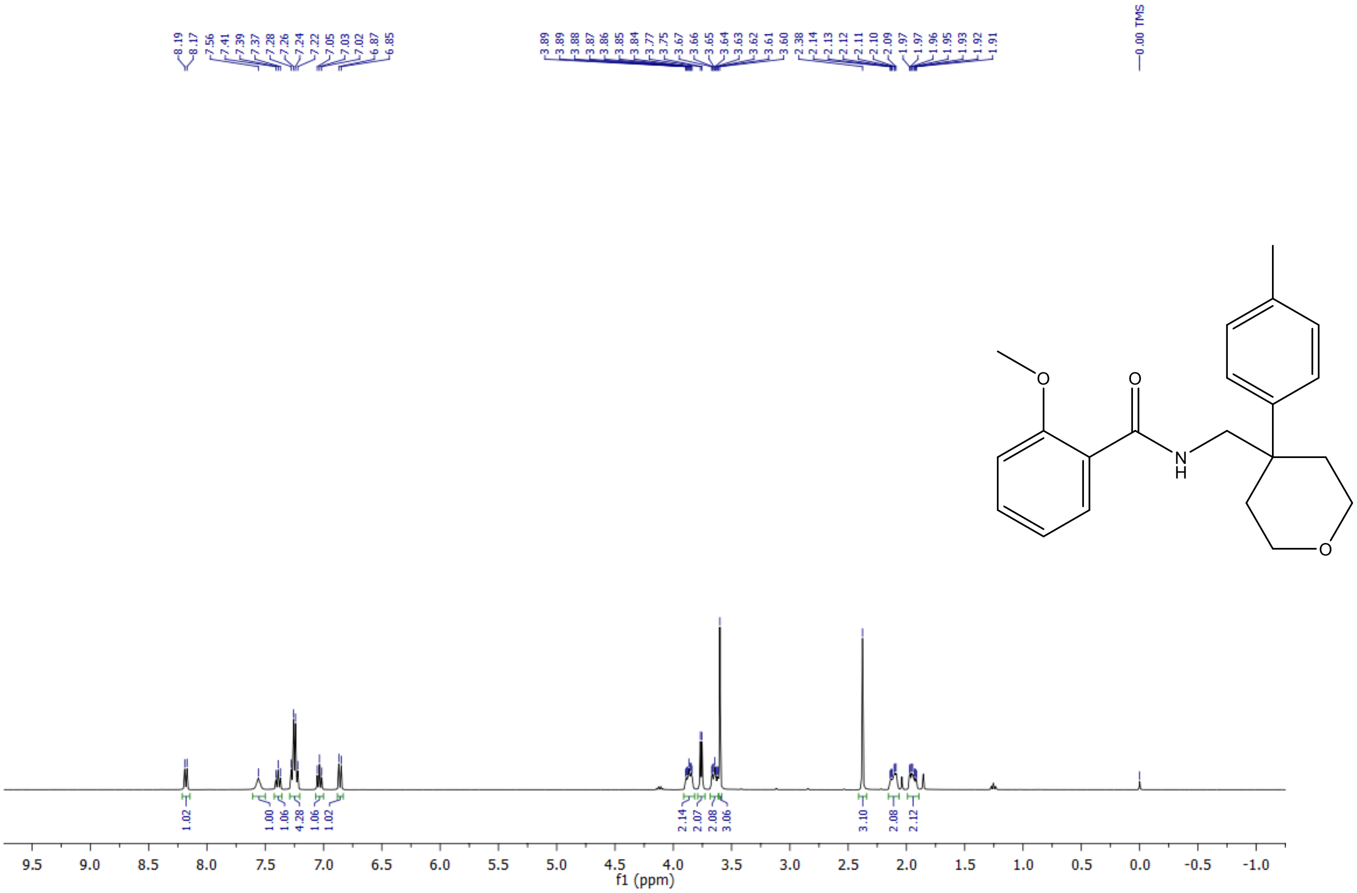

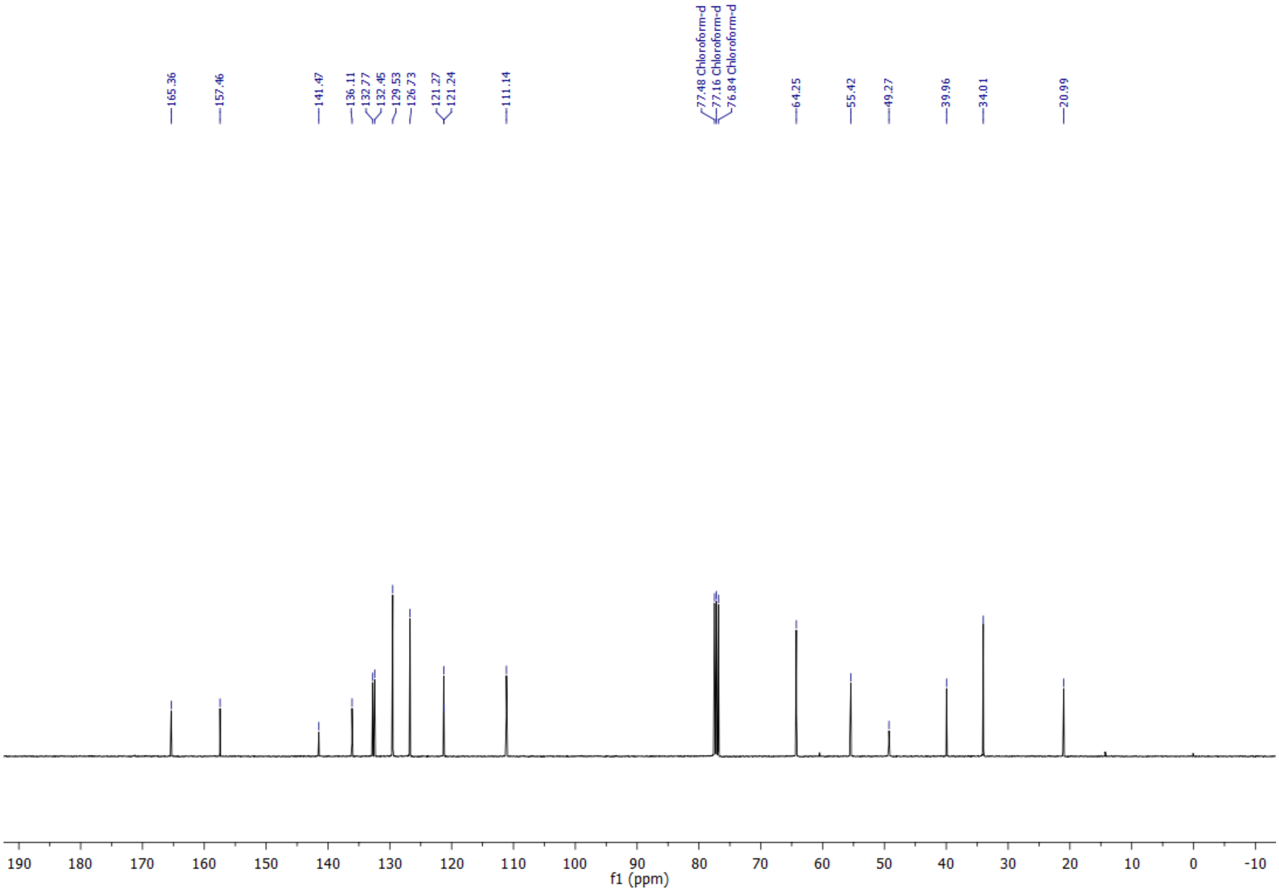

***N*-((4-(Benzo[*d*][1,3]dioxol-5-yl)tetrahydro-2*H*-pyran-4-yl)methyl)-2-methoxybenzamide (26)**

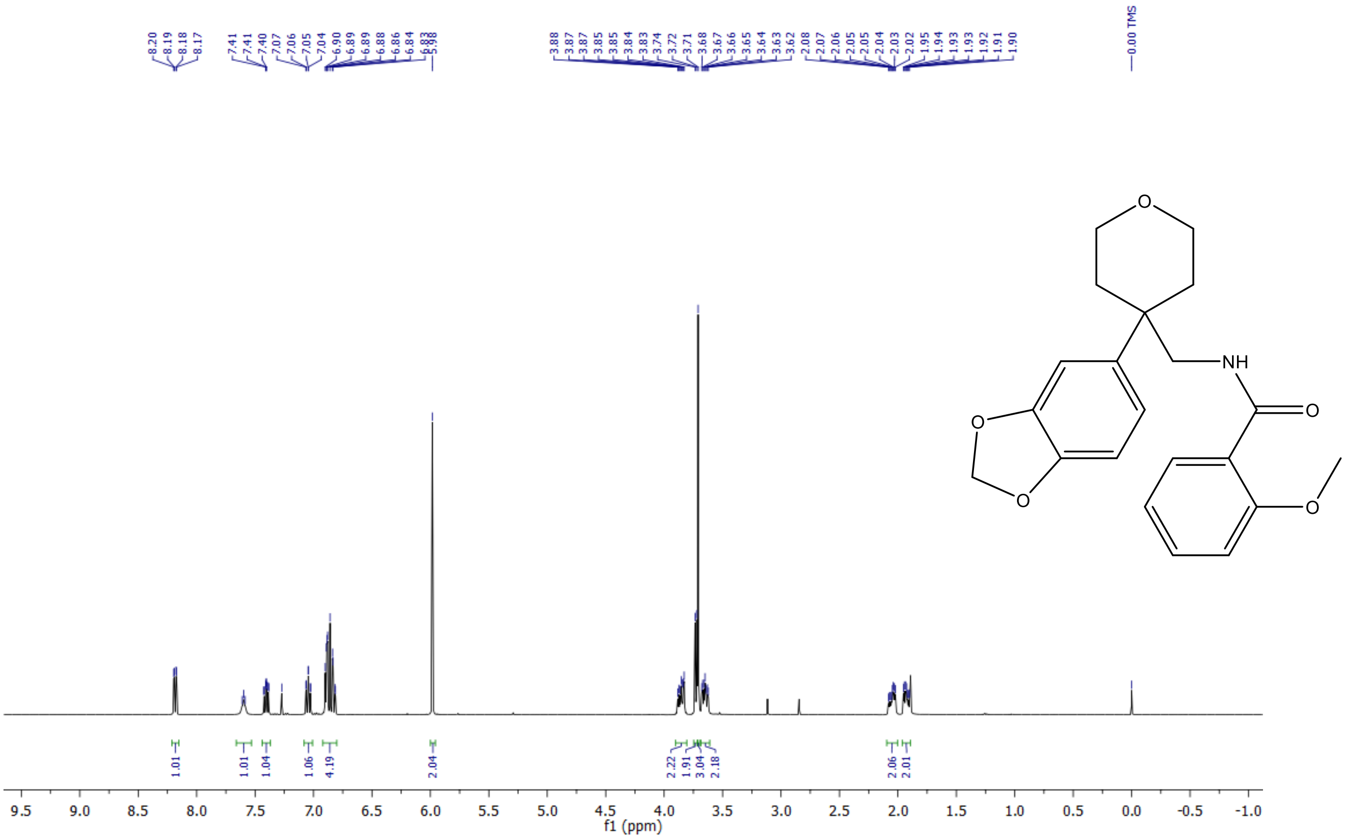

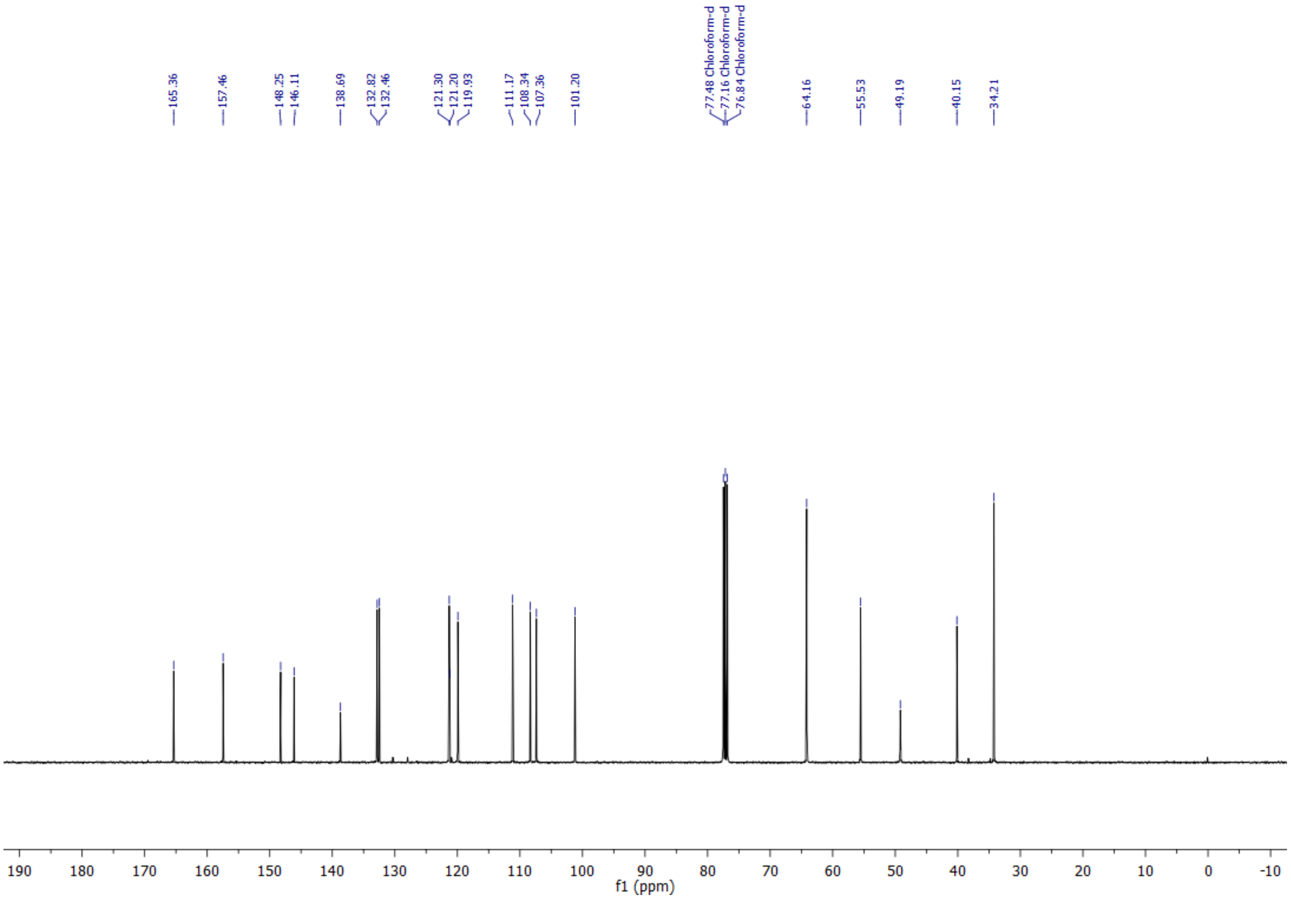

2-Methoxy-*N*-((4-(2-methoxyphenyl)tetrahydro-2*H*-pyran-4-yl)methyl)benzamide (27)

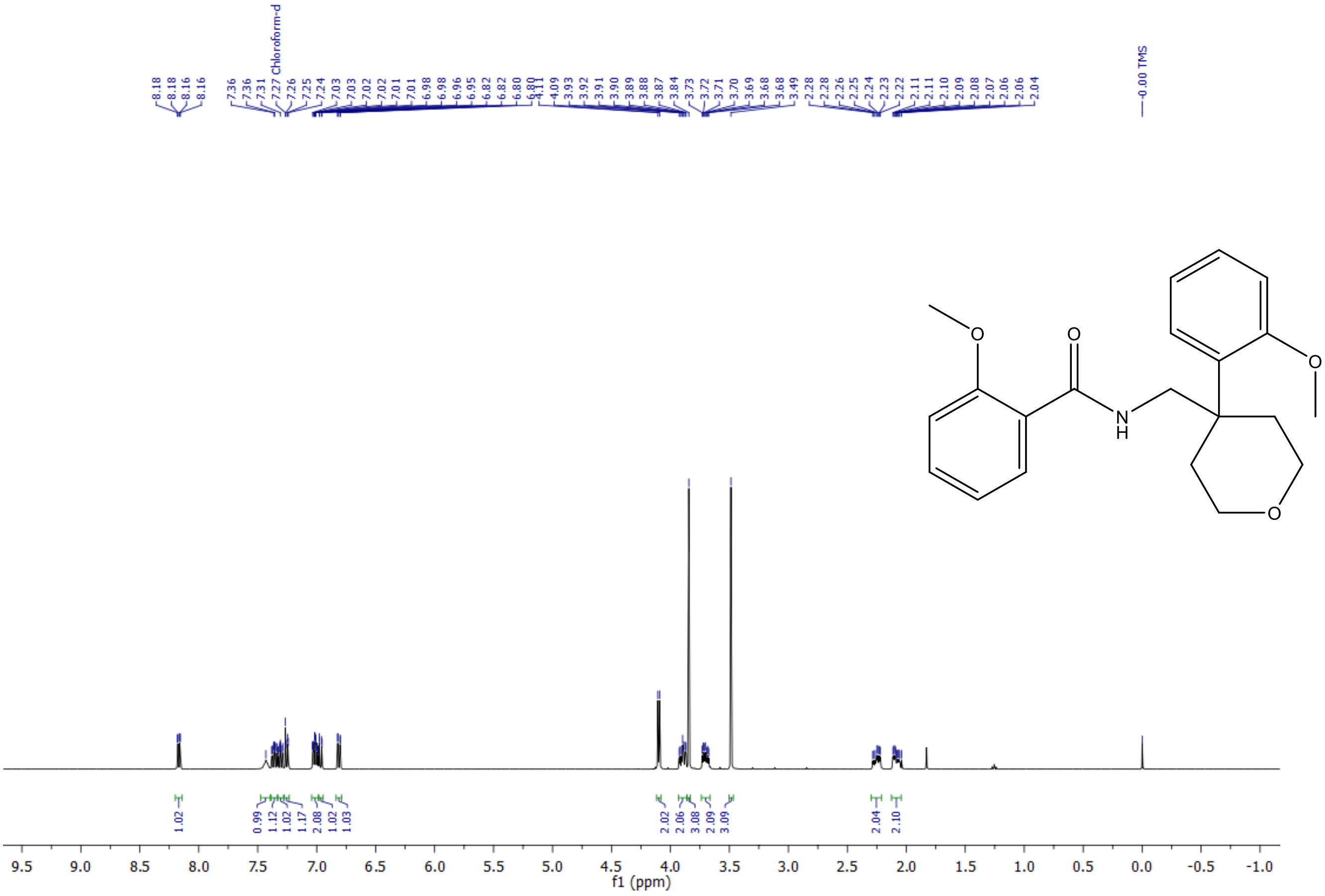

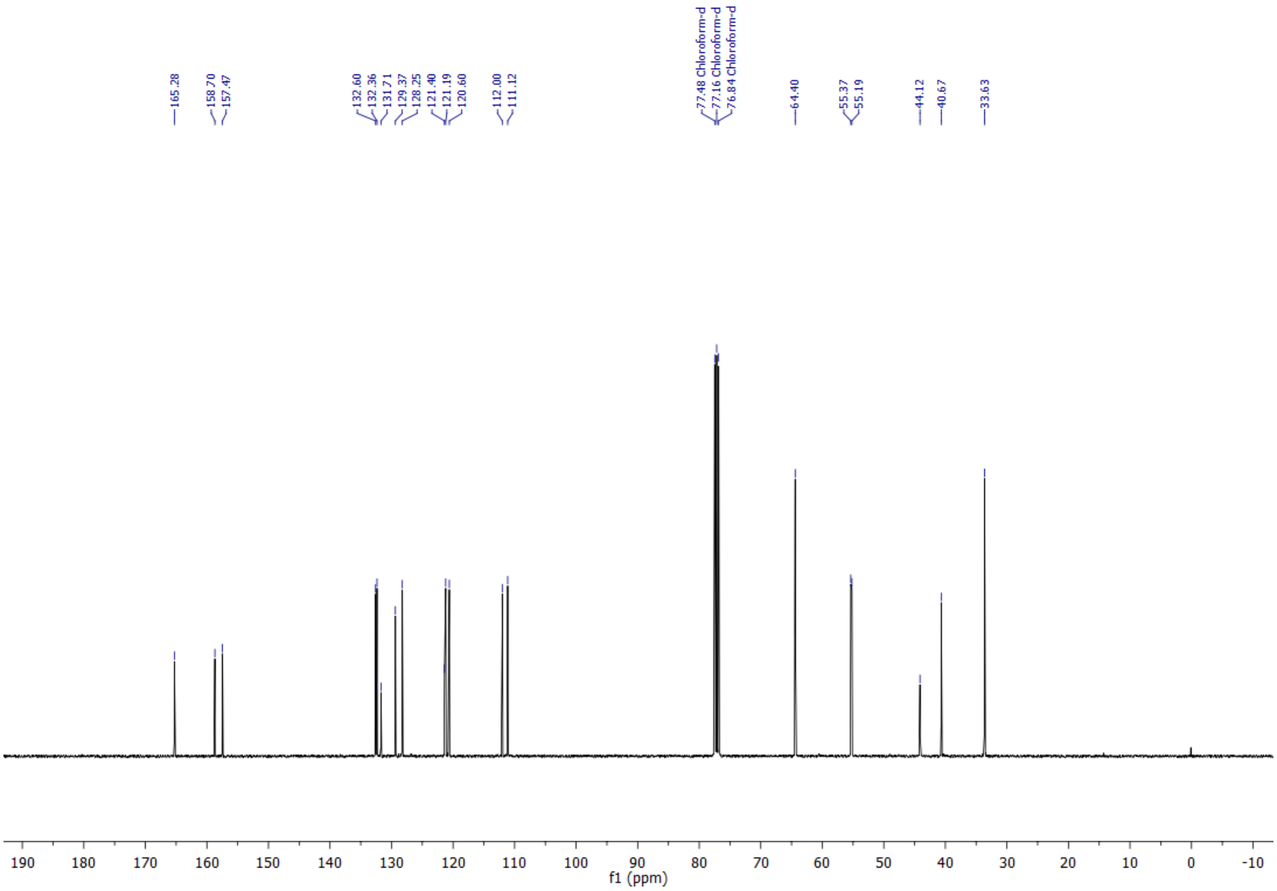

2-Methoxy-*N*-((4-(3-methoxyphenyl)tetrahydro-2*H*-pyran-4-yl)methyl)benzamide (28)

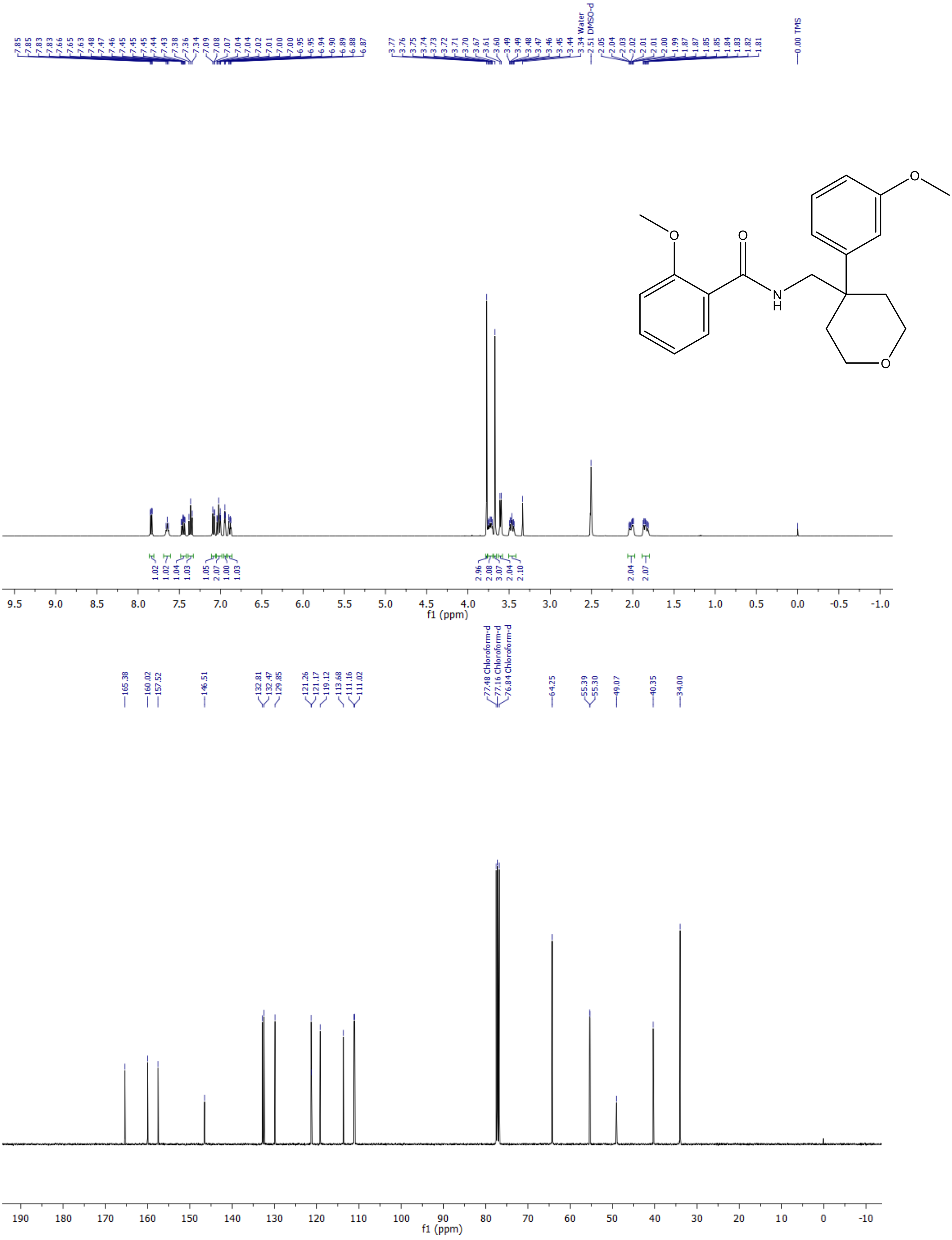

2-Methoxy-*N*-((4-(*m*-tolyl)tetrahydro-2*H*-pyran-4-yl)methyl)benzamide (29)

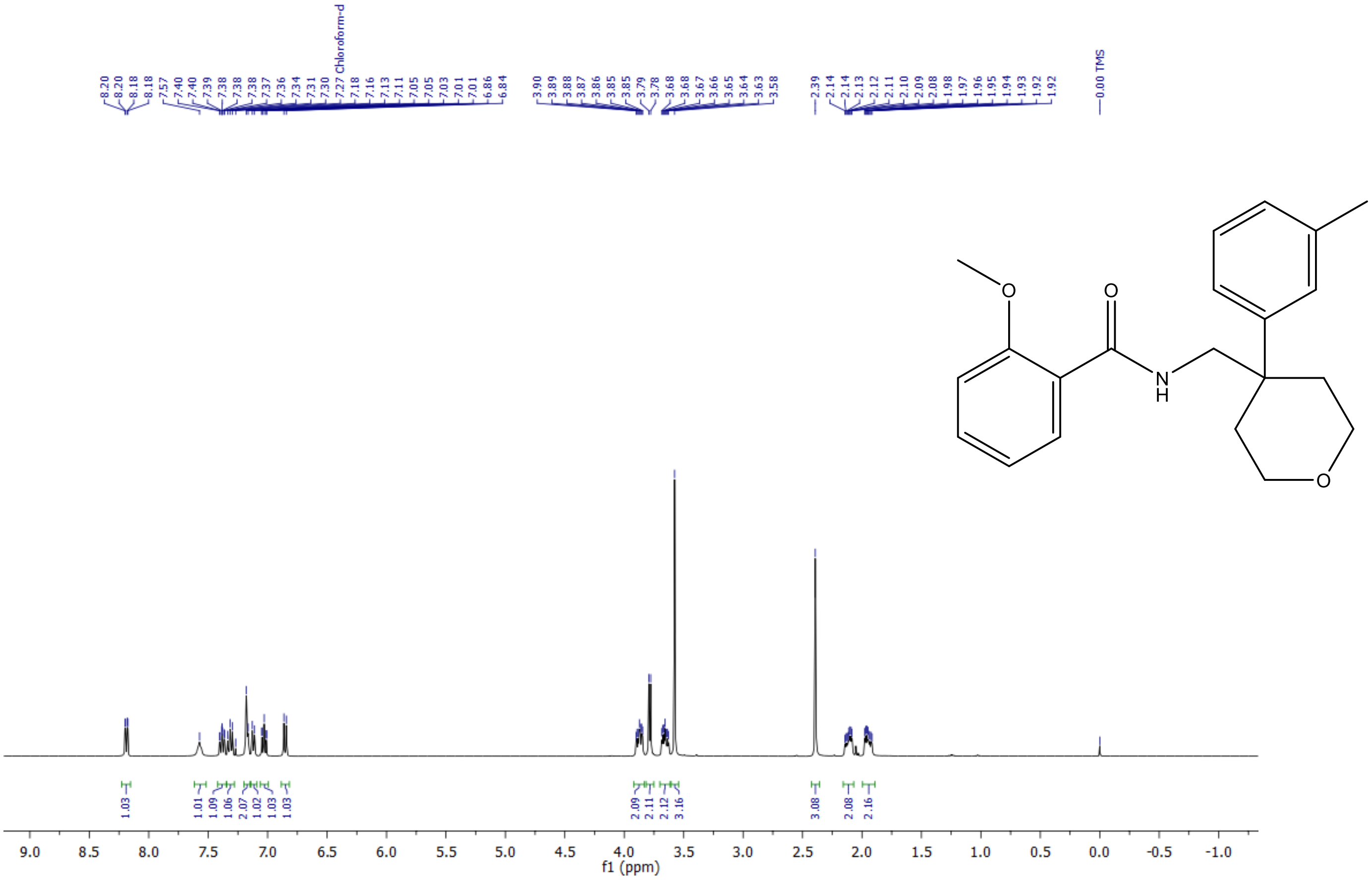

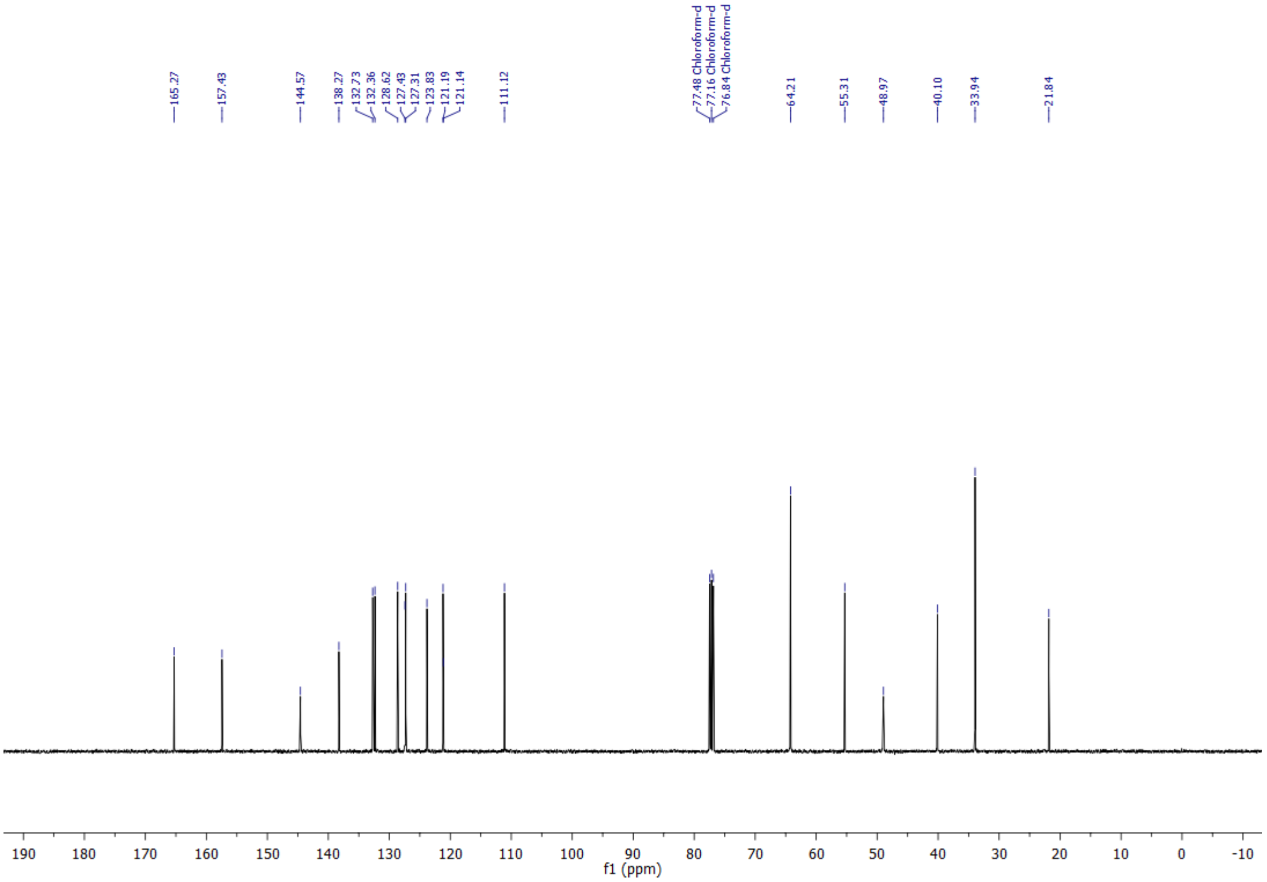

2,6-Dimethoxy-*N*-((4-phenyltetrahydro-2*H*-pyran-4-yl)methyl)benzamide (30)

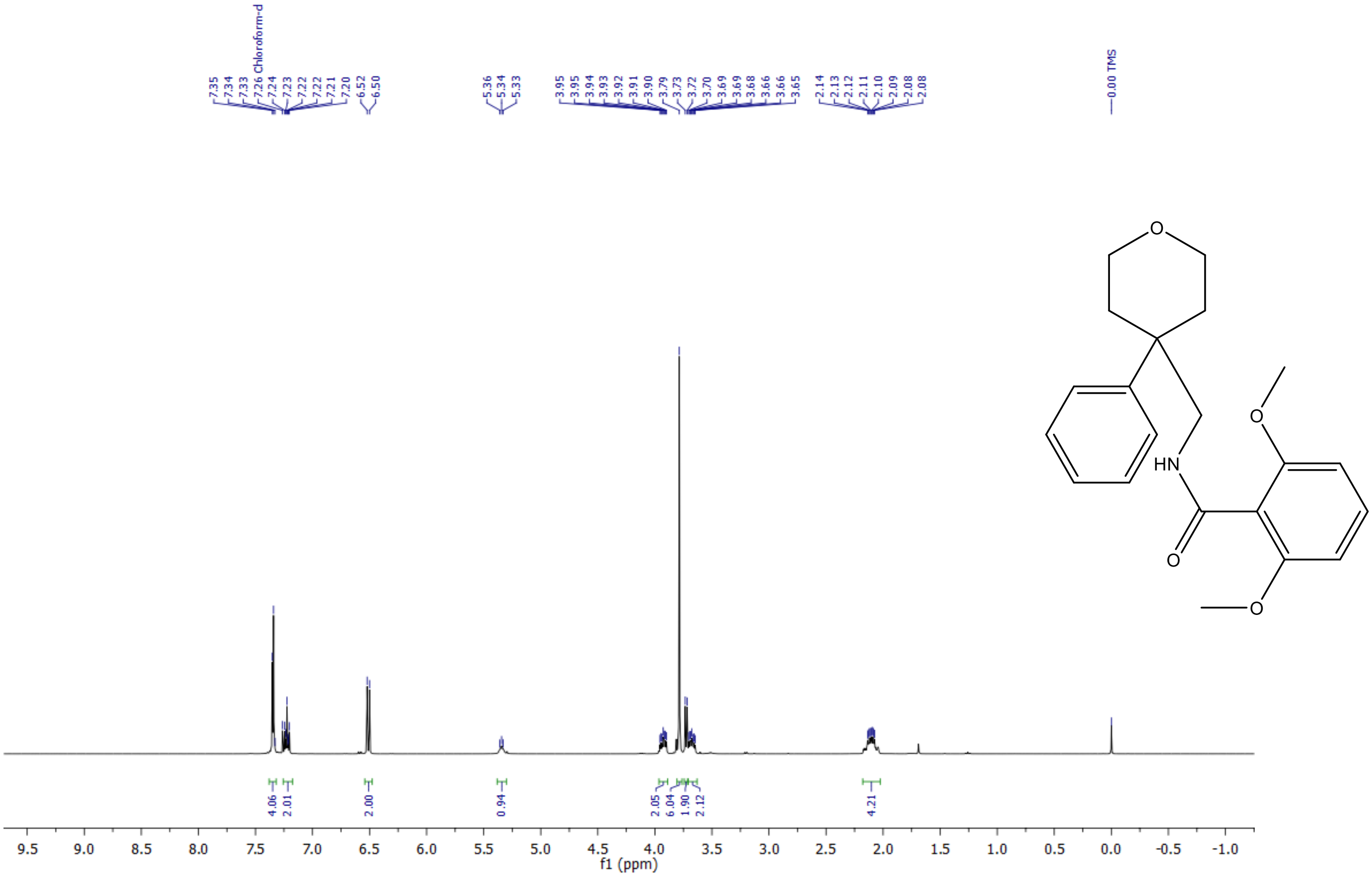

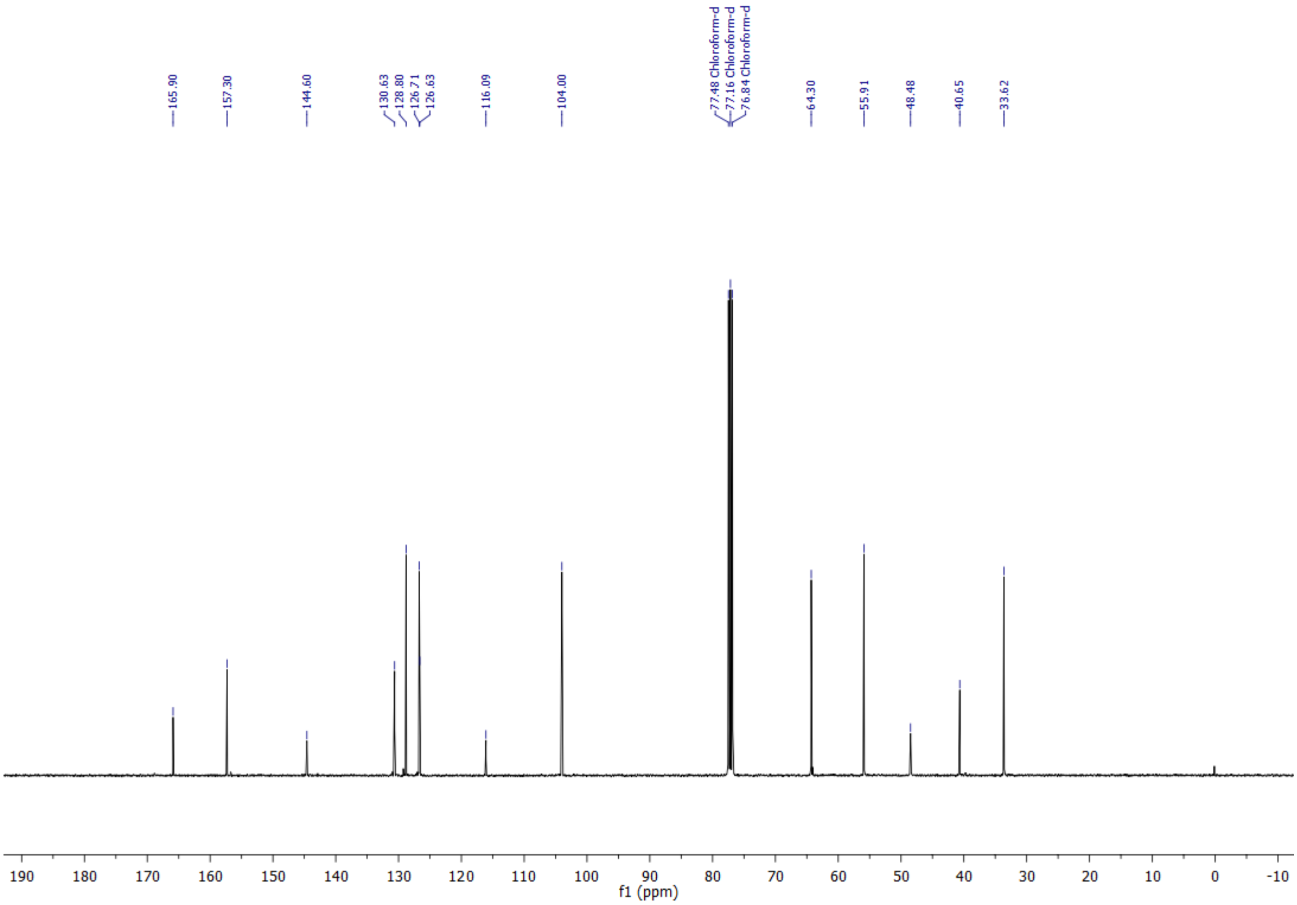

***N*-((4-Phenyltetrahydro-2*H*-pyran-4-yl)methyl)thiophene-3-carboxamide (31)**

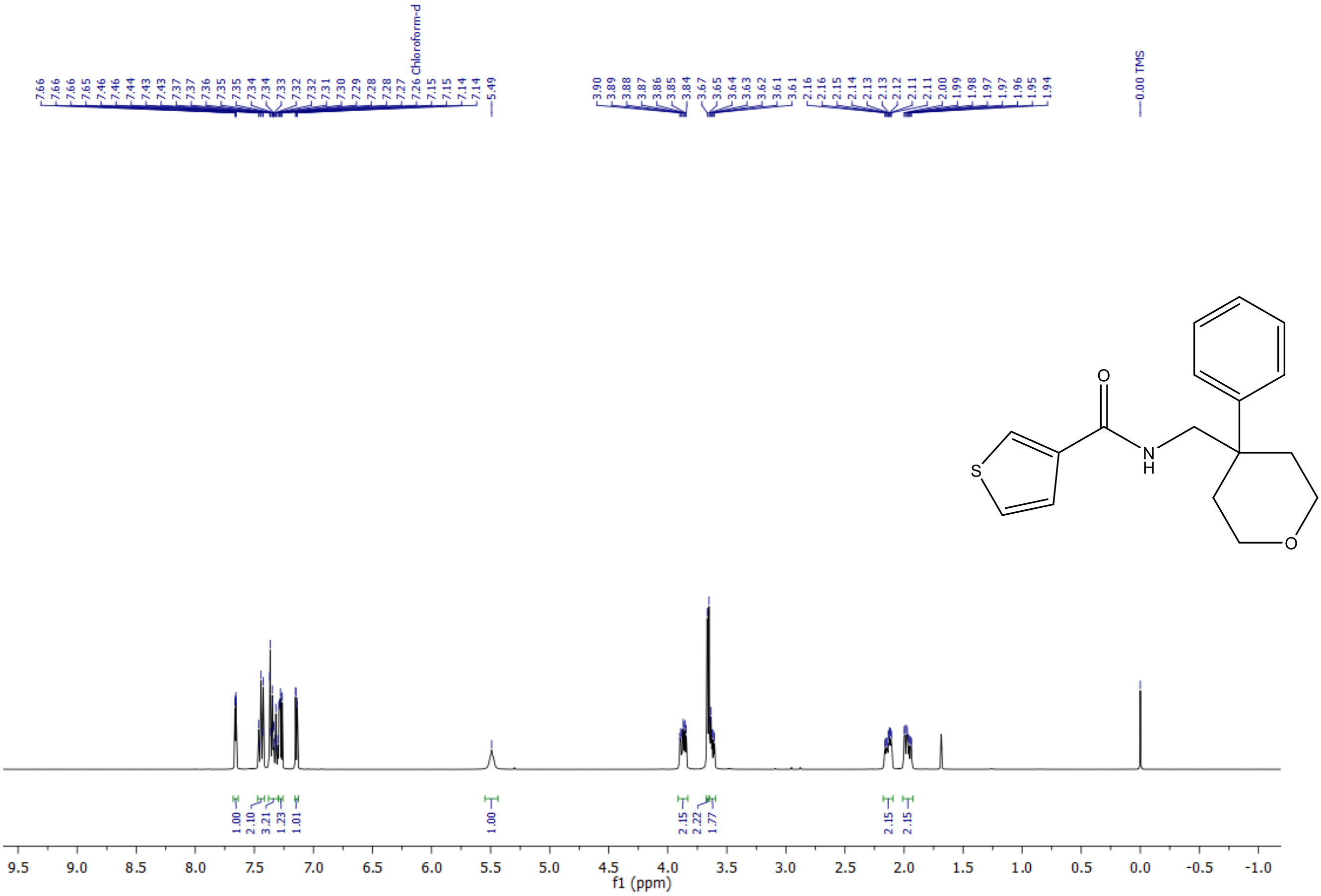

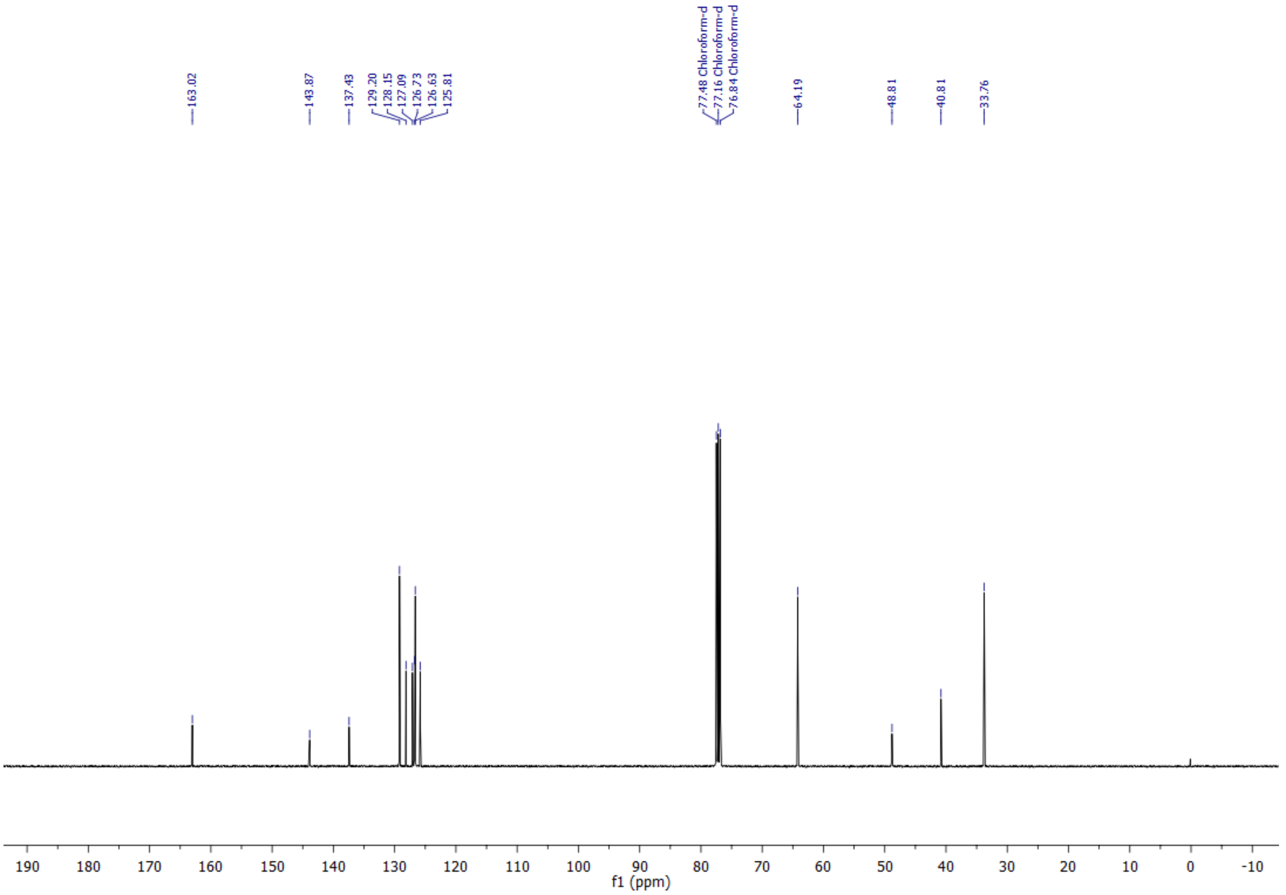

N-((4-Phenyltetrahydro-2H-pyran-4-yl)methyl)furan-3-carboxamide (32)

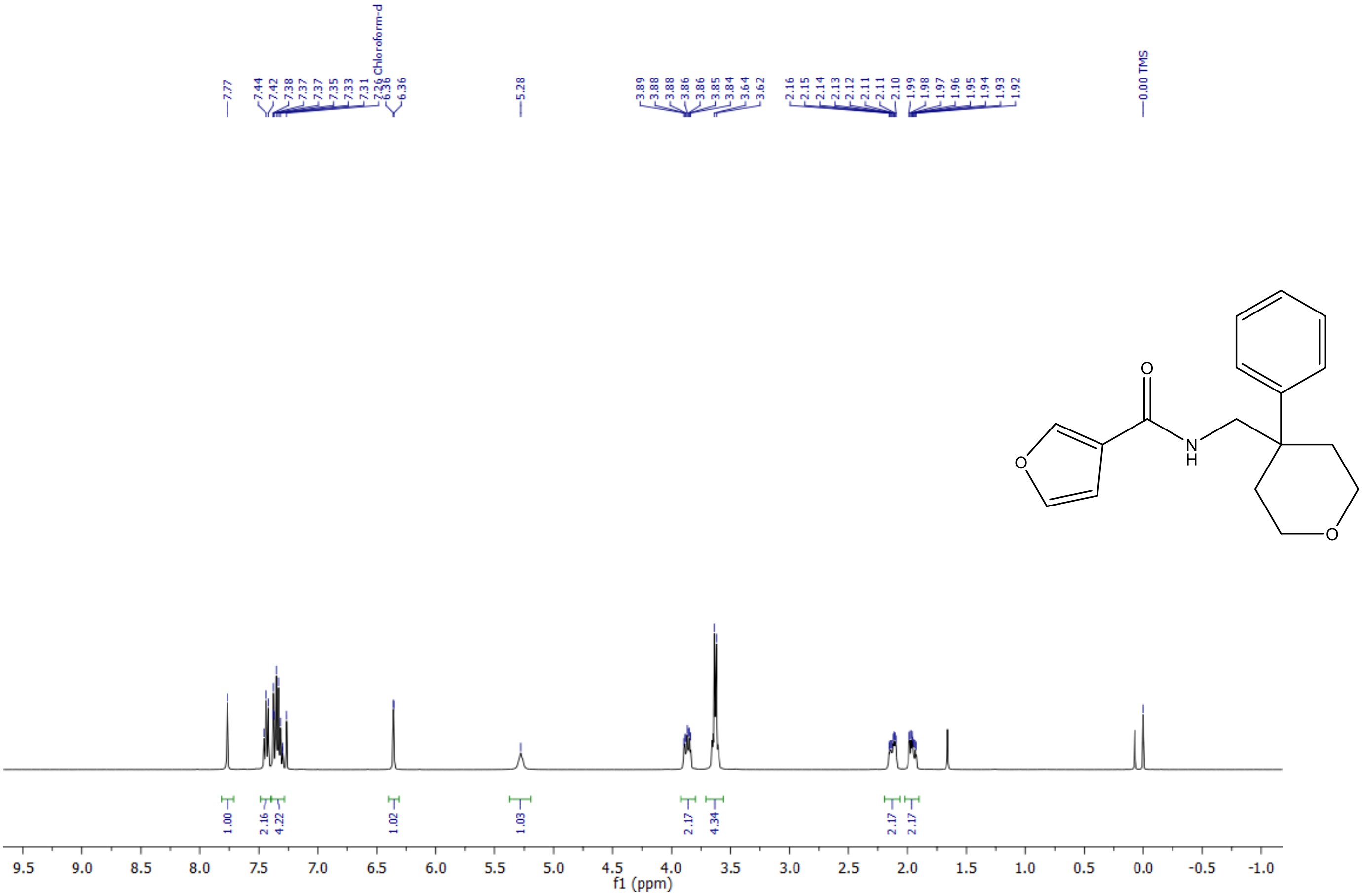

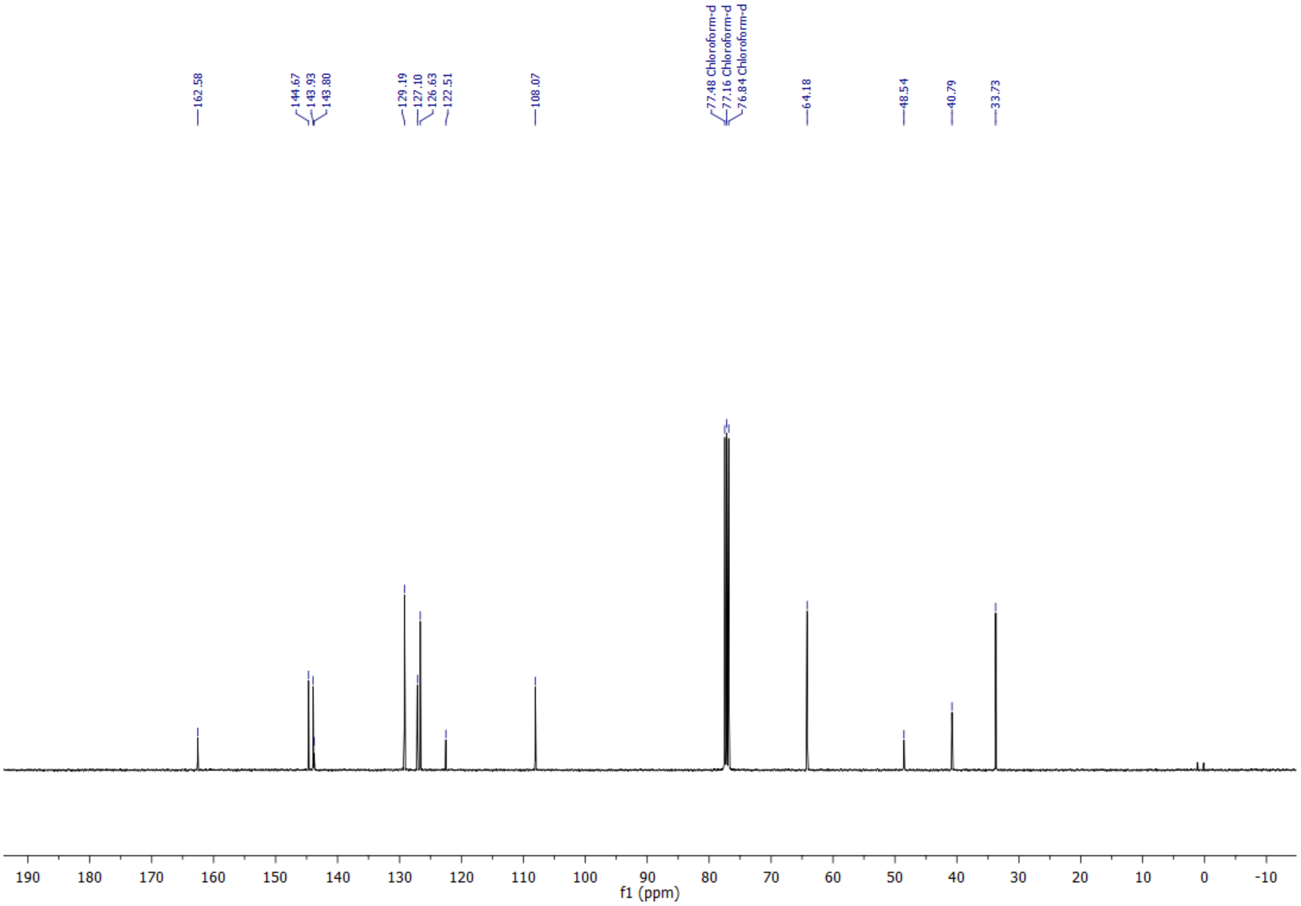

2-(2-Methoxyphenyl)-*N*-((4-phenyltetrahydro-2*H*-pyran-4-yl)methyl)acetamide (33)

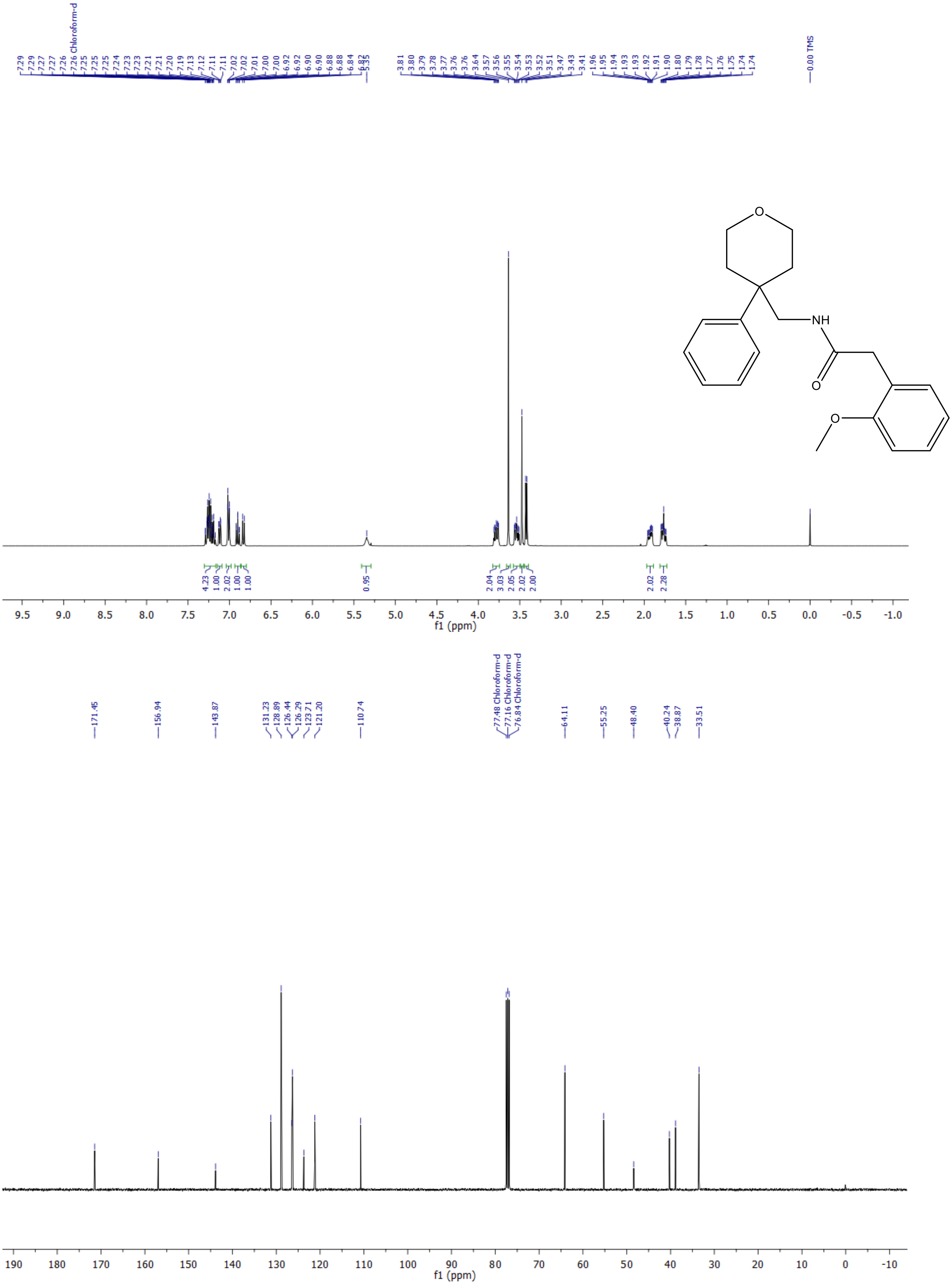

2-Methoxy-5-methyl-*N*-((4-phenyltetrahydro-2*H*-pyran-4-yl)methyl)benzamide (34)

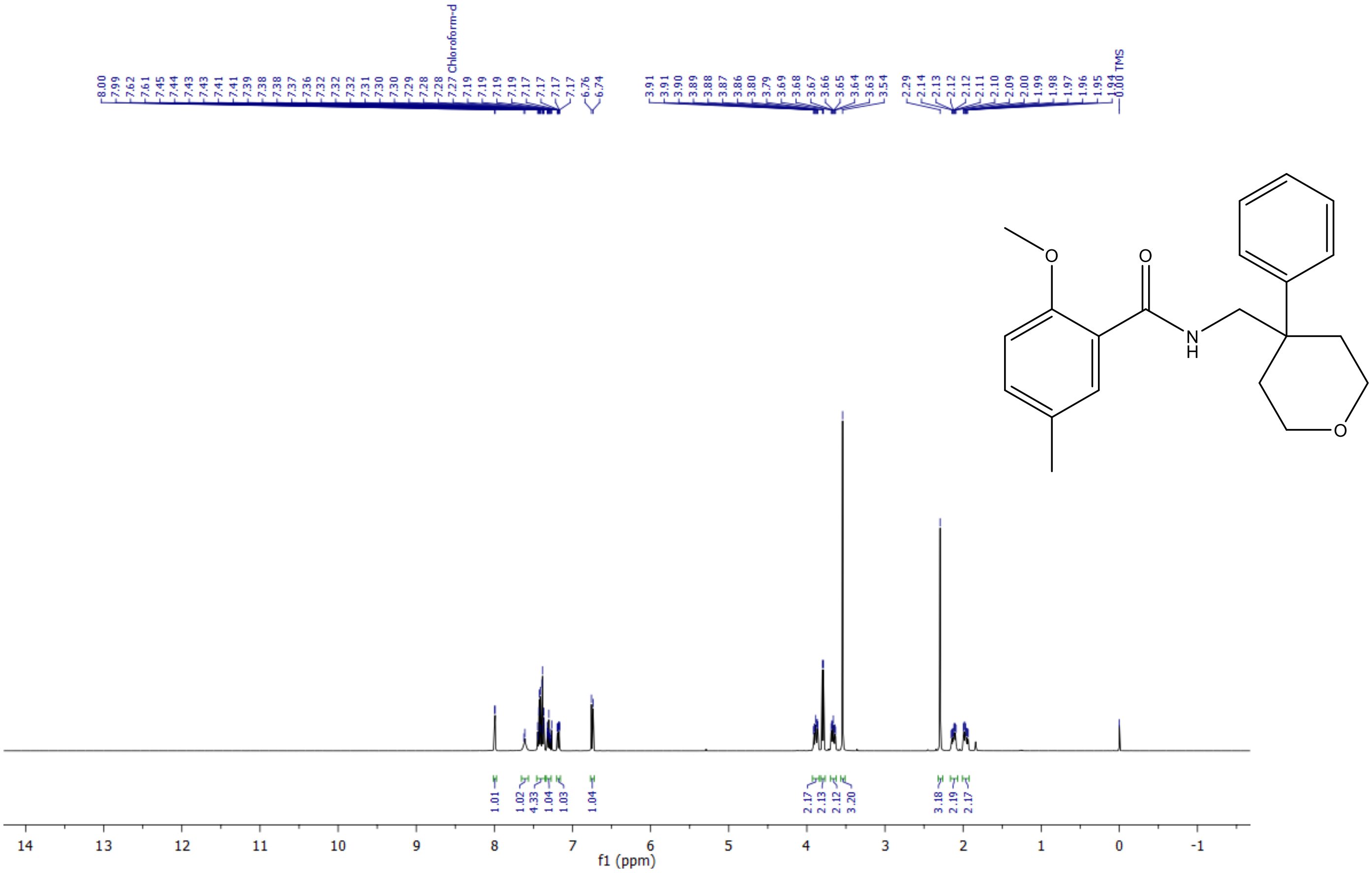

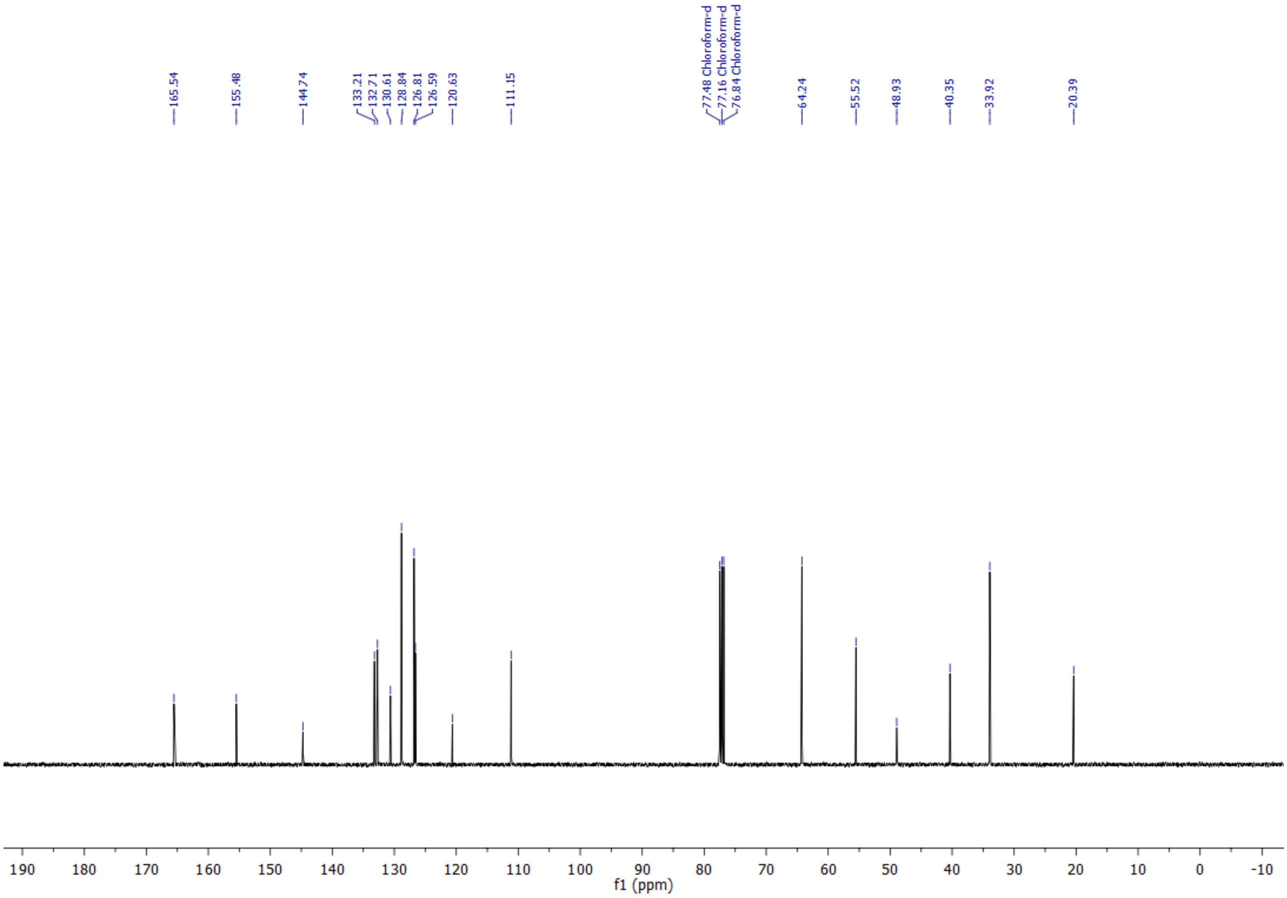

2-Ethoxy-*N*-((4-phenyltetrahydro-2*H*-pyran-4-yl)methyl)benzamide (35)

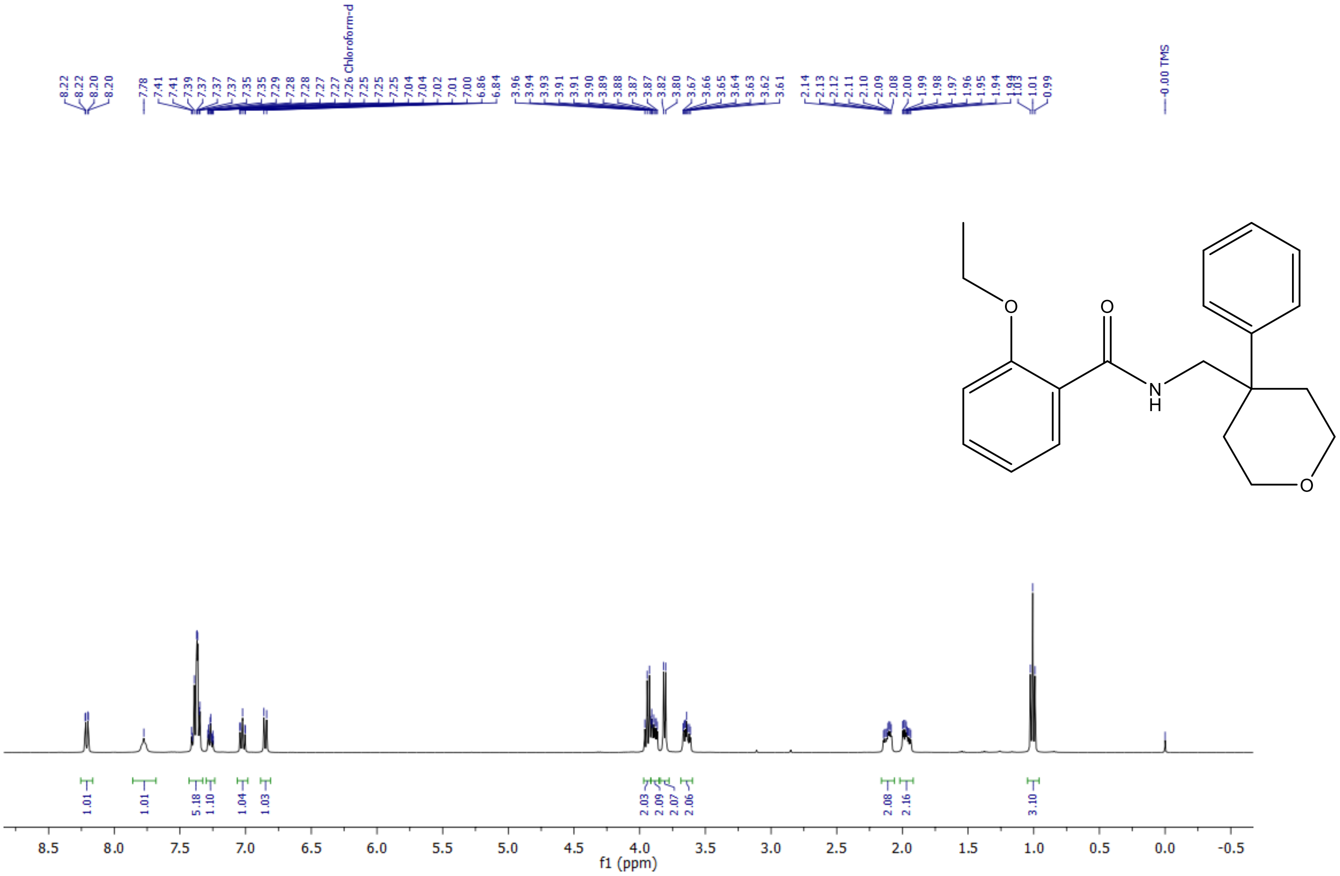

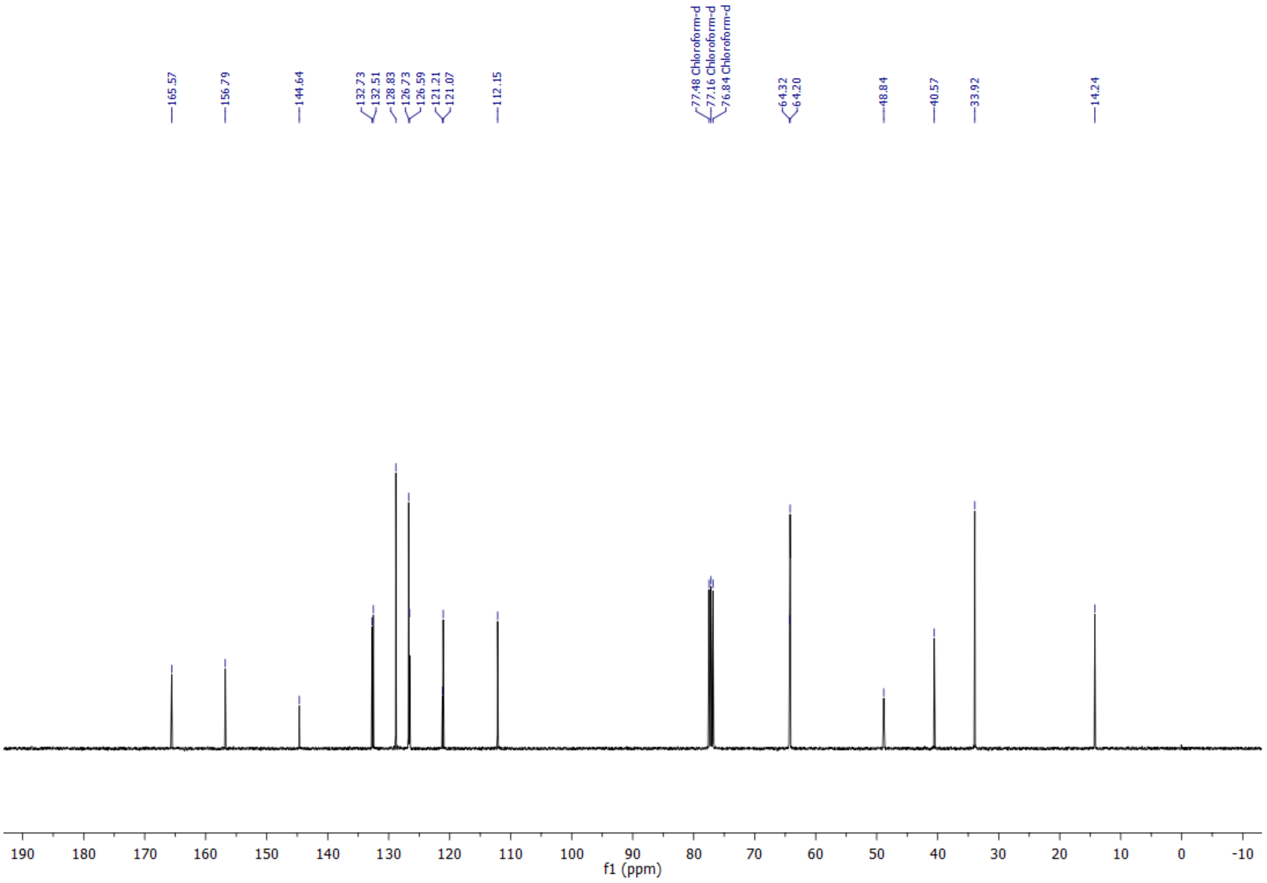

2-Methoxy-4-methyl-*N*-((4-phenyltetrahydro-2*H*-pyran-4-yl)methyl)benzamide (36)

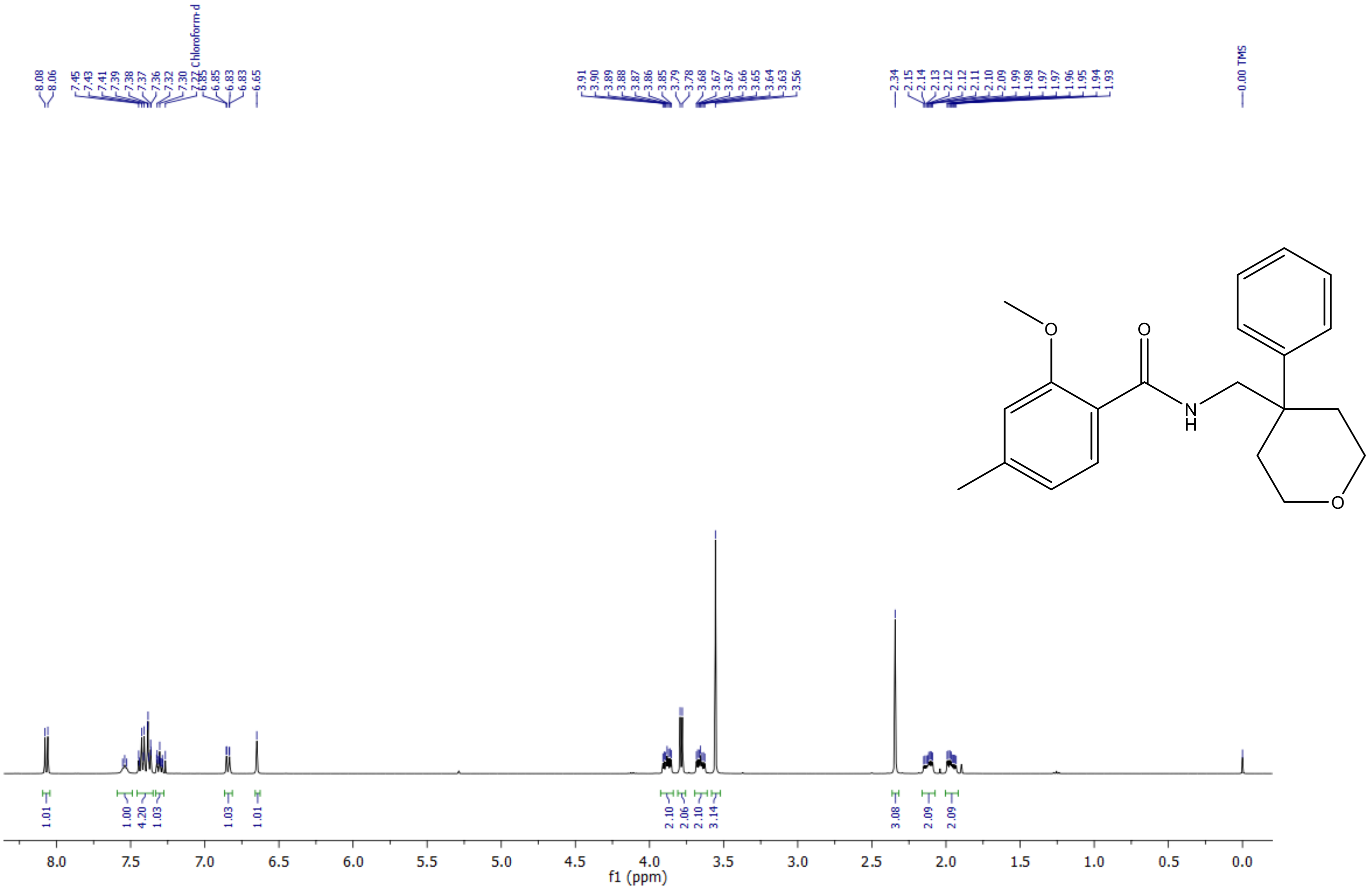

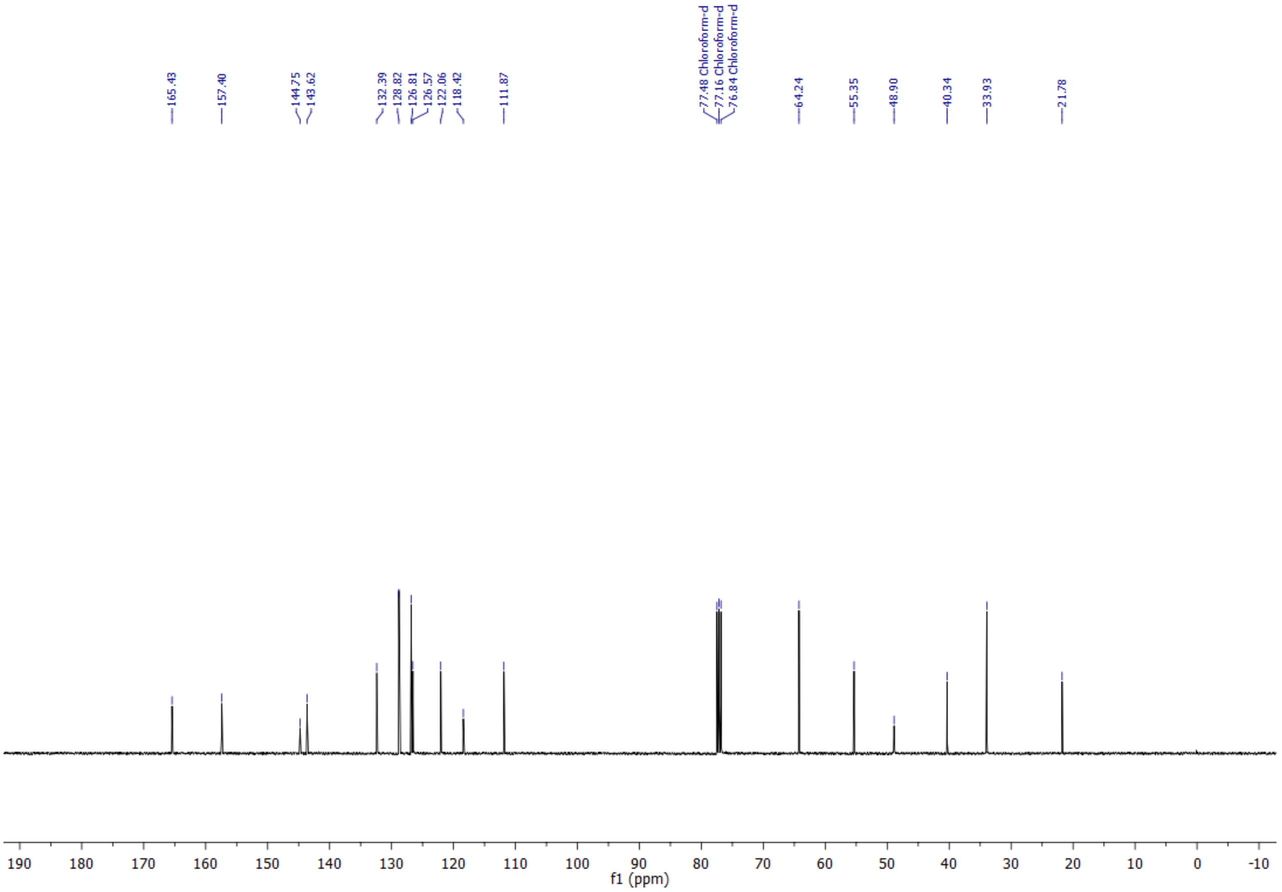

5-Fluoro-2-methoxy-*N*-((4-phenyltetrahydro-2*H*-pyran-4-yl)methyl)benzamide (37)

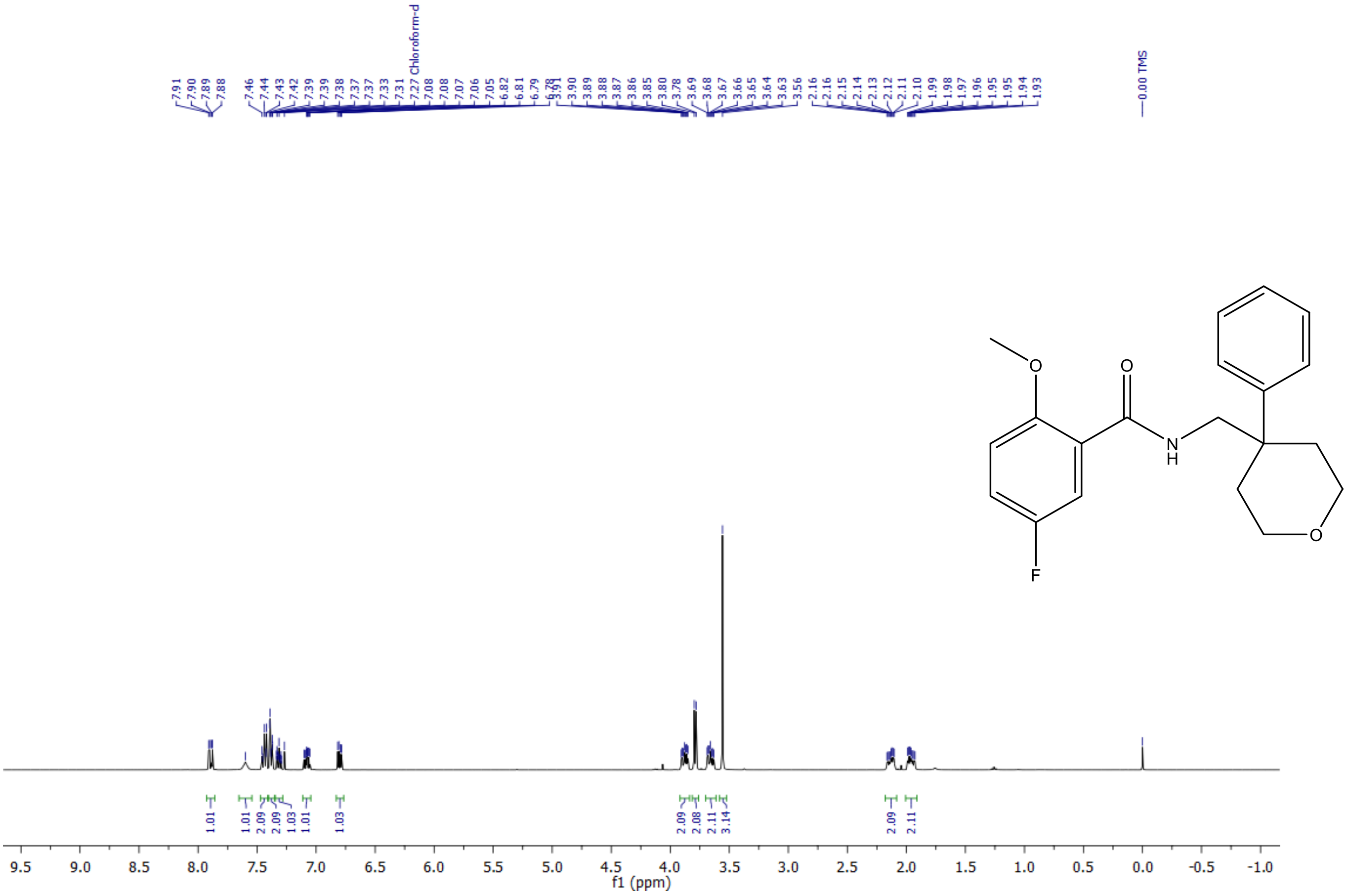

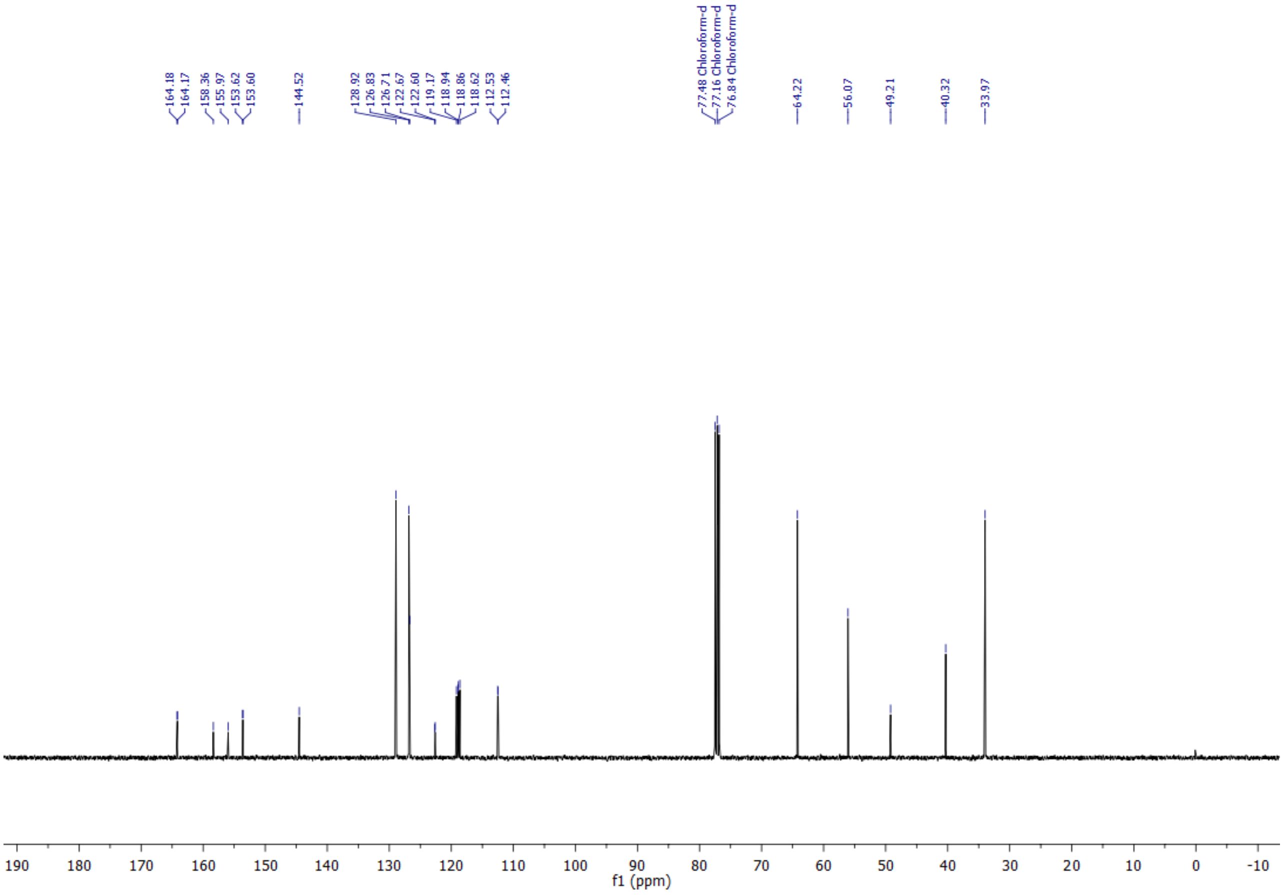

4-Phenyltetrahydro-2*H*-pyran-4-carbonitrile (86)

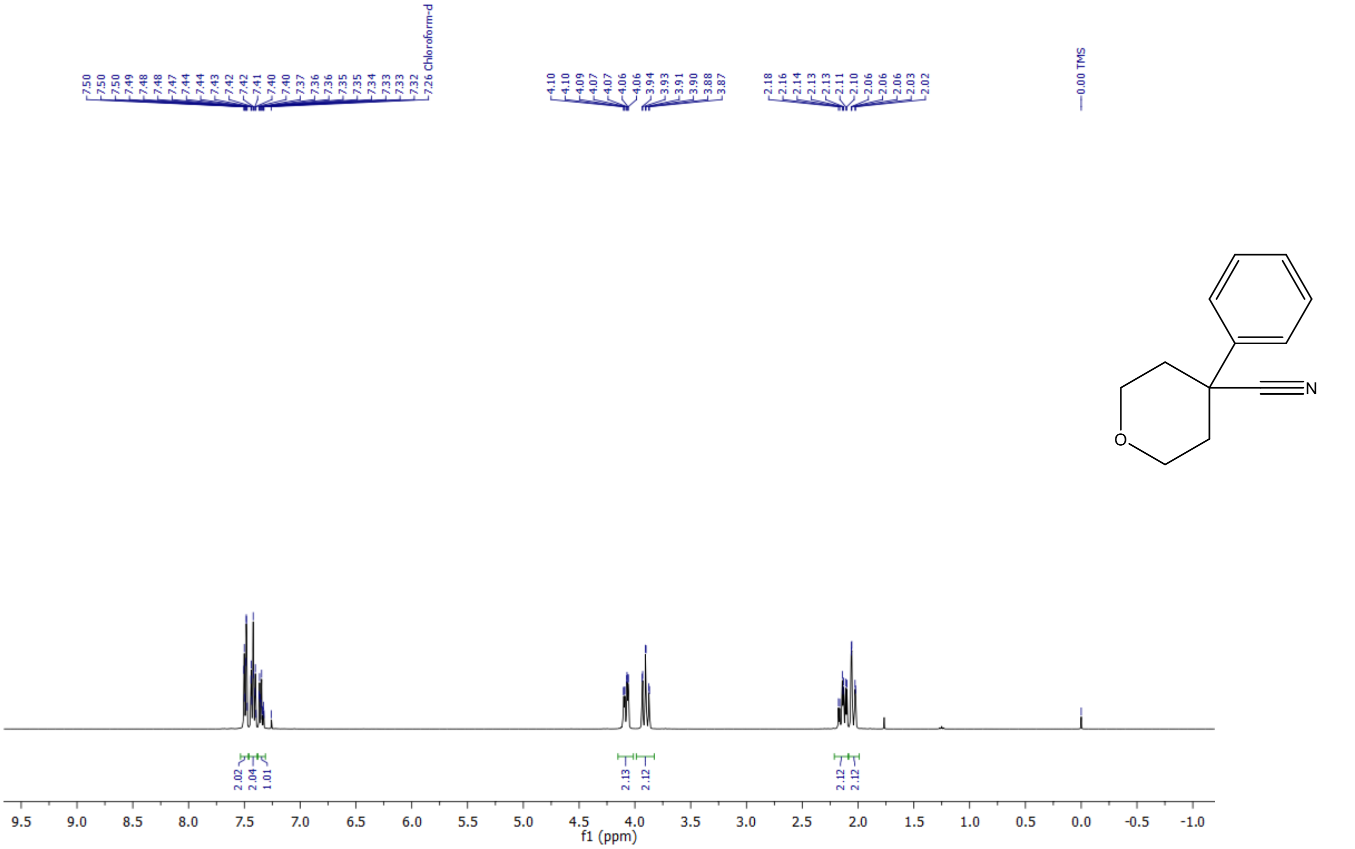

4-(4-(Trifluoromethyl)phenyl)tetrahydro-2*H*-pyran-4-carbonitrile (87)

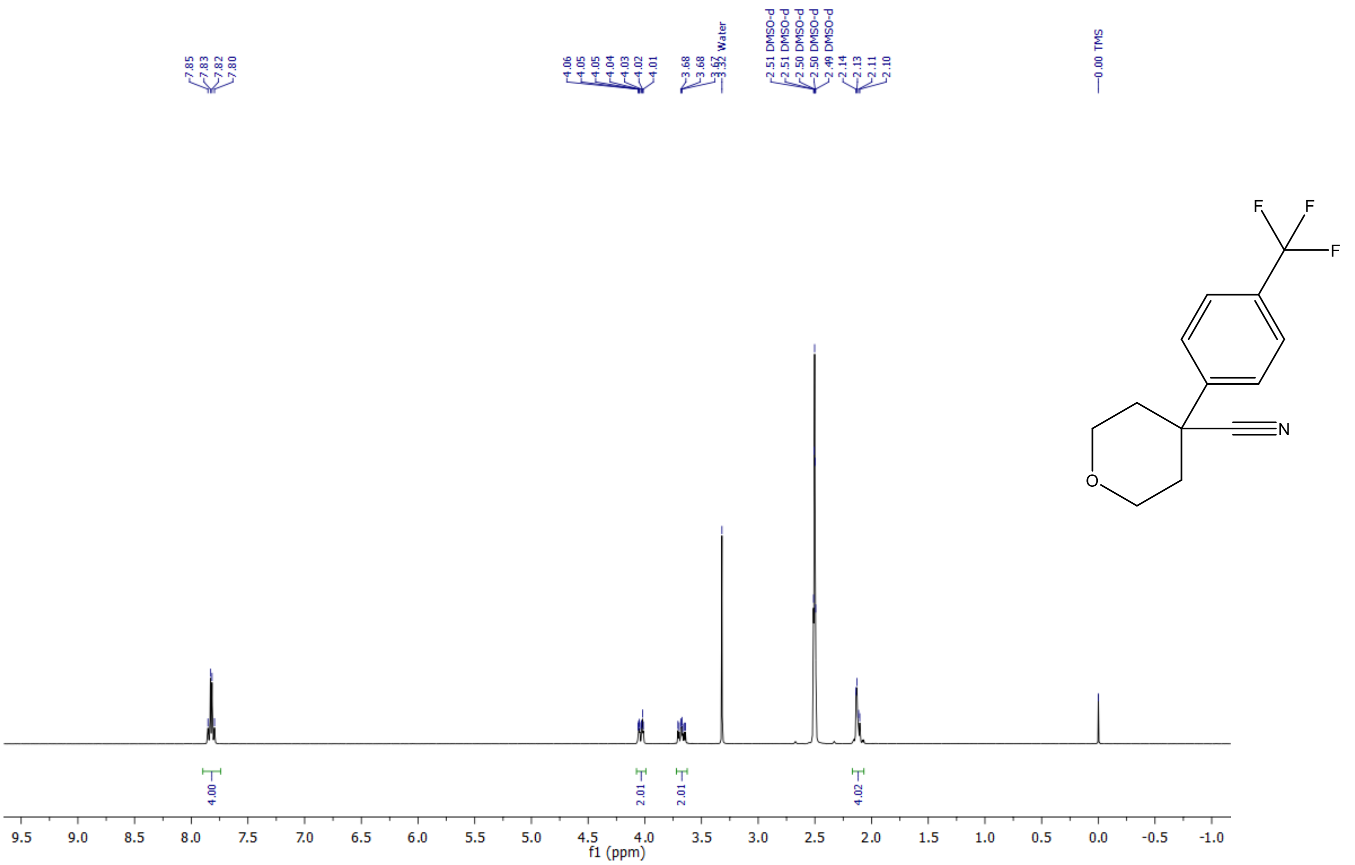

4-(3,4-Dimethoxyphenyl)tetrahydro-2*H*-pyran-4-carbonitrile (88)

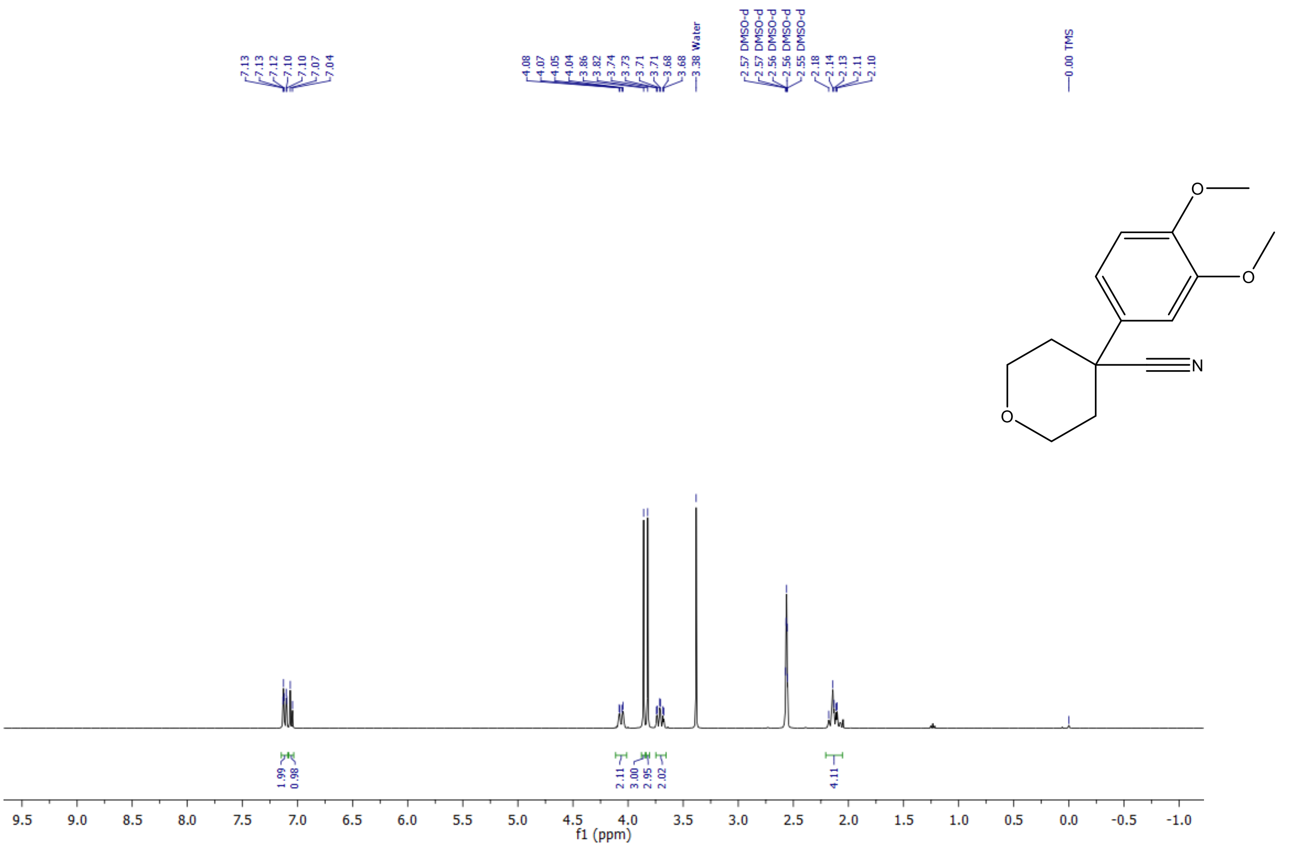

4-(4-Methoxyphenyl)tetrahydro-2H-pyran-4-carbonitrile (89)

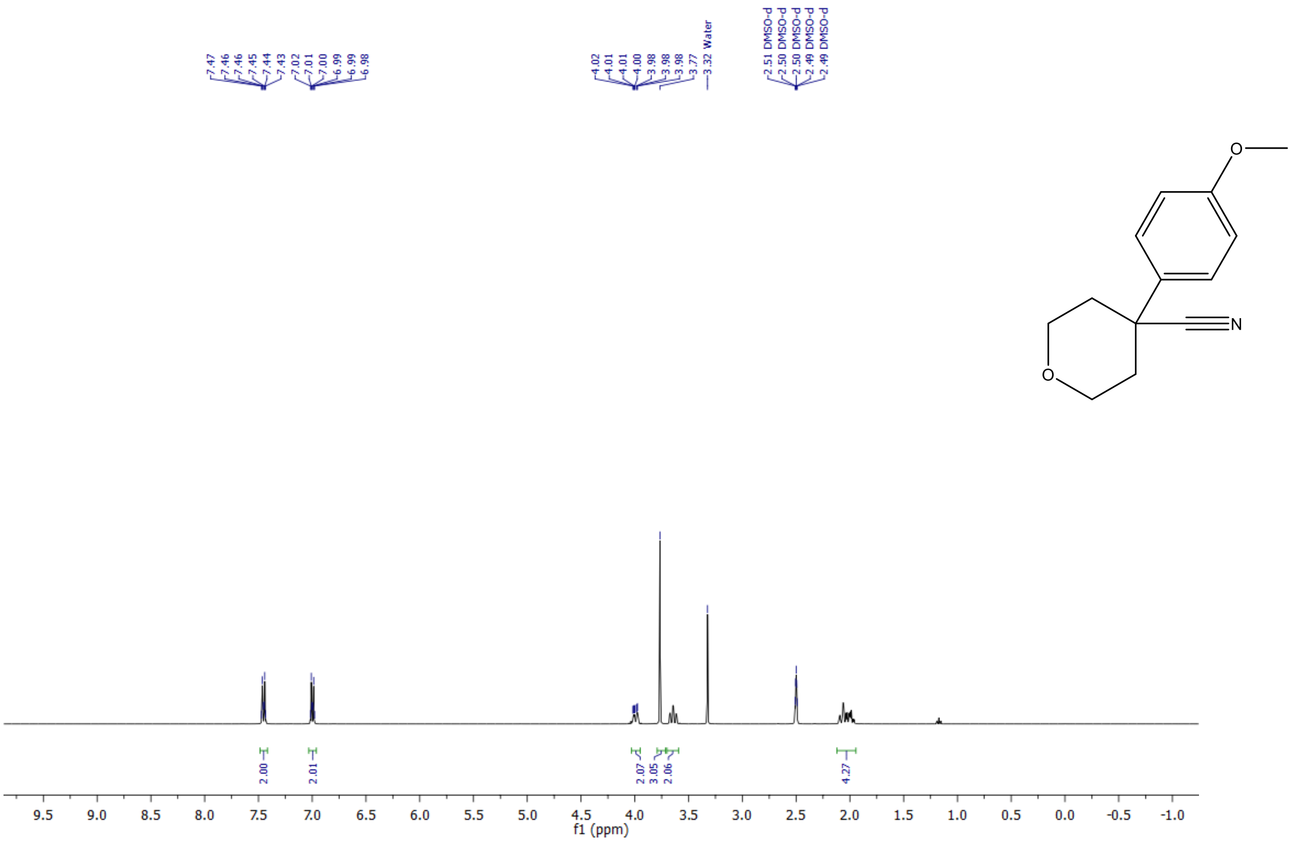

4-(*p*-Tolyl)tetrahydro-2H-pyran-4-carbonitrile (90)

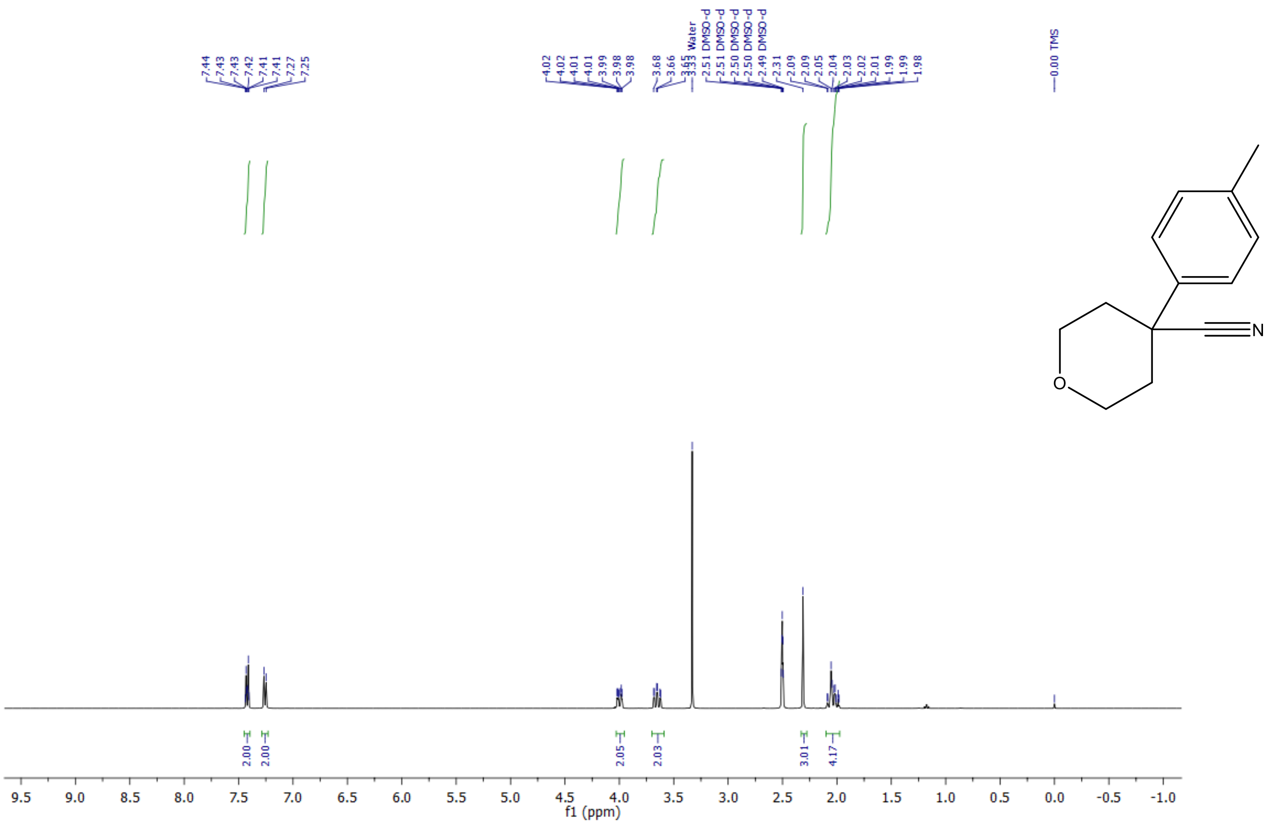

4-(Benzo[*d*][1,3]dioxol-5-yl)tetrahydro-2*H*-pyran-4-carbonitrile (91)

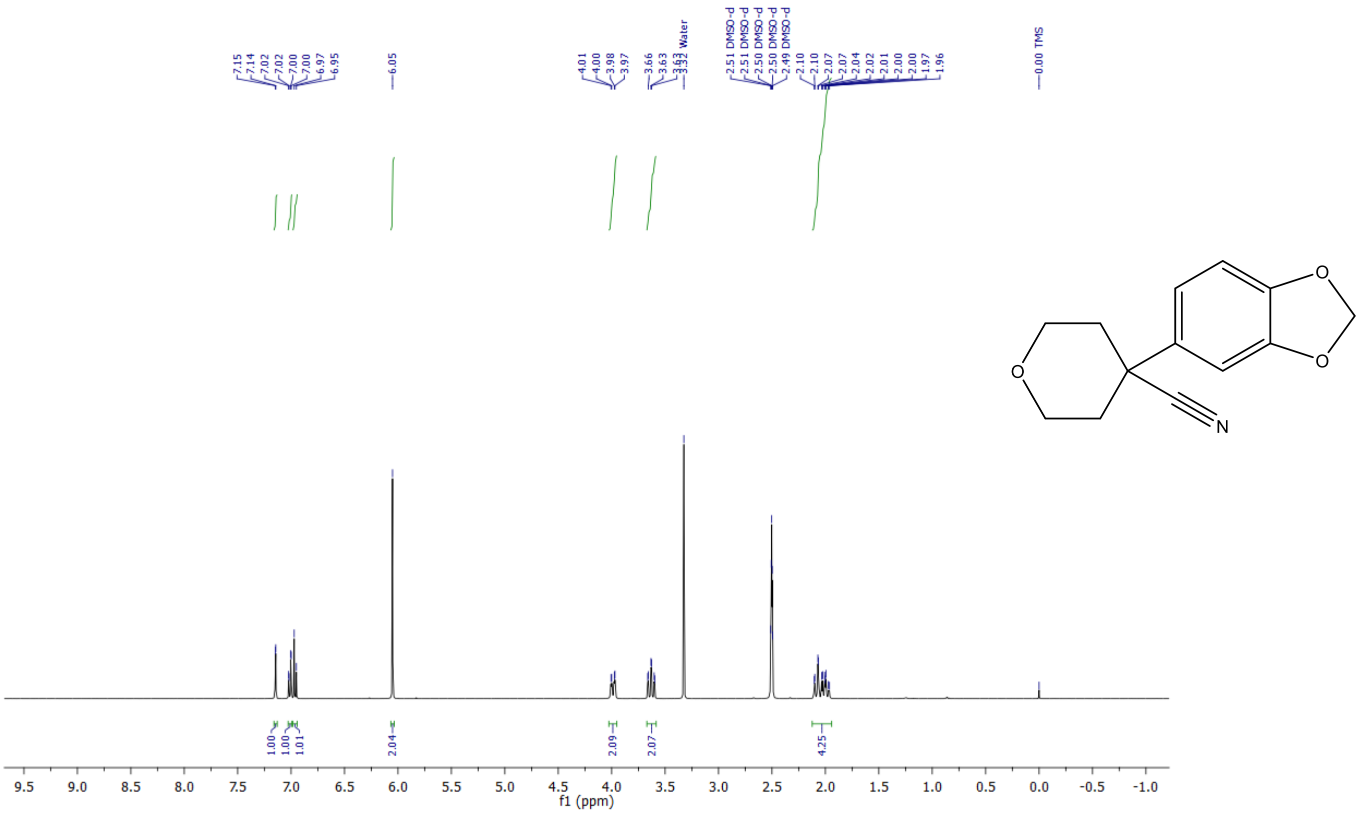

4-(2-Methoxyphenyl)tetrahydro-2*H*-pyran-4-carbonitrile (92)

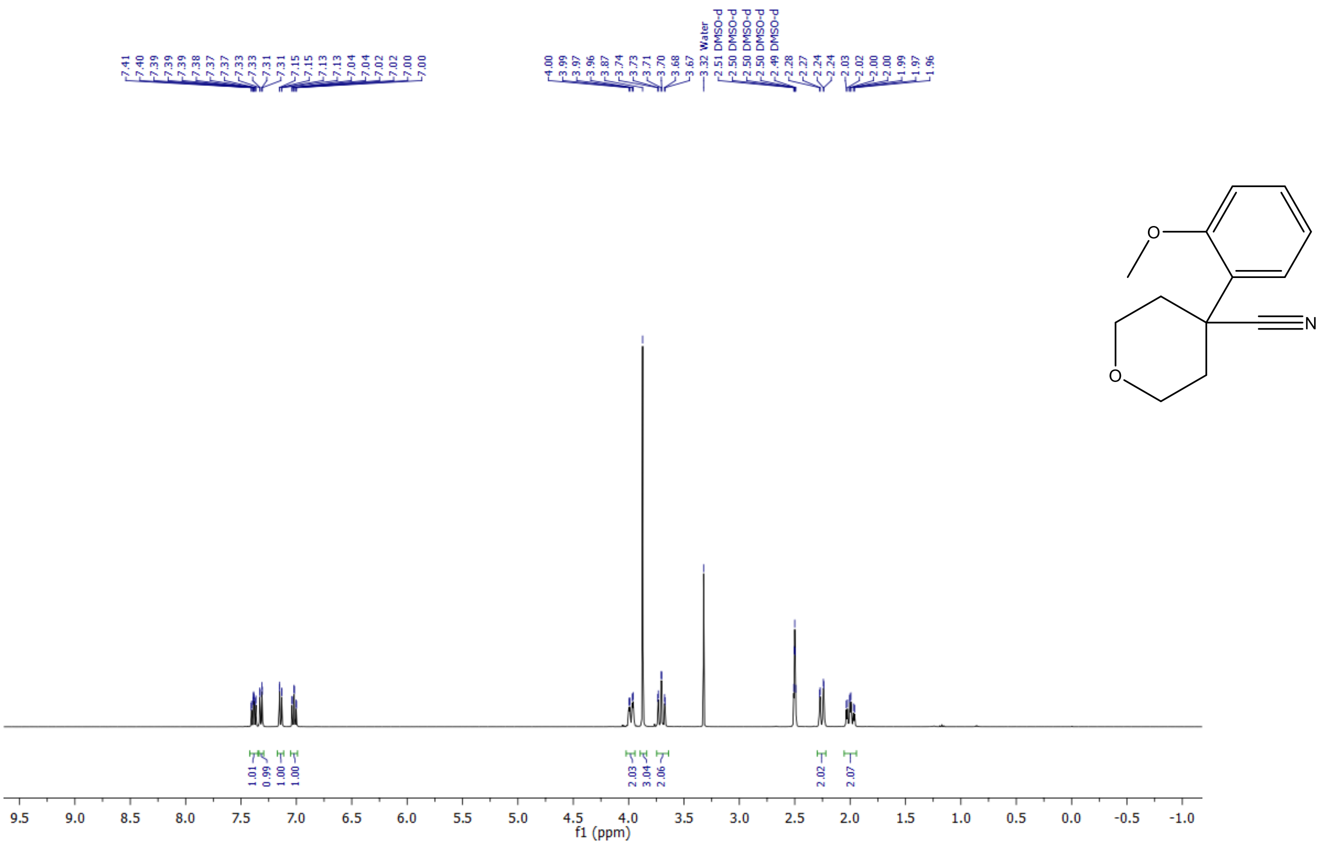

4-(3-Methoxyphenyl)tetrahydro-2*H*-pyran-4-carbonitrile (93)

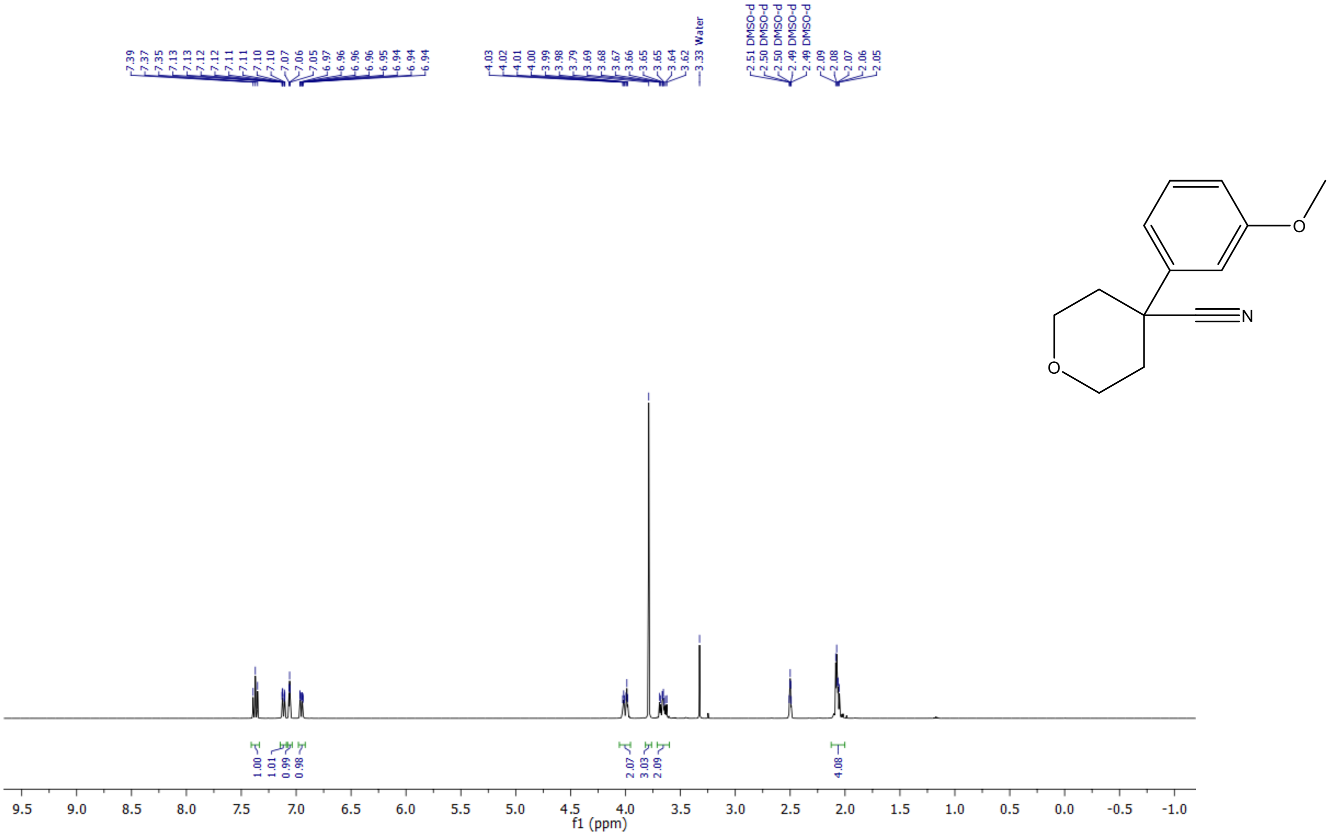

4-(*m*-tolyl)Tetrahydro-2*H*-pyran-4-carbonitrile (94)

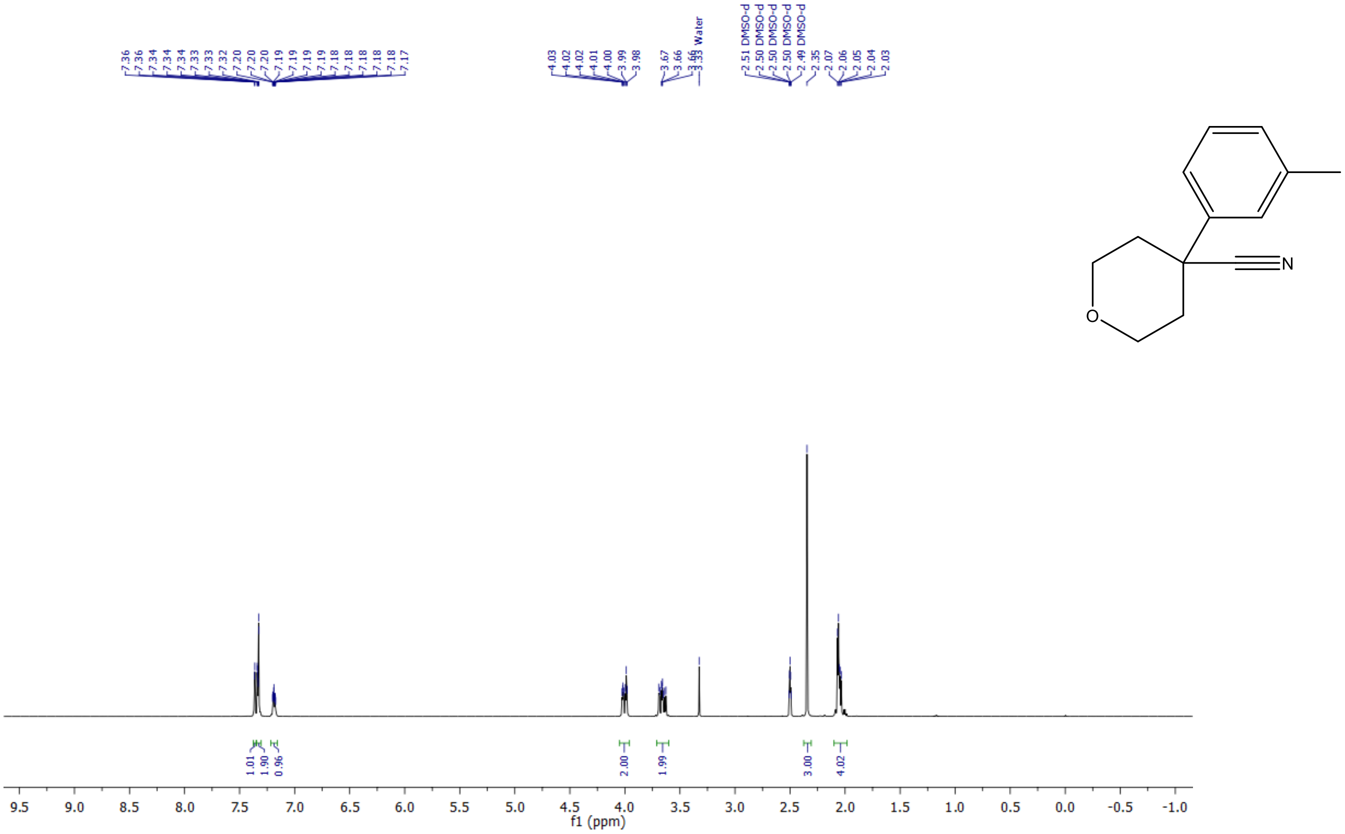

(4-Phenyltetrahydro-2H-pyran-4-yl)methanamine (95)

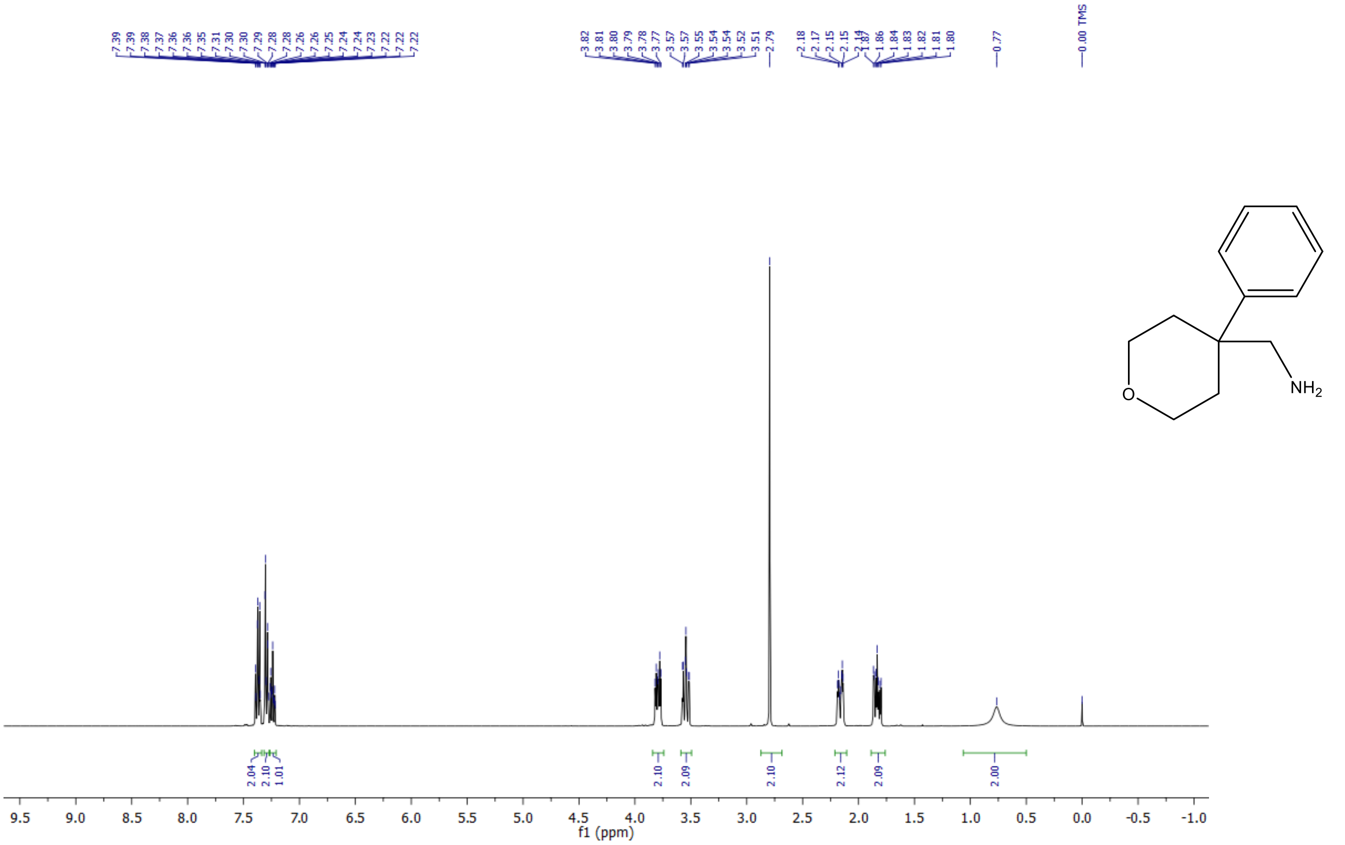

(4-(4-(Trifluoromethyl)phenyl)tetrahydro-2*H*-pyran-4-yl)methanamine (96)

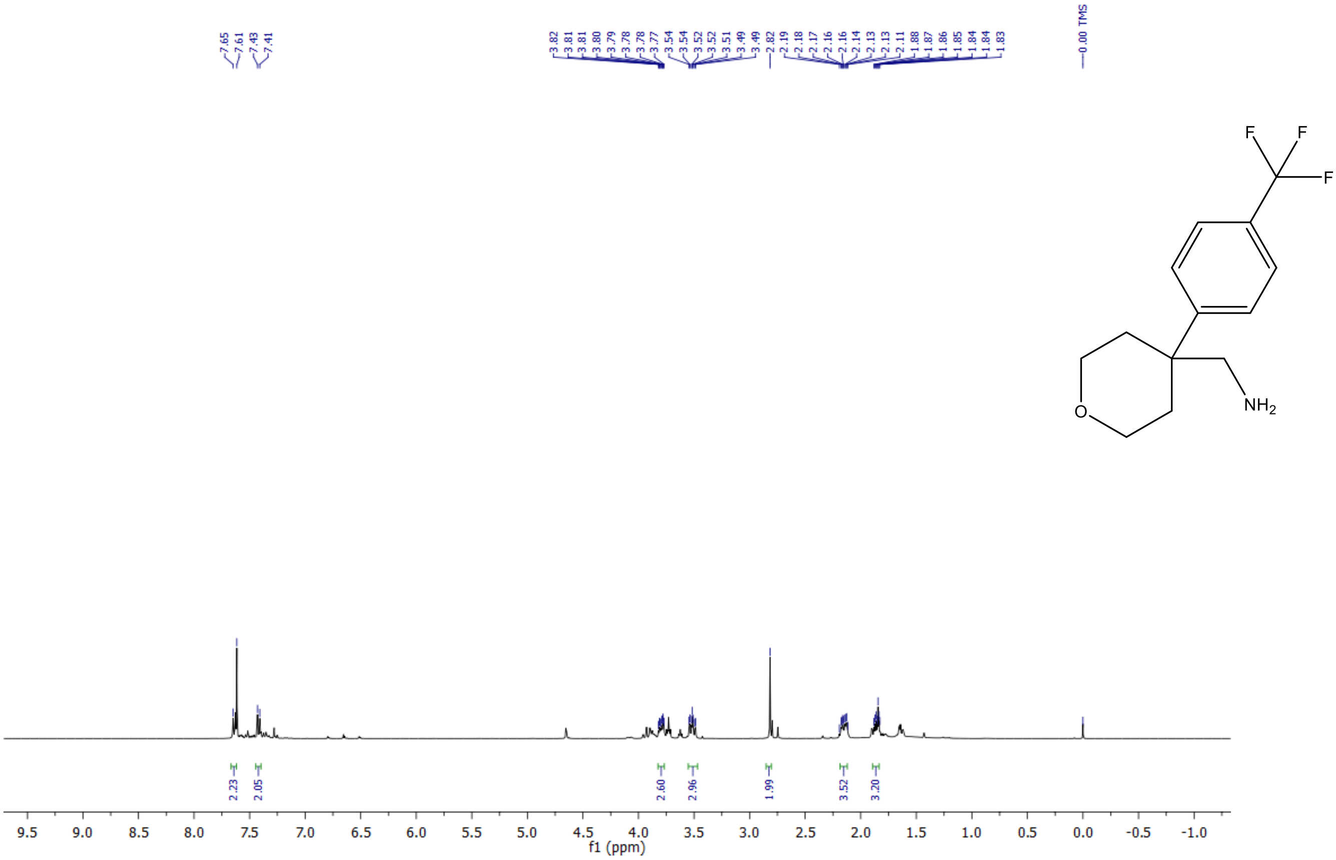

(4-(3,4-Dimethoxyphenyl)tetrahydro-2*H*-pyran-4-yl)methanamine (97)

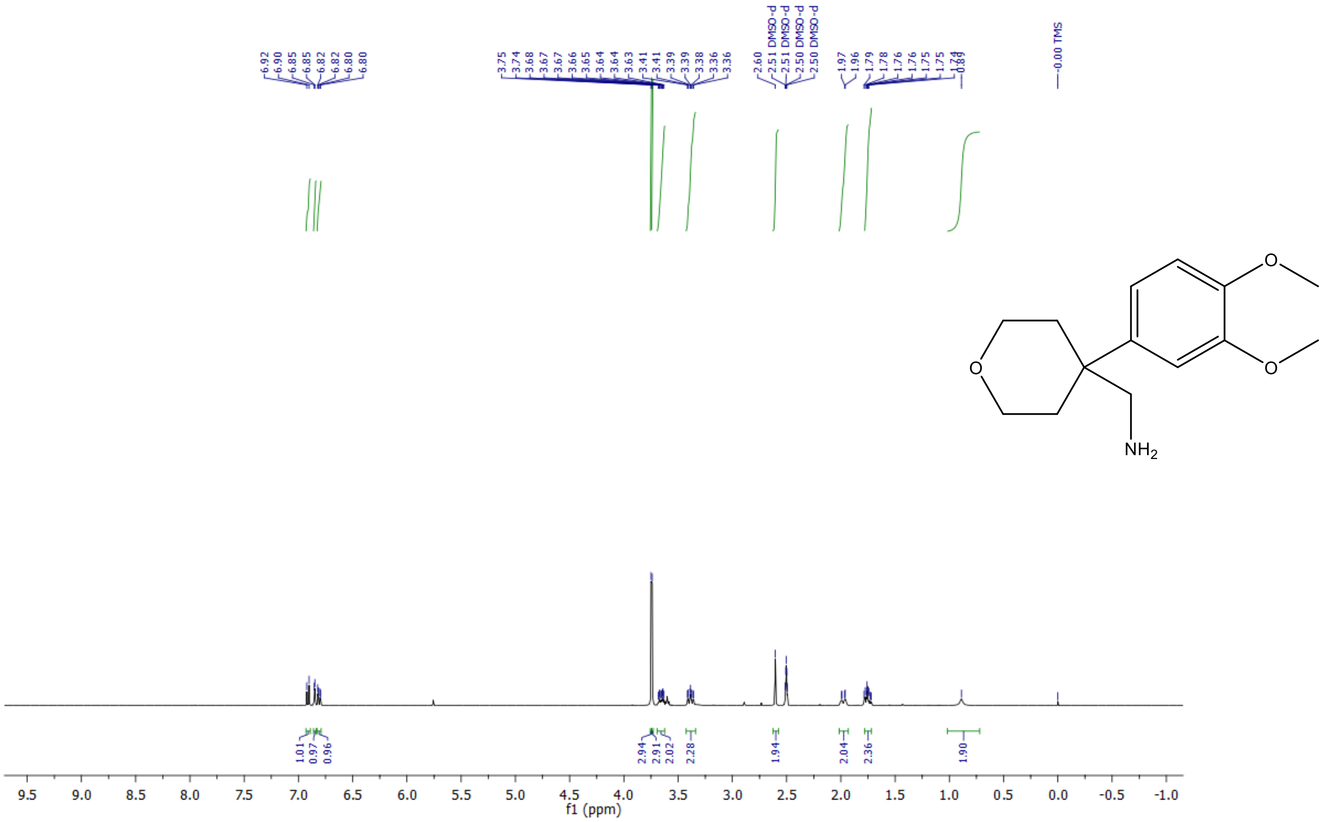

(4-(4-Methoxyphenyl)tetrahydro-2H-pyran-4-yl)methanamine (98)

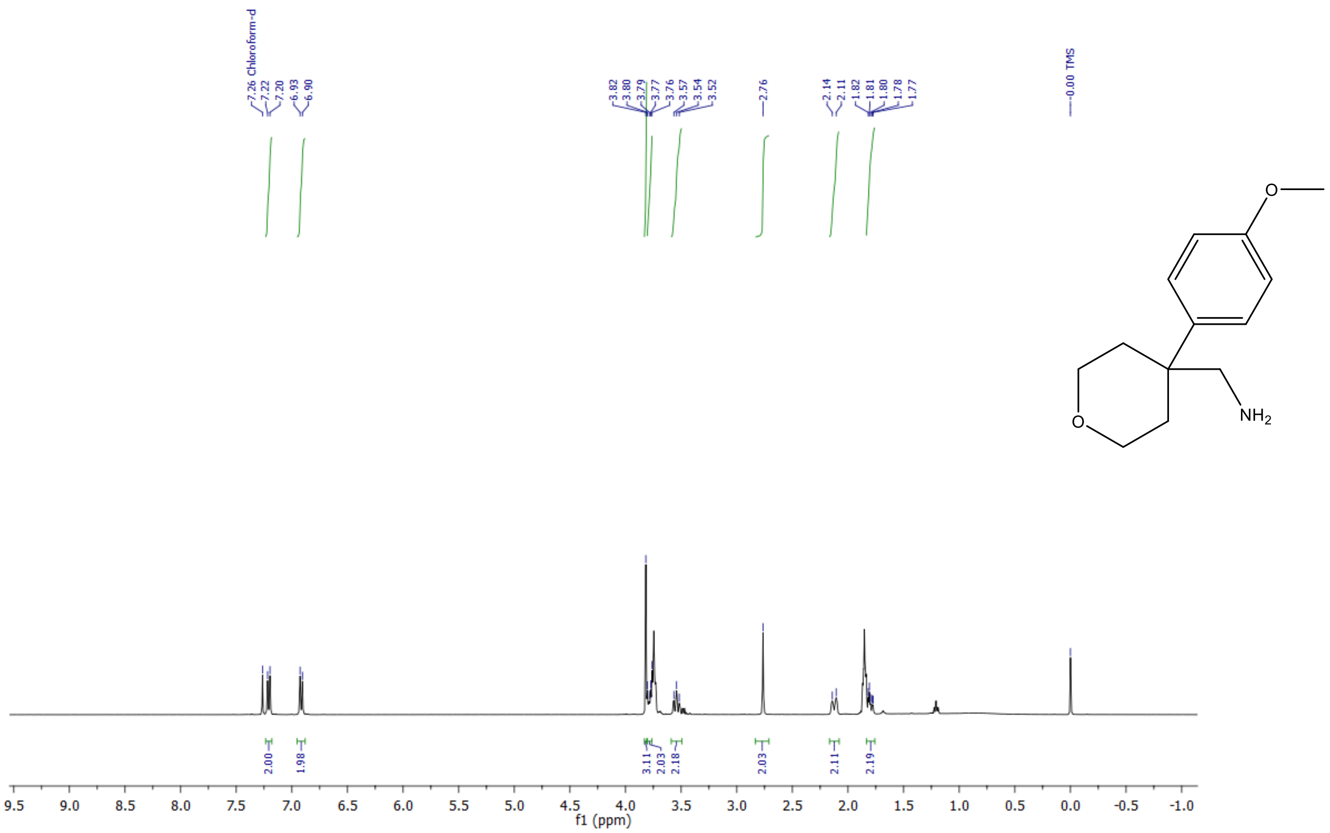

(4-(*p*-Tolyl)tetrahydro-2H-pyran-4-yl)methanamine (99)

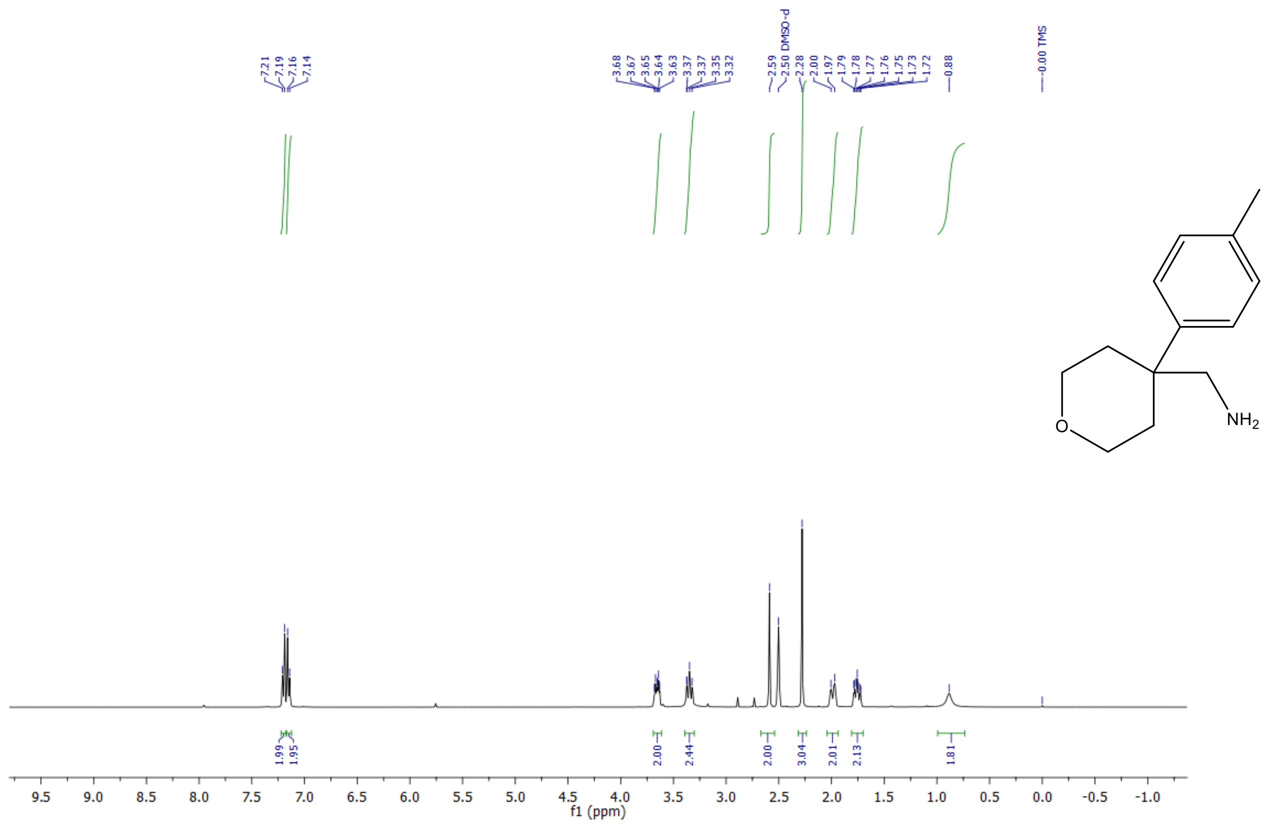

(4-(Benzo[*d*][1,3]dioxol-5-yl)tetrahydro-2*H*-pyran-4-yl)methanamine (100)

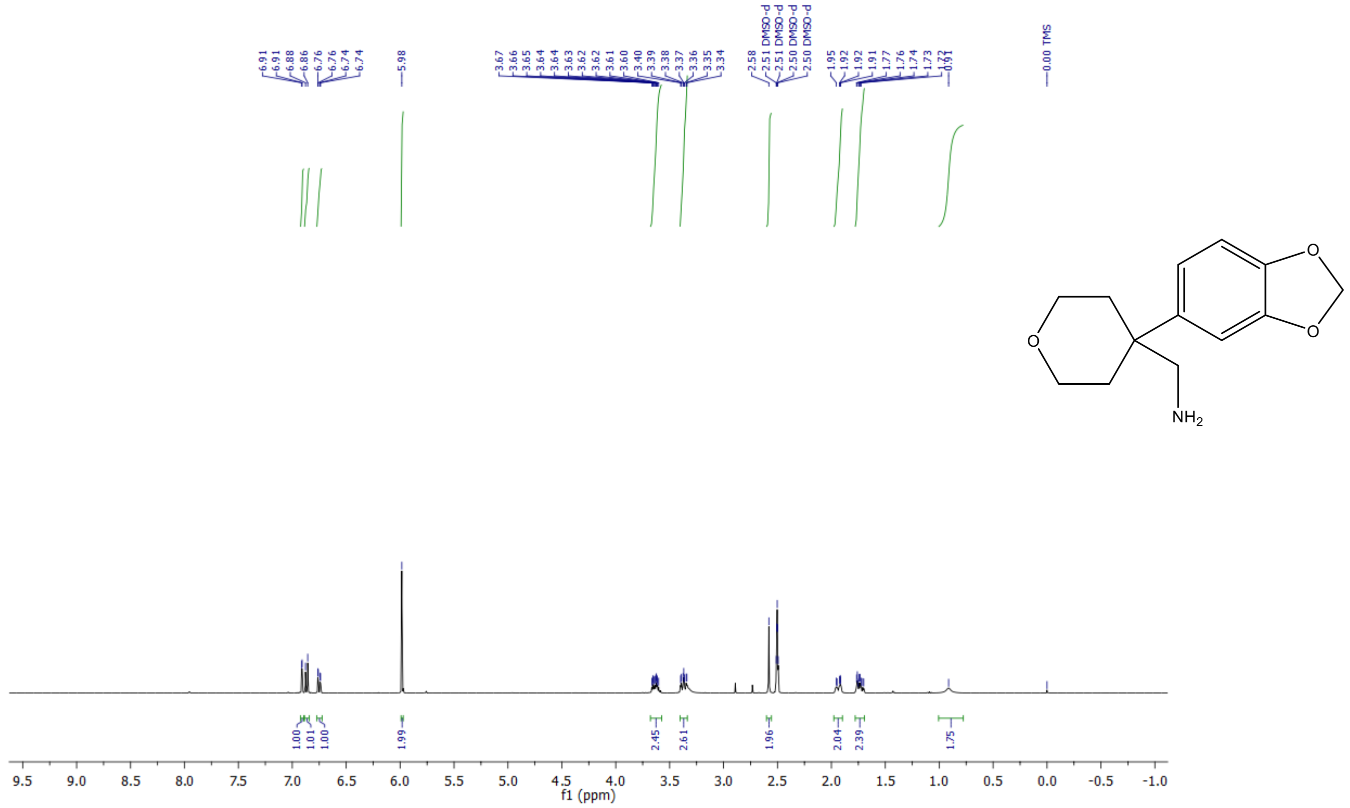

(4-(2-Methoxyphenyl)tetrahydro-2H-pyran-4-yl)methanamine (101

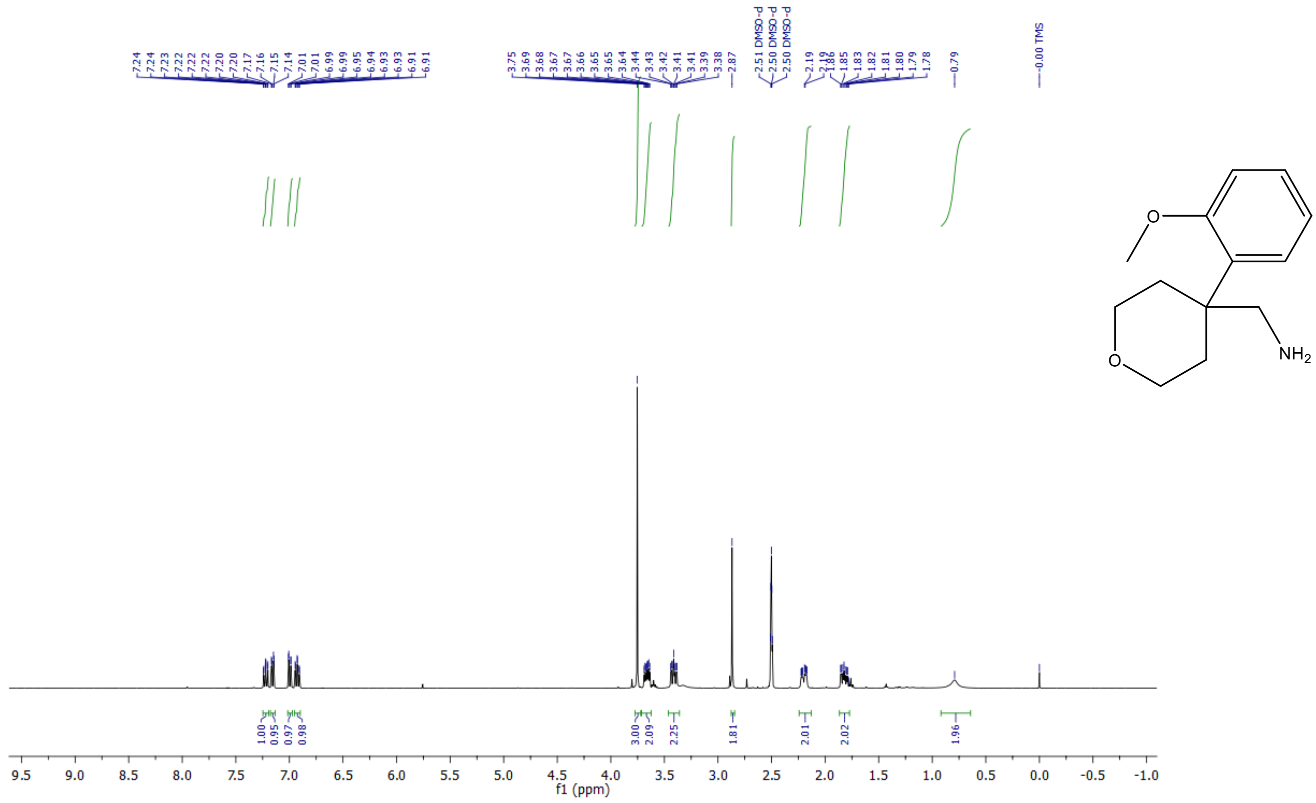

(4-(3-Methoxyphenyl)tetrahydro-2*H*-pyran-4-yl)methanamine (102)

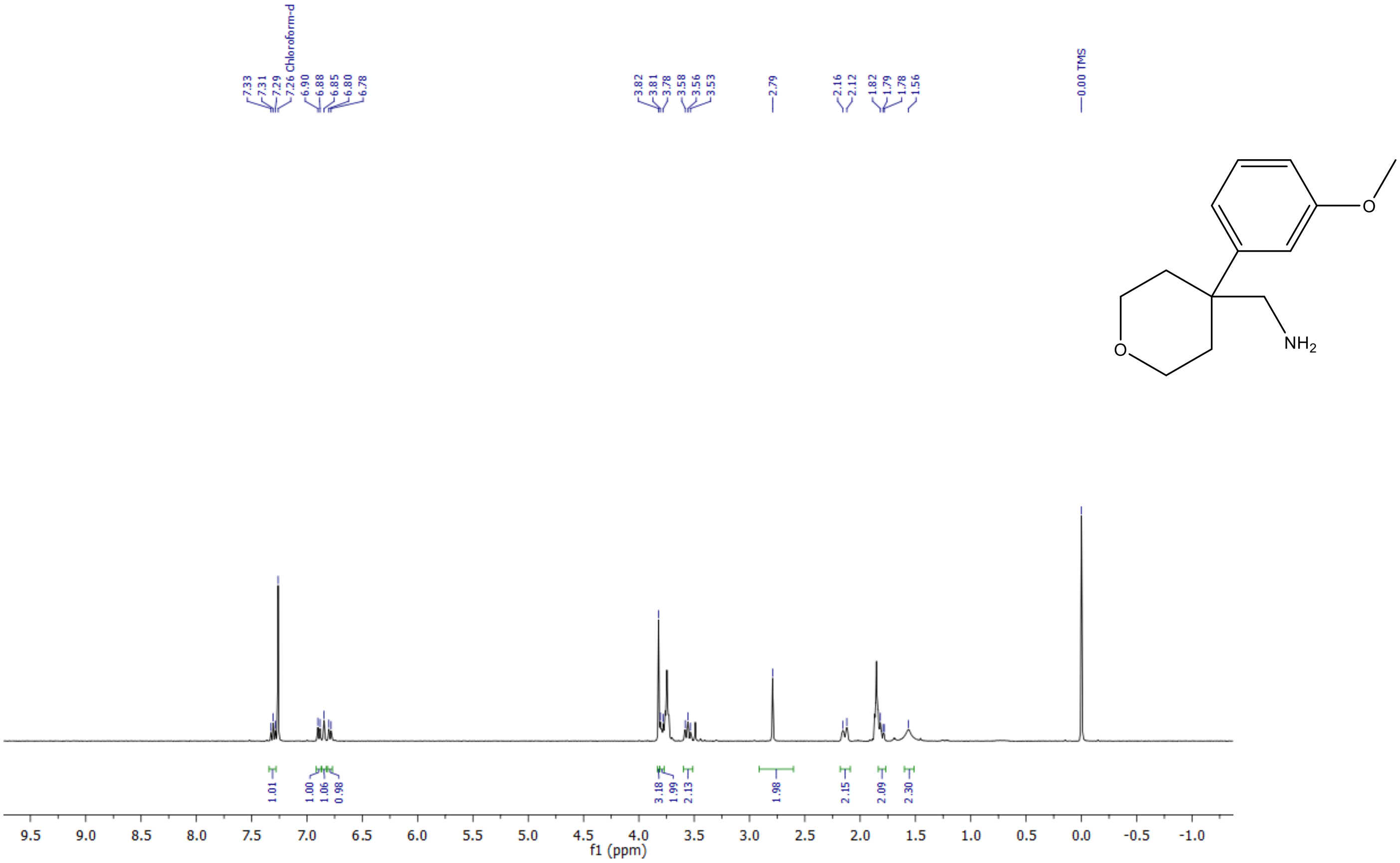

(4-(*m*-Tolyl)tetrahydro-2*H*-pyran-4-yl)methanamine (103)

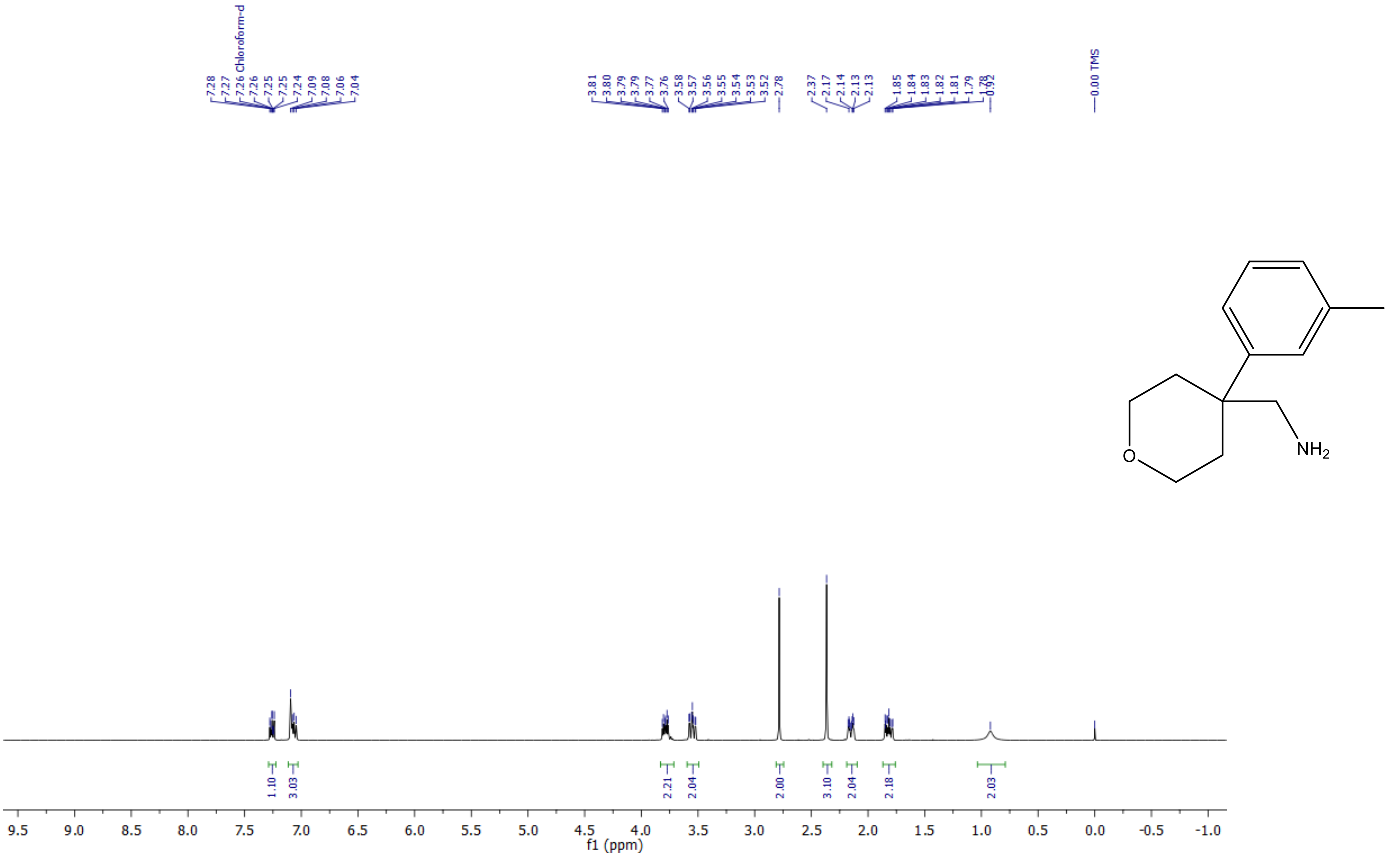

***N*-((4-(Benzo[*b*]thiophen-3-yl)tetrahydro-2*H*-pyran-4-yl)methyl)-2-methoxybenzamide (38)**

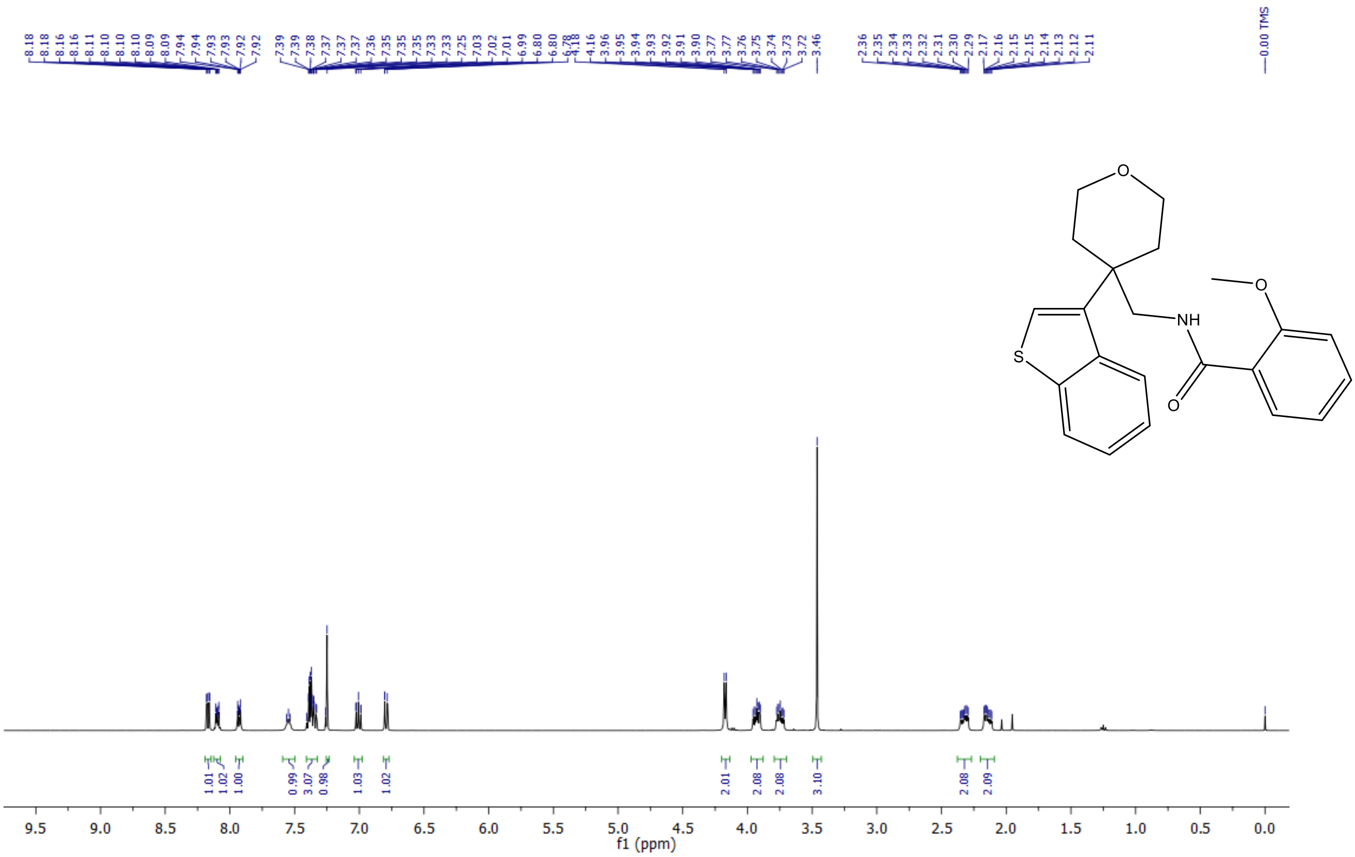

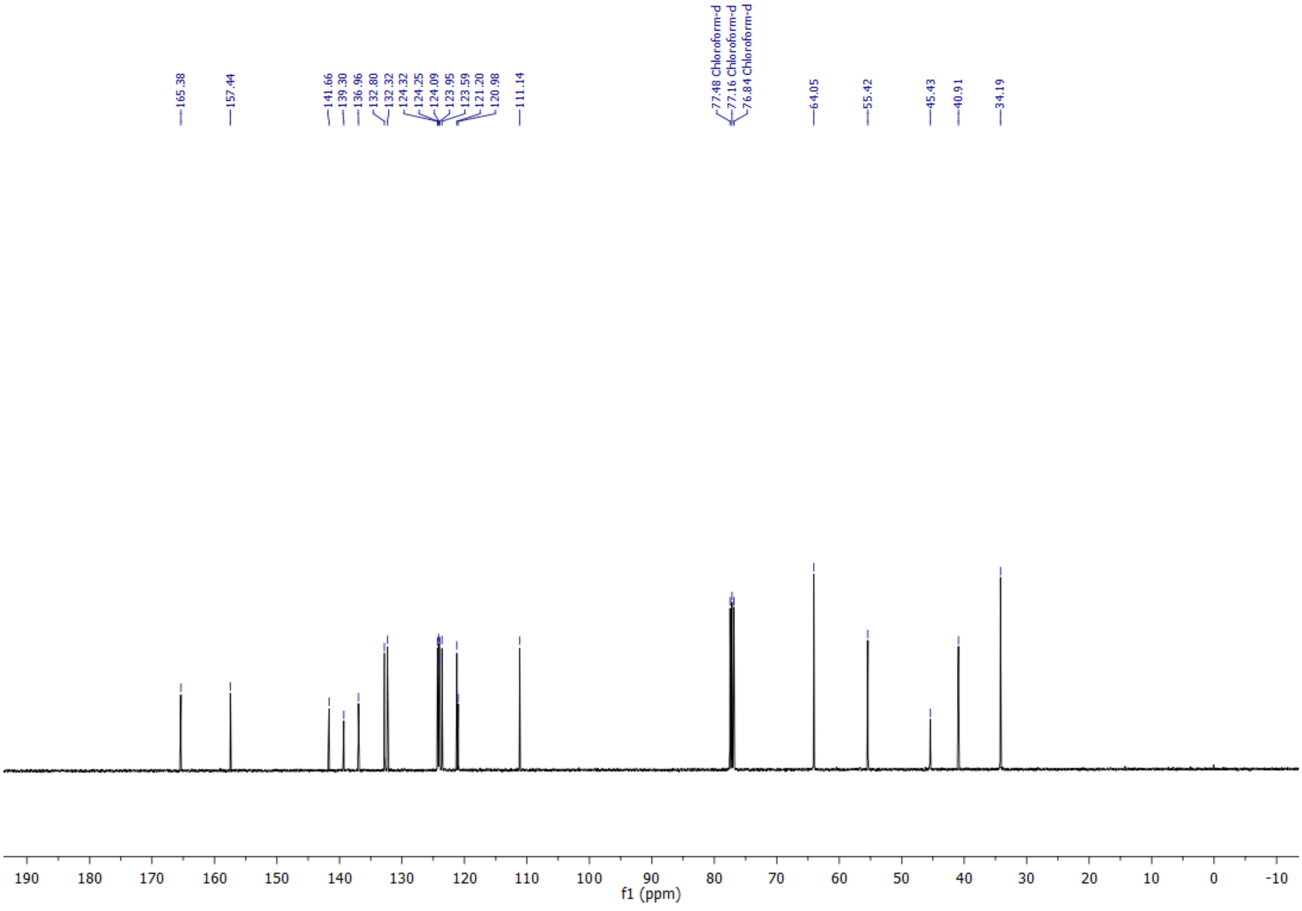

2-Methoxy-*N*-((4-(4-(4-methoxyphenyl)thiazol-2-yl)tetrahydro-2*H*-pyran-4-yl)methyl)benzamide (39)

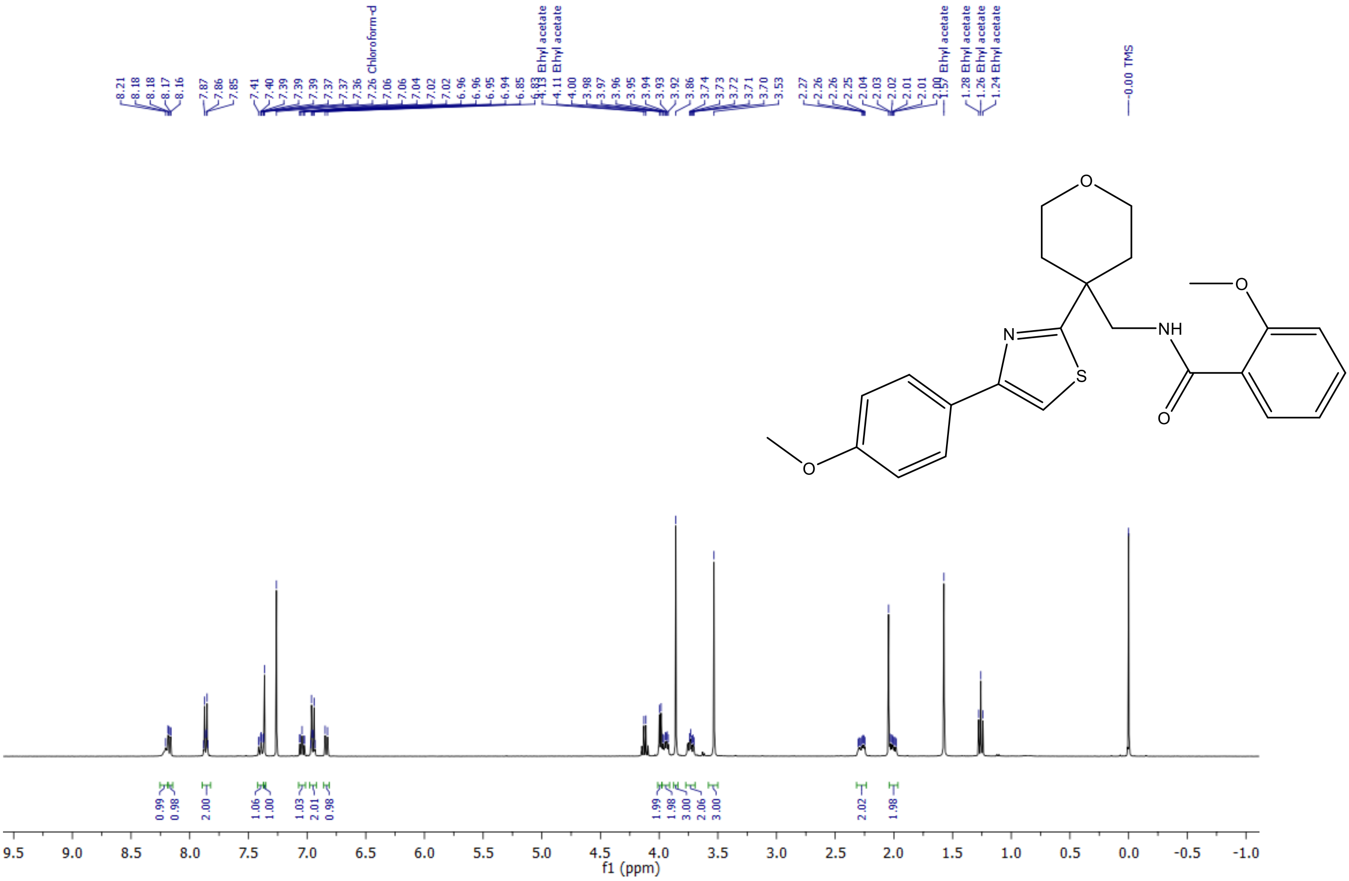

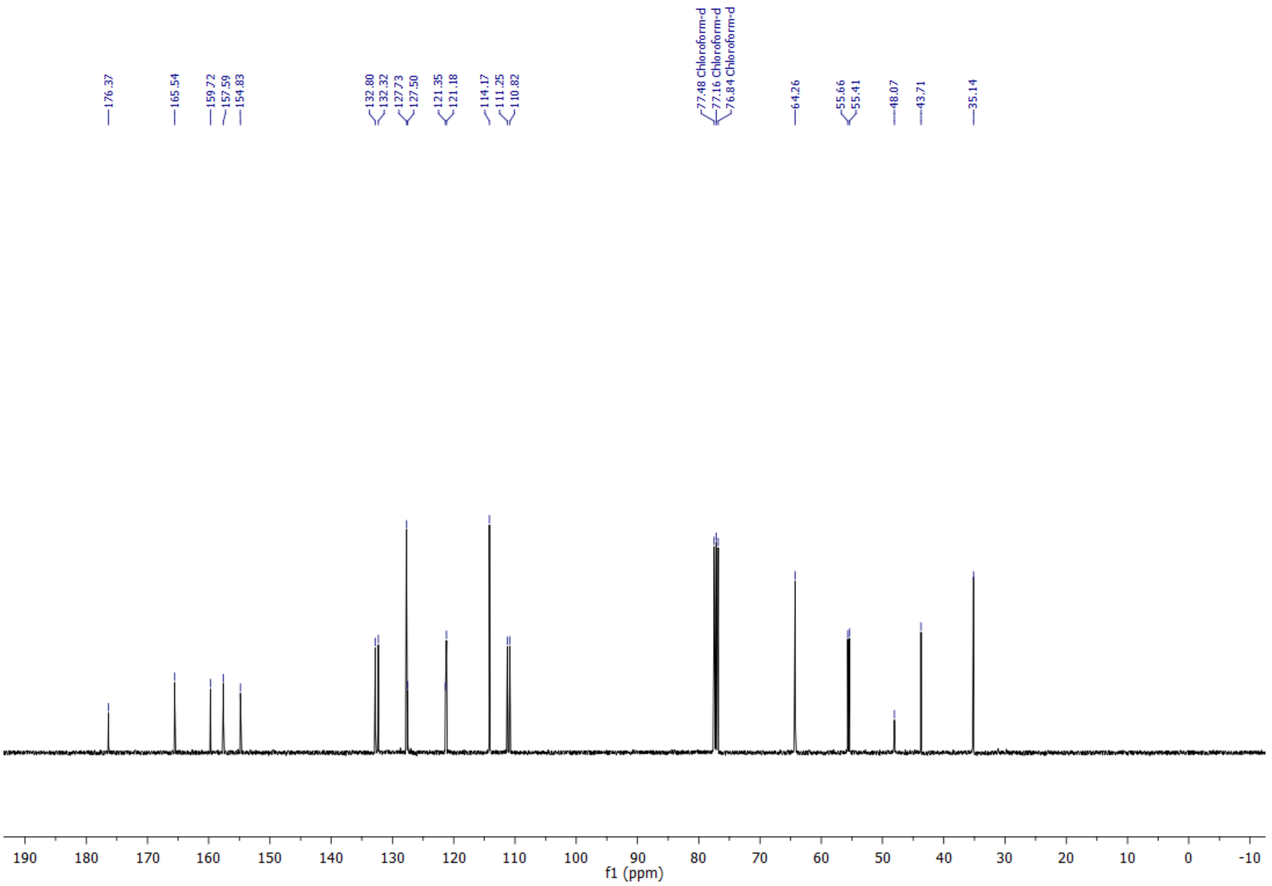

2-Methoxy-*N*-((4-(4-phenylthiazol-2-yl)tetrahydro-2*H*-pyran-4-yl)methyl)benzamide (40)

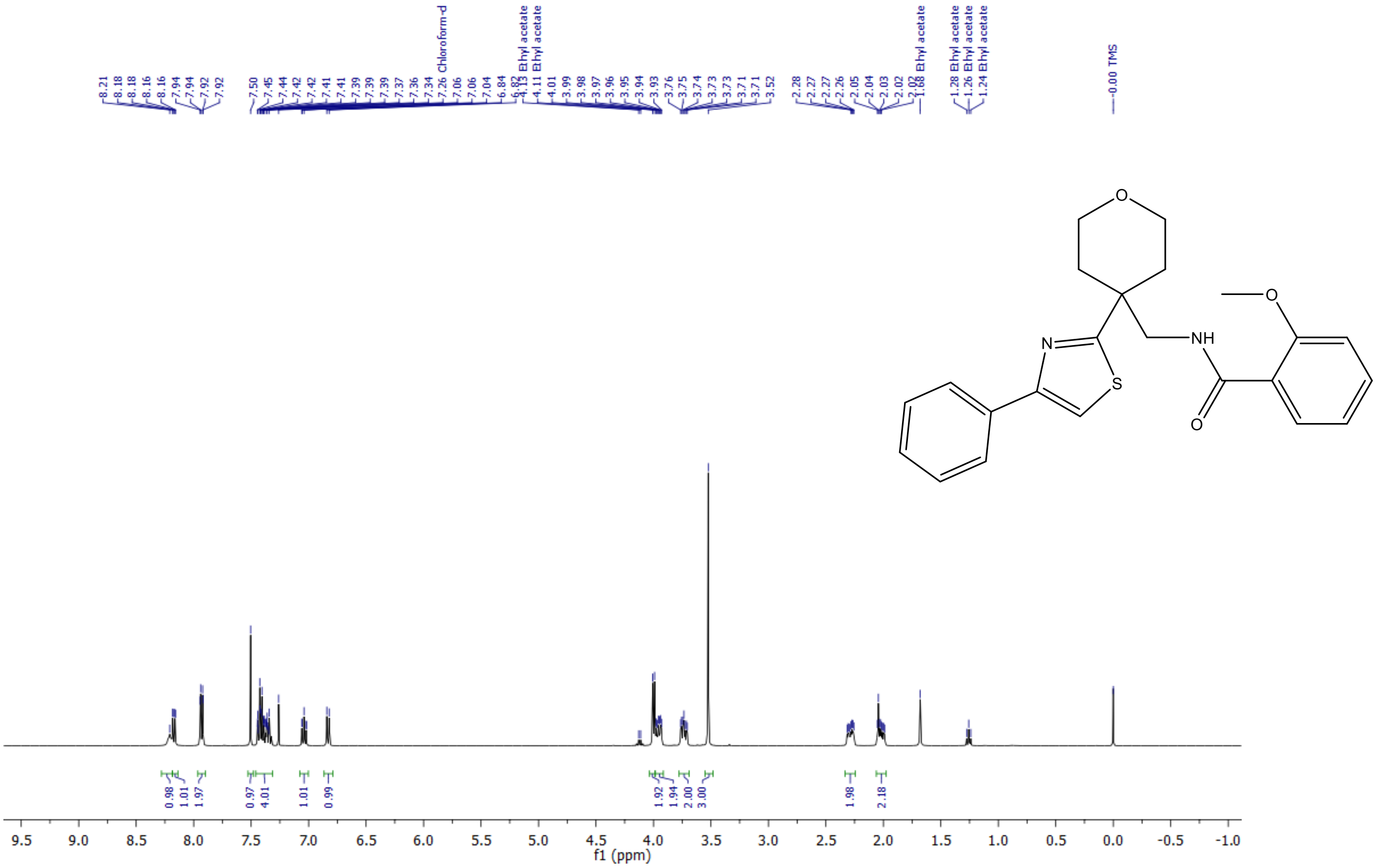

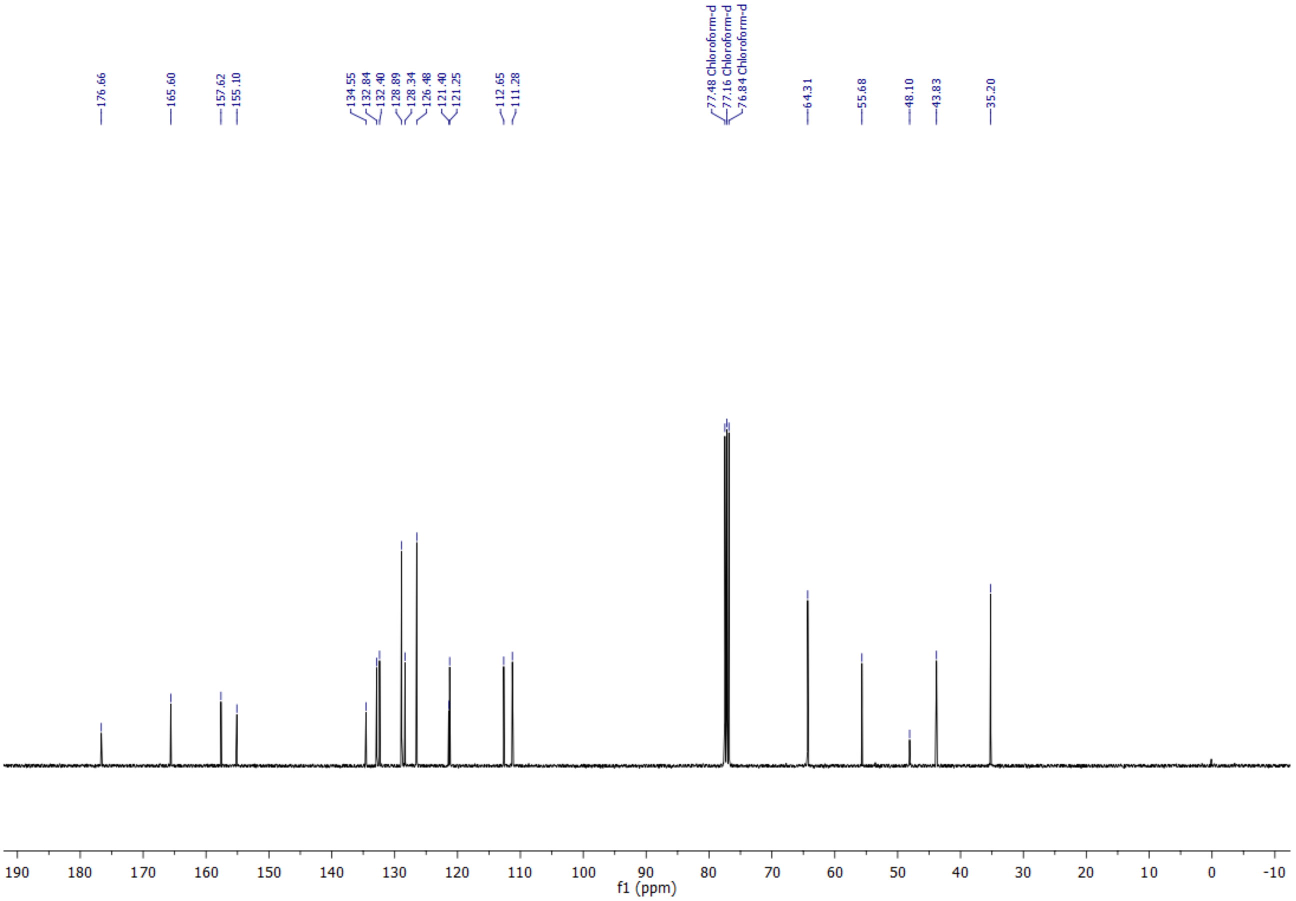

2-Methoxy-*N*-((4-(2-methylthiazol-4-yl)tetrahydro-2*H*-pyran-4-yl)methyl)benzamide (41)

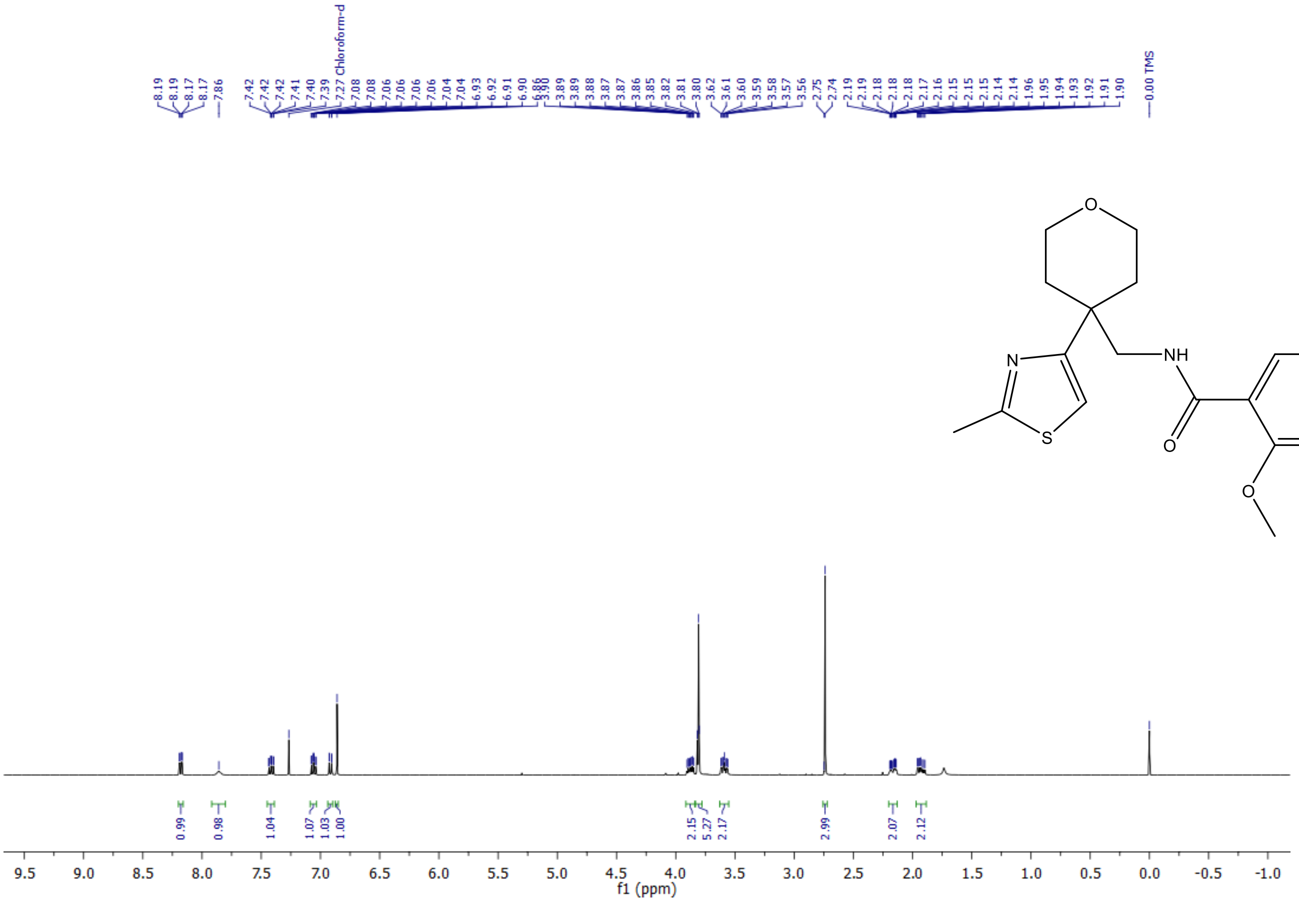

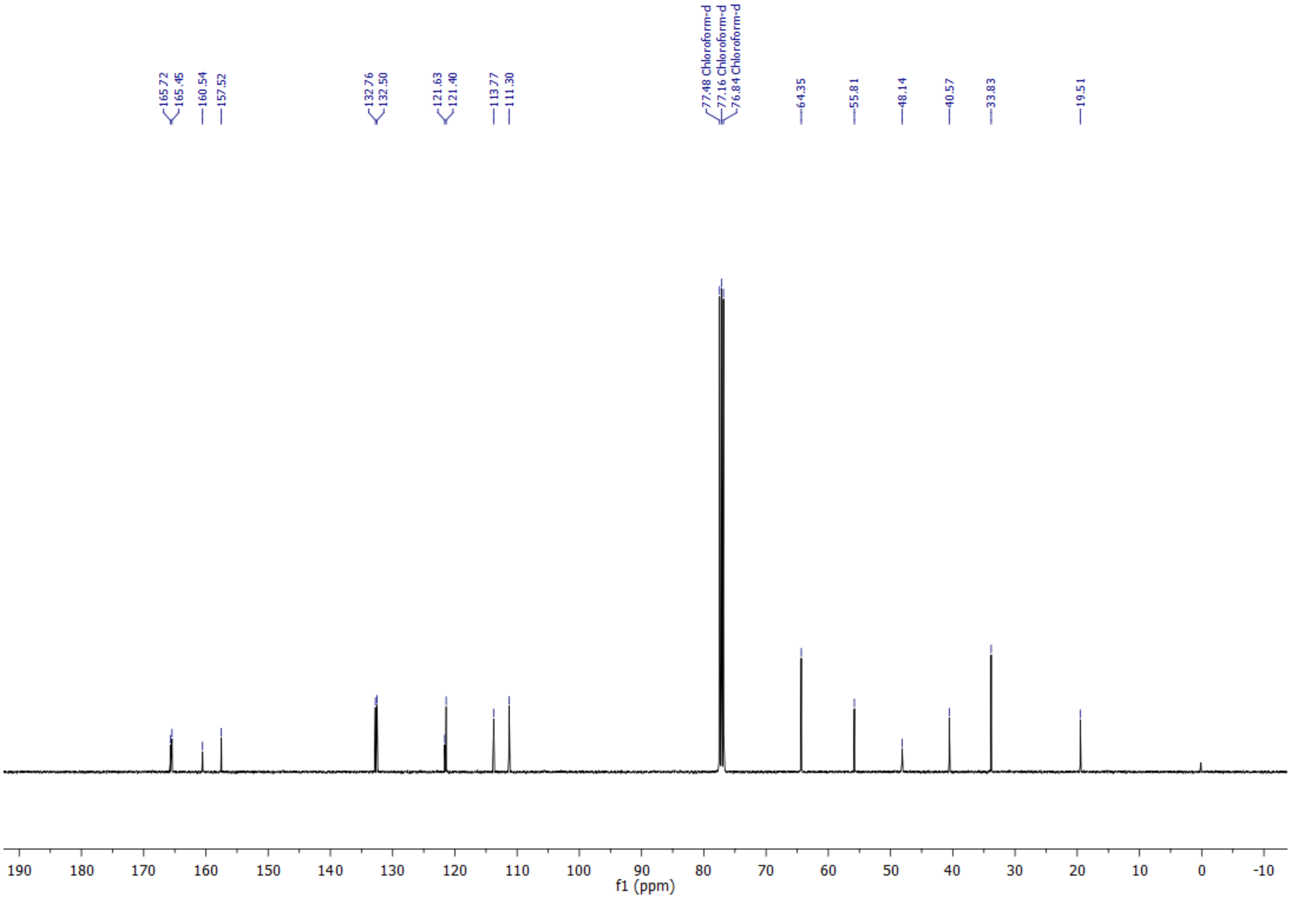

2-Methoxy-*N*-((4-(thiazol-4-yl)tetrahydro-2*H*-pyran-4-yl)methyl)benzamide (42)

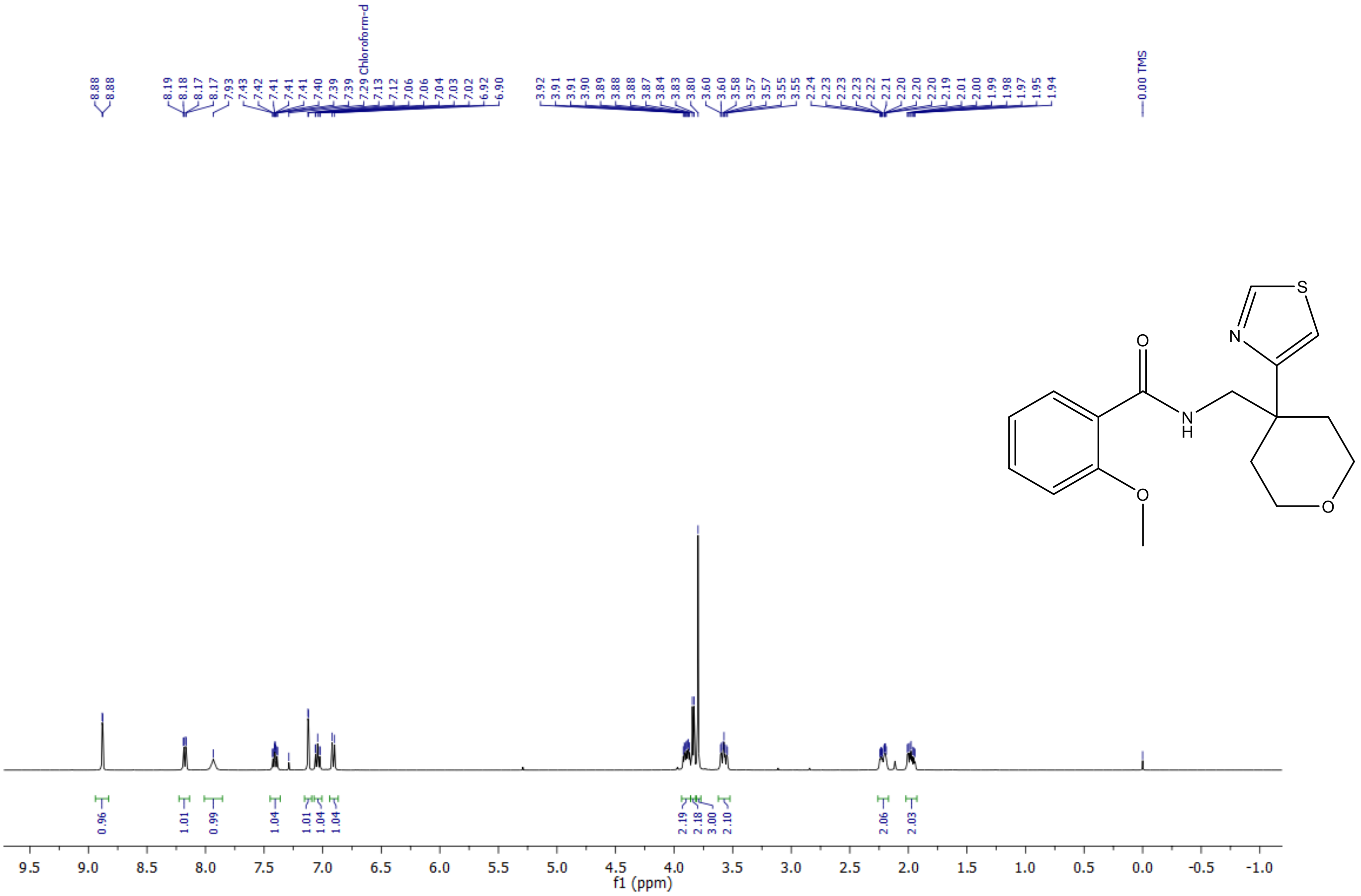

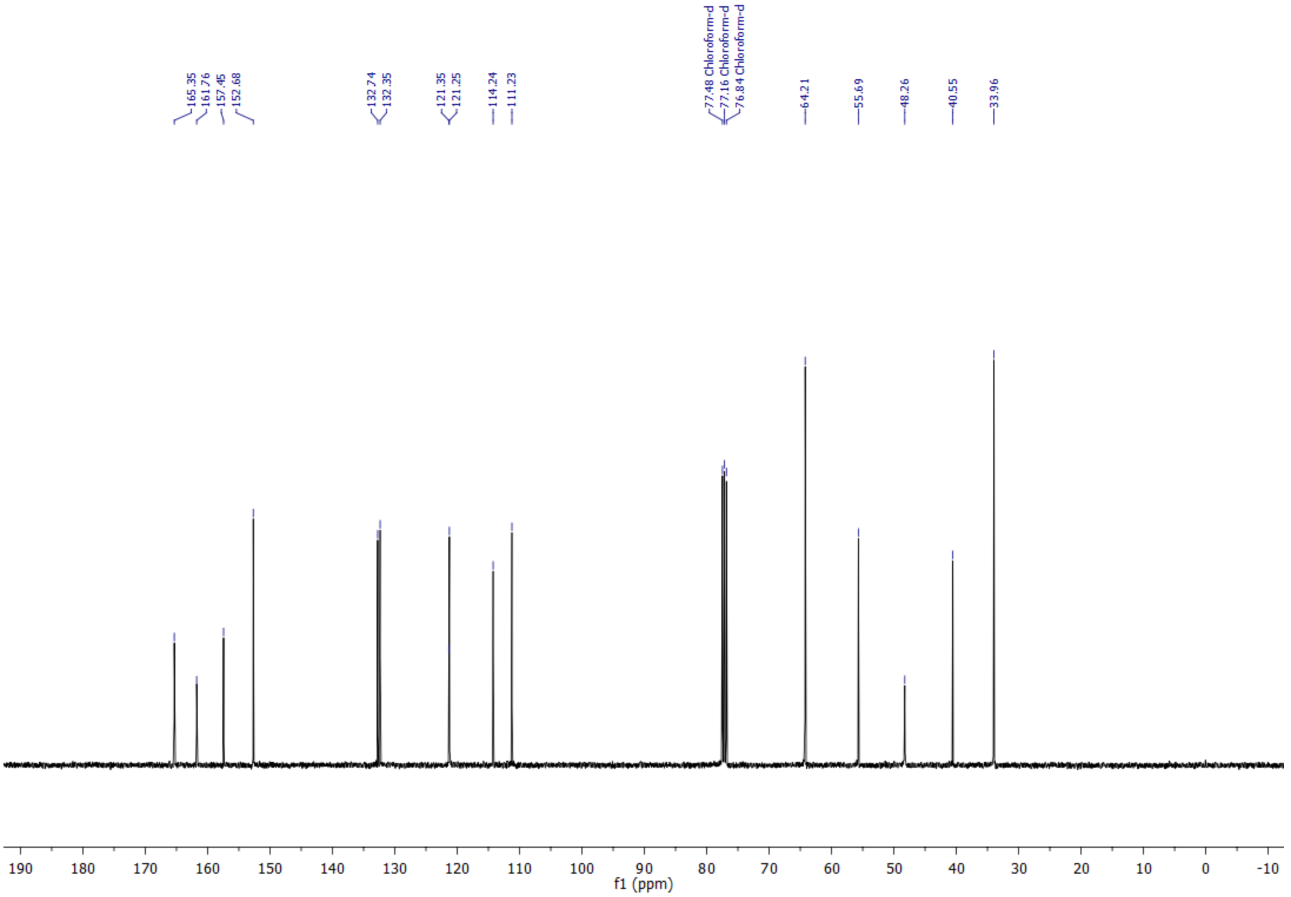

2-Methoxy-*N*-((4-(thiophen-2-yl)tetrahydro-2*H*-pyran-4-yl)methyl)benzamide (43)

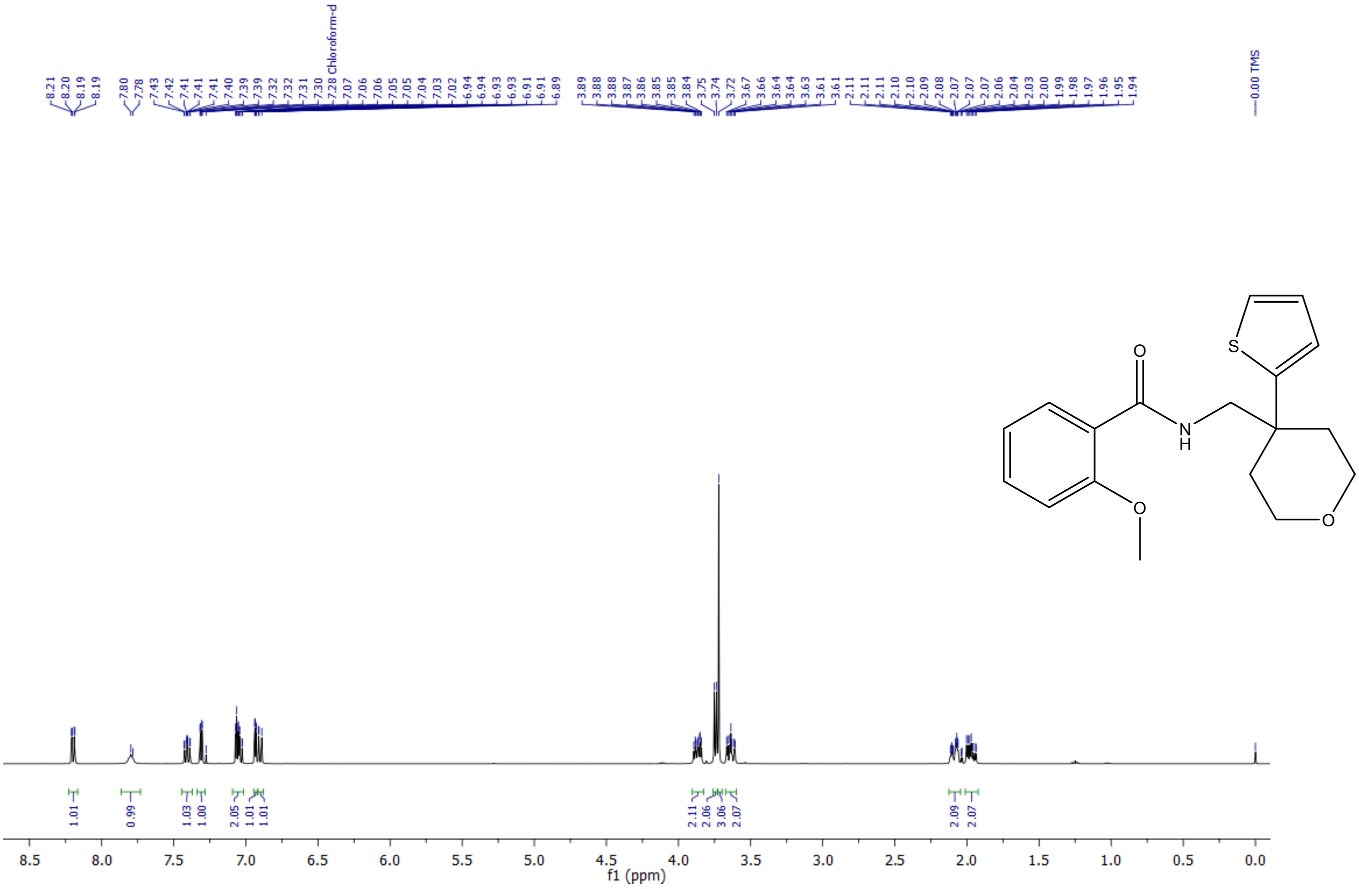

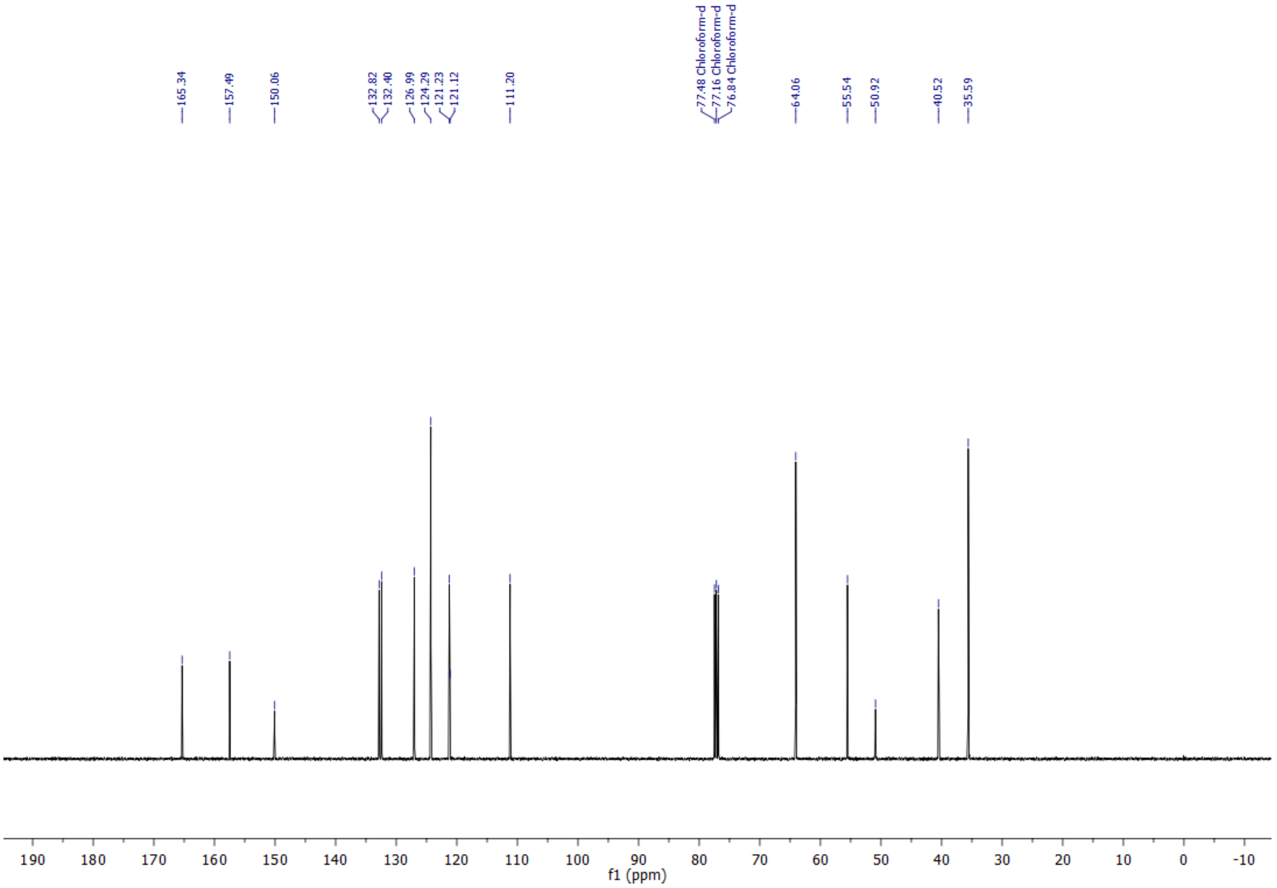

2-Methoxy-*N*-((4-(thiophen-3-yl)tetrahydro-2*H*-pyran-4-yl)methyl)benzamide (44)

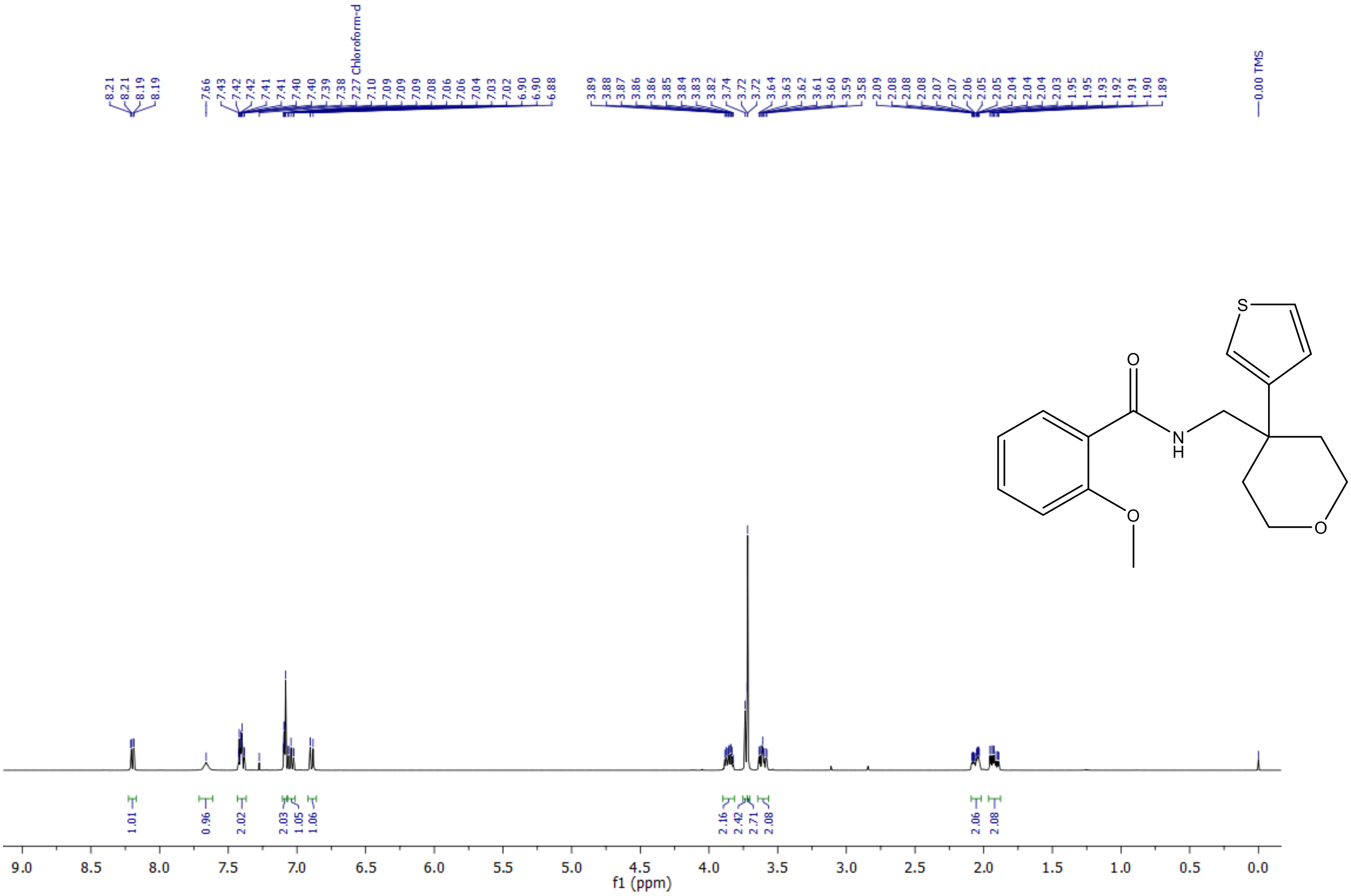

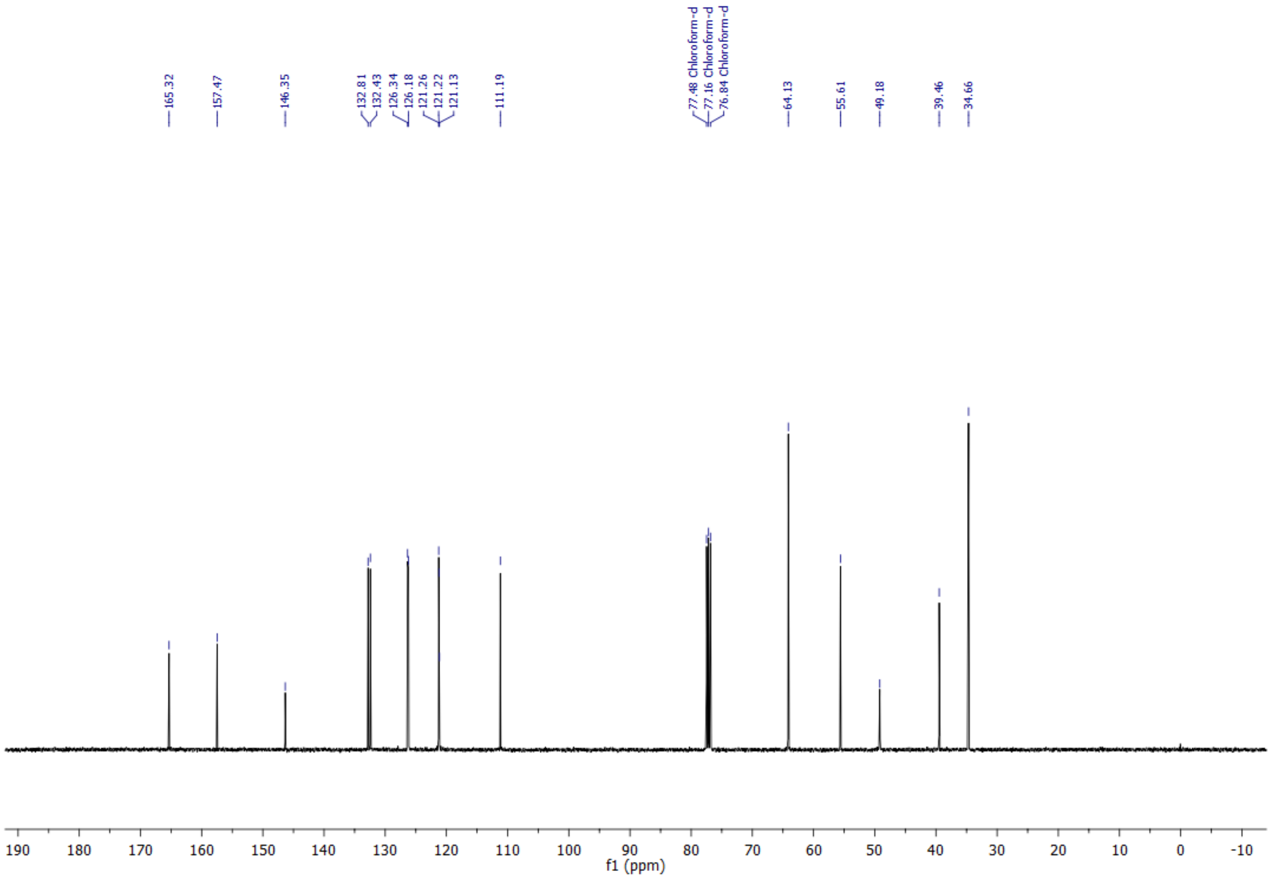

2,6-Dimethoxy-*N*-((4-(thiophen-3-yl)tetrahydro-2*H*-pyran-4-yl)methyl)benzamide (45)

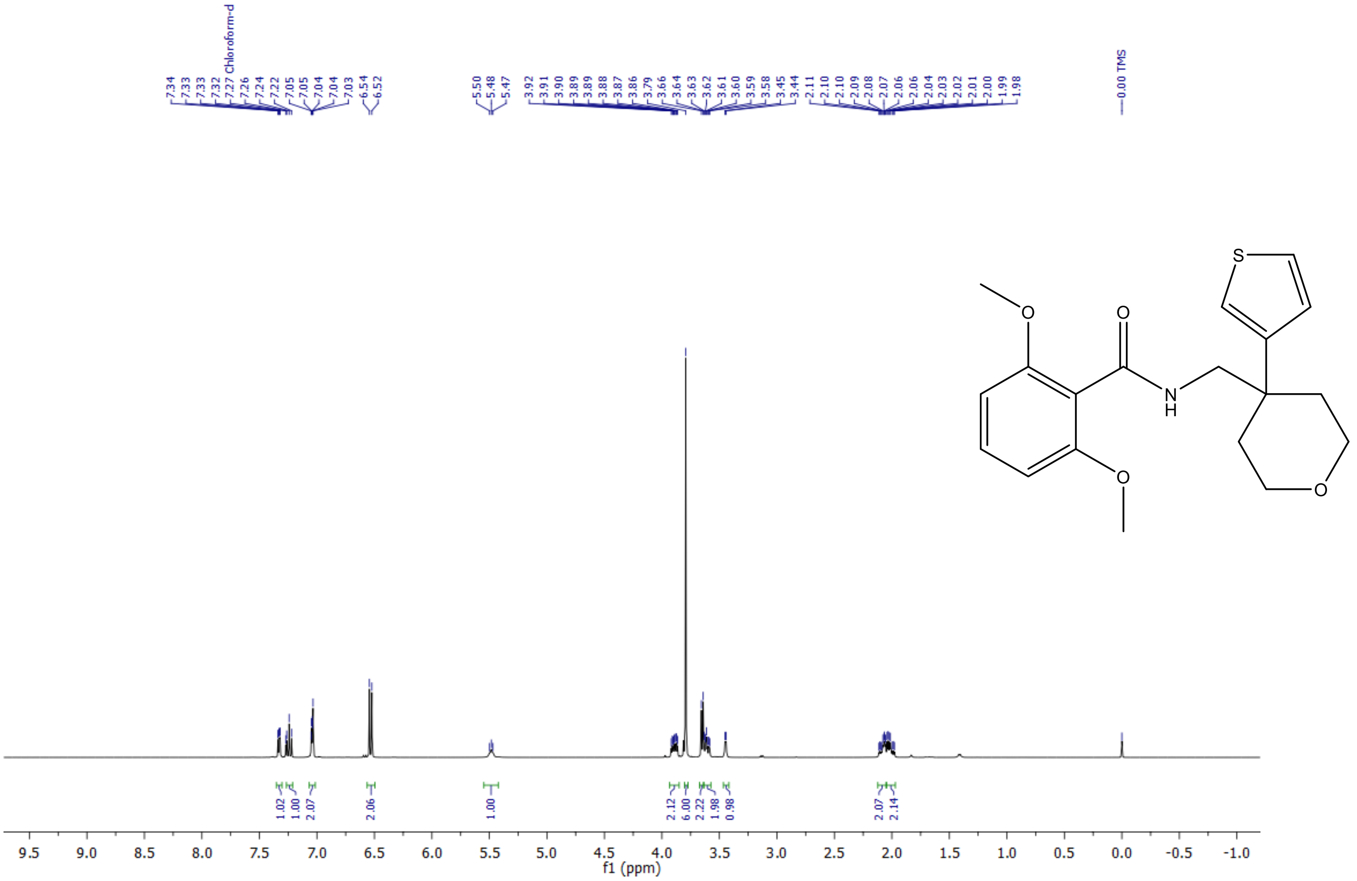

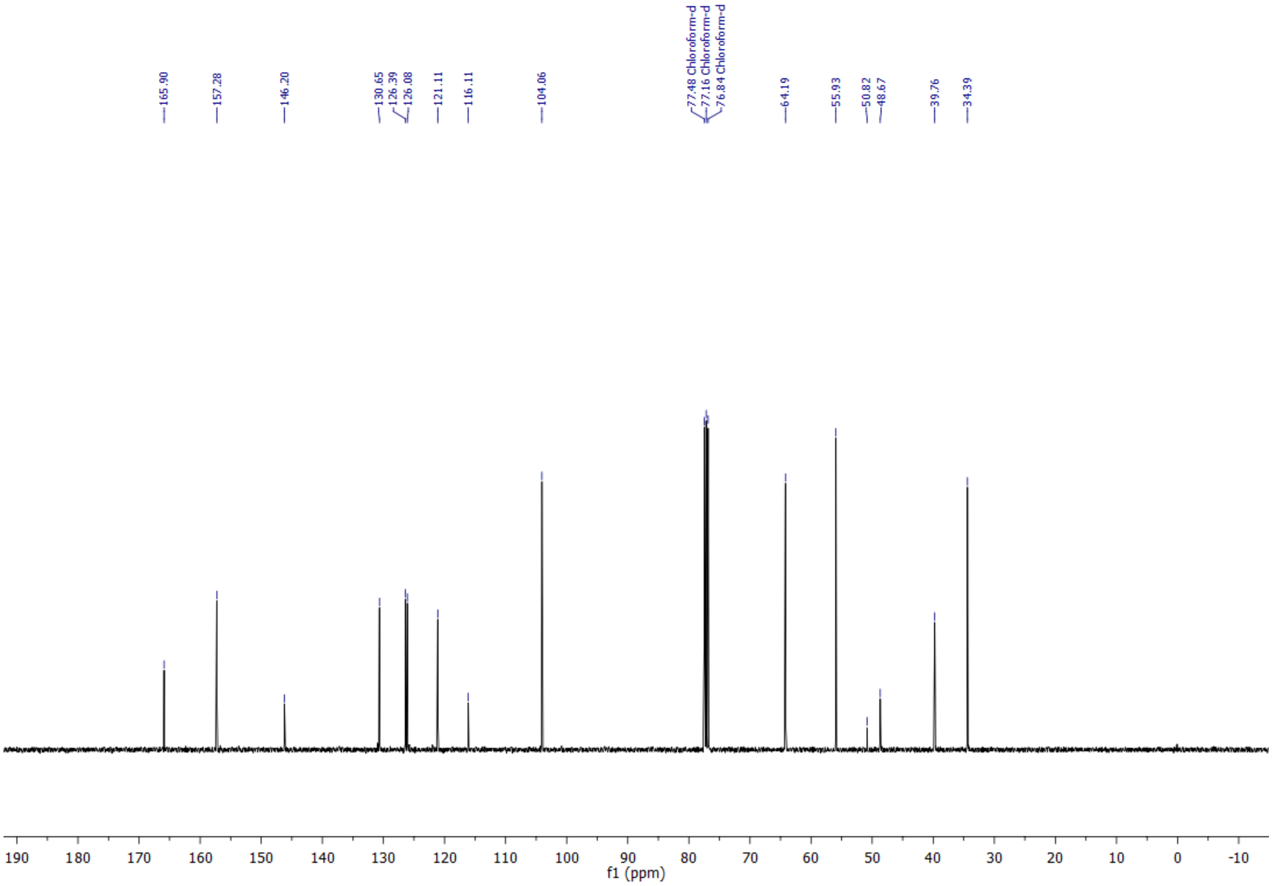

***N*-((4-(Thiophen-3-yl)tetrahydro-2*H*-pyran-4-yl)methyl)furan-3-carboxamide (46)**

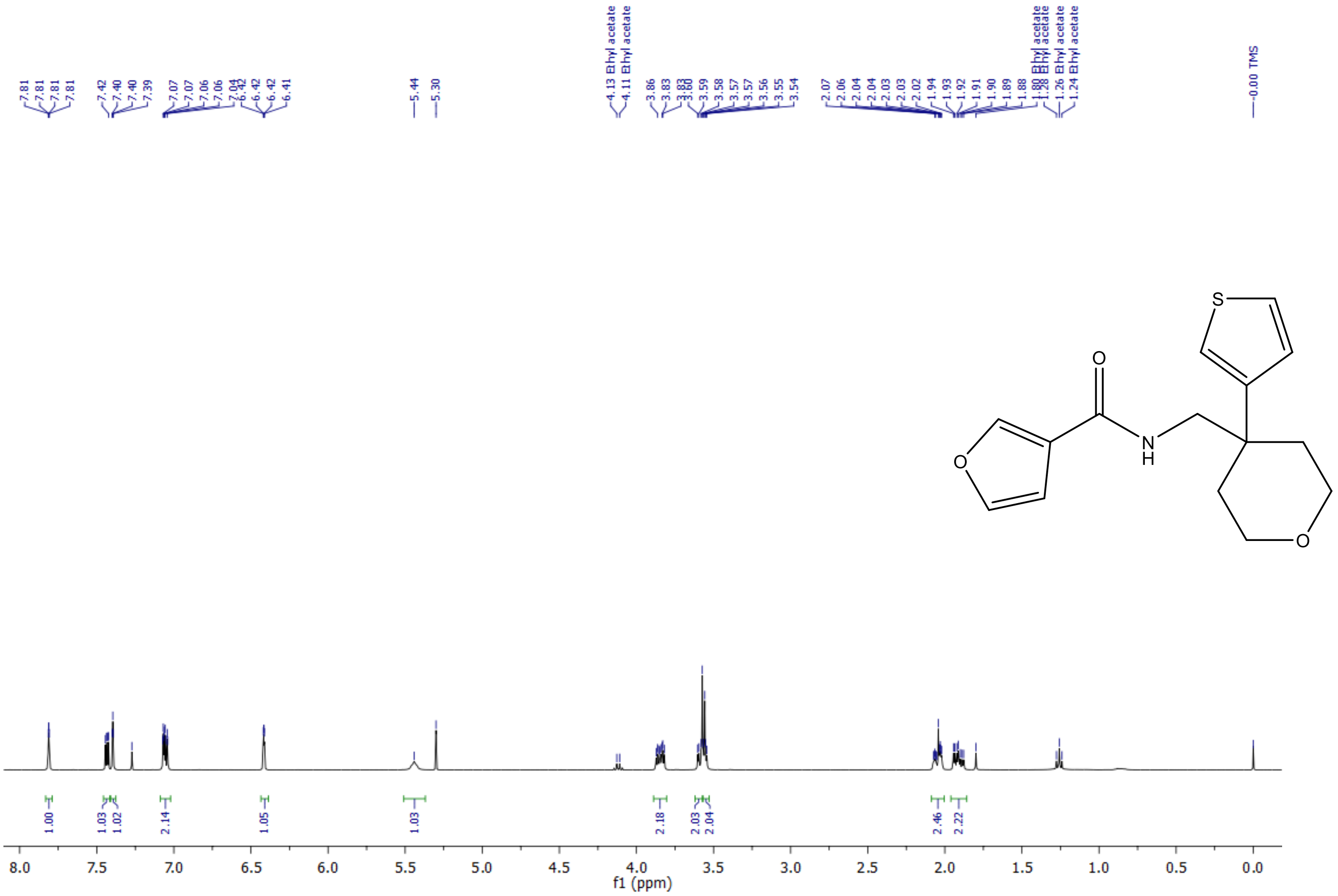

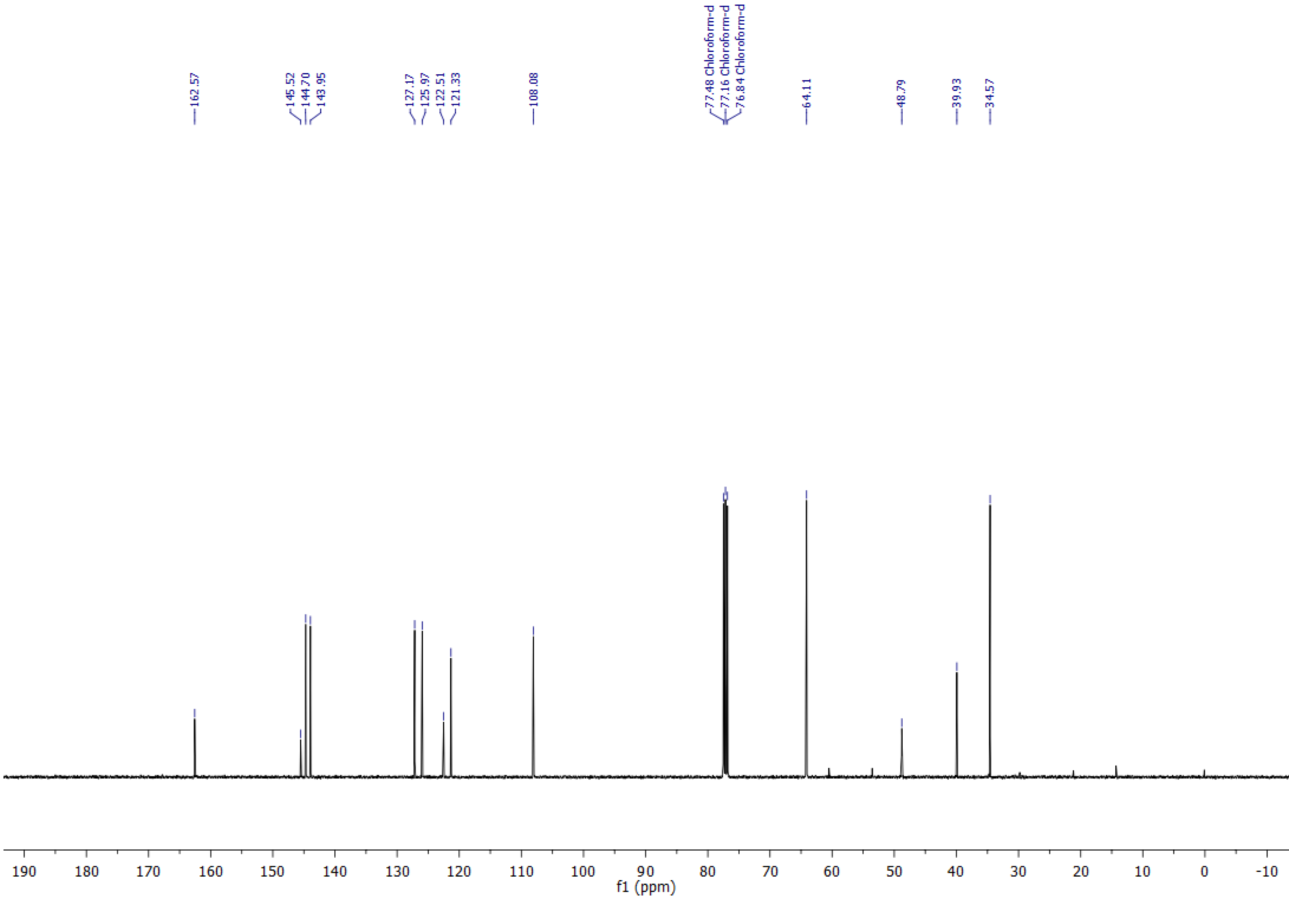

2-ethoxy-*N*-((4-(thiophen-3-yl)tetrahydro-2*H*-pyran-4-yl)methyl)benzamide (47)

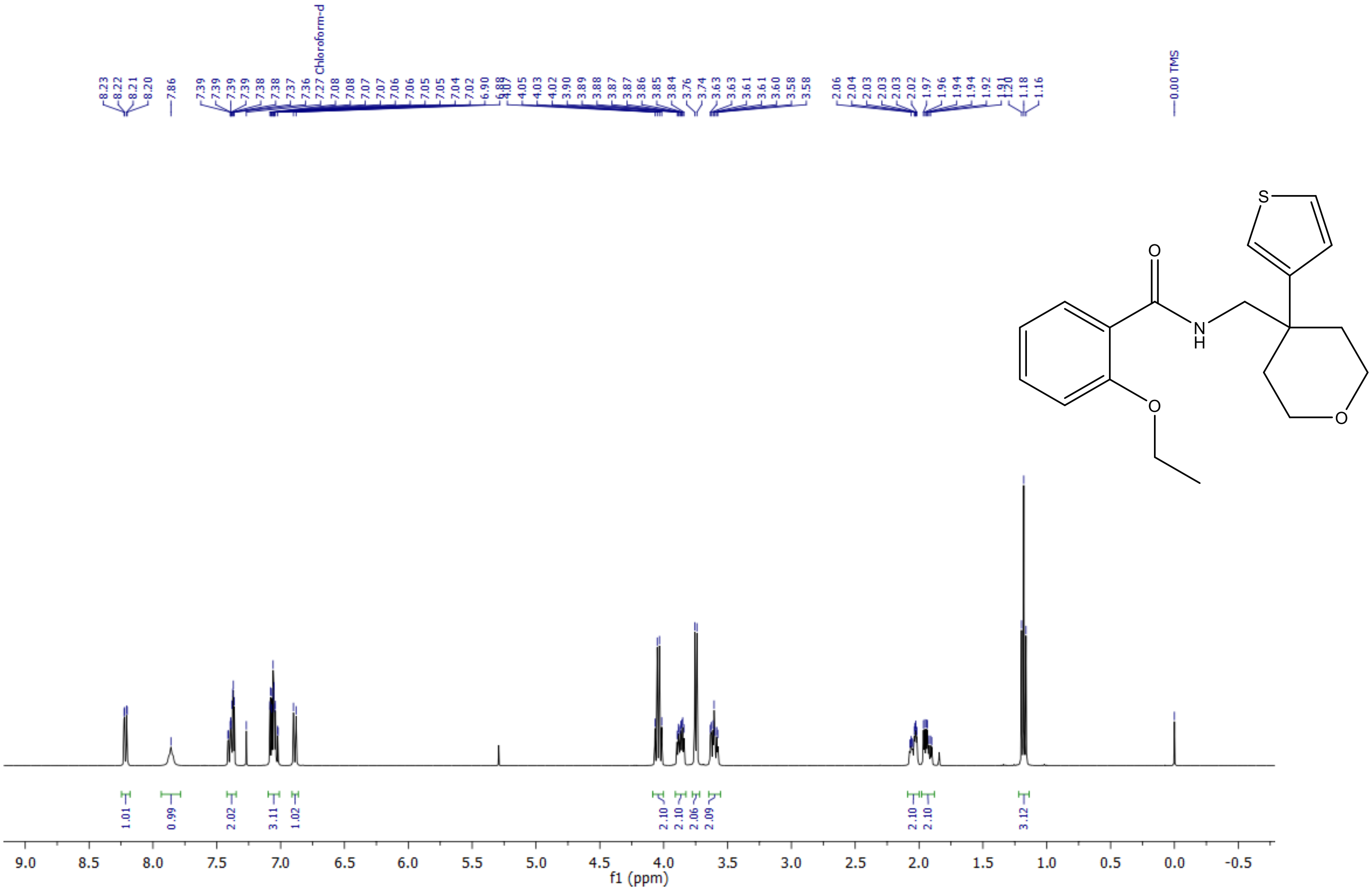

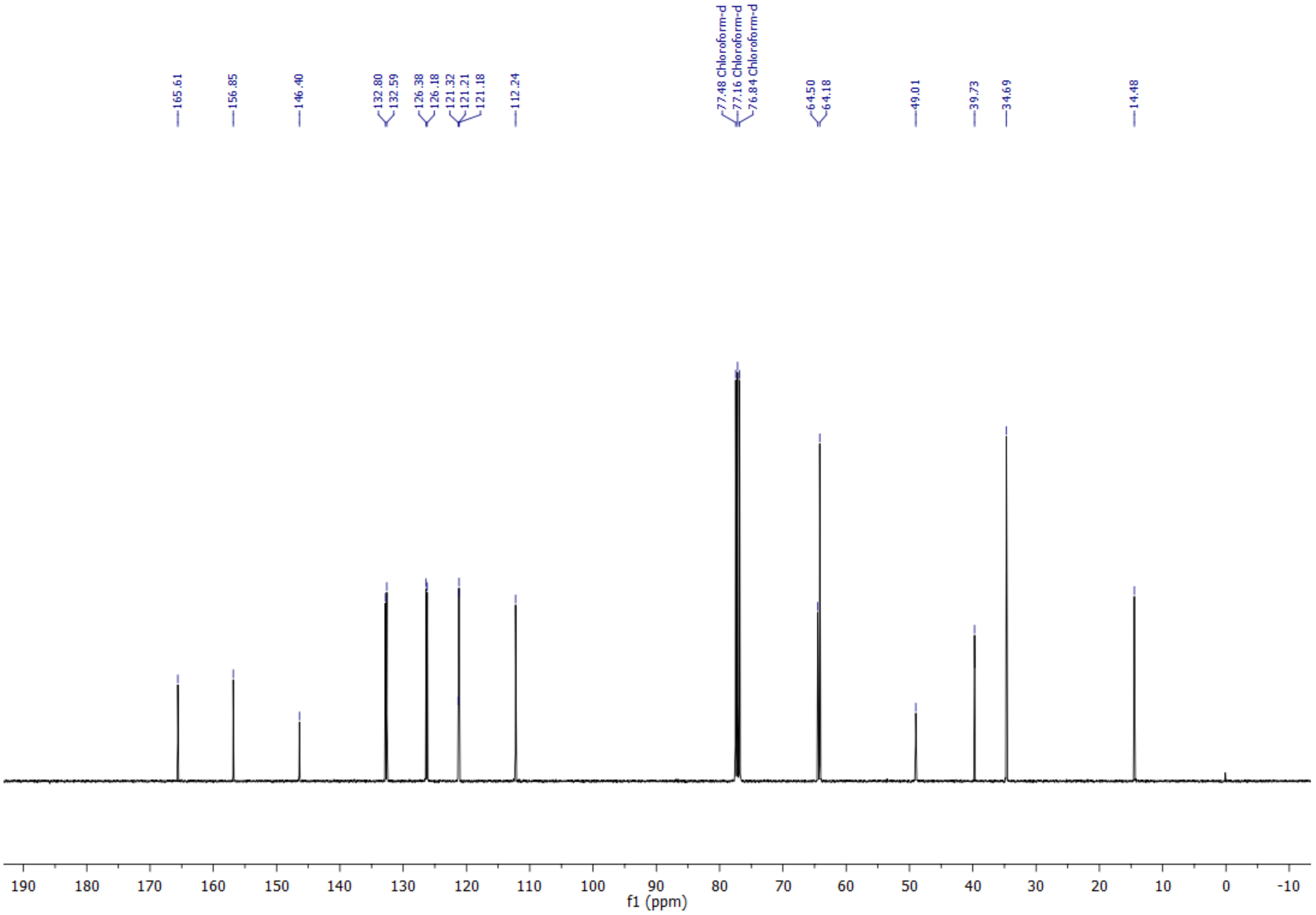

2-Methoxy-5-methyl-*N*-((4-(thiophen-3-yl)tetrahydro-2*H*-pyran-4-yl)methyl)benzamide (48)

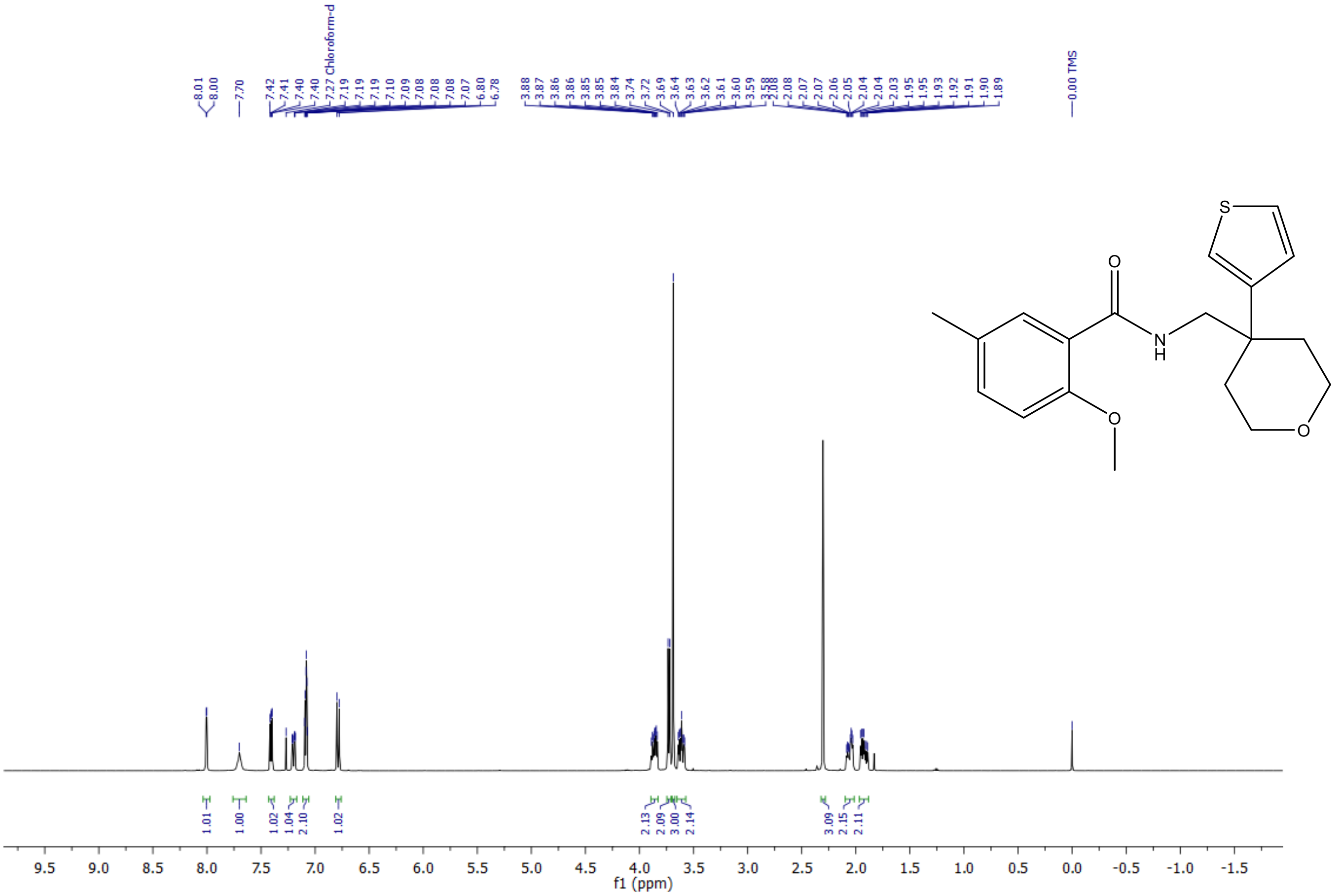

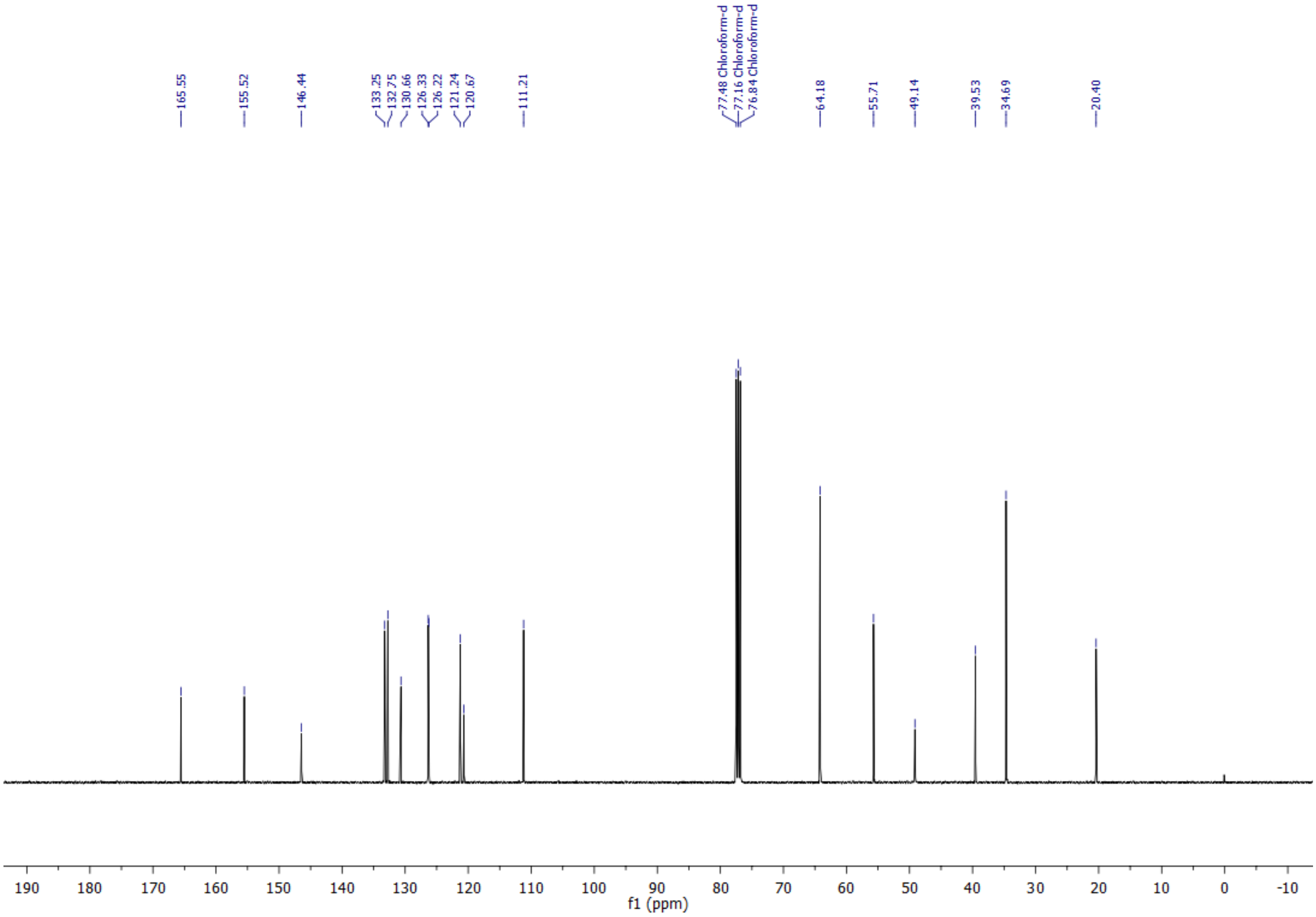

2-(2-Methoxyphenyl)-*N*-((4-(thiophen-3-yl)tetrahydro-2*H*-pyran-4-yl)methyl)acetamide (49)

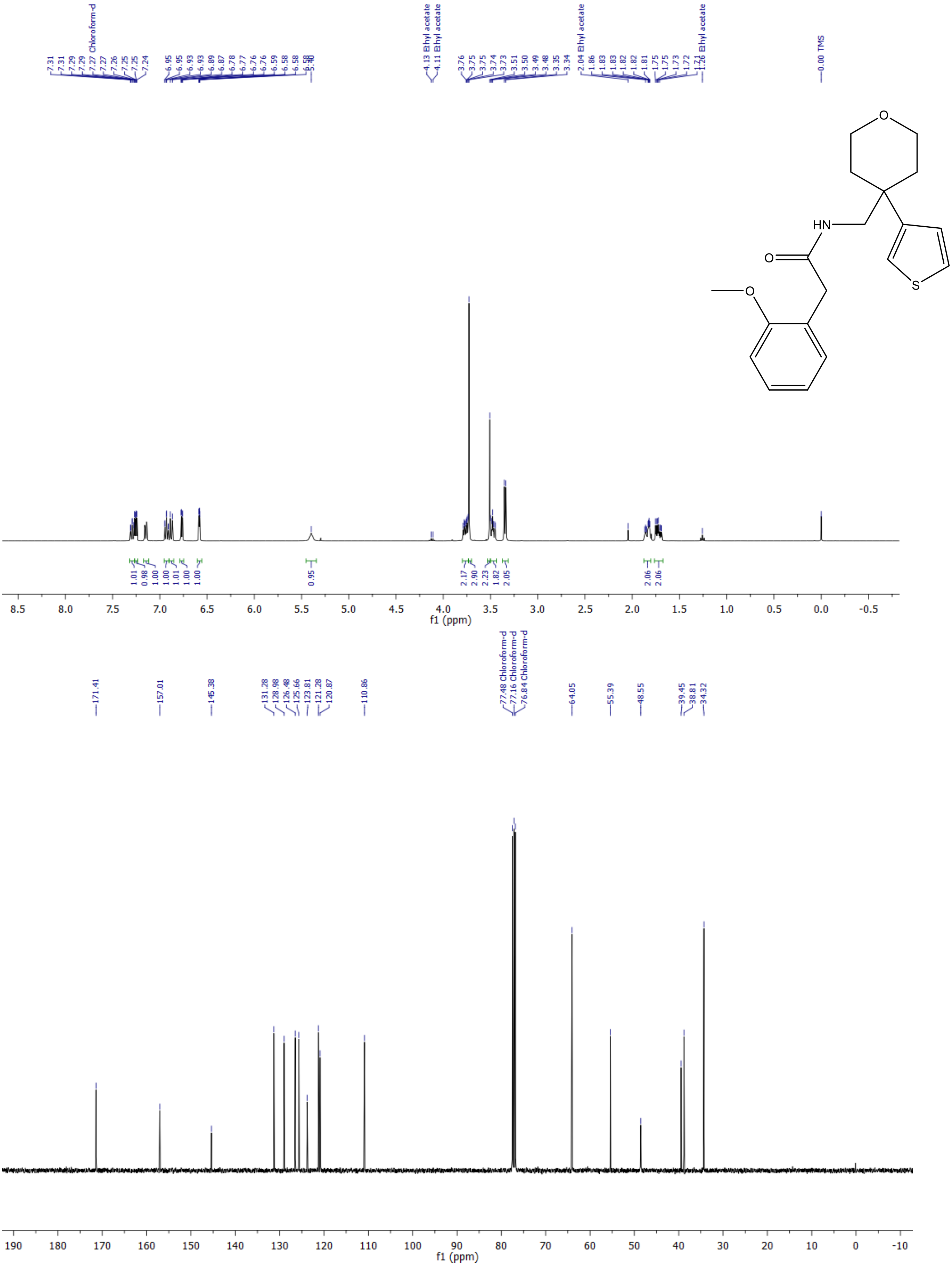

5-Fluoro-2-methoxy-*N*-((4-(thiophen-3-yl)tetrahydro-2*H*-pyran-4-yl)methyl)benzamide (50)

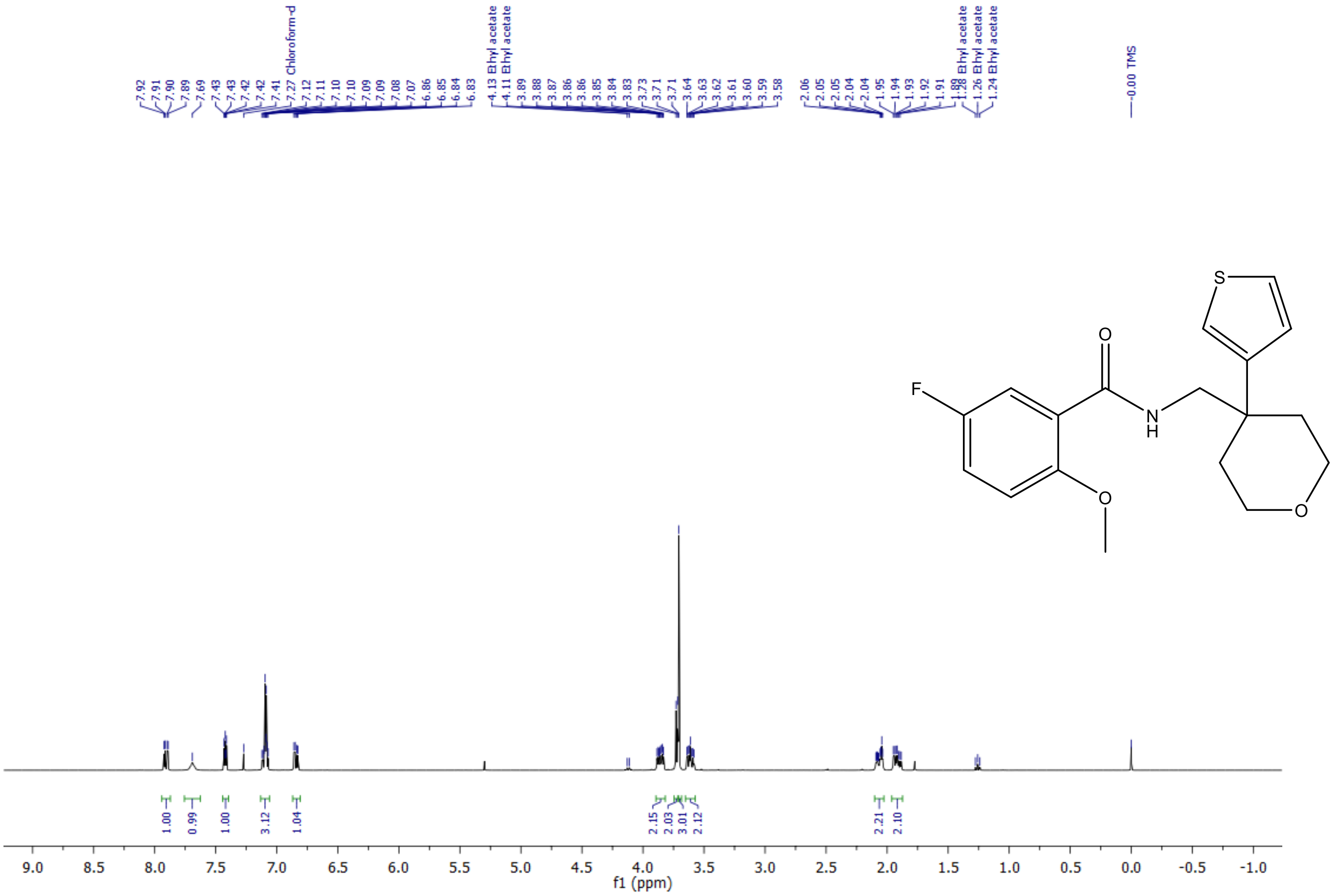

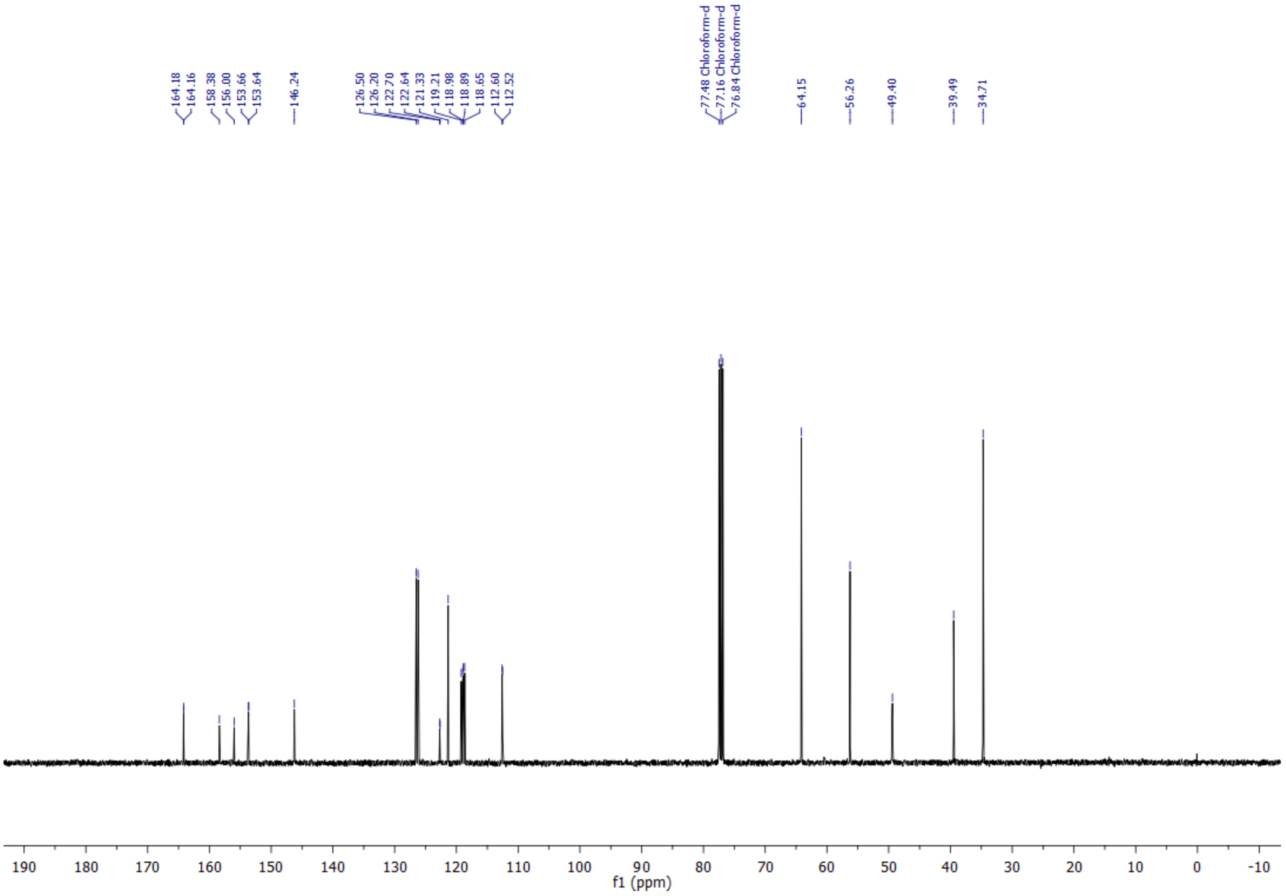

2-Methoxy-4-methyl-*N*-((4-(thiophen-3-yl)tetrahydro-2*H*-pyran-4-yl)methyl)benzamide (51)

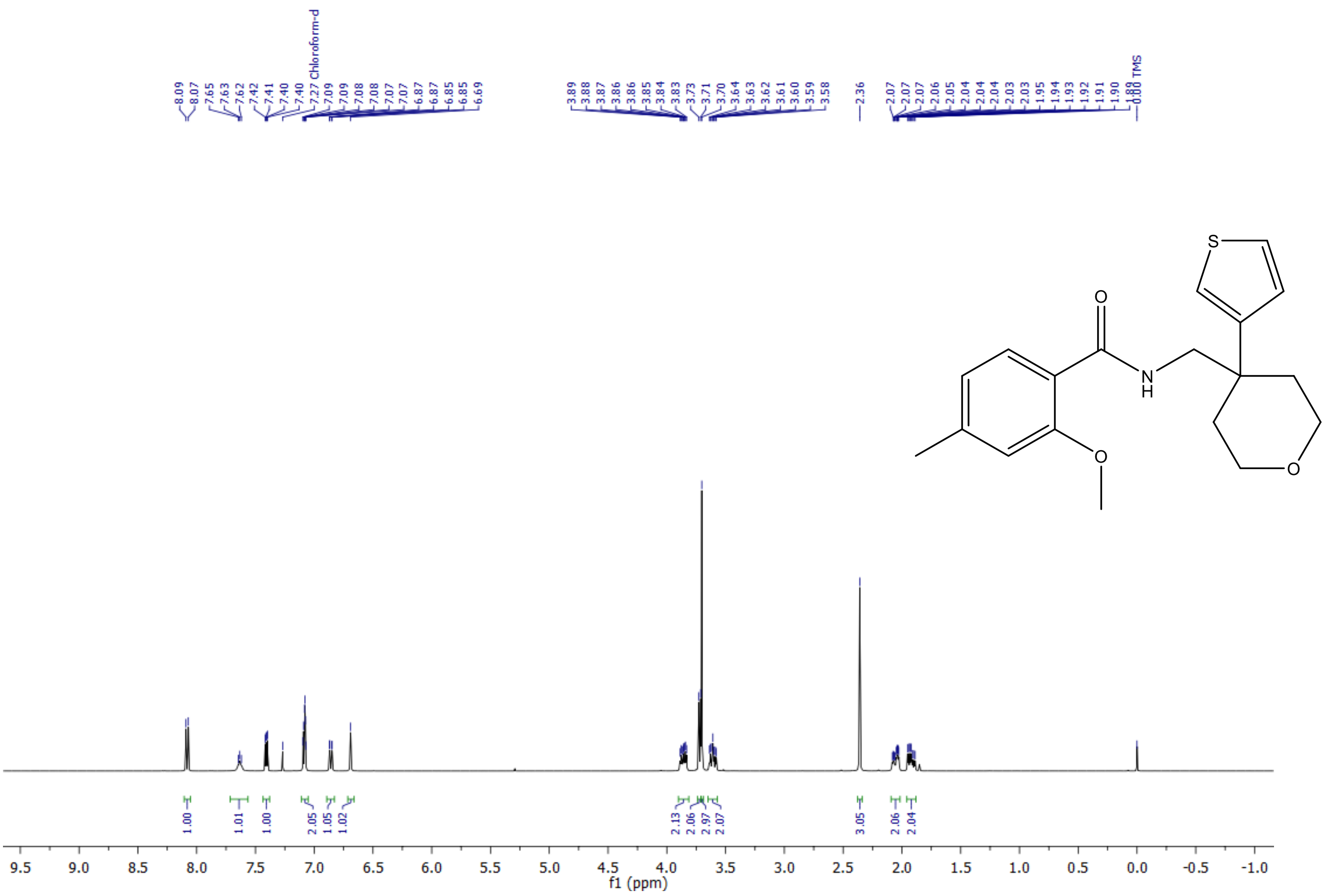

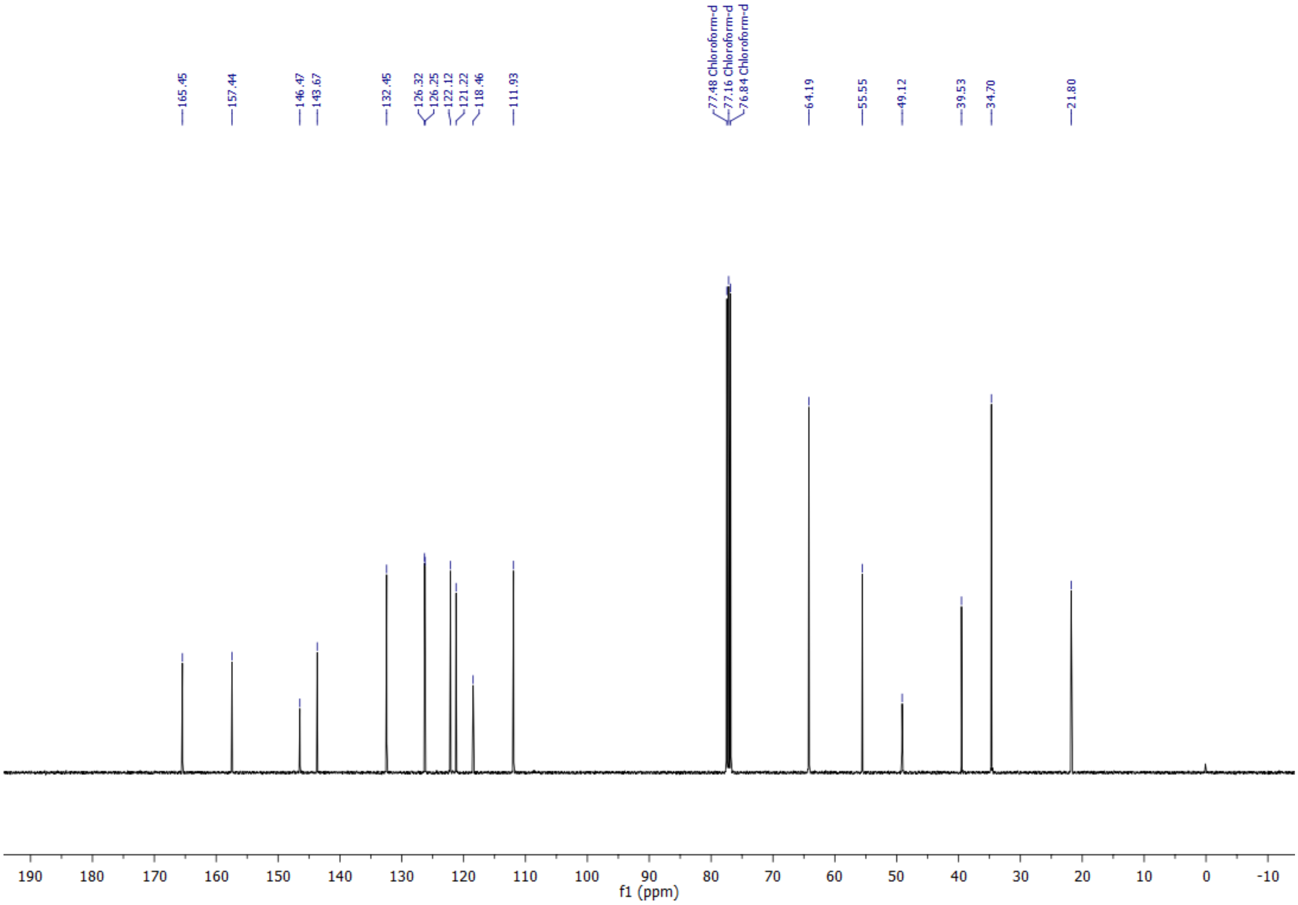

4-(Benzo[*b*]thiophen-3-yl)tetrahydro-2*H*-pyran-4-carbonitrile (104)

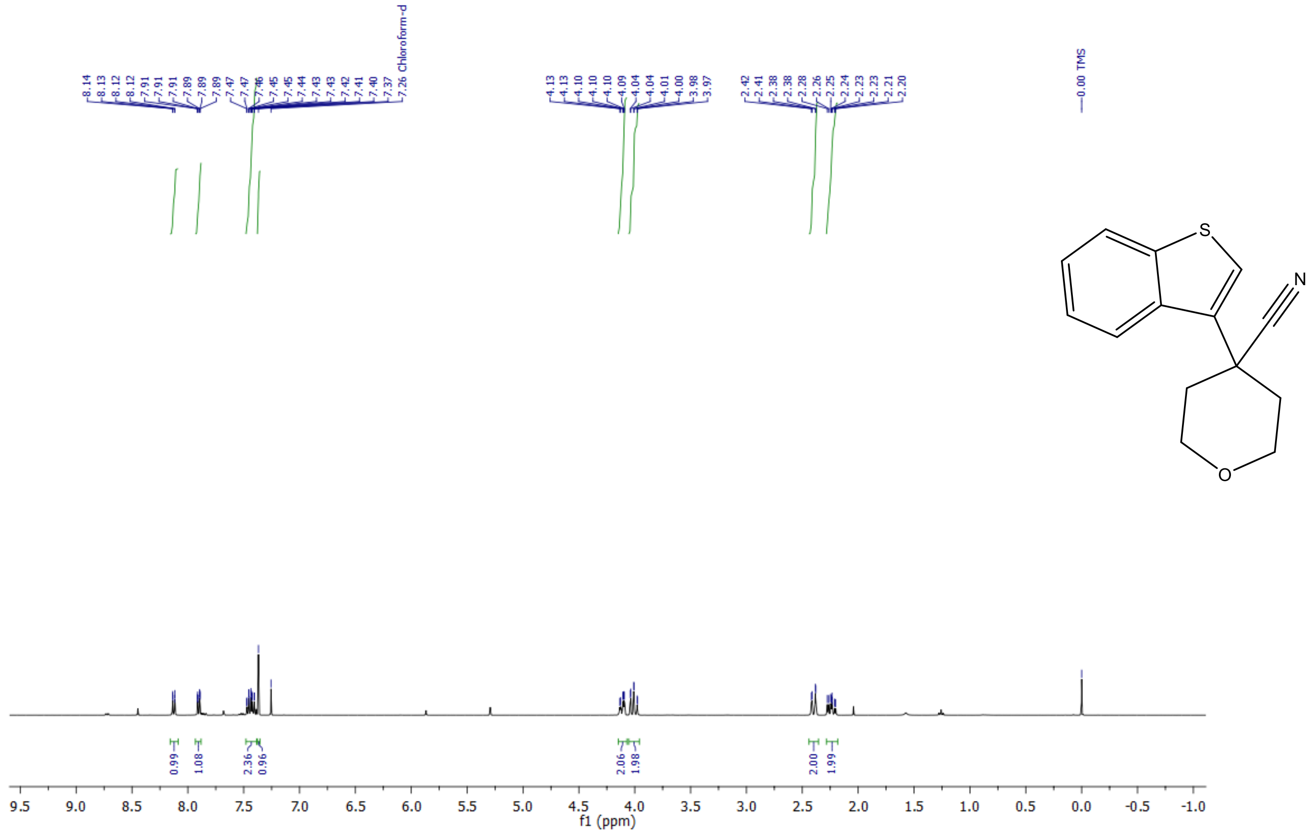

4-(Thiophen-3-yl)tetrahydro-2*H*-pyran-4-carbonitrile (105)

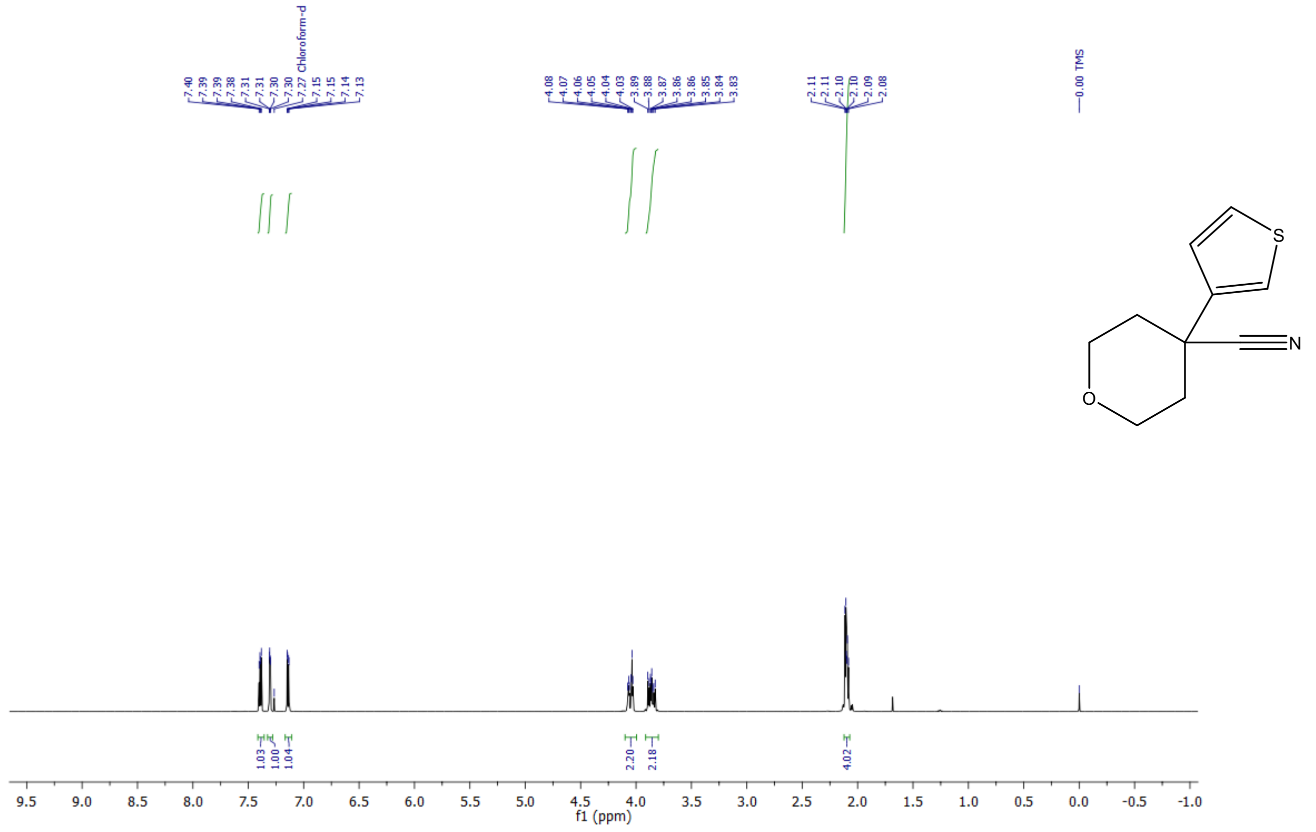

4-(4-(4-Methoxyphenyl)thiazol-2-yl)tetrahydro-2*H*-pyran-4-carbonitrile (106)

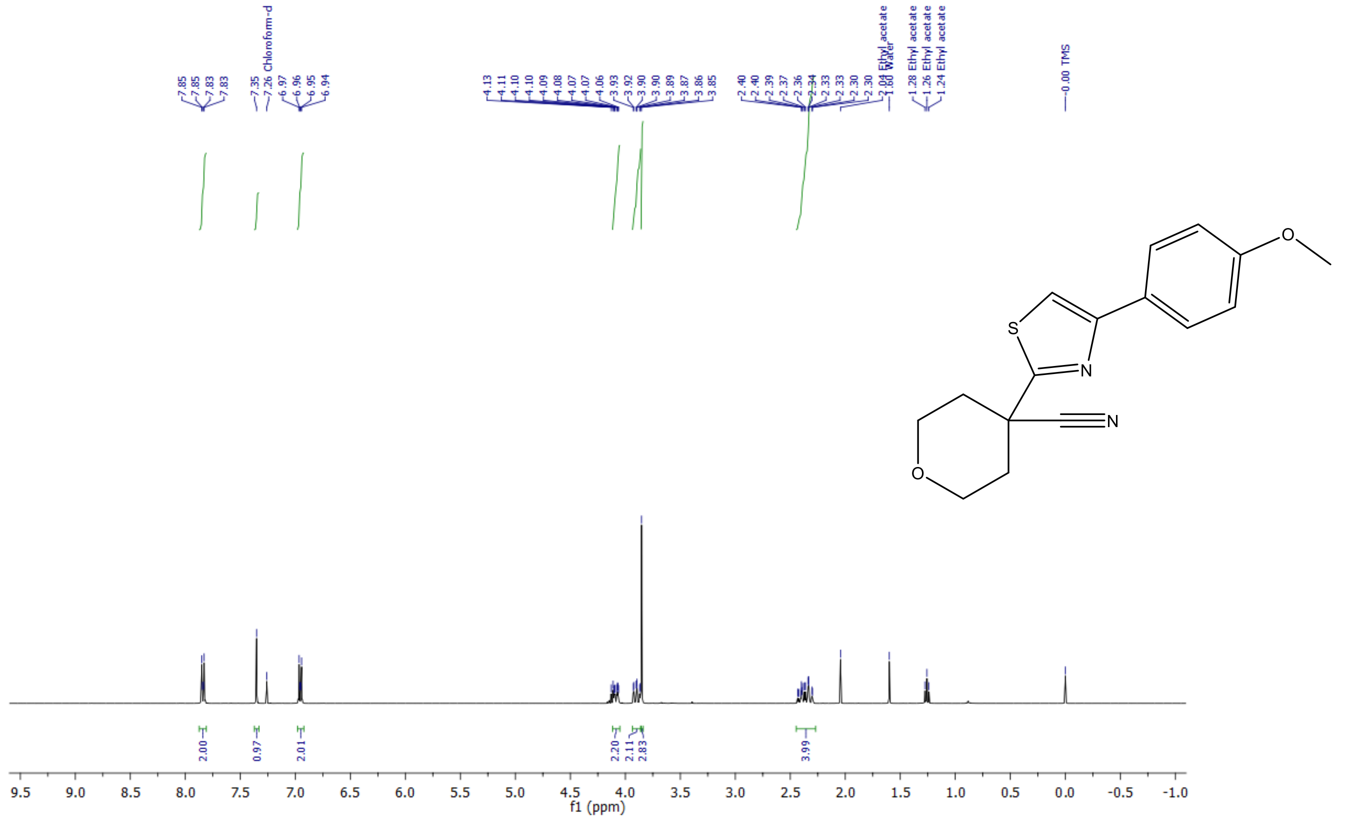

4-(4-Phenylthiazol-2-yl)tetrahydro-2H-pyran-4-carbonitrile (107)

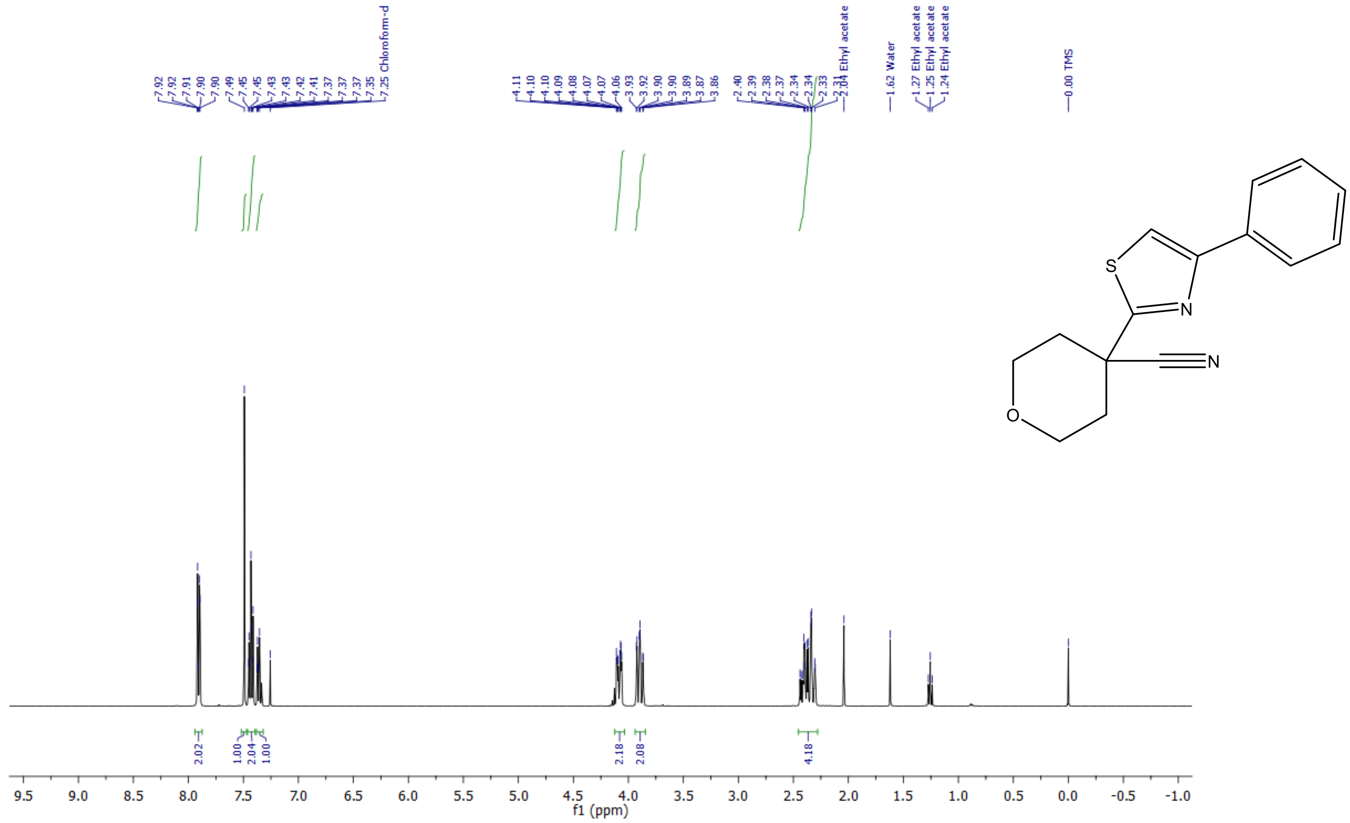

4-(2-Methylthiazol-4-yl)tetrahydro-2*H*-pyran-4-carbonitrile (108)

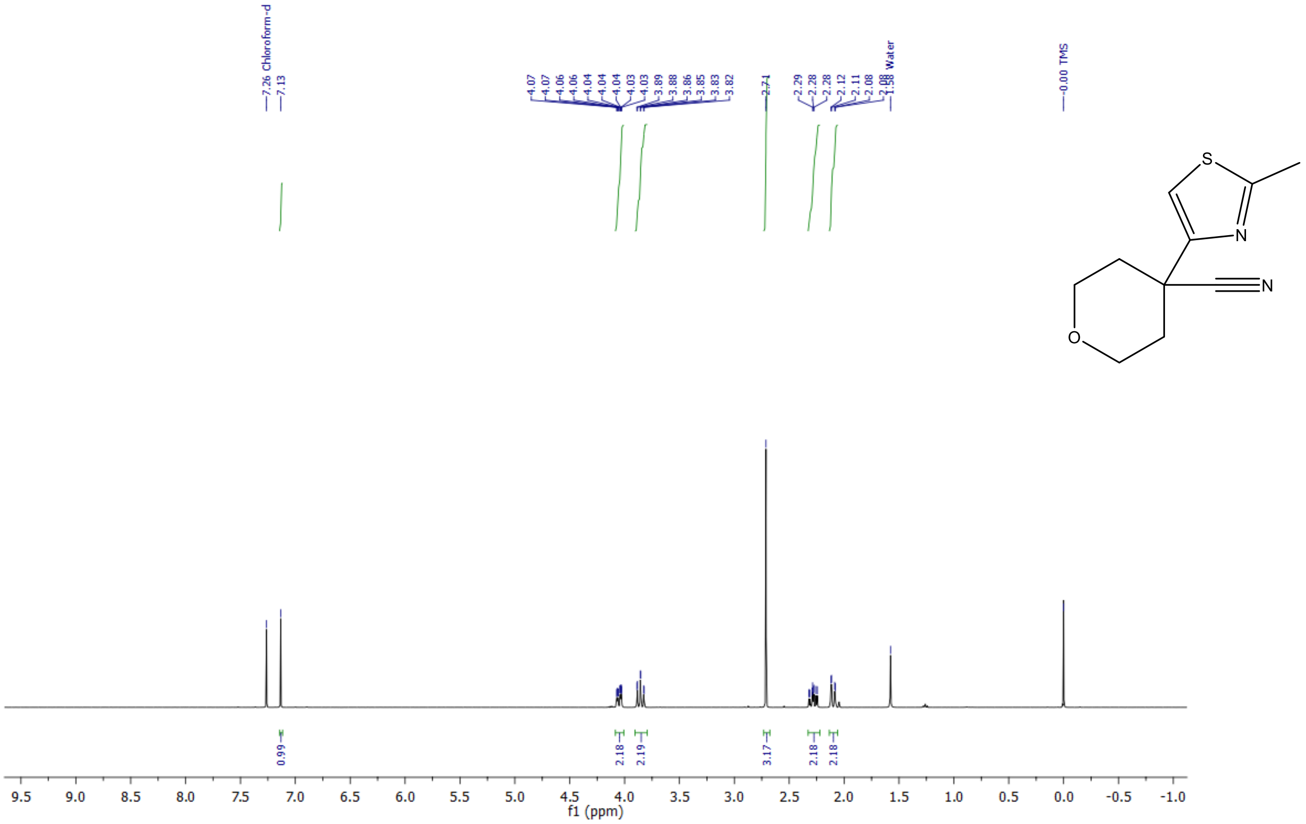

4-(Thiazol-4-yl)tetrahydro-2*H*-pyran-4-carbonitrile (109)

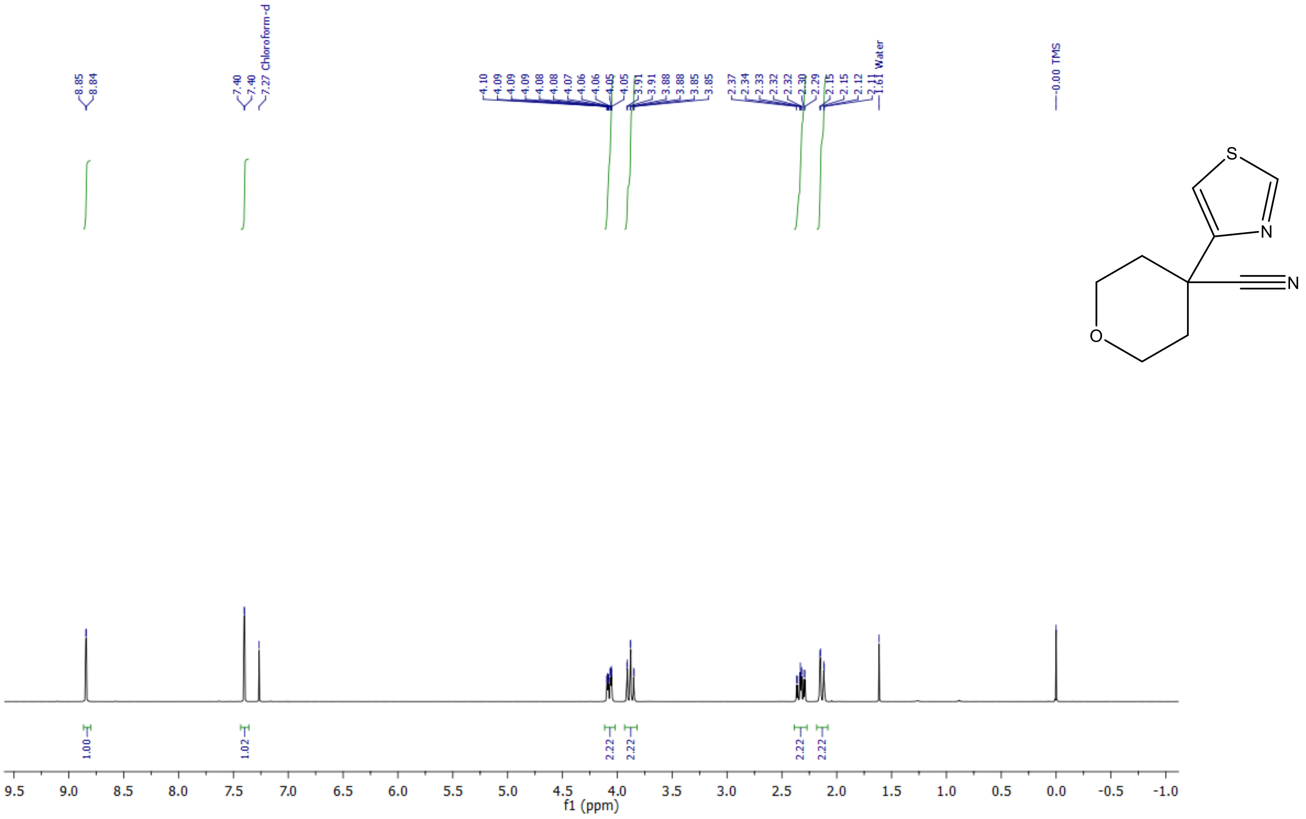

4-(Thiophen-2-yl)tetrahydro-2H-pyran-4-carbonitrile (110)

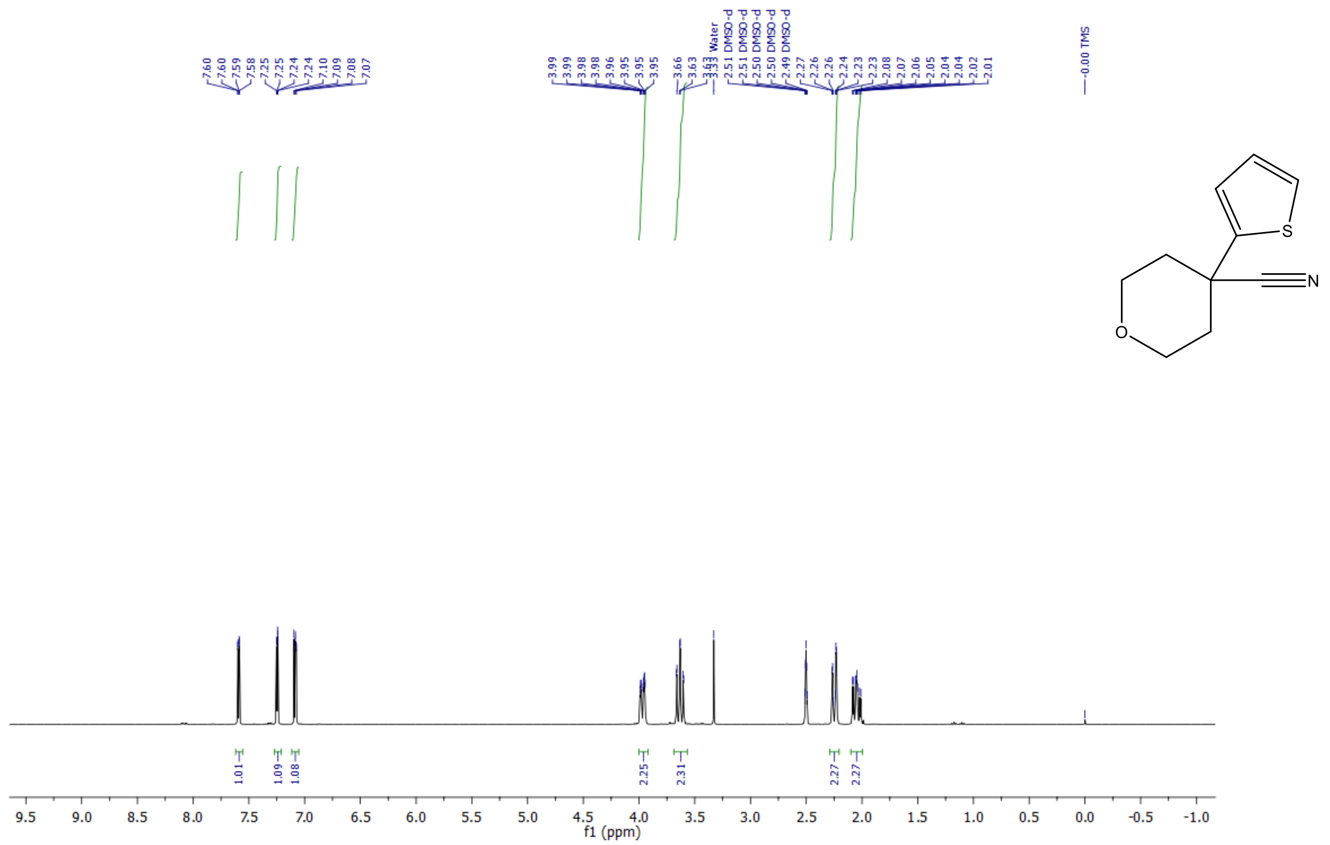

(4-(Benzo[*b*]thiophen-3-yl)tetrahydro-2*H*-pyran-4-yl)methanamine (111)

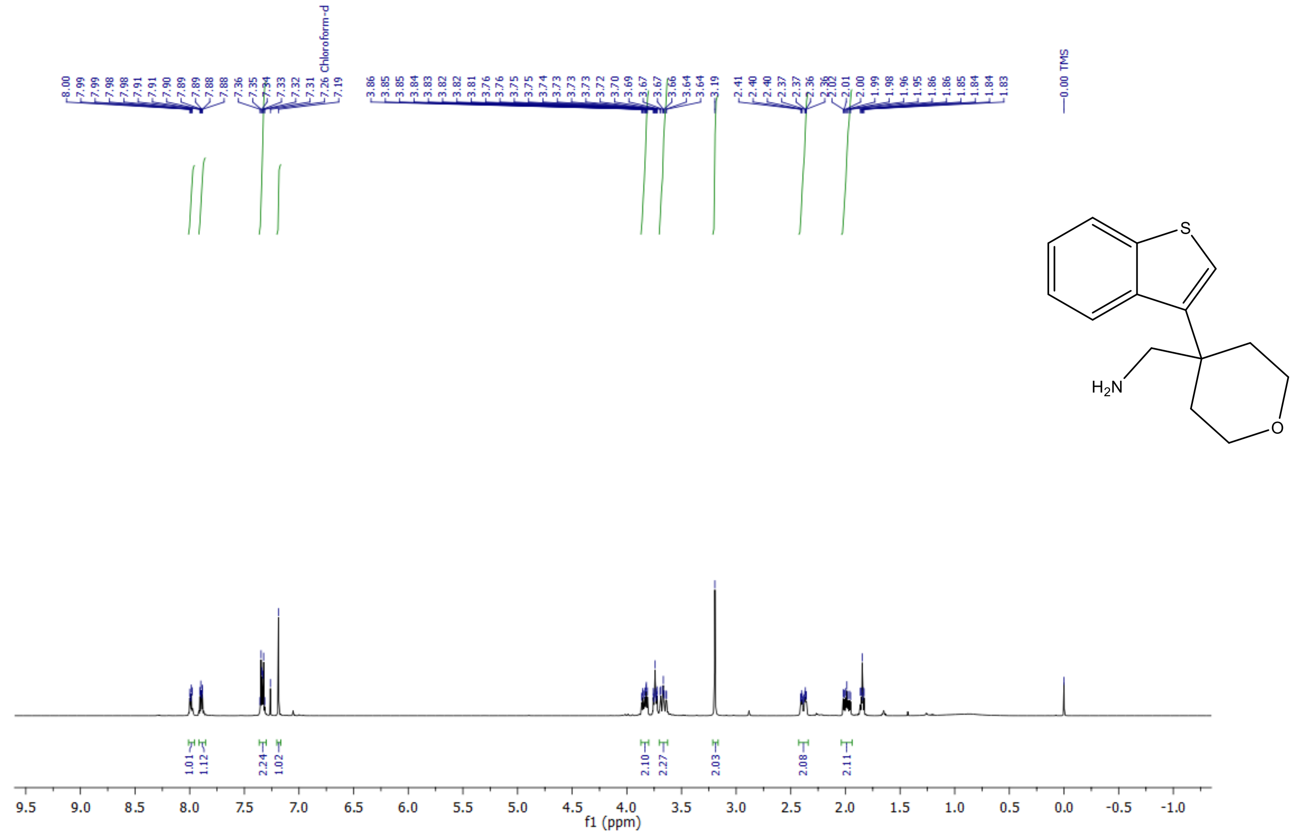

(4-(Thiophen-3-yl)tetrahydro-2H-pyran-4-yl)methanamine (112)

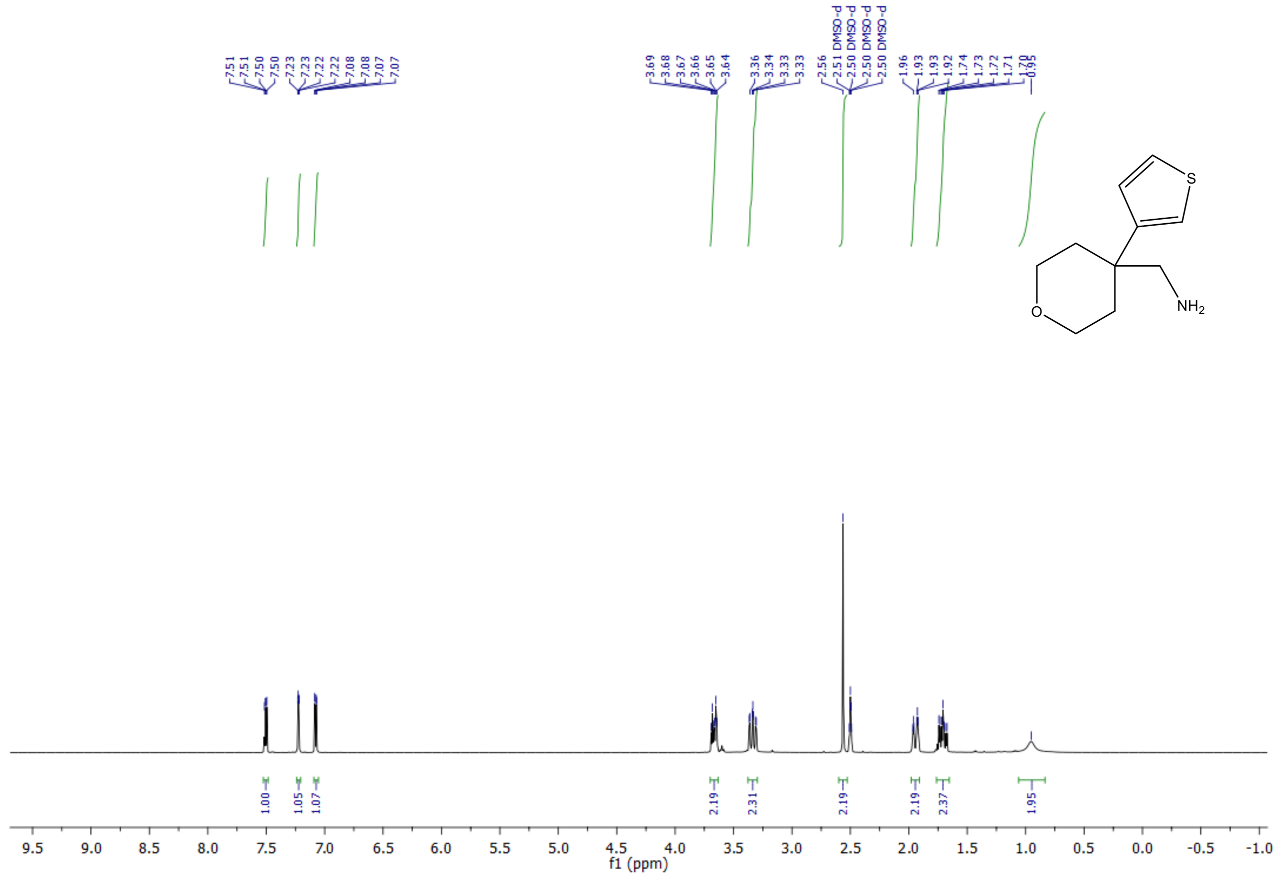

(4-(4-(4-Methoxyphenyl)thiazol-2-yl)tetrahydro-2*H*-pyran-4-yl)methanamine (113)

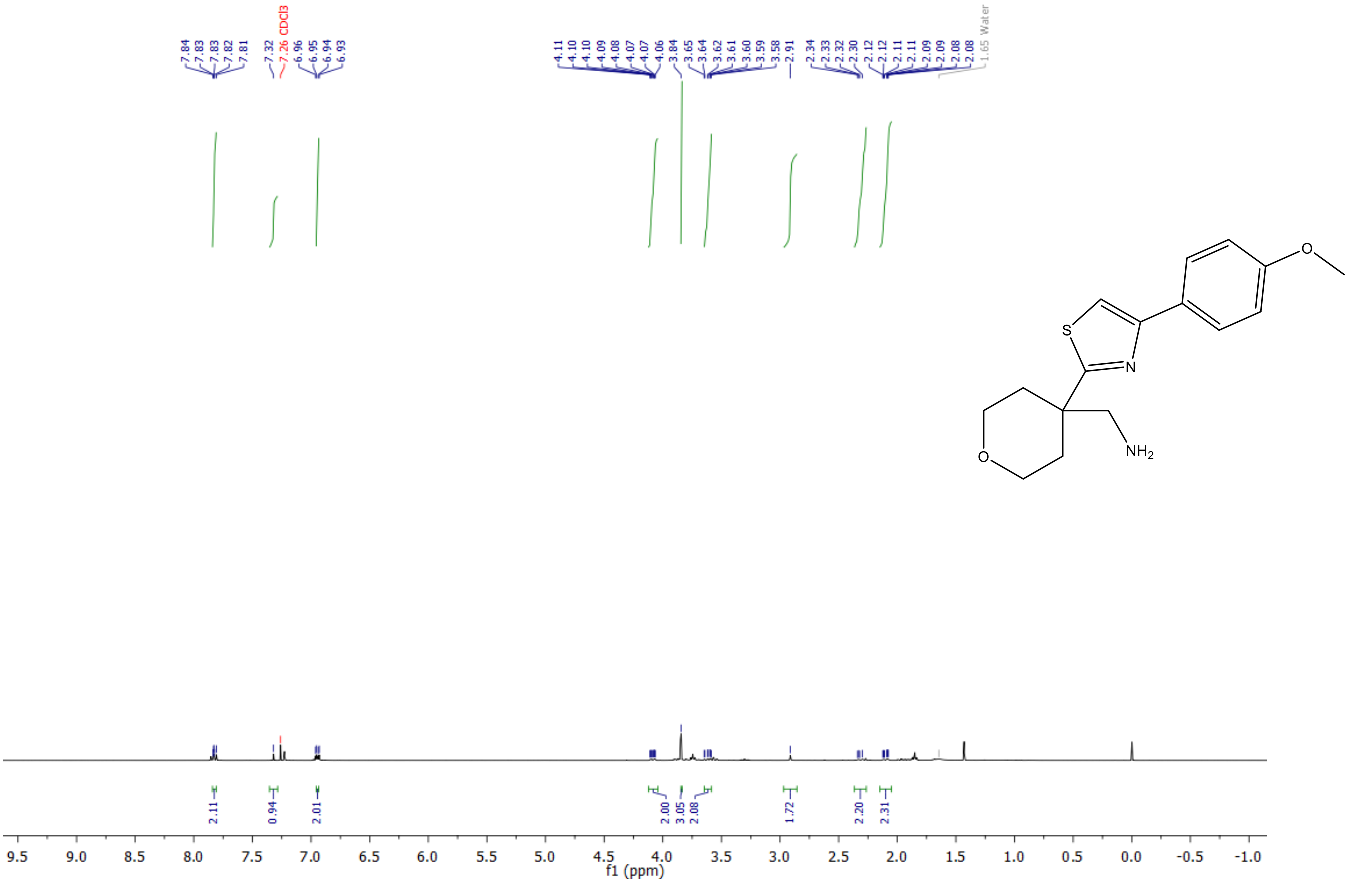

(4-(4-Phenylthiazol-2-yl)tetrahydro-2H-pyran-4-yl)methanamine (114)

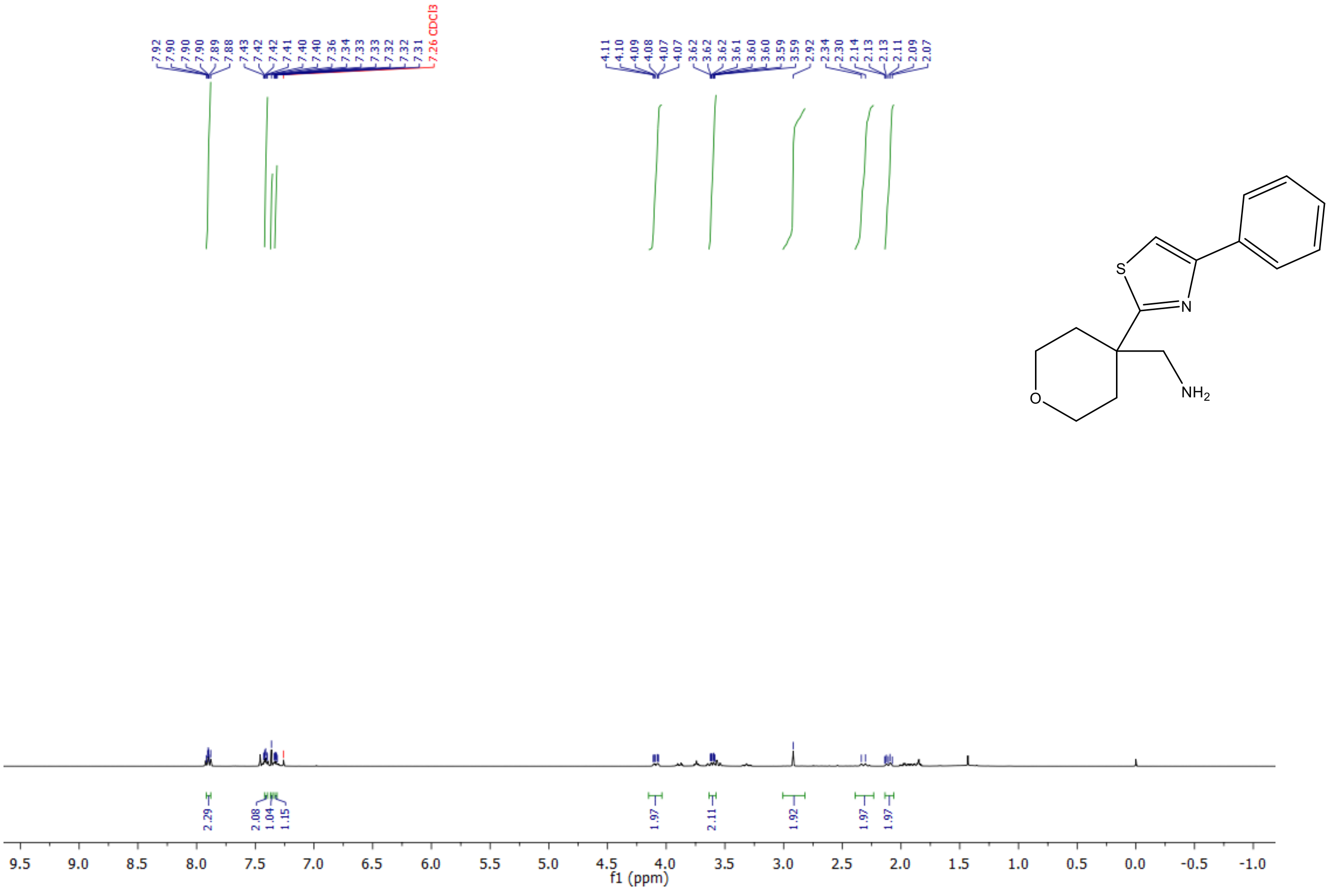

(4-(2-Methylthiazol-4-yl)tetrahydro-2*H*-pyran-4-yl)methanamine (115)

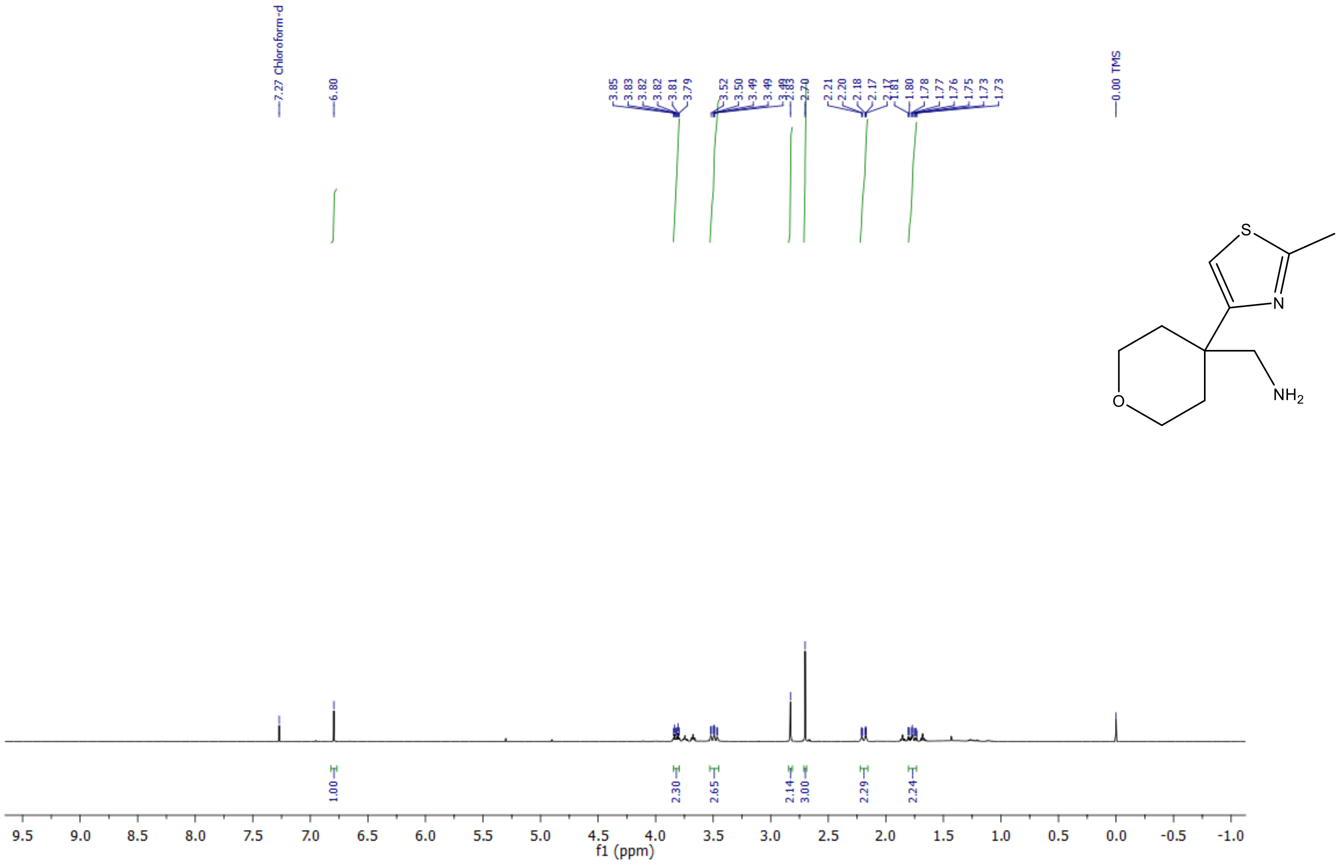

(4-(Thiazol-4-yl)tetrahydro-2*H*-pyran-4-yl)methanamine (116)

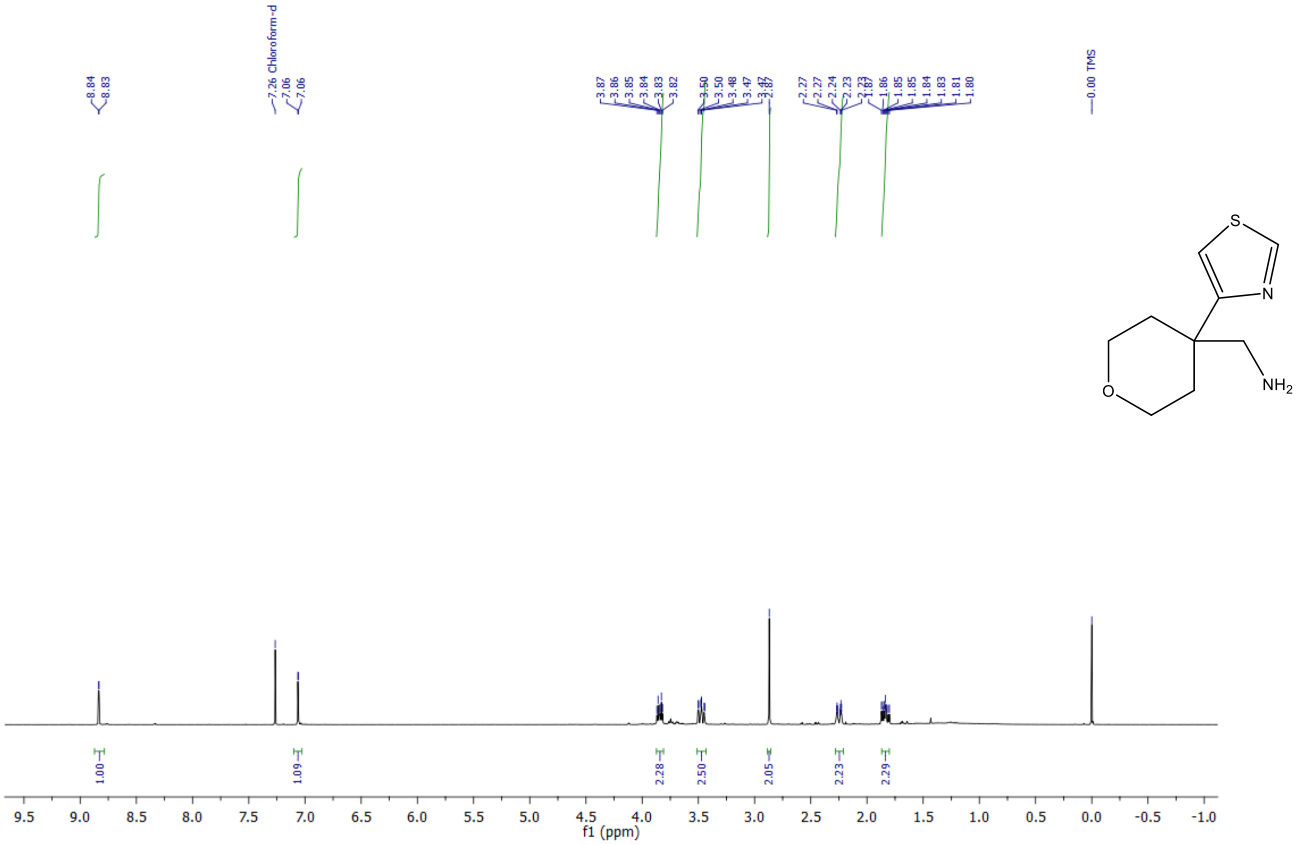

(4-(Thiophen-2-yl)tetrahydro-2H-pyran-4-yl)methanamine (117)

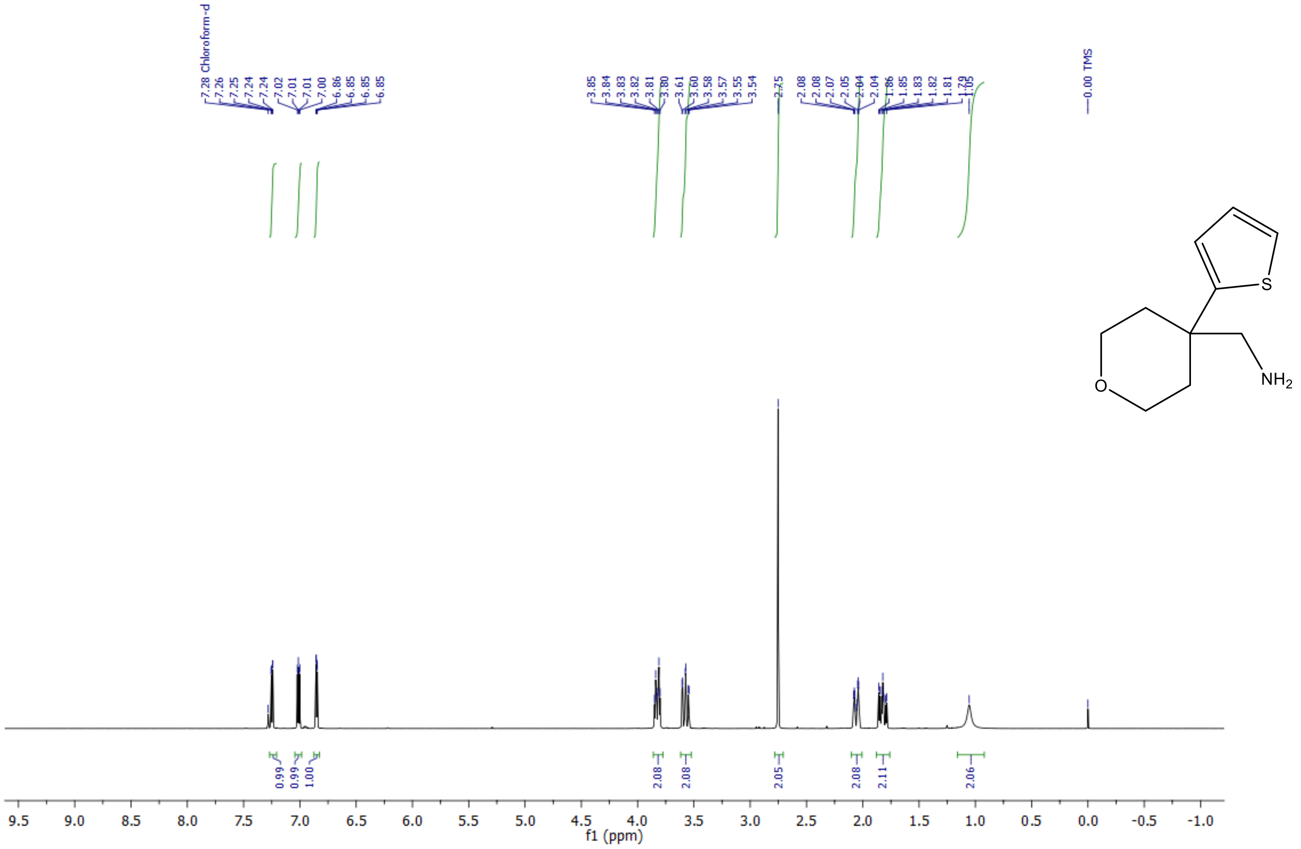

2-(4-(4-Methoxyphenyl)thiazol-2-yl)acetonitrile (118)

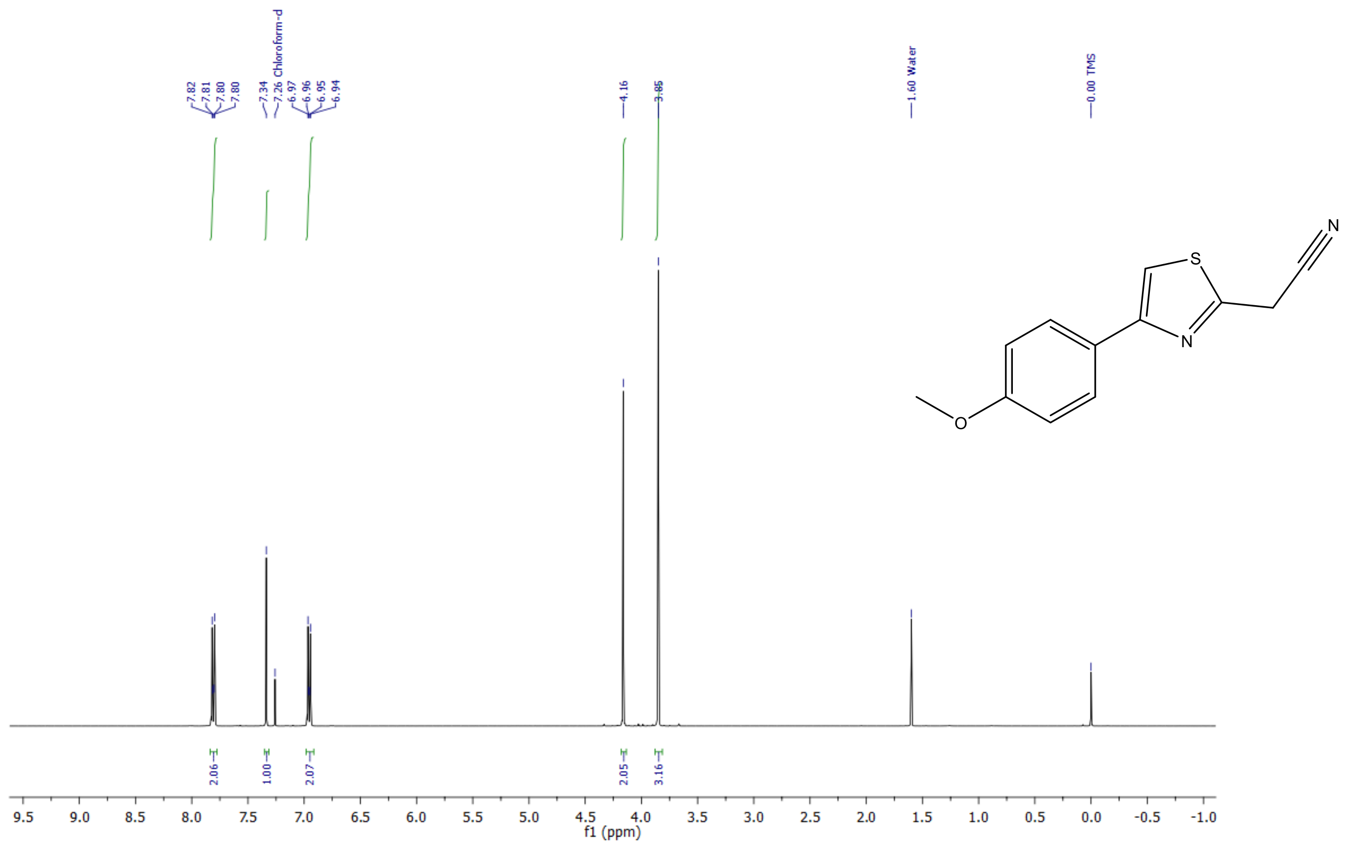

2-(4-Phenylthiazol-2-yl)acetonitrile (119)

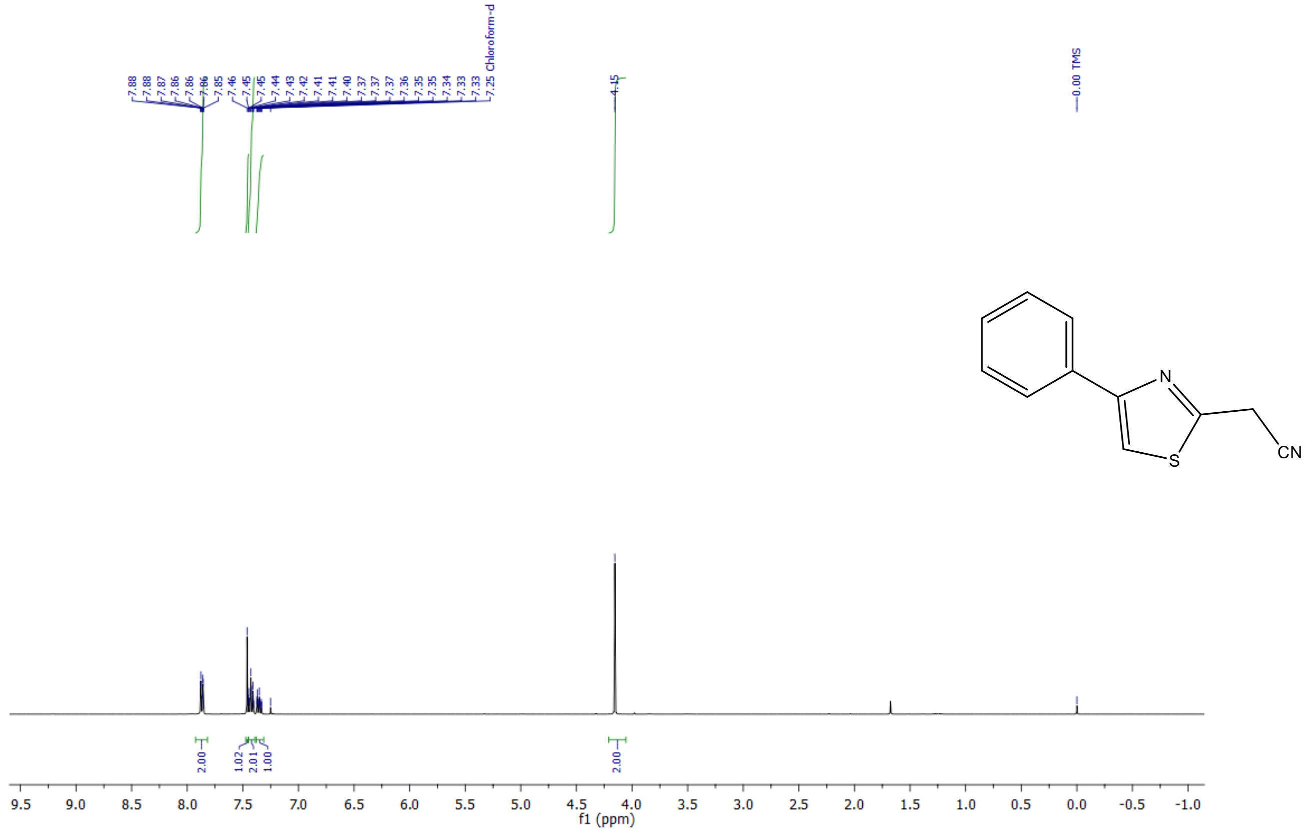

Supplement: Supplementary file 1 [file cancers-14-02595-s001.zip › cancers-1720154-supplementary.pdf]
